# An Enantioselective Suzuki–Miyaura Coupling To Form Axially Chiral Biphenols

**DOI:** 10.1021/jacs.2c06529

**Published:** 2022-08-15

**Authors:** Robert Pearce-Higgins, Larissa N. Hogenhout, Philip J. Docherty, David M. Whalley, Padon Chuentragool, Najung Lee, Nelson Y. S. Lam, Thomas M. McGuire, Damien Valette, Robert J. Phipps

**Affiliations:** † Yusuf Hamied Department of Chemistry, University of Cambridge, Lensfield Road, Cambridge CB2 1EW, United Kingdom; ‡ Oncology R&D, AstraZeneca, Cambridge CB4 0WG, United Kingdom; § GlaxoSmithKline Medicines Research Centre, Stevenage, Hertfordshire SG1 2NY, United Kingdom

## Abstract

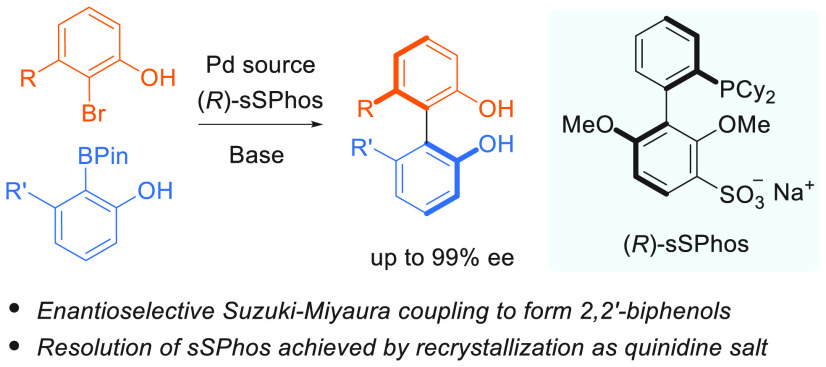

Axial chirality features prominently in molecules of biological interest as well as chiral catalyst designs, and atropisomeric 2,2′-biphenols are particularly prevalent. Atroposelective metal-catalyzed cross-coupling is an attractive and modular approach to access enantioenriched biphenols, and yet existing protocols cannot achieve this directly. We address this challenge through the use of enantiopure, sulfonated **SPhos** (**sSPhos**), an existing ligand that has until now been used only in racemic form and that derives its chirality from an atropisomeric axis that is introduced through sulfonation. We believe that attractive noncovalent interactions involving the ligand sulfonate group are responsible for the high levels of asymmetric induction that we obtain in the 2,2′-biphenol products of Suzuki–Miyaura coupling, and we have developed a highly practical resolution of **sSPhos** via diastereomeric salt recrystallization.

Transition metal-catalyzed cross-coupling has revolutionized the synthesis of biaryl compounds, and the Suzuki–Miyaura coupling has arguably had the greatest impact.^1^ Extensive ligand development has made it feasible to form increasingly hindered biaryl bonds, which often lead to axial chirality. Atropisomeric compounds can have dramatically different biological activities and have become an important feature in medicinal chemistry,^2^ so development of methodology that can control stereochemistry during their synthesis is extremely important. The development of enantioselective variants of the Suzuki–Miyaura reaction is the obvious approach. Seminal examples were reported in 2000,^3^ building on pioneering studies relating to asymmetric cross-coupling of Grignard reagents.^4^ In the ensuing decades, substantial efforts have been directed towards the development of new chiral ligands that can expand the scope of enantioselective Suzuki–Miyaura couplings.^5,6^ While tremendous advances have been made, it is notable that almost all protocols require either one or both substrate partners to possess an extended π system, typically in the form of a naphthalene ring, leading to either phenylnaphthyl or binaphthyl products, respectively (Figure 1A). Barring a few isolated examples, there is only a single report, from Tang and co-workers, which delivers high (>90% ee) selectivity in biphenyl-type products.^6s^ Although an important advance, it does, in most cases, require formyl and methoxy substituents to be present adjacent to the biphenyl bond on both partners, constraining synthetic utilization. The dearth of examples for obtaining enantioenriched biphenyls illustrates that this remains a largely unsolved problem in asymmetric cross-coupling chemistry.

**Figure 1:**
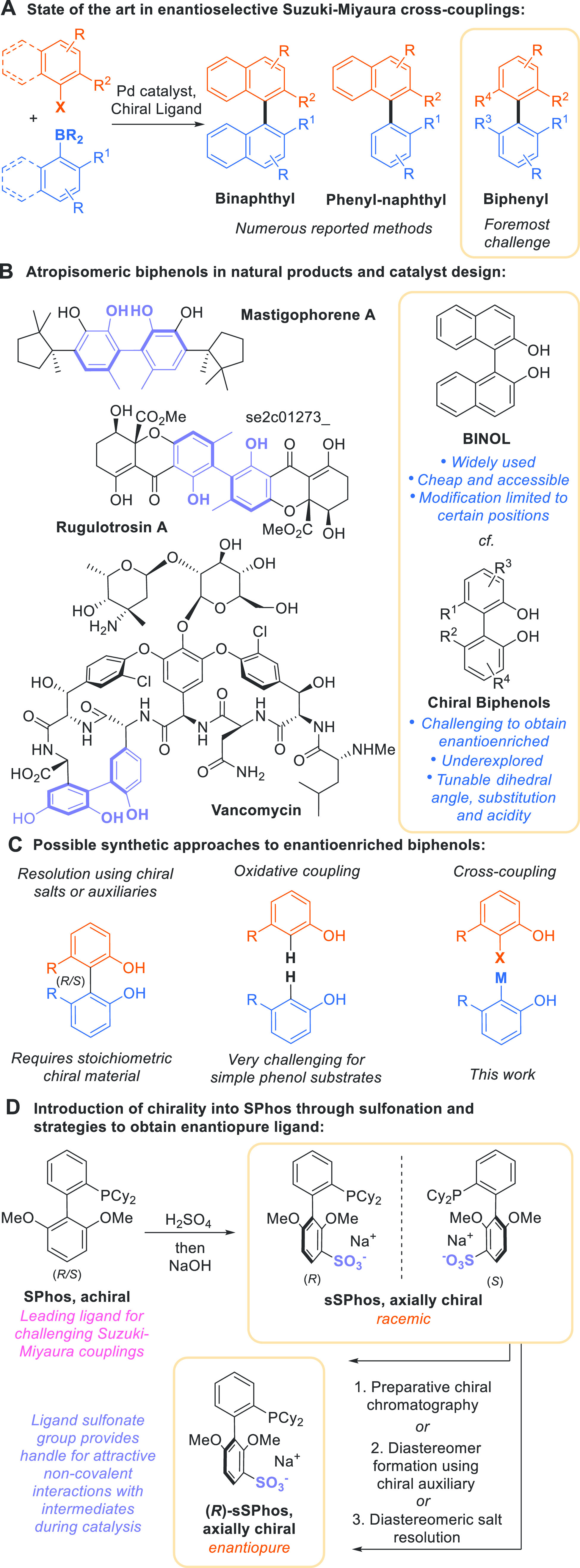
Background, 2,2′-biphenols and **sSPhos**.

One of the most prominent classes of atropisomeric biphenyl compounds are the 2,2′-biphenols. Axially chiral 2,2′-biphenols occur extensively in natural products, such as vancomycin, rugulotrosin A, and mastigophorene A.^7^ They also have numerous potential applications in catalyst and ligand design but remain underexplored in favor of readily available BINOL,^8^ despite the potential advantages offered such as ready tailoring of dihedral angle, substitution patterns, and acidity (Figure 1B).^9^ Enantioenriched biphenols can be accessed by resolution or covalently attached auxiliaries, but this requires stoichiometric chiral material (Figure 1C, left).^9c,10^ In nature, 2,2′-biphenols are obtained through enzymatic oxidative phenolic coupling,^11^ and recent important developments in their enantioselective synthesis using biocatalysis have been reported.^12^ A variety of chemical methods have enabled the asymmetric oxidative coupling of naphthols,^13^ but the analogous coupling of phenols remains challenging (Figure 1C, center).^14^ Asymmetric Suzuki–Miyaura coupling should be the most modular and general disconnection to obtain enantioenriched 2,2′-biphenols, but the paucity of effective methods for enantioselective biphenyl couplings have precluded this (Figure 1C, right).

We have recently repurposed the sulfonated phosphine ligand **sSPhos**, originally reported by Buchwald.^15^ Instead of using the sulfonate group to impart water-solubility, we utilized it to control site-selectivity in cross-couplings of polychlorinated arenes.^16^ We deduced that the sulfonate group engages in attractive electrostatic interactions with deprotonated, Brønsted acidic substrates via its associated cation, directing site-selective oxidative addition. We became intrigued by the fact that the sulfonation of **SPhos** renders the product **sSPhos** chiral through axial desymmetrization, albeit in racemic form (Figure 1D, upper). We speculated that enantiopure **sSPhos** may constitute a unique chiral phosphine ligand for enantioselective Suzuki–Miyaura couplings. This ligand would possess the parent **SPhos** scaffold, providing the ability to form very hindered C–C bonds, in combination with close proximity of the sulfonate group to the metal center, providing a chiral environment. Sulfonate groups are proficient at engaging in attractive noncovalent interactions, as we have utilized for enantioselectivity control in other metal-catalyzed reactions.^17^ We envisage it should be well-placed to engage in hydrogen bonding interactions with phenolic coupling partners during any of the three key mechanistic steps of the catalytic cycle, all of which have been suggested to potentially influence product selectivity in the formation of hindered biaryls.^6e,6h,6p,6r^ Interestingly, weaker attractive noncovalent interactions have been implicated in several asymmetric Suzuki–Miyaura protocols on the basis of DFT calculations,^6a,6e,6h,6l,6p^ and the leading protocol from Tang and co-workers invoked a key catalyst–substrate hydrogen bond.^6s,18^

One can envisage three potential avenues for accessing enantiopure **sSPhos**: preparative chiral chromatography, diastereomer separation after covalent attachment of an auxiliary, and resolution via diastereomeric salt formation (Figure 1D, lower). In the first instance, enantiopure material was obtained by preparative chiral SFC and we initially examined the Suzuki–Miyaura coupling of aryl bromide **1a** and boronate ester **2a** to give the atropisomeric biphenol **3a** (Table 1). We were delighted to find that an initial evaluation using K_3_PO_4_ as base showed very encouraging results, with optimal enantioselectivity obtained in a biphasic toluene/water solvent system (entries 1–3). The yield could be improved by modifying the inorganic base, and a brief survey revealed that enantioselectivity was unaffected by the base: potassium, cesium, and sodium carbonate bases all gave similar enantioselectivity (entries 4–6). The optimal was found to be Na_3_PO_4_, affording **3a** in 73% yield and 92% ee (entry 7).

**Table 1:**
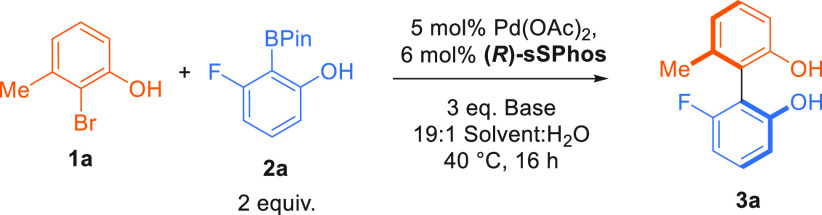
Optimization of Solvent and Base^a^

entry	base	solvent	% yield	% ee
1	K_3_PO_4_	THF	19	81
2	K_3_PO_4_	MeCN	5	74
3	K_3_PO_4_	toluene	18	95
4	K_2_CO_3_	toluene	40	90
5	Cs_2_CO_3_	toluene	16	90
6	Na_2_CO_3_	toluene	67	88
7	Na_3_PO_4_	toluene	73^b^	92^c^

a Yields determined by ^1^H NMR with internal standard. ee determined by SFC analysis of crude reaction mixture.

b Isolated yield.

c ee following isolation.

Using boronate ester **2a**, we evaluated the influence of substituents around the aryl bromide. A chlorine atom adjacent to the phenolic hydroxyl gave an excellent outcome (Scheme 1A, **3b**, 99% ee). Extra alkyl (**3c**) and fused alkyl (**3d**) substituents were well tolerated. Switching from alkyl to chloride as the *ortho* substituent on the aryl bromide also worked well (**3e**), and a methyl group (**3f**) and additional chlorine atoms (**3g**, **3h**) were accommodated on the ring at various positions. Any moderate yields were generally due to competitive debromination or incomplete conversion. Including a chloride atom adjacent to the phenol again resulted in a very high enantioselectivity (**3h**, 97% ee). This position could also accommodate a Boc-protected amine (**3i**); the NH of which does not interrupt putative hydrogen bonding interactions with the ligand. A methoxy group adjacent to the phenol gave **3j** in 98% ee. We next evaluated larger groups around the biaryl axis and found that a chain terminated in a nitro group (**3k**) was compatible, as were a silyl-protected alcohol (**3l**) and a Boc-protected amine (**3m**). To demonstrate the reaction on more complex partners, we brominated the antibacterial agent triclosan, which underwent highly selective coupling (**3n**). 4-Bromoestrone underwent smooth coupling giving **3o** in 19:1 dr. We successfully examined several aryl bromides in combination with a chloro-substituted phenol boronate ester following minor reoptimization (conditions B, **3p**–**3v**). *C*_2_-symmetric product **3r** was obtained in 97% ee. We were able to determine the absolute stereochemistry of this compound by X-ray crystallography (all others assigned by analogy) and have also evaluated the impact on ee of further transformation of the chlorine atom close to the axis in a derivative of **3e** (see Supporting Information). We explored two examples with trifluoromethoxy substitution on the boronate ester which worked well (**3u** and **3v**), although an *ortho* methyl-substituted boronate ester gave low yields (see Supporting Information). Synthesis of a biphenol that was not tetra-*ortho*-substituted revealed that the barrier to interconversion was too low for this compound to be configurationally stable at room temperature, and we have also included details of unsuccessful substrates (see Supporting Information for details in both cases). We were intrigued by the possibility of performing a double Suzuki–Miyaura coupling on dibromophenols to form triphenolic products that possess two independent atropisomeric axes (Scheme 1B). These reactions can form two diastereomers, one chiral and one achiral. We evaluated the coupling of two different sterically hindered dibromophenols and were pleased to find that in both cases excellent levels of enantioselectivity were obtained in the chiral diastereomers **5a** and **5c** (92 and 98% ee, respectively). The yield of **5a** was low due to very challenging purification required to obtain clean material.

**Scheme 1:**
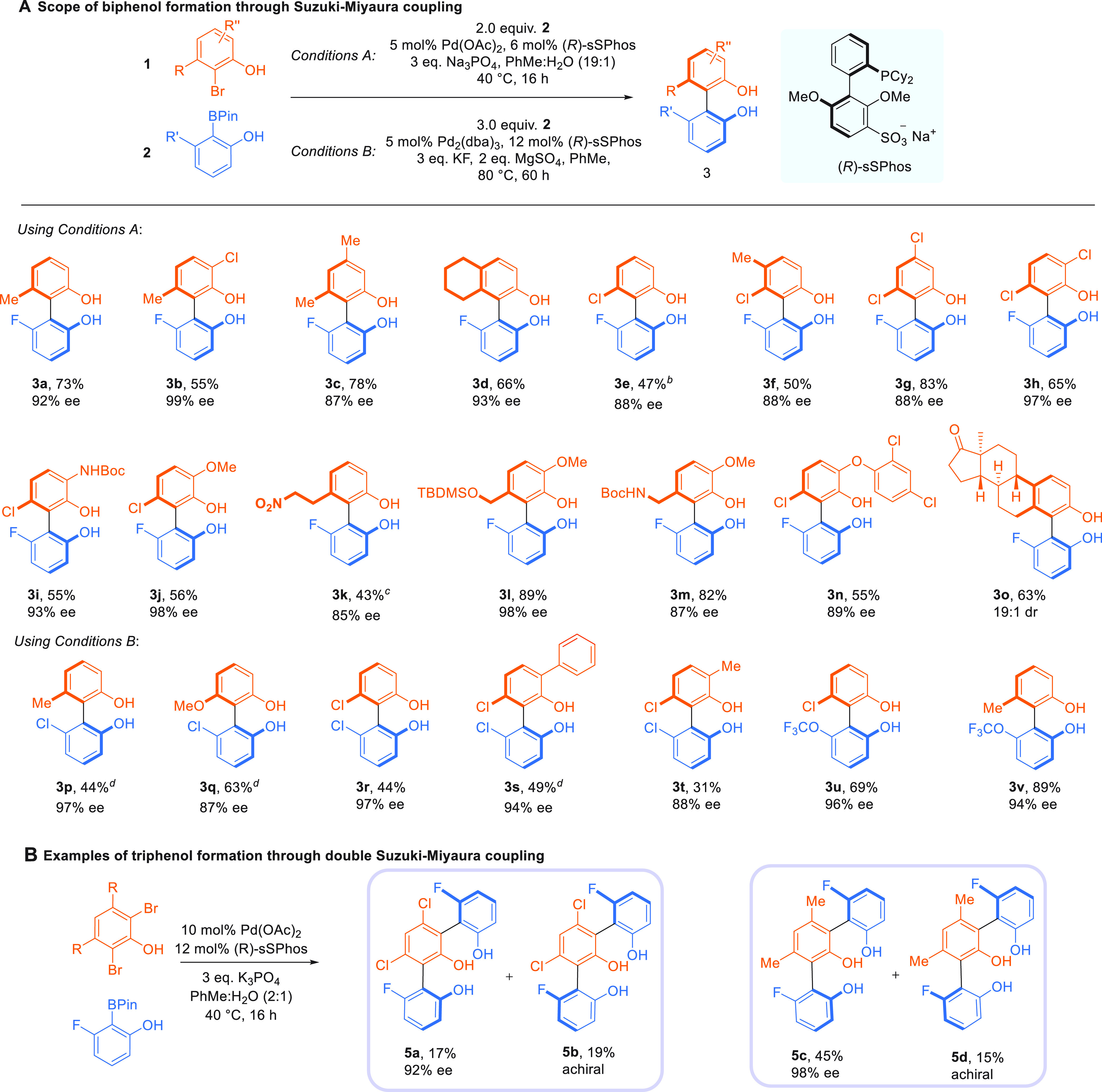
Scope of the Enantioselective Suzuki–Miyaura Coupling for Both Biphenols and Triphenols Yields are isolated. ee values determined by SFC. 2.6 equiv of **2** used. 5.0 equiv of **2** used with 10 mol % Pd(OAc)_2_ and 12 mol % (*R*)-**sSPhos** for 64 h. 2.0 equiv of **2** used with 2.5 mol % Pd_2_(dba)_3_ and 6 mol % (*R*)-**sSPhos** for 16 h.

We next probed the substrate features critical for high enantioselectivity, systematically comparing outcomes when the phenolic oxygen of each component or both are methylated (Scheme 2A). This revealed that phenolic hydroxyl groups on both boronic acid and aryl bromide are crucial to obtaining the highest enantioselectivities. With a hydroxyl on only one partner (**6a** or **6b**), appreciable selectivity could still be obtained but this was noticeably reduced when compared with **3a** (hydroxyls on both partners). With phenolic hydroxyls absent on both partners almost no enantioselectivity was observed (**6c**). A control experiment using a neutral, alkylated version of (*S*)-**sSPhos** gave product in good yield but only −8% ee, clearly demonstrating the crucial nature of the sulfonate group; the alkylation of which would greatly reduce its abilities as a hydrogen bond acceptor (Scheme 2B). We could replace one of the phenolic hydroxyls with an acetamide group, and although the yield was low, product **7** was obtained in highly encouraging 83% ee (Scheme 2C). This preliminary result demonstrates that our approach is effective for targeting atropisomeric 2-amino-2′-hydroxybiphenyl motifs, close relatives of NOBIN.^19^

**Scheme 2:**
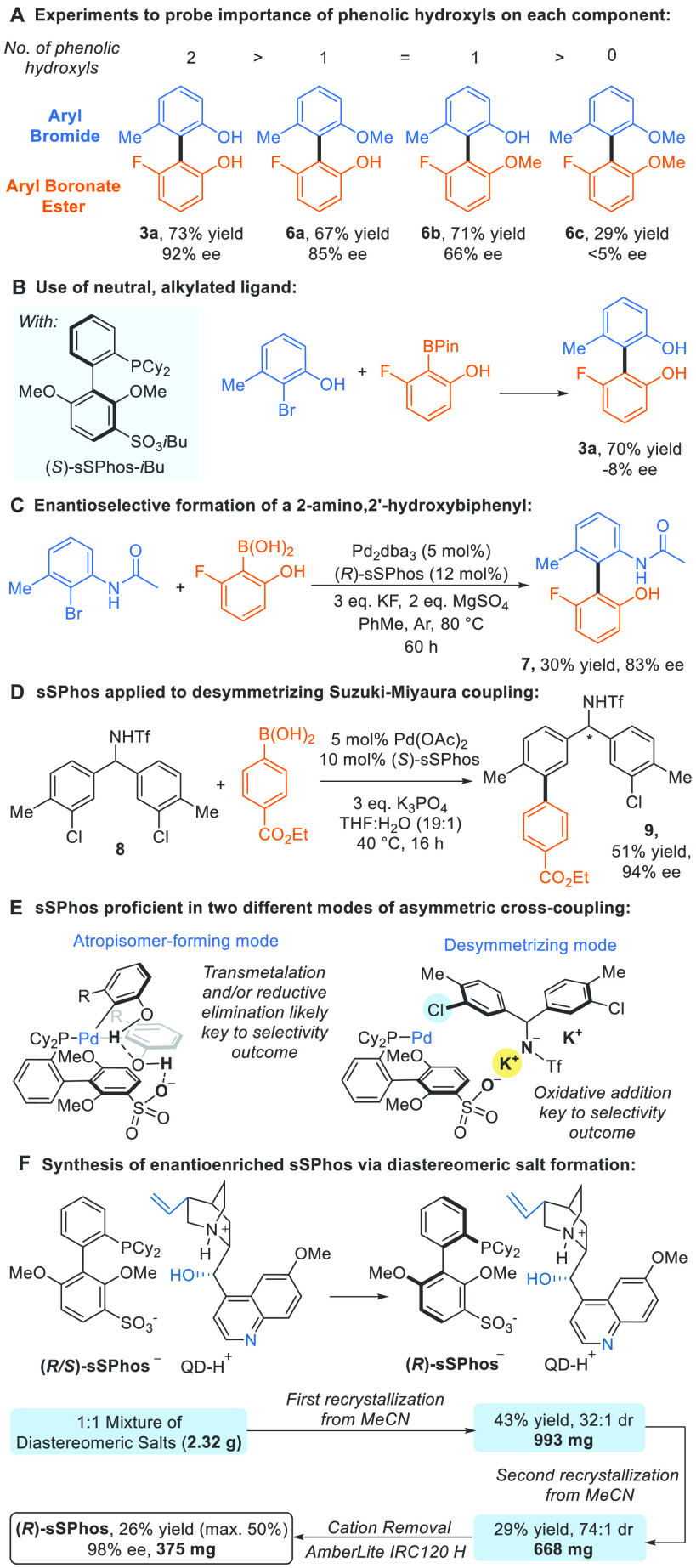
Control Experiments, Desymmetrizing Cross-Coupling, and Ligand Recrystallization

Having applied enantiopure **sSPhos** to the generation of axial chirality, we were keen to test its ability to introduce point chirality and evaluated it in the desymmetrization of *N*-triflated benzhydrylamine **8**, in which oxidative addition would now be enantiodetermining and occurs at a position remote from the new stereocenter (Scheme 2D). Such long-range stereoinduction is a challenge, and one in which catalysts that exploit attractive noncovalent interactions have demonstrated particular advantages.^17a,20^ In our previous work on site-selective cross-coupling of related Brønsted acidic substrates we proposed that ligand–substrate electrostatic interactions were key,^16^ and in a recent report Zhu and co-workers successfully accomplished a related desymmetrizing Suzuki–Miyaura coupling using a novel chiral phosphonate ligand inspired by that approach.^21^ Use of enantiopure **sSPhos** in the desymmetrizing Suzuki–Miyaura coupling of **8** resulted in high enantioselectivity in the product **9**. This demonstrates its proficiency in the formation of two fundamentally different chirality classes, axial chirality in the synthesis of atropisomers such as **3** and point chirality in the desymmetrization of substrates such as **8**. For atropisomer formation, all steps could potentially contribute to the selectivity outcome but it is the later stages, transmetalation and particularly reductive elimination, that have been most commonly suggested to dominate (Scheme 2E, left).^6e,6h,6p,6r^ In contrast, for point desymmetrization it is most likely initial oxidative addition which is selectivity-determining (Scheme 2E, right). For atroposelective coupling, our working hypothesis is that a hydrogen bonding interaction between one phenolic hydroxyl and the ligand sulfonate group is key and it is plausible that the second phenolic hydroxyl forms an additional hydrogen bond in an arrangement that results in the highest ee (Scheme 2E, left). Phenol deprotonation in reaction intermediates cannot be ruled out, but the lack of ee dependence on the nature of the cation (Table 1) together with experiments that suggest deprotonation of the phenolic starting material is not occurring (see Supporting Information) provides some evidence against this.

In parallel to the studies described above, we sought to develop synthetic access to enantiopure **sSPhos** without relying on preparative chromatography. The first strategy developed involved incorporating BINOL as a chiral auxiliary, allowing diastereomeric intermediates to be separated by flash chromatography (see Supporting Information). More practically, we have developed an approach based on the resolution of diastereomeric salts; the anionic nature of **sSPhos** presents the opportunity to pair it directly with a chiral cation. We discovered that the combination of protonated quinidine paired with racemic **sSPhos** anion led to highly diastereoselective crystallization upon cooling from acetonitrile (Scheme 2F). After one recrystallization the salt was determined to have a diastereomeric ratio (dr) of 32:1 and an X-ray crystal structure of the obtained material confirmed the **sSPhos** component to have *R* configuration. The dr increased to 74:1 after a second recrystallization and the sodium salt of **sSPhos** was obtained using AmberLite resin to give (*R*)-**sSPhos** in 26% total yield (theoretical maximum 50%) with 98% ee. This facile procedure, which uses commercially available and cheap starting materials, allows rapid access to highly enantioenriched **sSPhos** without specialist facilities.

In summary, we have developed an atroposelective Suzuki–Miyaura protocol that allows the rapid and modular synthesis of highly enantioenriched 2,2′-biphenols. A rare example of an asymmetric phenyl-phenyl cross-coupling, it has been enabled by the resolution of sulfonated **SPhos** (**sSPhos**), a ligand explored until this point only in racemic form. This work demonstrates the potential of this ligand to enable challenging asymmetric transformations and we have developed a practical recrystallization protocol which allows highly enantioenriched **sSPhos** to be obtained quickly and cheaply. We anticipate that with its versatile chiral structure, this ligand will find application in other branches of palladium chemistry and indeed more broadly within asymmetric transition metal catalysis.

## Supporting Information Available

Additional optimization, full experimental details, and characterization data for compounds (PDF)

## References

[ref1] Suzuki A. Cross-Coupling Reactions Of Organoboranes: An Easy Way To Construct C-C Bonds (Nobel Lecture). Angew. Chem., Int. Ed. 2011, 50, 6722–6737. 10.1002/anie.201101379. 21618370

[ref2] aClayden J.; Moran W. J.; Edwards P. J.; LaPlante S. R. The Challenge of Atropisomerism in Drug Discovery. Angew. Chem., Int. Ed. 2009, 48, 6398–6401. 10.1002/anie.200901719. 19637174

[ref3] aCammidge A. N.; Crépy K. V. L. The first asymmetric Suzuki cross-coupling reaction. Chem. Commun. 2000, 1723–1724. 10.1039/b004513f.

[ref4] Hayashi T.; Hayashizaki K.; Kiyoi T.; Ito Y. Asymmetric synthesis catalyzed by chiral ferrocenylphosphine-transition-metal complexes. 6. Practical asymmetric synthesis of 1,1′-binaphthyls via asymmetric cross-coupling with a chiral [(alkoxyalkyl)ferrocenyl]monophosphine/nickel catalyst. J. Am. Chem. Soc. 1988, 110, 8153–8156. 10.1021/ja00232a030.

[ref5] aCherney A. H.; Kadunce N. T.; Reisman S. E. Enantioselective and Enantiospecific Transition-Metal-Catalyzed Cross-Coupling Reactions of Organometallic Reagents To Construct C–C Bonds. Chem. Rev. 2015, 115, 9587–9652. 10.1021/acs.chemrev.5b00162. 26268813 PMC4566132

[ref6] aCammidge A. N.; Crépy K. V. L. Synthesis of chiral binaphthalenes using the asymmetric Suzuki reaction. Tetrahedron 2004, 60, 4377–4386. 10.1016/j.tet.2003.11.095.

[ref7] aBringmann G.; Price Mortimer A. J.; Keller P. A.; Gresser M. J.; Garner J.; Breuning M. Atroposelective Synthesis of Axially Chiral Biaryl Compounds. Angew. Chem., Int. Ed. 2005, 44, 5384–5427. 10.1002/anie.200462661. 16116589

[ref8] Brunel J. M. BINOL: A Versatile Chiral Reagent. Chem. Rev. 2005, 105, 857–898. 10.1021/cr040079g. 15755079

[ref9] aHua Z.; Vassar V. C.; Choi H.; Ojima I. New biphenol-based, fine-tunable monodentate phosphoramidite ligands for catalytic asymmetric transformations. Proc. Natl. Acad. Sci. U.S.A. 2004, 101, 5411–5416. 10.1073/pnas.0307101101. 15020764 PMC397395

[ref10] aShigeyoshi K.; Nobuyuki T.; Masatoshi M.; Hiroshi S. Optical Resolution and Absolute Configuration of Axially Dissymmetric 2,2′-Dihydroxy-6,6′-dimethylbiphenyl. Bull. Chem. Soc. Jpn. 1987, 60, 2307–2309. 10.1246/bcsj.60.2307.

[ref11] Hüttel W.; Müller M. Regio- and stereoselective intermolecular phenol coupling enzymes in secondary metabolite biosynthesis. Nat. Prod. Rep. 2021, 38, 1011–1043. 10.1039/D0NP00010H. 33196733

[ref12] aAldemir H.; Richarz R.; Gulder T. A. M. The Biocatalytic Repertoire of Natural Biaryl Formation. Angew. Chem., Int. Ed. 2014, 53, 8286–8293. 10.1002/anie.201401075. 25045123

[ref13] aMulrooney C. A.; Li X.; DiVirgilio E. S.; Kozlowski M. C. General Approach for the Synthesis of Chiral Perylenequinones via Catalytic Enantioselective Oxidative Biaryl Coupling. J. Am. Chem. Soc. 2003, 125, 6856–6857. 10.1021/ja027745k. 12783524

[ref14] aKang H.; Lee Y. E.; Reddy P. V. G.; Dey S.; Allen S. E.; Niederer K. A.; Sung P.; Hewitt K.; Torruellas C.; Herling M. R.; Kozlowski M. C. Asymmetric Oxidative Coupling of Phenols and Hydroxycarbazoles. Org. Lett. 2017, 19, 5505–5508. 10.1021/acs.orglett.7b02552. 29022352 PMC5654492

[ref15] aAnderson K. W.; Buchwald S. L. General Catalysts for the Suzuki–Miyaura and Sonogashira Coupling Reactions of Aryl Chlorides and for the Coupling of Challenging Substrate Combinations in Water. Angew. Chem., Int. Ed. 2005, 44, 6173–6177. 10.1002/anie.200502017. 16097019

[ref16] aGolding W. A.; Pearce-Higgins R.; Phipps R. J. Site-Selective Cross-Coupling of Remote Chlorides Enabled by Electrostatically Directed Palladium Catalysis. J. Am. Chem. Soc. 2018, 140, 13570–13574. 10.1021/jacs.8b08686. 30295472

[ref17] aGenov G. R.; Douthwaite J. L.; Lahdenperä A. S. K.; Gibson D. C.; Phipps R. J. Enantioselective remote C-H activation directed by a chiral cation. Science 2020, 367, 1246–1251. 10.1126/science.aba1120. 32165586

[ref18] Fanourakis A.; Docherty P. J.; Chuentragool P.; Phipps R. J. Recent Developments in Enantioselective Transition Metal Catalysis Featuring Attractive Noncovalent Interactions between Ligand and Substrate. ACS Catal. 2020, 10, 10672–10714. 10.1021/acscatal.0c02957. 32983588 PMC7507755

[ref19] Ding K.; Li X.; Ji B.; Guo H.; Kitamura M. Ten Years of Research on NOBIN Chemistry. Curr. Org. Synth. 2005, 2, 499–545. 10.2174/157017905774322631.

[ref20] aMetrano A. J.; Miller S. J. Peptide-Based Catalysts Reach the Outer Sphere through Remote Desymmetrization and Atroposelectivity. Acc. Chem. Res. 2019, 52, 199–215. 10.1021/acs.accounts.8b00473. 30525436 PMC6335614

[ref21] Lou Y.; Wei J.; Li M.; Zhu Y. Distal Ionic Substrate–Catalyst Interactions Enable Long-Range Stereocontrol: Access to Remote Quaternary Stereocenters through a Desymmetrizing Suzuki–Miyaura Reaction. J. Am. Chem. Soc. 2022, 144, 123–129. 10.1021/jacs.1c12345. 34979078 PMC9549467

