# Supplementary material for: An Enantioselective Suzuki–Miyaura Coupling To Form Axially Chiral Biphenols
Source: J Am Chem Soc. 2022 Aug 15;144(33):15026–32. doi: 10.1021/jacs.2c06529 (PMC9434994; doi:10.1021/jacs.2c06529)
Supplement: Supplementary file 1 — ja2c06529_si_001.pdf [file ja2c06529_si_001.pdf]

# Supporting Information

## An Enantioselective Suzuki-Miyaura Coupling to Form Axially Chiral Biphenols

Robert Pearce-Higgins,[1†] Larissa N. Hogenhout,[1†] Philip J. Docherty,[1†] David M. Whalley,[1] Padon Chuentragool,[1#] Najung Lee,[1] Nelson Y. S. Lam,[1] Thomas M. McGuire,[2] Damien Valette[3‡] and Robert J. Phipps[1*]

[1]Yusuf Hamied Department of Chemistry, University of Cambridge, Lensfield Road, Cambridge, CB2 1EW, United Kingdom.

[2]Oncology R&D, AstraZeneca; Cambridge CB4 0WG, United Kingdom.

[3]GlaxoSmithKline Medicines Research Centre; Stevenage, Hertfordshire SG1 2NY, United Kingdom.

†These authors contributed equally.

# Table of Contents


Supporting Information ........ S1

General Information ........ S3

Synthesis of Starting Materials ........ S4

Reaction Optimisation (Table 1, extended) ........ S18

Synthesis of Products (Scheme 1) ........ S20

Unsuccessful Substrates ........ S34

Experiments to probe whether a starting phenolic aryl bromide is deprotonated under reaction conditions ........ S35

Evaluation of a biphenol that is not tetra-ortho substituted ........ S36

Evaluation of an *ortho*-methyl substituted boronate ester ........ S37

Preliminary Investigation of Post-Functionalization of an Axially Positioned Chloride ........ S38

Control Reactions to Vary Phenolic Hydroxyls (Scheme 2A) ........ S41

Control Experiment Using Alkylated Ligand (Scheme 2B) ........ S43

Enantioselective formation of a 2-amino, 2'-hydroxybiphenyl ........ S44

sSPhos Applied to Desymmetrising Suzuki-Miyaura Coupling (Scheme 2D) ........ S46

Resolution of sSPhos by Preparative SFC ........ S48

Resolution of sSPhos using BINOL Auxiliary Approach ........ S49

Determination of ligand stereochemistry ........ S55

Resolution of sSPhos via recrystallisation of diastereomeric salts with quinidine ........ S56

Double recrystallisation procedure of *rac*-sSPhos/quinidinium salt (Scheme 2F) ........ S60

Starting Material NMR Spectra ........ S63

NMR Spectra of Products ........ S134

NMR Spectra for Ligand Synthesis ........ S237

Chiral SFC Traces ........ S272

References ........ S305

# General Information

**Reagents:** All reagents, unless otherwise stated, were used as supplied from commercial sources without further purification. $CH_2Cl_2$, THF was purified by distillation on site under inert atmosphere *via* the following processes: THF was pre-dried over sodium wire then distilled from calcium hydride and lithium aluminium hydride. $CH_2Cl_2$, *n*-hexane and toluene were distilled from calcium hydride.

**Reaction setup:** Reactions were carried out in 4 mL, 15x45mm crimp-top vials, which were purged with nitrogen or argon. In cases where the reactions were heated, the vials were heated in deep-welled heating blocks (IKA DB 5.2) or shallow heating blocks for reactions at 80 °C (IKA H135.101).

**NMR spectra:** $^{1}$H NMR spectra were recorded on a 700 MHz TXO Cryoprobe, 600 MHz Bruker Avance DRX-600 spectrometer, 500 MHz Bruker DCH Cryoprobe, 400 MHz Bruker QNP Cryoprobe or 400 MHz Bruker Avance NEO Prodigy $N_2$ Cryoprobe. Chemical shifts are reported in parts per million (ppm) and the spectra are calibrated to the resonance resulting from incomplete deuteration of the solvent ($CDCl_3$: 7.26 ppm; DMSO-$d_6$: 2.50 ppm, qn; MeOD-$d_4$: 3.31 ppm, qn). $^{13}$C NMR spectra were recorded on the same spectrometers with complete proton decoupling. Chemical shifts are reported in ppm with the solvent resonance as the internal standard ($^{13}CDCl_3$: 77.16 ppm, t; DMSO-$d_6$: 39.52 ppm, sept; MeOD-$d_4$: 49.00 ppm, sept). Data are reported as follows: chemical shift δ/ppm, multiplicity (s = singlet, d = doublet, t = triplet, q = quartet, qn = quintet, sept = septet, br = broad, m = multiplet or combinations thereof; $^{13}$C, $^{19}$F and $^{31}$P signals are singlets unless otherwise stated), coupling constants *J* in Hz, integration ($^{1}$H only). $^{1}$H-COSY, DEPT-135, HMQC and HMBC were used where appropriate to facilitate structural determination. The carbon atom attached to boron was generally not observed by $^{13}$C spectroscopy due to quadrupolar relaxation. $^{19}$F and $^{31}$P NMR spectra were recorded on a 400 MHz Bruker Avance III HD and 400 MHz Bruker Avance NEO Prodigy $N_2$ Cryoprobe Spectrometer with complete proton decoupling.

**High Resolution Mass Spectrometry (HRMS):** Some were recorded on a Waters Micromass LCT Premier spectrometer using an electrospray ionization (ESI) or on a Waters Xevo G2-S bench top QTOF using an electrospray ionization (ESI) or atmospheric solids analysis probe (ASAP). The calculated values are based on the most abundant isotope.

**Chromatography**: Analytical thin layer chromatography was performed using precoated Merck glass backed silica gel plates (Silicagel 60 F254). Visualisation was by ultraviolet fluorescence (λ = 254 or 365 nm) and/or staining with potassium permanganate ($KMnO_4$). Flash column chromatography was performed using silica gel 60 (0.040-0.063 µm) from Fluorochem (for borylated products) or Material Harvest Ltd (in rest of cases).

**Optical rotations**: Measured in $CHCl_3$ or MeOH on a Perkin Elmer 343 Polarimeter using a sodium lamp (λ 589 nm, D-line). Values are reported at a given temperature (°C) in degrees.$cm^2.g^{-1}$ with concentration in $cg.mL^{-1}$.

**Chiral SFC analysis**: Performed on a Waters ACQUITY UPC2 system with YMC CHIRAL ART SB, SC or SJ, or DAICEL CHIRAL PAK IE, IH or IG columns (4.6x250 mm, 3 µm) in a mixed solvent system of supercritical $CO_2$ and MeOH. A system backpressure of 138 bar was used in all cases.

**X-ray crystallography**: Performed on a Nonius Kappa CCD diffractometer or a Bruker D8- QUEST PHOTON-100 diffractometer using CuKa radiation (lambda = 1.5418 Å) at the Cambridge University

Chemistry X-Ray Laboratory. Data collection and analysis was performed by Dr. Andrew Bond (University of Cambridge).

## Synthesis of Starting Materials

**General procedure C: *ortho*-selective monobromination of phenols**

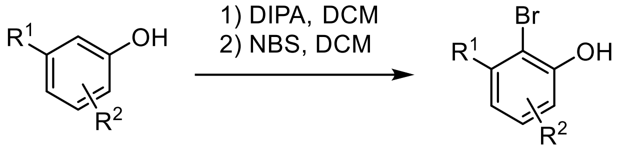


Synthesised according to the *ortho*-selective bromination procedure of Fujisaki et al. (*1*, *2*) To a solution of phenol (1.0 equiv.) in the minimum amount of DCM was added diisopropylamine (0.37 mL per mmol of substate). The mixture was further diluted in DCM (2.0 mL per mmol of substrate) and cooled to 0 °C, at which point NBS (0.80 equiv.) was added portionwise over 30 min. The reaction was allowed to warm slowly to rt and left to stir for 16 h. The reaction was quenched with saturated aqueous $Na_2SO_3$ solution and extracted with further DCM. The combined organic layers were dried over $MgSO_4$ and evaporated under reduced pressure to afford the crude material which was purified *via* column chromatography (0-5% EtOAc:petrol) to afford the desired product.

**General procedure D: *ortho*-selective dibromination of phenols**

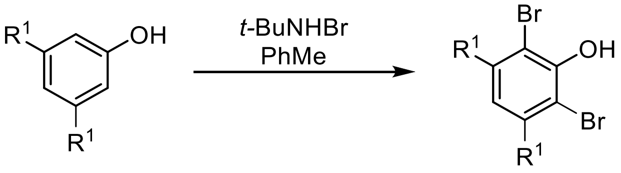


Synthesised according to the *ortho*-selective dibromination procedure of Shi et al. (*3*) To a solution of *tert*-butylamine (4.0 equiv.) in anhydrous toluene (1 mL per mmol of *tert*-butylamine) was added $Br_2$ (2.0 equiv.) dropwise at -30 °C. The reaction was stirred at -30 °C for 5 min, at which point a solution of 3,5-disubstituted phenol (1.0 equiv.) in toluene (4 mL per mmol of substrate) was added slowly at -30 °C. The resulting mixture was allowed to warm slowly up to rt and 10% aqueous $NaHCO_3$ (100 mL) was added and the crude material extracted with EtOAc (100 mL x 3). The combined organic layers were washed sequentially with saturated aqueous $Na_2S_2O_3$ solution (50 mL) and brine (30 mL), dried over $MgSO_4$, and the solvent removed under reduced pressure. The crude material was purified *via* column chromatography (0-2% EtOAc:petrol) to afford the desired product.

2-bromo-3-chloro-6-methylphenol

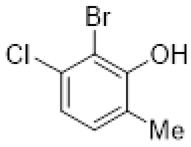

Synthesised according to general procedure **C** on a 1.53 mmol scale with respect to 5-chloro-2-methylphenol to afford the title compound as a white solid (142 mg, 0.642 mmol, 52%).

**$^1$H NMR** (500 MHz, $CDCl_3$) δ 7.03 (d, J = 8.1 Hz, 1H), 6.97 (d, J = 8.1 Hz, 1H), 5.76 (s, 1H), 2.29 (s, 3H). **$^{13}$C NMR** (126 MHz, $CDCl_3$) δ 151.7, 131.4, 130.1, 123.9, 121.4, 110.8, 16.4. **HRMS** $[M-H]^-$ calculated for $[C_7H_5BrClO]^-$ 218.9218, found 218.9228 Δ= 4.6 ppm.

2-bromo-3,5-dimethylphenol

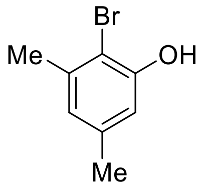


Synthesised according to the *ortho*-selective bromination procedure of Takeda Pharmaceutical Company. (*4*) To a solution of 3,5-dimethylphenol (1.00 g, 8.20 mmol) in carbon disulfide (20 mL) was added *N*-bromosuccinimide (1.46 g, 8.20 mmol) portionwise at 0 °C. The reaction mixture was allowed to warm to rt and stirred for 1 h. The solvent was removed under reduced pressure and the crude material was filtered and washed with a 10:1 EtOAc/hexane solvent system. The filtrate was concentrated and the crude material was purified *via* column chromatography (0-20% $CHCl_3$:petrol) to afford the title product as a colourless oil (890 mg, 4.43 mmol, 54%).

**$^1$H NMR** (400 MHz, $CDCl_3$) δ 6.72 (s, 1H), 6.66 (s, 1H), 5.52 (s, 1H), 2.38 (s, 3H), 2.27 (s, 3H). **$^{13}$C NMR** (101 MHz, $CDCl_3$) δ 152.0, 138.3, 138.0, 123.5, 113.8, 109.8, 22.9, 20.9.

**HRMS** $[M-H]^-$ calculated for $[C_8H_8BrO]^-$ 198.9764, found 198.9767 Δ= 1.5 ppm.

1-bromo-5,6,7,8-tetrahydronaphthalen-2-ol

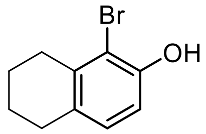


To a solution of 5,6,7,8-tetrahydro-2-naphol (493 mg, 3.33 mmol) in EtOH (50 mL) was added freshly prepared *N*-bromoacetamide (457 mg, 3.30 mmol, prepared according to ref. (*5*)) portionwise at rt. The reaction mixture was left to stir for 16 h, at which point the solvent was removed under reduced pressure. The crude material was purified *via* column chromatography (0-1% EtOAc:petrol) to afford the desired product as a white solid (400 mg, 1.76 mmol, 53%).

**$^1$H NMR** (500 MHz, $CDCl_3$) δ 6.97 (d, *J* = 8.3 Hz, 1H), 6.84 (d, *J* = 8.3 Hz, 1H), 5.49 (s, 1H), 2.77 – 2.70 (m, 4H), 1.89 – 1.79 (m, 2H), 1.79 – 1.71 (m, 2H). **$^{13}$C NMR** (126 MHz, $CDCl_3$) δ 150.0, 136.6, 131.0, 129.1, 113.5, 112.8, 30.5, 29.3, 23.2, 22.7.

Data in accordance with literature. (*6*)

1-chloro-3-(methoxymethoxy)benzene

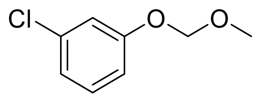

A stirred solution of 3-chlorophenol (2.1 mL, 20 mmol) in THF (30 mL) was cooled to 0 °C and sodium hydride (60% w/w in mineral oil, 0.95 g, 24 mmol, 1.2 eq.) was added portionwise. The mixture was stirred at rt for 30 min, then cooled back down to 0 °C and MOMCl (2.1 M in PhMe, prepared according to literature procedure (*7*), 10.3 mL, 21.5 mmol, 1.1 eq.) was added dropwise. The reaction was allowed to warm to rt whilst stirring over 16 h, after which the reaction was stopped by the addition of $NH_4Cl$ solution (sat., 15 mL). The reaction mixture was partially concentrated under reduced pressure, then EtOAc (50 mL) and water (50 mL) were added and the organic layer separated. The organic layer was washed with NaOH solution (2.5 M, 25 mL) then brine (sat., 25 mL), dried ($MgSO_4$) and solvent removed under reduced pressure. The residue was purified *via* column chromatography (30% EtOAc:petrol) to yield the product as a transparent oil (0.937 g, 5.3 mmol, 27%).

**$^1$H NMR** (400 MHz, $CDCl_3$) δ 7.30 – 7.16 (m, 1H), 7.09 (s, 1H), 7.02 (d, *J* = 7.9 Hz, 1H), 6.96 (d, *J* = 8.4 Hz, 1H), 5.19 (s, 2H), 3.51 (s, 3H). **$^{13}$C NMR** (101 MHz, $CDCl_3$) δ 158.0, 134.8, 130.2, 122.0, 116.8, 114.6, 94.5, 56.1.

Data in accordance with literature. (*8*)

<u>2-bromo-1-chloro-3-(methoxymethoxy)benzene</u>

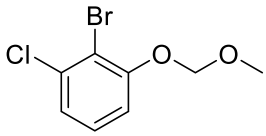

1-chloro-3-(methoxymethoxy)benzene (1.7 g, 10 mmol) was diluted in THF (20 mL), and cooled to -78 °C. *n*-BuLi (1.6 M in hexane, 6.9 mL, 11 mmol, 1.1 eq.) was added dropwise to the stirred solution, which turned pink initially. The solution was stirred for 1 h at -78 °C, after which the solution had turned a brown colour. NMR analysis of an aliquot quenched with $D_2O$ showed no starting material remaining. $Br_2$ (0.77 mL, 15 mmol, 1.5 eq.) was added dropwise to the solution, forming an orange solution, and was stirred at -78 °C for 1 h, then warmed to rt for 2 h. The reaction was stopped by addition of $NH_4Cl$ solution (sat., 10 mL), then $Et_2O$ (40 mL) and $Na_2SO_3$ solution (sat., 10 mL) added and the organic layer separated. The organic layer was washed with brine (sat., 20 mL), dried ($MgSO_4$), and solvent removed under reduced pressure to yield the crude product. The crude was purified by flash column chromatography (0-10% EtOAc:Petrol) to yield the title compound as a pale yellow oil (1.36 g, 5.4 mmol, 54%).

**$^1$H NMR** (500 MHz, $CDCl_3$) δ 7.21 (t, *J* = 8.1 Hz, 1H), 7.16 (dd, *J* = 8.1, 1.6 Hz, 1H), 7.07 (dd, *J* = 8.1, 1.6 Hz, 1H), 5.28 (s, 2H), 3.54 (s, 3H). **$^{13}$C NMR** (126 MHz, $CDCl_3$) δ 155.3, 135.8, 128.3, 123.5, 113.9, 113.8, 95.2, 56.5. **HRMS** $[M]^+$ calcd for $C_8H_8BrClO_2^+$; 249.9391, found 249.9385, Δ = 2.4 ppm.

<u>2-bromo-3-chlorophenol</u>

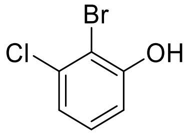

2-bromo-1-chloro-3-(methoxymethoxy)benzene (1.26 g, 5 mmol) was dissolved in THF (10 mL), and cooled to 0 °C. To the stirred solution conc. HCl (10 mL) was added and the reaction was warmed to rt over 16 h. EtOAc (20 mL) and ice-cold water (20 mL) was added, and the organic layer separated. The organic layer was washed with $NaHCO_3$ solution (sat., 20 mL), then brine (sat., 20 mL), dried ($MgSO_4$), then solvent removed under reduced pressure to yield crude product. The product was purified by flash column chromatography (0-15% EtOAc:Petrol) then crystallised from resulting fractions by removing most solvent under reduced pressure followed by slow evaporation, yielding the title compound as large transparent crystals (538 mg, 2.59 mmol, 52%).

**$^1$H NMR** (400 MHz, $CDCl_3$) δ 7.18 (t, *J* = 8.1 Hz, 1H), 7.06 (dd, *J* = 8.0, 1.5 Hz, 1H), 6.95 (dd, *J* = 8.2, 1.5 Hz, 1H), 5.74 (s, 1H). **$^{13}$C NMR** (126 MHz, $CDCl_3$) δ 153.8, 134.5, 129.0, 122.2, 114.1, 111.4.

Data in accordance with literature. (*9*)

<u>2-bromo-3-chloro-1-(methoxymethoxy)-4-methylbenzene</u>

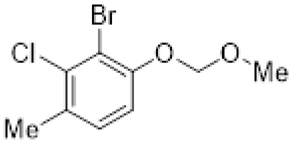


To a solution of 3-chloro-4-methylphenol (1.00 g, 7.01 mmol) in THF (10 mL) was added NaH (60% w/w in mineral oil, 365 mg, 9.12 mmol) portionwise at 0 °C. The reaction was allowed to warm to rt to stir for 30 min, before being re-cooled to 0 °C at which point MOM-Cl (2.1 M solution in toluene, 5.00 mL, 10.5 mmol) was added slowly. After complete addition, the reaction was allowed to warm to rt again and left to stir for 16 h. The reaction was quenched *via* the slow addition of saturated aqueous $NH_4Cl$ solution (50 mL) and extracted with EtOAc (3 x 50 mL). The combined organic extracts were dried over $MgSO_4$ and evaporated under reduced pressure to afford the crude MOM-protected material which was used without further purification. The crude material was dissolved in THF (30 mL) and *n*-BuLi (1.6 M in hexanes, 4.69 mL, 7.50 mmol) was added dropwise at -78 °C, at which point the reaction mixture turned yellow in colour. The reaction mixture was left to stir for 1 h at -78 °C before $Br_2$ (0.50 mL, 10 mmol) was added dropwise at -78 °C. The reaction mixture was left to stir for 1 h at -78 °C and then was allowed to warm to rt to stir for 2 h. Upon completion, the reaction was quenched with saturated aqueous $NH_4Cl$ solution (25 mL) and extracted with $Et_2O$ (3 x 25 mL). The combined organic extracts were dried over $MgSO_4$ and evaporated under reduced pressure. The crude material was purified *via* column chromatography (0-2% EtOAc:petrol) to afford the desired product as a brown oil containing 10% starting material which was removed in the subsequent synthetic step (642 mg, 0.607 mmol, 34% over two steps).

**$^1$H NMR** (500 MHz, $CDCl_3$) δ 7.14 (d, *J* = 9.1 Hz, 1H), 7.00 (d, *J* = 8.5 Hz, 1H), 5.25 (s, 2H), 3.54 (s, 3H), 2.40 (s, 3H). **$^{13}$C NMR** (126 MHz, $CDCl_3$) δ 153.2, 135.4, 131.3, 129.3, 114.3, 113.9, 95.4, 56.4, 20.9. **HRMS** [M-MOM]$^-$ calculated for $[C_7H_5BrClO]^-$ 218.9218, found 218.9220, Δ = 0.9 ppm.

<u>2-bromo-3-chloro-4-methylphenol</u>

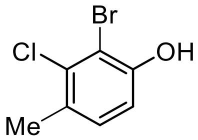

To a solution of 2-bromo-3-chloro-1-(methoxymethoxy)-4-methylbenzene (642 mg, 2.42 mmol) in THF (10 mL) was added concentrated HCl (36-38% w/w aqueous solution, 5.0 mL) slowly at rt. The reaction mixture was stirred at rt for 16 h, at which point the reaction mixture was extracted with EtOAc (3 x 5 mL). The combined organic extracted were dried over $MgSO_4$ and evaporated under reduced pressure to afford a brown oil. The crude material was purified *via* column chromatography (0-10% EtOAc:petrol) to afford the desired product as a brown solid (267 mg, 1.21 mmol, 50%).

**$^1$H NMR** (400 MHz, $CDCl_3$) δ 7.12 (d, *J* = 8.4 Hz, 1H), 6.89 (d, *J* = 8.4 Hz, 1H), 5.53 (s, 1H), 2.38 (s, 3H). **$^{13}$C NMR** (101 MHz, $CDCl_3$) δ 151.6, 133.9, 130.0, 129.6, 113.7, 111.5, 20.7. **HRMS** $[M-H]^-$ calculated for $[C_7H_5BrClO]^-$ 218.9218, found 218.9219, Δ = 0.5 ppm.

2-bromo-3,5-dichlorophenol

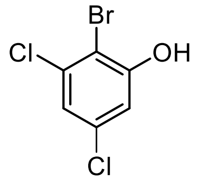


Synthesised according to general procedure **A** on a 6.1 mmol scale with respect to 3,5-dichlorophenol to afford the title compound as a white solid (75.5 mg, 0.312 mmol, 7%).

**$^1$H NMR** (400 MHz, $CDCl_3$) δ 7.09 (d, *J* = 1.8 Hz, 1H), 6.98 (d, *J* = 2.3 Hz, 1H), 5.81 (s, 1H). **$^{13}$C NMR** (101 MHz, $CDCl_3$) δ 154.0, 135.0, 134.5, 122.2, 114.7, 109.7. **HRMS** $[M-H]^-$ calculated for $[C_6H_2BrCl_2O]^-$ 238.8672, found 238.8673, Δ= 0.4 ppm.

2-bromo-3,6-dichlorophenol

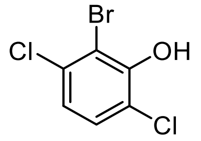


Synthesised according to general procedure **C** on a 6.1 mmol scale with respect to 2,5-dichlorophenol to afford the title compound as a white solid (502 mg, 2.07 mmol, 42%).

**$^1$H NMR** (400 MHz, $CDCl_3$) δ 7.27 (d, *J* = 8.6 Hz, 1H), 7.04 (d, *J* = 8.7 Hz, 1H), 6.07 (s, 1H). **$^{13}$C NMR** (101 MHz, $CDCl_3$) δ 150.0, 133.9, 128.6, 122.1, 118.9, 111.5. **HRMS** $[M-H]^-$ calculated for $[C_6H_2BrCl_2O]^-$ 238.8672, found 238.8673, Δ= 0.4 ppm.

tert-butyl (4-chloro-2-hydroxyphenyl)carbamate

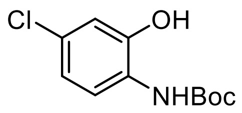


To a solution of 2-amino-5-chlorophenol (1.00 g, 7.00 mmol) in THF (50 mL) was added Boc anhydride (3.04 g, 14.0 mmol). The reaction was stirred at rt for 16 h, at which point complete consumption of starting material was observed by TLC. The solvent was removed under reduced pressure to afford a brown solid which was purified *via* column chromatography (0-10% EtOAc:petrol) to afford the desired product as an off-white solid (750 mg, 3.08 mmol, 44%).

**$^1$H NMR** (500 MHz, $CDCl_3$) δ 8.48 (s, 1H), 7.11 – 7.03 (m, 1H), 7.00 – 6.95 (m, 1H), 6.85 (dd, *J* = 8.5, 2.3 Hz, 1H), 6.72 (s, 1H), 1.55 (s, 9H). **$^{13}$C NMR** (126 MHz, $CDCl_3$) δ 155.0, 148.1, 130.3, 124.5, 122.0, 120.7, 118.9, 82.5, 28.4.

Data in accordance with literature. (*10*)

tert-butyl (3-bromo-4-chloro-2-hydroxyphenyl)carbamate

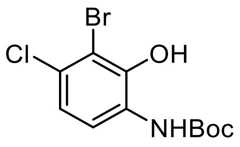


Synthesised according to general procedure **A** on a 1.53 mmol scale with respect to tert-butyl (4-chloro-2-hydroxyphenyl)carbamate to afford the title compound as a brown solid (248 mg, 0.769 mmol, 62%).

**$^1$H NMR** (400 MHz, $CDCl_3$) δ 8.45 (s, 1H), 7.00 (d, *J* = 7.6 Hz, 1H), 6.85 (d, *J* = 8.5 Hz, 1H), 6.63 (s, 1H), 1.55 (s, 9H). **$^{13}$C NMR** (101 MHz, $CDCl_3$) δ 155.0, 148.4, 130.5, 124.3, 122.1, 120.7, 119.2, 82.6, 28.2. **HRMS** $[M-H]^-$ calculated for $[C_{11}H_{12}BrClNO_3]^-$ 319.9695, found 319.9692, Δ= 0.9 ppm.

2-bromo-3-chloro-6-methoxyphenol

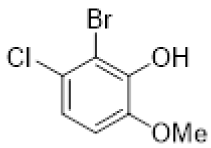


5-Chloro-2-methoxyphenylboronic acid (2.00 g, 10.7 mmol) and $NaHCO_3$ (4.50 g, 53.5 mmol) were dissolved in a biphasic solvent mixture of MeOH (10 mL) and THF (10 mL), at which point $H_2O_2$ (30% w/w in $H_2O$, 12.8 mL, 107 mmol) was added slowly. The reaction mixture was allowed to stir at for 1 h at rt, at which point the volatiles were removed under reduced pressure. The crude material was suspended in $H_2O$ (50 mL) and extracted with DCM (3 x 50 mL). The combined organic extracts were then washed with 3 M aqueous HCl solution (3 x 50 mL), dried over $MgSO_4$, and evaporated under reduced pressure to afford the crude material as a brown oil. The crude material was then brominated according to general procedure **C** on a 4.2 mmol scale with respect to the 5-chloro-2-methoxyphenol to afford the title compound as an orange oil (140 mg, 0.589 mmol, 10% yield over two steps).

**$^1$H NMR** (700 MHz, $CDCl_3$) δ 7.02 (d, *J* = 8.7 Hz, 1H), 6.79 (d, *J* = 8.7 Hz, 1H), 6.09 (s, 1H), 3.93 (s, 3H). **$^{13}$C NMR** (176 MHz, $CDCl_3$) δ 145.6, 144.6, 126.8, 120.4, 110.1, 109.4, 56.5. **HRMS** $[M-H]^-$ calculated for $[C_7H_5BrClO_2]^-$ 234.9167, found 234.9172, Δ= 2.1 ppm.

2-bromo-3-(2-nitroethyl)phenol

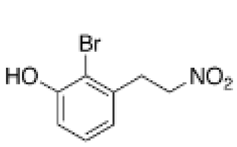


2-bromo-3-hydroxybenzylaldehyde (402 mg, 2.0 mmol, 1 eq.) was dissolved in acetic acid:nitromethane (12 mL, 2:1) with ammonium chloride (370 mg, 4.8 mmol, 1.4 eq). The reaction

mixture was heated to 100°C and stirred for 16 h. The reaction was cooled to rt, diluted with water and extracted three times with ethyl acetate, dried with $MgSO_4$, filtered and the solvent removed under vacuum. The crude mixture was then re-dissolved in toluene and the solvent removed under vacuum three times to remove any excess acetic acid. The crude nitroalkene product was then carried forward directly. The crude nitroalkene (c.a. 284 mg) was dissolved in an IPA:$CHCl_3$ (12 mL, 1:2.3) mixture, $SiO_2$ (1.25g, 21 mmol 18 eq.) was added, followed by a portionwise addition of $NaBH_4$ (132 mg, 3.48, 3 eq.) over 30 min at rt, then stirred for 16 h under nitrogen. The reaction mixture was then purified by flash column chromatography directly (20:80 to 30:70 to 40:60 ethyl acetate:petroleum ether) to give the product as a colourless solid, 192 mg, 0.78 mmol, 39% yield over two steps.

**$^1$H NMR** (500 MHz, $CDCl_3$) δ 7.18 (t, *J* = 7.8 Hz, 1H), 6.97 (dd, *J* = 8.1, 1.5 Hz, 1H), 6.83 (dd, *J* = 7.5, 1.6 Hz, 1H), 5.62 (s, 1H), 4.63 (t, *J* = 7.4 Hz, 2H), 3.46 (t, *J* = 7.4 Hz, 2H). **$^{13}$C NMR** (126 MHz, $CDCl_3$) δ 153.0, 135.8, 129.1, 122.8, 115.6, 112.5, 74.4, 34.1. **HRMS** $[M-H]^-$calcd for $[C_8H_7BrNO_3]^-$; 243.9615, found 243.9609, Δ= -2.5 ppm.

<u>2-bromo-3-(((*tert*-butyldimethylsilyl)oxy)methyl)-6-methoxyphenol</u>

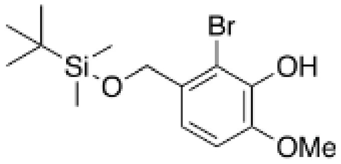


2-bromo-3-hydroxy-4-methoxybenzaldehyde (121 mg, 0.44 mmol) was dissolved in MeOH (2 mL) and sodium borohydride (46 mg, 1.32 mmol) was added portionwise at rt. The reaction mixture was stirred for 16 h under nitrogen then diluted with $H_2O$ and extracted three times with ethyl acetate. The organic extracts were dried with $MgSO_4$, filtered and the solvent removed under vacuum. The benzylic alcohol (127 mg, 0.54 mmol) intermediate was dissolved in anhydrous dichloromethane (5 mL), followed by sequential addition of imidazole (74 mg, 1.08 mmol) and TBSCl (86 mg, 0.57 mmol) at rt. The reaction mixture was stirred for 16 h, quenched with a saturated solution of ammonium chloride and extracted three times with dichloromethane. The organic extracts were dried with $MgSO_4$, filtered and the solvent removed under reduced pressure. The crude product was purified with flash column chromatography (10% to 20% to 30% ethyl acetate:petroleum ether) to give the product as a yellow amorphous solid (92 mg, 49%).

**$^1$H NMR** (400 MHz, $CDCl_3$) δ 7.05 (dt, *J* = 8.4, 1.0 Hz, 1H), 6.84 (d, *J* = 8.4 Hz, 1H), 5.94 (s, 1H), 4.70 (d, *J* = 1.0 Hz, 2H), 3.90 (s, 3H), 0.96 (s, 9H), 0.12 (s, 6H). **$^{13}$C NMR** (101 MHz, $CDCl_3$) δ 146.1, 142.8, 133.4, 118.3, 109.6, 107.6, 64.6, 56.5, 26.1, 18.6, -5.2. **HRMS** $[M-H]^-$ calcd for $[C_{14}H_{22}BrO_3Si]^-$; 345.0527, found 345.0524, Δ= -0.9 ppm.

<u>2-bromo-3-hydroxy-4-methoxybenzaldehyde</u>

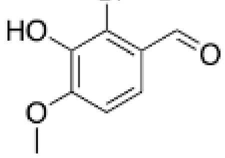

Isovanillin (1.0 mmol, 152 mg, 1.0 eq) and *N*-bromosuccinimide (1.15 mmol, 205 mg, 1.15 eq.) was dissolved in $CHCl_3$ (2.5 mL) and stirred at 70°C in a sealed microwave vial for 3 h. The reaction mixture was then cooled to rt, diluted with water and extracted five times with diethyl ether. The extracts were dried with $MgSO_4$, filtered and the solvent removed under vacuum to give the title compound as a light yellow solid (129 mg, 0.56 mmol, 56%).

**$^1$H NMR (**500 MHz, $CDCl_3$) δ 10.26 (d, *J* = 0.7 Hz, 1H), 7.58 (d, *J* = 8.6 Hz, 1H), 6.92 (dd, *J* = 8.6, 0.7 Hz, 1H), 6.08 (s, 1H), 4.00 (s, 3H). **$^{13}$C NMR** (126 MHz, $CDCl_3$) δ 191.1, 151.8, 143.4, 127.4, 122.9, 113.0, 109.4, 56.7. **HRMS** $[M-H]^-$ calcd for $[C_8H_6BrO_3]^-$; 228.9506, found 228.9505, Δ= -0.4 ppm.

*tert*-butyl (2-bromo-3-hydroxy-4-methoxybenzyl)carbamate

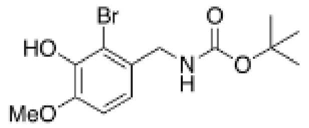


A microwave vial was charged with 2-bromo-3-hydroxy-4-methoxybenzaldehyde (231 mg, 1.0 mmol) and *tert*-butylcarbamate (351 mg, 3.0 mmol) and stirrer bar then sealed with a septum cap. The vial was then subjected to three vacuum/$N_2$ cycles.

Dichloromethane (1.1 mL) and acetonitrile (2.3 mL) were sparged with $N_2$ for 20 minutes and then added to the reaction mixture. Triethylsilane (0.48 mL, 3.0 mmol) and trifluoroacetic acid (0.15 mL, 2.0 mmol) were added sequentially *via* microsyringe under nitrogen atmosphere. The suspension was stirred overnight to give a yellow solution. The reaction was quenched with water and extracted three times with dichloromethane, dried with $MgSO_4$,filtered and the solvent removed under reduced pressure. The crude product was purified with flash column chromatography (20% to 30% to 40% ethyl acetate:petroleum ether), followed by a second column (0% to 3% MeOH:dichloromethane) to give the product as a colourless solid (112 mg, 34% yield).

**$^1$H NMR** (400 MHz, $CDCl_3$) δ 6.91 (d, *J* = 8.3 Hz, 1H), 6.77 (d, *J* = 8.3 Hz, 1H), 6.03 (s, 1H), 5.00 (s, 1H), 4.33 (d, *J* = 6.2 Hz, 2H), 3.88 (s, 3H), 1.44 (s, 9H). **$^{13}$C NMR** (101 MHz, $CDCl_3$) δ 155.8, 146.5, 143.4, 131.1, 120.6, 109.9, 109.6, 79.7, 56.5, 44.8, 28.5. **HRMS** $[M-H]^-$ calculated for $[C_{13}H_{17}BrNO_4]^-$; 330.0346, found 330.0342, Δ= -1.2 ppm.

2-bromo-3-chloro-6-(2,4-dichlorophenoxy)phenol

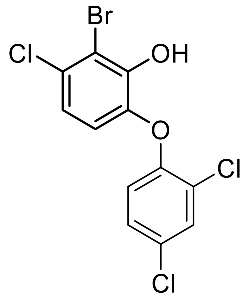


Synthesised according to general procedure **C** on a 3.1 mmol scale with respect to triclosan to afford the title compound as a colourless oil that solidified upon standing (259 mg, 0.703 mmol, 23%).

**$^1$H NMR** (400 MHz, $CDCl_3$) δ 7.50 (d, *J* = 2.5 Hz, 1H), 7.24 (dd, *J* = 8.8, 2.5 Hz, 1H), 7.01 (d, *J* = 8.8 Hz, 1H), 6.94 (d, *J* = 8.8 Hz, 1H), 6.72 (d, *J* = 8.8 Hz, 1H), 6.11 (s, 1H). **$^{13}$C NMR** (101 MHz, $CDCl_3$) δ 150.3, 145.6, 142.2, 130.7, 130.3, 130.2, 128.2, 126.2, 121.1, 120.7, 117.1, 111.5. **HRMS** $[M-H]^-$ calculated for $[C_{12}H_5BrCl_3O_2]^-$ 364.8544, found 364.8544, Δ= 0.0 ppm.

(8*R*,9*S*,13*S*,14*S*)-4-bromo-3-hydroxy-13-methyl-6,7,8,9,11,12,13,14,15,16-decahydro-17H-cyclopenta[a]phenanthren-17-one

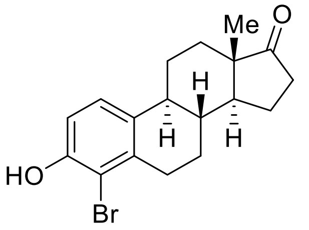


To a solution of estrone (541 mg, 2.00 mmol) in EtOH (30 mL) was added freshly prepared *N*-bromoacetamide (273 mg, 1.98 mmol, prepared according to ref. (*5*)) portionwise at rt. The reaction mixture was left to stir for 16 h, at which point the solvent was removed under reduced pressure. The crude material was purified *via* recrystallisation in EtOH to afford the title compound as a white solid (146 mg, 0.418 mmol, 21%).

**$^1$H NMR** (400 MHz, $CDCl_3$) δ 7.21 (d, *J* = 8.6 Hz, 1H), 6.90 (d, *J* = 8.5 Hz, 1H), 5.57 (s, 1H), 3.00 (dd, *J* = 17.8, 6.0 Hz, 1H), 2.76 (ddd, *J* = 18.0, 10.8, 6.1 Hz, 1H), 2.54 (dd, *J* = 18.7, 8.8 Hz, 1H), 2.41 (q, *J* = 6.2, 4.8 Hz, 1H), 2.30 (h, *J* = 4.8 Hz, 1H), 2.23 – 2.07 (m, 3H), 2.03 – 1.91 (m, 1H), 1.71 – 1.43 (m, 7H), 0.93 (s, 3H). **$^{13}$C NMR** (101 MHz, $CDCl_3$) δ 220.7, 150.3, 136.2, 133.7, 125.5, 113.7, 112.9, 50.3, 47.9, 44.1, 37.6, 35.9, 31.5, 31.0, 26.6, 26.2, 21.6, 13.8. $[\alpha]_D^{25}$= +86.9° (c. 1.0, $CHCl_3$).

Data in accordance with literature. (*12*)

2-bromo-3-methoxyphenol

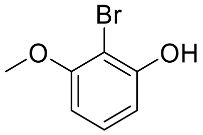


2,6-dimethoxybromobenzene (10.9 g, 50 mmol) was dissolved in $CH_2Cl_2$ (40 mL), and cooled to 0 °C. To this stirred solution under nitrogen was added $BBr_3$ (1M solution in $CH_2Cl_2$, 35 mL, 35 mmol, 0.7 eq.) dropwise. The reaction mixture was allowed to warm to rt and stirred for 5 h, after which water (75 mL) was added, and the organic layer separated. The organic layer was washed with brine (100 mL), dried ($MgSO_4$) then solvent removed under reduced pressure to yield crude product. The crude product was purified by flash column chromatography (0-20% EtOAc:petrol) to yield the title compound as a yellow oil which solidifies upon standing to a white solid (4.76 g, 23.4 mmol, 47%).

**$^1$H NMR** (500 MHz, $CDCl_3$) δ 7.19 (t, *J* = 8.3 Hz, 1H), 6.71 (dd, *J* = 8.3, 1.3 Hz, 1H), 6.51 (dd, *J* = 8.3, 1.3 Hz, 1H), 5.67 (s, 1H), 3.91 (s, 3H). **$^{13}$C NMR** (126 MHz, $CDCl_3$) δ 156.5, 153.5, 128.7, 108.6, 103.7, 100.0, 56.4.

Data in accordance with literature.(*13*)

4-chloro-[1,1'-biphenyl]-2-ol

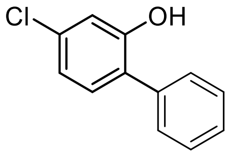


A 4 mL microwave vial was charged with 2-bromo-5-chlorophenol (208 mg, 1.00 mmol), phenyl boronic acid (183 mg, 1.5 mmol), diisopropylamine (0.28 mL, 2.0 mmol) and $Pd(OAc)_2$ (1.2mg, 5mol%). The vial was sealed and placed under an atmosphere of nitrogen *via* 3 evacuation-backfill cycles and $H_2O$ (2 mL) was added *via* syringe. The reaction mixture was stirred at 100 °C for 2 h before being diluted in brine (10 mL) and extracted with EtOAc (3 x 10 mL). The combined organic extracts were dried over $MgSO_4$ and evaporated under reduced pressure to afford a brown oil. The crude material was purified *via* column chromatography (0-5% EtOAc:petrol) to afford the desired product as a colourless oil (137 mg, 0.663 mmol, 66%).

**$^1$H NMR** (700 MHz, $CDCl_3$) δ 7.53 (m, 2H), 7.45 (m, 3H), 7.19 (d, *J* = 8.1 Hz, 1H), 7.04 (d, *J* = 2.0 Hz, 1H), 7.01 (dd, *J* = 8.1, 2.0 Hz, 1H), 5.32 (s, 1H). **$^{13}$C NMR** (101 MHz, $CDCl_3$) δ 153.1, 136.0, 134.3, 131.0, 129.5, 129.0, 128.2, 126.7, 121.1, 116.2.

Data in accordance with literature. (*14*)

3-bromo-4-chloro-[1,1'-biphenyl]-2-ol

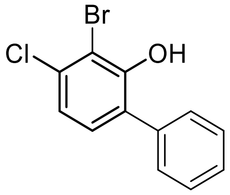


Synthesised according to general procedure **C** on a 1.3 mmol scale with respect to 4-chloro-[1,1'-biphenyl]-2-ol to afford the title compound as a white solid (153 mg, 0.54 mmol, 52%).

**$^1$H NMR** (400 MHz, $CDCl_3$) δ 7.56 – 7.46 (m, 4H), 7.45 – 7.39 (m, 1H), 7.23 (d, *J* = 8.3 Hz, 1H), 7.15 (d, *J* = 8.3 Hz, 1H), 5.89 (s, 1H). **$^{13}$C NMR** (101 MHz, $CDCl_3$) δ 150.6, 136.5, 133.7, 129.9, 129.1, 128.6, 128.0, 127.7, 122.0, 111.9. **HRMS** $[M-H]^-$ calculated for $[C_{12}H_7BrClO]^-$ 280.9374, found 280.9378, Δ= 1.4 ppm.

2-bromo-6-chloro-3-methylphenol

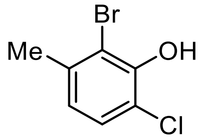


Synthesised according to general procedure **C** on a 15.3 mmol scale with respect to 2-chloro-5-methylphenol to afford the title compound as an orange oil (325 mg, 1.47 mmol, 12%).

**$^1$H NMR** (700 MHz, $CDCl_3$) δ 7.21 (d, *J* = 8.2 Hz, 1H), 6.80 (d, *J* = 8.2 Hz, 1H), 5.94 (s, 1H), 2.41 (s, 3H). **$^{13}$C NMR** (176 MHz, $CDCl_3$) δ 148.8, 138.0, 127.8, 122.2, 117.8, 113.3, 23.0. **HRMS** $[M-H]^-$ calculated for $[C_7H_5BrClO]^-$ 218.9218, found 218.9219, Δ= 0.5 ppm.

2,6-dibromo-3,5-dichlorophenol

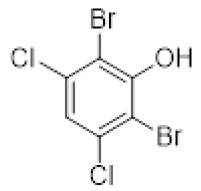


Synthesised according to **general procedure D** on a 4.10 mmol scale with respect to the phenol to afford the title compound as a white solid (315 mg, 0.982 mmol, 24%).

**$^1$H NMR** (400 MHz, $CDCl_3$) δ 7.27 (s, 1H, $H_5$), 6.23 (s, 1H, $H_1$). **$^{13}$C NMR** (101 MHz, $CDCl_3$) δ 152.0 ($C_2$), 134.4 ($C_4$), 122.5 ($C_5$), 109.3 ($C_3$). **HRMS** $[M-H]^-$ calculated for $[C_6HBr_2Cl_2O]^-$ 316.7777, found 316.7776, Δ = 0.3 ppm.

2,6-dibromo-3,5-dimethylphenol

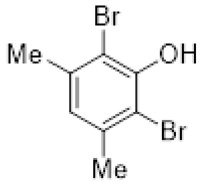


Synthesised according to **general procedure D** on a 8.19 mmol scale with respect to 3,5-dimethylphenol to afford the title compound as a white solid (1.96 g, 7.00 mmol, 85%).

**$^1$H NMR** (400 MHz, $CDCl_3$) δ 6.78 (s, 1H), 5.98 (s, 1H), 2.36 (s, 6H). **$^{13}$C NMR** (101 MHz, $CDCl_3$) δ 149.1, 137.5, 124.0, 109.3, 22.8.

Data in accordance with literature. (*15*)

3-fluoro-2-(4,4,5,5-tetramethyl-1,3,2-dioxaborolan-2-yl)phenol

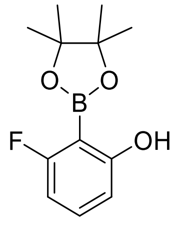


2-fluoro-6-methoxyphenylboronic acid (850 mg, 5 mmol) was dissolved in $CH_2Cl_2$ (20 mL), then cooled to 0 °C. $BBr_3$ (1M in $CH_2Cl_2$, 7.5 mL, 1.5 eq.) was added dropwise, and allowed to warm to rt for 2 h. Ice-cold water (5 mL) was added, then EtOAc (30 mL) was added and the organic layer extracted. The aqueous layer was extracted a further two times with EtOAc (20 mL), the organic layers combined, dried ($MgSO_4$), and solvent removed under reduced pressure. To the crude residue, pinacol (710 mg, 6 mmol, 1.2 eq.) was added, then $Et_2O$ (20 mL) was added and the resulting solution stirred at room temperature for 16 h. After this time, the solvent was removed under reduced pressure and the product purified by flash column chromatography (5-25% EtOAc:petrol) to yield the title compound as a colourless oil which solidifies on standing to a white solid (820 mg, 3.4 mmol, 69%).

**$^1$H NMR** (400 MHz, $CDCl_3$) δ 8.37 (s, 1H), 7.33 (q, *J* = 7.1 Hz, 1H), 6.70 (d, *J* = 8.2 Hz, 1H), 6.58 (t, *J* = 8.5 Hz, 1H), 1.42 (s, 12H). **$^{13}$C NMR** (101 MHz, $CDCl_3$) δ 167.8 (d, *J* = 251.5 Hz), 164.9 (d, *J* = 10.4 Hz), 134.5 (d, *J* = 11.4 Hz), 111.6, 106.4 (d, *J* = 23.9 Hz), 84.4, 24.8. **$^{19}$F NMR** (376 MHz, $CDCl_3$) δ -100.83.

Data in accordance with literature. (*16*)

3-chloro-2-(4,4,5,5-tetramethyl-1,3,2-dioxaborolan-2-yl)phenol

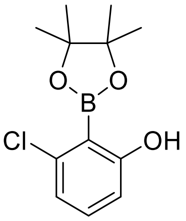

1-chloro-3-(methoxymethoxy)benzene (3.00 g, 17.4 mmol) was dissolved in THF (50 mL) and cooled to -78 °C. To this stirred solution, n-BuLi (1.6 M in hexane, 10.3 mL, 16.5 mmol) was added dropwise and stirred at -78 °C for 2 h. After this time, $^i$PrOBPin (4.1 mL, 20 mmol) was added and stirred at -78 °C for 1 h, then warmed to rt over 2 h. $NH_4Cl$ solution (sat., 30 mL) was added, then EtOAc (50 mL) and water (50 mL) and the organic layer separated. The aqueous layer was extracted twice more with EtOAc (2 x 50 mL), then the organic layers combined, dried ($MgSO_4$) and solvent removed under reduced pressure. This crude was dissolved in HCl in dioxane (4 M, 15 mL) and stirred at 60 °C for 1 h. EtOAc (50 mL) and water (50 mL) was added and the organic layer separated. The organic layer was washed with $NaHCO_3$ solution (sat., 30 mL), dried ($MgSO_4$) and solvent removed under reduced pressure. The crude product was purified by flash column chromatography (0-15% EtOAc:petrol) to yield the title compound as a pale-yellow oil (945 mg, 3.71 mmol, 21%).

**$^1$H NMR** (400 MHz, $CDCl_3$) δ 8.64 (s, 1H), 7.25 (t, *J* = 8.1 Hz, 1H), 6.92 (d, *J* = 7.9 Hz, 1H), 6.80 (d, *J* = 8.3 Hz, 1H), 1.42 (s, 12H). **$^{13}$C NMR** (101 MHz, $CDCl_3$) δ 165.2, 141.0, 133.5, 121.5, 114.5, 84.5, 24.8. **HRMS** $[M-H]^-$ calcd for $C_{12}H_{15}BClO_3^-$; 253.0808, found 253.0806, Δ= 0.8 ppm.

1-(methoxymethoxy)-3-(trifluoromethoxy)benzene

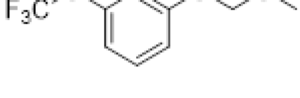

3-(trifluoromethoxy)phenol (2.5 mL, 20 mmol) was dissolved in THF (50 mL), and cooled to 0 °C. Sodium hydride (60% in mineral oil, 1.2 g, 30 mmol, 1.5 eq.) was added portionwise, and the mixture was stirred at room temperature for 30 min. The mixture was cooled to 0 °C, and MOMCl (2.1 M in PhMe, prepared according to literature procedure(*7*), 12 mL, 25 mmol, 1.25 eq.) was added dropwise. The reaction was allowed to warm to room temperature over 16 h, then $NH_4Cl$ solution (sat., 15 mL) was added. EtOAc (50 mL) and water (50 mL) was added and the organic layer separated. The aqueous layer was extracted with EtOAc (50 mL), the organic layers combined, dried ($MgSO_4$) and solvent removed under reduced pressure to yield the crude product. The product was purified by flash column chromatography (0-15% EtOAc:Petrol) to yield the title compound as a colourless oil (3.63 g, 16.4 mmol, 82%).

**$^1$H NMR** (400 MHz, $CDCl_3$) δ 7.31 (t, *J* = 8.3 Hz, 1H), 7.00 (ddd, *J* = 8.3, 2.4, 0.9 Hz, 1H), 6.94 (s, 1H), 6.89 (dd, *J* = 8.2, 2.2 Hz, 1H), 5.20 (s, 2H), 3.51 (s, 3H). **$^{13}$C NMR** (101 MHz, $CDCl_3$) δ 158.3, 150.0, 130.2,

120.4 (q, *J* = 258.0 Hz), 114.4, 114.0 (q, *J* = 1.1 Hz), 109.5 (q, *J* = 1.0 Hz), 94.5, 56.1. **$^{19}$F NMR** (376 MHz, $CDCl_3$) δ -58.78.

Data in accordance with literature.(*17*)

2-(4,4,5,5-tetramethyl-1,3,2-dioxaborolan-2-yl)-3-(trifluoromethoxy)phenol

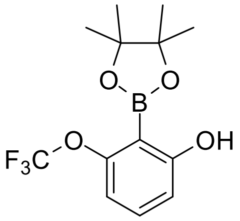


1-(methoxymethoxy)-3-(trifluoromethoxy)benzene (1.22 g, 5.5 mmol) was dissolved in THF (20 mL) and cooled to -78 °C. To this stirred solution, n-BuLi (1.6 M in hexane, 4.2 mL, 6.6 mmol, 1.2 eq.) was added dropwise and stirred at -78 °C for 2 h. After this time, $^{i}$PrOBPin (1.65 mL, 8.25 mmol, 1.5 eq.) was added and stirred at -78 °C for 1 h, then warmed to room temperature over 2 h. $NH_4Cl$ solution (sat.,10 mL) was added, then EtOAc (20 mL) and water (20 mL) and the organic layer separated. The aqueous layer was extracted twice more with EtOAc (2 x 20 mL), then the organic layers combined, dried ($MgSO_4$) and solvent removed under reduced pressure. The resulting crude was dissolved in HCl in dioxane (4 M, 10 mL), and stirred at 60 °C for 1 h. EtOAc (50 mL) and water (50 mL) were added, and the organic layer separated. The organic layer was washed with $NaHCO_3$ solution (sat., 10 mL), dried ($MgSO_4$) and solvent removed under reduced pressure. The crude product was purified by flash column chromatography (0-15% EtOAc:petrol) to yield the title compound as a yellow oil, which solidified on standing to a white solid (503 mg , 1.65 mmol, 33%).

**$^{1}$H NMR** (400 MHz, $CDCl_3$) δ 8.45 (s, 1H), 7.34 (t, *J* = 8.2 Hz, 1H), 6.84 (d, *J* = 8.4 Hz, 1H), 6.76 (d, *J* = 8.1 Hz, 1H), 1.37 (s, 12H). **$^{13}$C NMR** (101 MHz, $CDCl_3$) δ 164.9, 154.2 (q, *J* = 1.7 Hz), 133.8, 120.3 (q, *J* = 255.9 Hz), 114.9, 112.9, 84.5, 24.7. **$^{19}$F NMR** (376 MHz, $CDCl_3$) δ -58.66. **HRMS** $[M-H]^-$ calcd for $C_{13}H_{15}BF_3O_4^-$; 303.1021, found 303.1017, Δ= 1.3 ppm.

2-(2-fluoro-6-methoxyphenyl)-4,4,5,5-tetramethyl-1,3,2-dioxaborolane

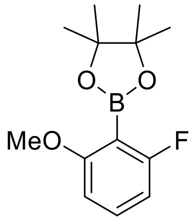


2-fluoro-6-methoxyphenylboronic acid (340 mg, 2 mmol) and pinacol (242 mg, 2.05 mmol) were dissolved in THF (10 mL) and $MgSO_4$ (1 g) added. The mixture was allowed to stir at room temperature for 16 h, after which the mixture was filtered and the solvent removed to yield the product as a colourless oil which solidified upon standing to a white solid (496 mg, 1.97 mmol, 98%).

**$^{1}$H NMR** (400 MHz, $CDCl_3$) δ 7.27 (td, *J* = 8.2, 7.0 Hz, 1H), 6.66 – 6.57 (m, 2H), 3.80 (s, 3H), 1.38 (s, 12H). **$^{13}$C NMR** (101 MHz, $CDCl_3$) δ 166.2 (d, *J* = 243.8 Hz), 164.0 (d, *J* = 13.4 Hz), 132.1 (d, *J* = 10.6 Hz), 107.7 (d, *J* = 24.2 Hz), 105.8 (d, *J* = 2.8 Hz), 84.1, 56.0, 24.7. **$^{19}$F NMR** (471 MHz, $CDCl_3$) δ -105.15.

Data in accordance with literature. (*18*)

3-methyl-2-(4,4,5,5-tetramethyl-1,3,2-dioxaborolan-2-yl)phenol

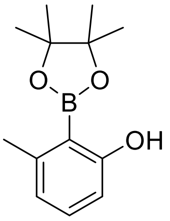

2-bromo-3-methylphenol (935 mg, 5 mmol) was dissolved in THF (40 mL) and cooled to -78 °C. *n*-BuLi (1.6 in THF, 8 mL, 12.5 mmol) was added dropwise, stirred at -78 °C for 30 min then allowed to warm to rt over 2 h. The solution was then cooled back to -78 °C and $^{i}$PrOBPin (3 mL, 15 mmol) was added. The solution was allowed to warm to rt over 16 h, then ammonium chloride solution (sat., 30 mL) was added. EtOAc (100 mL) and water (100 mL) was added and the layers separated. The aqueous layer was washed with EtOAc (2 x 50 mL), then the organic layers combined, washed with brine (100 mL), dried ($MgSO_4$) and solvent removed. The crude was purified by column chromatography to yield the product as a golden oil (287 mg, 1.23 mmol, 25%).

**$^{1}$H NMR** (500 MHz, $CDCl_3$) δ 8.56 (s, 1H), 7.24 (t, *J* = 7.8 Hz, 1H), 6.73 (t, *J* = 9.2, 8.0 Hz, 2H), 2.52 (s, 3H), 1.40 (s, 12H). **$^{13}$C NMR** (126 MHz, $CDCl_3$) δ 164.9, 147.2, 132.9, 121.7, 113.2, 83.9, 24.8, 22.9. **HRMS** $[M+2Li]^{2+}$ calcd for $C_{13}H_{19}BO_3Li_2^{2+}$; 124.0868, found 124.0869, Δ= 0.8 ppm.

ethyl (*E*)-3-(2-bromo-3-hydroxyphenyl)acrylate

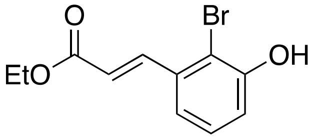

2-bromo-3-hydroxybenzaldehyde (402 mg, 2.0 mmol) was dissolved in anhydrous THF (8 mL) and cooled to 0°C. Sodium hydride (160 mg, 4.0 mmol) was added portionwise and the reaction mixture stirred for 1 h under $N_2$ atmosphere. Triethyl phosphonoacetate (0.44 mL, 2.2 mmol) was added dropwise and the reaction was stirred to r.t. overnight. The reaction mixture was then quenched with a saturated solution of $NH_4Cl$ and the mixture extracted three times with ethyl acetate. The organic layers were combined, dried with $MgSO_4$, filtered and the solvent removed under reduced pressure. The crude product was then purified with flash column chromatography (10 to 30% ethyl acetate: petroleum ether), giving the product as colourless solid (461 mg, 1.70 mmol, 85%)

**$^{1}$H NMR** (400 MHz, $CDCl_3$) δ 7.98 (d, *J* = 15.9 Hz, 1H), 7.25 – 7.16 (m, 2H), 7.05 (dd, *J* = 7.8, 1.8 Hz, 1H), 6.39 (d, *J* = 15.9 Hz, 1H), 5.82 (s, 1H), 4.29 (q, *J* = 7.1 Hz, 2H), 1.35 (t, *J* = 7.1 Hz, 3H). **$^{13}$C NMR** (101 MHz, $CDCl_3$) δ 166.6, 153.0, 142.9, 135.4, 128.8, 121.8, 119.9, 117.3, 113.4, 60.9, 14.4. **HRMS** $[M-H]^-$ calcd for $[C_{11}H_{10}BrO_3]^-$; 268.9819, found 268.9815, Δ = -1.5 ppm

## Reaction Optimisation (Table 1, extended)

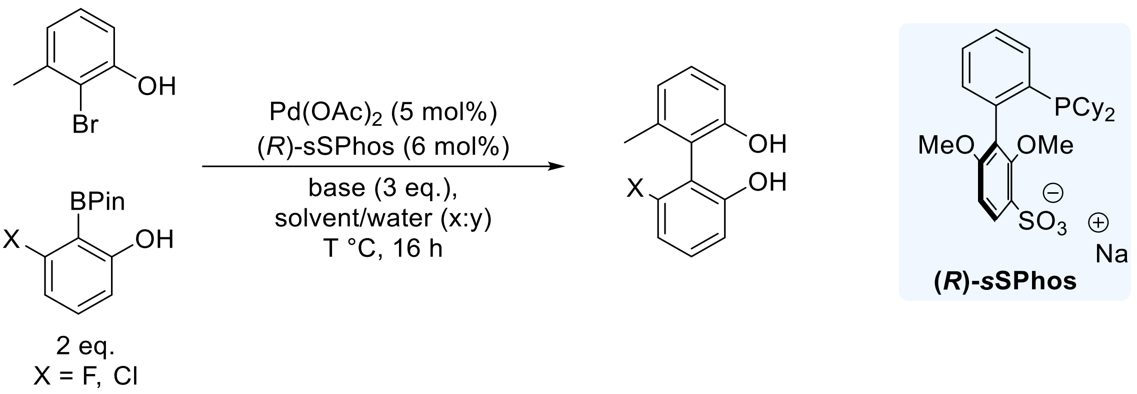


| Entry | X | solvent/water | base | T (°C) | Yield (%) | *ee* (%) |
|---|---|---|---|---|---|---|
| 1 | F | THF/water(19:1) | $K_3PO_4$ | 40 | 19 | 81 |
| 2 | F | MeCN/water (19:1) | $K_3PO_4$ | 40 | 5 | 74 |
| 3 | F | PhMe/water (19:1) | $K_3PO_4$ | 40 | 18 | 95 |
| 4 | F | PhMe/water (19:1) | $K_2CO_3$ | 40 | 40 | 90 |
| 5 | F | PhMe/water (19:1) | $Cs_2CO_3$ | 40 | 16 | 90 |
| 6 | F | PhMe/water (19:1) | $Na_2CO_3$ | 40 | 67 | 88 |
| **7** | **F** | **PhMe/water (19:1)** | **$Na_3PO_4$** | **40** | **73‡** | **92‡** |
| 8 | Cl | PhMe/water (19:1) | $Na_3PO_4$ | 40 | 11 | 96 |
| 9 | Cl | PhMe/water (19:1) | KF | 40 | 14[b] | 90 |
| 10[c] | Cl | PhMe | KF | 40 | 4 | 96 |
| 11[c] | Cl | PhMe | KF | 80 | 10 | 95 |
| **12[c,d]** | **Cl** | **PhMe** | **KF** | **80** | **44‡** | **97‡** |
| 13[c,d] | Cl | PhMe | KF | 100 | 23 | 90 |

[a]Yields determined by $^1H$ NMR of the crude reaction with dimethoxyethane as an internal standard, *ee* determined by chiral SFC analysis of the crude reaction. [b]As a significant amount of deborylated starting material was observed, anhydrous conditions were pursued. [c]2 eq. $MgSO_4$ additive. [d]Pd source changed to $Pd_2dba_3$ (2.5 mol%). ‡Isolated yield and ee.

Note: For other scope entries utilising 2-hydroxy-6-chlorophenylboronic acid pinacol ester it was found that if low yields were obtained, these could be increased by doubling the $Pd_2dba_3$ (5 mol%) and sSPhos loadings (12 mol%), as well as utilising 3 eq. of 2-hydroxy-6-chlorophenylboronic acid pinacol ester and stirring the reaction for 60 h, although this does not increase the yield in all cases.

To obtain NMR yields for entries utilising 2-hydroxy-6-fluorophenylboronic acid pinacol ester, the crude material was diluted in $CDCl_3$ containing a DME (0.03 mmol) internal standard and saturated aqueous $NH_4Cl$ solution (1 mL). The organic layer was isolated and the aqueous layer was extracted with further $CDCl_3$ (2 x 0.5 mL). The combined organic layers were passed through an $MgSO_4$ plug and a crude NMR was obtained.

To obtain NMR yields for entries utilising 2-hydroxy-6-chlorophenylboronic acid pinacol ester, the crude material was diluted in $CDCl_3$ containing a DME (0.03 mmol) internal standard and 3M aqueous HCl solution (1 mL). The organic layer was isolated and the aqueous layer was extracted with further $CDCl_3$ (2 x 0.5 mL). The combined organic layers were passed through an $MgSO_4$ plug and a crude NMR was obtained.

## Synthesis of Products (Scheme 1)

**General procedure A: asymmetric Suzuki cross-coupling to form 2,2'-biphenols with an *ortho*-fluoro BPin coupling partner**

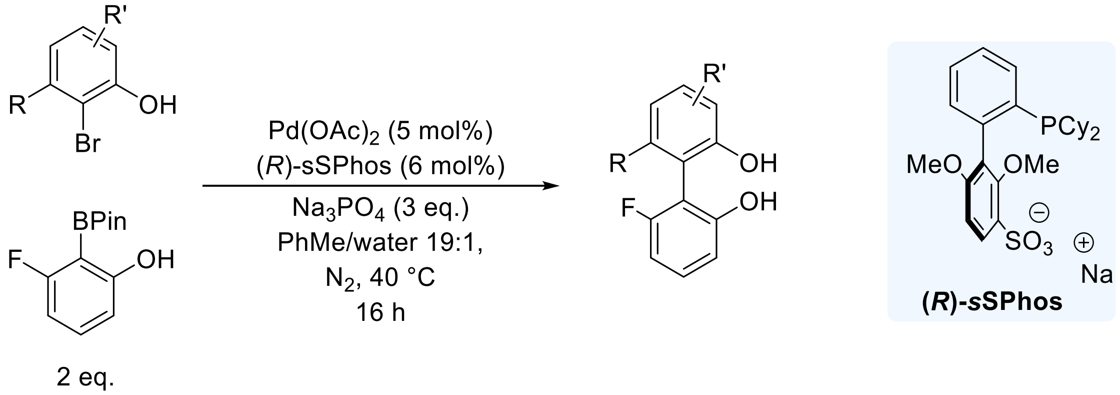


Aryl bromide (0.10 mmol), aryl boronic ester (0.20 mmol, 2 eq.), $Na_3PO_4$ (49.2 mg, 0.30 mmol), (*R*)-sSPhos (3.08 mg, 6 mol%) and $Pd(OAc)_2$ (1.12 mg, 5 mol%) were added to a 4 mL crimp vial. After 3 evacuation-backfill cycles with nitrogen, toluene (0.475 mL) and deionised water (0.025 mL) were added. The reaction mixture was stirred at 40 °C for 16 h and then the solvent was removed under a stream of air. The product was purified *via* column chromatography (petrol/EtOAc).

**General Procedure B: asymmetric Suzuki cross-coupling to form other 2,2'-biphenols.**

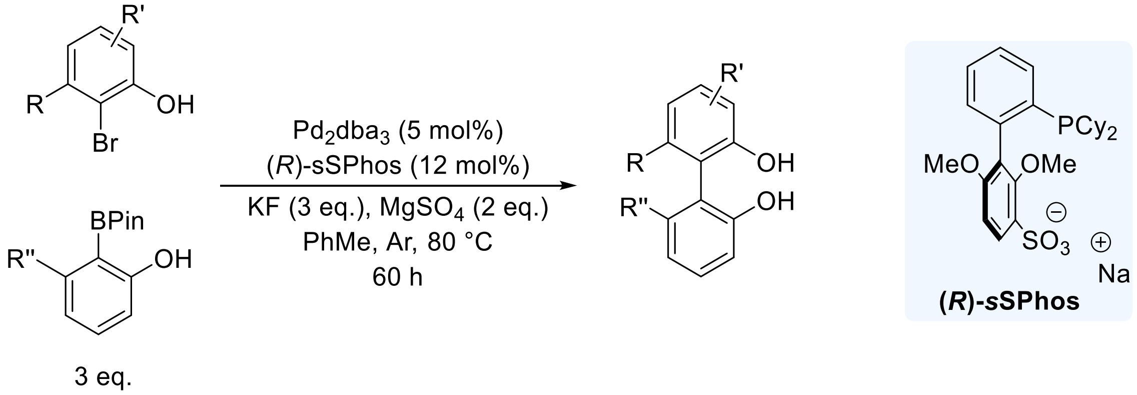


Aryl bromide (0.10 mmol), aryl boronic ester (0.30 mmol, 3 eq.), $Pd_2dba_3$ (4.6 mg, 5 mol%), (*R*)-sSPhos (6.2 mg, 12 mol%), KF (17.4 mg, 0.30 mmol, dried for 8h at 180 °C under vacuum) and $MgSO_4$ (24.1 mg, 0.20 mmol) were added to a 4 mL crimp vial. After 3 evacuation-backfill cycles with argon, toluene (0.5 mL) was added. The reaction mixture was then stirred at 80 °C for 60 h, after which solvent was removed under a stream of nitrogen. The product was purified *via* flash column chromatography (hexane/EtOAc).

(*R*)-fluoro-6'-methyl-[1,1'-biphenyl]-2,2'-diol (**3a**)

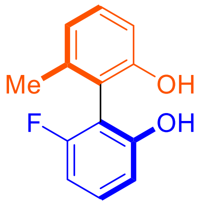


General procedure **A** was performed with 3-methyl-2-bromophenol (18.7 mg, 0.1 mmol) and 2-hydroxy-6-fluorophenylboronic acid pinacol ester (47 mg, 0.2 mmol). The product was purified by column chromatography (0-20% EtOAc:petrol) to yield the product as a brown oil (16 mg, 0.073 mmol, 73%, 92% *ee*).

**$^1$H NMR** (500 MHz, $CDCl_3$) δ 7.33 (td, *J* = 8.3, 6.5 Hz, 1H), 7.29 (d, *J* = 7.9 Hz, 1H), 6.95 (d, *J* = 7.6 Hz, 1H), 6.89 (m, 2H), 6.81 (td, *J* = 8.5, 1.0 Hz, 1H), 4.98 (s, 1H), 4.75 (s, 1H), 2.10 (s, 3H). **$^{13}$C NMR** (126 MHz, $CDCl_3$) δ 160.6 (d, *J* = 246.8 Hz), 154.8 (d, *J* = 5.9 Hz), 154.0, 139.9, 130.9 (d, *J* = 10.2 Hz), 130.6, 122.8, 114.9, 113.4, 111.6, 109.3 (d, *J* = 20.5 Hz), 108.0 (d, *J* = 22.3 Hz), 19.8. **$^{19}$F NMR** (376 MHz, $CDCl_3$) δ -112.87. **HRMS** $[M-H]^-$ calcd for $C_{13}H_{10}FO_2^-$; 217.0670, found 217.0667, Δ= 1.4 ppm. **Chiral SFC Analysis** (CHIRAL ART SJ, 90:10 $CO_2$:MeOH, 2.5 mL/min, 7.23 min [minor], 8.05 min [major]). $[\alpha]_D^{25}$= +15.6° (c 1.29, $CHCl_3$).

(*R*)-3-chloro-6'-fluoro-6-methyl-[1,1'-biphenyl]-2,2'-diol (**3b**)

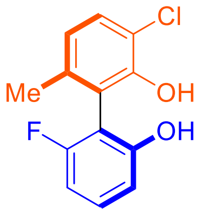


General procedure **A** was performed with 2-bromo-6-chloro-3-methylphenol (22.2 mg, 0.1 mmol) and 2-hydroxy-6-fluorophenylboronic acid pinacol ester (47.6 mg, 0.2 mmol). The product was purified by column chromatography (10-20% EtOAc:petrol) to yield the product as a colourless oil (13.8 mg, 0.055 mmol, 55%, 99% *ee*).

**$^1$H NMR** (400 MHz, $CDCl_3$) δ 7.36 (d, *J* = 8.3 Hz, 1H), 7.34 – 7.29 (m, 1H), 6.92 (d, *J* = 8.3 Hz, 1H), 6.87 (d, *J* = 8.3 Hz, 1H), 6.81 (t, *J* = 8.6 Hz, 1H), 2.12 (s, 3H). **$^{13}$C NMR** (101 MHz, $CDCl_3$) δ 160.3 (d, *J* = 245.9 Hz), 154.3 (d, *J* = 5.9 Hz), 149.6, 139.3, 130.3 (d, *J* = 10.3 Hz), 129.5, 123.2, 117.9, 117.1, 111.5 (d, *J* = 3.2 Hz), 110.7 (d, *J* = 20.6 Hz), 107.8 (d, *J* = 22.4 Hz), 19.6. **$^{19}$F NMR** (376 MHz, $CDCl_3$) δ -112.90. **HRMS** $[M-H]^-$ calculated for $[C_{13}H_9ClFO_2]^-$ 251.0281, found 251.0279, Δ = 0.8 ppm. **Chiral SFC Analysis** CHIRAL ART SJ ($CO_2$:MeOH, 85:15, 2.5 mL $min^{-1}$, 40 °C) $t_R$ = 4.55 (minor), 5.24 (major) minutes. $[\alpha]_D^{25}$**=** +22.0° (c. 1.0, $CHCl_3$).

(*R*)-6'-fluoro-4,6-dimethyl-[1,1'-biphenyl]-2,2'-diol (**3c**)

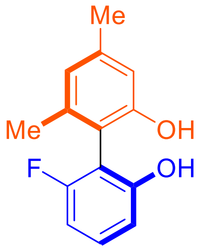


General procedure **A** was performed with 2-bromo-3,5-dimethylphenol (20.1 mg, 0.1 mmol) and 2-hydroxy-6-fluorophenylboronic acid pinacol ester (47.6 mg, 0.2 mmol). The product was purified by column chromatography (0-25% EtOAc:petrol) to yield the product as a colourless oil (18.2 mg, 0.078 mmol, 78%, 87% *ee*).

**$^1$H NMR** (400 MHz, $CDCl_3$) δ 7.33 (m, 1H), 6.89 (d, *J* = 8.3 Hz, 1H), 6.85 – 6.78 (m, 2H), 6.74 (s, 1H), 5.05 (s, 1H), 4.74 (s, 1H), 2.36 (s, 3H), 2.08 (s, 3H). **$^{13}$C NMR** (101 MHz, $CDCl_3$) δ 160.7 (d, *J* = 246.2 Hz), 155.0 (d, *J* = 5.6 Hz), 153.9, 141.0, 139.5, 130.7 (d, *J* = 10.3 Hz), 123.9, 114.1, 111.8, 111.5 (d, *J* = 3.0 Hz), 109.4 (d, *J* = 20.8 Hz), 107.9 (d, *J* = 22.4 Hz), 21.3, 20.0. **$^{19}$F NMR** (376 MHz, $CDCl_3$) δ -112.88. **HRMS** $[M\text{-}H]^-$ calculated for $[C_{14}H_{12}FO_2]^-$ 231.0827, found 231.0829, Δ = 0.9 ppm. **Chiral SFC Analysis** CHIRAL ART SJ ($CO_2$:MeOH, 93:07, 2.5 mL $min^{-1}$, 40 °C) $t_R$ = 6.64 (minor), 7.25 (major) minutes. $[\alpha]_D^{25}$ = +10.0° (c. 1.0, $CHCl_3$).

(*R*)-1-(2-fluoro-6-hydroxyphenyl)-5,6,7,8-tetrahydronaphthalen-2-ol (**3d**)

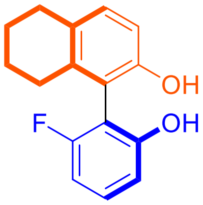


General procedure **A** was performed with 1-bromo-5,6,7,8-tetrahydronaphthalen-2-ol (22.7 mg, 0.1 mmol) and 2-hydroxy-6-fluorophenylboronic acid pinacol ester (47.6 mg, 0.2 mmol). The product was purified by column chromatography (10-30% EtOAc:petrol) to yield the product as a white solid (17.1 mg, 0.066 mmol, 66%, 93% *ee*).

**$^1$H NMR** (500 MHz, $CDCl_3$) δ 7.33 (td, *J* = 8.3, 6.5 Hz, 1H), 7.12 (d, *J* = 8.4 Hz, 1H), 6.89 (dt, *J* = 8.3, 0.8 Hz, 1H), 6.85 (d, *J* = 8.4 Hz, 1H), 6.81 (td, *J* = 8.5, 1.0 Hz, 1H), 5.04 (s, 1H), 4.63 (s, 1H), 3.07 – 2.56 (m, 2H), 2.53 – 2.19 (m, 2H), 1.87 – 1.63 (m, 4H). **$^{13}$C NMR** (126 MHz, $CDCl_3$) δ 160.6 (d, *J* = 246.1 Hz), 154.8 (d, *J* = 5.8 Hz), 151.6, 138.2, 131.8, 130.8 (d, *J* = 10.3 Hz), 130.4, 114.1, 113.3, 111.5 (d, *J* = 3.1 Hz), 109.3 (d, *J* = 21.1 Hz), 107.95 (d, *J* = 22.6 Hz), 29.2, 27.2, 22.9, 22.8. **$^{19}$F NMR** (376 MHz, $CDCl_3$) δ -112.85. **HRMS** $[M\text{-}H]^-$ calculated for $[C_{16}H_{14}FO_2]^-$ 257.0983, found 257.0986, Δ = 1.2 ppm. **Chiral SFC Analysis** CHIRAL ART SJ ($CO_2$:MeOH, 93:07, 2.5 mL $min^{-1}$, 40 °C) $t_R$ = 10.87 (minor), 11.76 (major) minutes. $[\alpha]_D^{25}$ = +46.2° (c. 1.0, $CHCl_3$).

(*S*)-6-chloro-6'-fluoro-[1,1'-biphenyl]-2,2'-diol (**3e**)

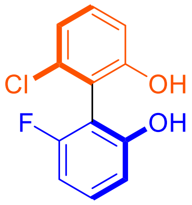

General procedure B was performed with 2-bromo-3-chlorophenol (20.7 mg, 0.1 mmol) and 2-hydroxy-6-fluorophenylboronic acid pinacol ester (61 mg, 0.26 mmol). The product was purified by column chromatography (0-20%EtOAc:hexane) to yield the product as a yellow oil (11.3 mg, 0.047 mmol, 47%, 88% *ee*).

**$^{1}$H NMR** (500 MHz, $CDCl_3$) δ 7.39 – 7.28 (m, 2H), 7.16 (d, *J* = 8.0 Hz, 1 H), 6.96 (d, *J* = 8.1 Hz, 1H), 6.88 – 6.81 (m, 2H), 5.12 (br s, 2H). **$^{13}$C NMR** (126 MHz, $CDCl_3$) δ 161.7, 159.8, 155.1, 154.7 (d, *J* = 5.8 Hz), 135.8, 131.4 (d, *J* = 10.5 Hz), 131.3, 122.2, 115.8, 114.7, 111.9 (d, *J* = 3.3 Hz), 108.2 (d, *J* = 21.8 Hz).**$^{19}$F NMR** (376 MHz, $CDCl_3$) δ -112.08. **HRMS** $[M-H]^-$ calcd for $C_{12}H_7ClFO_2^-$; 237.0128, found 237.0124, Δ= 1.69 ppm. **Chiral SFC Analysis** (CHIRAL ART SJ ($CO_2$:MeOH 90:10, 2.5 mL $min^{-1}$, 40 °C) $t_R$ = 12.12 (minor), 13.12 (major) minutes. $[\alpha]_D^{25}$= +22.2° (c 1.02, $CHCl_3$).

(*S*)-6-chloro-6'-fluoro-5-methyl-[1,1'-biphenyl]-2,2'-diol (**3f**)

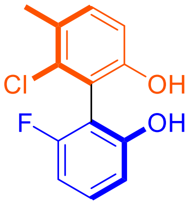


General procedure **A** was performed with 2-bromo-3-chloro-4-methylphenol (22.1 mg, 0.1 mmol) and 2-hydroxy-6-fluorophenylboronic acid pinacol ester (47.6 mg, 0.2 mmol). The product was purified by column chromatography (10-20% EtOAc:petrol) to yield the product as a colourless oil (12.6 mg, 49.8 µmmol, 50%, 88% *ee*).

**$^{1}$H NMR** (400 MHz, $CDCl_3$) δ 7.38 (td, *J* = 8.3, 6.5 Hz, 1H), 7.27 (m, 1H), 6.88 (m, 3H), 4.97 (s, 1H), 4.79 (s, 1H), 2.40 (s, 3H). **$^{13}$C NMR** (126 MHz, $CDCl_3$) δ 160.8 (d, *J* = 207.3 Hz), 154.8 (d, *J* = 5.4 Hz), 152.9, 135.2, 132.6, 131.3 (d, *J* = 10.4 Hz), 129.2, 115.4, 114.3, 111.9 (d, *J* = 19.4 Hz), 108.3 (d, *J* = 22.3 Hz), 108.1 (d, *J* = 22.0 Hz), 20.0. **$^{19}$F NMR** (376 MHz, $CDCl_3$) δ -112.35. **HRMS** $[M-H]^-$ calculated for $[C_{13}H_9ClFO_2]^-$ 251.0281, found 251.0283, Δ = 0.8 ppm. **Chiral SFC Analysis** CHIRAL ART SJ ($CO_2$:MeOH, 90:10, 2.5 mL $min^{-1}$, 40 °C) $t_R$ = 10.70 (minor), 11.71 (major) minutes. $[\alpha]_D^{25}$= +11.0° (c. 0.45, $CHCl_3$).

(*S*)-4,6-dichloro-6'-fluoro-[1,1'-biphenyl]-2,2'-diol (**3g**)

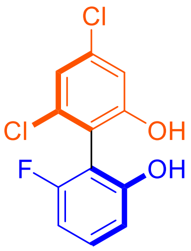


General procedure **A** was performed with 2-bromo-3,5-dichlorophenol (24.2 mg, 0.1 mmol) and 2-hydroxy-6-fluorophenylboronic acid pinacol ester (47.6 mg, 0.2 mmol). The product was purified by column chromatography (10-50% EtOAc:petrol) to yield the product as a colourless oil (22.7 mg, 0.083 mmol, 83%, 88% *ee*).

**$^{1}$H NMR** (400 MHz, $CDCl_3$) δ 7.38 (m, 1H), 7.23 – 7.14 (d, J = 1.6 Hz, 1H), 7.03 (d, J = 1.8 Hz, 1H), 6.85 (dt, J = 15.5, 7.5 Hz, 2H), 5.11 (s, 1H). **$^{13}$C NMR** (101 MHz, $CDCl_3$) δ 160.7 (d, *J* = 248.4 Hz), 155.4, 154.7 (d, *J* = 5.3 Hz), 136.4, 136.3, 131.8 (d, *J* = 10.4 Hz), 122.3, 115.3, 114.7, 112.0 (d, *J* = 3.2 Hz), 108.4 (d, *J* = 22.0 Hz), 107.4 (d, *J* = 19.9 Hz). **$^{19}$F NMR** (376 MHz, $CDCl_3$) δ -111.70. **HRMS** $[M-H]^-$ calculated for

$[C_{12}H_6Cl_2FO_2]^-$ 270.9734, found 270.9732, Δ = 0.7 ppm. **Chiral SFC Analysis** CHIRAL PAK IG ($CO_2$:MeOH, 90:10, 2.5 mL min$^{-1}$, 40 °C) $t_R$ = 7.24 (major), 9.25 (minor) minutes. $[\alpha]_D^{25}$= +43.1° (c. 0.72, $CHCl_3$).

(*S*)-3,6-dichloro-6'-fluoro-[1,1'-biphenyl]-2,2'-diol (**3h**)

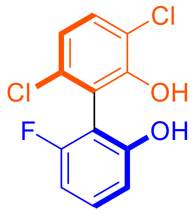


General procedure A was performed with 2-bromo-3,6-dichlorophenol (24.2 mg, 0.1 mmol) and 2-hydroxy-6-fluorophenylboronic acid pinacol ester (47.6 mg, 0.2 mmol). The product was purified by column chromatography (0-25% EtOAc:petrol) to yield the product as a colourless oil (17.7 mg, 0.065 mmol, 65%, 97% *ee*).

**$^1$H NMR** (400 MHz, $CDCl_3$) δ 7.41 (d, *J* = 8.7 Hz, 1H), 7.35 (m, 1H), 7.13 (d, *J* = 8.6 Hz, 1H), 6.84 (m, 2H), 5.77 (s, 1H), 5.02 (s, 1H). **$^{13}$C NMR** (101 MHz, $CDCl_3$) δ 160.4 (d, *J* = 247.2 Hz), 154.3 (d, *J* = 5.8 Hz), 150.7, 134.9, 130.9 (d, *J* = 10.5 Hz), 130.1, 122.3, 119.1, 117.9, 111.8 (d, *J* = 3.1 Hz), 109.6 (d, *J* = 20.0 Hz), 107.9 (d, *J* = 22.1 Hz). **$^{19}$F NMR** (376 MHz, $CDCl_3$) δ -112.07. **HRMS** [M-H]$^-$ calculated for $[C_{12}H_6Cl_2FO_2]^-$ 270.9734, found 270.9735, Δ = 0.4 ppm. **Chiral SFC Analysis** CHIRAL ART SJ ($CO_2$:MeOH, 85:15, 2.5 mL min$^{-1}$, 40 °C) $t_R$ = 7.55 (minor), 8.49 (major) minutes. $[\alpha]_D^{25}$**=** +19.8° (c. 1.0, $CHCl_3$).

tert-butyl (*S*)-(6-chloro-2'-fluoro-2,6'-dihydroxy-[1,1'-biphenyl]-3-yl)carbamate (**3i**)

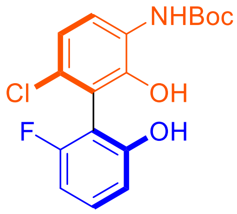


General procedure **A** was performed with tert-butyl (3-bromo-4-chloro-2-hydroxyphenyl)carbamate (32.3 mg, 0.1 mmol) and 2-hydroxy-6-fluorophenylboronic acid pinacol ester (47.6 mg, 0.2 mmol). The product was purified by column chromatography (0-25% EtOAc:petrol) to yield the product as a brown oil (19.4 mg, 0.055 mmol, 55%, 93% *ee*).

**$^1$H NMR** (400 MHz, $CDCl_3$) δ 7.52 (d, *J* = 8.7 Hz, 1H), 7.35 (m, 1H), 7.11 (d, *J* = 8.7 Hz, 1H), 6.85 (m, 3H), 5.42 (s, 1H), 1.54 (s, 9H). **$^{13}$C NMR** (126 MHz, $CDCl_3$) δ160.6 (d, *J* = 247.4 Hz), 154.6 (d, *J* = 5.5 Hz), 154.2, 145.5, 131.0 (d, *J* = 10.4 Hz), 130.3, 125.5, 121.9, 121.8, 117.9, 112.0 (d, *J* = 3.1 Hz), 109.5 (d, *J* = 20.9 Hz), 108.0 (d, *J* = 22.1 Hz), 82.3, 28.2. **$^{19}$F NMR** (376 MHz, $CDCl_3$) δ -111.56. **HRMS** [M-H]$^-$ calculated for $[C_{17}H_{16}ClFNO_4]^-$ 352.0757, found 352.0756, Δ = 0.3 ppm. **Chiral SFC Analysis** CHIRAL PAK IG ($CO_2$:MeOH, 90:10, 2.5 mL min$^{-1}$, 40 °C) $t_R$ = 6.84 (minor), 13.49 (major) minutes. $[\alpha]_D^{25}$ = +33.9 ° (c. 1.0, $CHCl_3$).

(*S*)-6-chloro-6'-fluoro-3-methoxy-[1,1'-biphenyl]-2,2'-diol (**3j**)

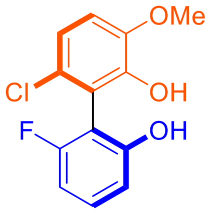


General procedure **A** was performed with 2-bromo-3-chloro-6-methoxyphenol (23.8 mg, 0.1 mmol) and 2-hydroxy-6-fluorophenylboronic acid pinacol ester (47.6 mg, 0.2 mmol). The product was purified by column chromatography (10-30% EtOAc:petrol) to yield the product as a colourless oil (15.2 mg, 0.056 mmol, 56%, 98% *ee*).

**$^1$H NMR** (500 MHz, $CDCl_3$) δ 7.32 (td, *J* = 8.3, 6.5 Hz, 1H), 7.10 (d, *J* = 8.7 Hz, 1H), 6.93 (d, *J* = 8.7 Hz, 1H), 6.87 (dt, *J* = 8.3, 0.9 Hz, 1H), 6.81 (ddd, *J* = 8.9, 8.3, 1.0 Hz, 1H), 5.94 (br s, 1H), 4.96 (br s, 1H), 3.97 (s, 3H). **$^{13}$C NMR** (101 MHz, $CDCl_3$) δ 160.5 (d, *J* = 246.6 Hz), 154.4 (d, *J* = 5.8 Hz), 145.6, 145.0, 130.4 (d, *J* = 10.5 Hz), 127.3, 120.7, 115.6, 111.8, 111.6 (d, *J* = 3.1 Hz), 110.2 (d, *J* = 20.0 Hz), 107.7 (d, *J* = 22.3 Hz), 56.3. **$^{19}$F NMR** (376 MHz, $CDCl_3$) δ -113.34. **HRMS** $[M-H]^-$ calculated for $[C_{13}H_9ClFO_3]^-$ 267.0230, found 267.0228, Δ = 0.7 ppm. **Chiral SFC Analysis** CHIRAL ART SJ ($CO_2$:MeOH, 85:15, 2.5 mL $min^{-1}$, 40 °C) $t_R$ = 9.91 (minor),$[\boldsymbol{\alpha}]_D^{25}$ = +19.8° (c. 1.0, $CHCl_3$).

(*R*)-6-fluoro-6'-(2-nitroethyl)-[1,1'-biphenyl]-2,2'-diol (**3k**)

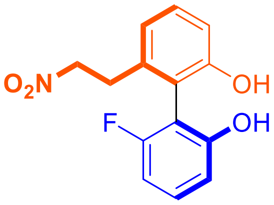


A modification of general procedure **A** using $Pd(OAc)_2$ (2.2 mg, 10 mol%), *R*-sSPhos (6.2 mg, 12 mol%), $Na_3PO_4$ (49 mg, 0.30 mmol)**,** 2-bromo-3-(2-nitroethyl)phenol (25 mg, 0.1 mmol) and 2-hydroxy-6-fluorophenylboronic acid pinacol ester (119 mg, 0.5 mmol) and reacted for 63 h. The product was purified by column chromatography (10-30% EtOAc:petrol) to yield the product as a colourless oil (12 mg, 0.043 mmol, 43%, 85% ee)

**$^1$H NMR** (400 MHz, $CDCl_3$) δ 7.39 – 7.31 (m, 2H), 6.99 (dd, *J* = 8.2, 1.1 Hz, 1H), 6.95 – 6.89 (m, 2H), 6.84 (td, *J* = 8.5, 1.1 Hz, 1H), 5.00 (s, 2H), 4.46 – 4.33 (m, 2H), 3.16 – 3.03 (m, 2H). **$^{13}$C NMR** (101 MHz, $CDCl_3$) δ 160.5 (d, *J* = 246.2 Hz), 154.9 (d, *J* = 5.3 Hz), 154.4, 137.3, 131.5 (d, *J* = 10.3 Hz), 131.1, 122.0, 115.6, 115.4, 112.2 (d, *J* = 3.1 Hz), 108.6 (d, *J* = 20.8 Hz), 108.4 (d, *J* = 22.2 Hz), 75.1, 31.3. **$^{19}$F NMR** (376 MHz, $CDCl_3$) δ -112.28. **HRMS** (FTMS -p NSI) $[M-H]^-$ calcd for $[C_{14}H_{11}FNO_4]^-$; 276.0678, found 276.0674, Δ= -1.4 ppm. **Chiral-SFC** (ChiralPAK IE ($CO_2$:MeOH 95:05, 2.5 $mLmin^{-1}$, 40 °C) $t_R$ = 10.99 (major), 12.01 (minor) minutes. $[\boldsymbol{\alpha}]_D^{25}$ = +22.0° (c. 1.0, $CHCl_3$).

(*R*)-6-(((*tert*-butyldimethylsilyl)oxy)methyl)-6'-fluoro-3-methoxy-[1,1'-biphenyl]-2,2'-diol (**3l**)

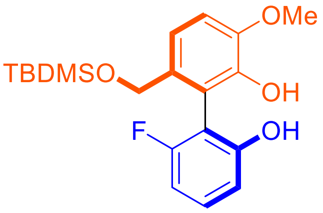


General procedure **A** was performed with 2-bromo-3-(((*tert*-butyldimethylsilyl)oxy)methyl)-6-methoxyphenol (35 mg,0.1 mmol) and 2-hydroxy-6-fluorophenylboronic acid pinacol ester (60 mg, 0.25 mmol). The product was purified by column chromatography (10-40% EtOAc:petrol) to yield the product as a colourless oil (33.7 mg, 0.089 mmol, 89%, 98% *ee*).

**$^1$H NMR** (400 MHz, Acetone-$d_6$) δ 7.21 (td, *J* = 8.2, 6.6 Hz, 1H), 7.03 (d, *J* = 8.4 Hz, 1H), 6.98 (d, *J* = 8.4 Hz, 1H), 6.78 (d, *J* = 8.2 Hz, 1H), 6.72 – 6.64 (m, 1H), 4.46 (q, *J* = 12.8 Hz, 2H), 3.87 (s, 3H), 0.87 (s, 9H), -0.03 (d, *J* = 3.1 Hz, 6H).**$^{13}$C NMR** (101 MHz, Acetone-$d_6$) δ 162.1 (d, *J* = 242.2 Hz), 157.4 (d, *J* = 7.2 Hz), 147.4, 145.6, 134.5, 130.1 (d, *J*= 10.8 Hz), 118.4, 117.3 (d, *J* = 1.4 Hz), 112.8, 112.5 (d, *J* = 2.8 Hz), 111.5, 107.2 (d, *J* = 23.3 Hz), 63.9, 56.4, 26.4, 19.0, -5.20, -5.21.**$^{19}$F NMR** (376 MHz, Acetone-*d6*) δ -114.79. **HRMS** [M-H]$^-$ calcd for [$C_{20}H_{26}FO_4Si$]$^-$; 377.1590, found 377.1584, Δ= -1.6 ppm. **Chiral-SFC** (CHIRAL PAK IE, 97:03 CO2:MeOH, 2.5 mLmin$^{-1}$, 40 °C, 7.02 (major) 9.11 (minor) minutes. $[\alpha]_D^{25}$ = -5.7° (c. 1.0, $CHCl_3$)

<u>*tert*-butyl (*R*)-((2'-fluoro-6,6'-dihydroxy-5-methoxy-[1,1'-biphenyl]-2-yl)methyl)carbamate (**3m**)</u>

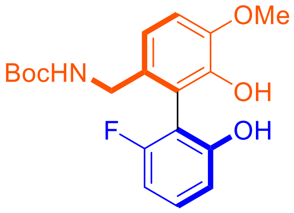


General procedure **A** was performed with *tert*-butyl (2-bromo-3-hydroxy-4-methoxybenzyl)carbamate (33 mg, 0.1 mmol) and 2-hydroxy-6-fluorophenylboronic acid pinacol ester (60 mg, 0.25 mmol). The product was purified by column chromatography (5-40% EtOAc:petrol) to yield the product as a colourless oil (29.8 mg, 0.082 mmol, 82%, 87% *ee*).

**$^1$H NMR** (400 MHz, $CDCl_3$) δ 7.27 – 7.19 (m, 1H), 6.99 (d, *J* = 8.3 Hz, 1H), 6.91 (d, *J* = 8.4 Hz, 1H), 6.81 (d, *J* = 8.2 Hz, 1H), 6.77 – 6.71 (m, 1H), 6.28 (s, 1H), 5.79 (s, 1H), 4.83 (t, *J* = 6.1 Hz, 1H), 4.09 – 3.95 (m, 2H), 3.91 (s, 3H), 1.39 (s, 9H). **$^{13}$C NMR** (101 MHz, $CDCl_3$) δ 160.6 (d, *J* = 244.1 Hz), 156.1, 155.1 (d, *J* = 5.9 Hz), 146.1, 144.0, 131.9, 130.1 (d, *J* = 10.4 Hz), 119.9, 112.2, 111.2, 110.7 (d, *J* = 20.4 Hz), 107.5 (d, *J* = 22.5 Hz), 75.3, 56.2, 28.5, 24.9. **$^{19}$F NMR** (376 MHz, $CDCl_3$) δ -111.78. **HRMS** [M-H]$^-$ calcd for [$C_{19}H_{21}FNO_5$]$^-$; 362.1409, found 362.1403, Δ= 1.7 ppm. **Chiral-SFC** (CHIRAL ART SB, 90:10 $CO_2$:MeOH, 2.5 mLmin$^{-1}$, 40 °C, 7.88 (major), 12.46 (minor) minutes. $[\alpha]_D^{25}$ = +5.4° (c. 1.0, $CHCl_3$).

<u>(*S*)-6-chloro-3-(2,4-dichlorophenoxy)-6'-fluoro-[1,1'-biphenyl]-2,2'-diol (**3n**)</u>

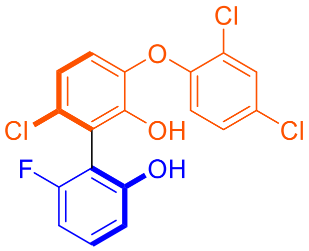

General procedure **A** was performed with 2-bromo-3-chloro-6-(2,4-dichlorophenoxy)phenol (36.8 mg, 0.1 mmol) and 2-hydroxy-6-fluorophenylboronic acid pinacol ester (47.6 mg, 0.2 mmol). The product was purified by column chromatography (0-30% EtOAc:petrol) to yield the product as a white solid (21.8 mg, 0.055 mmol, 55%, 89% *ee*).

**$^1$H NMR** (400 MHz, $CDCl_3$) δ 7.53 (d, J = 2.5 Hz, 1H), 7.35 (m, 1H), 7.29 (m, 1H), 7.07 (d, J = 8.8 Hz, 2H), 6.88 (d, J = 8.2 Hz, 1H), 6.86 – 6.82 (m, 1H), 6.80 (d, J = 8.8 Hz, 1H), 5.90 (s, 1H), 5.01 (s, 1H). **$^{13}$C NMR** (101 MHz, $CDCl_3$) δ 160.5 (d, *J* = 247.1 Hz), 154.4 (d, *J* = 5.6 Hz), 150.1, 146.1, 142.7, 130.8, 130.8, 130.7 (d, *J* = 2.7 Hz), 130.5, 128.4, 126.6, 121.3, 121.3, 118.1, 117.8, 111.8 (d, *J* = 3.0 Hz), 109.6 (d, *J* = 20.0 Hz), 107.9 (d, *J* = 22.2 Hz). **$^{19}$F NMR** (376 MHz, $CDCl_3$) δ -112.05. **HRMS** $[M-H]^-$ calculated for $[C_{18}H_9Cl_3FO_3]^-$ 396.9607, found 396.9606, Δ = 0.3 ppm. **Chiral SFC Analysis** CHIRAL ART SJ ($CO_2$:MeOH, 80:20, 2.5 mL min$^{-1}$, 40 °C) $t_R$ = 7.80 (major), 8.89 (minor) minutes. $[\alpha]_D^{25}$= +16.0° (c. 1.0, $CHCl_3$).

<u>(*4R*,*8R*,*9S*,*13S*,*14S*)-4-(2-fluoro-6-hydroxyphenyl)-3-hydroxy-13-methyl-6,7,8,9,11,12,13,14,15,16-decahydro-17H-cyclopenta[a]phenanthren-17-one (**3o**)</u>

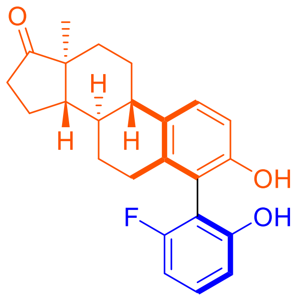


General procedure **A** was performed with (8*R*,9*S*,13*S*,14*S*)-4-bromo-3-hydroxy-13-methyl-6,7,8,9,11,12,13,14,15,16-decahydro-17H-cyclopenta[a]phenanthren-17-one (34.9 mg, 0.1 mmol) and 2-hydroxy-6-fluorophenylboronic acid pinacol ester (47.6 mg, 0.2 mmol). The product was purified by column chromatography (10-30% EtOAc:petrol) to yield the product as a colourless oil (24.1 mg, 0.063 mmol, 63%, 19:1 *dr*).

**$^1$H NMR** (400 MHz, $CDCl_3$) δ 7.41 – 7.29 (m, 2H), 6.90 (d, *J* = 8.4 Hz, 2H), 6.82 (t, *J* = 8.4 Hz, 1H), 5.11 (s, 1H), 4.80 (s, 1H), 2.64 – 2.38 (m, 4H), 2.31 (td, *J* = 10.8, 4.0 Hz, 1H), 2.15 (dt, *J* = 18.7, 8.7 Hz, 1H), 1.99 (dtd, *J* = 26.3, 11.4, 10.4, 6.4 Hz, 3H), 1.65 – 1.50 (m, 5H), 1.38 (td, *J* = 12.2, 6.1 Hz, 1H), 0.95 (s, 3H). **$^{13}$C NMR** (101 MHz, $CDCl_3$) δ 221.0, 160.7 (d, *J* = 246.1 Hz), 154.6 (d, *J* = 5.8 Hz), 150.0, 137.9, 133.1, 130.8 (d, *J* = 10.3 Hz), 128.0, 114.3, 113.4, 111.6 (d, *J* = 3.1 Hz), 109.5 (d, *J* = 21.1 Hz), 108.0 (d, *J* = 22.3 Hz), 50.4, 48.0, 44.3, 37.7, 35.0, 31.6, 27.5, 26.4, 26.0, 24.8, 21.5, 13.9. **$^{19}$F NMR** (376 MHz, $CDCl_3$) δ -112.92. **HRMS** $[M-H]^-$ calculated for $[C_{24}H_{24}FO_3]^-$ 379.1715, found 379.1709, Δ = 1.6 ppm. **Chiral SFC Analysis to assign dr:** CHIRAL ART SJ ($CO_2$:MeOH, 85:15, 2.5 mL min$^{-1}$, 40 °C) $t_R$ = 12.34 (major), 13.83 (minor) minutes. $[\alpha]_D^{25}$= +50.6° (c. 1.0, $CHCl_3$).

<u>(*R*)-6-chloro-6'-methyl-[1,1'-biphenyl]-2,2'-diol (**3p**)</u>

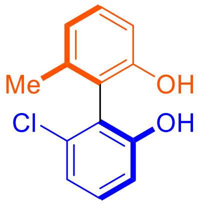


General procedure **B** was performed with 2-bromo-3-methylphenol (18.7 mg, 0.1 mmol), 2-hydroxy-6-chlorophenylboronic acid pinacol ester (50.9 mg, 0.2 mmol), $Pd_2dba_3$ (2.3 mg, 0.0025 mmol), (*R*)-sSPhos (3.1 mg, 0.006 mmol), KF (17.4 mg, 0.3 mmol) and $MgSO_4$ (24.1 mg, 0.2 mmol) in PhMe at 80

°C for 16 h. The product was purified by column chromatography (0-20% EtOAc:hexane) to yield the product as a white crystalline solid (10.3 mg, 0.044 mmol, 44%, 97% *ee*).

**$^1$H NMR** (400 MHz, $CDCl_3$) δ 7.28 (t, *J* = 8.3 Hz, 1H), 7.27 (t, *J* = 7.7 Hz, 1H), 7.12 (d, *J* = 8.3 Hz, 1H), 6.97 (d, *J* = 8.3 Hz, 1H), 6.93 (d, *J* = 7.6 Hz, 1H), 6.88 (d, *J* = 8.2 Hz, 1H), 4.60 (br s, 2H), 2.04 (s, 3H). **$^{13}$C NMR** (101 MHz, $CDCl_3$) δ 154.8, 153.8, 139.4, 135.1, 130.9, 130.8, 122.8, 122.0, 120.0, 118.2, 114.3, 113.6, 19.6. **HRMS** $[M-H]^-$ calcd for $C_{13}H_{10}ClO_2$; 233.0375, found 233.0369, Δ= 2.6 ppm. **Chiral SFC Analysis** (CHIRAL PAK IG($CO_2$:MeOH 90:10, 2.5 mL min$^{-1}$, 40 °C) $t_R$ = 7.18 (major), 7.92 (minor) minutes. $[\alpha]_D^{25}$= -5.7° (c 0.26, $CHCl_3$).

(*S*)-6-chloro-6'-methoxy-[1,1'-biphenyl]-2,2'-diol (**3q**)

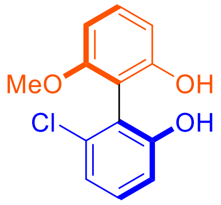


General procedure **B** was performed with 2-bromo-3-methoxyphenol (20.3 mg, 0.1 mmol), 2-hydroxy-6-chlorophenylboronic acid pinacol ester (50.9 mg, 0.2 mmol), $Pd_2dba_3$ (2.3 mg, 0.0025 mmol), (*R*)-sSPhos (3.1 mg, 0.006 mmol), KF (17.4 mg, 0.3 mmol) and $MgSO_4$ (24.1 mg, 0.2 mmol) in PhMe at 80 °C for 16 h. The product was purified by column chromatography (0-20% EtOAc:hexane) to yield the product as a yellow oil, containing a 23 mol% impurity of 6,6'-dichloro-[1,1'-biphenyl]-2,2'-diol (19.5 mg, 0.063 mmol (adjusted), 63% (adjusted), 87% *ee*).

**$^1$H NMR** (400 MHz, $CDCl_3$) δ 7.38 (t, *J* = 8.7 Hz, 1H), 7.31 (t, *J* = 8.3 Hz, 1H), 7.15 (d, *J* = 8.0 Hz, 1H), 7.00 (d, *J* = 8.0 Hz, 1H), 6.74 (d, *J* = 8.3 Hz, 1H), 6.66 (d, *J* = 8.3 Hz, 1H), 4.92 (br s, 2H), 3.79 (s, 3H). **$^{13}$C NMR** (101 MHz, $CDCl_3$) δ 158.3, 155.3, 154.8, 135.6, 131.4, 130.7, 121.9, 117.8, 114.4, 109.0, 107.7, 103.6, 56.0. **HRMS** $[M-H]^-$ calcd for $C_{13}H_{10}ClO_3$; 249.0312, found 249.0324, Δ= -4.65 ppm. **Chiral SFC Analysis** (CHIRAL PAK IG($CO_2$:MeOH 90:10, 2.5 mL min$^{-1}$, 40 °C) $t_R$ = 9.16 (major), 10.62 (minor) minutes. $[\alpha]_D^{25}$= -7.5° (c 0.77, $CHCl_3$).

(*S*)-6,6'-dichloro-[1,1'-biphenyl]-2,2'-diol (**3r**)

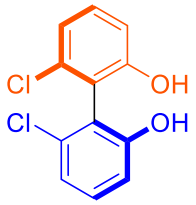


General procedure **B** was performed with 2-bromo-3-chlorophenol (20.7 mg, 0.1 mmol) and 2-hydroxy-6-chlorophenylboronic acid pinacol ester (76.4 mg, 0.3 mmol). The product was purified by column chromatography (0-20% EtOAc:hexane) to yield the product as a yellow crystalline solid (11.3 mg, 0.044 mmol, 44%, 97% *ee*).

**$^1$H NMR** (700 MHz, $CDCl_3$) δ 7.35 (t, *J* = 8.1 Hz, 2H), 7.18 (dd, *J* = 8.1, 1.0 Hz, 2H), 7.01 (dd, *J* = 8.2, 1.0 Hz, 2H), 4.98 (br s, 2H). **$^{13}$C NMR** (176 MHz, $CDCl_3$) δ 155.0, 135.5, 131.4, 122.0, 118.8, 114.7. **HRMS** $[M-H]^-$ calcd for $C_{12}H_7Cl_2O_2^-$; 252.9837, found 252.9829, Δ= 3.2 ppm. **Chiral SFC Analysis** (CHIRAL PAK

IG($CO_2$:MeOH 90:10, 2.5 mL min$^{-1}$, 40 °C) $t_R$ = 8.62 (major), 9.80 (minor) minutes. $[\alpha]_D^{25}$= -4.7° (c 0.74, $CHCl_3$).

**X-Ray Structure of (*S*)-6,6'-dichloro-[1,1'-biphenyl]-2,2'-diol**

Crystals of the purified sample were grown *via* slow evaporation from chloroform, which were analysed by x-ray diffraction. The absolute configuration was determined to be (*S*). The structure was deposited in the Cambridge Crystallographic Data Centre (deposition no. CCDC 2171202). The absolute stereochemistry of the other atropisomeric products in the scope have been assigned in analogy to this compound.

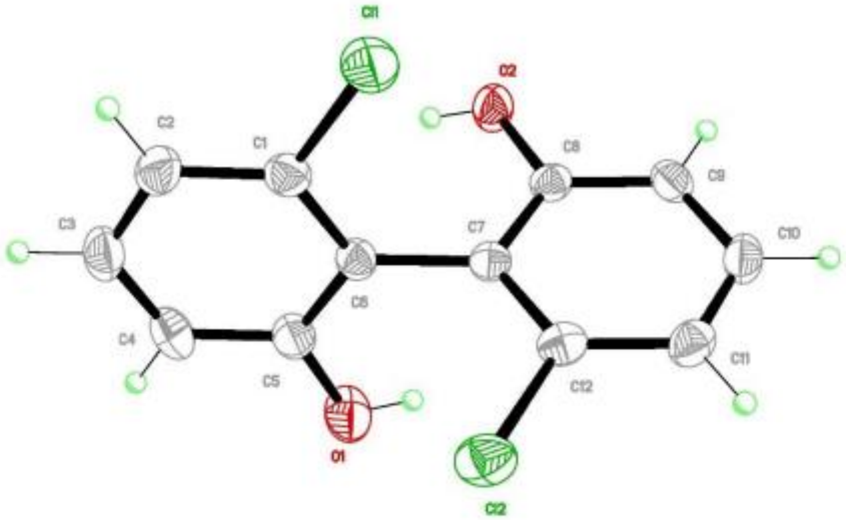


(*S*)-6,6'-dichloro-[1,1':3',1''-terphenyl]-2,2'-diol (**3s**)

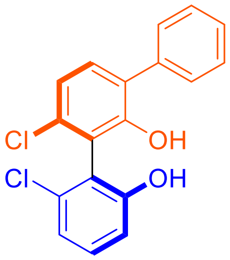


General procedure **B** was performed with 3-bromo-4-chloro-[1,1'-biphenyl]-2-ol (28.4 mg, 0.1 mmol), 2-hydroxy-6-chlorophenylboronic acid pinacol ester (50.9 mg, 0.2 mmol), $Pd_2dba_3$ (2.3 mg, 0.0025 mmol), (*R*)-sSPhos (3.1 mg, 0.006 mmol), KF (17.4 mg, 0.3 mmol) and $MgSO_4$ (24.1 mg, 0.2 mmol) in PhMe at 80 °C for 16 h. The product was purified by column chromatography (0-20% EtOAc:hexane) to yield the product as a yellow oil (16.3 mg, 0.049 mmol, 49%, 94% *ee*).

**$^1$H NMR** (400 MHz, $CDCl_3$) δ 7.57 – 7.48 (m, 4H), 7.46 – 7.34 (m, 2H), 7.33 (t, *J* = 8.2 Hz, 1H), 7.27 (t, *J* = 8.2 Hz, 1H), 7.17 (dd, *J* = 8.0, 1.0 Hz, 1H), 7.01 (dd, *J* = 8.1, 1.0 Hz, 1H), 5.30 (s, 1H), 4.93 (s, 1H). **$^{13}$C NMR** (101 MHz, $CDCl_3$) δ 154.6, 151.7, 136.1, 135.1, 134.8, 132.0, 131.0, 129.12, 129.10, 128.2, 127.6, 122.1, 122.0, 120.0, 119.3, 114.5 **HRMS** [M-H]$^-$ calcd for $C_{18}H_{11}Cl_2O_2$; 329.0160, found 329.0142, Δ=

5.69 ppm. **Chiral SFC Analysis** (CHIRAL ART SJ($CO_2$:MeOH 80:20, 2.5 mL min$^{-1}$, 40 °C) $t_R$ = 9.84 (major), 12.00 (minor) minutes. $[\alpha]_D^{25}$ = -17.0° (c 1.40, $CHCl_3$).

(*S*)-6,6'-dichloro-3-methyl-[1,1'-biphenyl]-2,2'-diol (**3t**)

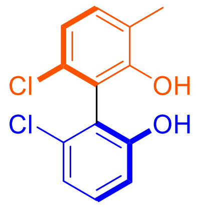


General procedure **B** was performed with 2-bromo-3-chloro-6-methylphenol (22.1 mg, 0.1 mmol) and 2-hydroxy-6-chlorophenylboronic acid pinacol ester (76.4 mg, 0.3 mmol). The product was purified by column chromatography (0-20% EtOAc:hexane) to yield the product as a yellow oil (8.3 mg, 0.031 mmol, 31%, 88% *ee*).

**$^1$H NMR** (500 MHz, $CDCl_3$) δ 7.36 (t, *J* = 8.0 Hz, 1 H), 7.22 (dd, *J* = 8.1, 0.9 Hz, 1H), 7.18 (dd, *J* = 8.0, 1.0 Hz, 1 H), 7.08 (d, *J* = 8.1 Hz, 1 H), 7.02 (dd, *J* = 8.2, 1.1 Hz, 1H), 4.88 (br s, 2H), 2.31 (s, 3H). **$^{13}$C NMR** (126 MHz, $CDCl_3$) δ 155.0, 152.9, 135.5, 132.7, 132.2, 131.5, 123.9, 122.1, 121.5, 118.8, 117.6, 114.7, 15.9. **HRMS** [M-H]$^-$ calcd for $C_{13}H_9Cl_2O_2$; 266.9985, found 266.9997, Δ= 4.40 ppm. **Chiral SFC Analysis** (CHIRAL PAK IG($CO_2$:MeOH 90:10, 2.5 mL min$^{-1}$, 40 °C) $t_R$ = 7.31 (major), 8.65 (minor) minutes. $[\alpha]_{25}^{D}$ = -6.39° (c 1.01, $CHCl_3$).

(*S*)-6-chloro-6'-(trifluoromethoxy)-[1,1'-biphenyl]-2,2'-diol (**3u**)

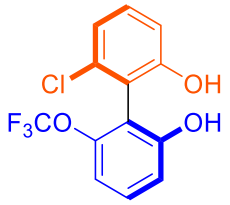


General procedure **B** was performed with 2-bromo-3-chlorophenol (20.7 mg, 0.1 mmol) and 2-hydroxy-6-(trifluoromethoxy)phenylboronic acid pinacol ester (91.2 mg, 0.3 mmol). The product was purified by column chromatography (0-20% EtOAc:hexane) to yield the product as a yellow solid (21 mg, 0.069 mmol, 69%, 96% *ee*).

**$^1$H NMR** (500 MHz, $CDCl_3$) δ 7.43 (t, *J* = 8.2 Hz, 1H), 7.33 (t, *J* = 8.2 Hz, 1H), 7.16 (d, *J* = 8.0 Hz, 1H), 7.03 (d, *J* = 8.3, 2H), 6.98 (d, *J* = 8.3, 1H), 3.90 (br s, 2H). **$^{13}$C NMR** (101 MHz, $CDCl_3$) δ 155.0, 154.9, 148.0, 135.6, 131.4, 131.3, 122.1, 114.9 (q, *J* = 272.3 Hz), 114.6, 114.5, 114.4, 112.7, 112.6. **$^{19}$F NMR** (471 MHz, $CDCl_3$) δ -58.37. **HRMS** [M-H]$^-$ calcd for $C_{13}H_7ClF_3O_3^-$; 303.0041, found 303.0036, Δ= 1.7 ppm. **Chiral SFC Analysis** (CHIRAL PAK IG($CO_2$:MeOH 95:5, 2.5 mL min$^{-1}$, 40 °C) $t_R$ = 6.44 (major), 8.89 (minor) minutes. $[\alpha]_D^{25}$= +7.7° (c 1.66, $CHCl_3$).

(*R*)-6-methyl-6'-(trifluoromethoxy)-[1,1'-biphenyl]-2,2'-diol (**3v**)

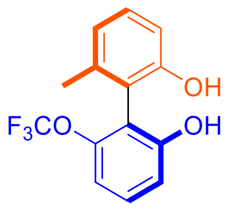


General procedure **B** was performed with 2-bromo-3-methylphenol (18.7 mg, 0.1 mmol) and 2-hydroxy-6-(trifluoromethoxy)phenylboronic acid pinacol ester (91.2 mg, 0.3 mmol). The product was purified by column chromatography (0-20% EtOAc:hexane) to yield the product as a white solid (25.3 mg, 0.089 mmol, 89%, 94% *ee*).

**$^1$H NMR** (500 MHz, $CDCl_3$) δ 7.39 (t, *J* = 8.2 Hz, 1H), 7.28 (t, *J* = 7.4 Hz, 1H), 7.04 – 6.99 (m, 2H), 6.94 (d, *J* = 7.4 Hz, 1H), 6.88 (d, *J* = 8.1 Hz, 1H), 5.00 (br s, 1H), 4.66 (br s, 1H), 2.05 (s, 3H). **$^{13}$C NMR** (126 MHz, $CDCl_3$) δ 154.9, 153.9, 147.9 (q, *J* = 1.5 Hz), 139.7, 130.69, 130.67, 122.9, 120.3 (d, *J* =257.4 Hz), 115.6, 114.8, 114.2, 113.5, 112.7 (q, *J* = 1.6 Hz), 19.5.**$^{19}$F NMR** (376 MHz, $CDCl_3$) δ -57.34. **HRMS** $[M-H]^-$ calcd for $C_{14}H_{10}F_3O_3^-$; 283.0588, found 283.0581, Δ = 2.5 ppm. **Chiral SFC Analysis** (CHIRAL ART SJ($CO_2$:MeOH 96:4, 2.5 mL min$^{-1}$, 40 °C) $t_R$ = 6.15 (major), 6.76 (minor) minutes. $[\alpha]_D^{25}$ = -3.8° (c 1.19, $CHCl_3$).

Ethyl (*R*,*E*)-3-(2'-fluoro-6,6'-dihydroxy-[1,1'-biphenyl]-2-yl)acrylate (**3w**)

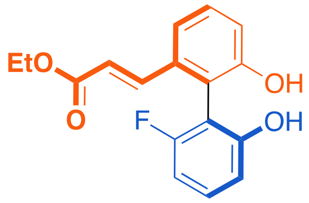


General procedure A was performed with ethyl (*E*)-3-(2-bromo-3-hydroxyphenyl)acrylate (27 mg, 0.1 mmol) and 2-hydroxy-6-fluorophenylboronic acid pinacol ester (48 mg, 0.20 mmol). The product was purified by column chromatography (10-40% EtOAc:petrol) to yield the product as a colourless solid (23.0 mg, 0.076 mmol, 76%, 64% ee).

**$^1$H NMR** (400 MHz, $CDCl_3$) δ 7.42 – 7.27 (m, 4H), 7.07 (dd, *J* = 6.0, 3.2 Hz, 1H), 6.85 – 6.75 (m, 2H), 6.35 (d, *J* = 15.9 Hz, 1H), 5.57 (s, 1H), 5.20 (s, 1H), 4.16 (q, *J* = 7.1 Hz, 2H), 1.26 (t, *J* = 7.1 Hz, 3H). **$^{13}$C NMR** (101 MHz, $CDCl_3$) δ 167.2, 160.8 (d, *J* = 246.7 Hz), 155.1 (d, *J* = 5.6 Hz), 154.4, 142.2, 135.9, 131.5 (d, *J* = 10.3 Hz), 130.7, 120.4, 119.4, 117.7, 117.2, 112.2 (d, *J* = 3.1 Hz), 108.3 (d, *J* = 20.3 Hz), 108.2 (d, *J* = 22.1 Hz), 60.9, 14.3. **$^{19}$F NMR** (376 MHz, $CDCl_3$) δ -112.11. **HRMS** $[M-H]^-$ calcd for $[C_{17}H_{14}FO_4]^-$; 301.0882, found 301.0887, Δ = 1.9 ppm. **Chiral-SFC** (ChiralART SB ($CO_2$:MeOH 85:15, 2.5 mLmin$^{-1}$, 40 °C) $t_R$ = 4.71(major), 5.69 (minor) minutes. $[\alpha]_D^{25}$ = -20.6° (c. 0.76, $CHCl_3$).

6,6''-difluoro-4',6'-dichloro-[1,1':3',1''-terphenyl]-2,2',2''-triol (5a + 5b)

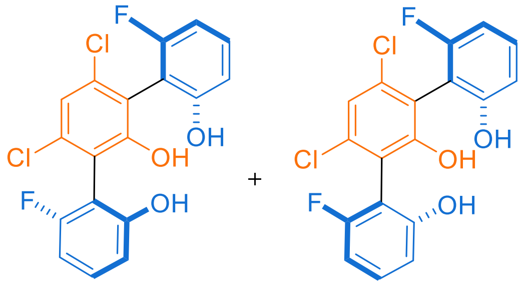

A 4 mL microwave vial was charged with 2,6-dibromo-3,5-dichlorophenol (32.1 mg, 0.10 mmol), 3-fluoro-2-(4,4,5,5-tetramethyl-1,3,2-dioxaborolan-2-yl)phenol (190.5 mg, 0.80 mmol), $K_3PO_4$ (63.6 mg, 0.30 mmol), (*R*)-Na.sSPhos (6.12 mg, 12mol%) and $Pd(OAc)_2$ (2.24 mg, 10 mol%). The vial was sealed and placed under an atmosphere of nitrogen via three evacuation-refill cycles, at which point toluene (0.33 mL) and deionised water (0.17 mL) were added. The reaction mixture was stirred at 40 °C for 16 h and then the solvent was removed under a stream of air. The mixture was partitioned in saturated aqueous $NH_4Cl$ solution (1 mL) and EtOAc (1 mL). The organic layer was isolated and the aqueous layer was extracted with further EtOAc (2 x 0.5 mL). The combined organic layers were dried ($MgSO_4$) and the solvent was evaporated under reduced pressure. The crude material was purified via flash column chromatography using a 10-50% EtOAc in petrol solvent system to isolate the crude *syn* (mixed with pinacol and unreacted boronate/boronic acid) and *anti* diastereomers (mixed with homocoupled product). The crude *syn* and *anti* diastereomers were further purified by flash column chromatography using a 10-35% acetone in petrol solvent system to afford the two desired products. If necessary, flash column chromatography was repeated under an acetone/petrol solvent system to isolate the pure product.

*Note – the isolated yields obtained were low in part due to the very challenging purification that was necessary in order to get analytically clean samples of each, which resulted in significant material loss.*

*Syn-Diastereomer (**5a**)*:

Isolated as a colourless oil (7.4 mg, 19.3 µmol, 19%)

**$^1$H NMR** (400 MHz, MeOD) δ 7.22 (td, J = 8.3, 6.6 Hz, 2H), 7.17 (s, 1H), 6.72 (d, J = 8.3 Hz, 2H), 6.65 (td, J = 8.3, 1.0 Hz, 2H). **$^{13}$C NMR** (101 MHz, MeOD) δ 162.5 (d, J = 244.0 Hz), 158.2 (d, J = 6.2 Hz), 158.1, 136.8, 131.0 19 (d, J = 10.6 Hz), 121.3, 119.9, 112.8 (d, J = 2.1 Hz), 112.1 (d, J = 19.3 Hz), 107.2 (d, J = 22.8 Hz). **$^{19}$F NMR** (376 MHz, MeOD) δ -114.4

*Anti-Diastereomer (**5b**)*:

Isolated as a colourless oil (6.6 mg, 17.2 µmol, 17%, 92% ee)

**$^1$H NMR** (400 MHz, MeOD) δ 7.28 – 7.19 (m, 2H), 7.19 (s, 1H), 6.72 (d, *J* = 8.0 Hz, 2H), 6.64 (t, *J* = 8.7 Hz, 2H). **$^{13}$C NMR** (101 MHz, MeOD) δ 162.7 (d, J = 243.8 Hz), 158.2 (d, J = 6.9 Hz), 156.7, 136.5, 131.1 (d, J = 10.8 Hz), 121.6, 119.8, 112.3 (d, J = 2.9 Hz), 111.7 (d, J = 19.7 Hz), 107.1 (d, J = 22.7 Hz). **$^{19}$F NMR** (376 MHz, MeOD) δ -114.1. **HRMS** $[M-H]^-$ calculated for $[C_{18}H_9Cl_2F_2O_3]^-$ 380.9902, found 380.9899. **Chiral SFC Analysis:** CHIRAL PAK IE ($CO_2$:MeOH, 90:10, 2.5 mL $min^{-1}$, 40 °C) $t_R$ = 6.72 (major), 7.99 (minor) minutes. $[\alpha]_D^{25}$ = + 40.2° (c. 0.3, $CHCl_3$).

<u>6,6''-difluoro-4',6'-dimethyl-[1,1':3',1''-terphenyl]-2,2',2''-triol (**5c** +**5d**)</u>

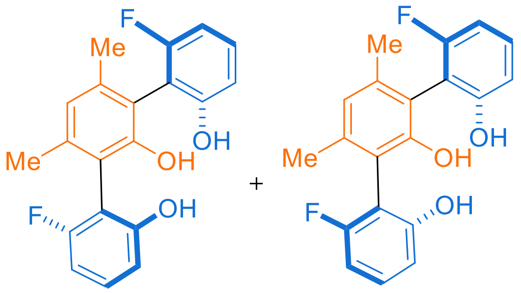


A 4 mL microwave vial was charged with 2,6-dibromo-3,5-dimethylphenol (28.0 mg, 0.10 mmol), 3-fluoro-2-(4,4,5,5-tetramethyl-1,3,2-dioxaborolan-2-yl)phenol (190.5 mg, 0.80 mmol), $K_3PO_4$ (63.6 mg, 0.30 mmol), (*R*)-Na.sSPhos (6.12 mg, 12mol%) and $Pd(OAc)_2$ (2.24 mg, 10mol%). The vial was sealed

and placed under an atmosphere of argon via three evacuation-refill cycles, at which point toluene (0.33 mL) and deionised water (0.17 mL) were added. The reaction mixture was stirred at 40 °C for 16 h and then the solvent was removed under a stream of air. The crude material was diluted in $CDCl_3$ containing a DME (0.03 mmol) internal standard and saturated aqueous $NH_4Cl$ solution (1 mL). The organic layer was isolated and the aqueous layer was extracted with further $CDCl_3$ (2 x 0.5 mL). The combined organic layers were passed through an $MgSO_4$ plug and a crude NMR was obtained, after which point the solvent was evaporated under reduced pressure and the crude material was purified via flash column chromatography using a 10-50% v/v EtOAc in petrol solvent system to isolate the pure *syn*-diastereomer. The *anti*-diastereomer was further purified by flash column chromatography using a 10-35% v/v acetone in petrol solvent system, to afford the two desired products.

*Syn-Diastereomer (**5c**):*

Isolated as a colourless oil (5.23 mg, 15.3 µmol, 15%).

**$^1$H NMR** (400 MHz, $CDCl_3$) δ 7.31 (m, 2H), 6.98 (s, 1H), 6.81 (m, 4H), 5.46 (s, 2H), 4.87 (s, 1H), 2.15 (s, 6H). **$^{13}$C NMR** (101 MHz, $CDCl_3$) δ 160.7 (d, J = 246.1 Hz), 154.7 (d, J = 5.9 Hz), 152.0, 140.6, 130.4 (d, J = 10.4 Hz), 124.8, 113.8, 111.6 (d, J = 3.1 Hz), 110.2 (d, J = 20.8 Hz), 108.0 (d, J = 22.5 Hz), 19.8. **$^{19}$F NMR** (376 MHz, $CDCl_3$) δ -112.64. **HRMS** $[M-H]^-$ calculated for $[C_{20}H_{15}F_2O_3]^-$ 341.0995, found 341.0989.

*Anti-Diastereomer (**5d**):*

Isolated as a colourless oil (15.3 mg, 44.8 µmol, 45%, 98% ee)

**$^1$H NMR** (400 MHz, $CDCl_3$) δ 7.37 – 7.29 (m, 2H), 7.01 (s, 1H), 6.88 (m, 2H), 6.80 (m, 2H), 5.16 (s, 2H), 4.91 (s, 1H), 2.16 (s, 6H).**$^{13}$C NMR** (101 MHz, $CDCl_3$) δ 160.5 (d, J = 245.5 Hz, $C_8$), 154.8 (d, J = 5.7 Hz, $C_{12}$), 152.1 ($C_2$), 141.1 ($C_3$), 130.5 (d, J = 10.4 Hz, $C_{10}$), 125.1 ($C_6$), 113.4 ($C_4$), 111.7 (d, J = 3.1 Hz, $C_{11}$), 109.9 (d, J = 20.5 Hz, $C_7$), 107.8 (d, J = 22.5 Hz, $C_9$), 19.7 ($C_5$).**$^{19}$F NMR** (471 MHz, $CDCl_3$) δ -113.24. **HRMS** $[M-H]^-$ calculated for $[C_{20}H_{15}F_2O_3]^-$ 341.0995, found 341.0990. **Chiral SFC Analysis** CHIRAL ART SJ ($CO_2$:MeOH, 90:10, 2.5 mL $min^{-1}$, 40 °C) $t_R$ = 6.77 (minor), 7.66 (major) minutes. $[\alpha]_D^{25}$ = + 55.2° (c. 0.5, $CHCl_3$).

## Unsuccessful Substrates

*All the below reactions evaluated under the following conditions:*
2.0 equiv. **2a**
5 mol% $Pd(OAc)_2$, 6 mol% (*R*)-sSPhos
3 eq. $Na_3PO_4$, $PhMe:H_2O$ (19:1)
40 °C, 16 h

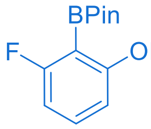

**2a**

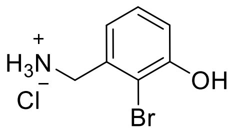

*No product detected*

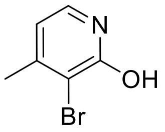

*No product detected, starting material degradation*

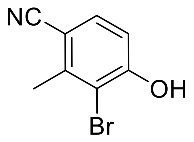

*No product detected*

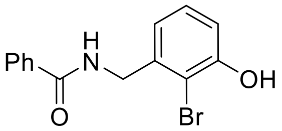

*No product detected*

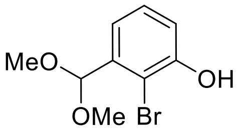

*Complex mixture*

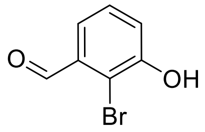

*Complex mixture*

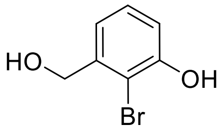

*No product detected*

*Reduced ee obtained with vinyl substituent:*

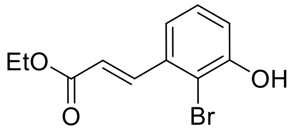

+

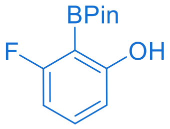

**2a**

5 mol% $Pd(OAc)_2$,
6 mol% (*R*)-sSPhos

3 eq. $Na_3PO_4$,
$PhMe:H_2O$ (19:1)
40 °C, 16 h

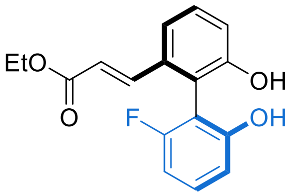

**3w**, 76%
64% ee

## Experiments to probe whether a starting phenolic aryl bromide is deprotonated under reaction conditions

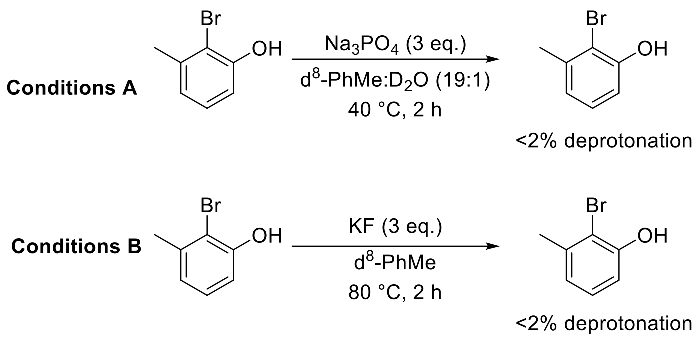


The follow procedure was followed to investigate whether the phenol was deprotonated under the reaction conditions, both A and B. Since there was a chance that the phenolate (if formed) would be insoluble, an internal standard (1,3,5-trimethoxybenzene) was included to establish if there was any mass loss in solution to try to account for this possibility.

**Conditions A:** 3-methyl-2-bromophenol (37.4 mg, 0.2 mmol) and 1,3,5-trimethoxybenzene (33.6 mg, 0.2 mmol) were dissolved in $d^8$-PhMe:$D_2O$ (19:1, 1 mL), and a $^1H$ NMR run to get the baseline ratio of the two components and observe chemical shifts. The solution was then placed in a microwave vial, and $Na_3PO_4$ (98.4 mg, 0.6 mmol) was added, then sealed and stirred at 40 °C for 2 h. The vial was cooled to rt and the mixture filtered through cotton wool, washed with $d^8$-PhMe, then a second $^1H$ NMR taken to determine the ratio of phenol to internal standard after treatment with base. No change in chemical shifts was observed.

**Conditions B:** 3-methyl-2-bromophenol (37.4 mg, 0.2 mmol) and 1,3,5-trimethoxybenzene (33.6 mg, 0.2 mmol) were dissolved in $d^8$-PhMe (1 mL), and a $^1H$ NMR run to get the baseline ratio of the two components. The solution was then placed in a microwave vial, and KF (34.9 mg, 0.6 mmol) was added, then sealed and stirred at 80 °C for 2 h. The vial was cooled to rt and the mixture filtered through cotton wool, washed with $d^8$-PhMe, then a second $^1H$ NMR taken to determine the ratio of phenol to internal standard after treatment with base. No change in chemical shifts was observed.

**Results:**

| Reaction | SM:TMB starting ratio | SM:TMB ratio 2 h | % remaining |
|---|---|---|---|
| **A** | 0.9502:1 | 0.9290:1 | **97.8%** |
| **B** | 0.9543:1 | 0.9498:1 | **99.5%** |

The lack of change in chemical shifts together with the lack of evidence for mass loss from solution suggest that there is not significant deprotonation of the phenolic starting material. However, this does not rule out the possibility of deprotonation occurring at a later point in the mechanism.

## Evaluation of a biphenol that is not tetra-ortho substituted

6-methyl-[1,1'-biphenyl]-2,2'-diol

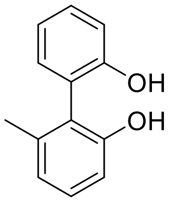

General procedure A was performed with 3-methyl-2-bromophenol (18.7 mg, 0.1 mmol), 2-hydroxyphenylboronic acid (20.7 mg, 0.15 mmol), $Pd(OAc)_2$ (1.1 mg, 0.005 mmol), (±)-sSPhos (5.1 mg, 0.01 mmol) and $Na_3PO_4$ (49 mg, 0.3 mmol) in THF:$H_2O$ (19:1) at 40 °C. The product was purified by column chromatography (0-20% EtOAc:petrol) to yield the product as a colourless residue (13.3 mg, 0.066 mmol, 66%). **$^1$H NMR** (400 MHz, $CDCl_3$) δ 7.36 (ddd, *J* = 8.2, 7.3, 1.8 Hz, 1H), 7.23 (t, *J* = 7.9 Hz, 1H), 7.14 (dd, *J* = 7.5, 1.8 Hz, 1H), 7.10 – 7.02 (m, 2H), 6.94 – 6.85 (m, 2H), 4.86 (br s, 2H), 2.07 (s, 3H). **$^{13}$C NMR** (101 MHz, $CDCl_3$) δ 153.9, 153.7, 139.0, 130.9, 130.6, 129.9, 122.5, 121.5, 121.0, 120.3, 116.2, 113.2, 19.9. Full separation of enantiomers was not observed on any chiral columns tested, with significant bridging between the peaks. Partial separation was observed with CHIRALART SC, 94:6 $CO_2$:MeOH, 2.5 mL/min, 4.62 min, 5.04 min; although significant bridging between the peaks was seen, suggesting interconversion of the isomers at the SFC analysis temperature of 40 $^o$C.

Rerunning the above reaction using **(*S*)-sSPhos** indicated an ee of 0% by analysis of the crude reaction mixture.

Data in accordance with literature: Hu, Z.; Liu, G. *Adv. Synth. Catal.* **2017**, *359*, 1643-1648.

## Evaluation of an *ortho*-methyl substituted boronate ester

We have evaluated an *ortho*-methyl substituted boronate ester with an *ortho*-methyl substituted aryl bromide in an attempt to form the C2 symmetric product shown below. This gave only traces (<5%) of product, as determined by comparison of the crude NMR with literature data. We presume that this coupling is too sterically challenging for the present ligand system.

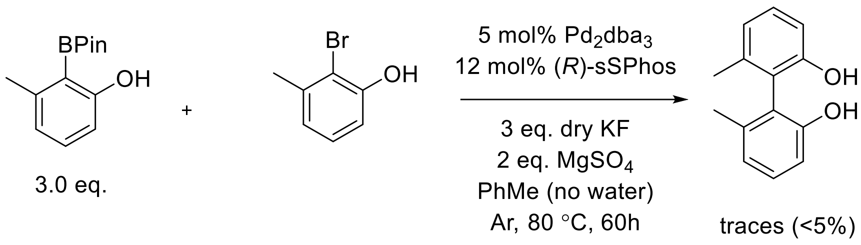


We have evaluated the same boronate ester with a less hindered aryl bromide (ortho-fluoro) and this proceeds with a higher yield, but it is still relatively low when compared to synthesis of the same product with the reversed coupling partners. The enantioselectivity is very similar.

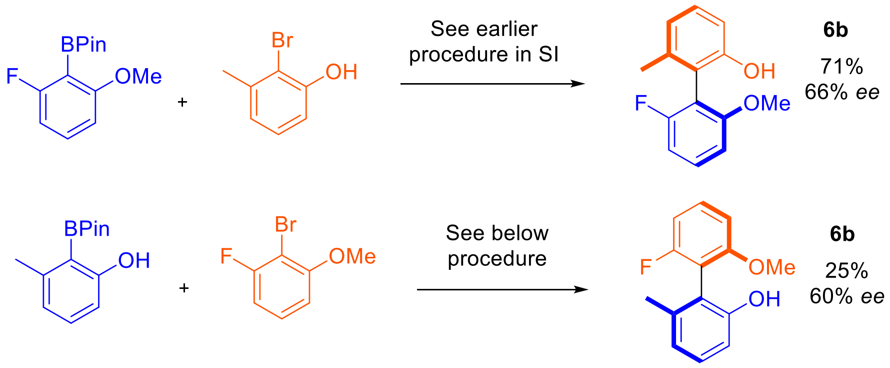


General procedure **A** was performed with 2-bromo-1-fluoro-3-methoxybenzene (20.4 mg, 0.1 mmol) and hydroxy-6-methylphenylboronic acid pinacol ester (70.2 mg, 0.3 mmol), $Pd(OAc)_2$ (1.1 mg, 0.005 mmol), (*R*)-sSPhos (5.1 mg, 0.01 mmol) and $Na_3PO_4$ (49 mg, 0.3 mmol) in PhMe:$H_2O$ (19:1) at 40 °C. The product was purified by column chromatography (0-15% EtOAc:petrol) to yield the product as a colourless oil (5.8 mg, 0.025 mmol, 25%, 60% *ee*). NMR data matched previously isolated scope entry **6b**. Sample *ee* was determined by chiral-SFC (CHIRAL ART SJ, 94:6 $CO_2$:MeOH, 2.5 mL/min, 6.09 min, 6.84 min).

## Preliminary Investigation of Post-Functionalization of an Axially Positioned Chloride

We have carried out a preliminary investigation of cross coupling of product **3e** at the chlorine located in the axial position to evaluate whether enantioenrichment can be maintained. Compound 3e was obtained in a scaled-up version of the reaction shown in the substrate scope and in this case the ee in **3e** was higher than that obtained on the smaller scale reaction, at 97% ee. This was submitted to a standard Suzuki coupling with SPhos as ligand at 80 °C but no conversion was obtained. We anticipated that this could be due to the two phenolic hydroxyls and so methylated these to give compound **15.** This was subjected to similar Suzuki conditions but at a higher temperature of 105 °C and a longer time of 36h. This resulted in a low yield of compound **16** which was able to be isolated and characterized. The ee was found to have partially degraded, from 97% to 56%. We anticipate that this is a result of the very challenging coupling requiring an elevated temperature. However, we imagine that an exhaustive survey of ligands and conditions would be likely to identify a combination that would be able to couple at lower temperature without loss of ee. It is also worth to note that compound 3e is probably quite a challenging compound to couple without ee loss in this manner since fluorine is a particularly small substituent at the axial position. It seems likely that a larger substituent here may result in less racemization.

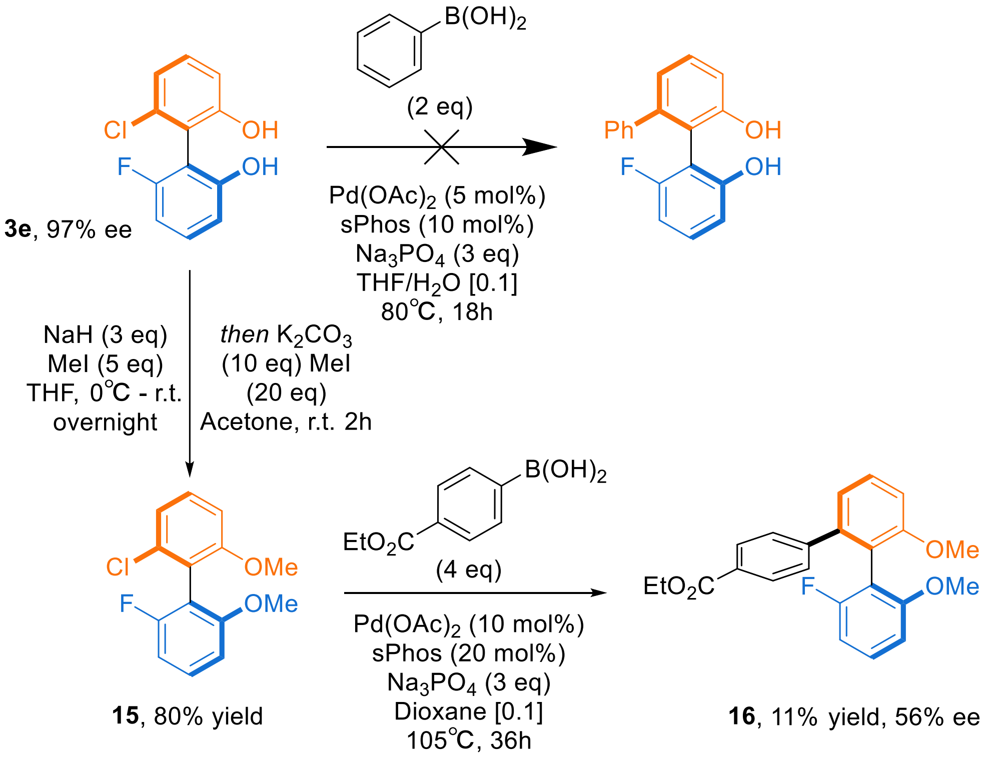


<u>(*S*)-2-chloro-2'-fluoro-6,6'-dimethoxy-1,1'-biphenyl (**15**)</u>

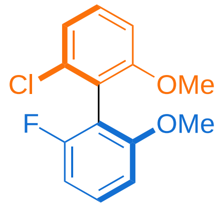

($S$)-6-chloro-6'-fluoro-[1,1'-biphenyl]-2,2'-diol (23 mg, 0.096 mmol) was dissolved in anhydrous THF (1 mL) and cooled to 0 °C. Sodium hydride (20 mg, 0.50 mmol) was added in one portion and the mixture stirred for 1 h under $N_2$ atmosphere. Iodomethane (31 µL, 0.50 mmol) was added dropwise and the mixture stirred overnight to room temperature. The reaction was quenched with the addition of a saturated aqueous solution of $NH_4Cl$ and the mixture extracted three times with ethyl acetate. The organic extracts were combined, dried with $MgSO_4$, filtered and the solvent removed under reduced pressure. The crude product was purified via flash column chromatography (10 – 20 – 30% ethyl acetate:petroleum ether) to give the title compound as a colourless solid (9 mg, 0.033 mmol, 34% yield) and recovered starting material (13 mg, 0.054 mmol).

The recovered starting material (12 mg, 0.05 mmol) was dissolved in anhydrous acetone (1 mL), to which anhydrous potassium carbonated (69 mg, 0.50 mmol) was added, followed by a dropwise addition of iodomethane (62 µL, 1.0 mmol) under $N_2$. The reaction mixture was stirred for 2 h and quenched with the addition of a saturated aqueous solution of $NH_4Cl$ and the mixture extracted three times with ethyl acetate. The organic extracts were combined, dried with $MgSO_4$, filtered and the solvent removed under reduced pressure. The crude product was purified via flash column chromatography (10 – 20 – 30% ethyl acetate:petroleum ether) to give the title compound as a colourless solid (7.5 mg, 0.028 mmol, 56% yield).

**$^1$H NMR** (400 MHz, $CDCl_3$) δ 7.38 – 7.27 (m, 1H), 7.11 (d, $J$ = 8.1 Hz, 0H), 6.89 (d, $J$ = 8.3 Hz, 0H), 6.83 – 6.76 (m, 1H), 3.78 (s, 1H), 3.75 (s, 1H). **$^{13}$C NMR** (101 MHz, $CDCl_3$) δ 160.7 (d, $J$ = 245.0 Hz), 158.7, 158.6 (d, $J$ = 7.4 Hz), 135.7, 129.9, 129.7, 121.7, 120.8, 112.9 (d, $J$ = 19.9 Hz), 109.5, 108.0 (d, $J$ = 22.6 Hz), 106.8 (d, $J$ = 2.8 Hz), 56.4, 56.3. **$^{19}$F NMR** (376 MHz, $CDCl_3$) δ -113.18.

Ethyl (R)-2''-fluoro-3',6''-dimethoxy-[1,1':2',1''-terphenyl]-4-carboxylate (16)

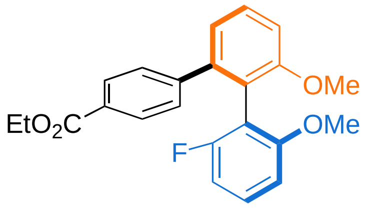


(4-(ethoxycarbonyl)phenyl)boronic acid (39 mg, 0.20 mmol), $Pd(OAc)_2$ (1.1 mg, 0.005 mmol), sPhos (dicyclohexyl(2',6'-dimethoxy-[1,1'-biphenyl]-2-yl)phosphane, 4.1 mg, 0.010 mmol) and $Na_3PO_4$ (25 mg, 0.30 mmol) were added to a microwave vial. The vial was sealed with a septum and the mixture underwent three vacuum/$N_2$ cycles followed by the addition of ($S$)-2-chloro-2'-fluoro-6,6'-dimethoxy-1,1'-biphenyl (13 mg, 0.05 mmol) in anhydrous 1,4-dioxane (0.5 mL). The reaction mixture was stirred at 105 °C for 36 h then diluted with a saturated aqueous solution of $NH_4Cl$ and the mixture extracted three times with ethyl acetate. The organic extracts were combined, dried with $MgSO_4$, filtered and the solvent removed under reduced pressure. The crude product was purified via flash column chromatography (0 – 10 – 20% ethyl acetate:petroleum ether) to give the title compound as a colourless solid (2 mg, 0.0053 mmol, 11% yield, 56% ee).

**$^1$H NMR** (400 MHz, $CDCl_3$) δ 7.83 (d, $J$ = 8.1 Hz, 2H), 7.44 (t, $J$ = 8.0 Hz, 1H), 7.17 (dd, $J$ = 10.7, 7.5 Hz, 3H), 7.03 (dd, $J$ = 8.0, 2.8 Hz, 2H), 6.63 (t, $J$ = 8.5 Hz, 1H), 6.50 (d, $J$ = 8.2 Hz, 1H), 4.33 (q, $J$ = 7.1 Hz, 2H), 3.80 (s, 3H), 3.47 (s, 3H), 1.36 (t, $J$ = 7.1 Hz, 3H).

$^{13}$**C NMR** (126 MHz, $CDCl_3$) δ 166.7, 160.8 (d, *J* = 243.8 Hz), 158.0 (d, *J* = 7.5 Hz), 157.6, 146.3, 143.2, 129.3, 129.2, 128.7, 128.6, 128.5, 121.8, 119.5, 113.9 (d, *J* = 19.8 Hz), 110.5, 107.6 (d, *J* = 23.1 Hz), 106.3 (d, *J* = 2.8 Hz), 60.9, 56.1, 55.7, 14.3. $^{19}$**F NMR** (376 MHz, $CDCl_3$) δ -113.75. **HRMS** $[M]^+$ calcd for $[C_{23}H_{21}FO_4]^+$; 380.1424, found 380.1420, Δ = 0.4 ppm. **Chiral-SFC** (CHIRAL PAK IG (CO2:MeOH 96:4, 2.5 mL min-1, 40 °C) tR= 7.35 (minor), 8.65 (major) minutes. $[\alpha]_D^{25}$ = -16.8° (c. 0.08, $CHCl_3$).

## Control Reactions to Vary Phenolic Hydroxyls (Scheme 2A)

(*R*)-6-fluoro-2'-methoxy-6'-methyl-[1,1'-biphenyl]-2-ol (**6a**)

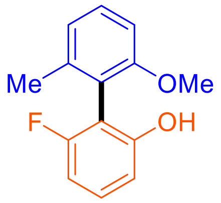


**6a**, 67% yield
85% *ee*

General procedure **A** was performed with 3-methyl-2-bromoanisole (20.1 mg, 0.1 mmol), 2-hydroxy-6-fluorophenylboronic acid pinacol ester (48 mg, 0.2 mmol), $Pd(OAc)_2$ (1.1 mg, 0.005 mmol), (*R*)-sSPhos (3.1 mg, 0.006 mmol) and $Na_3PO_4$ (49 mg, 0.3 mmol) in PhMe:$H_2O$ (19:1) at 40 °C. The product was purified by column chromatography (0-10% EtOAc:petrol) to yield the product as a colourless oil (15.6 mg, 0.067 mmol, 67%, 85% *ee*).

**$^1$H NMR** (500 MHz, $CDCl_3$) δ 7.36 (t, *J* = 8.0 Hz, 1H), 7.27 (td, *J* = 8.3, 6.5 Hz, 1H), 7.00 (d, *J* = 7.6 Hz, 1H), 6.90 (d, *J* = 8.3 Hz, 1H), 6.85 (dt, *J* = 8.3, 1.0 Hz, 1H), 6.77 (ddd, *J* = 9.0, 8.3, 1.1 Hz, 1H), 4.94 (s, 1H), 3.78 (s, 3H), 2.14 (s, 3H). **$^{13}$C NMR** (126 MHz, $CDCl_3$) δ 160.3 (d, *J* = 244.4 Hz), 157.7, 154.3 (d, *J* = 6.3 Hz), 140.4, 130.1, 129.4 (d, *J* = 10.4 Hz), 123.0, 117.6, 112.2 (d, *J* = 20.7 Hz), 111.1 (d, *J* = 3.0 Hz), 108.7, 107.4 (d, *J* = 22.7 Hz), 55.9, 19.7. **$^{19}$F NMR** (471 MHz, $CDCl_3$) δ -114.40. **HRMS** $[M-H]^-$ calcd for $C_{14}H_{12}FO_2^-$ ; 231.0827, found 231.0832, Δ= 2.3 ppm. **Chiral SFC Analysis** (CHIRAL PAK IG, 98:2 $CO_2$:MeOH, 2.5 mL/min, 5.91 min, 6.61 min). $[\alpha]_D^{25}$ = +32.8° (c 0.94, $CHCl_3$).

(*R*)-2'-fluoro-6'-methoxy-6-methyl-[1,1'-biphenyl]-2-ol (**6b**)

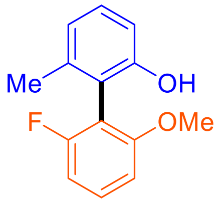


**6b**, 71% yield
66% *ee*

General procedure A was performed with 3-methyl-2-bromophenol (18.7 mg, 0.1 mmol), 2-methoxy-6-fluorophenylboronic acid pinacol ester (50.4 mg, 0.2 mmol), $Pd(OAc)_2$ (1.1 mg, 0.005 mmol), (*R*)-sSPhos (5.1 mg, 0.01 mmol) and $Na_3PO_4$ (49 mg, 0.3 mmol) in PhMe:$H_2O$ (19:1) at 40 °C. The product was purified by column chromatography (0-15% EtOAc:petrol) to yield the product as a colourless oil (16.4 mg, 0.071 mmol, 71%, 66% *ee*).

**$^1$H NMR** (400 MHz, $CDCl_3$) δ 7.39 (td, *J* = 8.4, 6.6 Hz, 1H), 7.21 (t, *J* = 7.8 Hz, 1H), 6.88 (d, *J* = 8.3 Hz, 1H), 6.87 – 6.80 (m, 3H), 4.68 (s, 1H), 3.79 (s, 3H), 2.06 (s, 3H). **$^{13}$C NMR** (101 MHz, $CDCl_3$) δ 160.91 (d, *J* = 245.6 Hz), 158.4 (d, *J* = 7.1 Hz), 153.3, 138.8, 130.4 (d, *J* = 10.5 Hz), 129.2, 122.0, 118.0, 112.8,

111.7 (d, *J* = 20.2 Hz), 108.7 (d, *J* = 23.0 Hz), 106.9 (d, *J* = 3.0 Hz), 56.2, 19.9. **$^{19}$F NMR** (376 MHz, $CDCl_3$) δ -111.22. **HRMS** $[M]^+$ calcd for $C_{14}H_{13}FO_2^+$; 232.0898, found 232.0894, Δ= 1.7 ppm. **Chiral SFC Analysis** (CHIRAL ART SJ, 94:6 $CO_2$:MeOH, 2.5 mL/min, 6.09 min, 6.84 min). $[\alpha]_D^{25}$= +19.9° (c 1.05, $CHCl_3$).

(*R*)-2-fluoro-2',6-dimethoxy-6'-methyl-1,1'-biphenyl (**6c**)

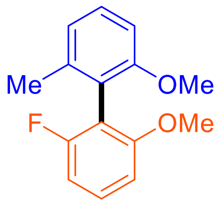


**6c**, 29% yield
<5% *ee*

General procedure A was performed with 3-methyl-2-bromoanisole (20.1 mg, 0.1 mmol), 2-methoxy-6-fluorophenylboronic acid pinacol ester (50.4 mg, 0.2 mmol), $Pd(OAc)_2$ (1.1 mg, 0.005 mmol), (*R*)-sSPhos (3.1 mg, 0.006 mmol) and $Na_3PO_4$ (49 mg, 0.3 mmol) in PhMe:$H_2O$ (19:1) at 40 °C. The product was purified by column chromatography (0-5% EtOAc:petrol) to yield the product as a clear residue (7.2 mg, 0.029 mmol, 29%, <5% *ee*).

**$^{1}$H NMR** (400 MHz, $CDCl_3$) δ 7.35 – 7.22 (m, 2H), 6.91 (d, *J* = 7.6 Hz, 1H), 6.83 (d, *J* = 8.3 Hz, 1H), 6.81 – 6.73 (m, 2H), 3.75 (s, 3H), 3.73 (s, 3H), 2.06 (s, 3H). **$^{13}$C NMR** (101 MHz, $CDCl_3$) δ 160.5 (d, *J* = 243.3 Hz), 158.3 (d, *J* = 7.8 Hz), 157.4, 139.0, 128.9 (d, *J* = 10.5 Hz), 128.7, 122.1, 120.6, 114.3 (d, *J* = 20.4 Hz), 108.3, 107.9 (d, *J* = 23.3 Hz), 106.5 (d, *J* = 2.8 Hz), 56.1, 55.9, 19.7. **$^{19}$F NMR** (471 MHz, $CDCl_3$) δ -114.31. **HRMS** $[M+H]^+$ calcd for $C_{15}H_{16}FO_2^+$; 247.1129, found 247.1130, Δ= 0.4 ppm. **Chiral SFC Analysis** (CHIRAL ART SJ, 99:1 $CO_2$:MeOH, 2.5 mL/min, 3.77 min, 4.03 min).

# Control Experiment Using Alkylated Ligand (Scheme 2B)

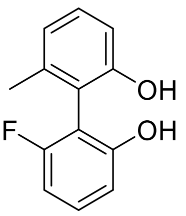


**3a**

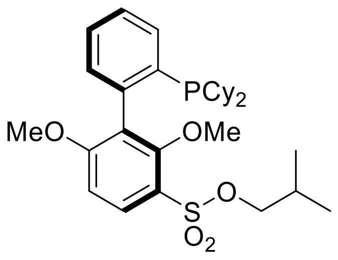


(*S*)-**13**

| **Using (*S*)-13** | **Using (*R*)-sSPhos** |
| --- | --- |
| 66% | 73% |
| -8% *ee* | 91% *ee* |

**Reaction using (*S*)-13:** General procedure **A** was performed with 3-methyl-2-bromophenol (18.7 mg, 0.1 mmol) and 2-hydroxy-6-fluorophenylboronic acid pinacol ester (47 mg, 0.2 mmol), $Pd(OAc)_2$ (1.1 mg, 0.005 mmol), (*S*)-**13** (3.3 mg, 0.006 mmol) and $Na_3PO_4$ (49 mg, 0.3 mmol) in PhMe:$H_2O$ (19:1) at 40 °C. The crude was purified by column chromatography to yield the product as a white solid (14.4 mg, 0.066 mmol, 66%, -8% *ee*). Sample *ee* determined by chiral-SFC (CHIRAL ART SJ, 90:10 $CO_2$:MeOH, 2.5 mL/min, 7.23 min [major], 8.05 min [minor]). NMR data corresponds to previously isolated samples ($^1H$, $^{13}C$, $^{19}F$).

## Enantioselective formation of a 2-amino, 2'-hydroxybiphenyl

*N*-(2-bromo-3-methylphenyl)acetamide

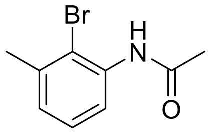


2-Bromo-3-methylaniline (0.626 mL, 5 mmol) and acetic anhydride (0.71 mL, 7.5 mmol, 1.5 eq.) were added to $CH_2Cl_2$ (20 mL) and the resulting solution cooled to 0 °C. Triethylamine (1.04 mL, 7.5 mmol, 1.5 eq.) was added dropwise to the solution and the reaction was allowed to warm to rt over 4 h. The reaction was deemed incomplete by LCMS analysis, so $Ac_2O$ (0.71 mL,7.5 mmol, 1.5 eq.) was added and the reaction stirred at rt for a further 2 h. Water (20 mL) was added, the organic layer separated, dried ($MgSO_4$) and solvent removed under reduced pressure to yield crude product. The crude product was purified by flash silica chromatography (30-100% EtOAc:petrol) to yield the title compound as a white solid (1.05 g, 4.58 mmol, 92%).

**$^1$H NMR** (400 MHz, $CDCl_3$) δ 8.18 (d, *J* = 8.1 Hz, 1H), 7.74 (br s, 1H), 7.23 (t, *J* = 7.9 Hz, 1H), 7.03 (d, *J* = 7.4 Hz, 1H), 2.45 (s, 3H), 2.26 (s, 3H). **$^{13}$C NMR** (101 MHz, $CDCl_3$) δ 168.2, 138.4, 135.8, 127.4, 126.3, 119.7, 116.3, 24.9, 23.8.

Data in accordance with literature (*Synthesis*, **2020**, *52*, 917).

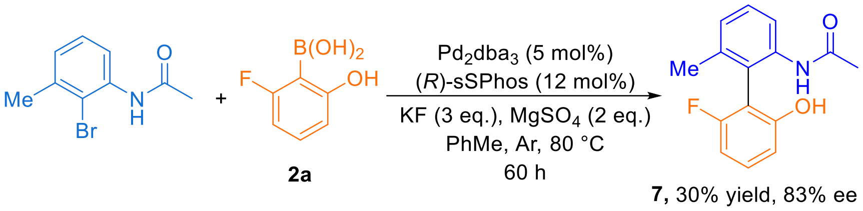


N-(2'-fluoro-6'-hydroxy-6-methyl-[1,1'-biphenyl]-2-yl)acetamide (**7**)

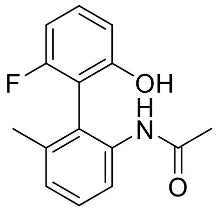


General procedure **B** was performed with *N*-(2-bromo-3-methylphenyl)acetamide (22.8 mg, 0.1 mmol) and 2-hydroxy-6-fluorophenylboronic acid pinacol ester (71 mg, 0.3 mmol). The product was purified by column chromatography (0-50%EtOAc:hexane) to yield the product as a white solid (7.9 mg, 0.030 mmol, 30%, 83% *ee*).

**$^1$H NMR** (400 MHz, $CDCl_3$) δ 7.89 (d, *J* = 8.2 Hz, 1H), 7.39 (t, *J* = 8.2 Hz, 1H), 7.33 (m, 1 H), 7.21 (d, *J* = 7.7 Hz, 1H), 6.93 – 6.85 (d and br s, 2H), 6.80 (t, *J* = 8.4 Hz, 1H), 5.59 (br s, 1H), 2.10 (s, 3H), 1.98 (s, 3H). **$^{13}$C NMR** (126 MHz, $CDCl_3$) δ 169.3, 159.9 (d, *J* = 245.0 Hz), 154.8 (d, *J* = 5.7 Hz), 139.2, 136.5,

130. 5 (d, *J* = 10.5 Hz), 129.8, 127.4, 122.0, 121.2, 112.4, 111.6 (d, *J* = 19.8 Hz), 107.8 (d, *J* = 21.6 Hz), 24.2, 20.0. **$^{19}$F NMR** (376 MHz, $CDCl_3$) δ -113.74. **HRMS** $[M-H]^-$ calcd for $[C_{15}H_{13}FNO_2]^-$; 258.0936, found 258.0940, Δ = 1.6 ppm. **Chiral-SFC** (ChiralPAK IE ($CO_2$:MeOH 95:05, 2.5 mLmin$^{-1}$, 40 °C) $t_R$ = 10.85 (major), 11.98 (minor) minutes. $[\alpha]_D^{25}$ = -10.7° (c. 0.45, $CHCl_3$).

## sSPhos Applied to Desymmetrising Suzuki-Miyaura Coupling (Scheme 2D)

**Substrate synthesis:**

<u>bis(3-chloro-4-methylphenyl)methanaminium chloride</u>

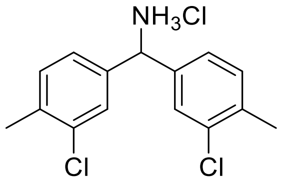


Magnesium turnings (270 mg, 11 mmol) were suspended in THF (5 mL), and 2-chloro-4-bromotoluene (1.47 mL, 11 mmol) was added dropwise, until the reaction flask warmed up. The remaining 2-chloro-4-bromotoluene was diluted in THF (3 mL) and added dropwise to the flask. The resulting solution was heated at reflux for 1 h, after which time it was cooled to rt and 3-chloro-4-methylbenzonitrile (dissolved in THF (5 mL), 1.52 g, 10 mmol) was added dropwise. Once addition was complete, the solution was heated at reflux for 16 h. The solution was then cooled in ice, and methanol (10 mL) added. After 10 min of stirring, $NaBH_4$ (800 mg, 20 mmol) was added portionwise and the solution stirred at room temperature for 1 h, then at reflux for 2 h. The reaction was then partially concentrated to a thick oil, and poured into HCl solution (3 M, 50 mL), instantly forming a yellow precipitate. The mixture was filtered, and the residue washed with $H_2O$ (2 x 40 mL) and $Et_2O$ (3 x 40 mL) until the residue was a white solid. This solid was taken and dried under high vacuum to yield the product as a white solid (1.48 g, 4.6 mmol, 46%).

**$^1$H NMR** (400 MHz, MeOD) δ 7.42 (d, *J* = 2.0 Hz, 2H), 7.39 (d, *J* = 7.9 Hz, 2H), 7.24 (dd, *J* = 8.0, 2.0 Hz, 2H), 5.62 (s, 1H), 2.38 (s, 6H). **$^{13}$C NMR** (101 MHz, MeOD) δ 137.0, 136.1, 134.7, 131.5, 127.5, 125.4, 56.2, 18.3. **HRMS** $[M-NH_3]^+$ calcd for $C_{15}H_{13}Cl_2^+$; 263.0389, found 263.0396, Δ= 2.7 ppm.

<u>*N*-(bis(3-chloro-4-methylphenyl)methyl)-1,1,1-trifluoromethanesulfonamide (**8**)</u>

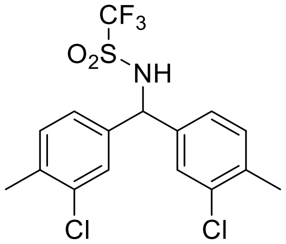


Bis(3-chloro-4-methylphenyl)methanaminium chloride (1.27 g, 4 mmol) was suspended in $CH_2Cl_2$ (50 mL), and cooled to -78 °C. Triflic anhydride (0.7 mL, 4.2 mmol) was added slowly, then triethylamine (1.1 mL, 8 mmol) added dropwise. The mixture was stirred at -78 °C for 2 h, then water (50 mL) was added and allowed to warm to rt. The organic layer was then separated, dried ($MgSO_4$), and solvent removed. The residue was purified by column chromatography to give the product as a white solid (305 mg, 0.7 mmol, 18%).

**$^1$H NMR** (400 MHz, $CDCl_3$) δ 7.24 (d, *J* = 7.9 Hz, 2H), 7.19 (d, *J* = 1.9 Hz, 2H), 7.04 (dd, *J* = 7.9, 2.0 Hz, 2H), 5.75 (d, *J* = 5.4 Hz, 1H), 5.54 (s, 1H), 2.38 (s, 6H). **$^{13}$C NMR** (101 MHz, $CDCl_3$) δ 138.4, 136.6,

135.1, 131.5, 127.6, 125.2, 119.4 (q, *J* = 321.0 Hz), 61.0, 19.8. **$^{19}$F NMR** (376 MHz, $CDCl_3$) δ -77.10. **HRMS** $[M-H]^-$ calcd for $C_{16}H_{13}Cl_2F_3NO_2S$; 410.0002, found 410.0002, Δ= 0 ppm.

**Desymmetrizing Suzuki Miyaura Coupling:**

ethyl 5'-((3-chloro-4-methylphenyl)((trifluoromethyl)sulfonamido)methyl)-2'-methyl-[1,1'-biphenyl]-4-carboxylate (**9**)

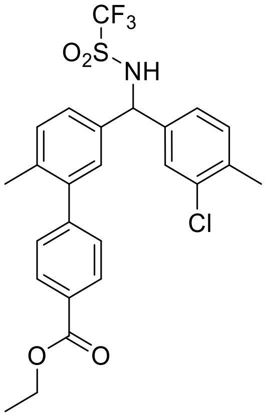


*N*-(bis(3-chloro-4-methylphenyl)methyl)-1,1,1-trifluoromethanesulfonamide (41.2 mg, 0.1 mmol), $Pd(OAc)_2$ (1.1 mg, 0.005 mmol), $K_3PO_4$ (63 mg, 0.3 mmol) and (*S*)-sSPhos (5.1 mg, 0.01 mmol) were placed in a 4 mL microwave vial and sealed. The vial was placed under a nitrogen atmosphere *via* 3 back-fill cycles then THF:$H_2O$ (19:1, 0.5 mL) added. The reaction was stirred at 40 °C for 16 h, after which the vial was opened, and solvent removed under a stream of air. $CDCl_3$ (0.5 mL, containing 3 mg of 1,2-dimethoxyethane as internal standard) and HCl solution (3 M, 2 mL) were added, and layers separated. The aqueous layer was extracted with $CDCl_3$ (1 mL), and the combined organic layers filtered through $MgSO_4$. The crude was purified by column chromatography (0-20% EtOAc:petrol) to yield the product as a white residue (26.7 mg, 0.051 mmol, 51%, 94% *ee*).

**$^1$H NMR** (400 MHz, $CDCl_3$) δ 8.05 (dt, *J* = 8.4, 1.8 Hz, 2H), 7.32 (dt, *J* = 8.4, 1.9 Hz, 2H), 7.29 (d, *J* = 8.0 Hz, 1H), 7.26 (s, 1H), 7.23 (d, *J* = 7.8 Hz, 1H), 7.15 (dd, *J* = 7.9, 2.1 Hz, 1H), 7.13 – 7.06 (m, 2H), 5.95 (d, *J* = 8.9 Hz, 1H), 5.82 (d, *J* = 8.8 Hz, 1H), 4.37 (q, *J* = 7.1 Hz, 2H), 2.36 (s, 3H), 2.25 (s, 3H), 1.39 (t, *J* = 7.1 Hz, 3H). **$^{13}$C NMR** (101 MHz, $CDCl_3$) δ 166.6, 145.8, 141.6, 139.0, 137.0, 136.3, 135.8, 134.9, 131.4, 131.2, 129.5, 129.2, 129.1, 128.1, 127.7, 126.5, 125.3, 119.4 (q, *J* = 321.0 Hz), 61.4, 61.2, 20.1, 19.7, 14.3. **$^{19}$F NMR** (376 MHz, $CDCl_3$) δ -77.12. **HRMS** $[M-H]^-$ calcd for $C_{25}H_{22}ClF_3NO_4S^-$; 524.0916, found 524.0919, Δ= 0.6 ppm. Sample *ee* was determined by chiral SFC (CHIRAL PAK IE, 92:08 $CO_2$:MeOH, 2.5 mL/min, 4.84 min [major], 5.47 min [minor]). $[\alpha]_D^{25}$ = -2.9° (93% *ee*, c 1.81, $CHCl_3$).

The absolute stereochemistry of this compound is presently unestablished as we have not been successful in growing X-ray quality crystals despite a number of attempts.

## Resolution of sSPhos by Preparative SFC

Racemic sSPhos was prepared according to literature procedures. (*19*–*21*)

<u>(*R*)-sSPhos **AND** (*S*)-sSPhos</u>

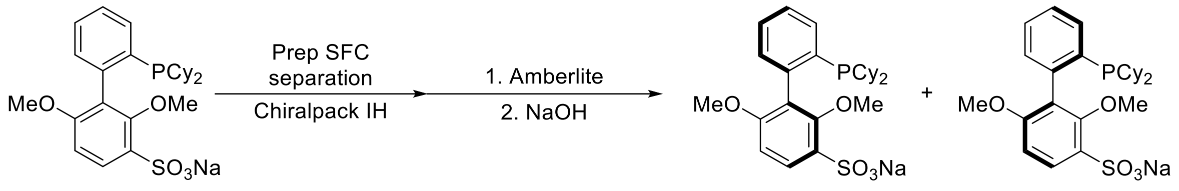


sSPhos was separated at AstraZeneca in three batches (3 x 5g) by preparatory SFC. sSPhos (5 g) was dissolved in $CH_2Cl_2$ (15 mL) and MeOH (20 mL), then purified by preparative SFC (Column: CHIRAL PAK IH, 20 x 250 mm, 5 micron, Mobile phase: 30 % [MeOH + 0.1% $NH_3$]: 70% $CO_2$, Flow rate: 60 mL/min, Back pressure regulator (BPR): 120 bar, Column temperature: 40 °C). Two fractions were isolated: an earlier fraction which was subsequently determined to be (*R*)-sSPhos (2.0 - 4.2 minutes) and a later fraction that was subsequently determined to be (*S*)-sSPhos (5.5 - 8.2 minutes). The solvent was removed to yield off-white solids. The three batches of (*R*)-sSPhos were dissolved in MeOH (100 mL) and passed through a column of Amberlite IRC120 H, hydrogen form (washed first with MeOH until run clear) five times, then solvent removed to yield the protonated zwitterionic (*R*)-sSPhos. This residue was dissolved in $CH_2Cl_2$ (500 mL) and cooled in ice. NaOH (2.5 M, 200 mL) and $H_2O$ (300 mL) was added, and the layers separated. The aqueous layer was washed with $CH_2Cl_2$ (400 mL), the organic layers combined, dried ($MgSO_4$) and solvent removed (keeping the flask below 25 °C). The residue was redissolved in MeOH (50 mL), sonicated, then filtered through a pad of celite. The celite was washed with ice-cold MeOH (2 x 5 mL), and solvent removed under reduced pressure(<25 °C). $Et_2O$ (2 x 100 mL) was added then removed (<25 °C), then hexane (2 x 100 mL) was added then removed (<25 °C) to yield (*R*)-sSPhos as a white solid (6.07 g, 11.9 mmol, 40%, 99.3% ee Sample *ee* was determined by chiral SFC analysis following derivatisation to the neopentyl sulfonate ester (see p S51): YMC SC, 82:18 $CO_2$:MeOH, 2.5 mL/min, 7.83 min [minor], 9.61 min [minor]. NMR data corresponded to the racemic compound. $[\alpha]_D^{25}$= -46.5° (c 0.43, MeOH).

The identification assignment of absolute stereochemistry to the two fractions is detailed on page S45 by conversion to an intermediate for which an X-ray crystal structure had been obtained.

When the same process was repeated with the (*S*)-sSPhos batch, a white solid with $[\alpha]_D^{25}$= +45.2° (c 0.46, MeOH) was obtained.

## Resolution of sSPhos using BINOL Auxiliary Approach

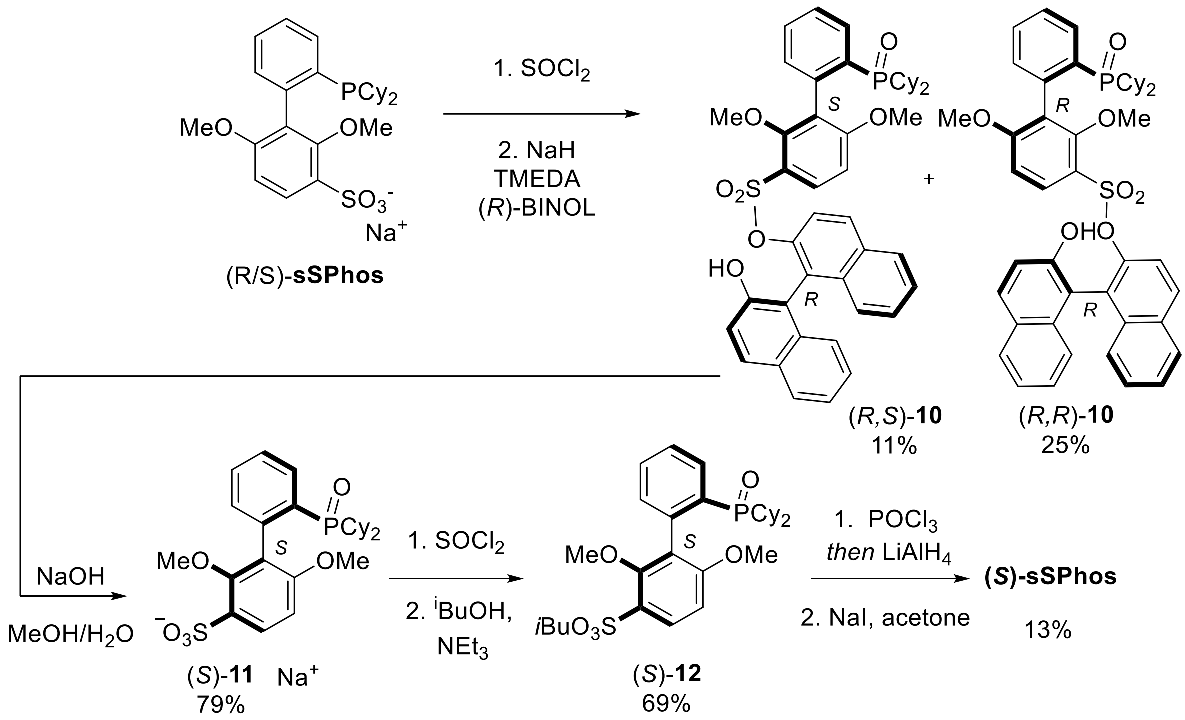

(*R*)-2'-hydroxy-[1,1'-binaphthalen]-2-yl (*R*)-2'-(dicyclohexylphosphoryl)-2,6-dimethoxy-[1,1'-biphenyl]-3-sulfonate (*R,R*)-**10 and** (*R*)-2'-hydroxy-[1,1'-binaphthalen]-2-yl (*S*)-2'-(dicyclohexylphosphoryl)-2,6-dimethoxy-[1,1'-biphenyl]-3-sulfonate (*R,S*)-**10**

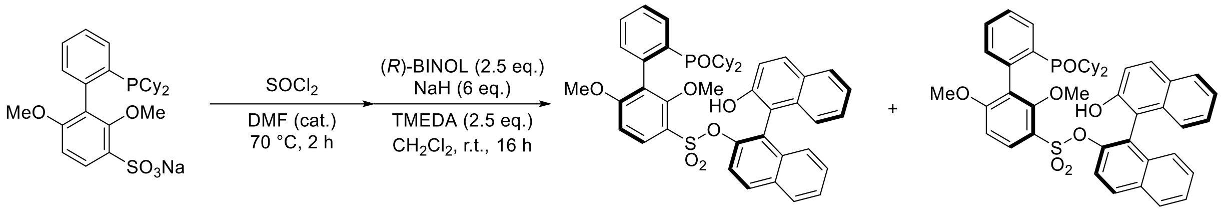


(*R*,*R*)-**10**
25%

(*R*,*S*)-**10**
11%

(±)-sSPhos (10.2 g, 20 mmol) was dissolved in thionyl chloride (40 mL) under nitrogen. DMF (1 mL) was added, and the solution stirred at 70 °C for 2 h. The solvent was then removed under a stream of air, then under high vacuum for 1 h. The residue was cooled to 0 °C, and EtOAc (50 mL) and $H_2O$ (50 mL) were used to wash the residue into water (200 mL). EtOAc (200 mL) was added, and the organic layer separated. The aqueous layer was washed with EtOAc (250 mL), the organic layers combined, dried ($MgSO_4$) and solvent removed under reduced pressure. (*R*)-BINOL (14.3 g, 50 mmol) was dissolved in $CH_2Cl_2$ (250 mL) and sodium hydride (60% w/w in mineral oil, 4.8 g, 24 mmol) was added portionwise. The mixture was stirred for 15 min, then the crude sulfonyl chloride (dissolved in 50 mL $CH_2Cl_2$) was added slowly, followed by TMEDA (7.5 mL, 50 mmol). The reaction mixture was stirred at 40 °C for 16 h, after which time the solvent was removed under reduced pressure. EtOAc (100 mL) and $H_2O$ (100 mL) was added, the layers separated and the aqueous layer washed with EtOAc (2 x 100 mL). The aqueous layers were combined, dried ($MgSO_4$) and solvent removed under reduced pressure to yield the crude material. The crude was dissolved in a minimum amount of $CH_2Cl_2$, then purified by column chromatography (20-100% EtOAc:petrol) to yield (*R*,*R*)-**10** (3.8 g, 4.9 mmol, 25%) as an off-white solid, and (*R*,*S*)-**10** (4.6 g) with impurities. The (*R*,*S*) diastereomer was purified by adding EtOAc (90 mL), sonicating and then filtering. The filter cake was washed with boiling EtOAc (450 mL) to yield pure product (735 mg). The filtrate was allowed to cool and crystalise over 64 h and filtered to yield further product (510 mg). The filtrate was concentrated to about 20% of the volume (100 mL), hexane (500 mL) added, then allowed to stand for 4 h. The mixture was filtered to yield further product (457 mg). In total, (*R*,*S*)-**10** was isolated as a white crystalline solid (1.7 g, 2.20 mmol, 11%).

(*R*,*R*)-**10**: Off-white solid. **$^1$H NMR** (400 MHz, $CDCl_3$) δ 7.95 (d, *J* = 9.0 Hz, 1H), 7.91 – 7.85 (m, 2H), 7.80 (dd, *J* = 8.0, 1.3 Hz, 1H), 7.70 (d, *J* = 8.9 Hz, 1H), 7.48 (ddd, *J* = 7.6, 5.3, 2.7 Hz, 1H), 7.43 – 7.36 (m, 3H), 7.35 – 7.27 (m, 2H), 7.26 – 7.17 (m, 3H), 7.06 – 6.99 (m, 2H), 6.83 (d, *J* = 9.0 Hz, 1H), 6.37 (d, *J* = 9.0 Hz, 1H), 3.77 (s, 3H), 3.02 (s, 3H), 2.09 – 0.94 (m, 22H). **$^{13}$C NMR** (101 MHz, $CDCl_3$) δ 161.6, 157.3, 153.9, 146.4, 138.5 (d, *J* = 3.6 Hz), 134.2, 134.1 (d, *J* = 9.2 Hz), 132.1, 130.9, 130.7 (d, *J* = 2.6 Hz), 130.1 (d, *J* = 12.1 Hz), 130.0 (d, *J* = 84.3 Hz), 129.1 (d, *J* = 1.1 Hz), 128.0, 127.7, 127.5, 127.1, 127.0 (d, *J* = 11.7 Hz), 126.6, 126.2, 125.6, 124.9, 124.7 (d, *J* = 2.7 Hz), 122.4, 122.3 (d, *J* = 1.7 Hz), 119.7, 113.9, 105.8, 61.4, 55.7, 37.8 (d, *J* = 107.7 Hz), 37.2 (d, *J* = 107.5 Hz), 27.0 (d, *J* = 12.4 Hz), 26.7 (d, *J* = 5.8 Hz), 26.5 (d, *J* = 5.7 Hz), 26.3 (d, *J* = 12.7 Hz), 26.2 (d, *J* = 3.3 Hz), 25.9, 25.7, 25.6, 25.5 (d, *J* = 3.3 Hz), 25.3 (d, *J* = 3.0 Hz). **$^{31}$P NMR** (162 MHz, $CDCl_3$) δ 48.19. **HRMS** $[M+2Na]^{2+}$ calcd for $C_{46}H_{47}Na_2O_7PS^{2+}$; 410.1282, found 410.1288, Δ= 1.5 ppm. $[\alpha]_D^{25}$ = +33.7° (c 0.34, DMSO).

(*R*,*S*)-**10**: White crystalline solid. **$^1$H NMR** (400 MHz, $CDCl_3$) δ 8.16 (d, *J* = 9.1 Hz, 1H), 8.02 (d, *J* = 9.1 Hz, 1H), 7.93 (d, *J* = 8.2 Hz, 1H), 7.75 (d, *J* = 7.9 Hz, 1H), 7.73 (s, 1H), 7.58 – 7.48 (m, 2H), 7.47 – 7.38 (m, 4H), 7.30 – 7.19 (m, 2H), 7.20 – 7.11 (m, 3H), 6.89 (d, *J* = 8.4 Hz, 1H), 6.43 (d, *J* = 9.1 Hz, 1H), 3.64 (s, 3H), 3.19 (s, 3H), 2.13 – 1.03 (m, 22H). **$^{13}$C NMR** (101 MHz, $CDCl_3$) δ 161.9, 156.4, 152.9, 147.1, 137.5 (d, *J* = 4.4 Hz), 134.2, 134.1 (d, *J* = 9.2 Hz), 133.8, 132.2, 132.1, 131.0 (d, *J* = 10.6 Hz), 130.8 (d, *J* = 84.0 Hz), 130.6 (d, *J* = 2.6 Hz), 129.8, 129.1, 128.4, 128.0, 127.7, 127.2 (d, *J* = 11.1 Hz), 127.0, 126.5, 126.4,

126.1 (d, $J$ = 2.8 Hz), 126.1, 125.3, 124.7, 122.9, 121.9, 120.8, 119.0, 114.0, 105.5, 62.1, 55.6, 37.7 (d, $J$ = 169.5 Hz), 37.0 (d, $J$ = 170.7 Hz), 26.9 (d, $J$ = 12.5 Hz), 26.7 (d, $J$ = 4.4 Hz), 26.5 (d, $J$ = 5.9 Hz), 26.4 (d, $J$ = 10.0 Hz), 26.1 (d, $J$ = 2.2 Hz), 26.0 (d, $J$ = 12.5 Hz), 25.8 (d, $J$ = 3.0 Hz), 25.5 (d, $J$ = 3.5 Hz). **$^{31}$P NMR** (162 MHz, $CDCl_3$) δ 47.37. **HRMS** $[M+H]^+$ calcd for $C_{46}H_{48}O_7PS^+$; 775.2853, found 775.2853, Δ= 0 ppm. $[\boldsymbol{\alpha}]_{\boldsymbol{D}}^{\mathbf{25}}$= -82.4° (c 0.43, DMSO).

**X-Ray Structure of (*R*)-2'-hydroxy-[1,1'-binaphthalen]-2-yl (*R*)-2'-(dicyclohexylphosphoryl)-2,6-dimethoxy-[1,1'-biphenyl]-3-sulfonate,** (*R*,*R*)-**10**

Crystals of the purified sample of (*R*,*R*)-**10** were grown *via* slow evaporation from the column solvent system in the column fractions, which were analysed by x-ray diffraction. The configuration was determined to be (R,R). The structure was deposited in the Cambridge Crystallographic Data Centre (deposition no. CCDC 2170066).

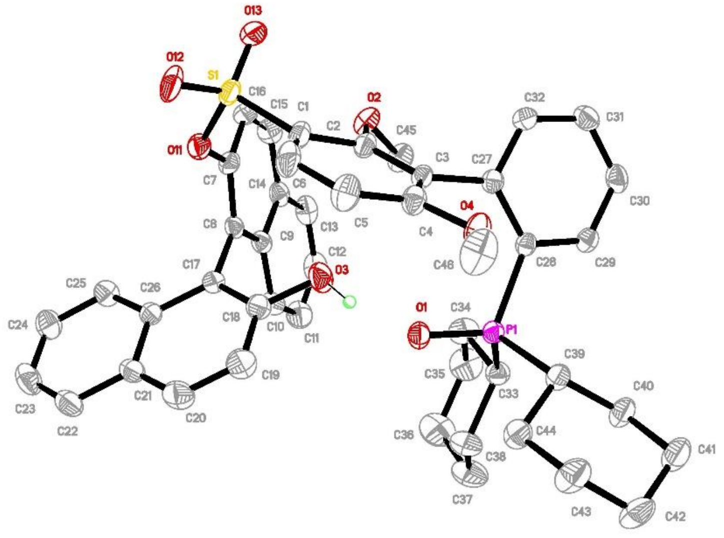


## sodium (*S*)-2'-(dicyclohexylphosphoryl)-2,6-dimethoxy-[1,1'-biphenyl]-3-sulfonate (*S*)-**11**

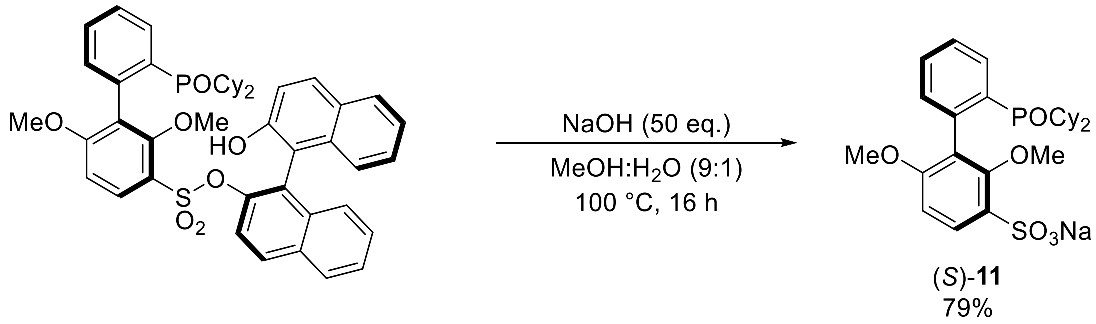


(*R*,*S*)-**10** (1.2 g, 1.55 mmol) and NaOH (3 g, 7.5 mmol) were dissolved in a $MeOH:H_2O$ mixture (9:1, 20 mL) and heated at 100 °C for 16 h. After the reaction was complete, the solution was partially concentrated, and HCl solution (3 M, 150 mL), $H_2O$ (500 mL) and $CH_2Cl_2$ (500 mL) added. The organic layer was taken, dried ($MgSO_4$) and solvent removed to yield crude product. This was purified by column chromatography (0-15% $MeOH:CH_2Cl_2$) to yield (*S*)-**11** as a black powder, (645 mg, 1.22 mmol, 79%).

**$^{1}$H NMR** (400 MHz, MeOD) δ 7.97 (d, *J* = 8.8 Hz, 1H), 7.87 (dd, *J* = 11.8, 7.6 Hz, 1H), 7.69 (t, *J* = 7.5 Hz, 1H), 7.60 (t, *J* = 7.5, 6.9 Hz, 1H), 7.42 (dd, *J* = 7.4, 4.0 Hz, 1H), 6.93 (d, *J* = 8.8 Hz, 1H), 3.75 (s, 3H), 3.43 (s, 3H), 2.37 – 2.19 (m, 1H), 2.10 – 1.52 (m, 11H), 1.50 – 1.05 (m, 8H). **$^{13}$C NMR** (101 MHz, MeOD) δ 159.5, 155.8, 138.0 (d, *J* = 6.5 Hz), 134.3 (d, *J* = 9.9 Hz), 132.2 (d, *J* = 10.4 Hz), 131.6 (d, *J* = 2.7 Hz), 130.7, 129.8, 127.3 (d, *J* = 11.7 Hz), 125.6 (d, *J* = 88.1 Hz), 124.5 (d, *J* = 2.5 Hz), 105.7, 60.6, 55.0, 36.3 (d, *J* = 65.2 Hz), 35.3 (d, *J* = 60.8 Hz), 26.2 (d, *J* = 2.8 Hz), 25.9 (d, *J* = 3.0 Hz), 25.8 (d, *J* = 2.9 Hz), 25.6, 25.5 (m), 25.3, 25.0 (d, *J* = 3.3 Hz), 24.8 (d, *J* = 3.7 Hz). **$^{31}$P NMR** (162 MHz, MeOD) δ 52.97. $[\alpha]_D^{25}$ = -0.45° (c 0.66, MeOH). **HRMS** $[M-H]^-$ calcd for $C_{26}H_{34}O_6PS^-$; 505.1819, found 505.1816, Δ= 0.6 ppm

isobutyl (*S*)-2'-(dicyclohexylphosphoryl)-2,6-dimethoxy-[1,1'-biphenyl]-3-sulfonate (*S*)-**12**

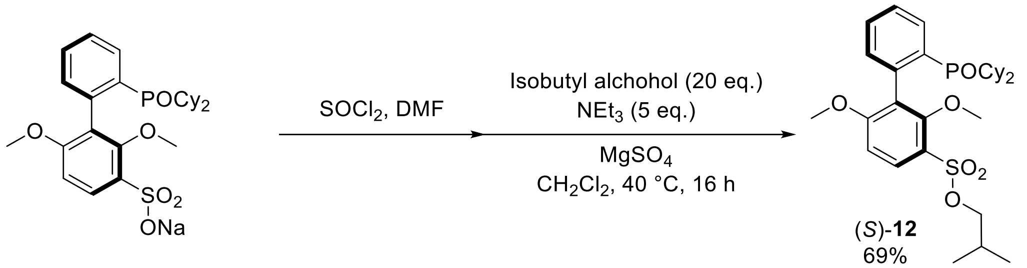


(*S*)-**11** (610 mg, 1.15 mmol) was dissolved in thionyl chloride (4 mL) and sealed into a microwave vial under nitrogen. DMF (0.1 mL) was added, and the solution stirred at 70 °C for 2 h. The solvent was then removed under a stream of air, then under high vacuum for 1 h. The residue was cooled to 0 °C, and EtOAc (10 mL) and $H_2O$ (10 mL) were used to wash the residue into water (40 mL). EtOAc (40 mL) was added, and the organic layer separated. The aqueous layer was washed with EtOAc (50 mL), the organic layers combined, dried ($MgSO_4$) and solvent removed under reduced pressure. The crude sulfonyl chloride was then dissolved in $CH_2Cl_2$ (10 mL) and cooled to 0 °C. $MgSO_4$ (1g) was added, followed by isobutyl alcohol (2.2 mL, 23 mmol), then triethylamine (0.8 mL, 5.75 mmol) was added dropwise. The reaction was heated to 40 °C and stirred for 16 h. Once complete, water (50 mL) was added, the layers separated, and the aqueous layer washed with $CH_2Cl_2$ (2 x 50 mL). The combined organic layers were washed with HCl (3M, 50 mL) and brine (saturated, 50 mL), dried ($MgSO_4$) and solvent removed to yield crude product. This material was purified by column chromatography (20-100% EtOAc:petrol) to yield (*S*)-**12** as a white solid (444 mg, 0.79 mmol, 69%). $[\alpha]_D^{25}$= -34.6° (c 1.15, $CHCl_3$).

**$^{1}$H NMR** (400 MHz, $CDCl_3$) δ 7.91 (d, *J* = 8.9 Hz, 1H), 7.60 – 7.51 (m, 1H), 7.50 – 7.42 (m, 2H), 7.38 – 7.28 (m, 1H), 6.76 (d, *J* = 8.9 Hz, 1H), 3.99 (dd, *J* = 9.1, 5.9 Hz, 1H), 3.88 (dd, *J* = 9.1, 7.5 Hz, 1H), 3.74 (s, 3H), 3.43 (s, 3H), 2.10 – 1.95 (m, 1H), 1.93 – 1.53 (m, 12H), 1.53 – 1.05 (m, 10H), 0.99 (d, *J* = 6.7 Hz, 3H), 0.95 (d, *J* = 6.7 Hz, 3H). **$^{13}$C NMR** (101 MHz, $CDCl_3$) δ 162.0, 157.5, 138.6, 133.4 (d, *J* = 8.8 Hz), 132.0, 131.1, 130.6 (d, *J* = 10.9 Hz), 130.2 (d, *J* = 2.5 Hz), 127.1 (d, *J* = 11.1 Hz), 126.3 (d, *J* = 2.6 Hz), 121.4, 105.4, 76.9, 61.7, 55.7, 38.4 (d, *J* = 66.1 Hz), 36.7 (d, *J* = 67.0 Hz), 28.2, 26.8 (d, *J* = 3.0 Hz), 26.7 (d, *J* = 3.1 Hz), 26.5 (d, *J* = 12.5 Hz), 26.2 (d, *J* = 2.9 Hz), 25.9 (d, *J* = 1.4 Hz), 25.8 (d, *J* = 1.3 Hz), 25.5 (d, *J* = 3.3 Hz), 25.3 (d, *J* = 3.2 Hz), 25.1 (d, *J* = 2.9 Hz), 19.1, 18.8. **$^{31}$P NMR** (162 MHz, $CDCl_3$) δ 46.12. **HRMS** $[M+H]^+$ calcd for $C_{30}H_{44}O_6PS$; 563.2591, found 563.2603, Δ= 2.1 ppm.

(*S*)-sSPhos **AND** isobutyl (*S*)-2'-(dicyclohexylphosphaneyl)-2,6-dimethoxy-[1,1'-biphenyl]-3-sulfonate (*S*)-**13**

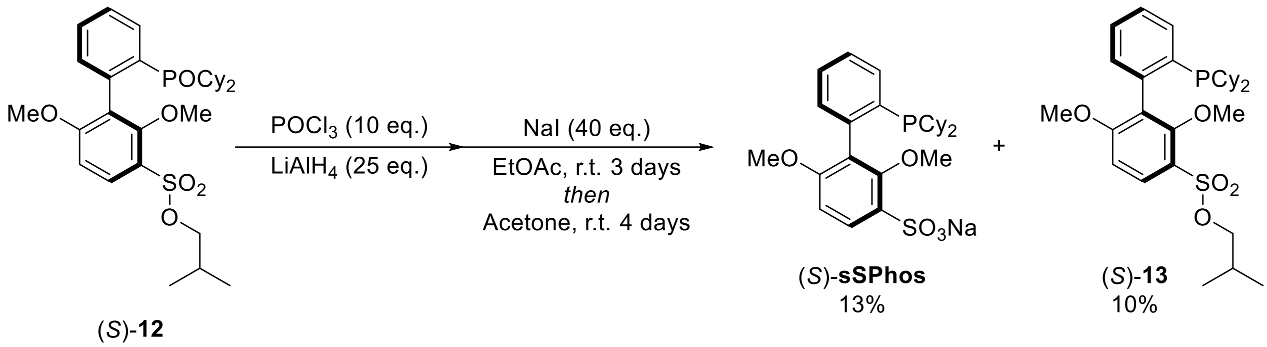


All solvents used (THF, 1,4-dioxane, EtOAc, acetone, MeOH) were sparged for 30 min before use. $LiAlH_4$ (pellet, ~500 mg) was placed under an argon atmosphere with a balloon and dissolved in THF to form a 2 M solution by stirring for 3 h. (*S*)-**12** (281 mg, 0.5 mmol) was placed under an argon atmosphere and dissolved in 1,4-dioxane (1.5 mL) and THF (6 mL). $POCl_3$ (0.47 mL, 5 mmol) was then added, and the solution stirred at 30 °C for 2 h. After this time, the reaction mixture was cooled in ice, and $LiAlH_4$ solution (2M, 6.25 mL, 12.5 mmol) was added dropwise and the solution was stirred for 2 h at rt. The reaction was observed to proceed to 85% conversion by LCMS. The reaction mixture was cooled in ice and EtOAc (10 mL) was slowly added, followed by MeOH (5 mL) and stirred for a further 10 min. After this time, the solvent was removed under a stream of nitrogen gas. $H_2O$ (200 mL) and $CHCl_3$ (200 mL) were then added and the organic layer taken, dried ($MgSO_4$) and solvent removed under reduced pressure. The reaction mixture was kept under 25 °C during removal of solvent to prevent re-oxidation. Sodium iodide (3 g, 40 eq.) was added to the residue, and the solids were placed under an argon atmosphere. EtOAc (10 mL) was added, and the reaction stirred for 64 h at rt. The reaction was analysed by LCMS and determined to have low conversion (<10% sSPhos), so the solvent was removed under a stream of nitrogen gas, more sodium iodide (1.5 g) added and placed under an argon atmosphere. Acetone (15 mL) was added and after 86 h, LCMS analysis showed 28% of **13 (P)**, 25% of **11 (P=O SO3)** and 46% of the desired **sSPhos**. The solvent was removed under a stream of nitrogen, and $H_2O$ (40 mL), HCl (3M, 5 mL) and EtOAc (50 mL) added. The layers were separated, and the aqueous layer was washed with EtOAc (5 x 50 mL) until only sSPhos was seen by LCMS in the aqueous layer. The organic layers were combined and purified to yield (*S*)-**13** (see below). The aqueous layer was cooled in ice, and NaOH (2.5 M, 15 mL) was added until the solution was alkaline, then $CH_2Cl_2$ (200 mL) added and the layers separated. The aqueous layer was washed with $CH_2Cl_2$ (200 mL), the organic layers combined, dried ($MgSO_4$) and solvent removed (<25 °C) to yield crude (*S*)-sSPhos. The residue was dissolved in ice-cold MeOH (1 mL), sonicated, and filtered through a celite pad. The celite was washed with ice-cold MeOH (2 x 0.25 mL), then the solvent removed under reduced pressure (<25 °C). $Et_2O$ (2 x 20 mL) was added and removed under reduced pressure (<25 °C), then hexane (2 x 20 mL) was added and removed under reduced pressure (<25 °C) until a white solid formed. The solid was dried for 4 h under high vacuum to yield (*S*)-sSPhos as a white solid (34 mg, 0.066 mmol, 13%). NMR data corresponded to the racemic compound. $[\alpha]_D^{25}$ = +43.4° (c 0.65, MeOH).

(*S*)-**13**: The combined EtOAc washes above were dried ($MgSO_4$) and solvent removed (<25 °C) to yield the crude mix of **13** and **11** (P=O $SO_3$). The crude mixture was purified by column chromatography (0-20 % EtOAc:petrol) to yield (*S*)-**13** as a white solid (27 mg, 0.049 mmol, 10%). **$^1$H NMR** (500 MHz, $CDCl_3$) δ 7.96 (d, *J* = 8.9 Hz, 1H), 7.62 (dt, *J* = 7.1, 2.1 Hz, 1H), 7.45 – 7.38 (m, 2H), 7.31 – 7.24 (m, 1H), 6.79 (d, *J* = 8.9 Hz, 1H), 3.92 (dd, *J* = 6.7, 1.6 Hz, 2H), 3.77 (s, 3H), 3.36 (s, 3H), 2.15 – 2.05 (m, 1H), 2.05 – 1.98 (m, 1H), 1.83 – 1.45 (m, 11H), 1.39 – 0.83 (m, 10H), 1.02 (d, *J* = 6.7 Hz, 3H), 0.97 (d, *J* = 6.8 Hz, 3H). **$^{13}$C NMR** (126 MHz, $CDCl_3$) δ 162.4, 157.1 (d, *J* = 1.5 Hz), 140.6 (d, *J* = 32.3 Hz), 136.6 (d, *J* = 20.6 Hz), 132.8 (d, *J* = 3.7 Hz), 131.7, 131.5 (d, *J* = 6.0 Hz), 128.1, 127.1, 127.0 (d, *J* = 7.1 Hz), 121.7, 105.4, 76.9 (d, *J* = 3.2 Hz), 61.2, 55.7, 35.4 (d, *J* = 15.2 Hz), 33.0 (d, *J* = 13.5 Hz), 30.2 (d, *J* = 19.1 Hz), 30.0 (d, *J* = 15.5 Hz), 29.3 (d, *J* = 12.4 Hz), 28.7 (d, *J* = 6.5 Hz), 28.2, 27.6 (d, *J* = 12.1 Hz), 27.5 (d, *J* = 6.5 Hz), 27.2 (d, *J* = 9.4

Hz), 27.1, 26.5 (d, *J* = 11.6 Hz), 19.1, 18.9. **$^{31}$P NMR** (203 MHz, $CDCl_3$) δ -7.85. **HRMS** $[M+Li]^+$ calcd for $C_{30}H_{43}O_5PSLi^+$; 553.2723, found 553.2741, Δ = 3.3 ppm. $[\alpha]_D^{25}$= -2.6° (c 0.49, $CHCl_3$).

## Determination of ligand stereochemistry

To determine ligand stereochemistry after prep chiral SFC, the earlier fraction of material obtained from the separation was derivatised to form the (*R*)-BINOL adduct of the corresponding phosphine oxie and the **$^{1}$H** and **$^{31}$P** NMR compared to the diastereomers formed in the linear ligand synthesis to determine whether (*R,R*)-**10** or (*R,S*)-**10** was obtained.

The following procedure was used:

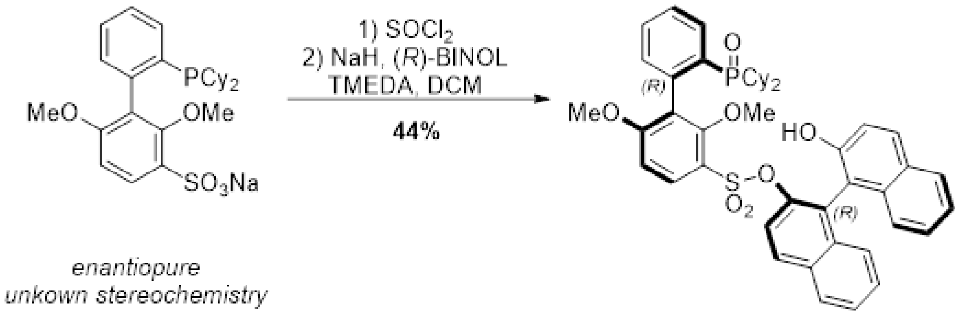


Enantioenriched sSPhos from the earlier fraction obtained by prep chiral SFC (25 mg, 0.050 mmol) was dissolved in thionyl chloride (0.5 mL) under nitrogen. DMF (1 drop) was added, and the solution stirred at 70 °C for 2 h. The solvent was then removed under a stream of air, then under high vacuum for 1 h. The residue was dissolved in EtOAc (5 mL) and $H_2O$ (5 mL) was added at 0 °C and the layers were isolated. The aqueous layer was washed with EtOAc (2 x 10 mL) and the organic layers were combined, dried over $MgSO_4$ and the solvent removed under reduced pressure. To a solution of sodium hydride (60% dispersion in mineral oil, 11.0 mg, 0.275 mmol) in DCM (0.5 mL) at 0 °C was added a solution of (*R*)-BINOL (72.0 mg, 0.251 mmol) in DCM (0.5 mL). The reaction mixture was allowed to stir for 30 mins at 0 °C, at which point a solution of the enantiopure sulfonyl chloride in DCM (0.5 mL) was added dropwise at 0 °C, followed by the dropwise addition of TMEDA (0.02 mL, 0.133 mmol). The reaction mixture was then heated to 45 °C and left to stir for 48 h, at which point the reaction was quenched with saturated aqueous ammonium chloride solution (5 mL) and extracted with DCM (3 x 5 mL). The combined organic extracts were dried over $MgSO_4$ and the solvent removed under reduced pressure. The crude material was purified via flash column chromatography using a 30% v/v EtOAc in pet. ether solvent system to afford the target compound as a single diastereomer (17.2 mg, 0.022 mmol, 44%).

The **$^{1}$H** and **$^{31}$P** NMR data matched with that of (*R,R*)**-10** (the diastereomer that afforded the crystal structure), indicating that the earlier eluting enantiomer of sSPhos obtained from the prep SFC conditions described on page S38 (and that used for the scope of the asymmetric Suzuki-Miyaura cross-coupling) was the (*R*)-enantiomer.

# Resolution of sSPhos via recrystallisation of diastereomeric salts with quinidine

**Starting Material**

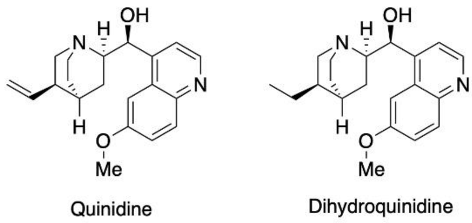


Quinidine purchased from Acros Organics (cat number 163680250), determined to contain 10% of dihydroquinidine, was used in the resolution of sSPhos via recrystallisation of diasteromeric salts.

**$^{1}$H NMR** (500 MHz, $CDCl_3$) Quinidine: δ 8.69 (d, *J* = 4.5 Hz, 1H), 7.99 (d, *J* = 9.2 Hz, 1H), 7.53 (d, *J* = 4.5 Hz, 1H), 7.33 (dd, *J* = 9.2, 2.7 Hz, 1H), 7.20 (d, *J* = 2.7 Hz, 1H), 5.99 (ddd, *J* = 16.9, 10.6, 7.5 Hz, 1H), 5.58 (d, *J* = 4.7 Hz, 1H), 5.04 (q, *J* = 1.5 Hz, 1H), 5.01 (dt, *J* = 9.3, 1.7 Hz, 1H), 3.89 (s, 3H), 3.24 (ddd, *J* = 13.7, 7.8, 2.3 Hz, 1H), 3.10 (td, *J* = 9.2, 4.5 Hz, 1H), 2.94 – 2.86 (m, 2H), 2.77 (dt, *J* = 13.3, 9.0 Hz, 1H), 1.76 (dq, *J* = 3.8, 2.2 Hz, 1H), 1.56 – 1.49 (m, 2H), 1.21 (dddd, *J* = 14.4, 9.3, 4.8, 1.6 Hz, 1H).

[Non-overlapping] dihydroquinidine peaks: δ 1.69 (s, 0.17H (1H)), 1.40 1.43 – 1.36 (m, 0.46H, (3H)), 0.86 (t, *J* = 7.2 Hz, 0.46H, (3H)). Peaks matched reported literature $^{1}$H NMR peaks for dihydroquinidine. (*22*)

**Quinidinium trifluoroacetate**

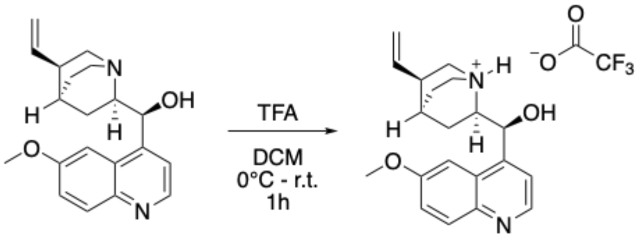


Quinidine (Acros organics, 98%, anhydrous, CAS 56-54-2, Code: 163680250, 6.49 g, 20.0 mmol) was dissolved in DCM (50 mL) and cooled to 0°C with an ice bath under $N_2$. TFA (Fluorochem, 2.30 mL, 30 mmol, 1.5 eq) was added dropwise and stirred for 1 h to room temperature. The reaction mixture was diluted with DI water (c.a. 80 mL) and extracted three times with DCM. The organic extracts were then washed twice with water, dried with $MgSO_4$, filtered and the solvent removed under vacuum. Dried under high vacuum to give a pale yellow solid (5.066g, 11.55 mmol, 58% yield). This material was judged to contain ~18% of the analogous dihydroquinidine impurity by $^{1}$H-NMR.

**$^{1}$H NMR** (400 MHz, $CDCl_3$) δ 12.37 (s, 1H), 8.66 (d, *J* = 4.6 Hz, 1H), 7.82 (d, *J* = 9.2 Hz, 1H), 7.63 (d, *J* = 4.5 Hz, 1H), 7.16 (dd, *J* = 9.2, 2.5 Hz, 1H), 6.95 (d, *J* = 2.7 Hz, 1H), 6.39 (s, 1H), 6.20 (s, 1H), 6.03 (ddd, *J* = 17.4, 10.5, 7.3 Hz, 1H), 5.33 – 5.17 (m, 2H), 4.36 (ddd, *J* = 13.3, 8.4, 2.3 Hz, 1H), 3.79 (s, 3H), 3.40 – 3.23 (m, 3H), 3.17 (dt, *J* = 13.0, 9.7 Hz, 1H), 3.02 (s, 1H), 2.56 (q, *J* = 8.8 Hz, 1H), 2.39 (dd, *J* = 13.5, 9.9 Hz, 1H), 1.97 (d, *J* = 4.6 Hz, 1H), 1.90 (t, *J* = 12.7 Hz, 1H), 1.74 – 1.63 (m, 1H).

**$^{13}$C NMR** (101 MHz, $CDCl_3$) δ 163.1 (q, *J* = 35.2 Hz), 158.4, 146.5, 144.65, 142.9, 136.1, 130.9, 125.3, 122.6, 118.5, 117.8, 121.7 – 111.8 (m), 99.5, 66.5, 60.1, 55.9, 49.3, 48.3, 37.4, 27.5, 23.4, 17.9.

**$^{19}$F NMR** (376 MHz, $CDCl_3$) δ -76.34.

**HRMS** [M]$^+$ calculated for $[C_{20}H_{25}N_2O_2]^+$ 325.1911, found 325.1915, Δ = 1.2 ppm.

$[\alpha]_D^{25}$= + 134.0° (c 1.0, $CHCl_3$).

**Synthesis of Quinidinium/racemic sSPhos salt**

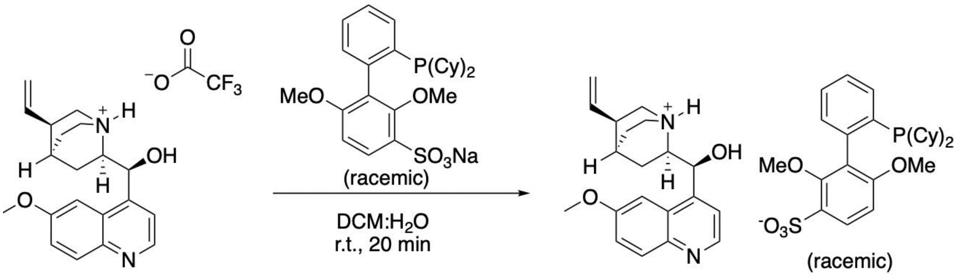


Sodium sSPhos (4.75 g, 9.26 mmol, 1.0 eq) was stirred in DCM (20 mL, anhydrous) and then quinidinium trifluoroacetate (4.26 g, 9.73 mmol, 1.05 eq) was added in one portion and stirred for 40 mins at room temperature. Water was added (3 mL) and the mixture was stirred for a further 20 mins. The mixture was then diluted with water (50 mL) and extracted with DCM three times, dried with $MgSO_4$, filtered and the solvent removed under vacuum and the solid dried under high vacuum to give a light yellow solid (5.318 g, 6.53 mmol, 71% yield). This material was judged to contain ~15% of the analogous dihydroquinidine impurity by $^1$H-NMR.

It was noted by the authors that for the subsequent recrystallisation to work effectively, the quinidinium/sSPhos salt needed to be thoroughly dried under high vacuum to remove any traces of dichloromethane.

**$^1$H NMR** (500 MHz, $CDCl_3$) δ 11.18 (s, 1H), 8.71 (dt, *J* = 4.5, 2.2 Hz, 1H), 8.12 (dd, *J* = 8.8, 1.4 Hz, 0.5H), 7.96 (dd, *J*= 8.8, 2.0 Hz, 0.5H), 7.92 (dd, *J* = 9.3, 2.0 Hz, 1H), 7.66 (dd, *J* = 4.6, 1.7 Hz, 1H), 7.55 (dd, *J* = 7.0, 2.4 Hz, 0.5H), 7.51 (dq, *J* = 7.3, 2.4 Hz, 0.5H), 7.39 – 7.30 (m, 3H), 7.24 – 7.17 (m, 1H), 6.61 (ddd, *J* = 8.6, 6.2, 1.7 Hz, 1H), 6.41 (d, *J* = 12.4 Hz, 1H), 6.04 – 5.87 (m, 2H), 5.29 (d, *J* = 1.7 Hz, 0.5H), 5.22 – 5.16 (m, 2H), 4.26 – 4.14 (m, 1H), 3.93 (dd, *J*= 6.0, 2.2 Hz, 4H), 3.67 (d, *J* = 1.8 Hz, 1.50H), 3.60 (d, *J* = 1.8 Hz, 2H), 3.52 – 3.38 (m, 4H), 3.35 (s, 1.5H) 3.31 – 3.21 (m, 1H), 2.53 (q, *J* = 8.9 Hz, 1H), 2.38 – 2.29 (m, 1H), 1.82 – 1.45 (m, 10H), 1.32 – 0.80 (m, 15H).

**$^{13}$C NMR** (126 MHz, $CDCl_3$) δ 159.9, 159.8, 158.86, 155.7, 155.5, 147.1, 144.8, 143.9 141.9, 141.8, 141.6, 141.6, 136.5, 132.6 – 132.4 (m), 132.4 – 132.2 (m), 131.4, 131.0, 129.7, 129.6, 128.4, 126.9 (d, *J* = 4.3 Hz), 126.88, 125.9, 122.8, 119.0, 117.7, 105.2, 105.0, 100.7, 66.6, 61.2, 61.1, 60.2, 57.1, 55.6, 53.6, 50.5, 49.6, 48.7, 37.52, 35.4 – 35.2 (m), 32.9 – 32.4 (m), 30.2 (d, *J* = 16.9 Hz), 29.6 – 29.2 (m), 27.8 – 27.5 (m) 27.27 – 27.03 (m), 26.56 – 26.35 (m),26.15, 23.51, 18.15.

**$^{31}$P NMR** (162 MHz, $CDCl_3$) δ -8.50. $^{31}$P NMR showed 1-2% oxidised impurity

**HRMS** [M]$^-$ calculated for $[C_{26}H_{34}O_5PS]^-$ 489.1870, found 489.1877, Δ = 1.4 ppm.

**HRMS** [M]$^+$ calculated for $[C_{20}H_{25}N_2O_2]^+$ 325.1911, found 325.1907, Δ = 1.2 ppm.

$[\alpha]_D^{25}$= + 85.5° (c 1.0, $CHCl_3$).

**Procedure for first recrystallisation:**

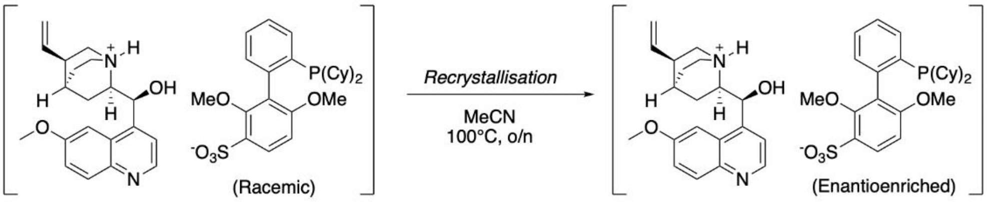


Quinidinium/racemic sSPhos salt (2.32 g, 2.85 mmol) in MeCN (50 mL, HPLC Grade S obtained from Rathburn Chemicals (Cat No. RH1016)) in a 100 mL round bottom flask with reflux condenser attached was heated to 100 °C in a heating block with stirring. When the salt had fully dissolved, both heating and stirring were turned off and the solution was allowed to cool to room temperature in the heating block overnight, forming a colourless precipitate. The precipitate was filtered and washed with MeCN at room temperature and dried at room temperature under high vacuum to give a colourless solid (873 mg, 1.07 mmol, 38% yield). This material was judged to contain ~12% of the analogous dihydroquinidine impurity by $^{1}$H-NMR.

If the racemic quinidinium/sSPhos salt immediately dissolves in MeCN at room temperature it is likely there is residual solvent present and further drying *in vacuo* is required to carry out the recrystallisation successfully. If the quinidinium/sSPhos salt does not fully dissolve at 100 °C, further MeCN can be added (up to 20 mL). If the salt has still not dissolved a hot filtration can be used to remove any excess racemic salt. Likewise, if no precipitate has formed after the solution has been left overnight at room temperature, scratching the inside of the recrystallisation glassware with a metal spatula can encourage crystallisation to occur. The recrystallisation should not be left precipitating for multiple days, as this has been found to yield a precipitate with lower diastereomeric enrichment.

**$^{1}$H NMR** (400 MHz, $CDCl_3$) δ 11.10 (s, 1H), 8.70 (d, *J* = 4.5 Hz, 1H), 7.94 (dd, *J* = 9.0, 5.2 Hz, 2H), 7.65 (d, *J* = 4.5 Hz, 1H), 7.50 (dt, *J* = 5.1, 2.7 Hz, 1H), 7.38 (d, *J* = 2.7 Hz, 1H), 7.34 – 7.30 (m, 2H), 7.27 (d, *J* = 8.2 Hz, 1H), 7.16 (dt, *J* = 5.6, 3.4 Hz, 1H), 6.62 (d, *J* = 8.8 Hz, 1H), 6.51 (d, *J* = 4.9 Hz, 1H), 5.99 (ddd, *J* = 17.4, 10.6, 7.3 Hz, 1H), 5.78 (d, *J*= 5.1 Hz, 1H), 5.25 – 5.14 (m, 2H), 4.22 (ddd, *J* = 13.2, 8.4, 2.3 Hz, 1H), 3.90 (s, 3H), 3.67 (s, 3H), 3.56 – 3.42 (m, 2H), 3.34 (s, 3H), 2.86 (s, 1H), 2.56 (q, *J* = 8.8 Hz, 1H), 2.43 – 2.32 (m, 1H), 1.93 – 1.80 (m, 2H), 1.76 – 1.46 (m, 9H), 1.46 – 1.35 (m, 4H), 1.19 – 0.77 (m, 13H).

**$^{13}$C NMR** (101 MHz, $CDCl_3$) δ 159.9, 158.9, 155.5, 147.2, 144.7, 144.5, 144.1, 141.7 (d, *J* = 30.8 Hz), 136.5, 132.5 (d, *J* = 3.1 Hz), 132.2 (d, *J* = 6.4 Hz), 131.5, 130.7, 129.5, 128.3, 126.9, 126.5 (d, *J* = 6.5 Hz), 125.9, 122.8, 119.0, 117.7, 105.0, 100.5, 66.5, 61.1, 60.3, 57.2, 55.5, 49.6, 48.7, 37.5, 35.2 (d, *J* = 11.9 Hz), 32.8 (d, *J* = 10.2 Hz), 30.1, 29.9, 29.6 – 29.5 (m), 29.5 – 29.4 (m), 27.8 – 27.7 (m), 27.7, 27.7 – 27.6 (m), 27.2 (d, *J* = 9.5 Hz), 26.9 (d, *J* = 11.5 Hz), 26.5, 26.2, 23.5, 18.1.

**$^{31}$P NMR** (162 MHz, $CDCl_3$) δ -8.67.

**HRMS** $[M]^-$ calculated for $[C_{26}H_{34}O_5PS]^-$ 489.1870, found 489.1865, Δ = 1.0 ppm.

**HRMS** $[M]^+$ calculated for $[C_{20}H_{25}N_2O_2]^+$ 325.1911, found 325.1910, Δ = 0.3 ppm.

$[\alpha]_D^{25}$= + 65.2° (c 1.0, $CHCl_3$).

**X-Ray Structure of the Enantioenriched Quinidinium/sSPhos Salt**

Crystals of the enantioenriched quinidinium/sSphos salt could be grown by redissolving the salt obtained by the above procedure in MeCN, heating to 100 °C again and then allowing the sample to

slowly cool. The crystals were analysed by x-ray diffraction, confirming the absolute configuration of the enantioenriched sSPhos to be (*R*). The structure was deposited in the Cambridge Crystallographic Data Centre (deposition no. CCDC 2171203).

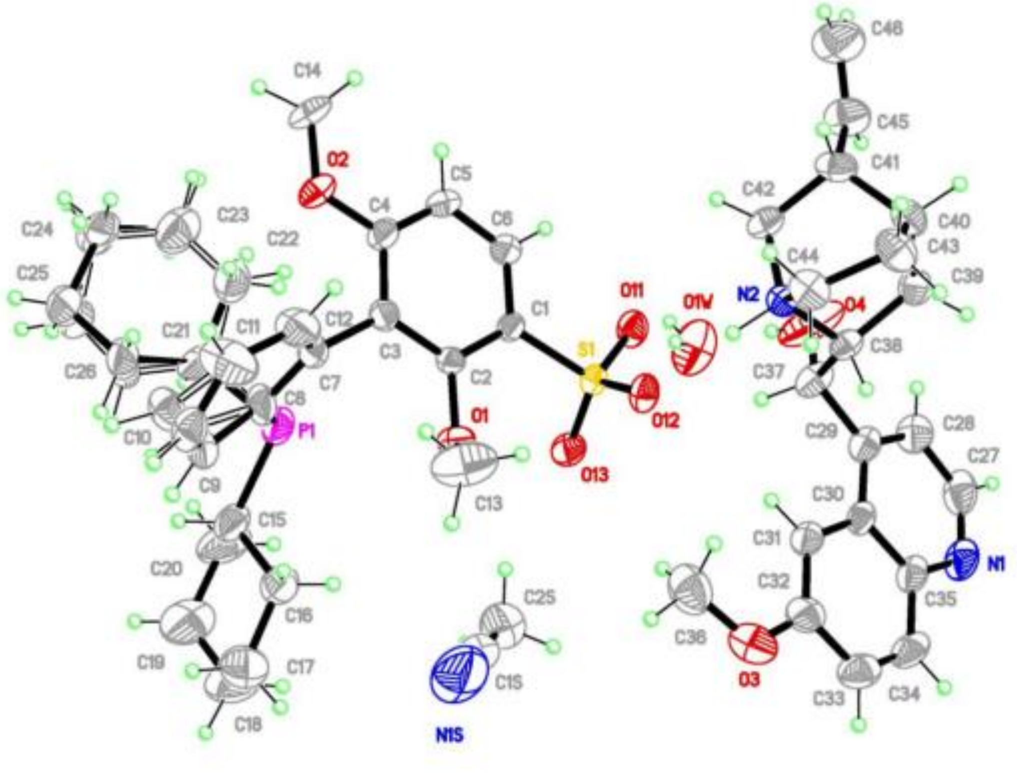

**Procedure to generate sodium sSPhos from enantioenriched quinidinium/sSPhos salt**

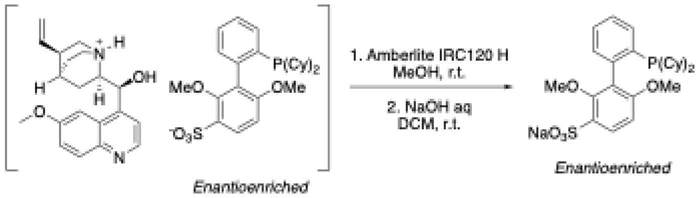


Enantioenriched quinidinium/sSPhos salt (200 mg, 0.245 mmol) was dissolved in MeOH (c.a. 10 mL), and passed through a column of Amberlite IRC120 H, hydrogen form (washed first with MeOH until run clear) five times and the solvent removed under reduced pressure to give the protonated zwitterionic (*R*)-sSPhos ($^{31}$**P NMR** (MeOD) δ 21.26). The protonated zwitterionic (*R*)-sSPhos was then dissolved in NaOH solution (10%, c.a. 30 mL) and extracted three times with DCM, washed with deionised water and the solvent removed under reduced pressure to give (*R*)-Na sSPhos (116 mg, 0.226 mmol, 92%).

The overall single recrystallisation procedure followed by cation exchange to give the sodium salt of sSPhos was repeated independently three times using separately synthesised Quinidinium/sSPhos salts from several batches of *rac*-NasSPhos and quinidine obtained from Acros Organics. This proved to be very reproducible as indicated in the table below. In these cases the enantioenrichment of Na sSPhos was determined by chiral SFC analysis of the sodium salt (column details). Due to broadening of the minor peak and peak shape distortion at higher injection concentrations it was difficult get a precise ee value but it could be determined to be in the region of 94-96% ee in all three cases.

| Exp | Yield of quinidinium TFA salt | Yield of *rac*-sSPhos/quinidinium salt | Yield of recrystallized sSPhos/quinidinium salt | % *ee* of NasSPhos | $[\alpha]_D^{25}$ of NasSPhos in MeOH |
|---|---|---|---|---|---|
| 1 | 5.067 g, 58% | 5.318 g, 71% | 873 mg, 38% | 94-96% | -44.3 (c = 0.46) |
| 2 | 5.610 g, 64% | 6.967 g, 93% | 687 mg, 30% | 94-96% | -42.5 (c = 0.12) |
| 3 | 4.330 g, 49% | 6.154 g, 81% | 692 mg, 30% | 94-96% | -43.0 (c = 0.46) |

## Double recrystallisation procedure of *rac*-sSPhos/quinidinium salt (Scheme 2F)

In order to obtain higher ee final material, racemic quinidinium/sSPhos salt was subjected to two successive rounds of recrystallisation. In order to accurately determine the ee at the intermediate and final stages, the material was derivatised to the sSphos neopentyl sulfonate ester (with concomitant oxidation to the phosphine oxide). This allowed accurate analysis on chiral SFC.

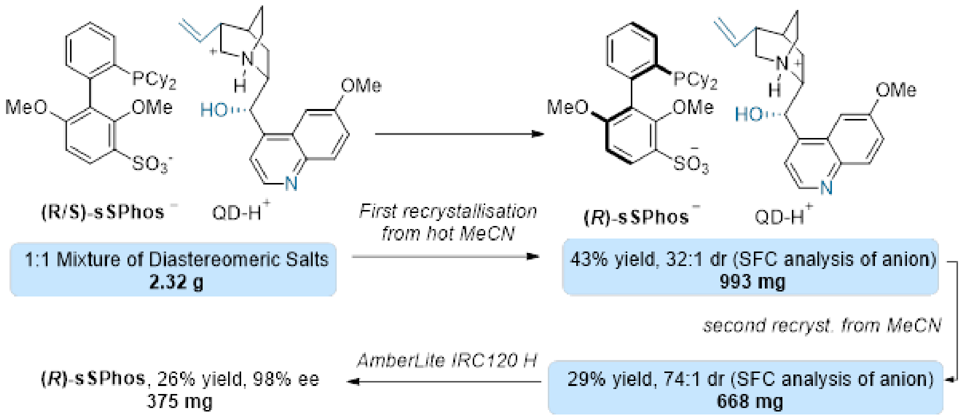


*Rac*-sSPhos/quindinium salt (2.32 g, 2.85 mmol) was suspended in MeCN (50 mL) at which point the salt fully dissolved. The MeCN was removed under reduced pressure to azeotrope off excess DCM from the previous step, and then the sSPhos/quindine salt was re-suspended in MeCN (50 mL), this time without going into solution. The mixture was heated to reflux at 100 °C under air for 5 minutes, at which point the mixture had mostly dissolved. The hot mixture was passed through a frit before being left to cool for 16 h in the heating mantle that was used for the recrystallisation. The crystalline precipitate was filtered and washed with room temperature MeCN (2 x 10 mL) before being dried under vacuum to afford the diastereomerically-enriched salt (993 mg, 1.23 mmol, 43%, 32:1 dr). At this stage, 20 mg of salt was taken out and first converted to sodium sSPhos and then derivatized to the neopentyl sulfonate ester/phosphine oxide for SFC analysis which showed a 94% ee of the anion (corresponding to a 32:1 dr in the salt). The remaining salt was re-suspended in MeCN (21 mL) and heated to 100 °C. At 100 °C some of the salt remained insoluble so further MeCN (6 mL) was added, at which point most of the salt dissolved. The hot mixture was passed through a frit before being left to cool for 16 h in the heating mantle that was used for the recrystallisation. The crystalline precipitate was filtered and washed with room temperature MeCN (2 x 10 mL) before being dried under vacuum to afford the further enantioenriched salt (668 mg, 1.23 mmol, 29%, 74:1 dr). Again, 20 mg of salt was taken out and derivatized as above for SFC analysis, which showed a 97% ee of the anion (corresponding to a 74:1 dr in the salt). The remaining salt was dissolved in MeOH

(20 mL) and passed through a column of Amberlite IRC120 H, hydrogen form (washed first with MeOH until run clear) five times and the solvent removed under reduced pressure to give the protonated zwitterionic (*R*)-sSPhos (**$^{31}$P** NMR (MeOD) δ 21.26). The protonated zwitterionic (*R*)-sSPhos was then dissolved in NaOH solution (10%, 30 mL), extracted with DCM (3 x 30 mL), washed with deionised water (2 x 30 mL) and the solvent removed under reduced pressure to give (*R*)-Na.sSPhos (375 mg, 0.732 mmol, 26% from original racemic salt). A sample of this material was derivatized to the neopentyl sulfonate ester/phosphine oxide for SFC analysis, which showed a 98% ee in the final material (see procedure below).

**$^{1}$H NMR** (400 MHz, MeOD) δ 7.91 (d, J = 8.8 Hz, 1H), 7.63 (dt, J = 6.5, 2.6 Hz, 1H), 7.49 – 7.31 (m, 2H), 7.29 – 7.22 (m, 1H), 6.78 (d, J = 8.8 Hz, 1H), 3.71 (s, 3H), 3.41 (s, 3H), 2.12 – 0.95 (m, 22H).

**$^{13}$C NMR** (101 MHz, MeOD) δ 159.8, 155.7, 141.7 (d, J = 31.9 Hz), 136.4 (d, J = 17.7 Hz), 132.2 (d, J = 3.5 Hz), 131.8 (d, J = 6.2 Hz), 130.4, 128.5, 127.7, 126.4 – 126.3 (m), 104.3, 60.1, 54.6, 35.4 (d, J = 14.3 Hz), 33.3 (d, J = 13.0 Hz), 30.2 – 29.9 (m), 29.6 (d, J = 12.2 Hz), 29.3 (d, J = 12.5 Hz), 27.3 (d, J = 8.3 Hz), 27.2 (d, J = 8.9 Hz), 26.9 (d, J = 9.4 Hz), 26.8 (d, J = 11.4 Hz), 26.3, 26.1.

**$^{31}$P NMR** (162 MHz, MeOD) δ -9.18.

**HRMS** $[M]^-$ calculated for $[C_{26}H_{34}O_5PS]^-$ 489.1870, found 489.1871, Δ = 0.2 ppm.

$[\alpha]_D^{25}$ = -44.2° (c 0.45, MeOH).

| **Yield after 1$^{st}$recrystallisation of sSPhos/quinidinium salt** | **Yield after 2$^{nd}$recrystallisation of sSPhos/quinidinium salt** | **% *ee* of 2$^{nd}$recrystallisation of sSPhos/quinidinium salt** | **$[\alpha]_D^{25}$of NasSPhos in MeOH** |
|---|---|---|---|
| 993 mg, 43% | 668 mg, 29% | 98 % *ee* | -44.2° |

**Derivatisation procedure:**

**Neopentyl (*R*)-2'-(dicyclohexylphosphoryl)-2,6-dimethoxy-[1,1'-biphenyl]-3-sulfonate**

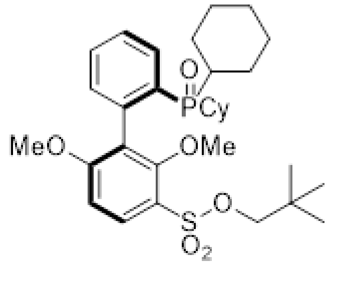


$SOCl_2$ (1.0 mL) was added slowly to sSPhos enriched in the (R) enantiomer, obtained after the second recrystallisation described above (51.2 mg, 0.100 mmol) in a vial at room temperature and the vial was sealed and placed under an atmosphere of nitrogen by three evacuation/refill cycles. DMF (0.1 mL) was added and the reaction mixture was heated to 70 °C for 2 h, at which point the $SOCl_2$ was removed under a stream of air. The crude mixture was diluted in EtOAc (1 mL) and $H_2O$ (0.5 mL) was added at 0 °C to quench any residual $SOCl_2$ remaining. The layers were isolated and the aqueous layer was extracted with further EtOAc (3 x 1 mL). The combined organic extracts were dried over $MgSO_4$ and concentrated under a stream of air to afford the crude sulfonyl chloride intermediate. The crude material was taken up in anhydrous DCM (0.5 mL) and neopentyl alcohol (44.1 mg, 0.500 mmol), pyridine (0.02 mL, 0.200 mmol) and DMAP (1.22 mg, 10mol%) were added sequentially. The reaction

mixture was sealed and heated to 45 °C for 16 h, at which point the solvent was removed under a stream of air. The crude material was purified via flash column chromatography eluting in a 50-80% EtOAc in pet. ether solvent system to afford the title product as a colourless solid (26.9 mg, 46.6 µmol, 47%, 98% ee).

**$^{1}$H NMR** (400 MHz, $CDCl_3$) δ 7.93 (d, J = 8.9 Hz, 1H, $H_5$), 7.64 – 7.41 (m, 3H), 7.32 (dd, J = 6.2, 4.0 Hz, 1H), 6.77 (d, J = 8.9 Hz, 1H), 3.88 (s, 2H), 3.76 (s, 3H), 3.46 (s, 3H), 1.95 – 1.18 (m, 22H), 1.01 (s, 9H).

**$^{13}$C NMR** (101 MHz, $CDCl_3$) δ 162.0, 157.3, 138.6, 133.5 (d, J = 8.5 Hz), 131.9, 130.7 (m), 130.2 (d, J = 2.4 Hz), 127.1 (d, J = 11.0 Hz), 126.4 (d, J = 2.5 Hz), 121.7, 105.3, 79.9, 61.6, 55.7, 38.4 (d, J = 66.3 Hz), 36.9 (d, J = 67.1 Hz), 31.7, 26.8 (d, J = 3.5 Hz), 26.7 (d, J = 3.3 Hz), 26.6 (m), 26.4, 26.3 (m), 25.8 (d, J = 8.1 Hz), 25.5 (d, J = 3.3 Hz), 25.4 (d, J = 2.9 Hz), 25.1 (d, J = 2.9 Hz).

**$^{31}$P NMR** (162 MHz, $CDCl_3$) δ 45.79.

**HRMS** $[M+H]^+$ calculated for $[C_{31}H_{49}O_6PS]^+$ 577.2747, found 577.2752, Δ = 0.9 ppm.

**Chiral SFC Analysis** CHIRAL ART SC ($CO_2$:MeOH, 82:18, 2.5 mL $min^{-1}$, 40 °C) $t_R$ = 7.94 (minor), 9.64 (major) minutes.

$[\alpha]_D^{25}$ = +31.5° (c 1.0, $CHCl_3$).

# Starting Material NMR Spectra

**$^1$H NMR** ($CDCl_3$)**:** 2-bromo-3-chloro-6-methylphenol

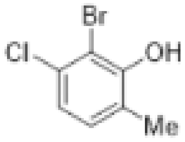


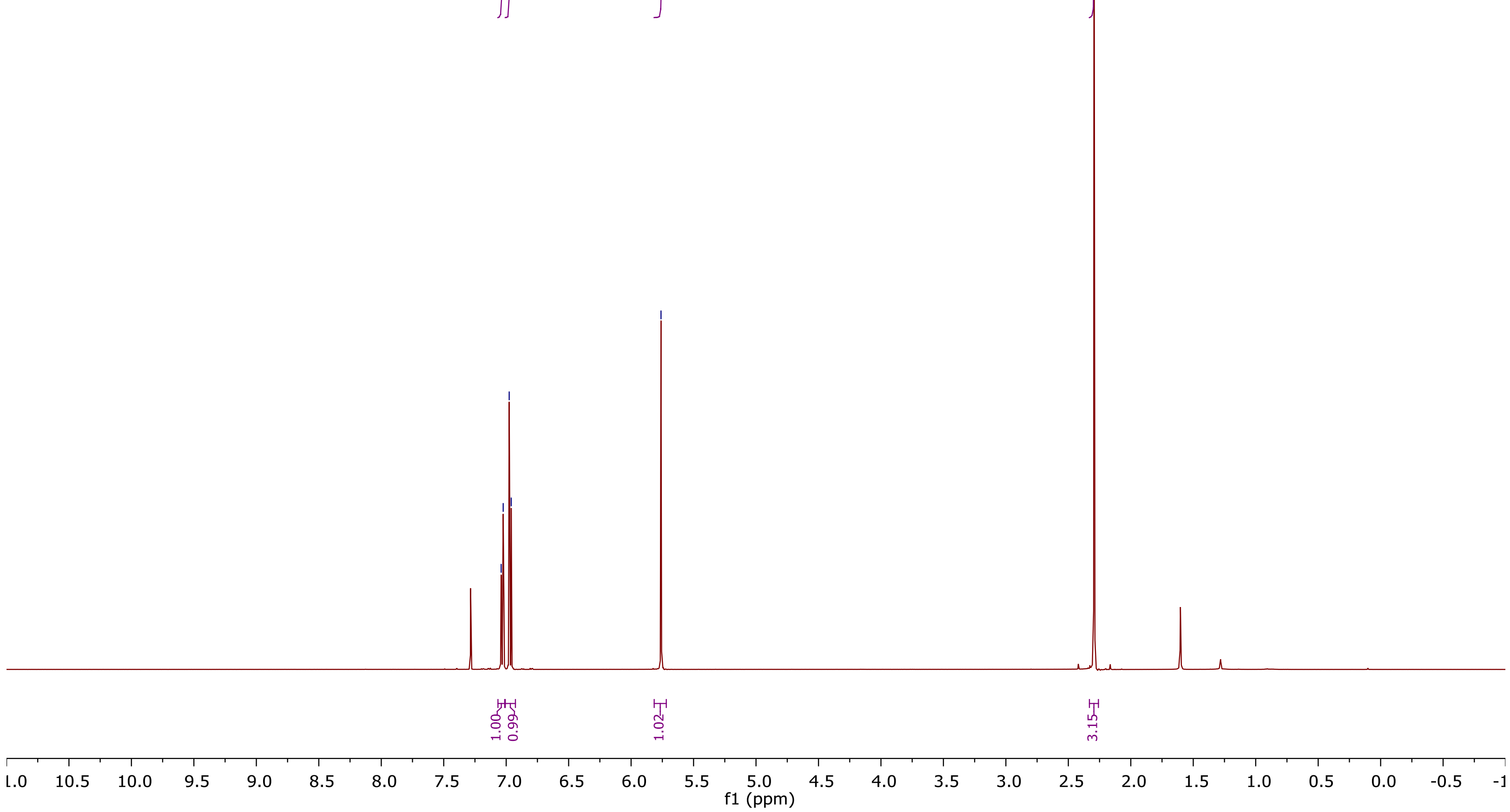

**$^{13}$C NMR** ($CDCl_3$)**:** 2-bromo-3-chloro-6-methylphenol

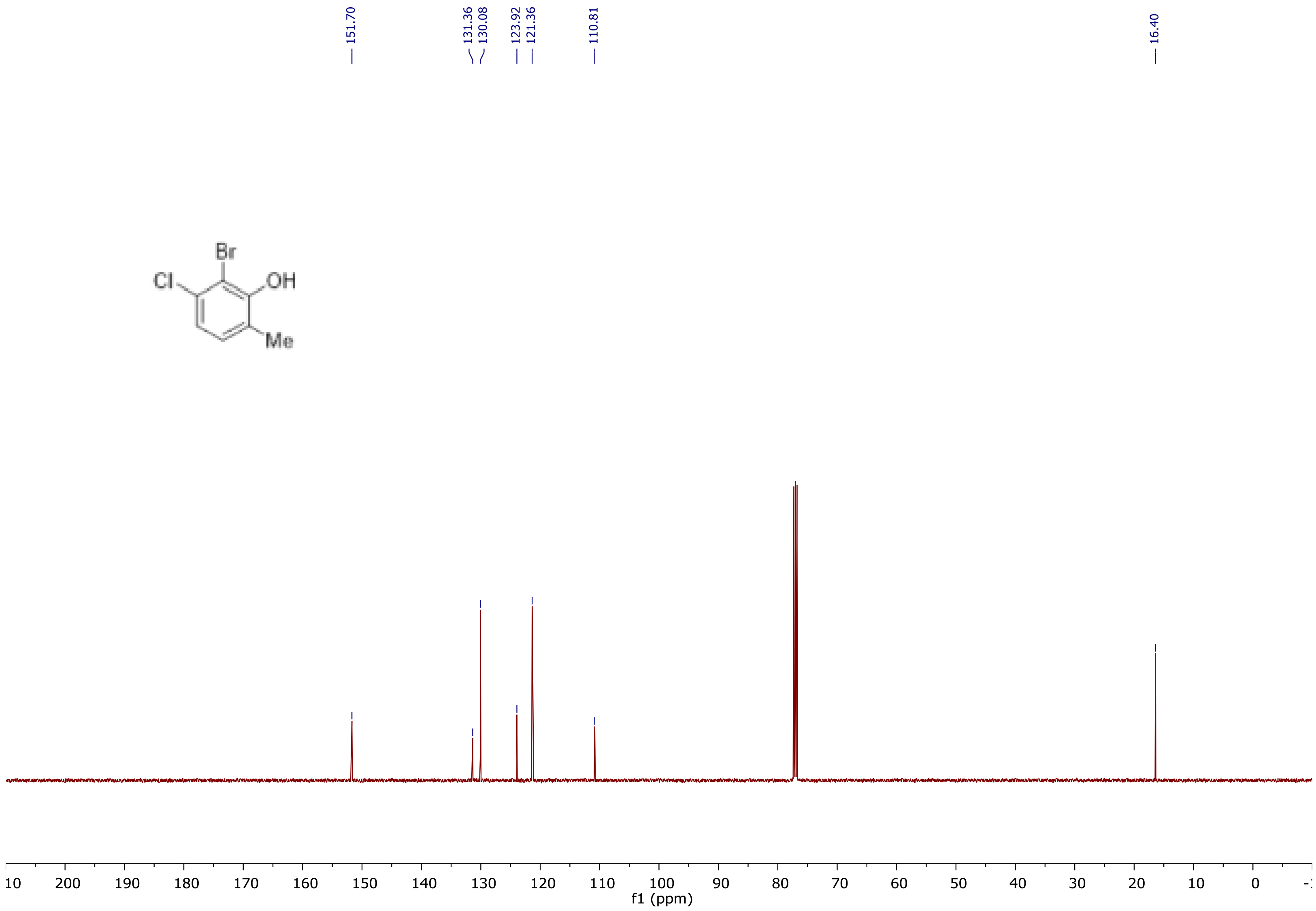

**¹H NMR** ($CDCl_3$)**:** 2-bromo-3,5-dimethylphenol

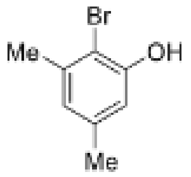

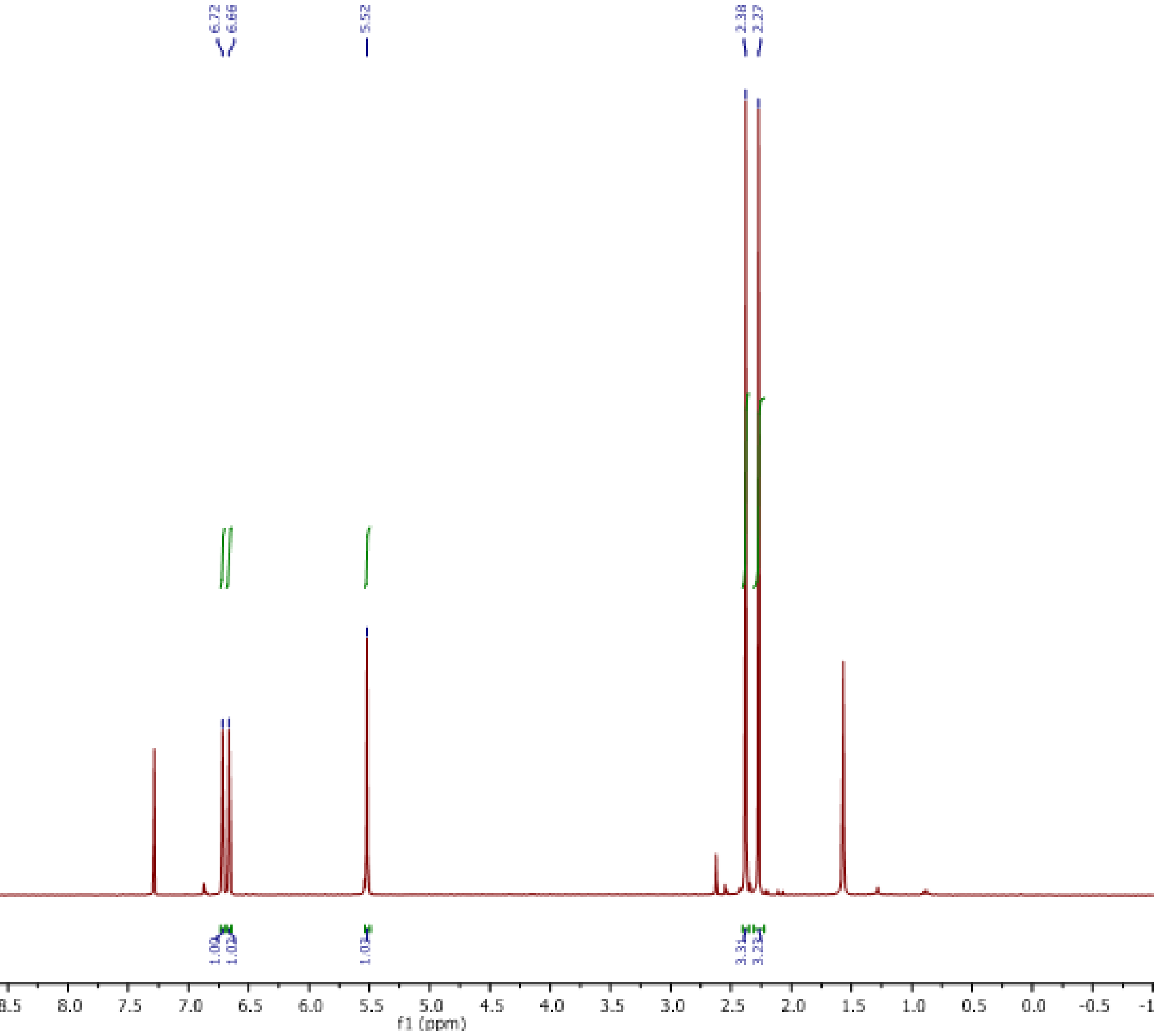

**$^{13}$C NMR** ($CDCl_3$)**:** 2-bromo-3,5-dimethylphenol

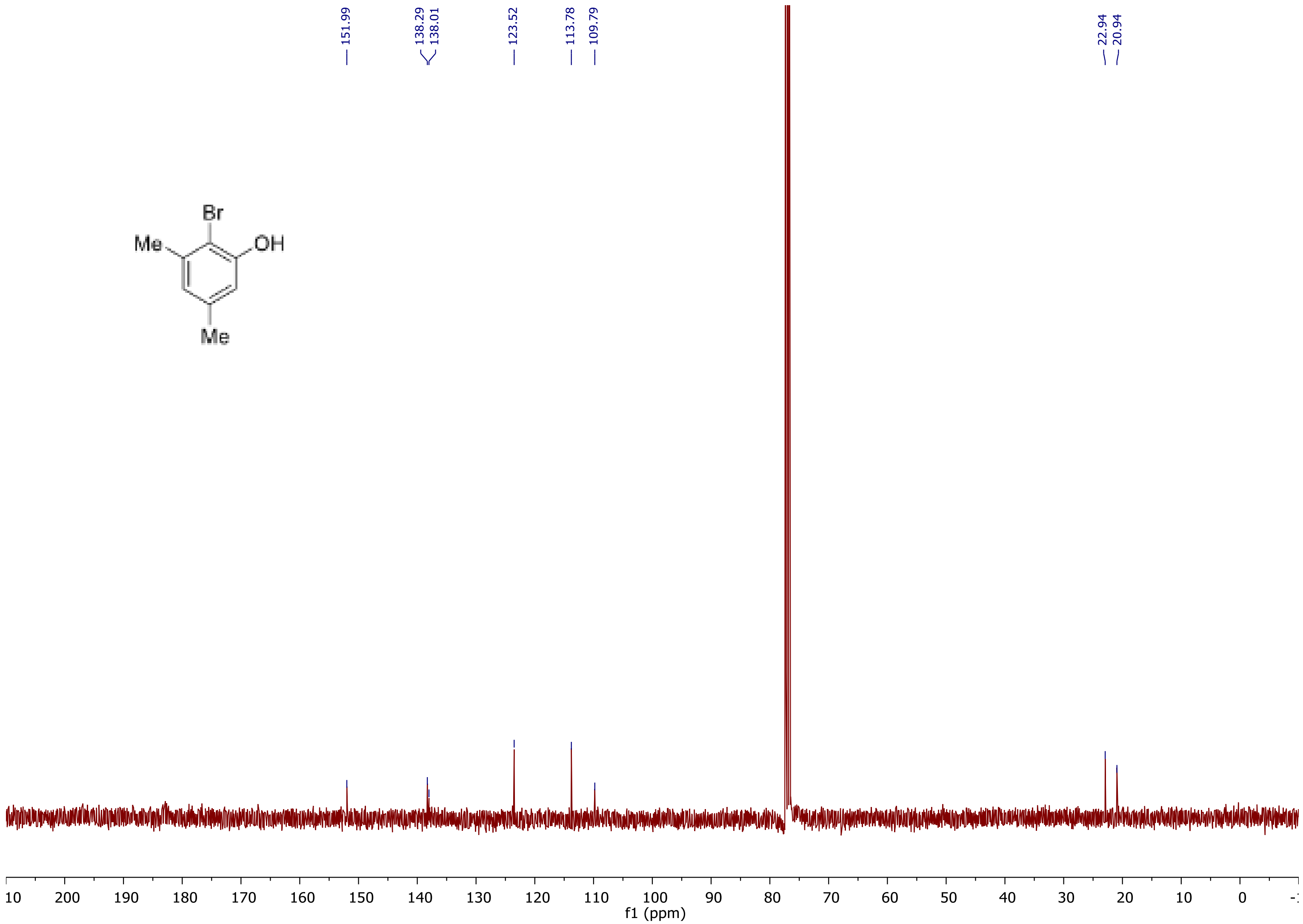

**$^1$H NMR** ($CDCl_3$)**:** 1-bromo-5,6,7,8-tetrahydronaphthalen-2-ol

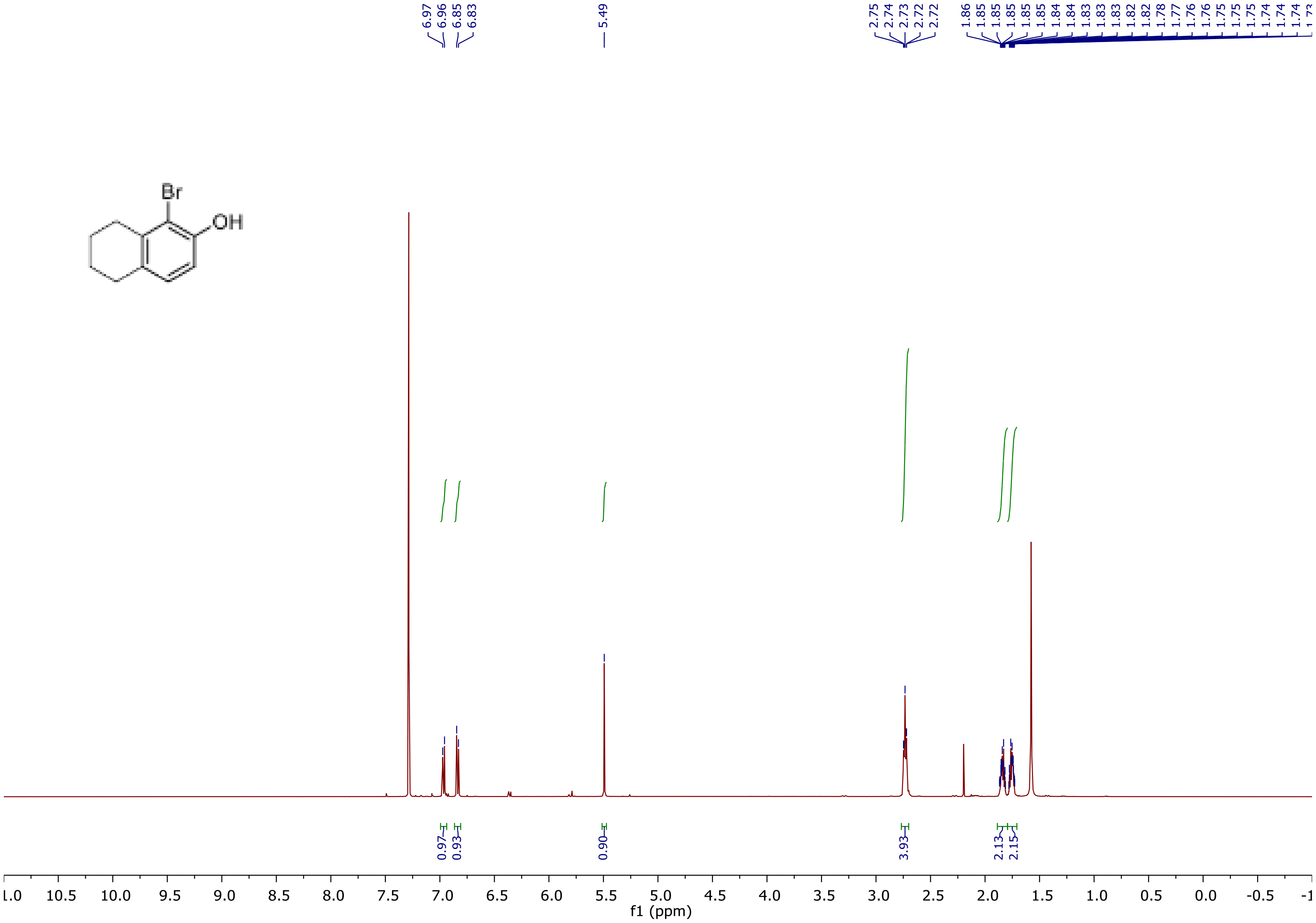

**$^{13}$C NMR** (CDCl$_3$)**:** 1-bromo-5,6,7,8-tetrahydronaphthalen-2-ol

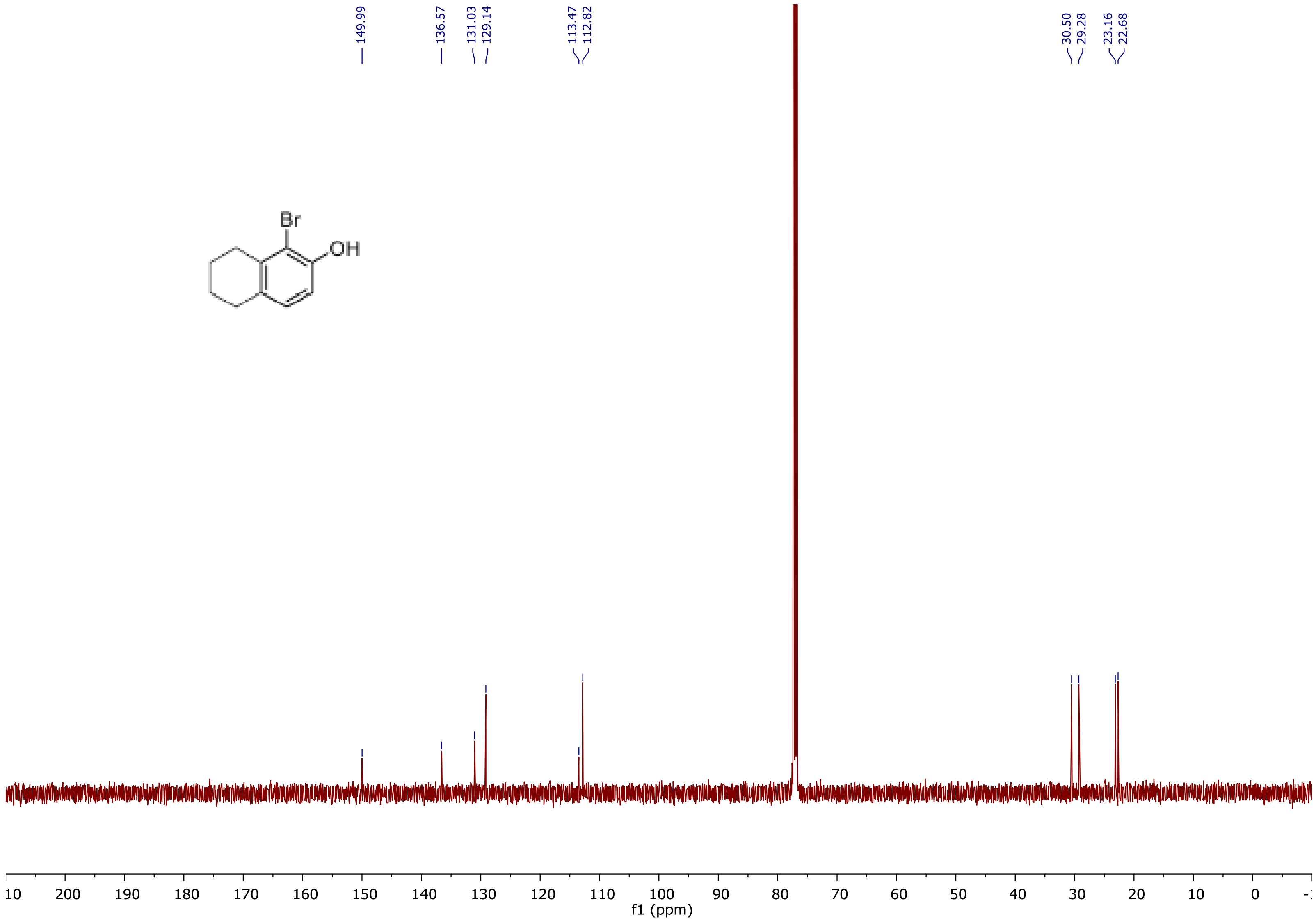

**$^{1}$H NMR** ($CDCl_3$): 1-chloro-3-(methoxymethoxy)benzene

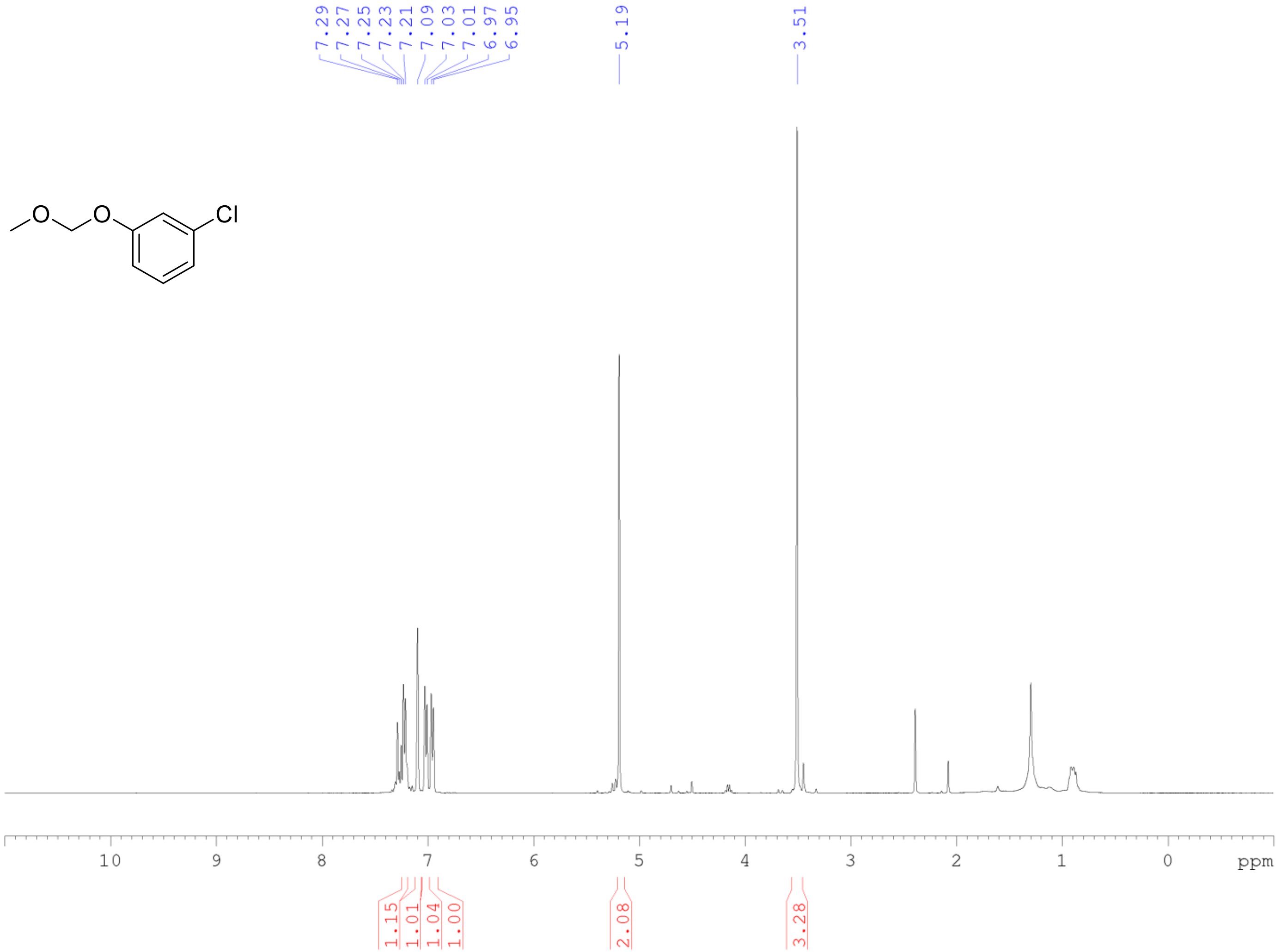

**$^{13}$C NMR** ($CDCl_3$): 1-chloro-3-(methoxymethoxy)benzene

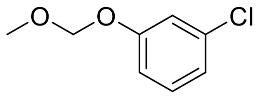

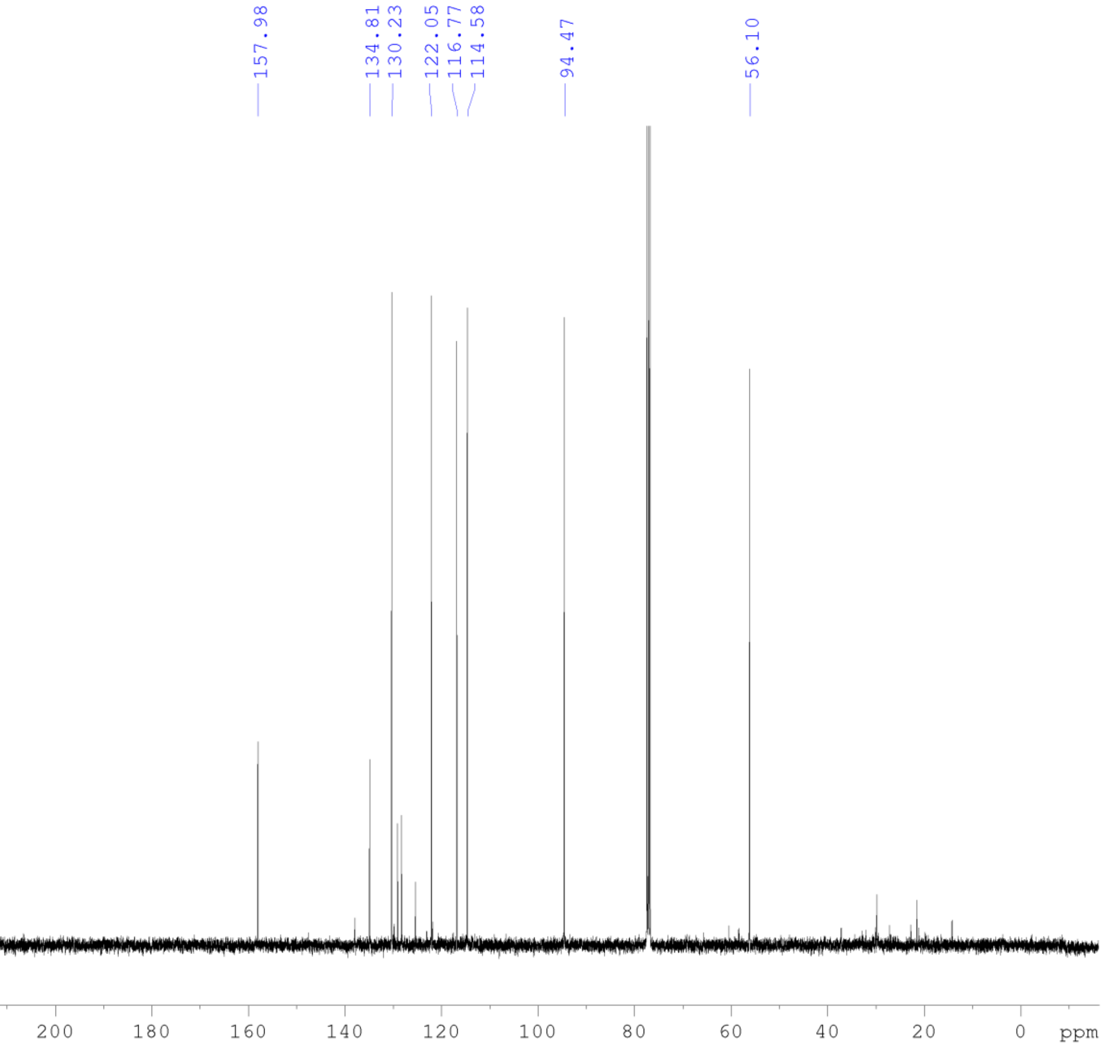

**$^{1}$H NMR** ($CDCl_3$): 2-bromo-1-chloro-3-(methoxymethoxy)benzene

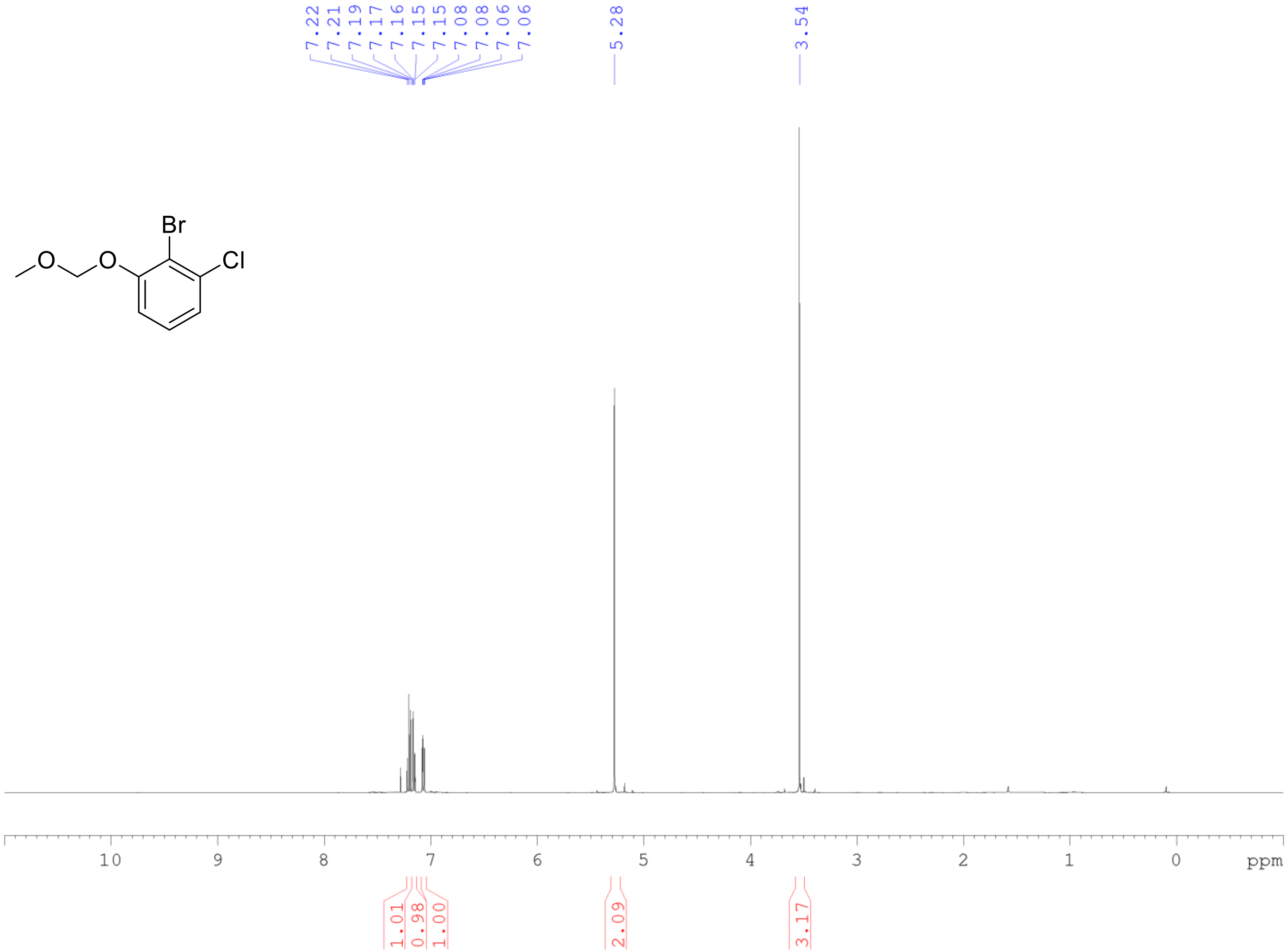

**$^{13}$C NMR** ($CDCl_3$): 2-bromo-1-chloro-3-(methoxymethoxy)benzene

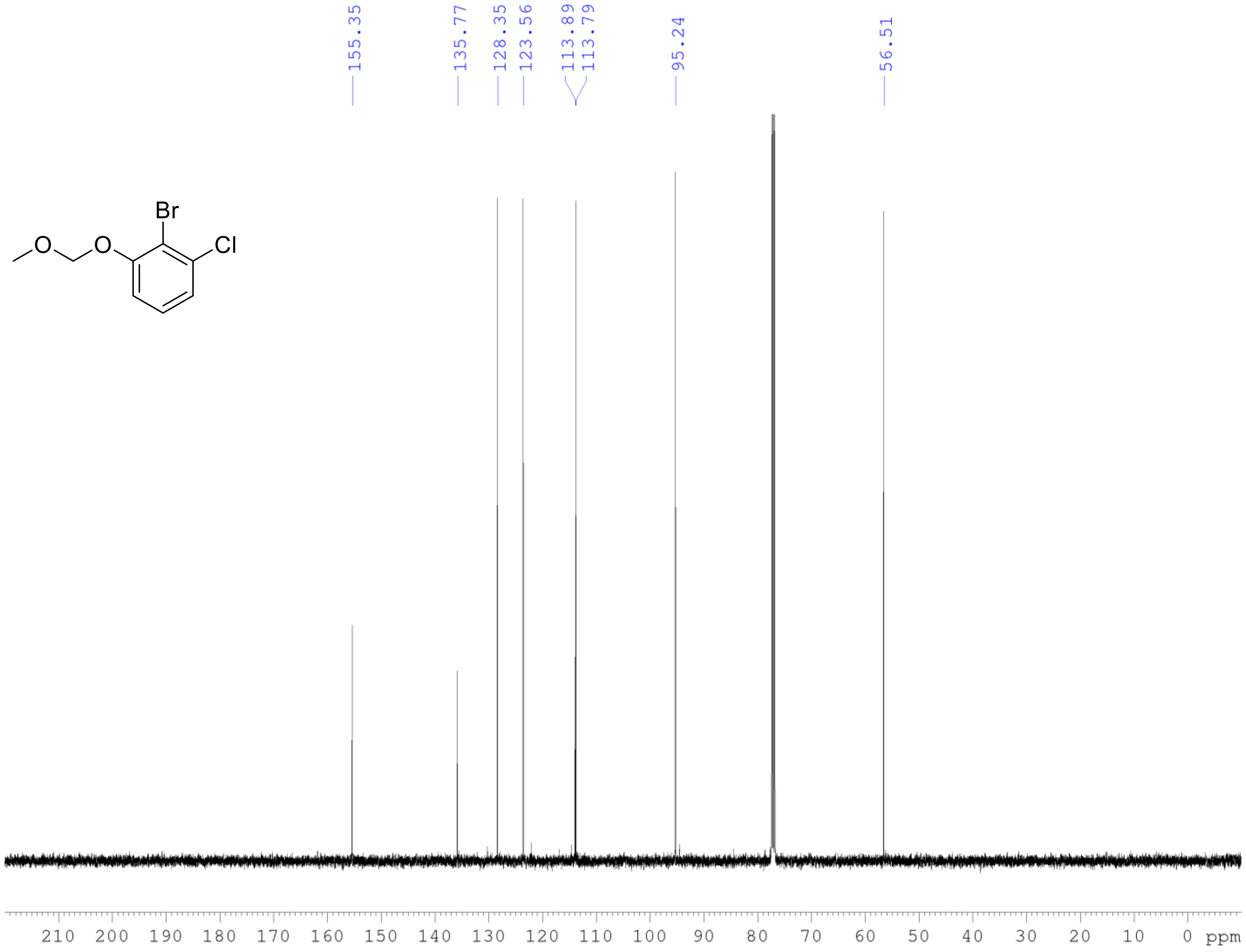

**1H NMR** ($CDCl_3$): 2-bromo-3-chlorophenol

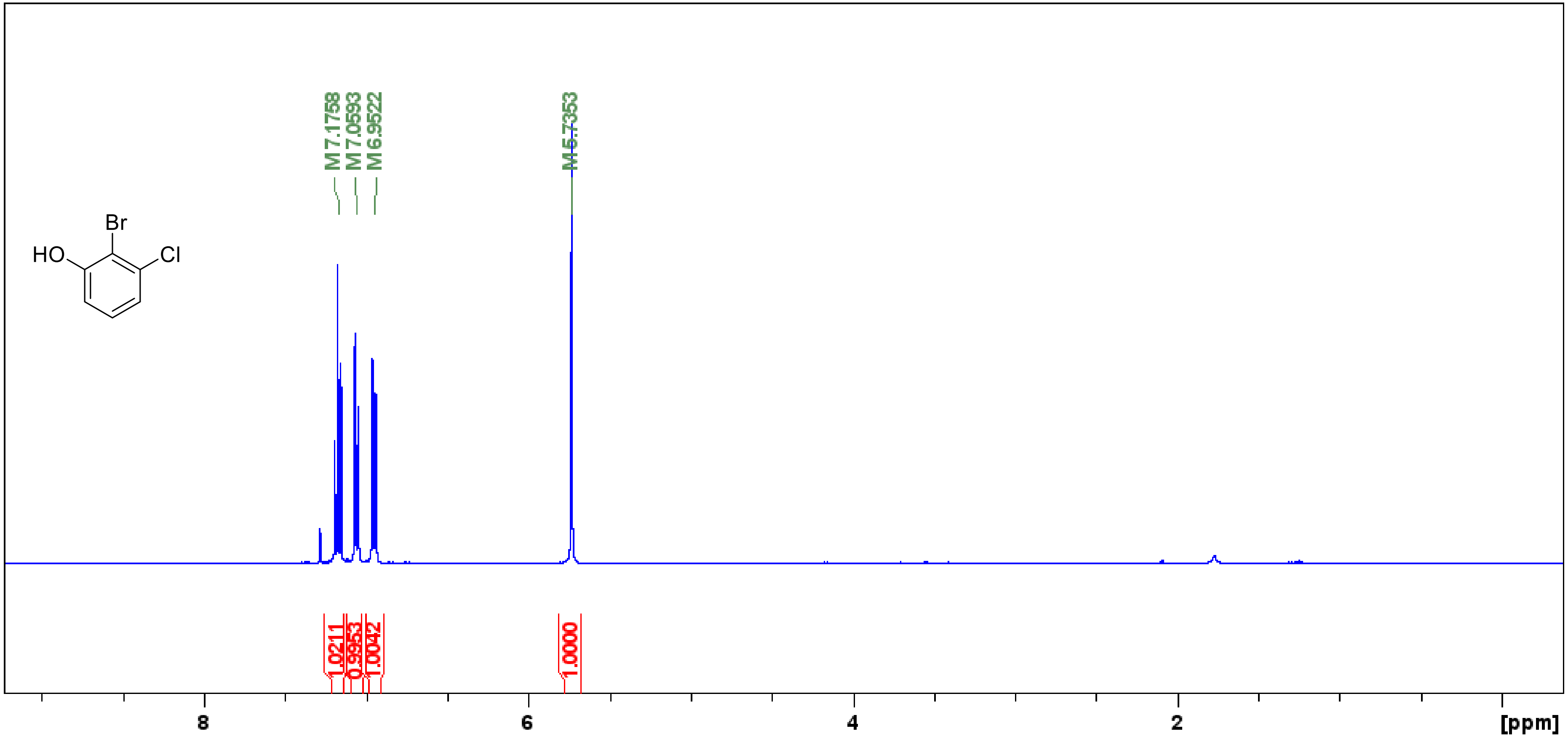

**$^{13}$C NMR** ($CDCl_3$): 2-bromo-3-chlorophenol

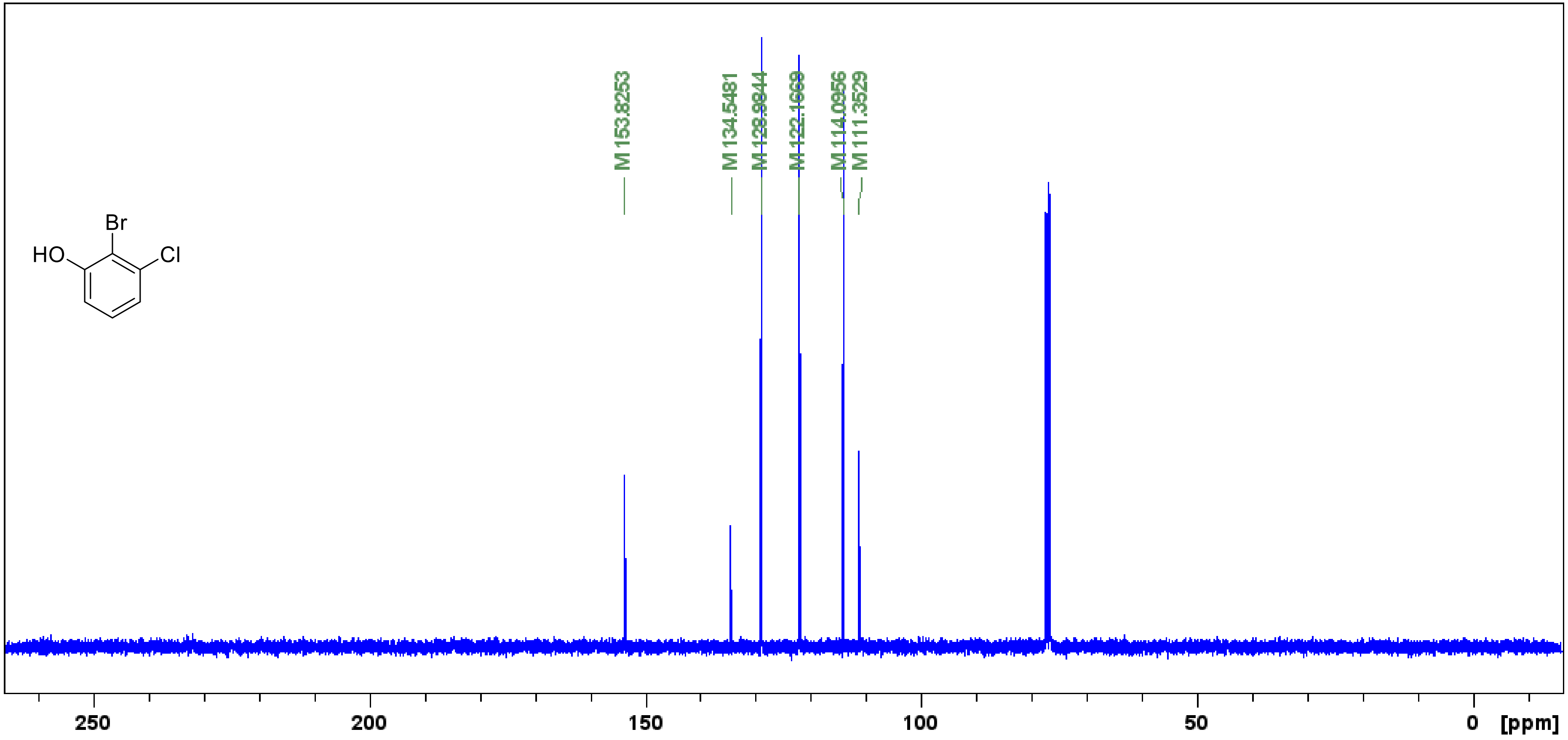

**$^{1}$H NMR** (CDCl$_3$)**:** 2-bromo-3-chloro-1-(methoxymethoxy)-4-methylbenzene

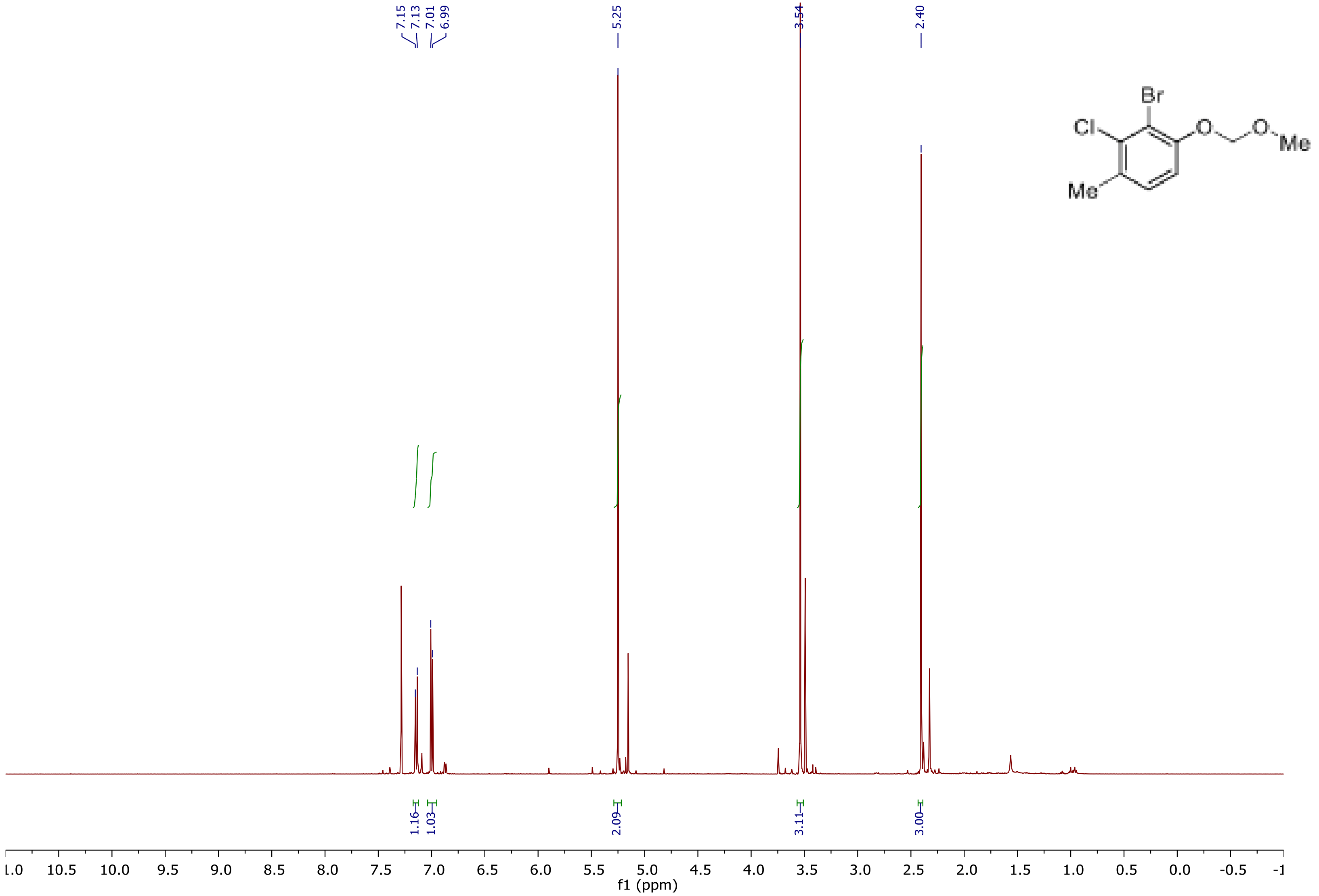

**$^{13}$C NMR** ($CDCl_3$): 2-bromo-3-chloro-1-(methoxymethoxy)-4-methylbenzene

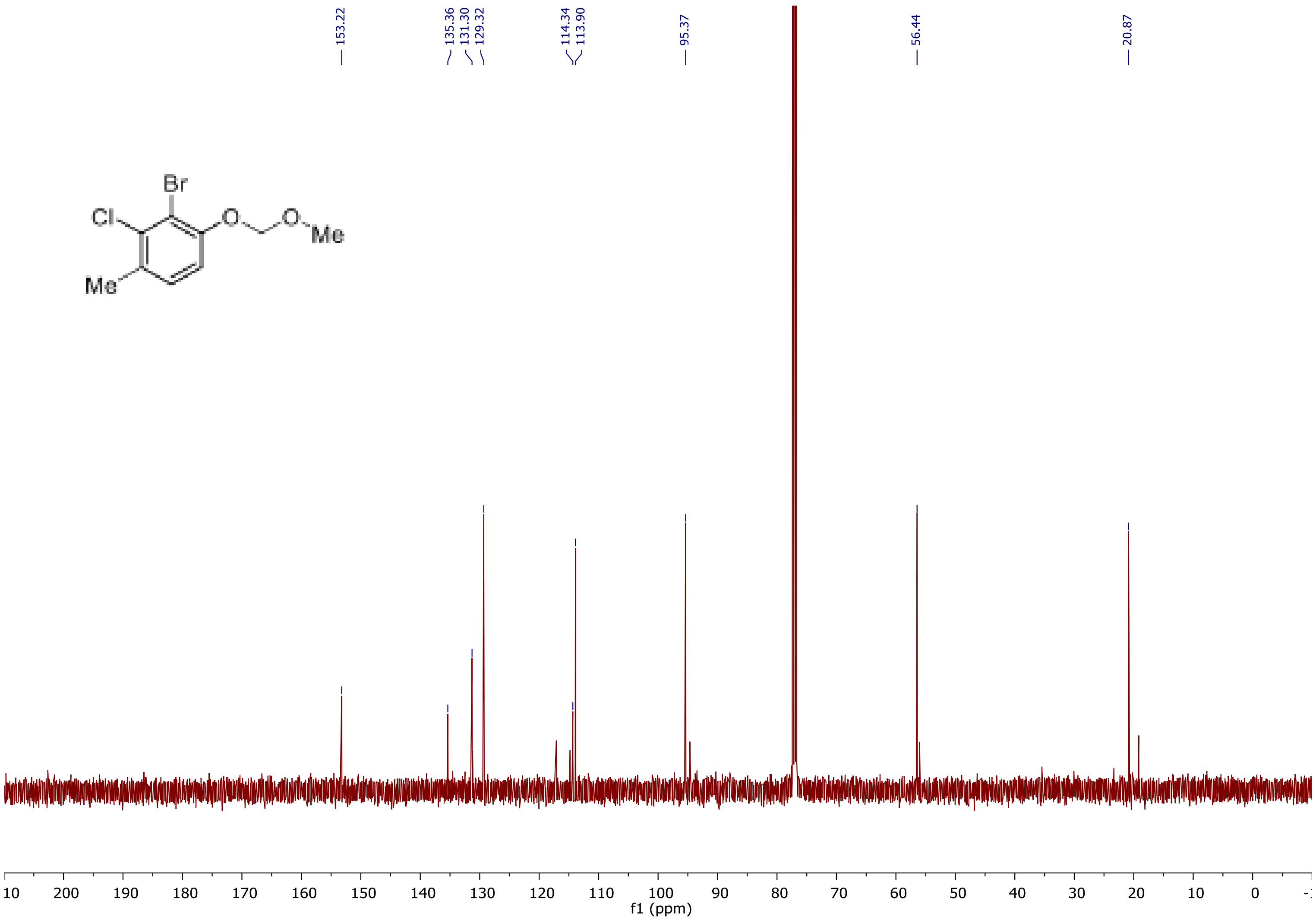

**$^{1}$H NMR** (CDCl$_3$)**:** 2-bromo-3-chloro-4-methylphenol

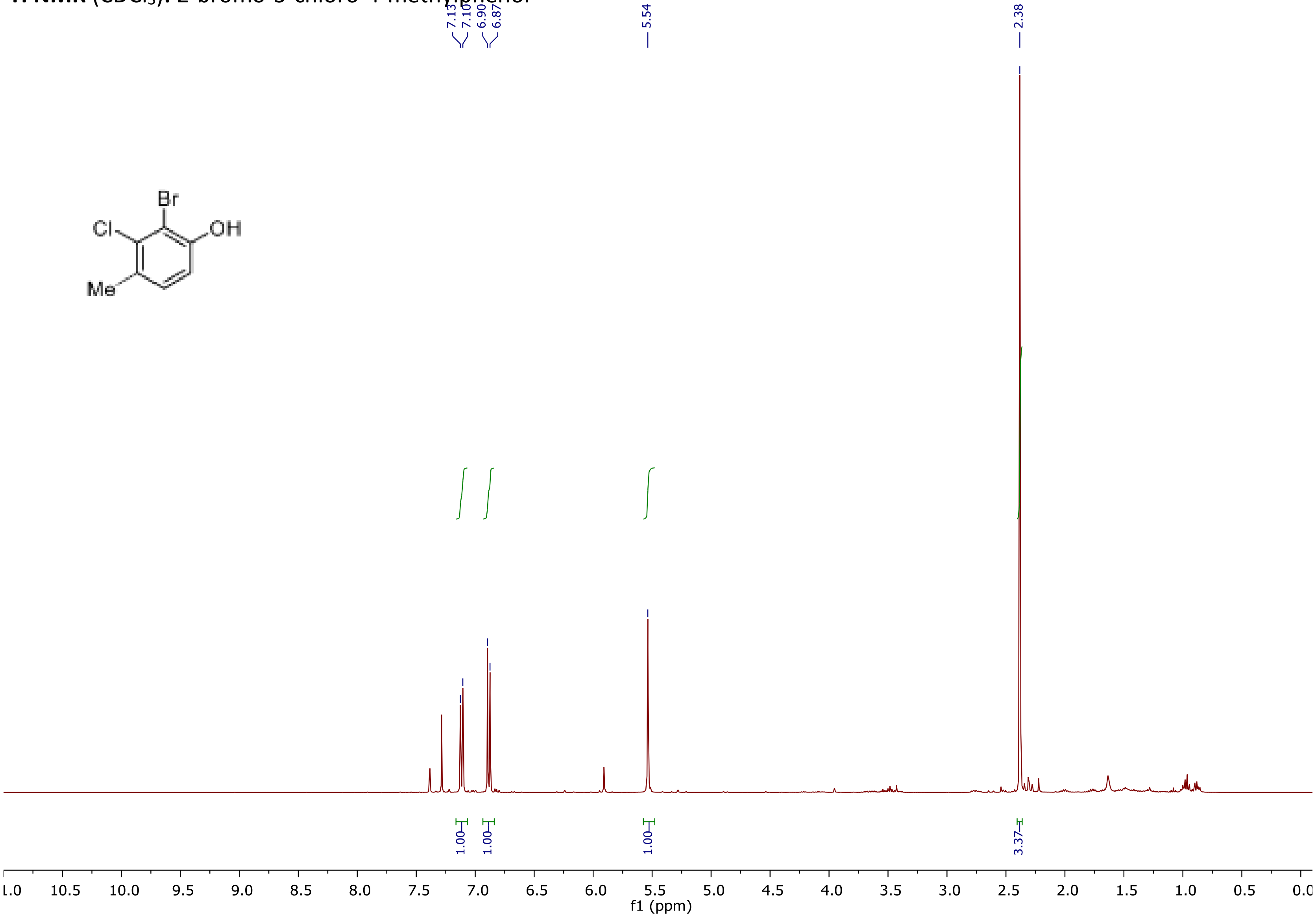

**$^{13}$C NMR** ($CDCl_3$)**:** 2-bromo-3-chloro-4-methylphenol

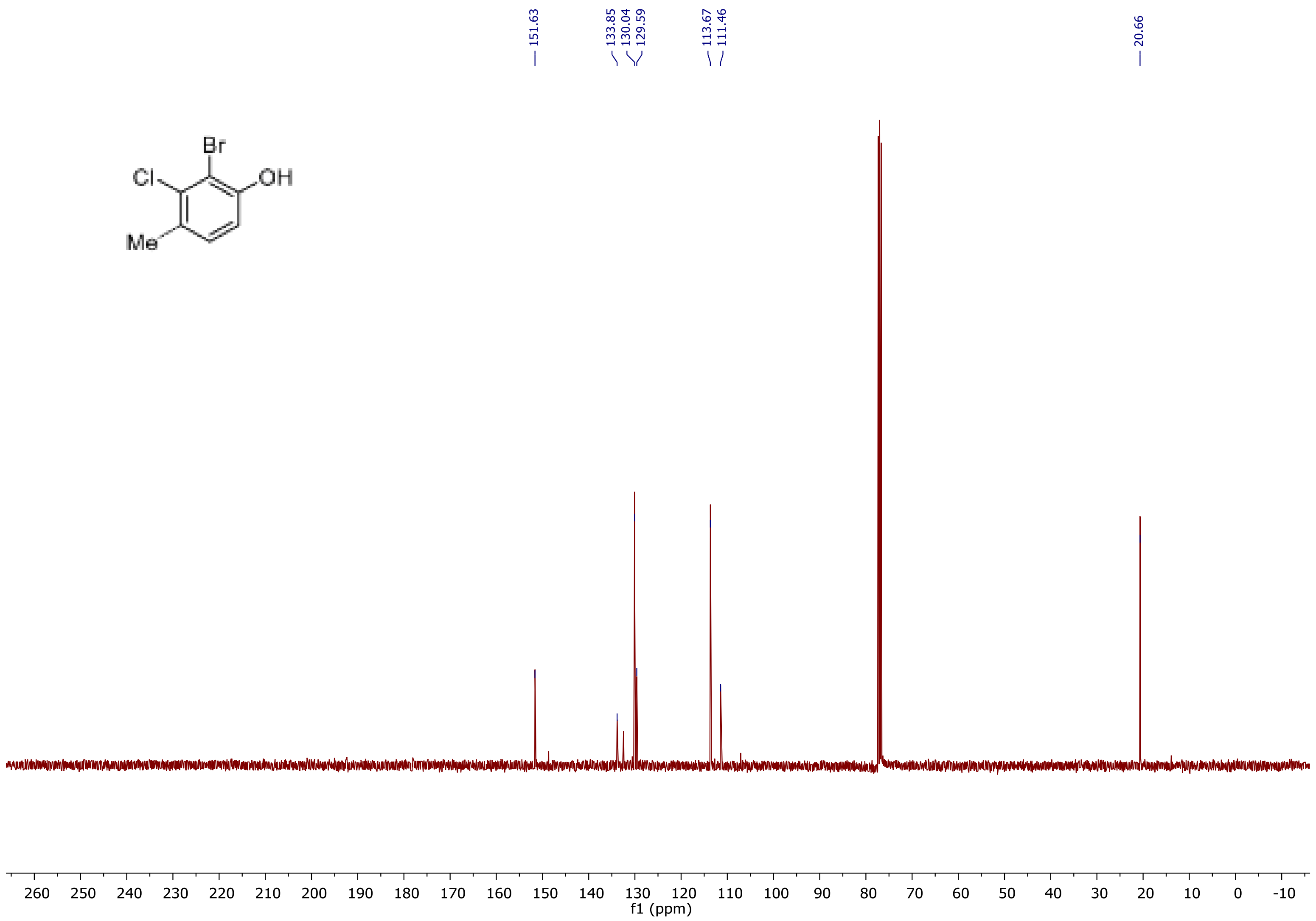

**$^{1}$H NMR** ($CDCl_3$)**:** 2-bromo-3,5-dichlorophenol

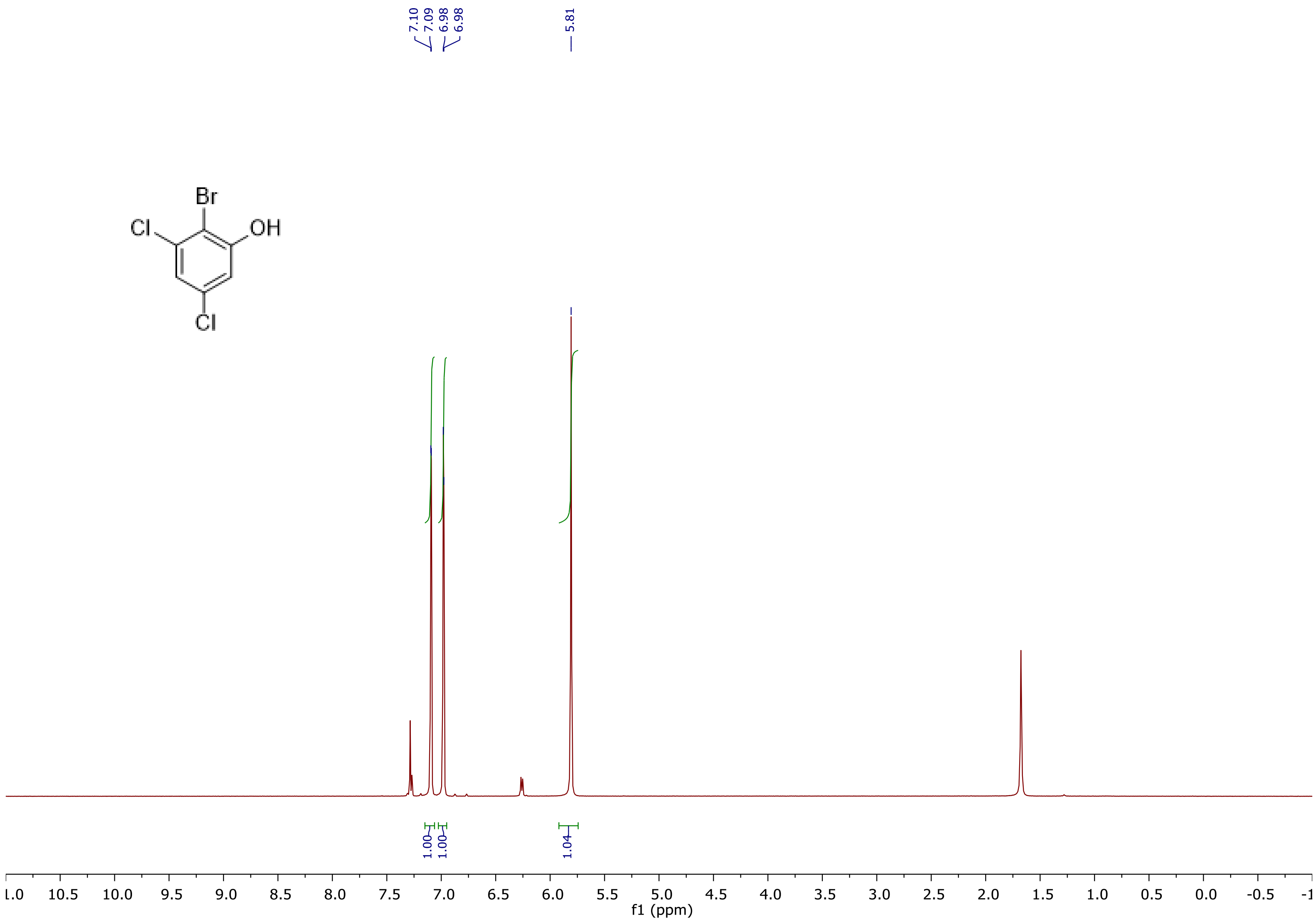

**$^{13}$C NMR** ($CDCl_3$)**:** 2-bromo-3,5-dichlorophenol

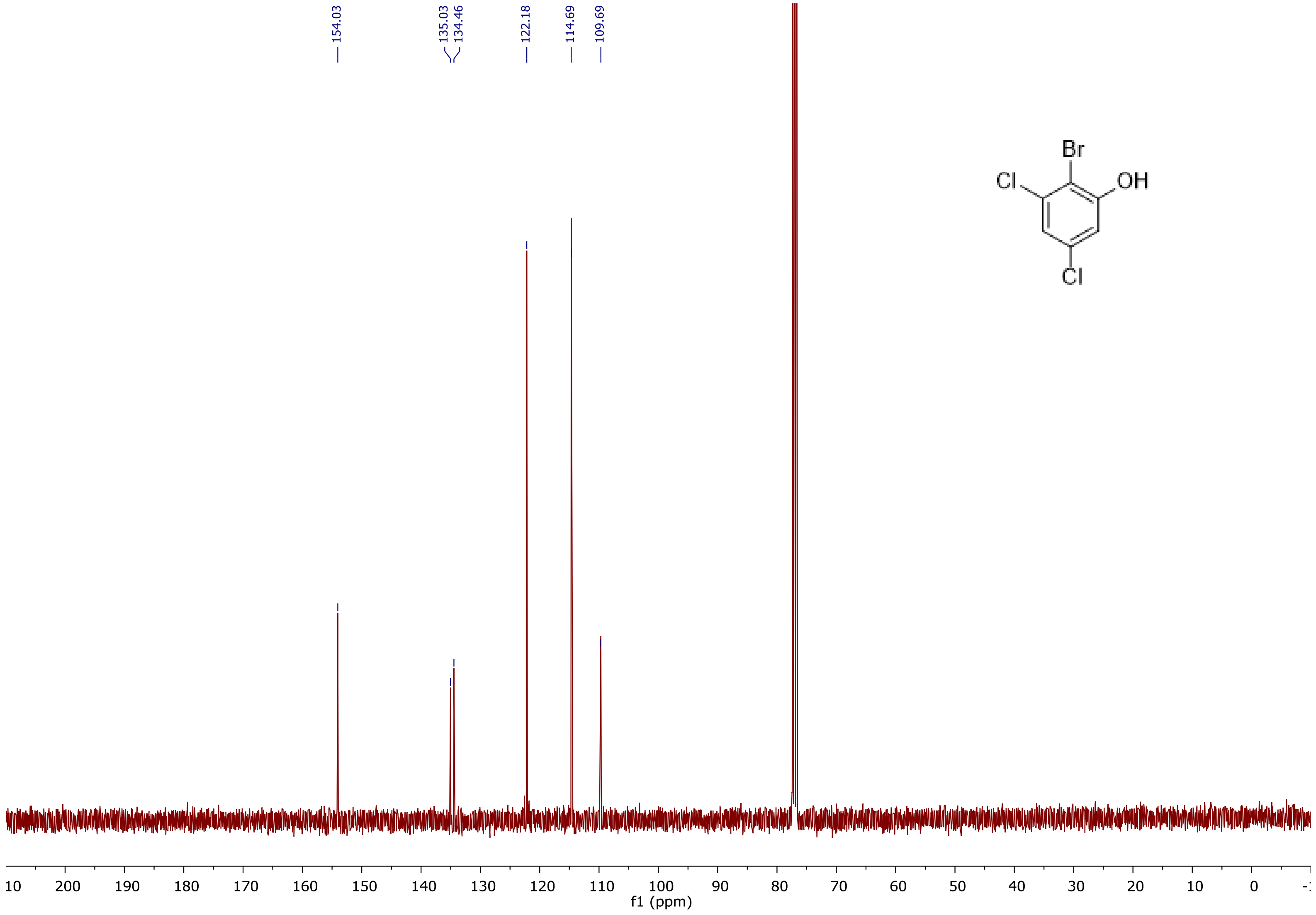

**¹H NMR** ($CDCl_3$): 2-bromo-3,6-dichlorophenol

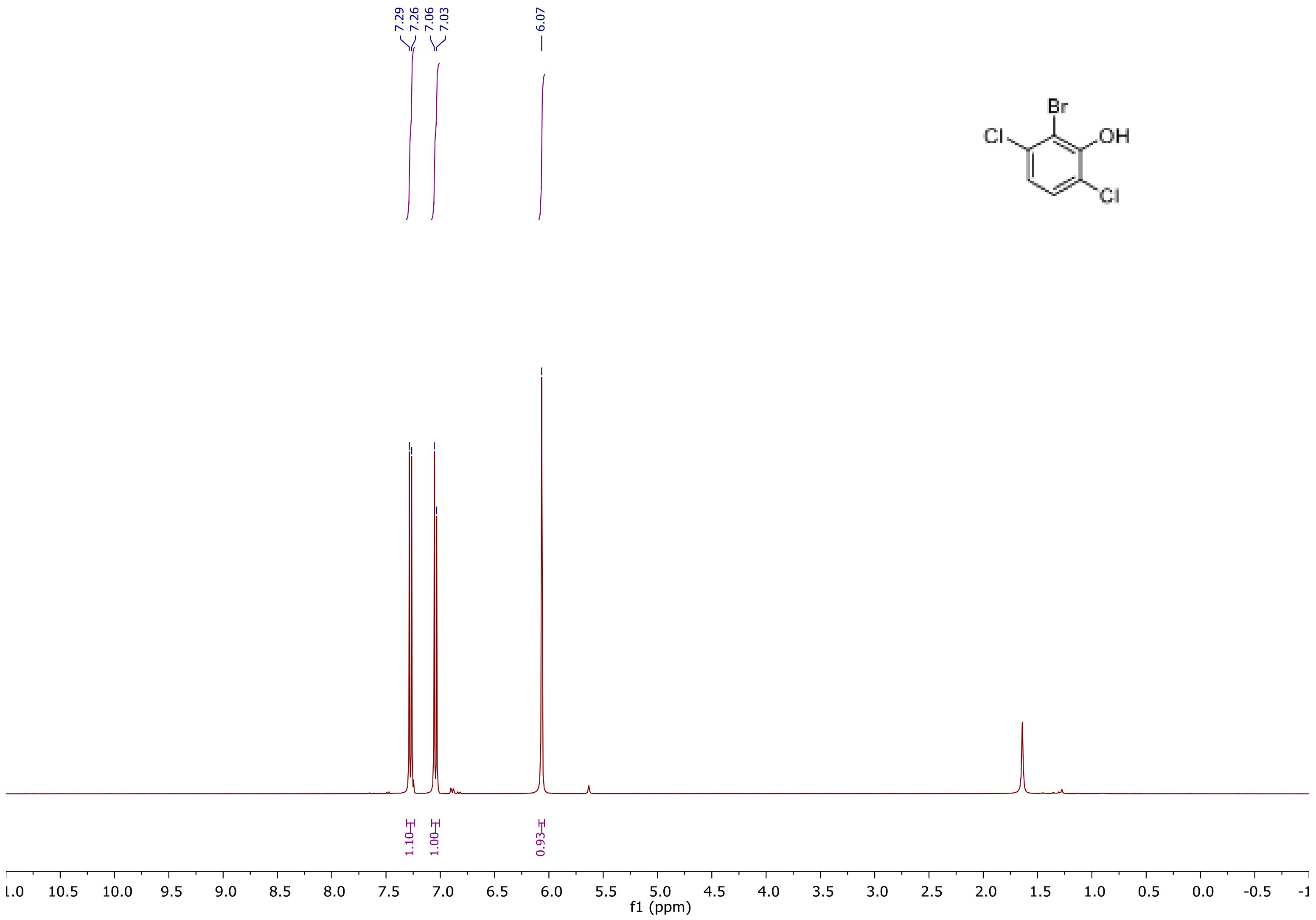

**$^{13}$C NMR** ($CDCl_3$)**:** 2-bromo-3,6-dichlorophenol

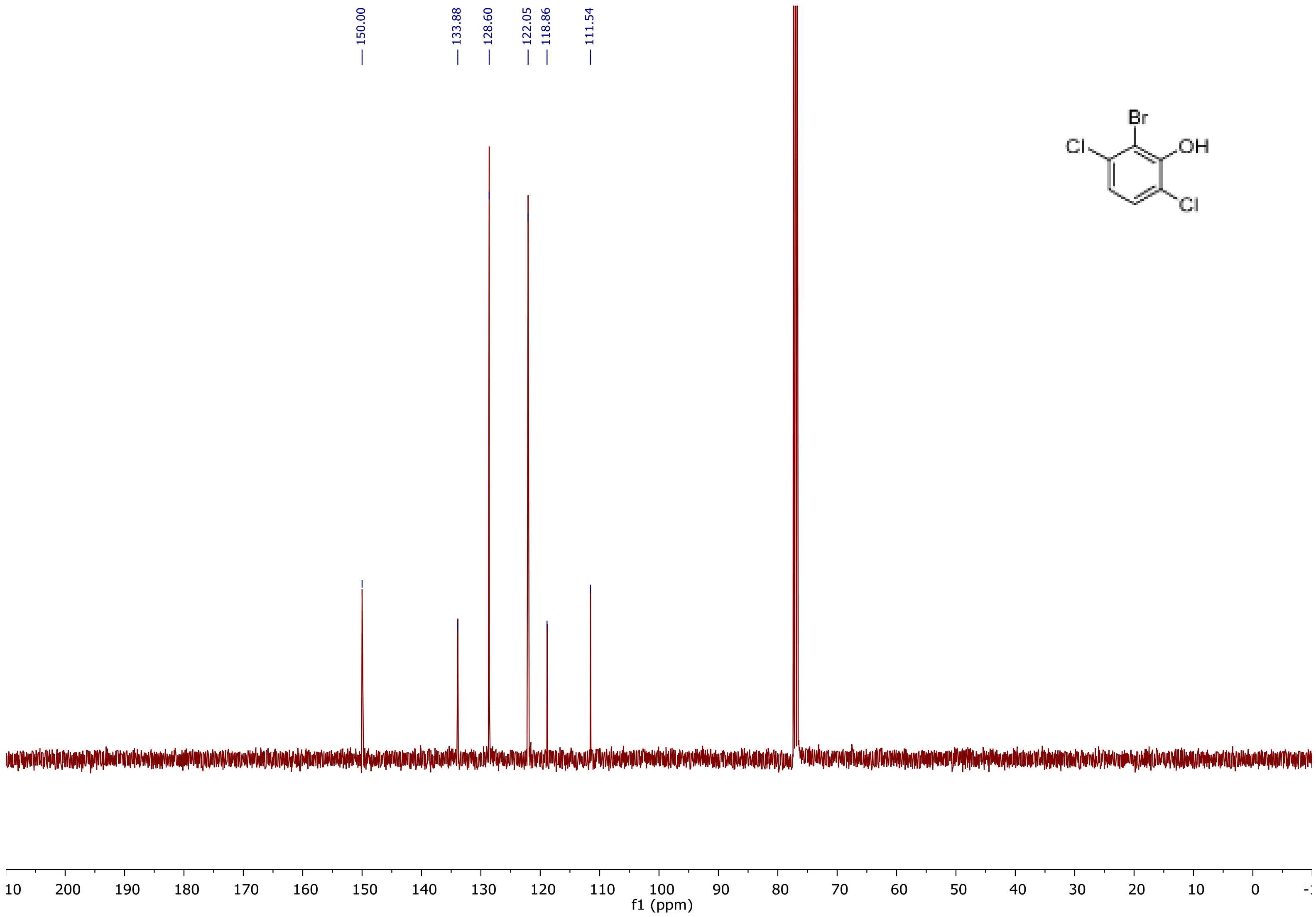

**$^1$H NMR** ($CDCl_3$)**:** tert-butyl (4-chloro-2-hydroxyphenyl)carbamate

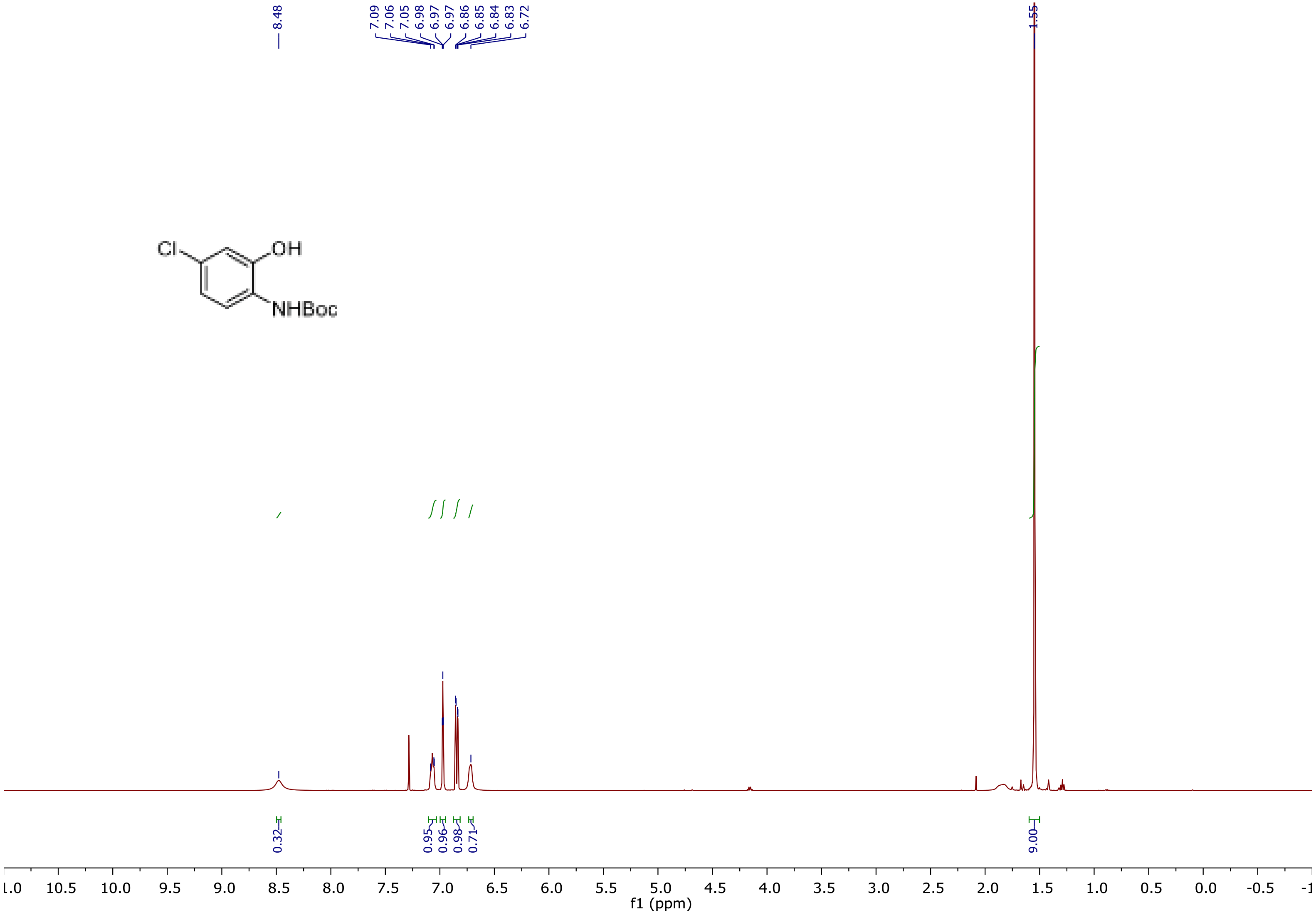

**$^{13}$C NMR** (CDCl$_3$)**:** tert-butyl (4-chloro-2-hydroxyphenyl)carbamate

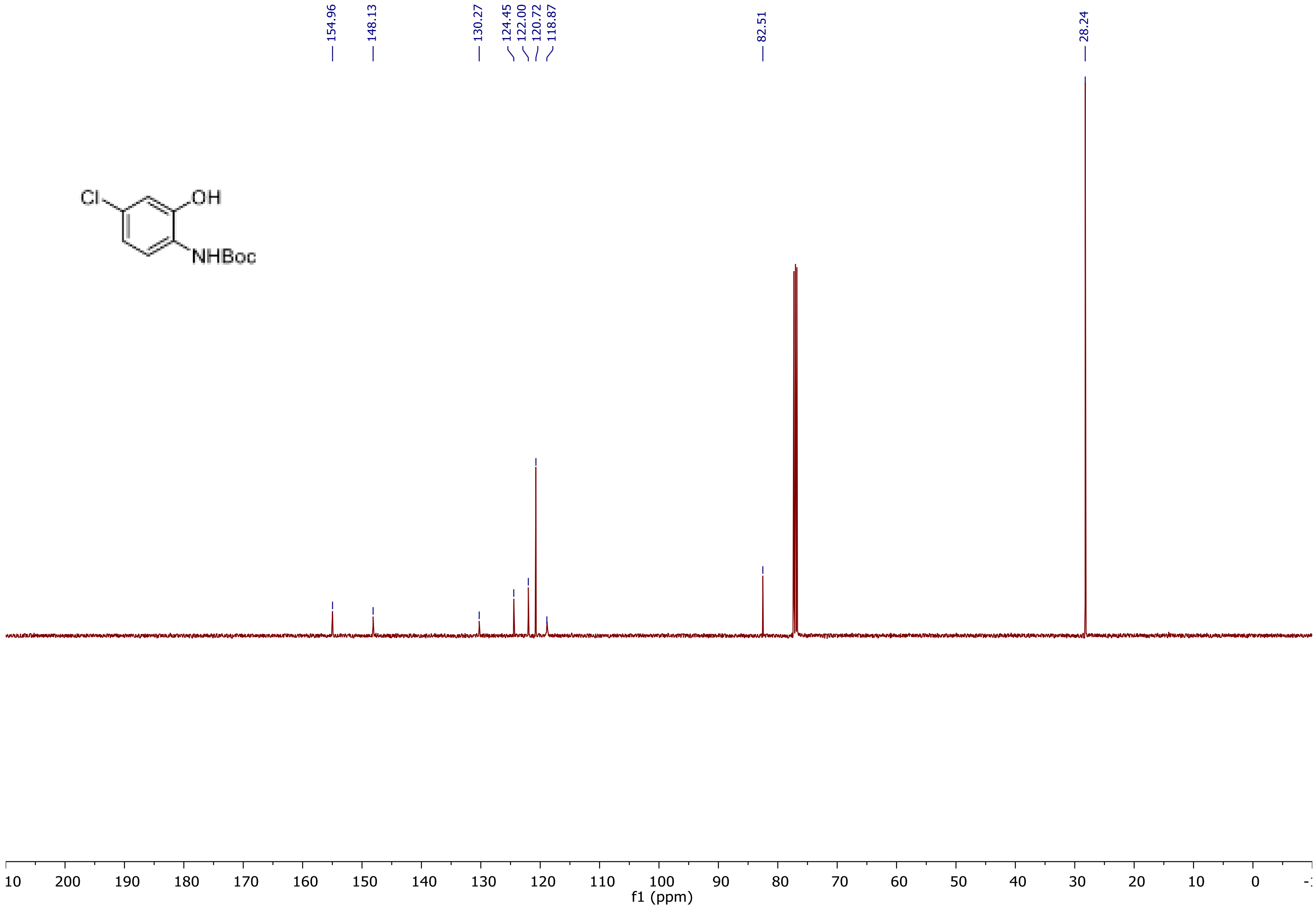

**¹H NMR** (CDCl$_3$)**:** tert-butyl (3-bromo-4-chloro-2-hydroxyphenyl)carbamate

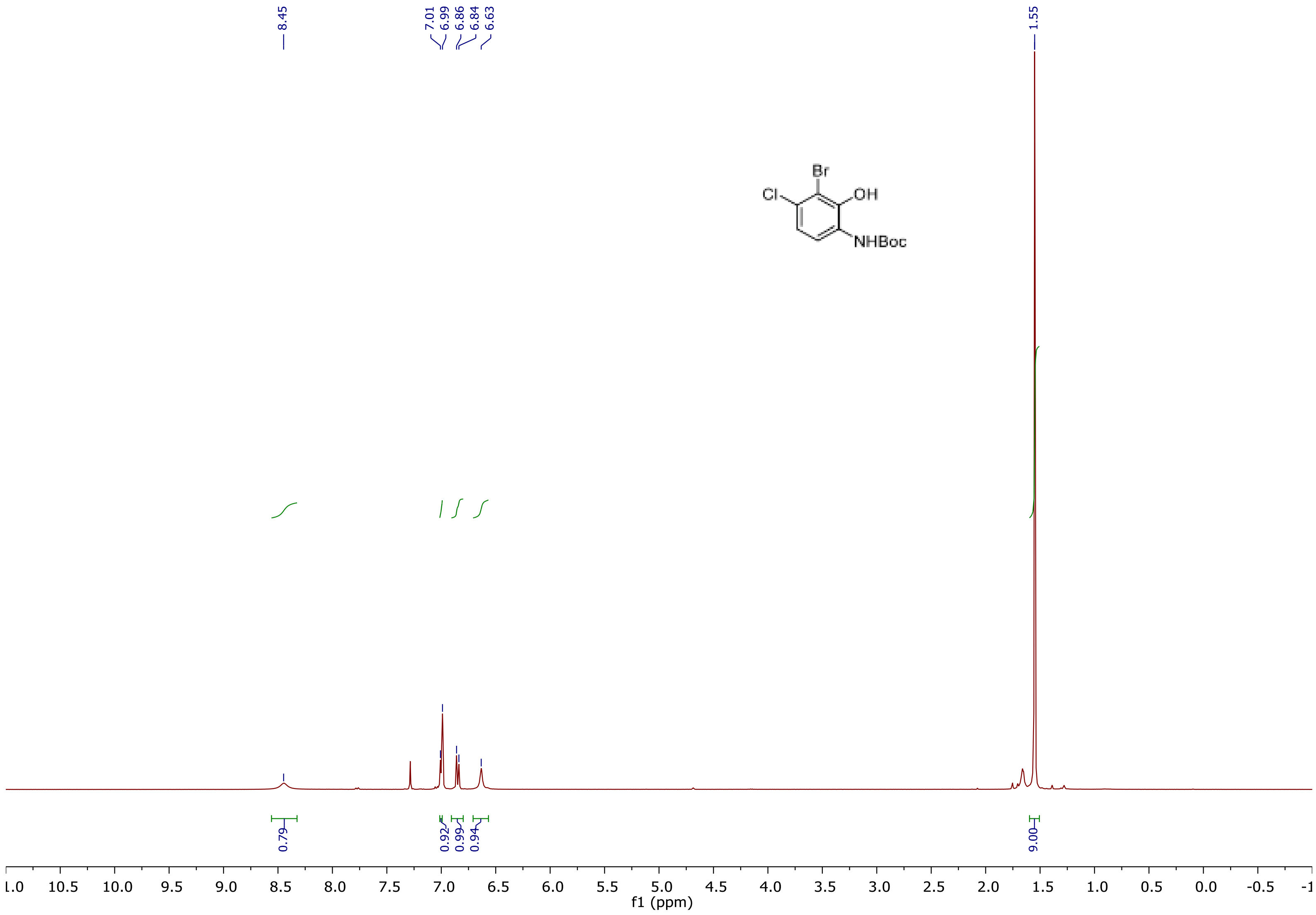

**$^{13}$C NMR** (CDCl$_3$)**:** tert-butyl (3-bromo-4-chloro-2-hydroxyphenyl)carbamate

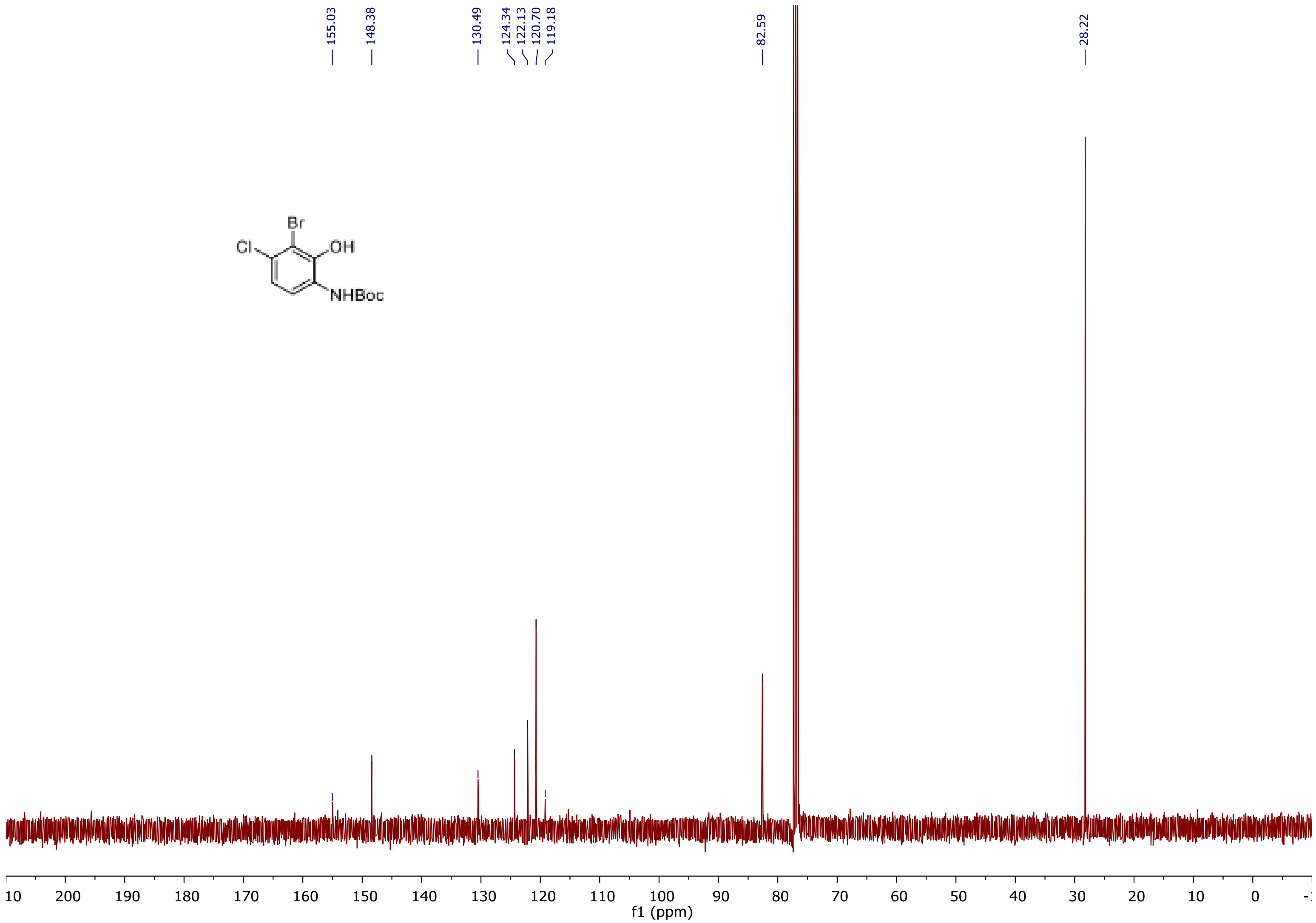

**$^{1}$H NMR** ($CDCl_3$)**:** 5-chloro-2-methoxyphenol

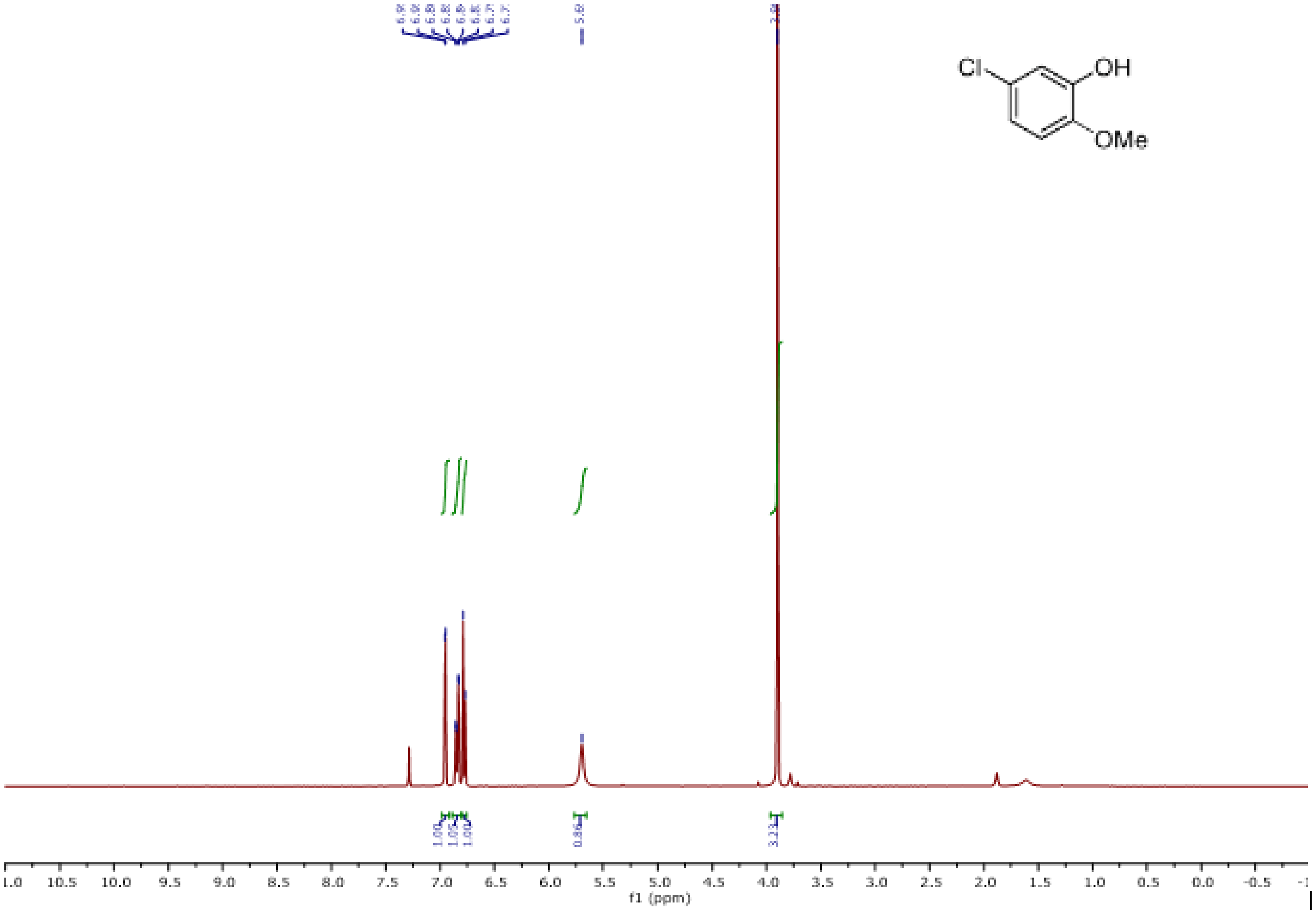

$^{13}$**C NMR** ($CDCl_3$) **:** 5-chloro-2-methoxyphenol

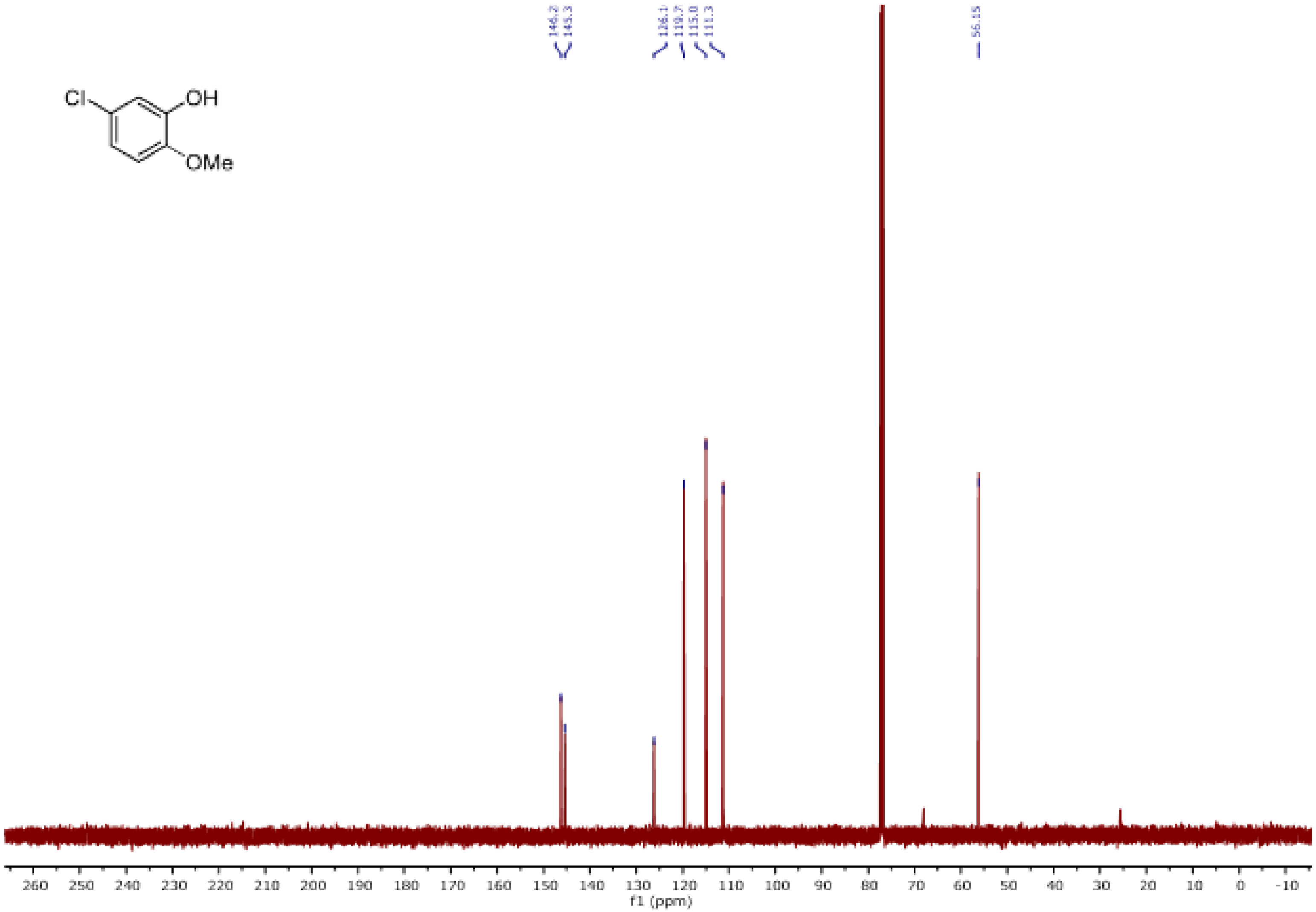

**$^1$H NMR** ($CDCl_3$)**:** 2-bromo-3-chloro-6-methoxyphenol

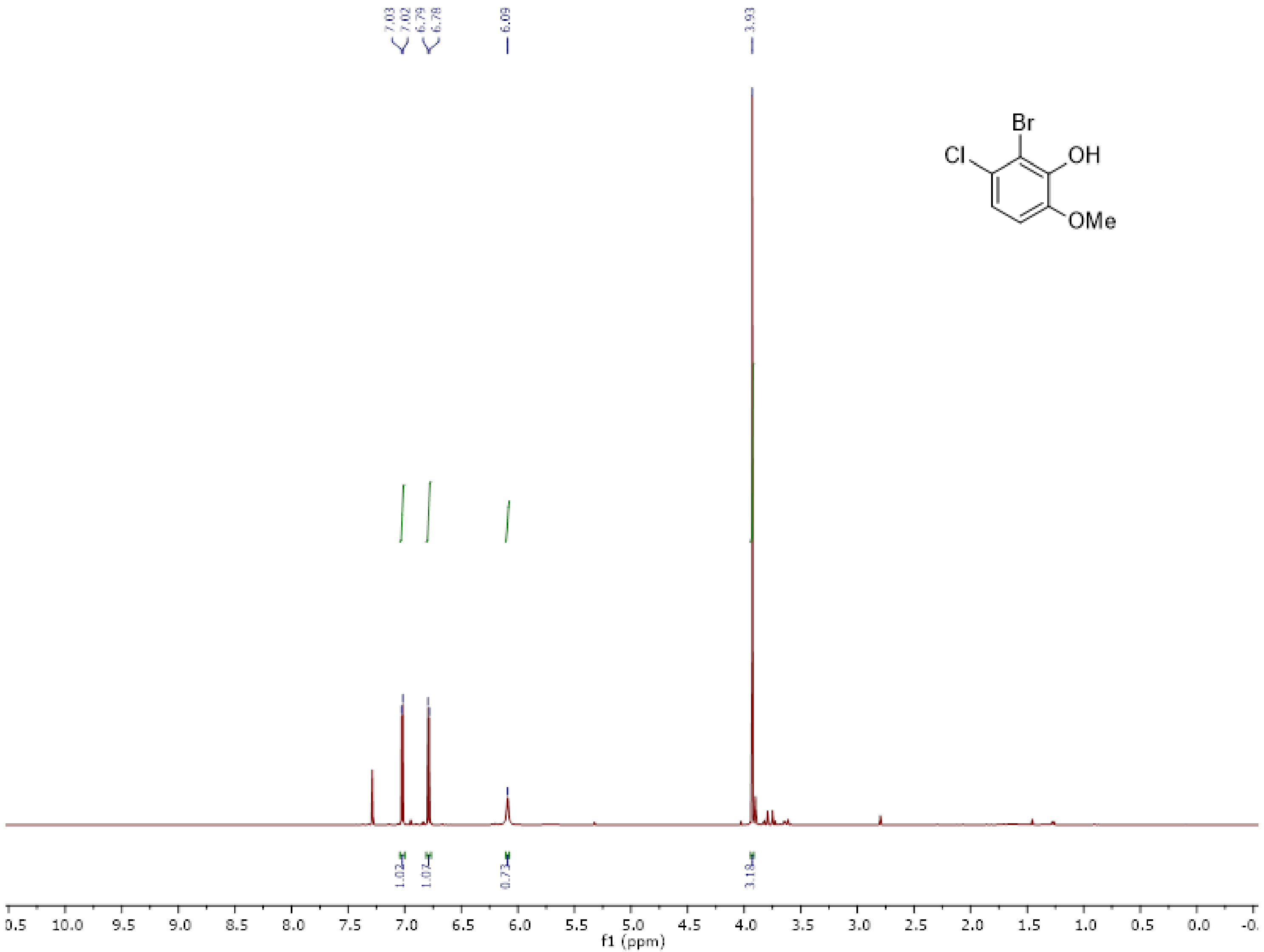

**$^{13}$C NMR** (CDCl$_3$)**:** 2-bromo-3-chloro-6-methoxyphenol

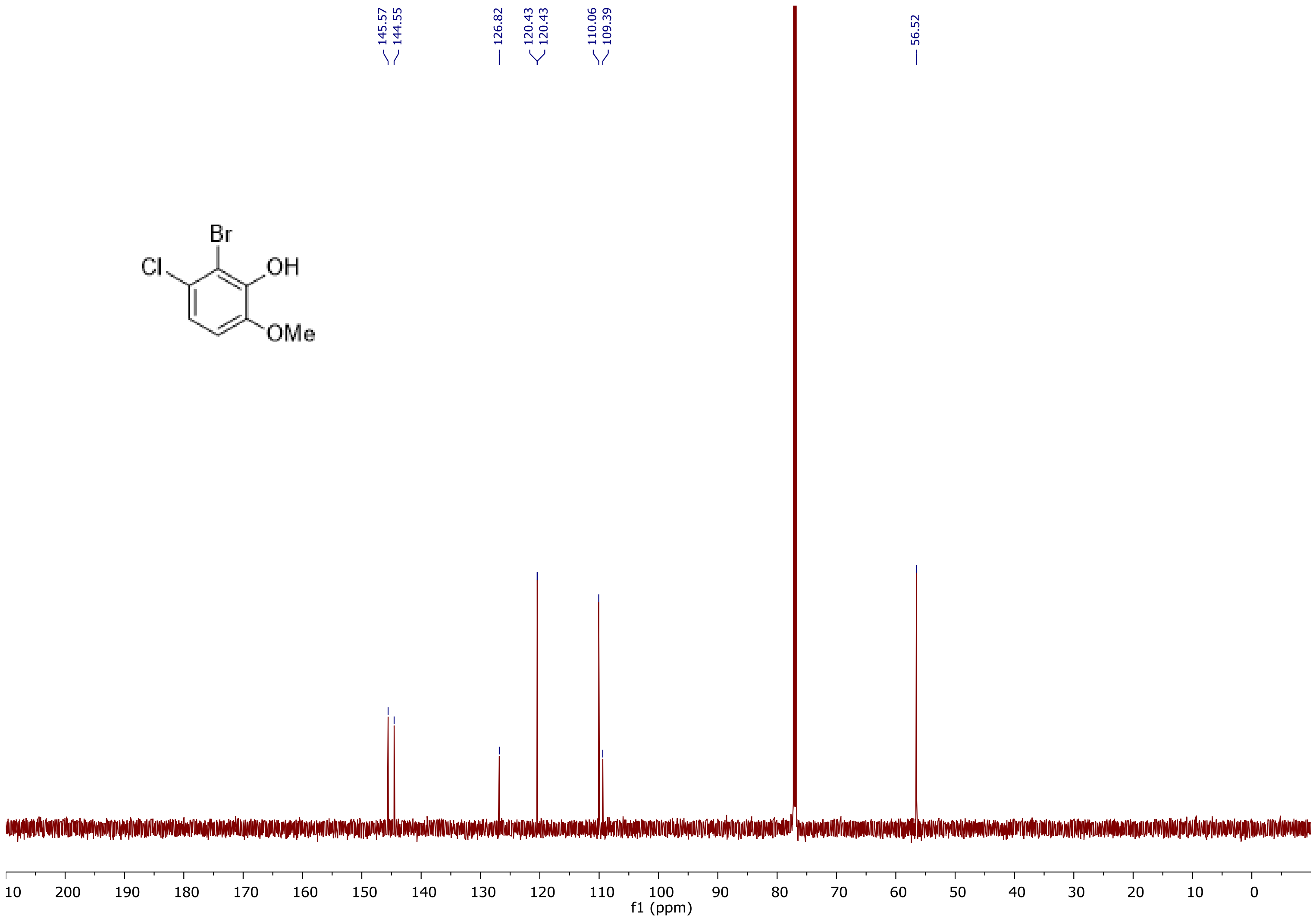

$^1$**H NMR** ($CDCl_3$): 2-bromo-3-(2-nitroethyl)phenol

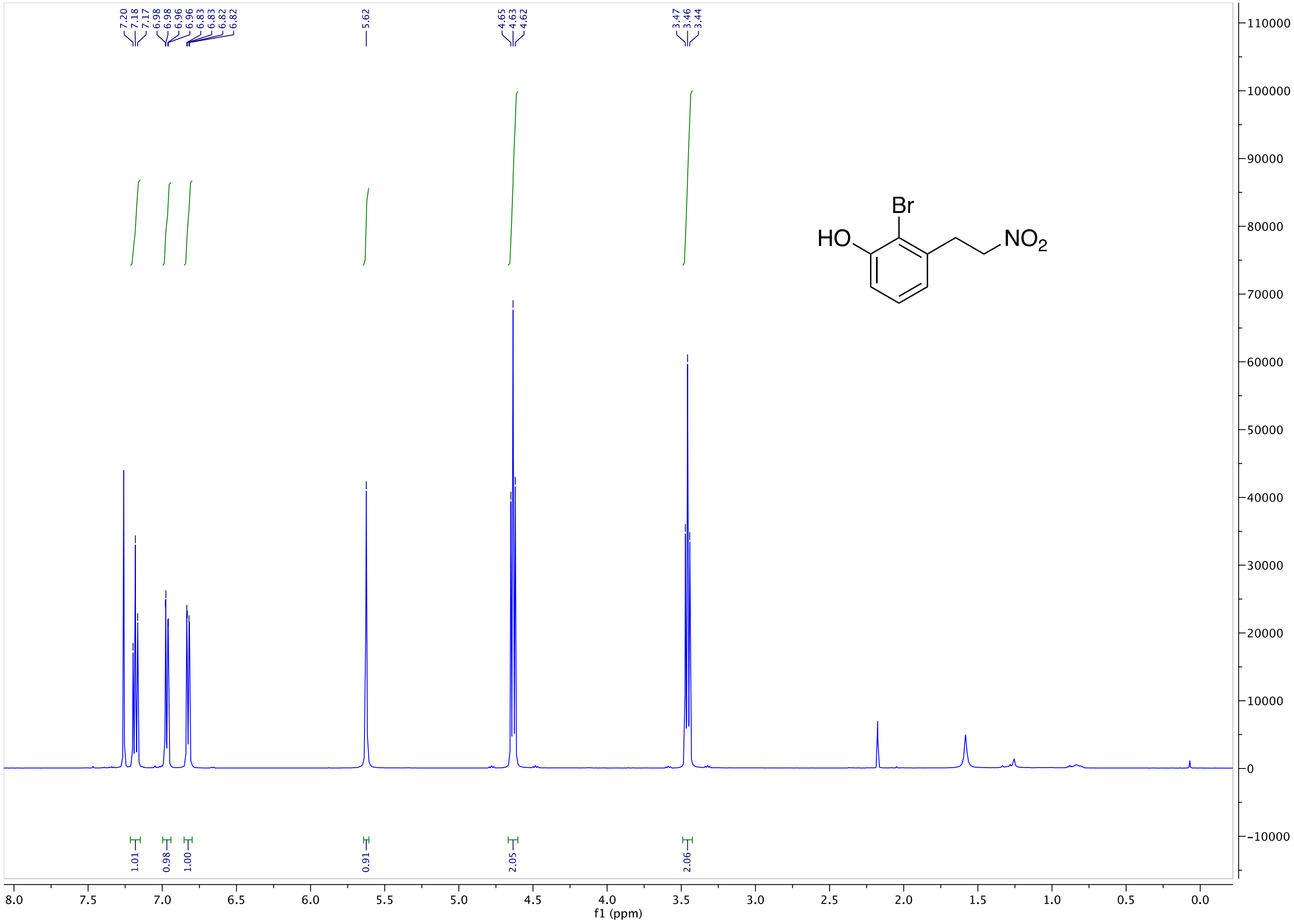

**$^{13}$C NMR** ($CDCl_3$): 2-bromo-3-(2-nitroethyl)phenol

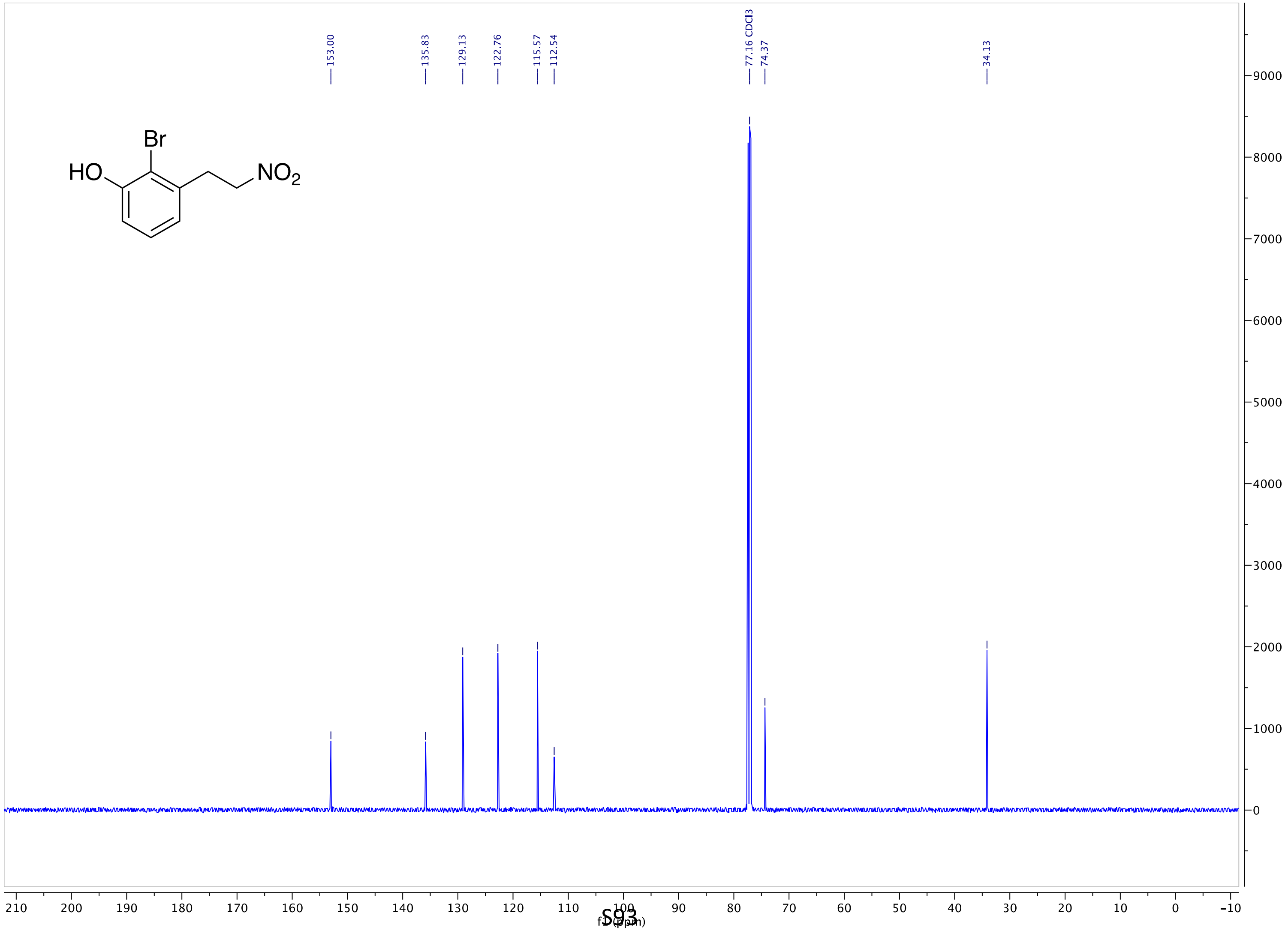

**¹H NMR** ($CDCl_3$): 2-bromo-3-hydroxy-4-methoxybenzaldehyde

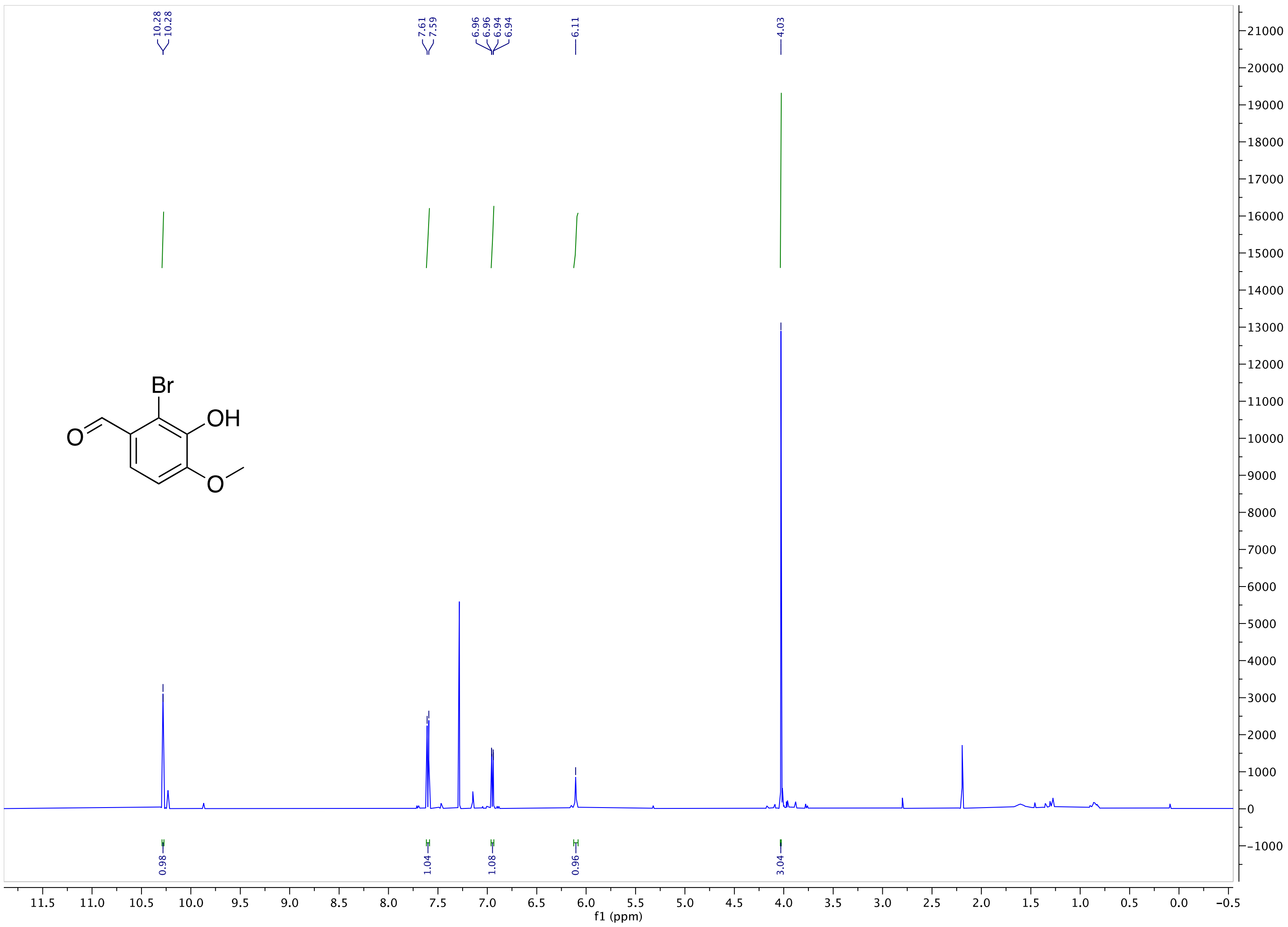

**$^{13}$C NMR** ($CDCl_3$) 2-bromo-3-hydroxy-4-methoxybenzaldehyde

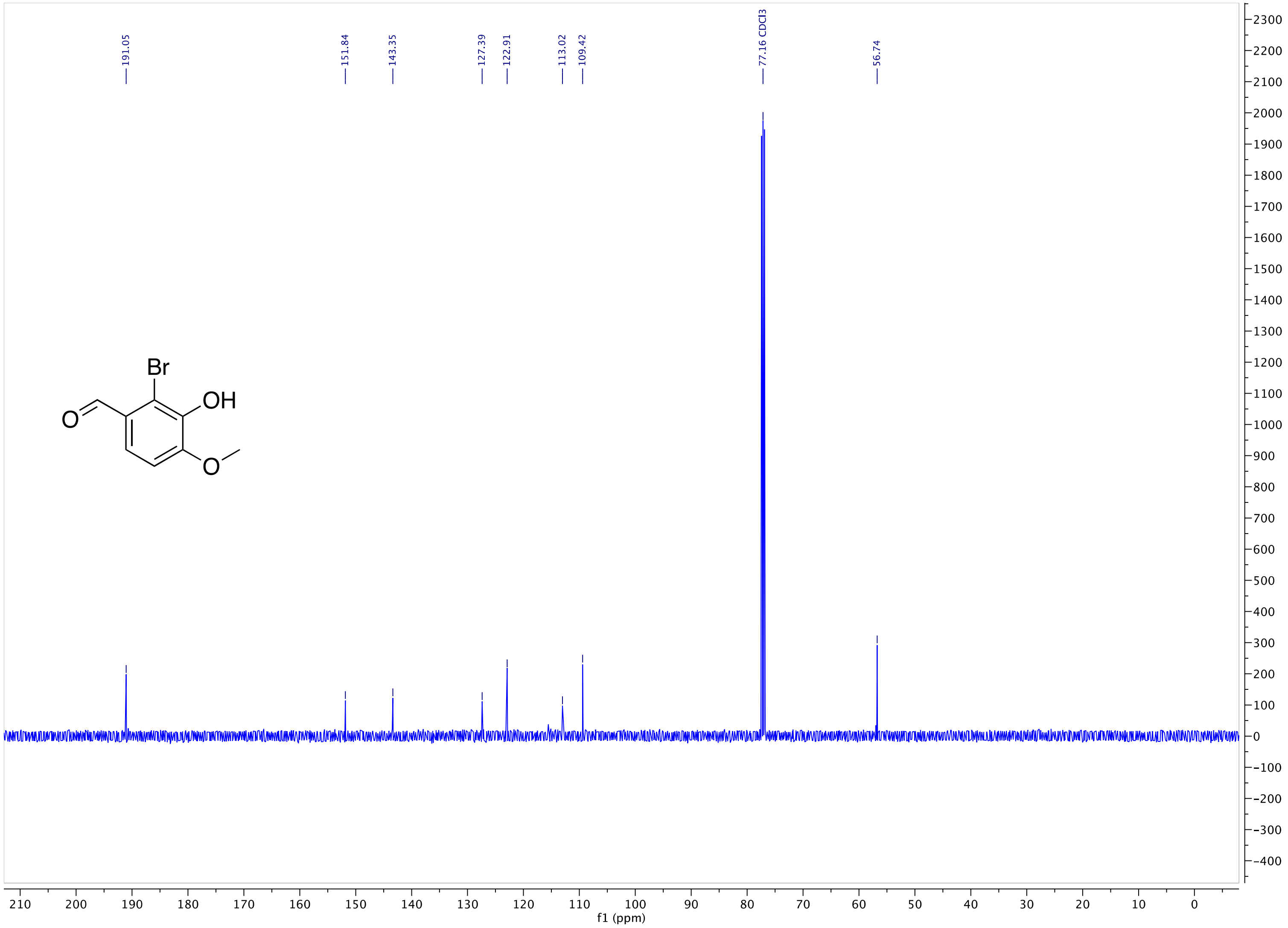

**$^1$H NMR** ($CDCl_3$): 2-bromo-3-(((tert-butyldimethylsilyl)oxy)methyl)-6-methoxyphenol

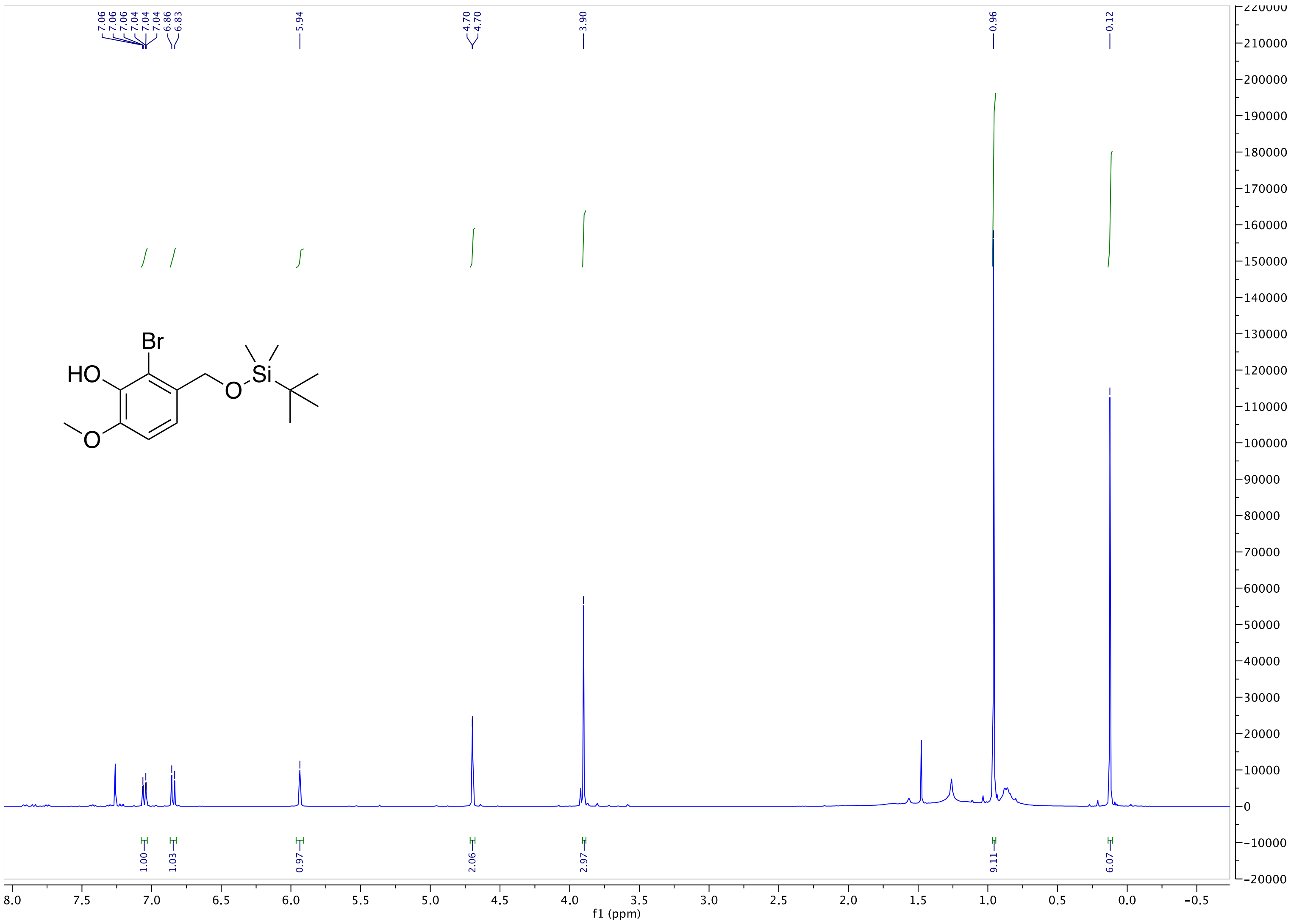

**$^{13}$C NMR** ($CDCl_3$): 2-bromo-3-(((tert-butyldimethylsilyl)oxy)methyl)-6-methoxyphenol

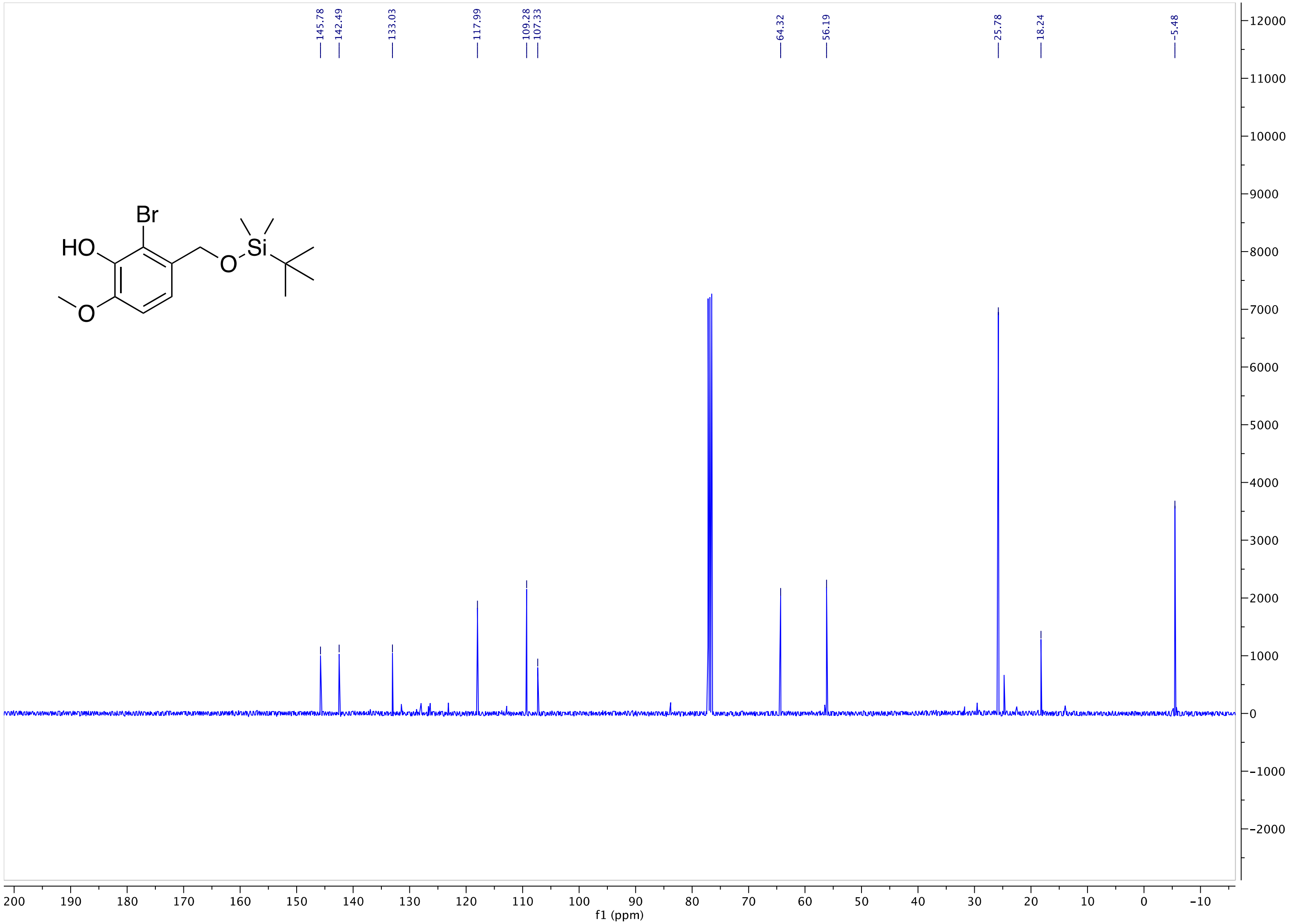

**$^{1}$H NMR** ($CDCl_3$): *tert*-butyl (2-bromo-3-hydroxy-4-methoxybenzyl)carbamate

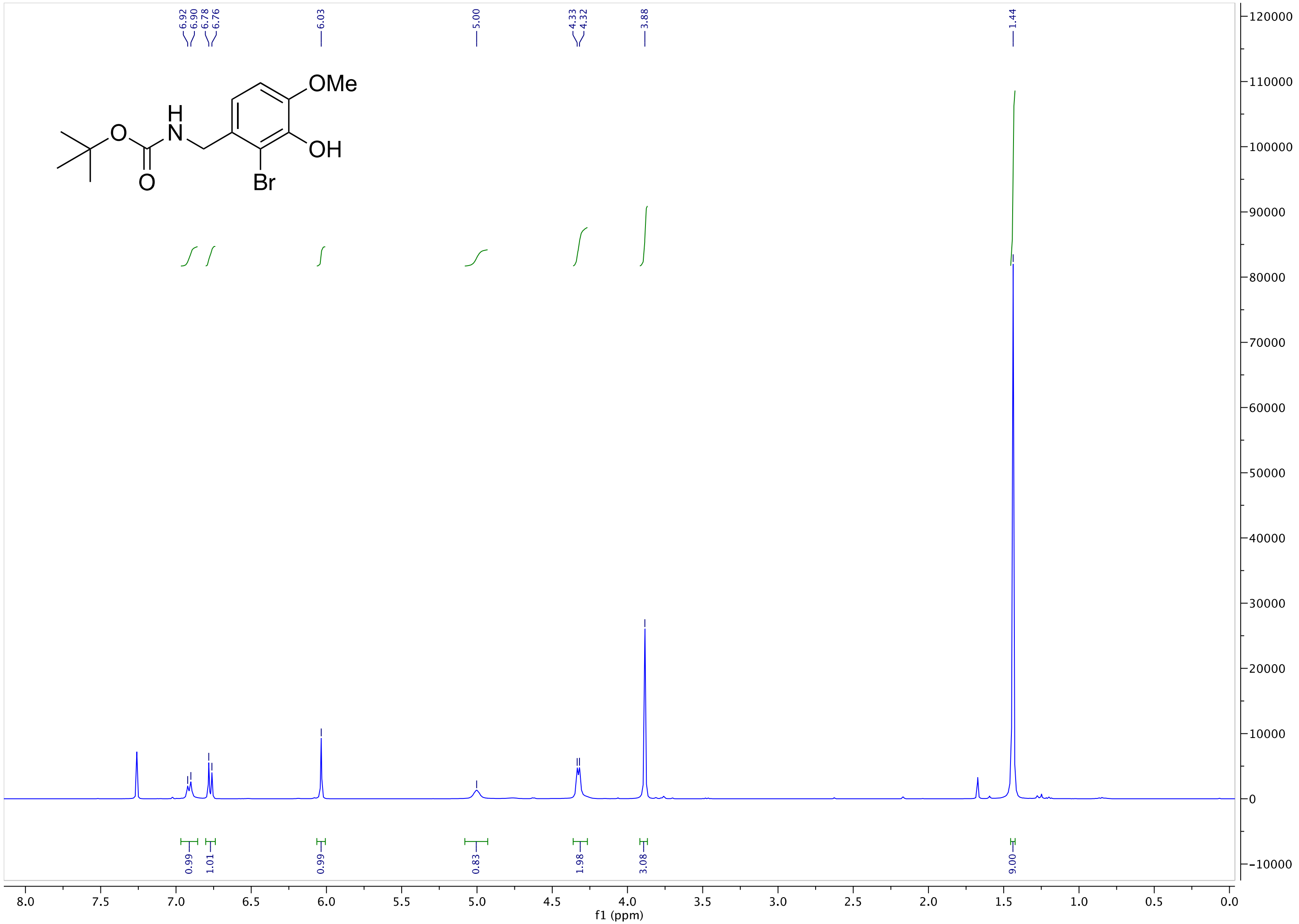

**$^{13}$C NMR** ($CDCl_3$): *tert*-butyl (2-bromo-3-hydroxy-4-methoxybenzyl)carbamate

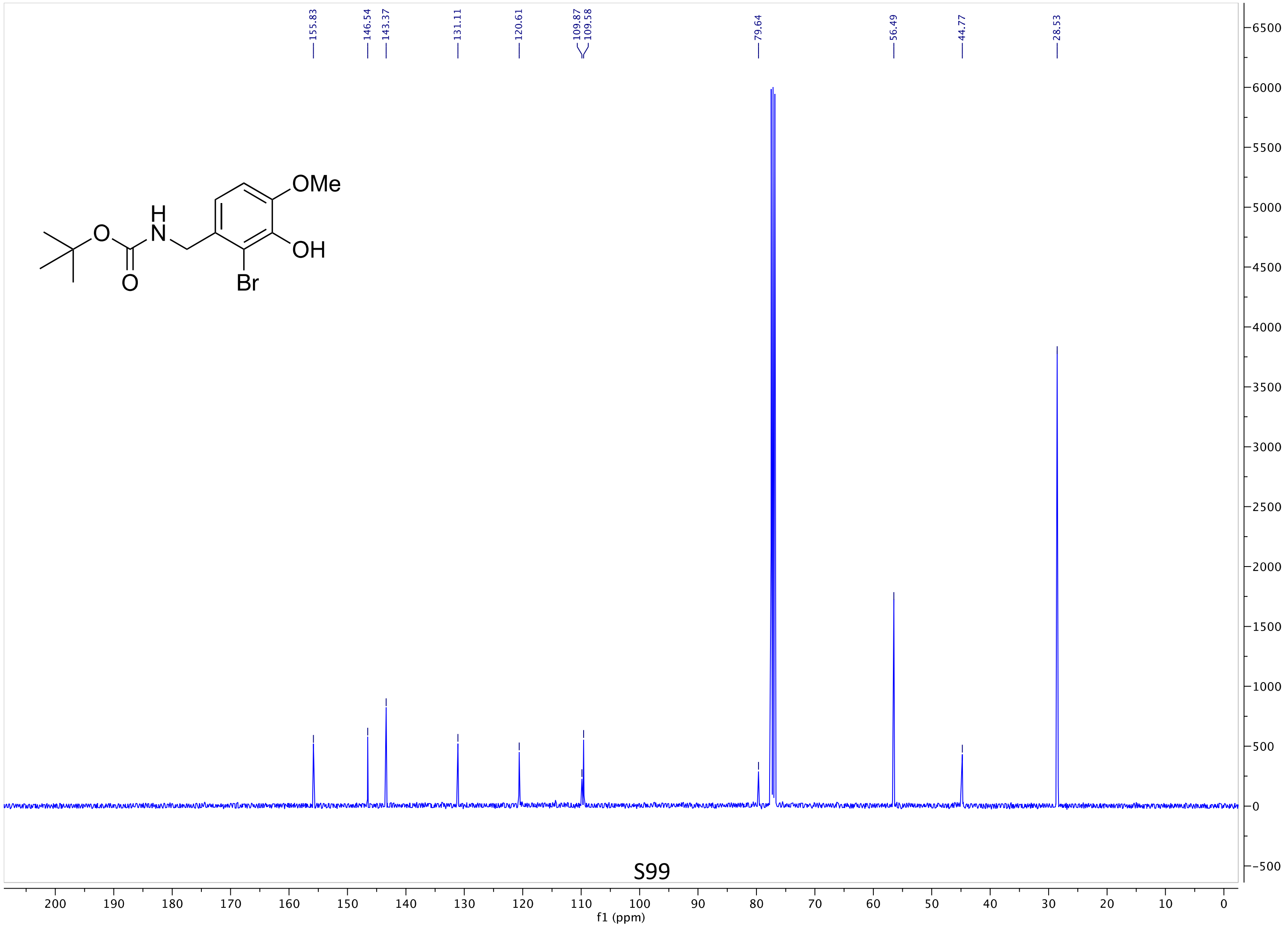

**¹H NMR** (CDCl$_3$)**:** 2-bromo-3-chloro-6-(2,4-dichlorophenoxy)phenol

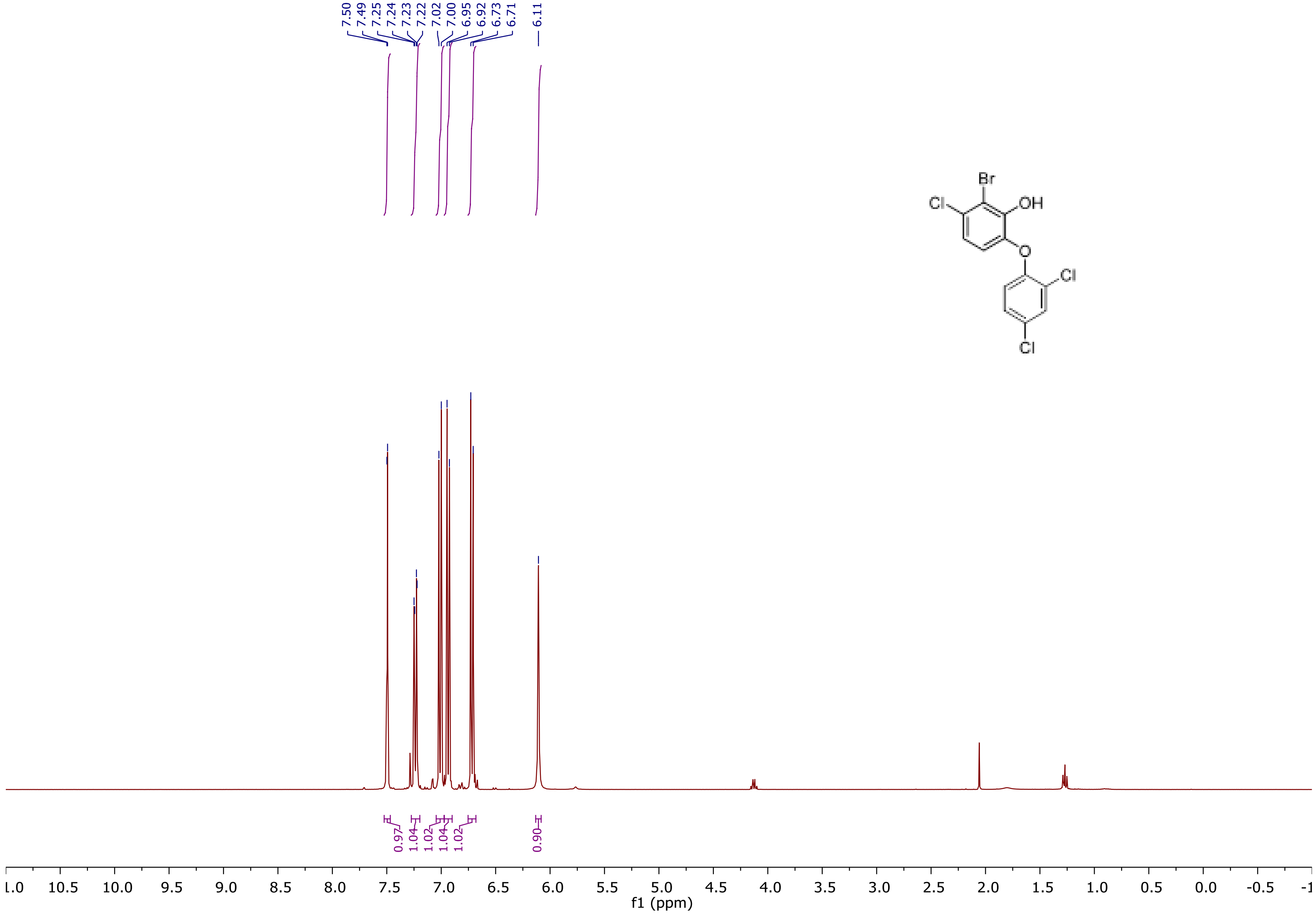

**$^{13}$C NMR** ($CDCl_3$)**:** 2-bromo-3-chloro-6-(2,4-dichlorophenoxy)phenol

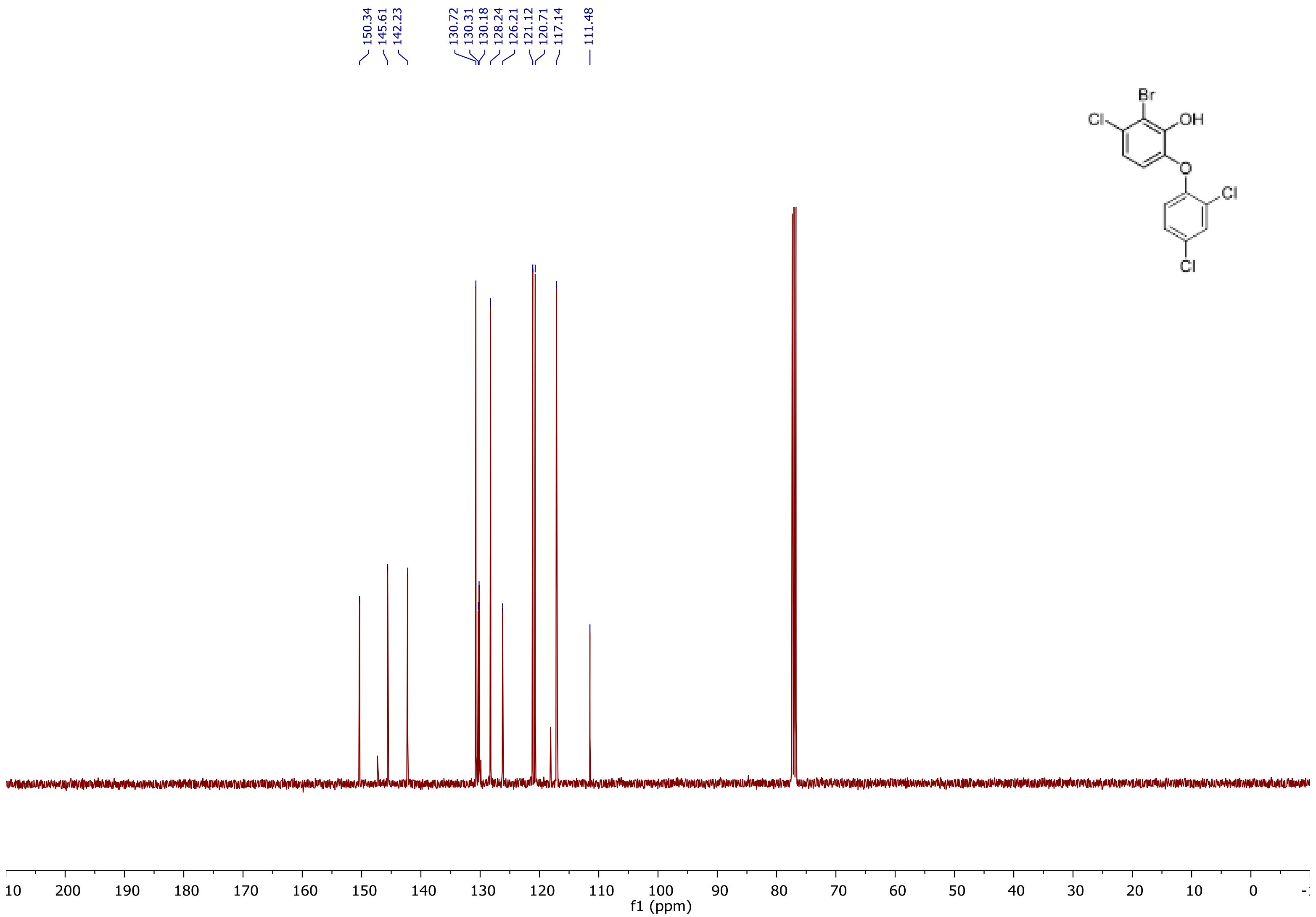

**$^1$H NMR** (CDCl$_3$)**:** (8*R*,9*S*,13*S*,14*S*)-4-bromo-3-hydroxy-13-methyl-6,7,8,9,11,12,13,14,15,16-decahydro-17H-cyclopenta[a]phenanthren-17-one

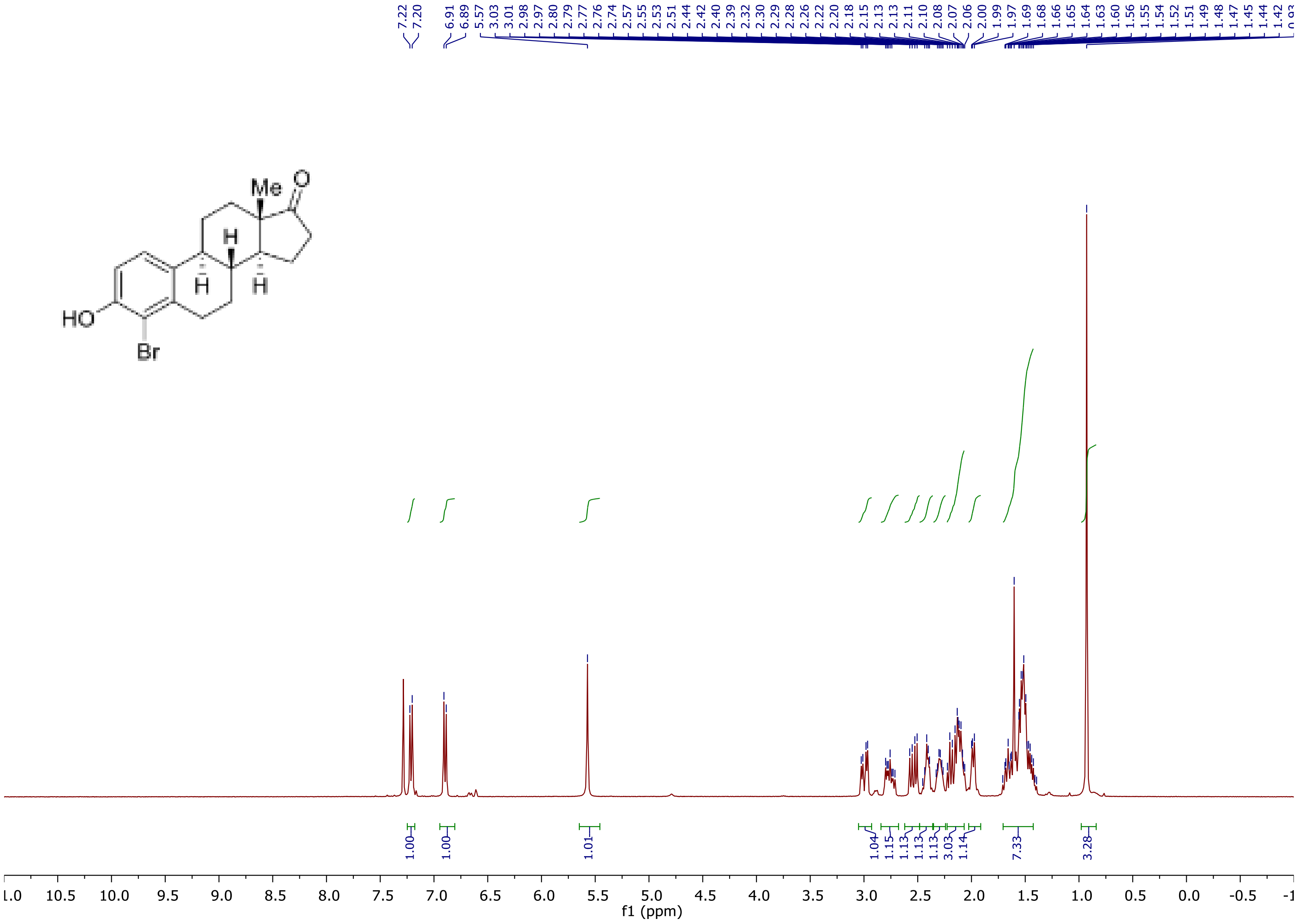

**$^{13}$C NMR** ($CDCl_3$)**:** (8*R*,9*S*,13*S*,14*S*)-4-bromo-3-hydroxy-13-methyl-6,7,8,9,11,12,13,14,15,16-decahydro-17H-cyclopenta[a]phenanthren-17-one

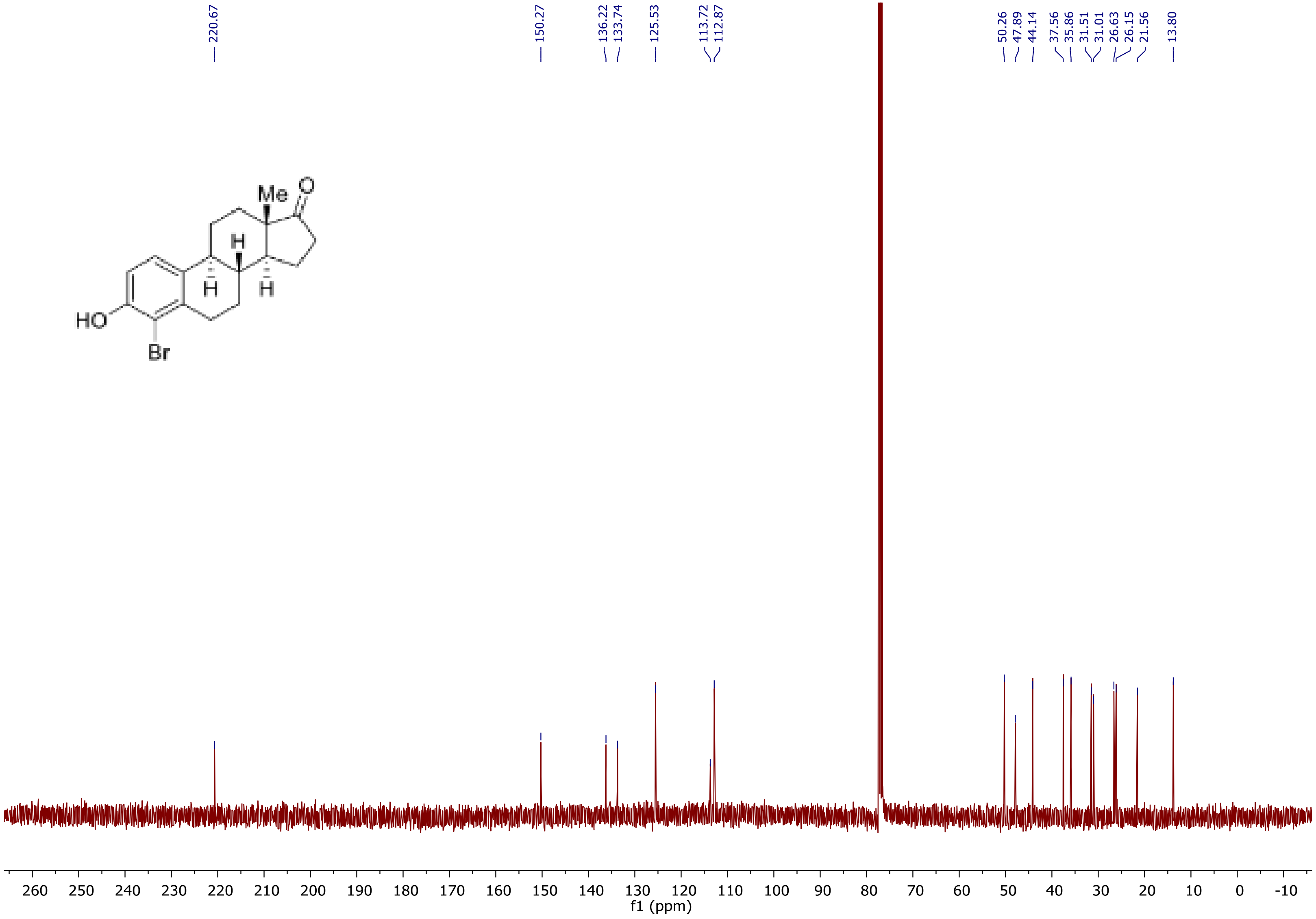

**$^{1}$H NMR** ($CDCl_3$): 2-bromo-3-methoxyphenol

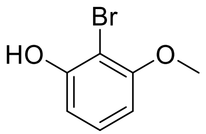


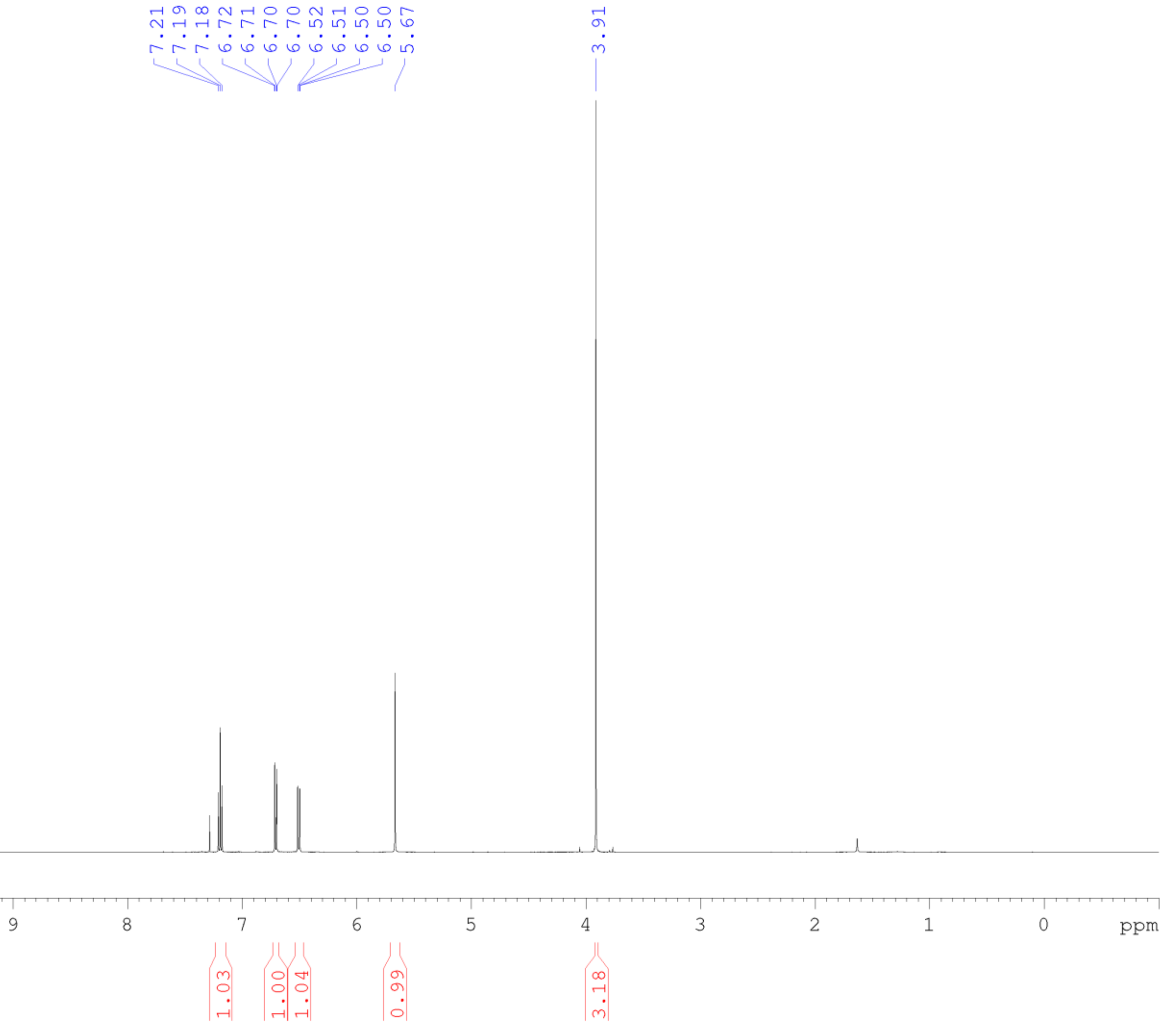

**$^{13}$C NMR** ($CDCl_3$): 2-bromo-3-methoxyphenol

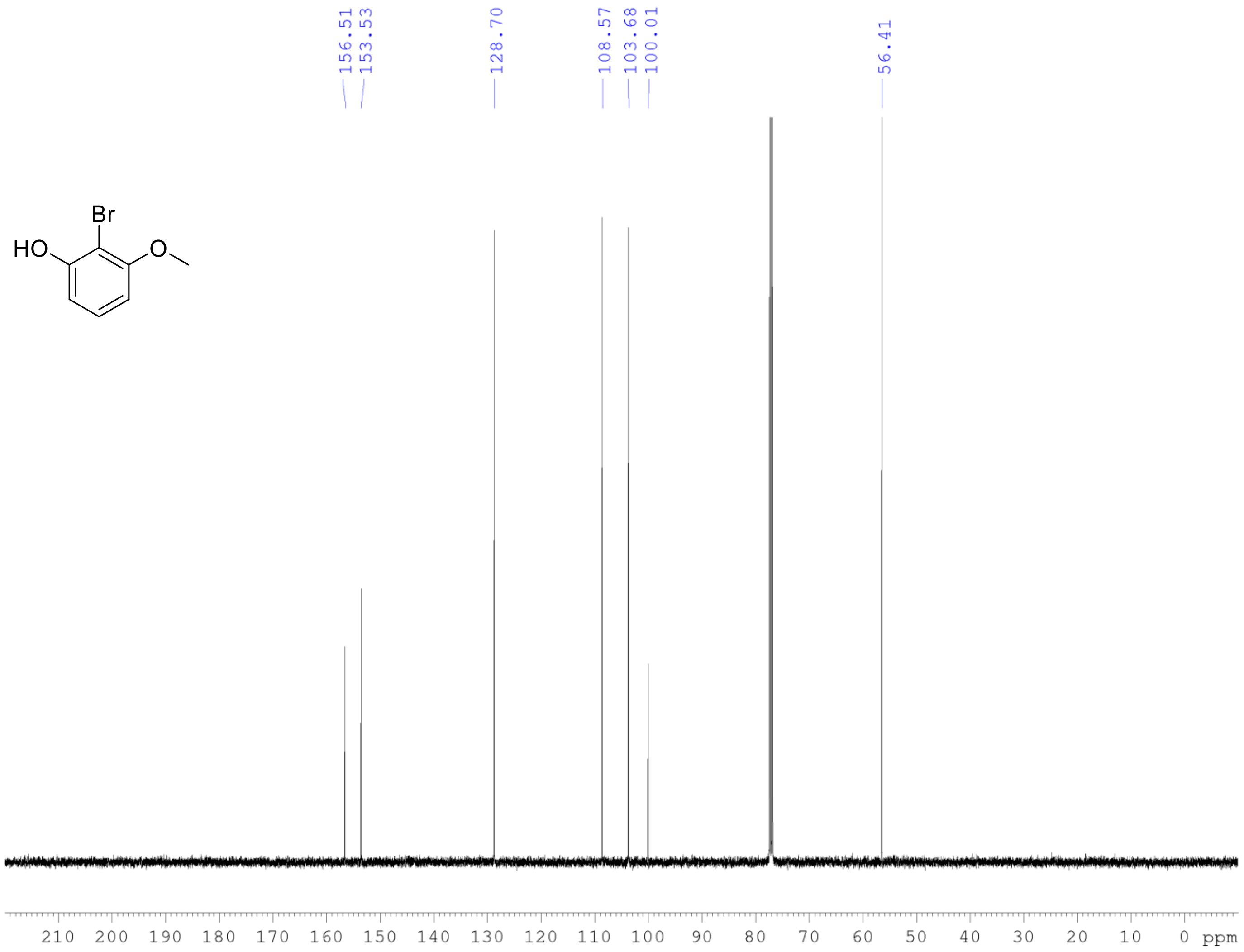

**$^1$H NMR** ($CDCl_3$)**:** 4-chloro-[1,1'-biphenyl]-2-ol

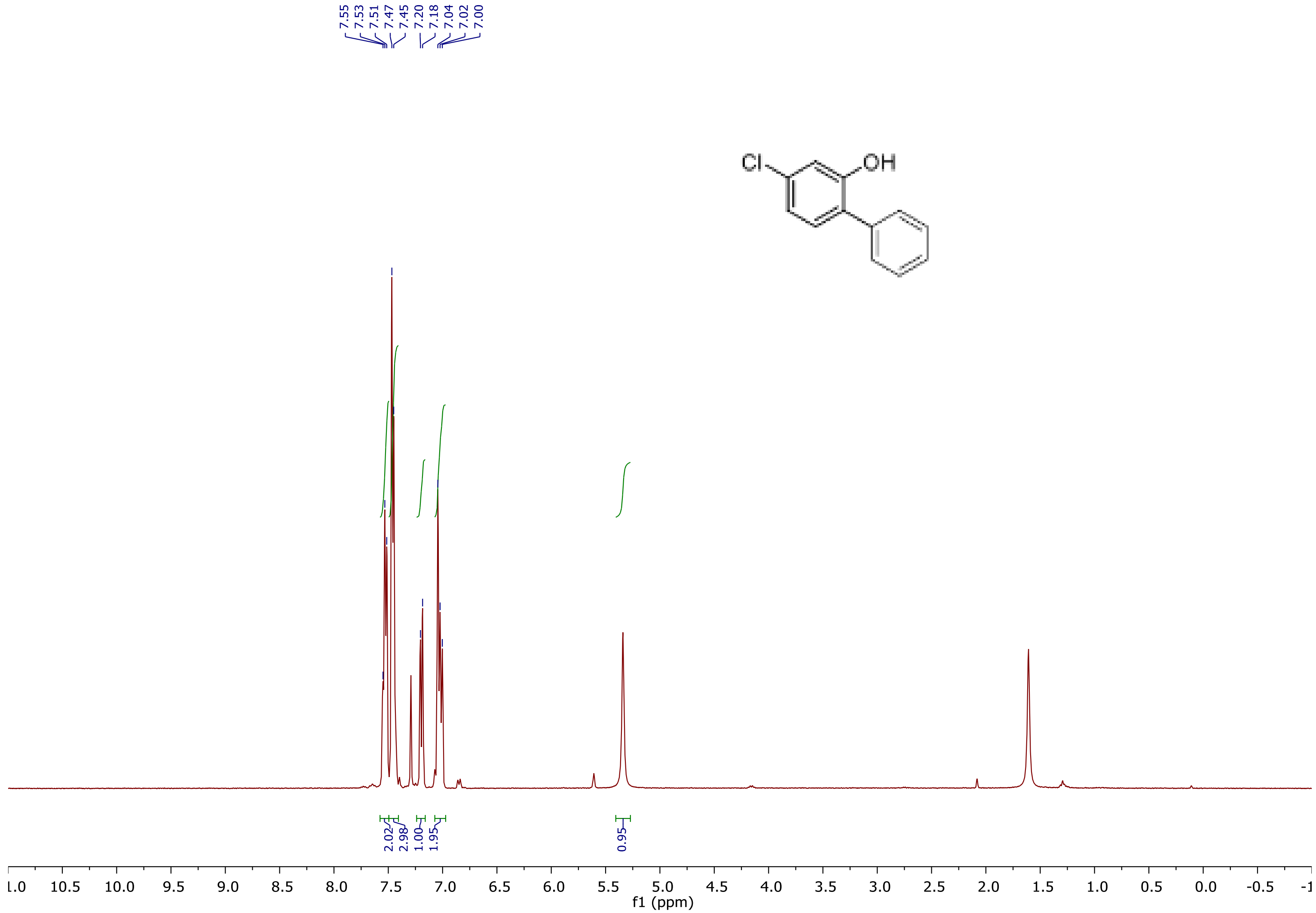

**$^{13}$C NMR** ($CDCl_3$)**:** 4-chloro-[1,1'-biphenyl]-2-ol

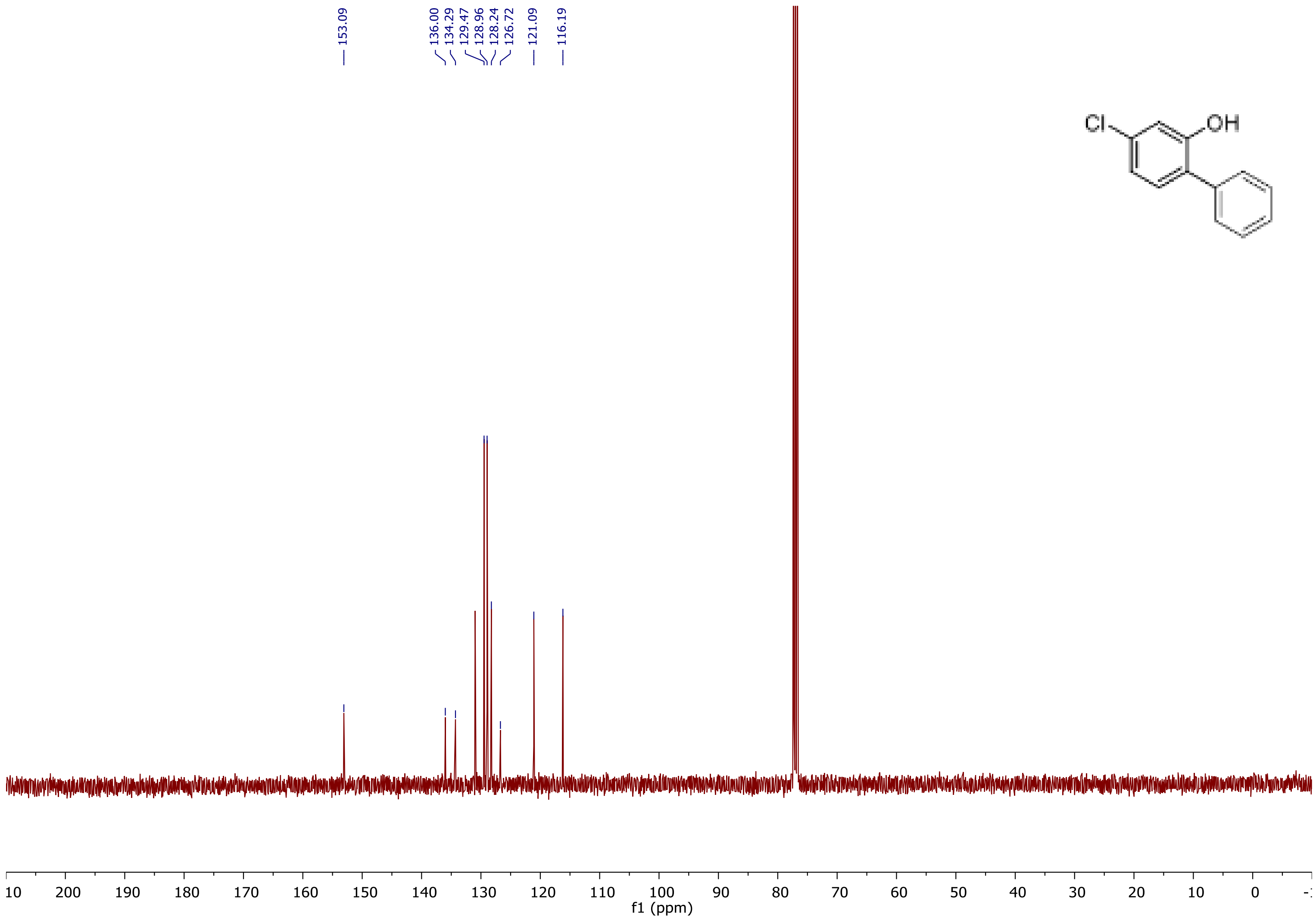

**$^1$H NMR** ($CDCl_3$)**:** 3-bromo-4-chloro-[1,1'-biphenyl]-2-ol

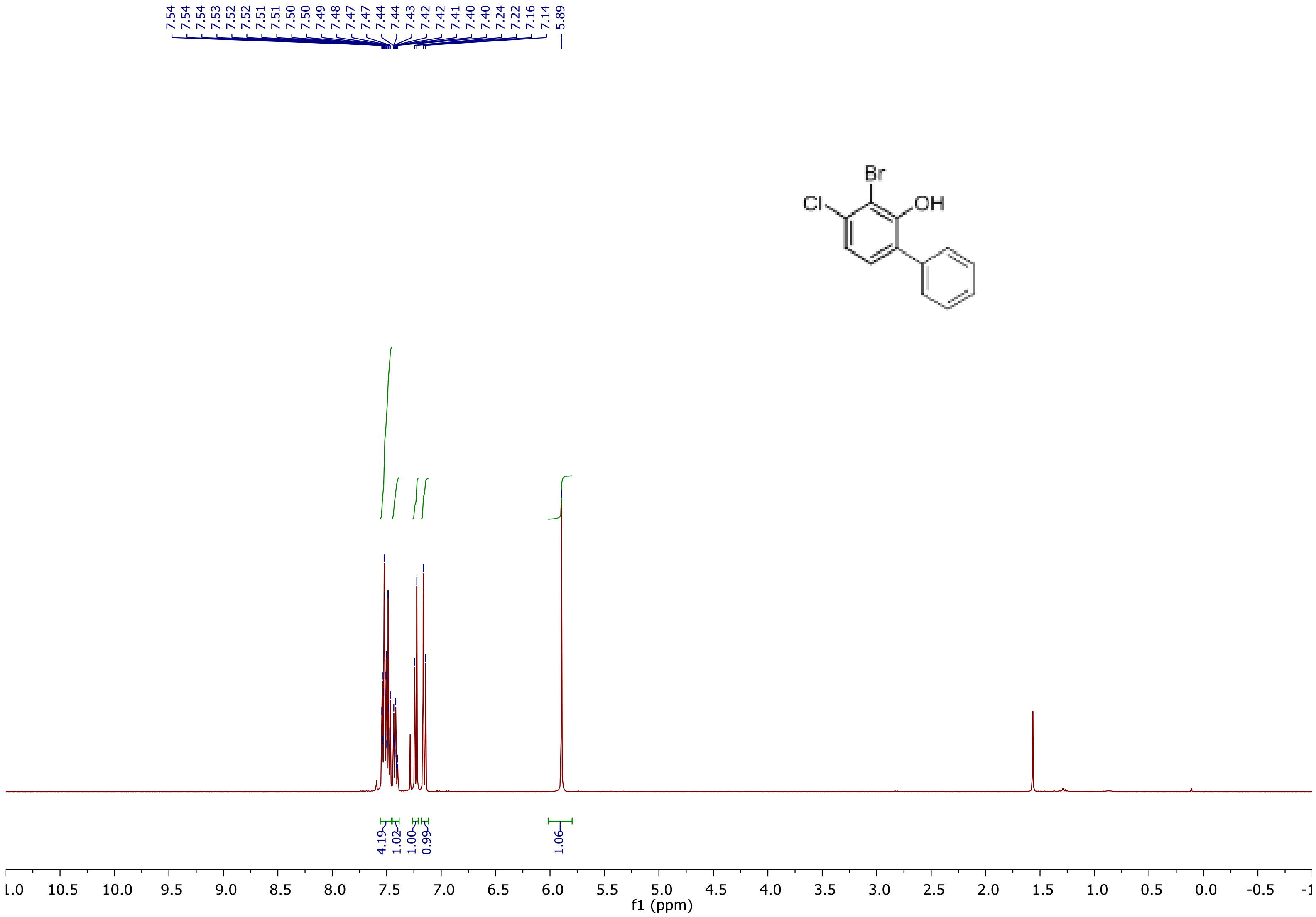

**$^{13}$C NMR** ($CDCl_3$)**:** 3-bromo-4-chloro-[1,1'-biphenyl]-2-ol

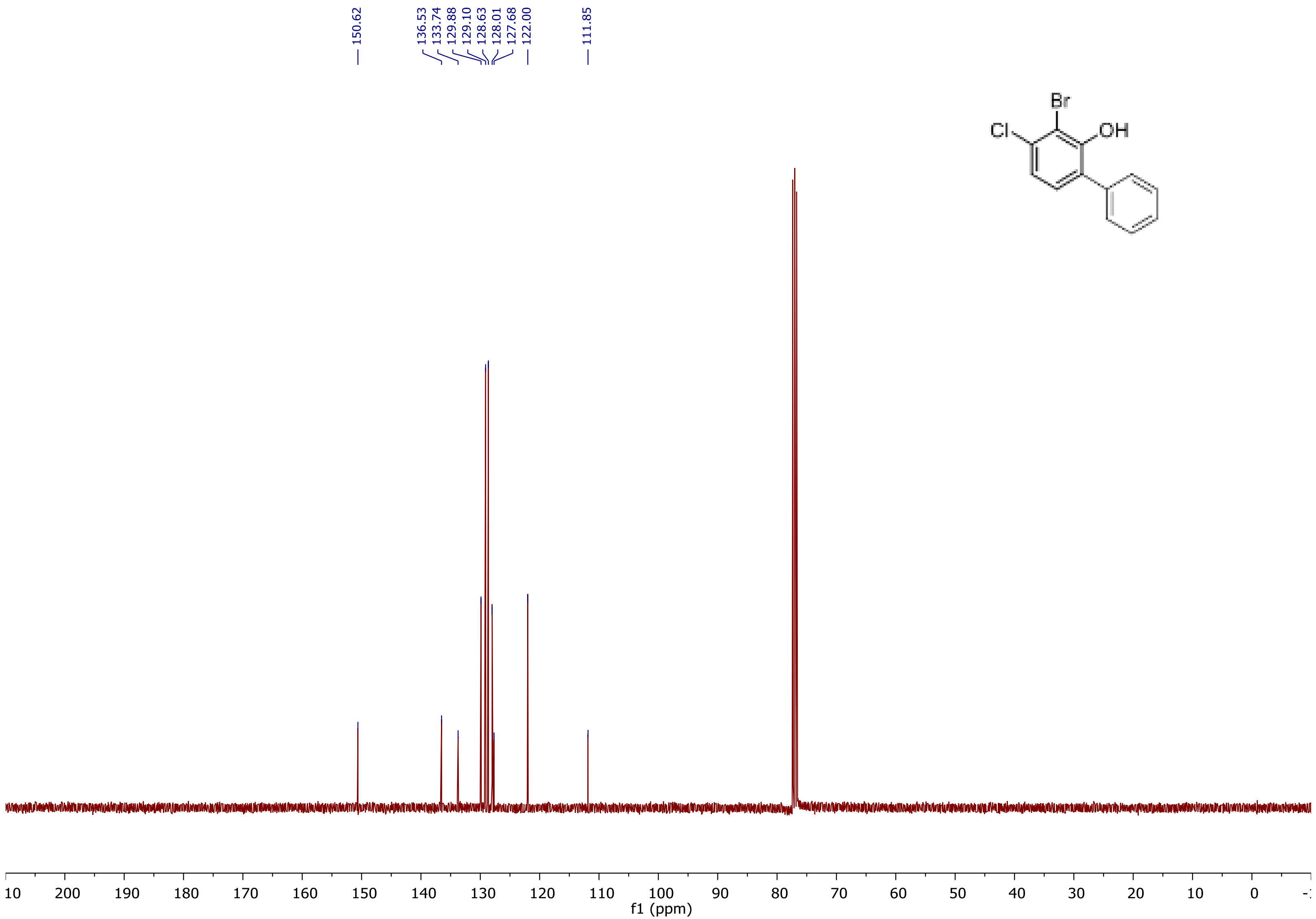

**$^{1}$H NMR** ($CDCl_3$)**:** 2-bromo-6-chloro-3-methylphenol

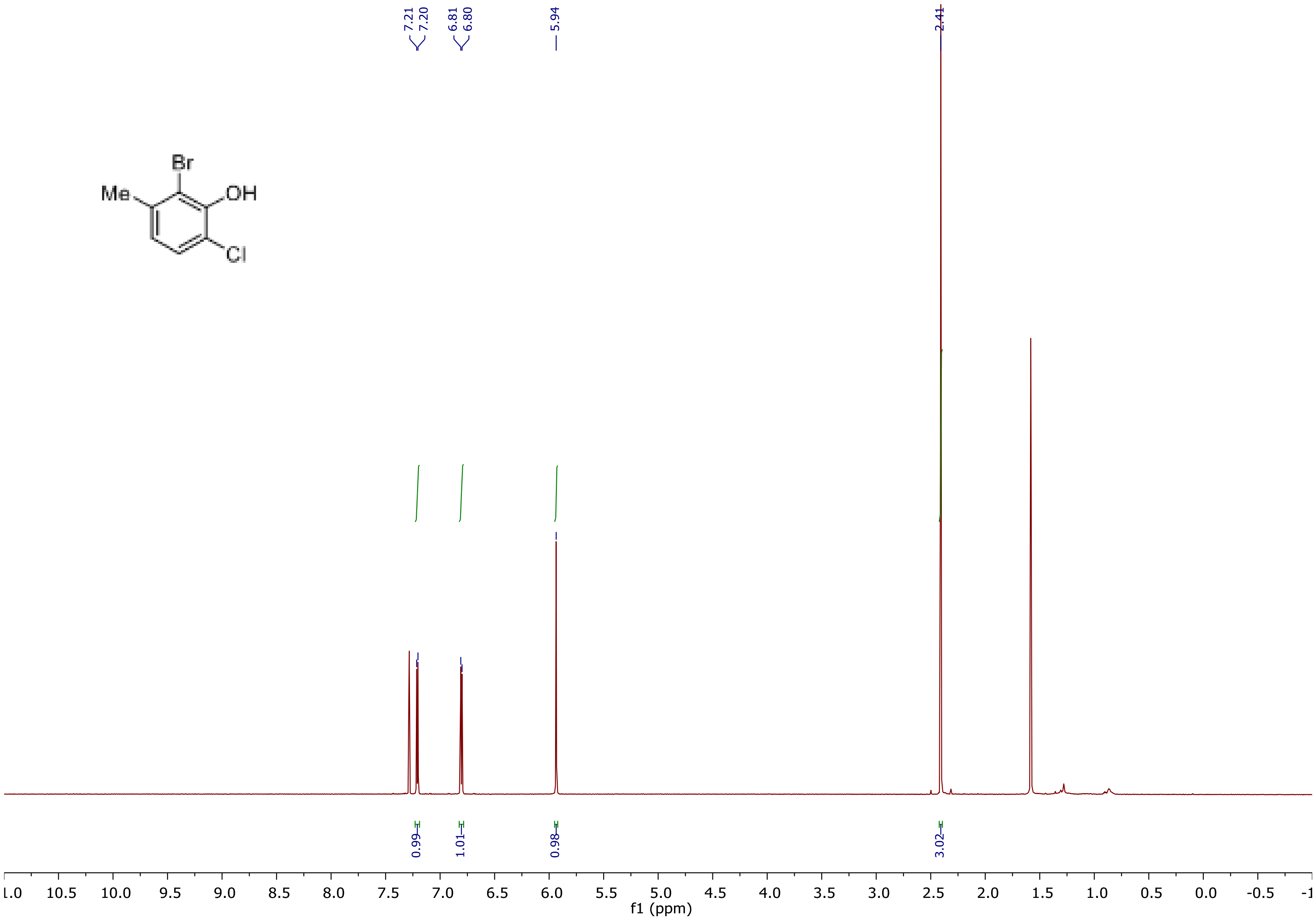

**$^{13}$C NMR** ($CDCl_3$)**:** 2-bromo-6-chloro-3-methylphenol

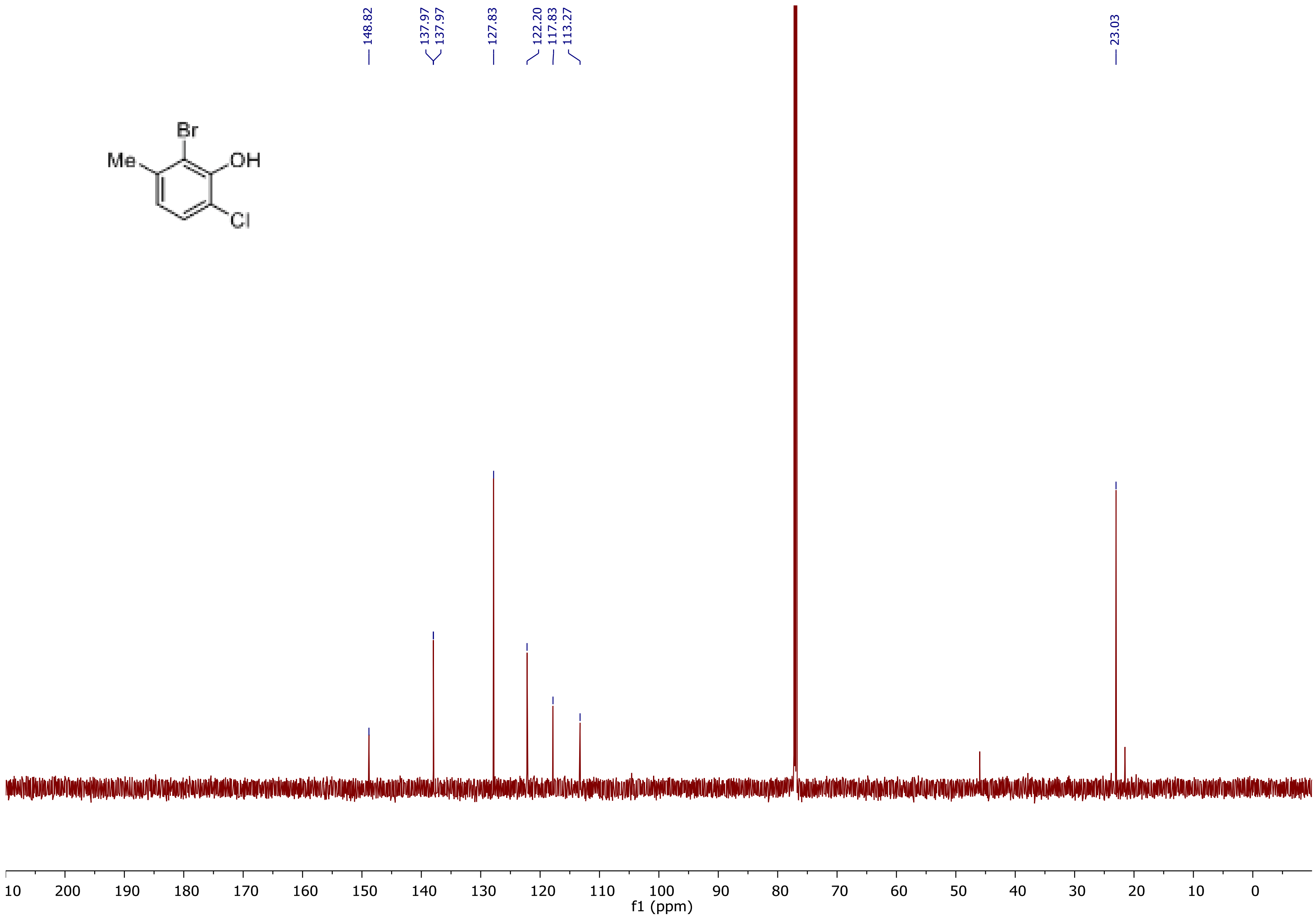

**$^1$H NMR** ($CDCl_3$)**:** 2,6-dibromo-3,5dichlorophenol

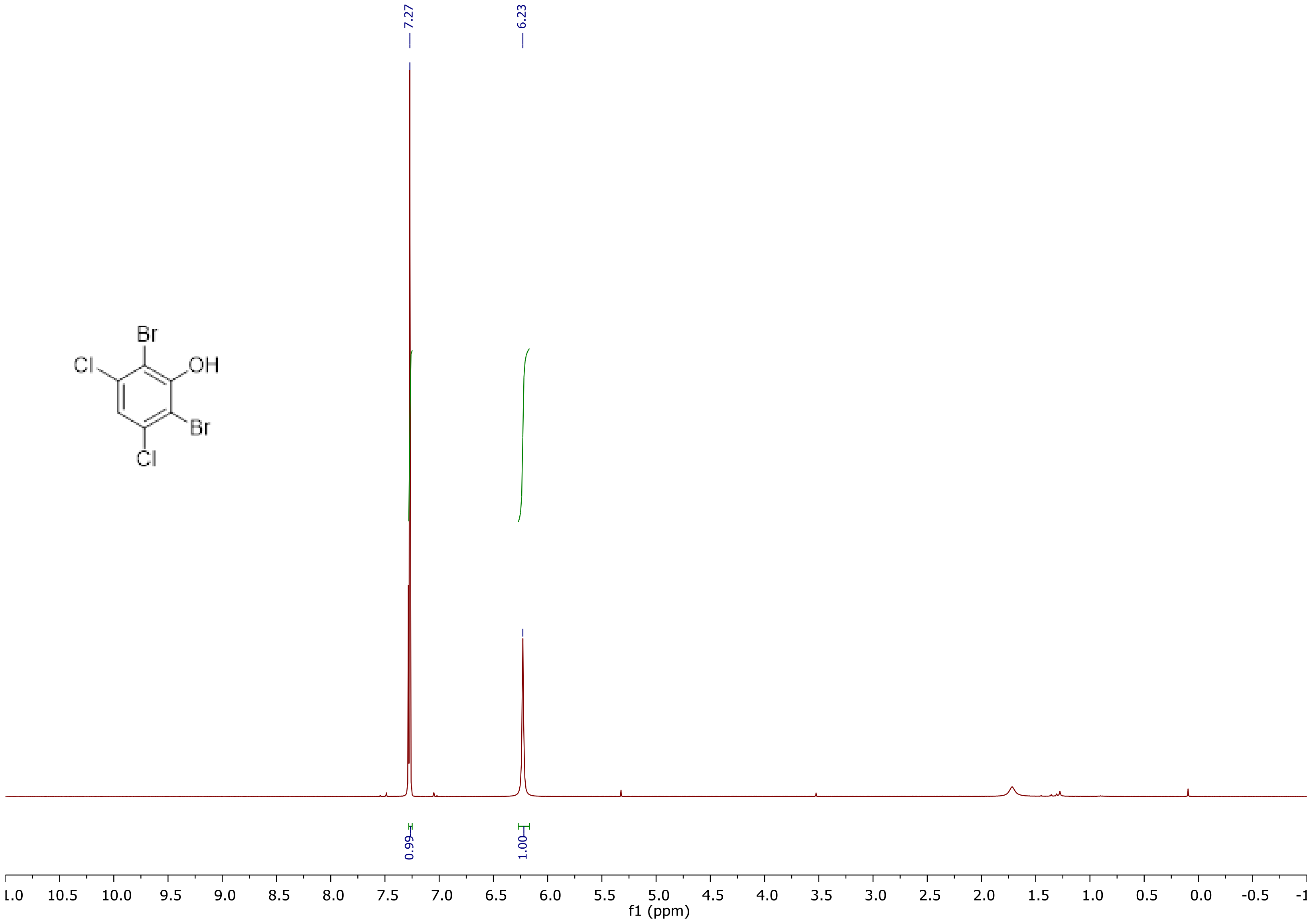

**$^{13}$C NMR** ($CDCl_3$)**:** 2,6-dibromo-3,5dichlorophenol

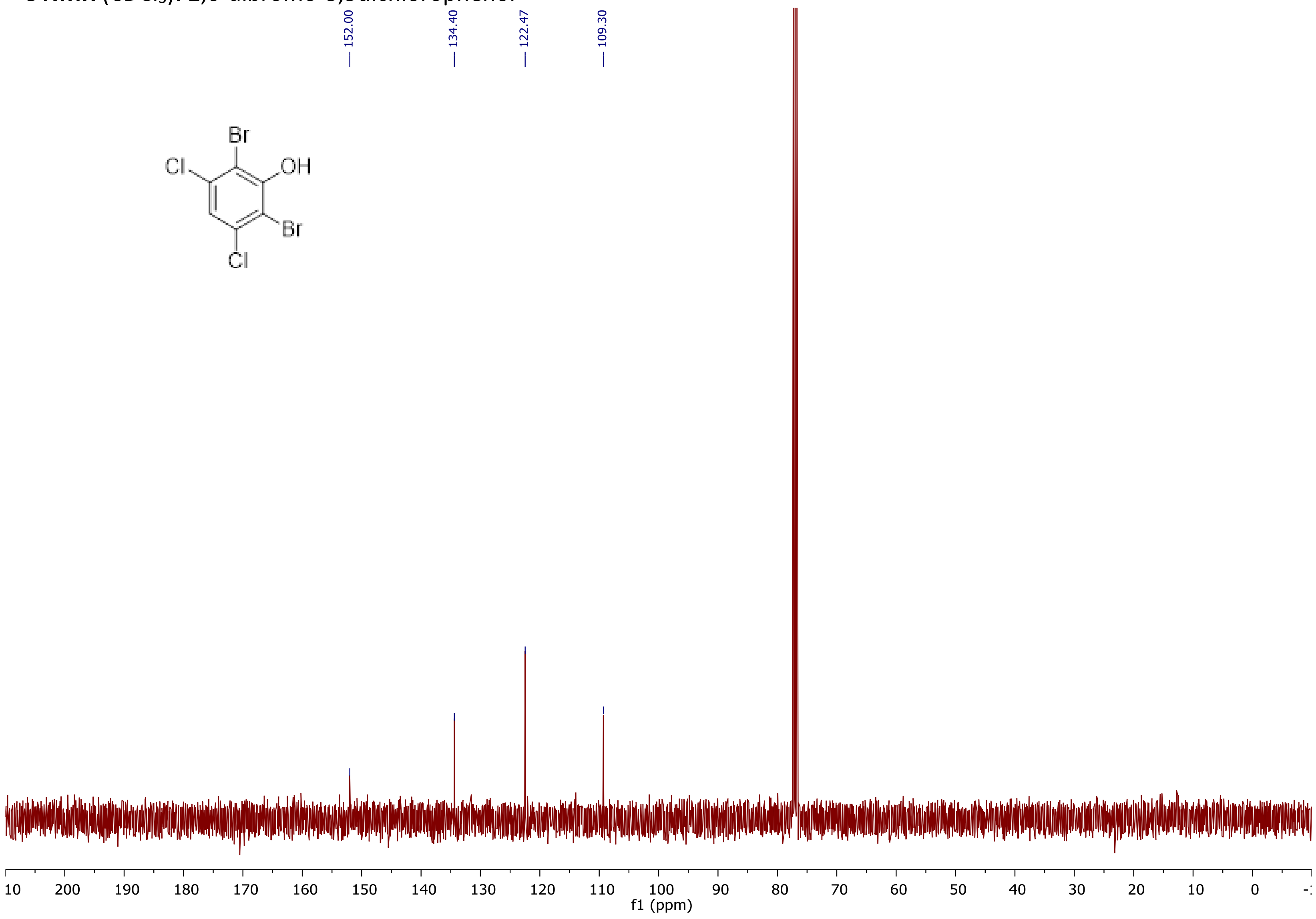

**$^1$H NMR** ($CDCl_3$)**:** 2,6-dibromo-3,5-dimethylphenol

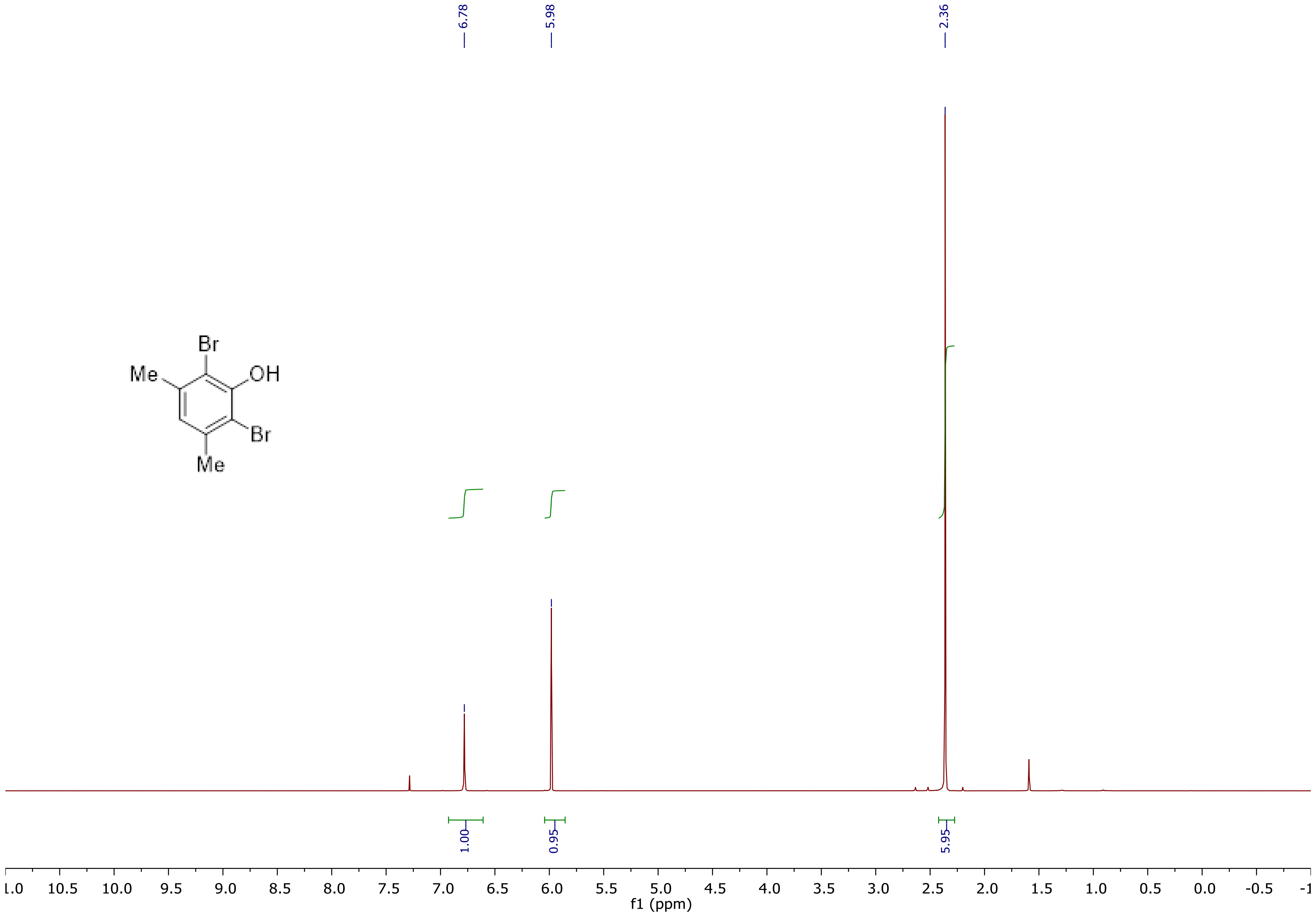

**$^{13}$C NMR** ($CDCl_3$)**:** 2,6-dibromo-3,5-dimethylphenol

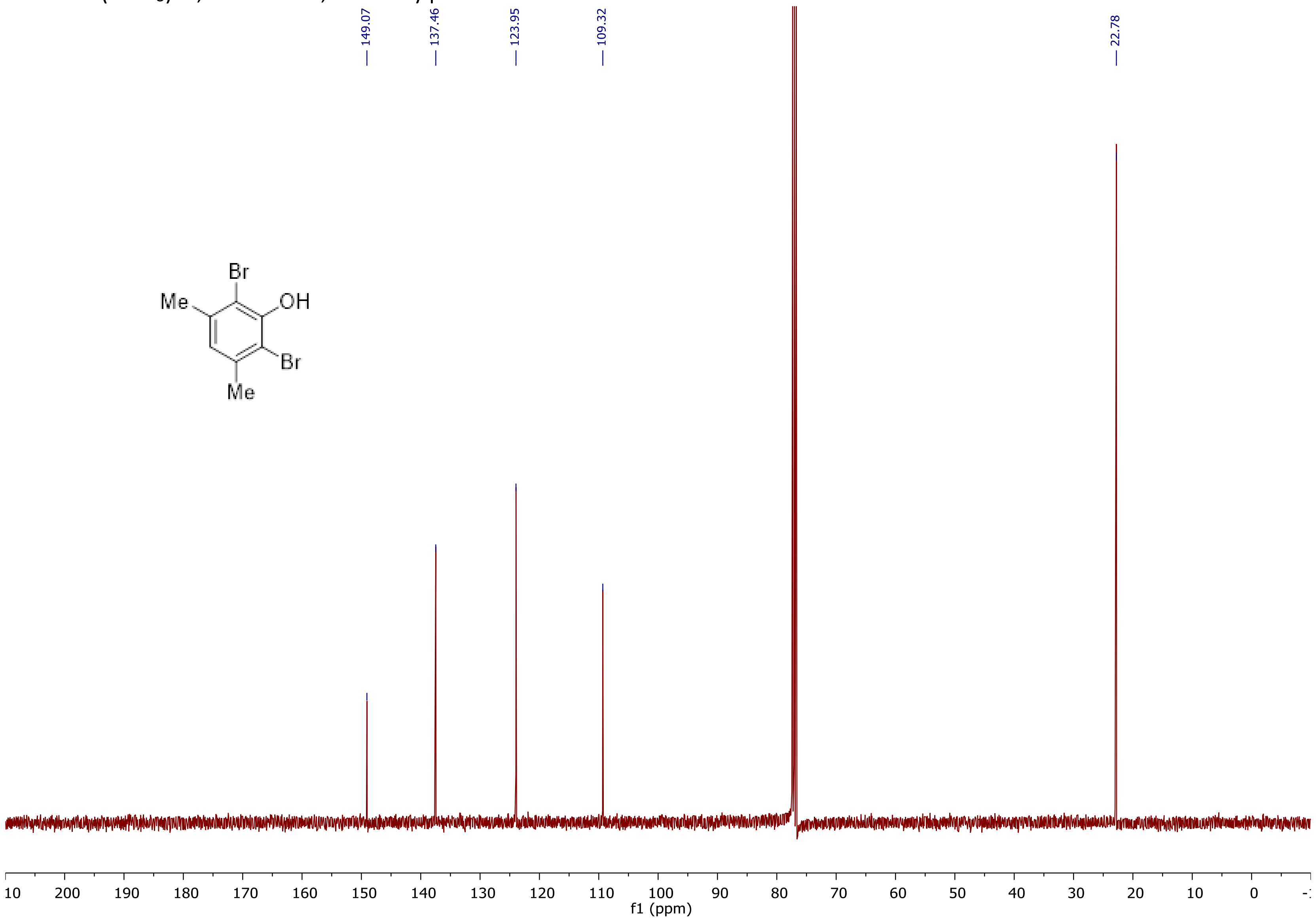

**$^{1}$H NMR** ($CDCl_3$): 3-fluoro-2-(4,4,5,5-tetramethyl-1,3,2-dioxaborolan-2-yl)phenol

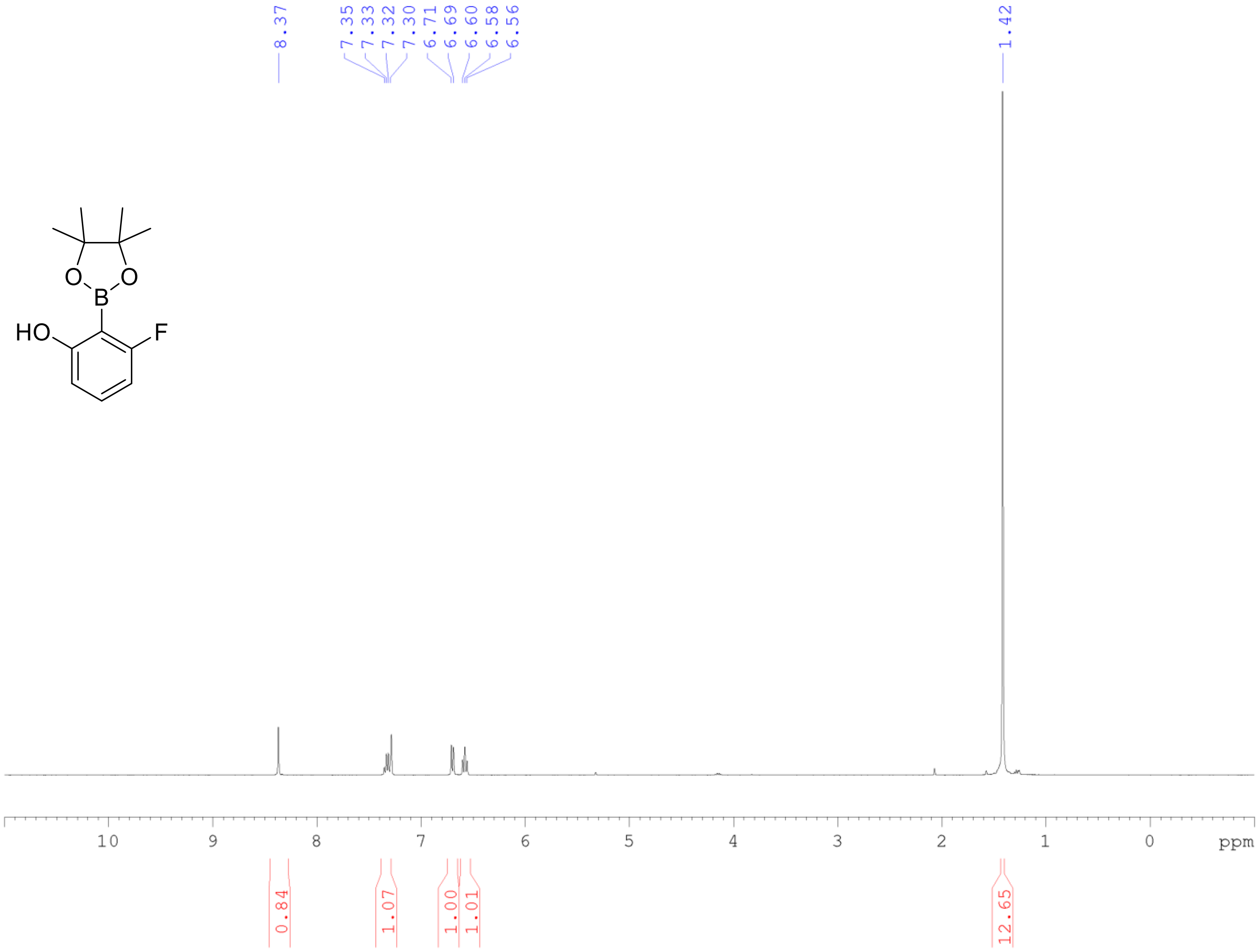

**$^{13}$C NMR** (CDCl$_3$): 3-fluoro-2-(4,4,5,5-tetramethyl-1,3,2-dioxaborolan-2-yl)phenol

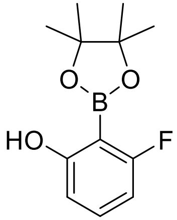


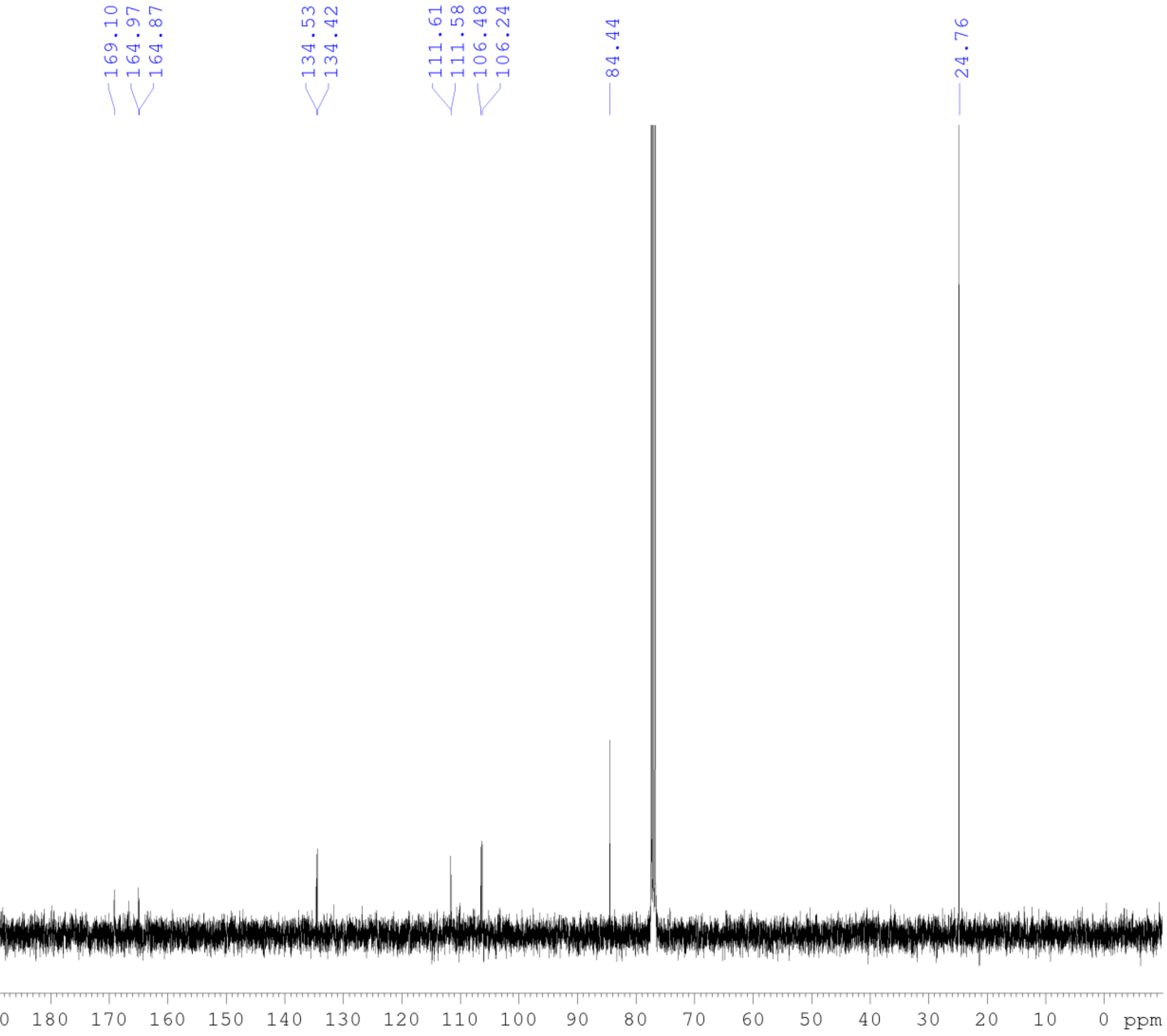

**$^{19}$F NMR** ($CDCl_3$): 3-fluoro-2-(4,4,5,5-tetramethyl-1,3,2-dioxaborolan-2-yl)phenol

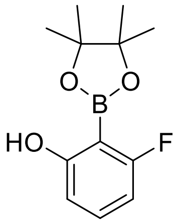


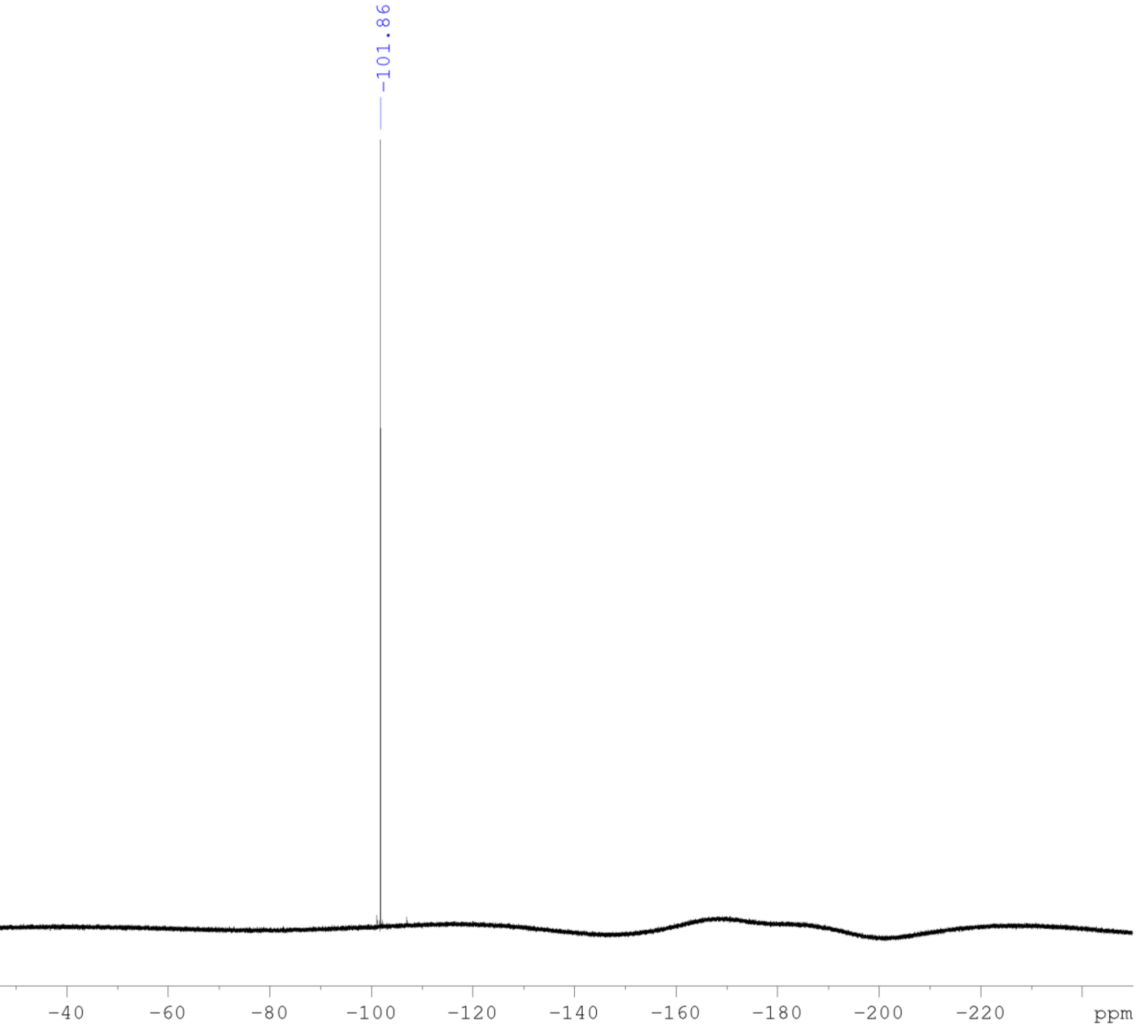

**¹H NMR** ($CDCl_3$): 3-chloro-2-(4,4,5,5-tetramethyl-1,3,2-dioxaborolan-2-yl)phenol

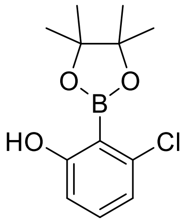


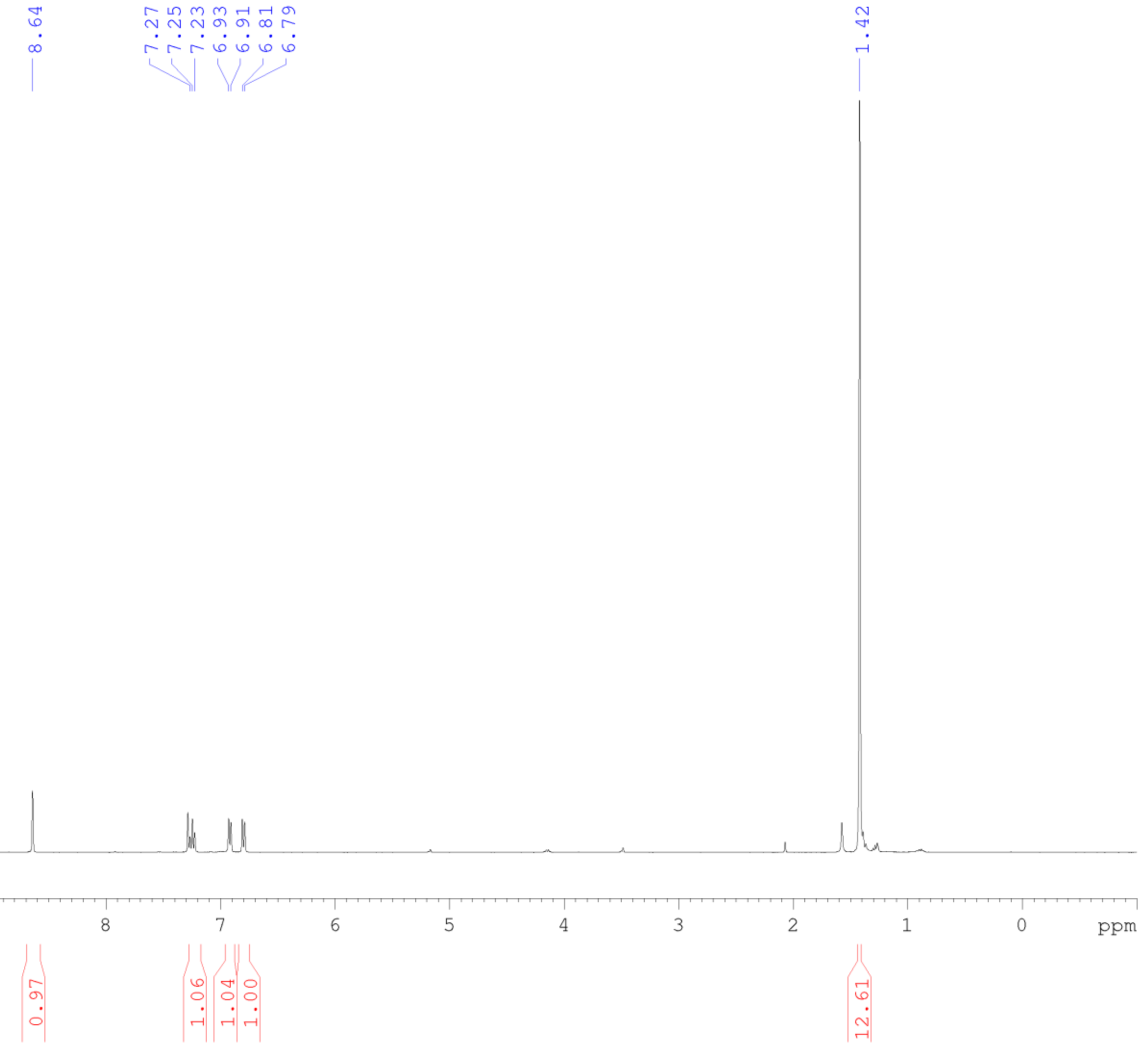

**$^{13}$C NMR** ($CDCl_3$): 3-chloro-2-(4,4,5,5-tetramethyl-1,3,2-dioxaborolan-2-yl)phenol

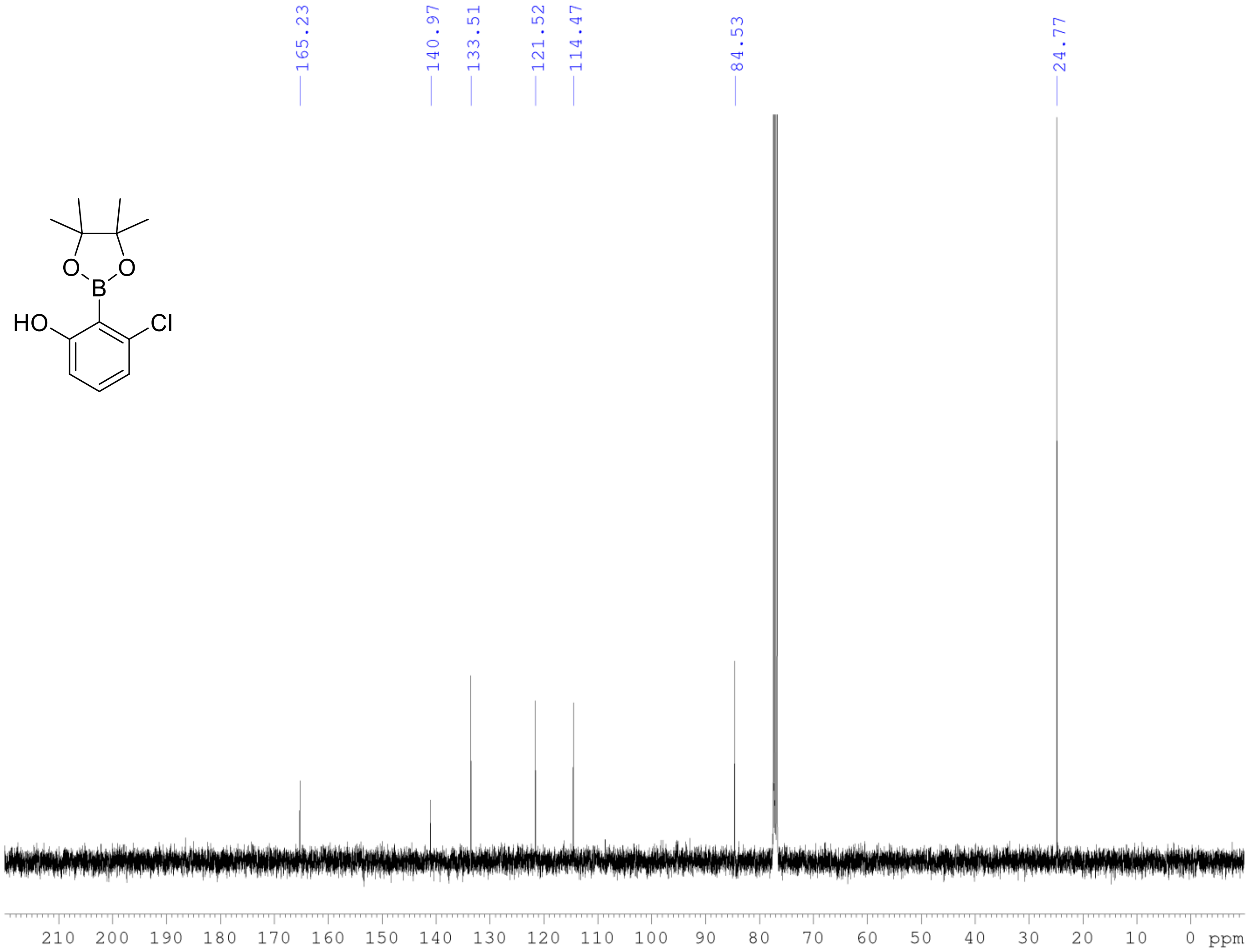

**$^{1}$H NMR** ($CDCl_3$): 1-(methoxymethoxy)-3-(trifluoromethoxy)benzene

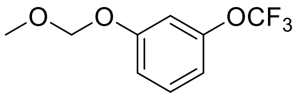

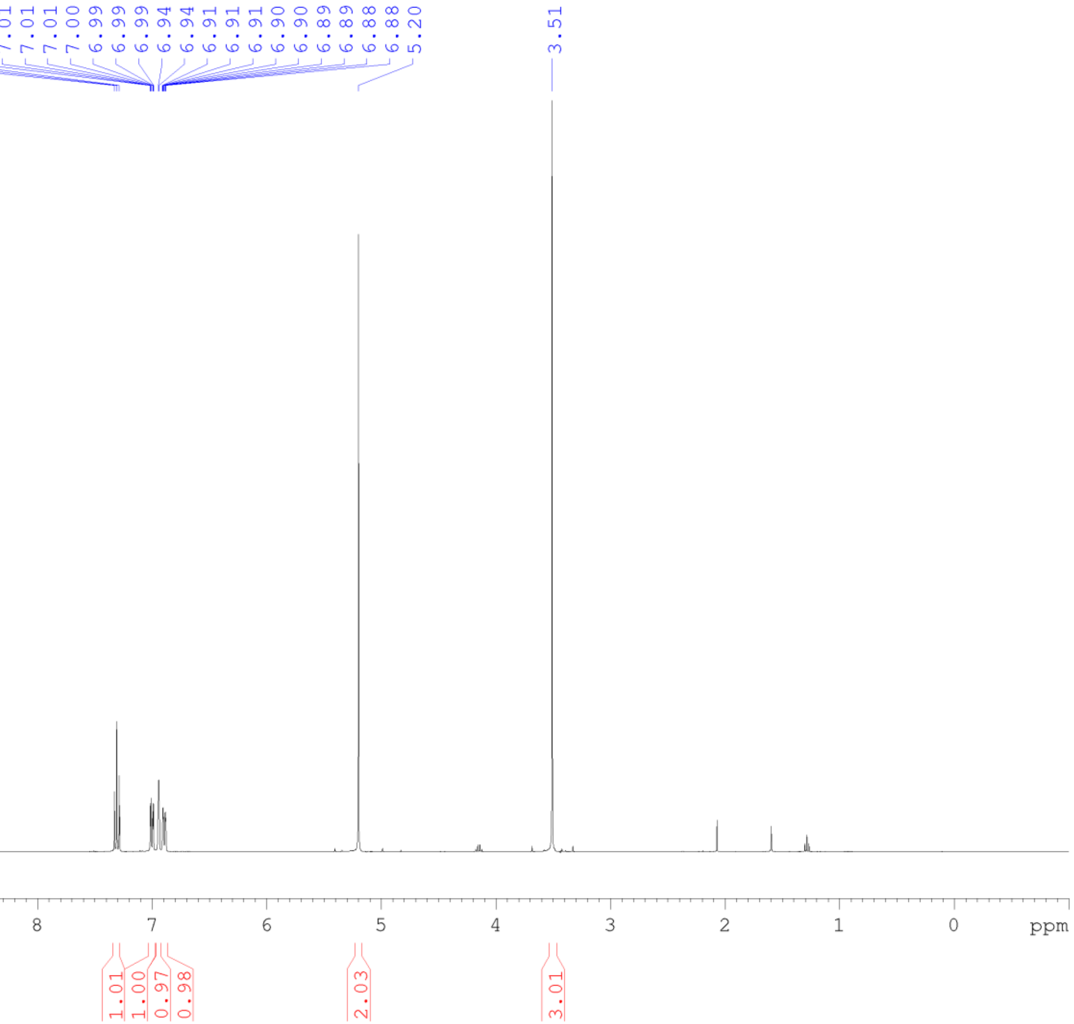

**$^{13}$C NMR** ($CDCl_3$): 1-(methoxymethoxy)-3-(trifluoromethoxy)benzene

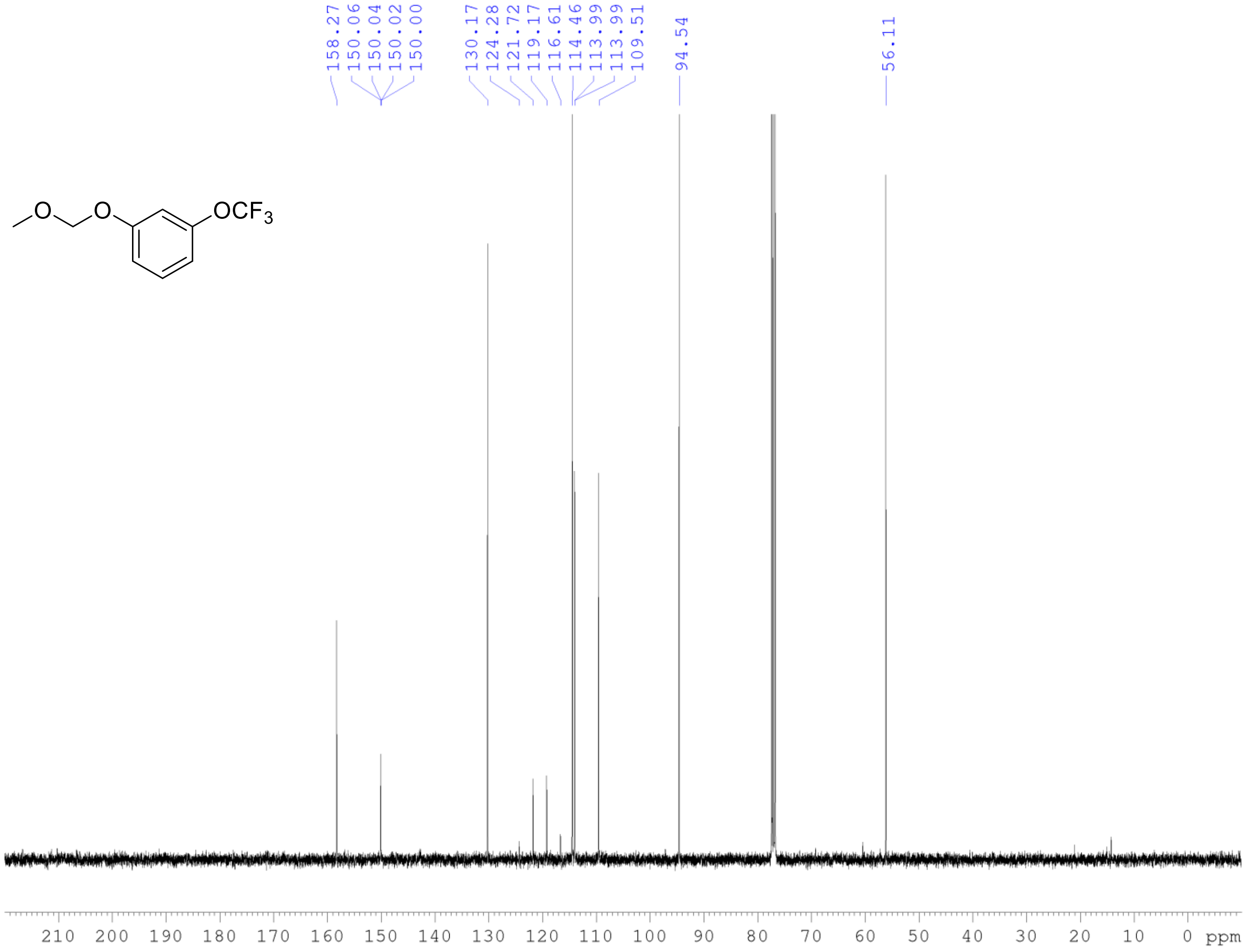

**$^{19}$F NMR** ($CDCl_3$): 1-(methoxymethoxy)-3-(trifluoromethoxy)benzene

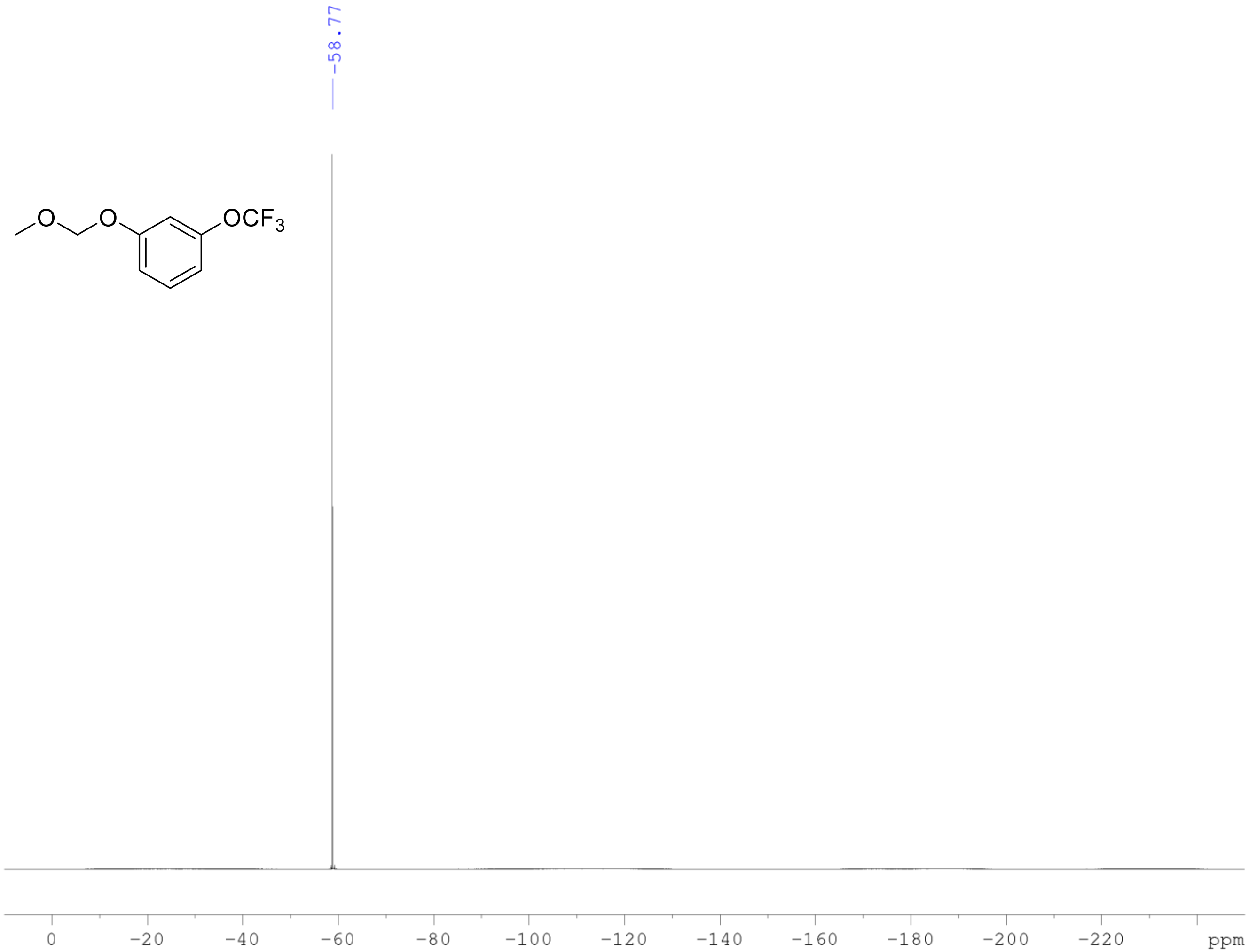

**¹H NMR** ($CDCl_3$): 2-(4,4,5,5-tetramethyl-1,3,2-dioxaborolan-2-yl)-3-(trifluoromethoxy)phenol

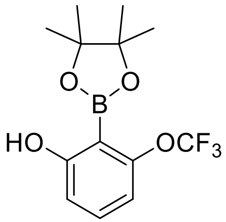


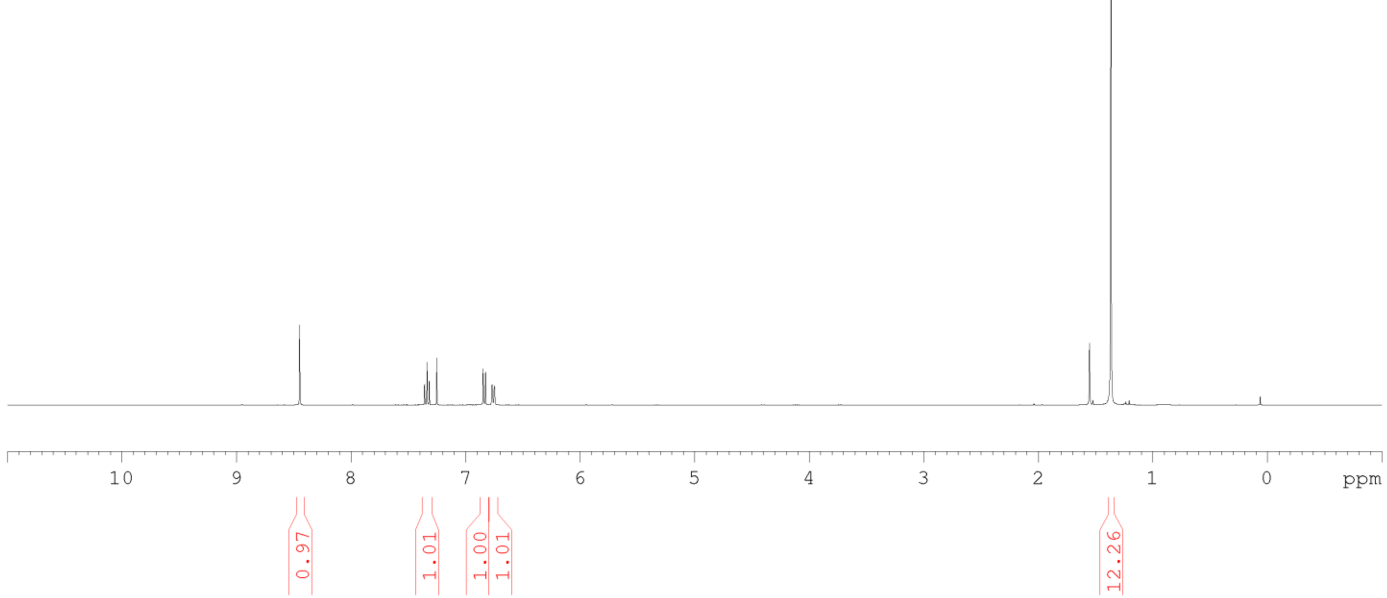

**$^{13}$C NMR** ($CDCl_3$): 2-(4,4,5,5-tetramethyl-1,3,2-dioxaborolan-2-yl)-3-(trifluoromethoxy)phenol

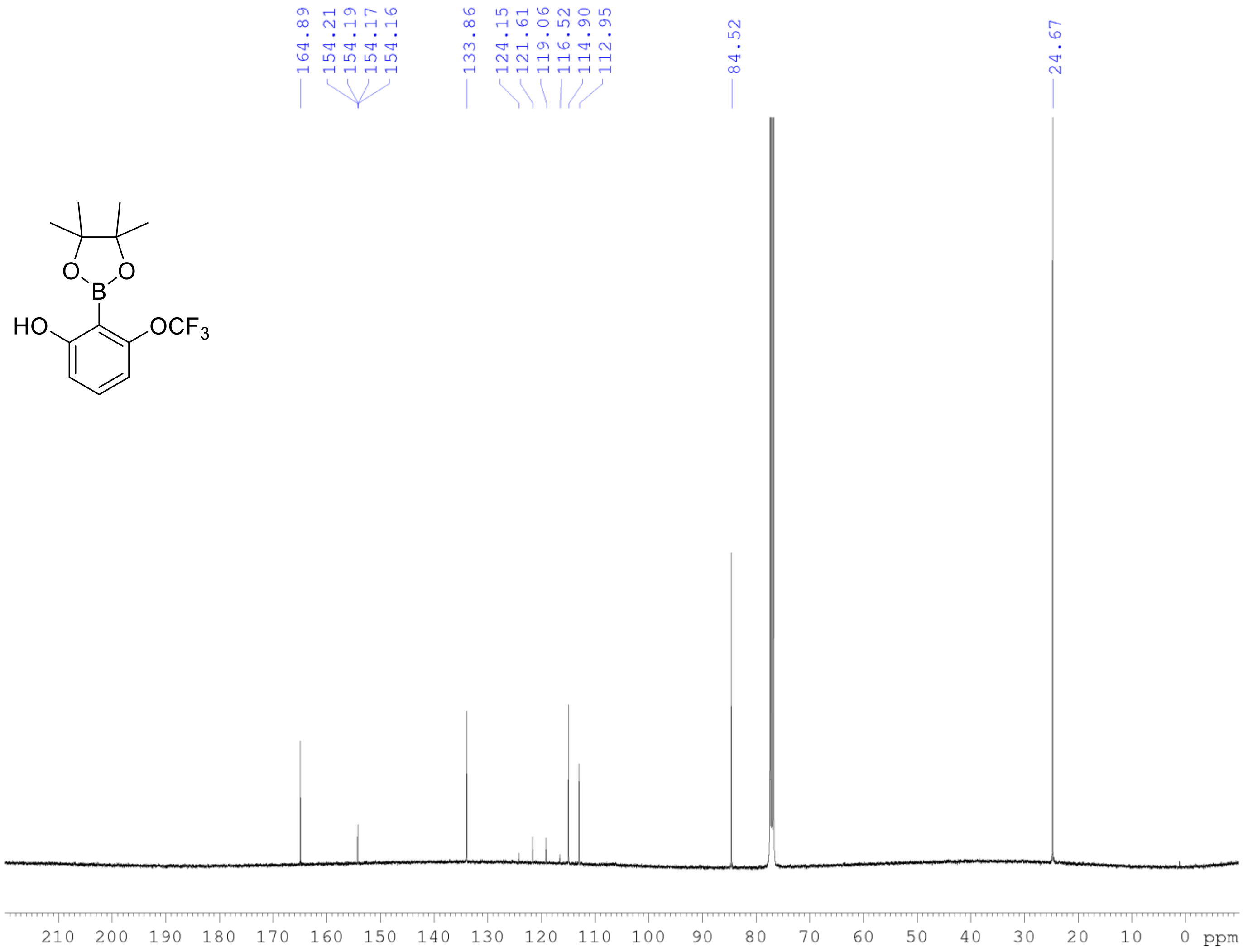

**$^{19}$F NMR** ($CDCl_3$): 2-(4,4,5,5-tetramethyl-1,3,2-dioxaborolan-2-yl)-3-(trifluoromethoxy)phenol

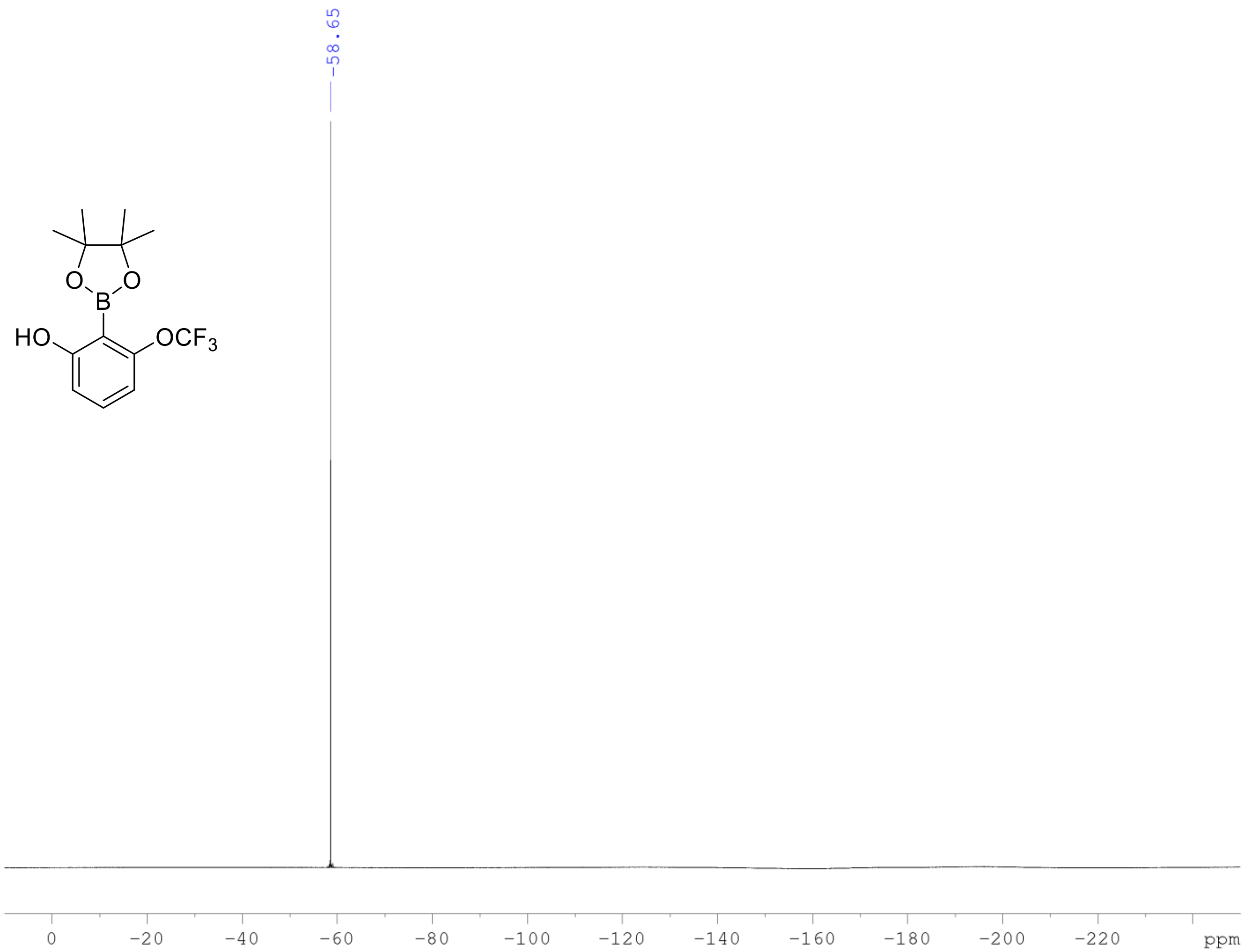

**$^{1}$H NMR** ($CDCl_3$): 2-(2-fluoro-6-methoxyphenyl)-4,4,5,5-tetramethyl-1,3,2-dioxaborolane

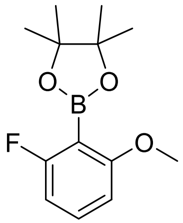


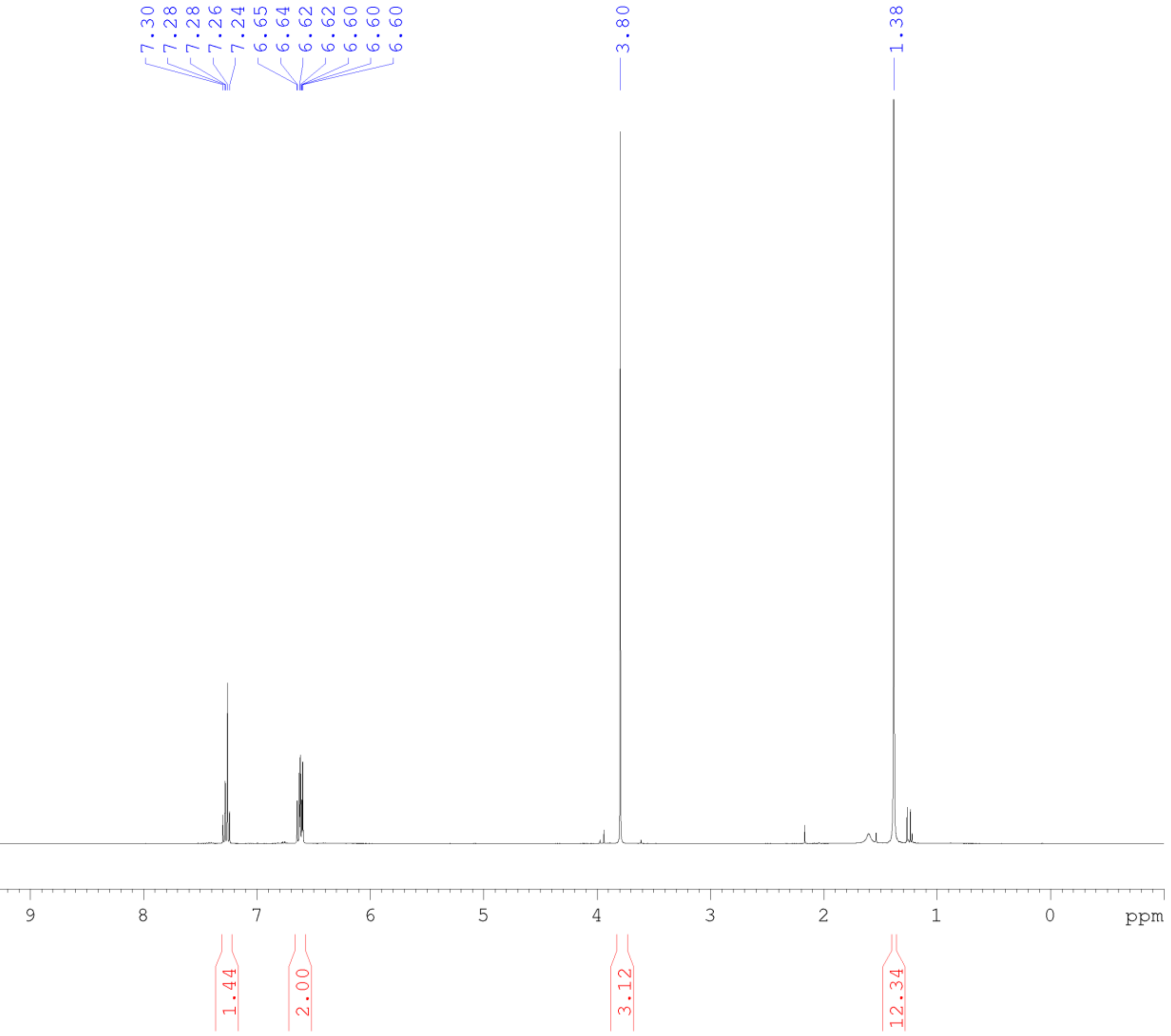

**$^{13}$C NMR** ($CDCl_3$): 2-(2-fluoro-6-methoxyphenyl)-4,4,5,5-tetramethyl-1,3,2-dioxaborolane

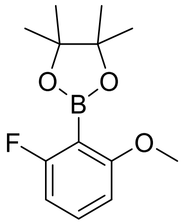


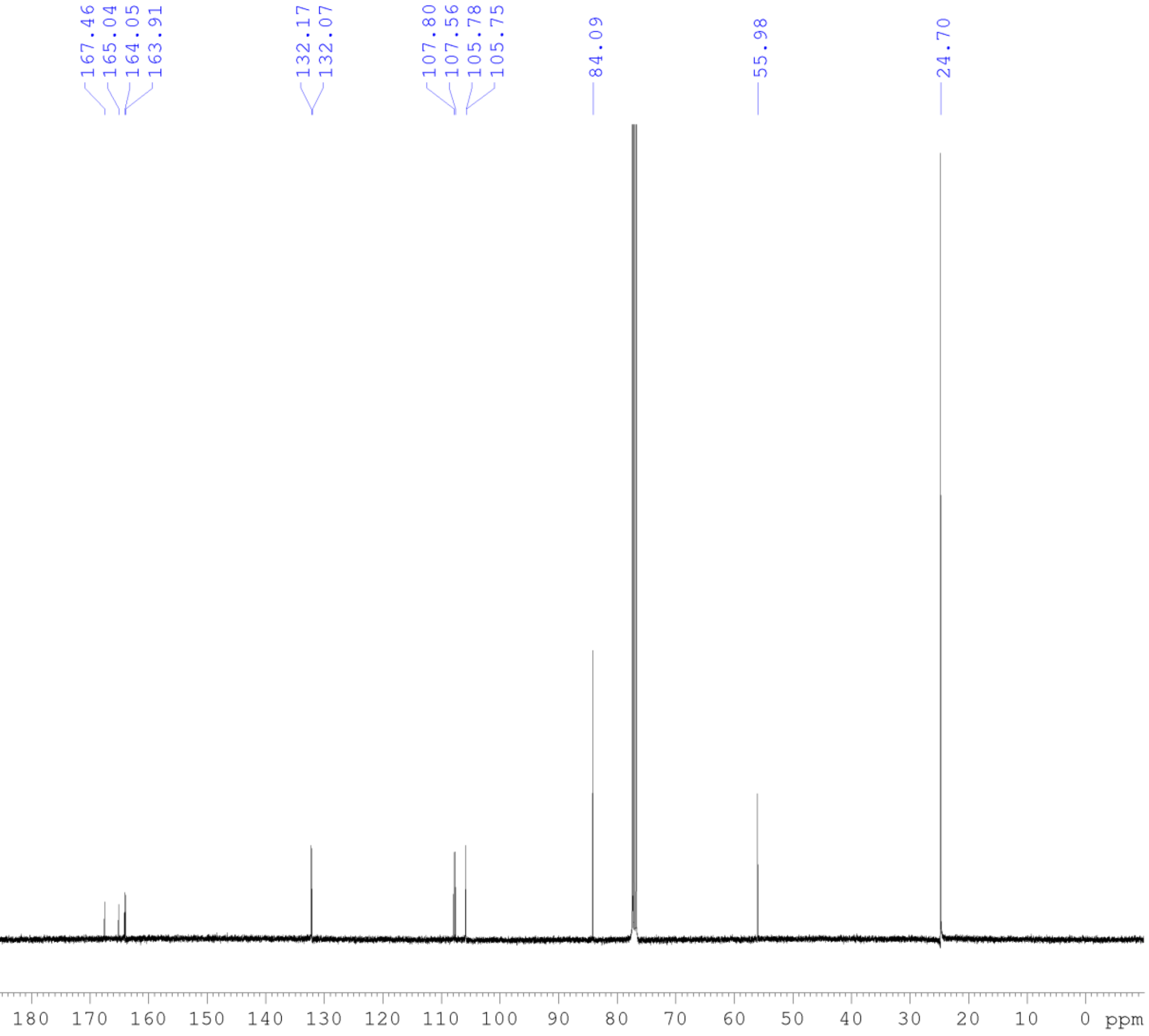

**$^{19}$F NMR** ($CDCl_3$): 2-(2-fluoro-6-methoxyphenyl)-4,4,5,5-tetramethyl-1,3,2-dioxaborolane

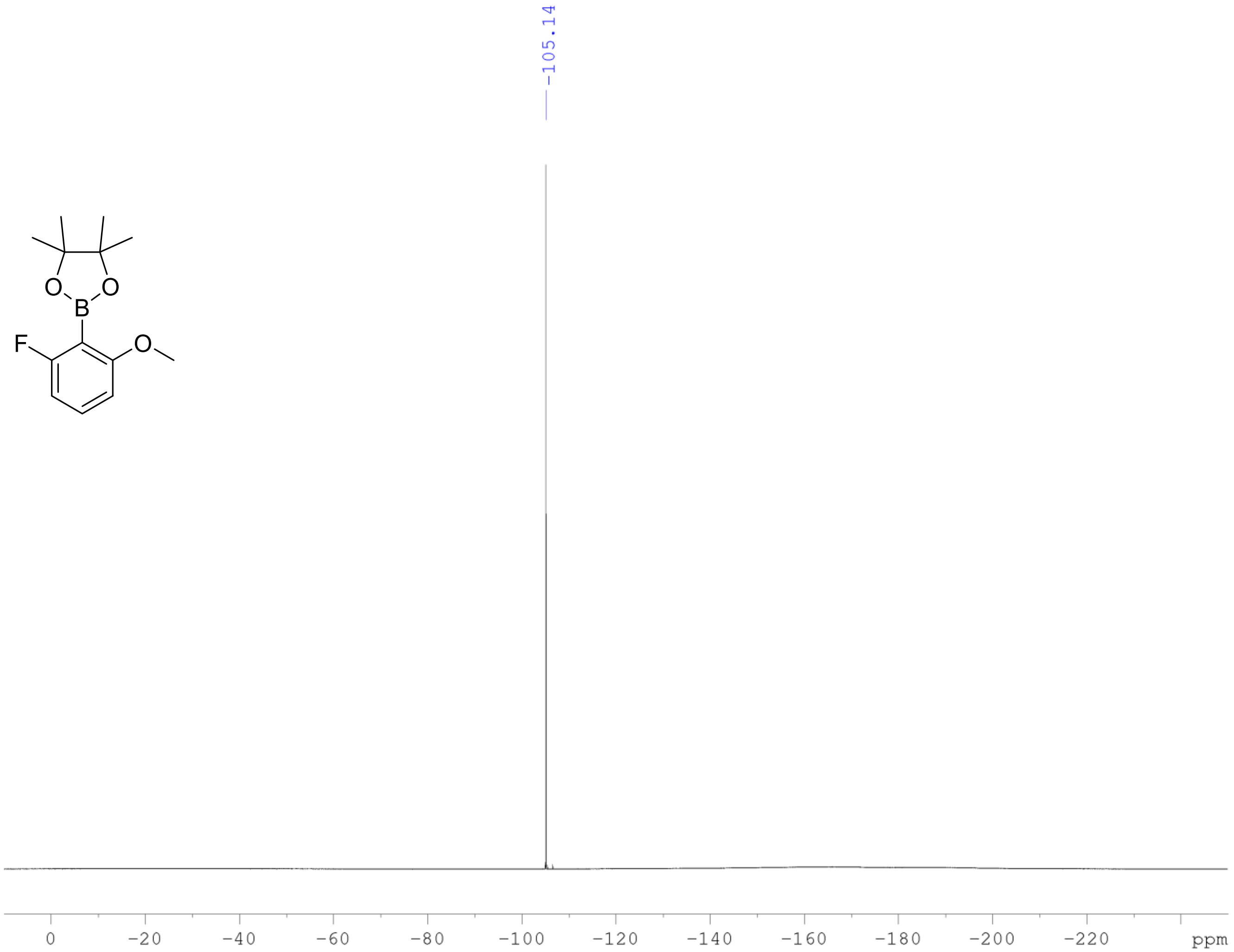

**¹H NMR** (CDCl$_3$): *N*-(2-bromo-3-methylphenyl)acetamide

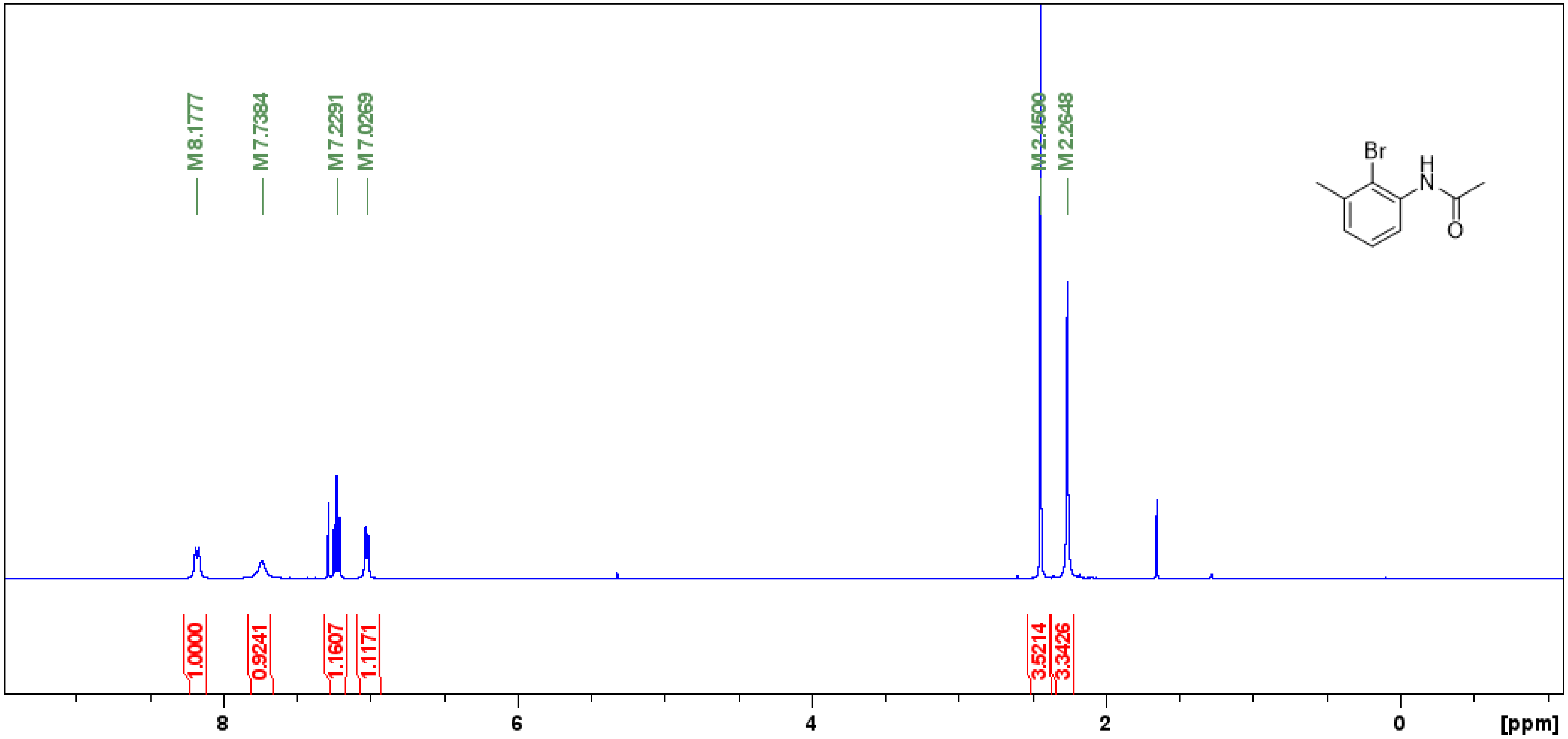

**$^{13}$C NMR** ($CDCl_3$)**:** *N*-(2-bromo-3-methylphenyl)acetamide

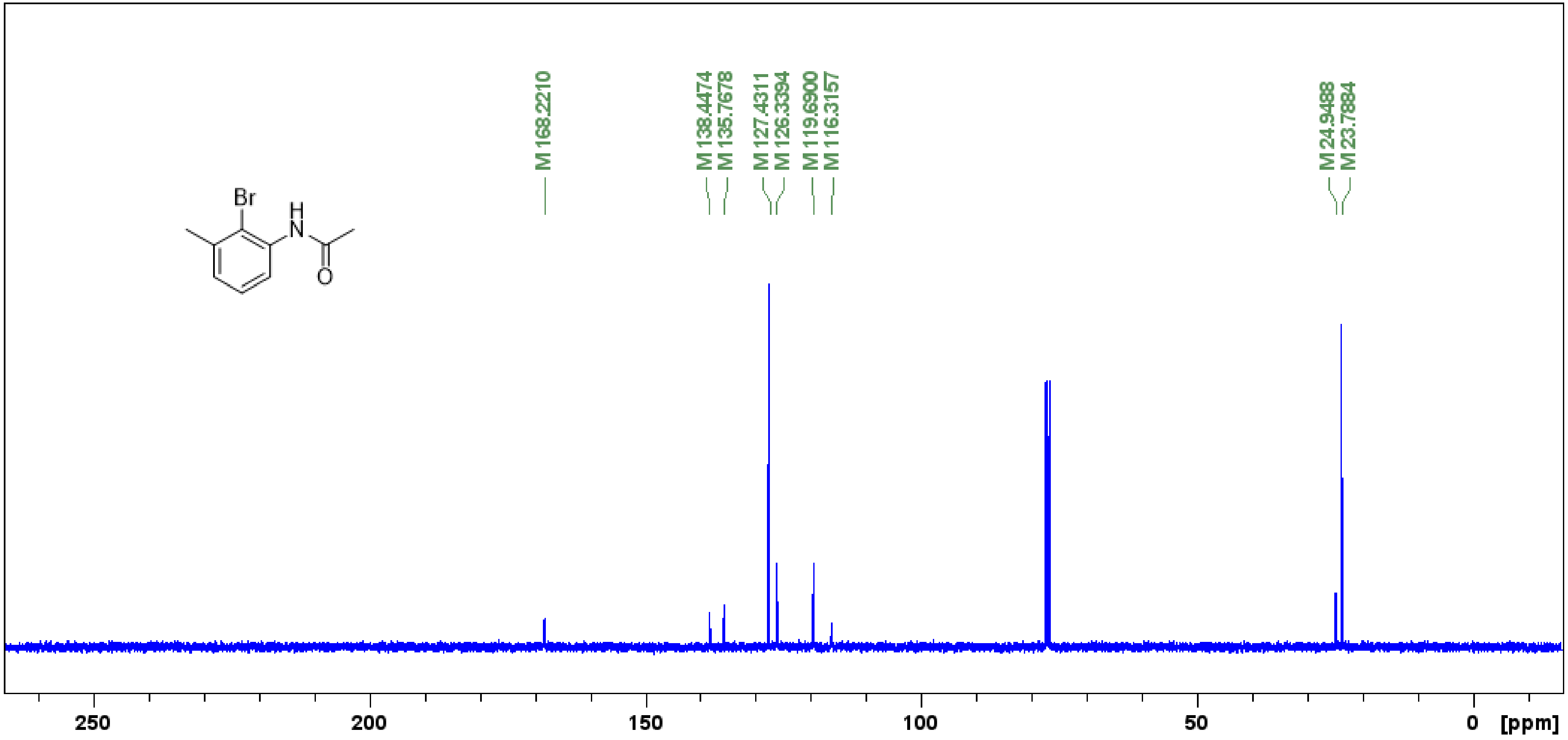

**$^1$H NMR** ($CDCl_3$) ethyl (E)-3-(2-bromo-3-hydroxyphenyl)acrylate

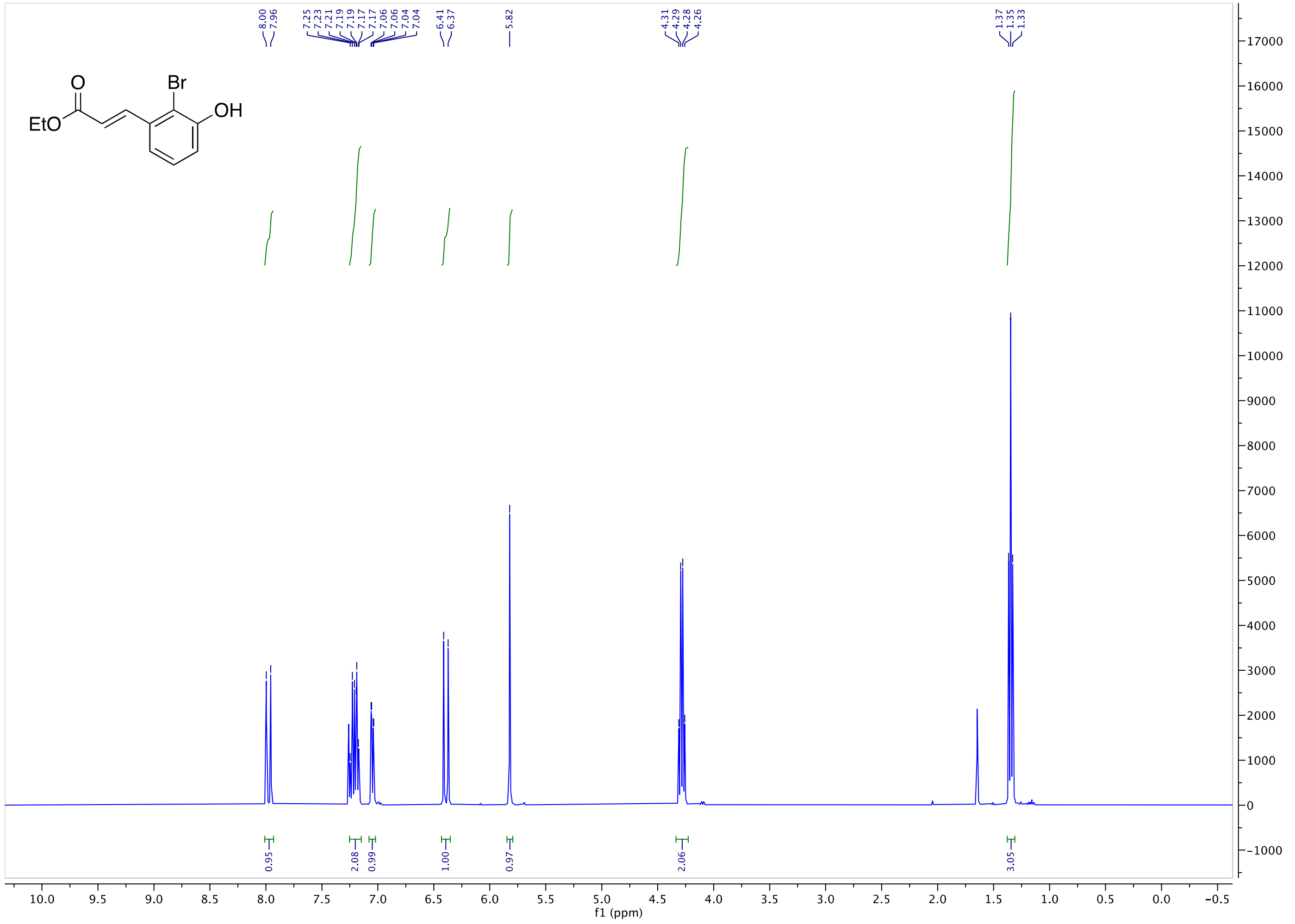

**$^{13}$C NMR** ($CDCl_3$) ethyl (E)-3-(2-bromo-3-hydroxyphenyl)acrylate

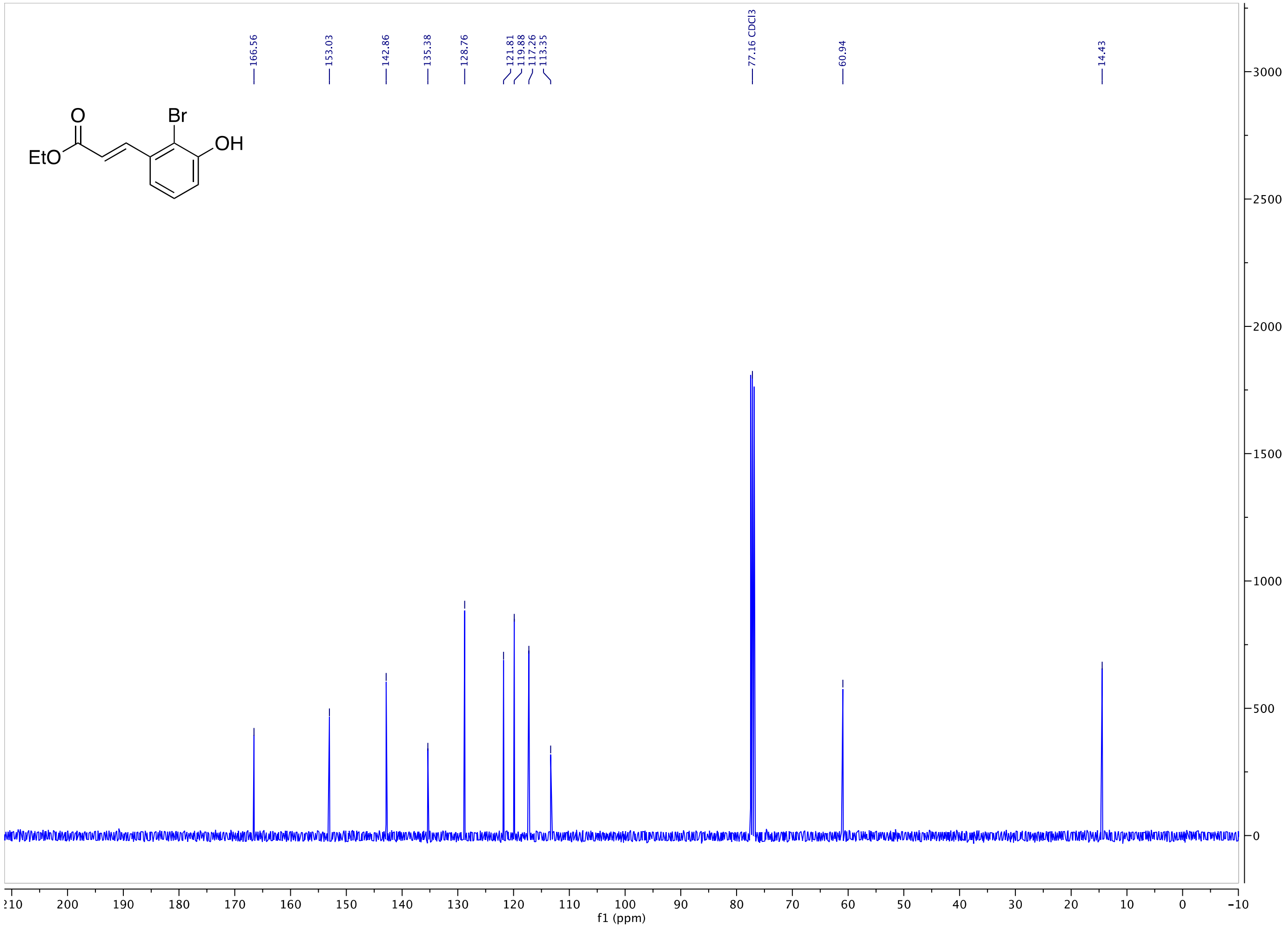

# NMR Spectra of Products

**$^1$H NMR** ($CDCl_3$): 6-fluoro-6'-methyl-[1,1'-biphenyl]-2,2'-diol (**3a**)

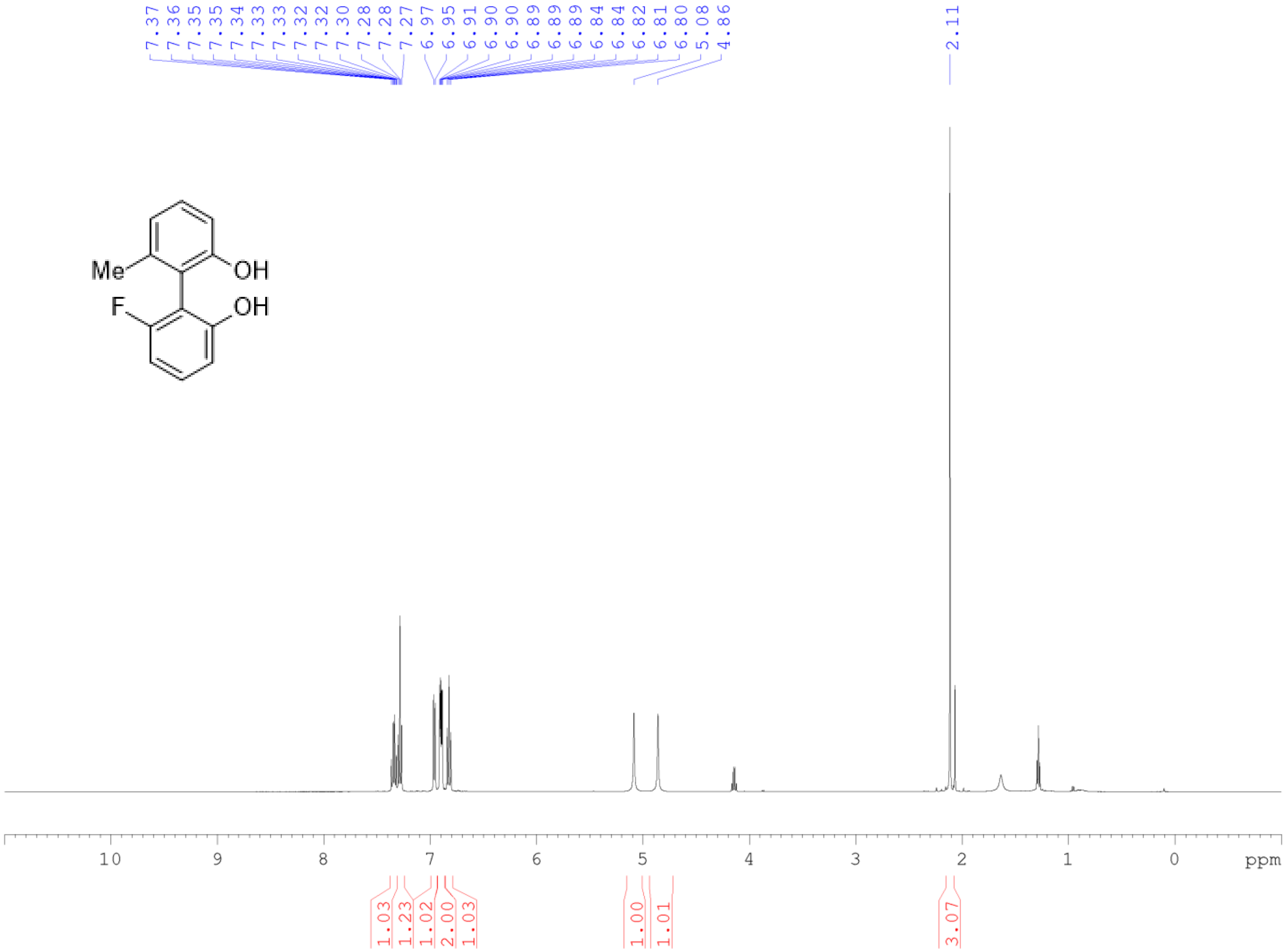

**$^{13}$C NMR** ($CDCl_3$): 6-fluoro-6'-methyl-[1,1'-biphenyl]-2,2'-diol (**3a**)

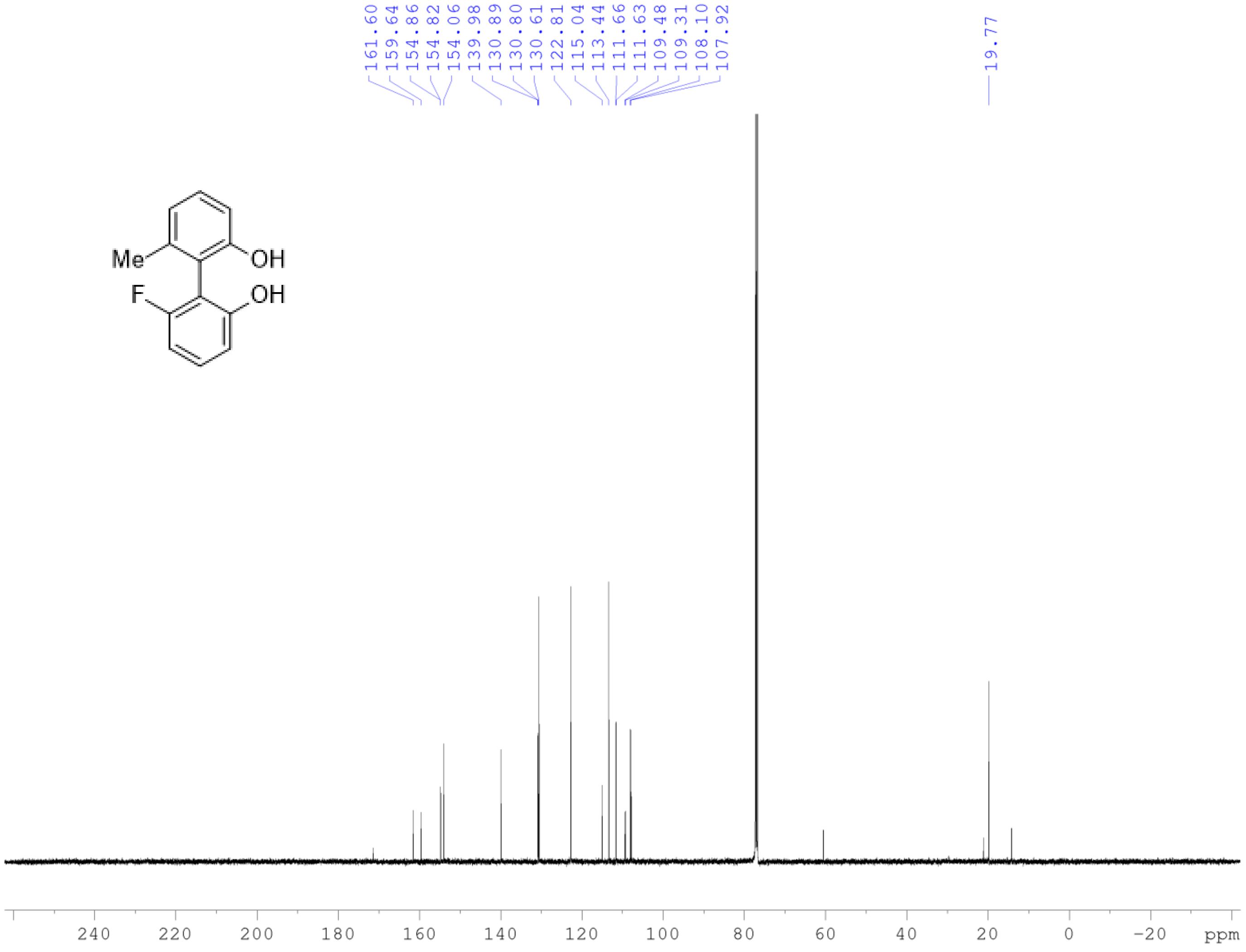

**$^{19}$F NMR** ($CDCl_3$): 6-fluoro-6'-methyl-[1,1'-biphenyl]-2,2'-diol (**3a**)

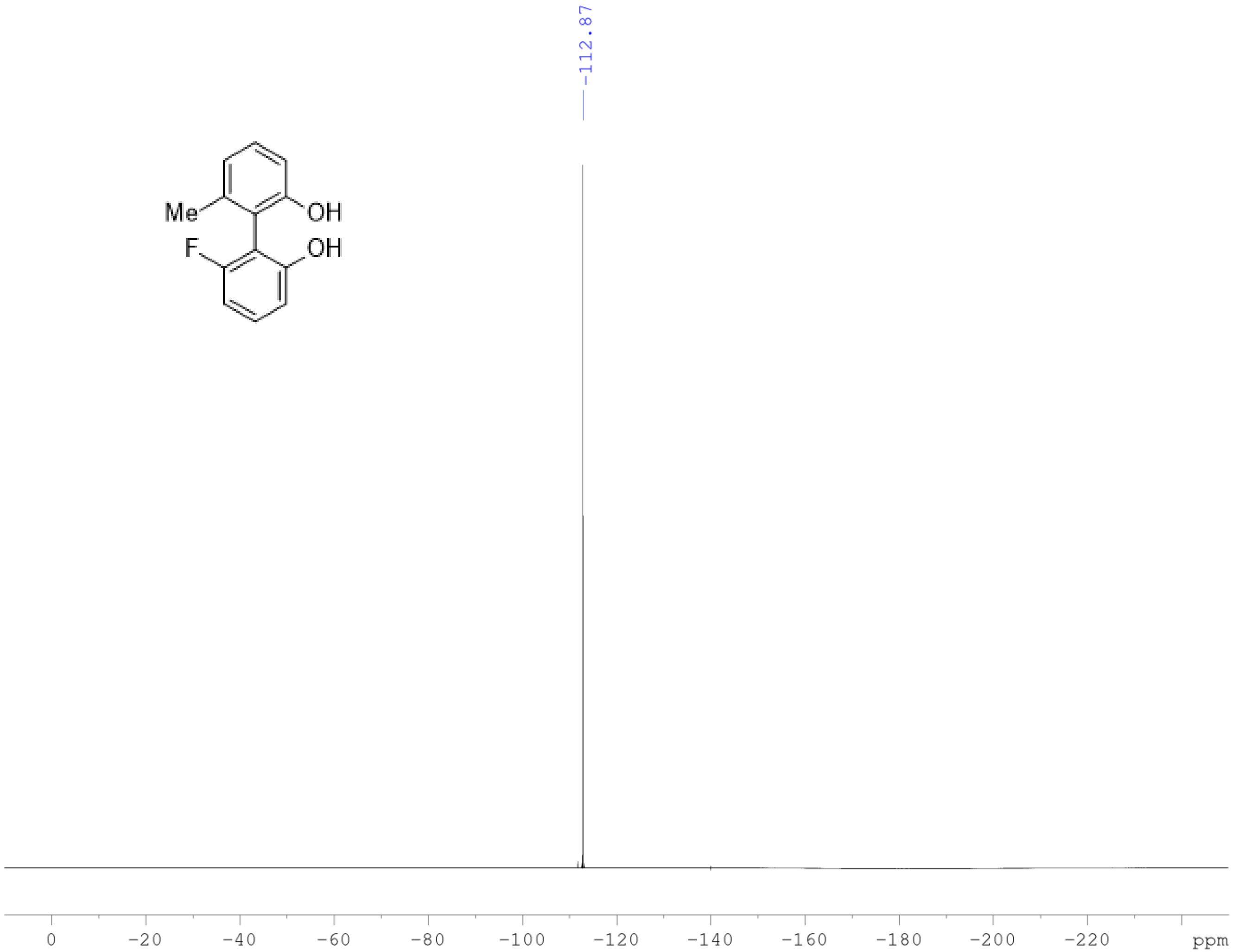


**$^{1}$H NMR** ($CDCl_3$)**:** 3-chloro-6'-fluoro-6-methyl-[1,1'-biphenyl]-2,2'-diol (**3b**)

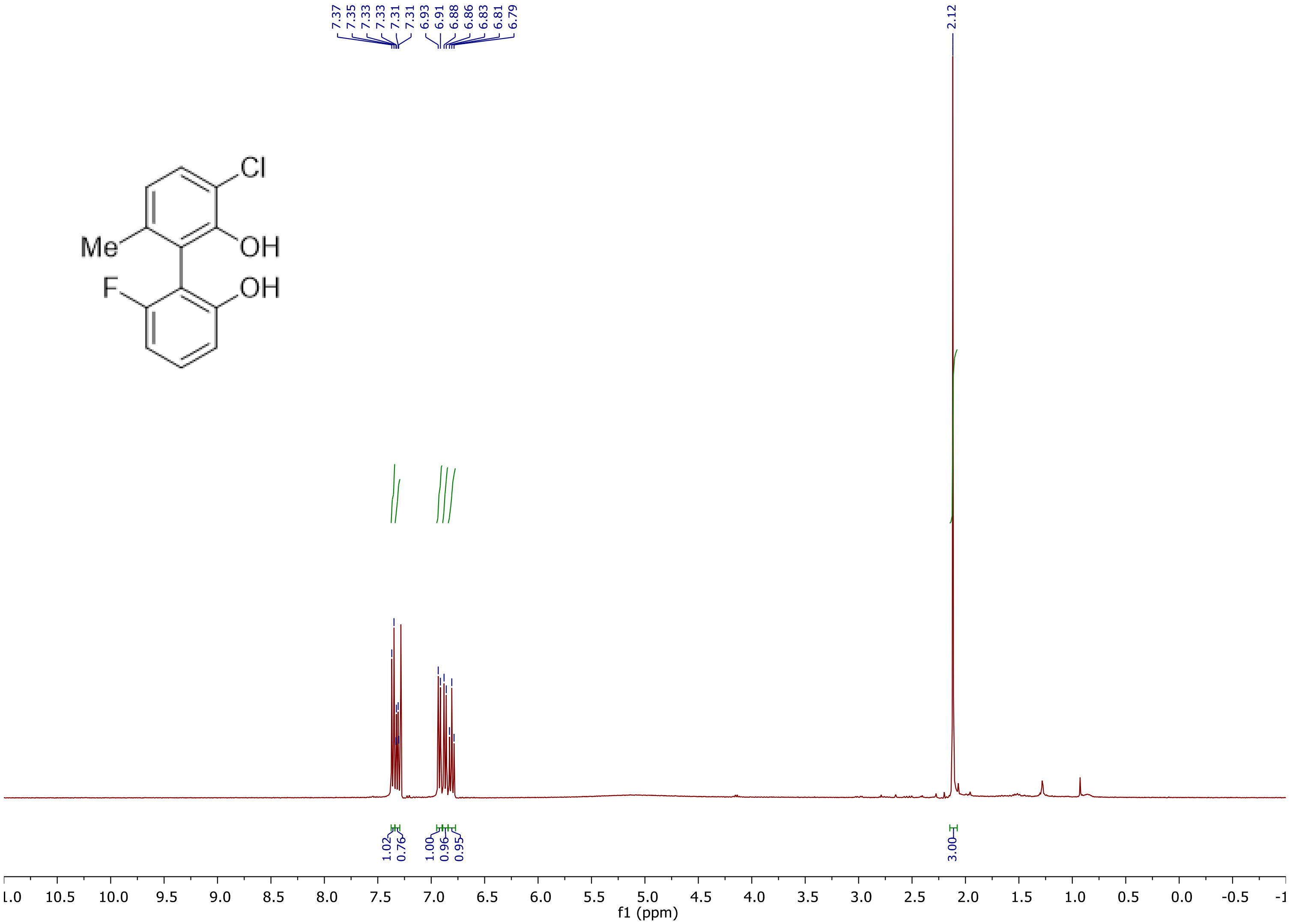


**$^{13}$C NMR** (CDCl$_3$)**:** 3-chloro-6'-fluoro-6-methyl-[1,1'-biphenyl]-2,2'-diol (**3b**)

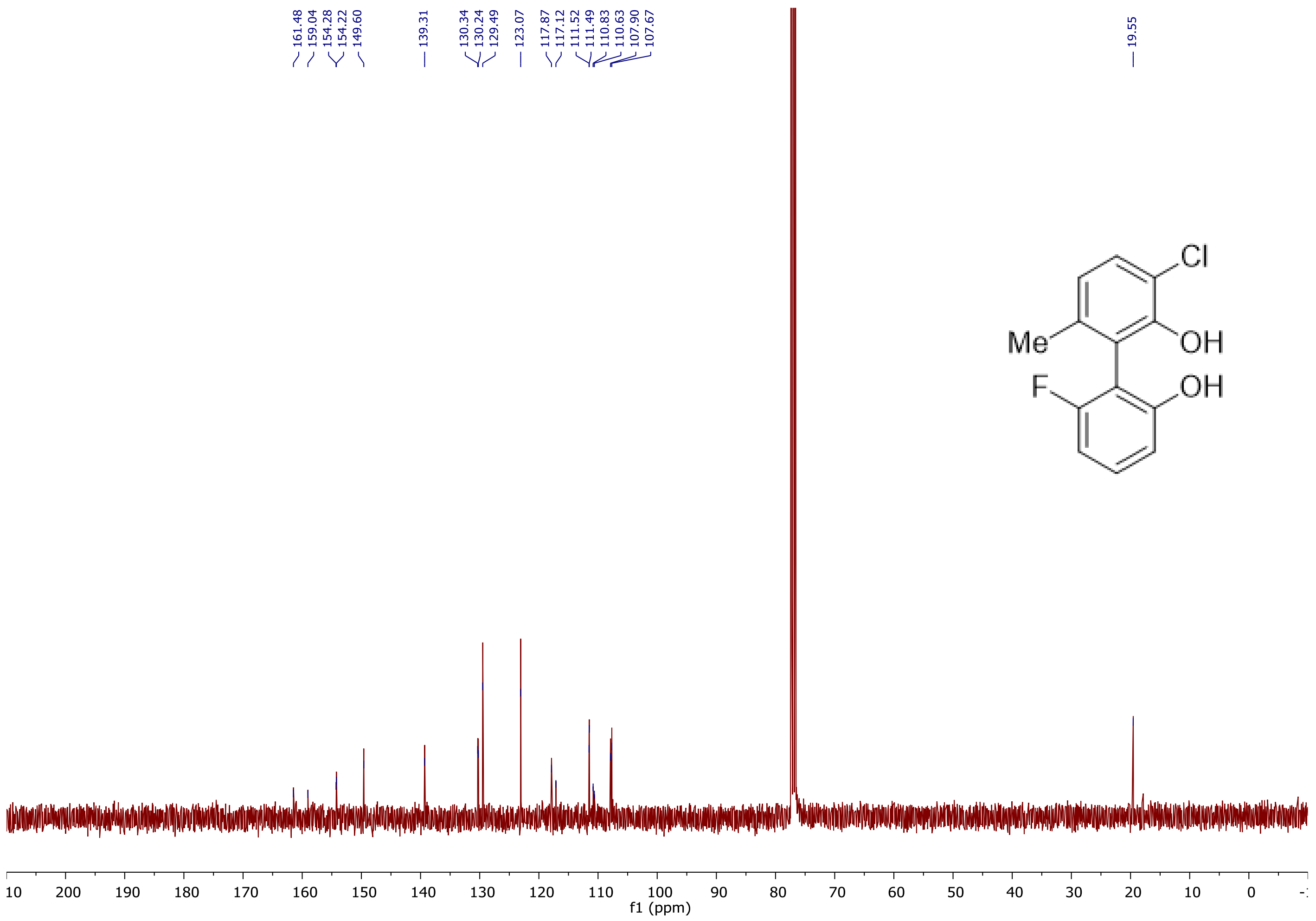


**$^{19}$F NMR** ($CDCl_3$)**:** 3-chloro-6'-fluoro-6-methyl-[1,1'-biphenyl]-2,2'-diol (**3b**)

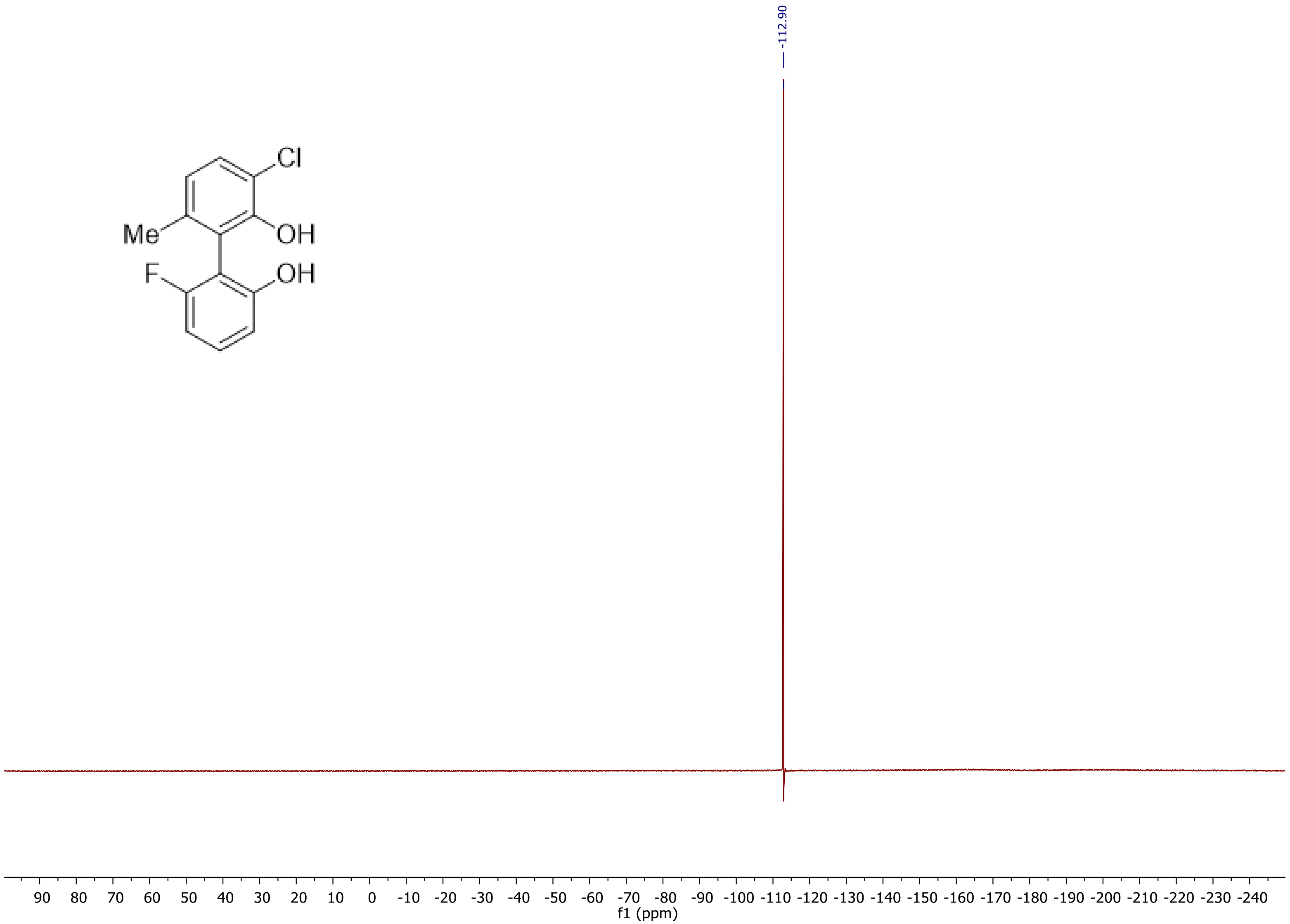


**$^{1}$H NMR** (CDCl$_3$)**:** 6'-fluoro-4,6-dimethyl-[1,1'-biphenyl]-2,2'-diol (**3c**)

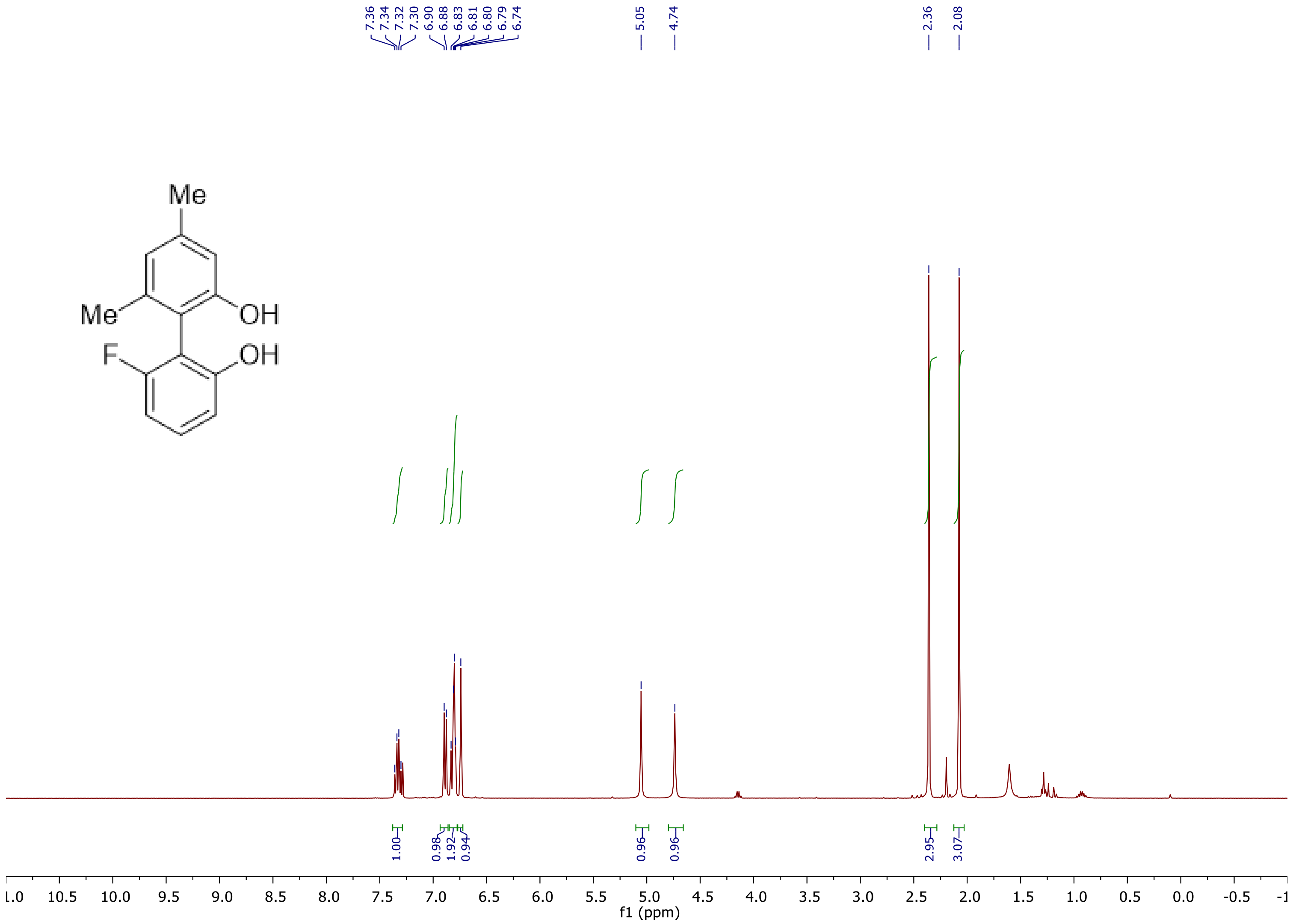

**$^{13}$C NMR** (CDCl$_3$)**:** 6'-fluoro-4,6-dimethyl-[1,1'-biphenyl]-2,2'-diol (**3c**)

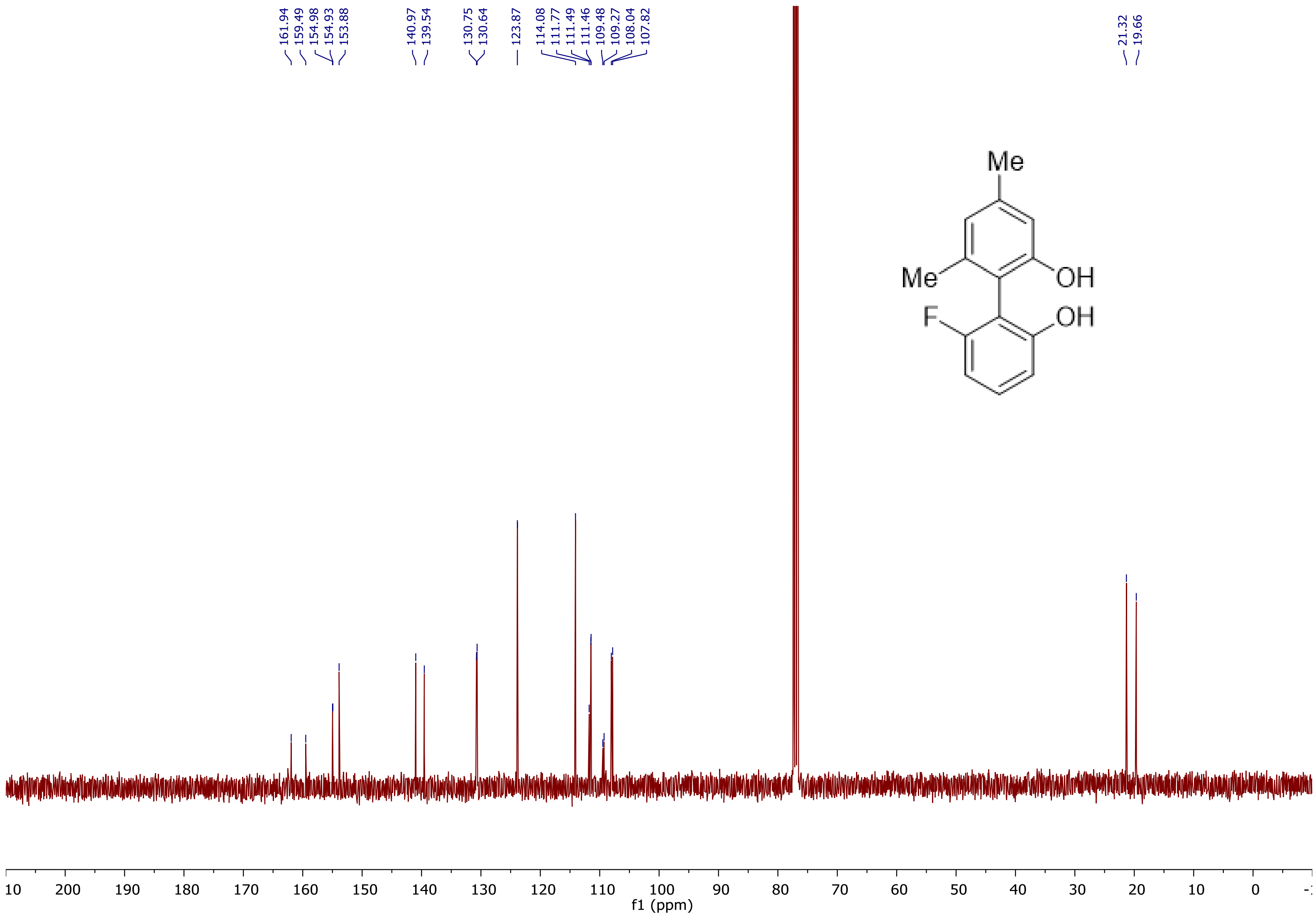


**$^{19}$F NMR** ($CDCl_3$)**:** 6'-fluoro-4,6-dimethyl-[1,1'-biphenyl]-2,2'-diol (**3c**)

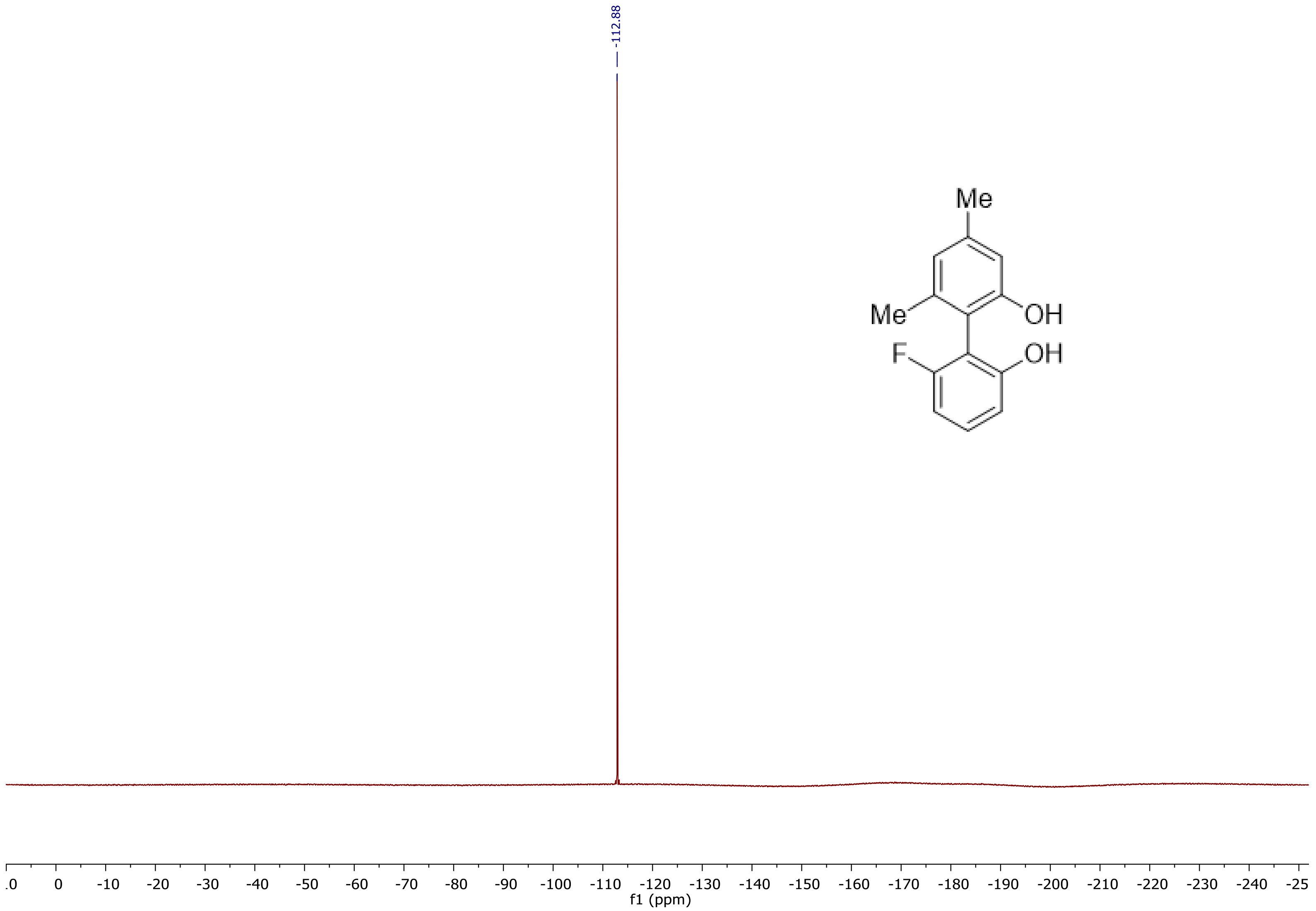


**$^{1}$H NMR** ($CDCl_3$)**:** 1-(2-fluoro-6-hydroxyphenyl)-5,6,7,8-tetrahydronaphthalen-2-ol (**3d**)

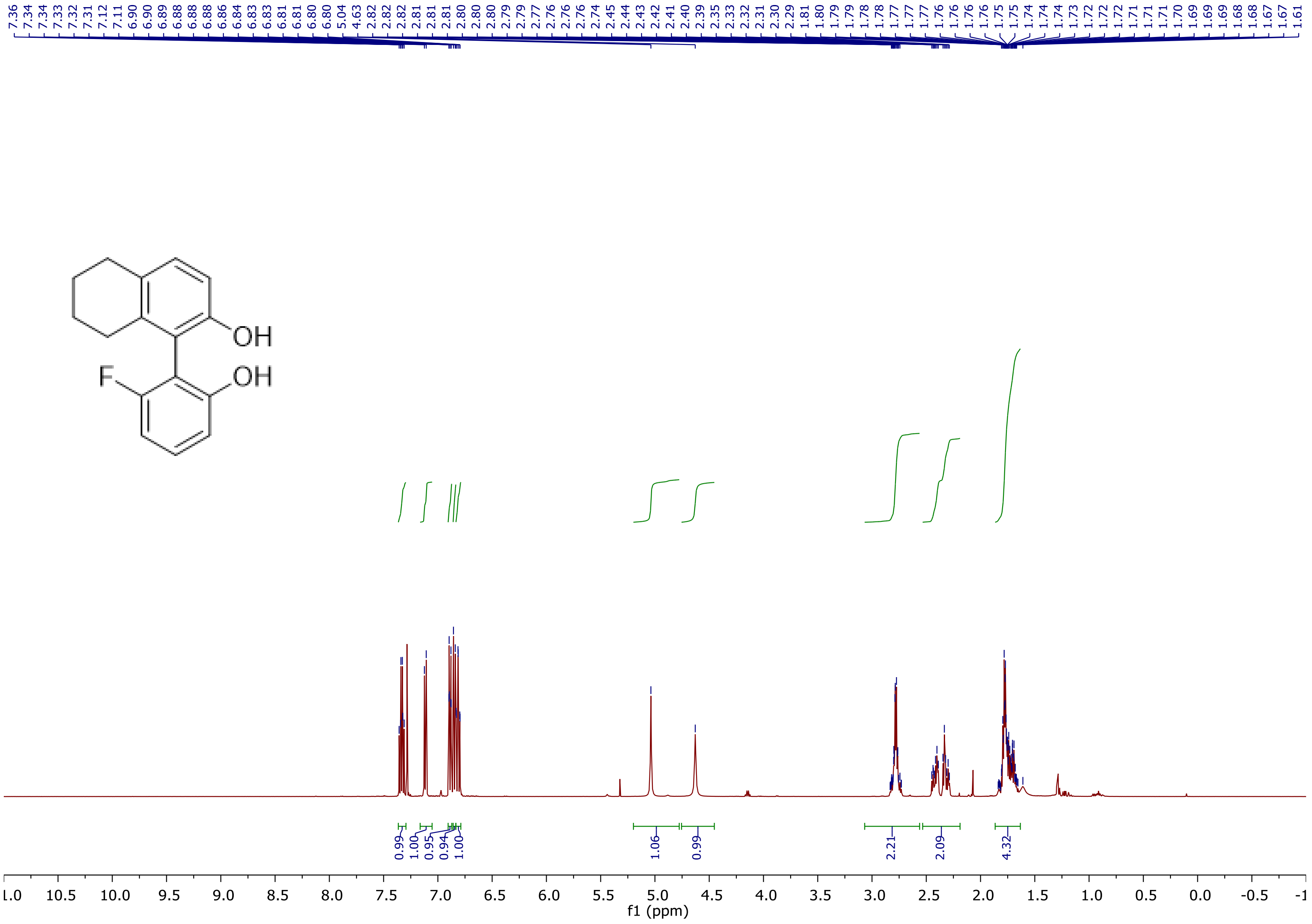


**$^{13}$C NMR** (CDCl$_3$)**:** 1-(2-fluoro-6-hydroxyphenyl)-5,6,7,8-tetrahydronaphthalen-2-ol (**3d**)

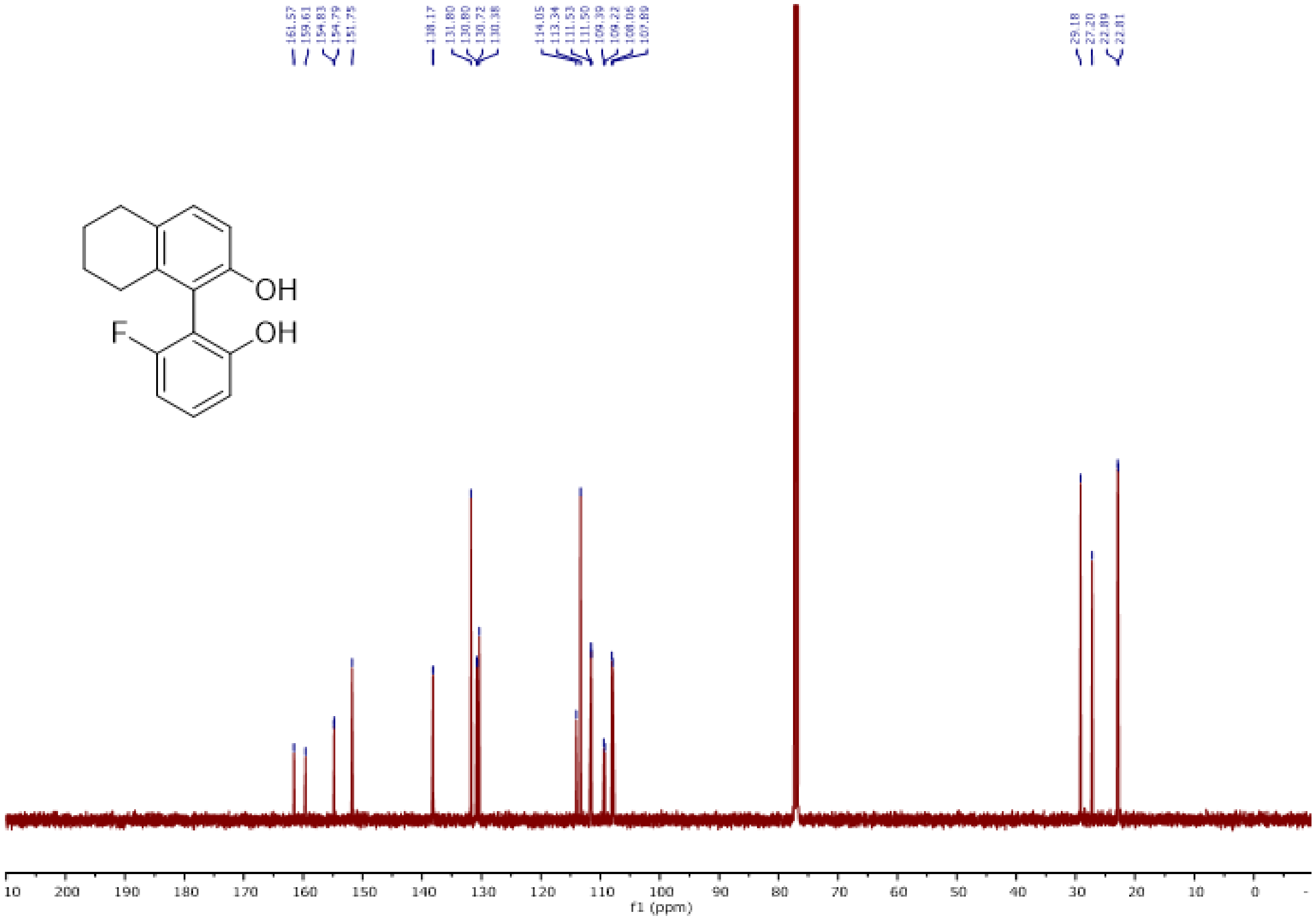


**$^{19}$F NMR** ($CDCl_3$)**:** 1-(2-fluoro-6-hydroxyphenyl)-5,6,7,8-tetrahydronaphthalen-2-ol (**3d**)

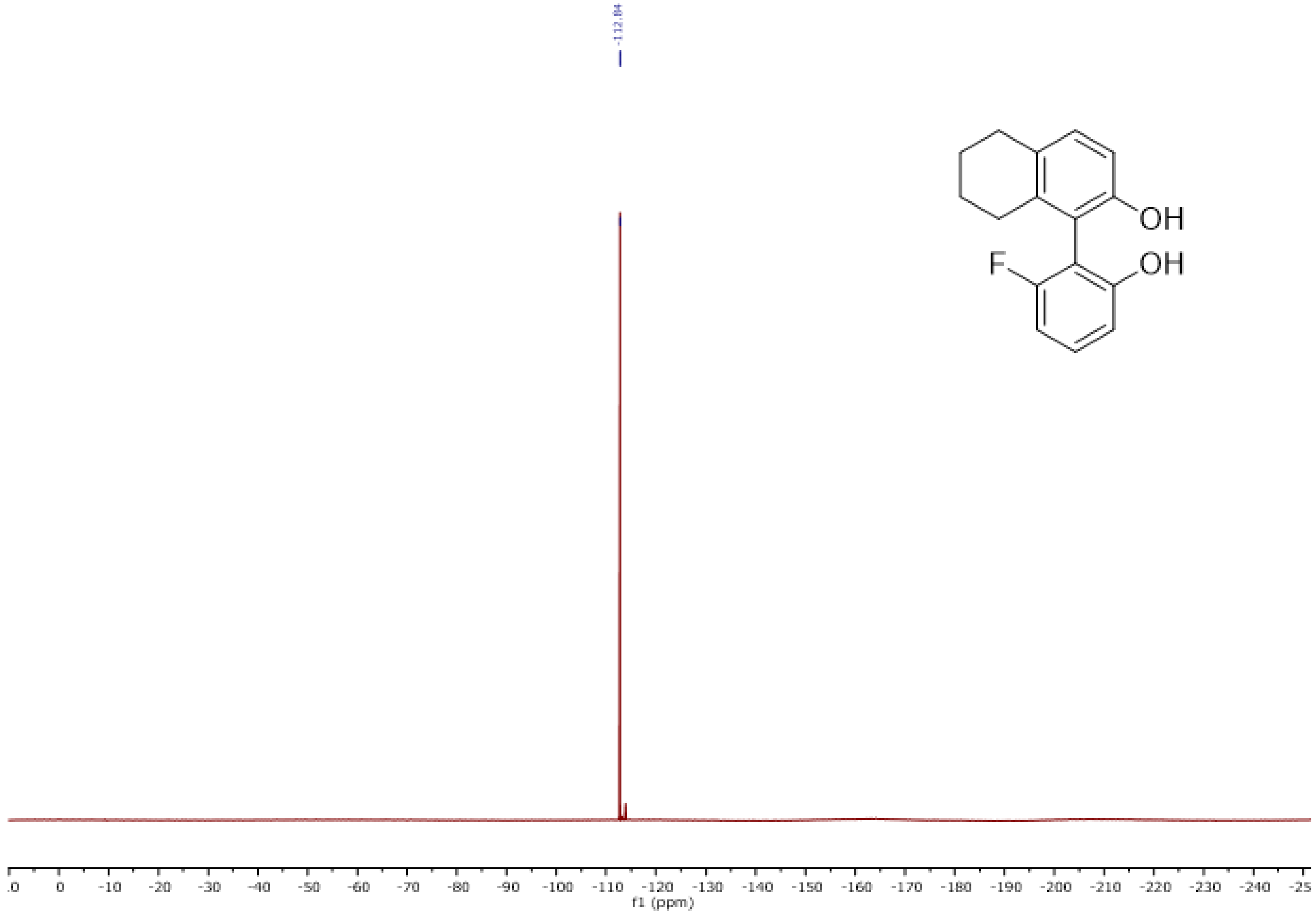


**$^{1}$H NMR** ($CDCl_3$)**:** 6-chloro-6'-fluoro-[1,1'-biphenyl]-2,2'-diol (**3e**)

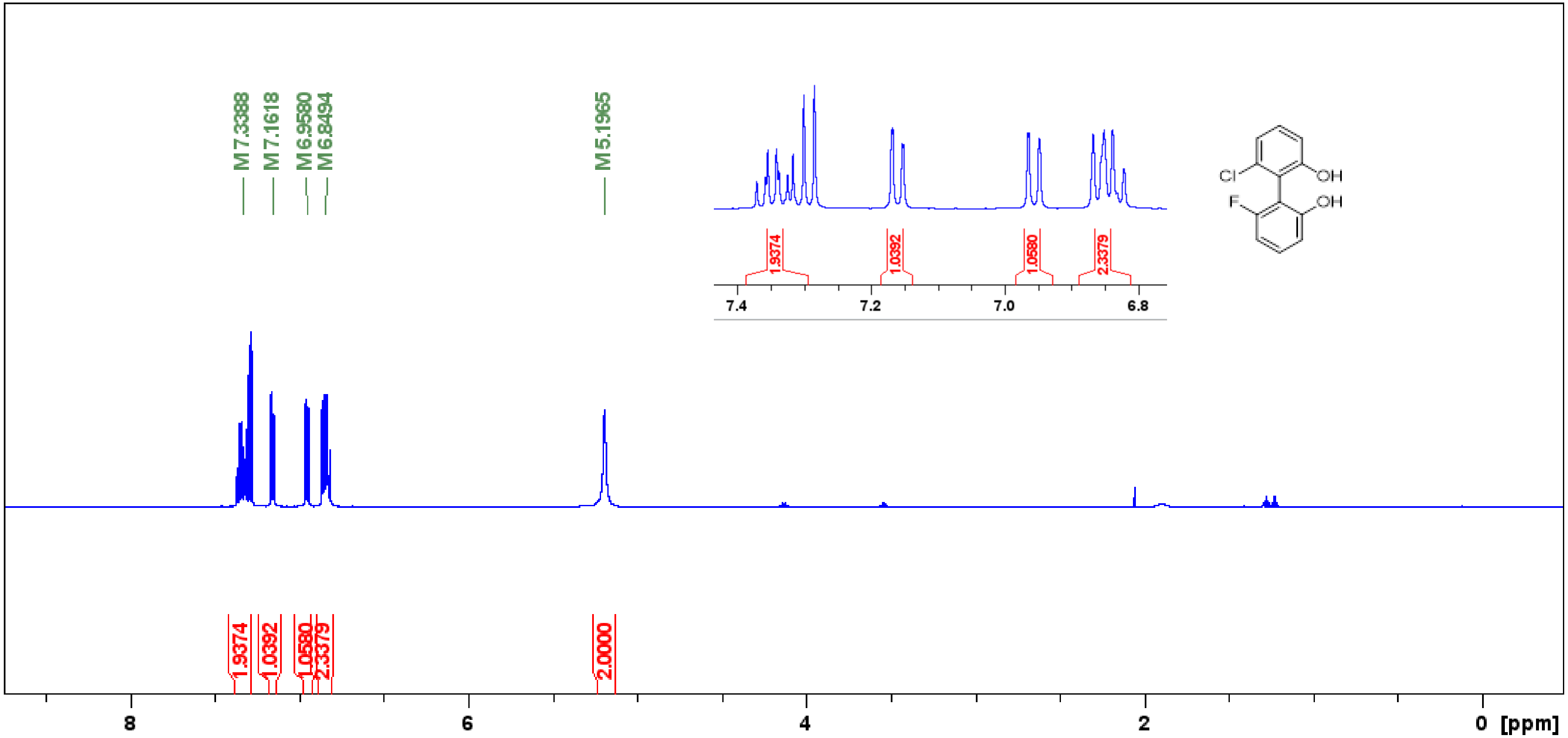

M7.3388
M7.1618
M6.9580
M6.8494
M5.1965
1.9374
1.0392
1.0580
2.3379
7.4
7.2
7.0
6.8
Cl
OH
F
OH
1.9374
1.0392
1.0580
2.3379
2.0000
8
6
4
2
0 [ppm]

**$^{13}$C NMR** ($CDCl_3$)**:** 6-chloro-6'-fluoro-[1,1'-biphenyl]-2,2'-diol (**3e**)

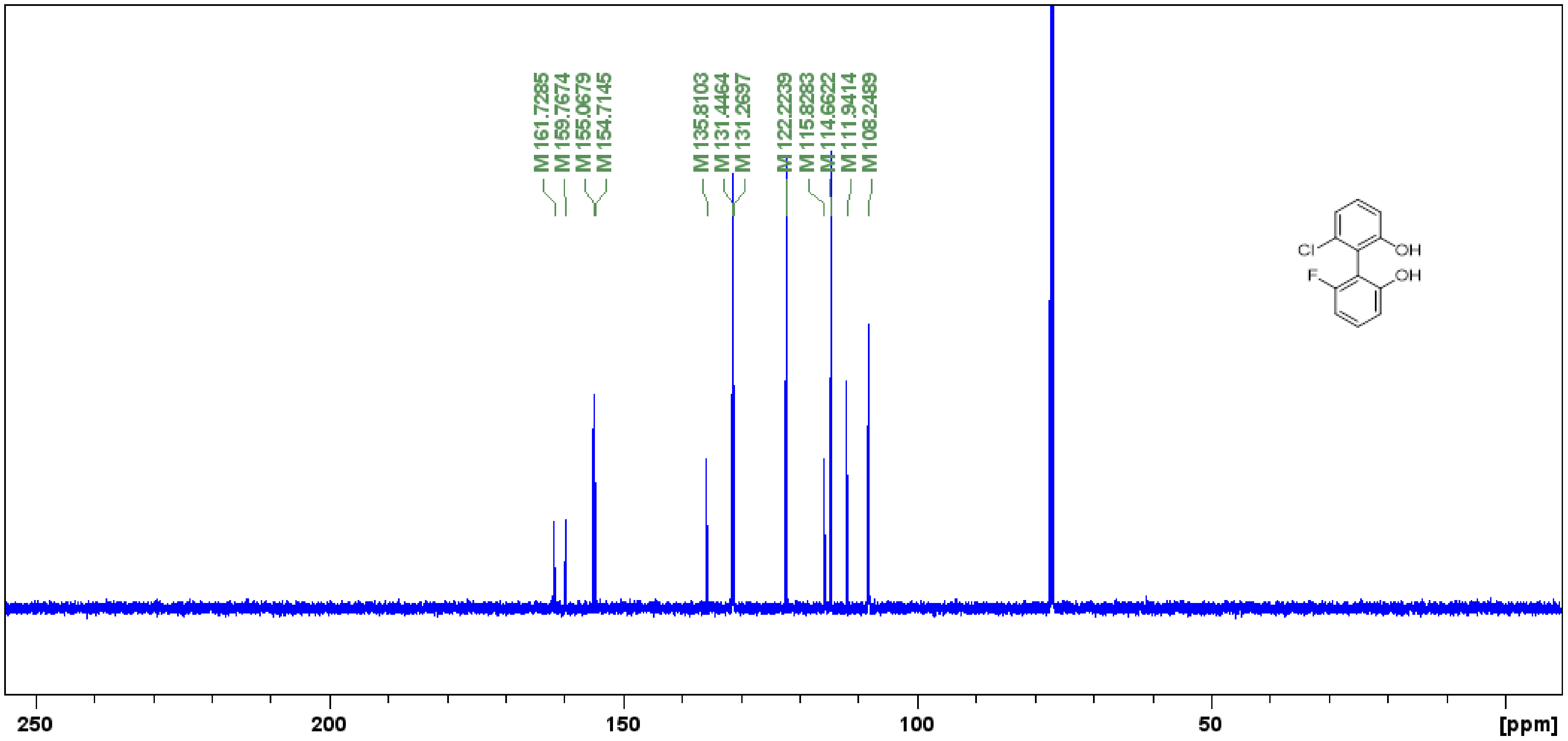

**$^{19}$F NMR** ($CDCl_3$)**:** 6-chloro-6'-fluoro-[1,1'-biphenyl]-2,2'-diol (**3e**)

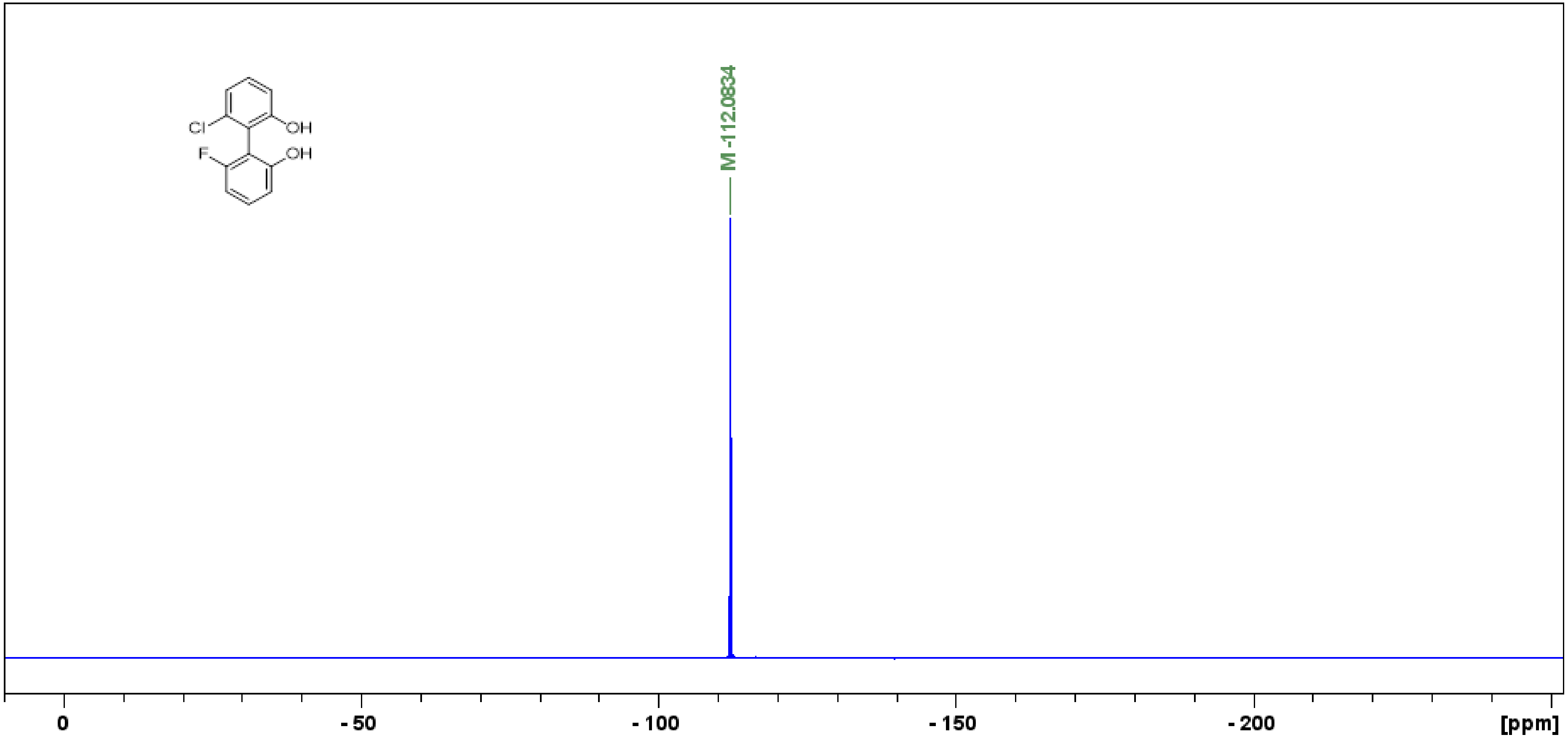


**$^{1}$H NMR** ($CDCl_3$)**:** 6-chloro-6'-fluoro-5-methyl-[1,1'-biphenyl]-2,2'-diol (**3f**)

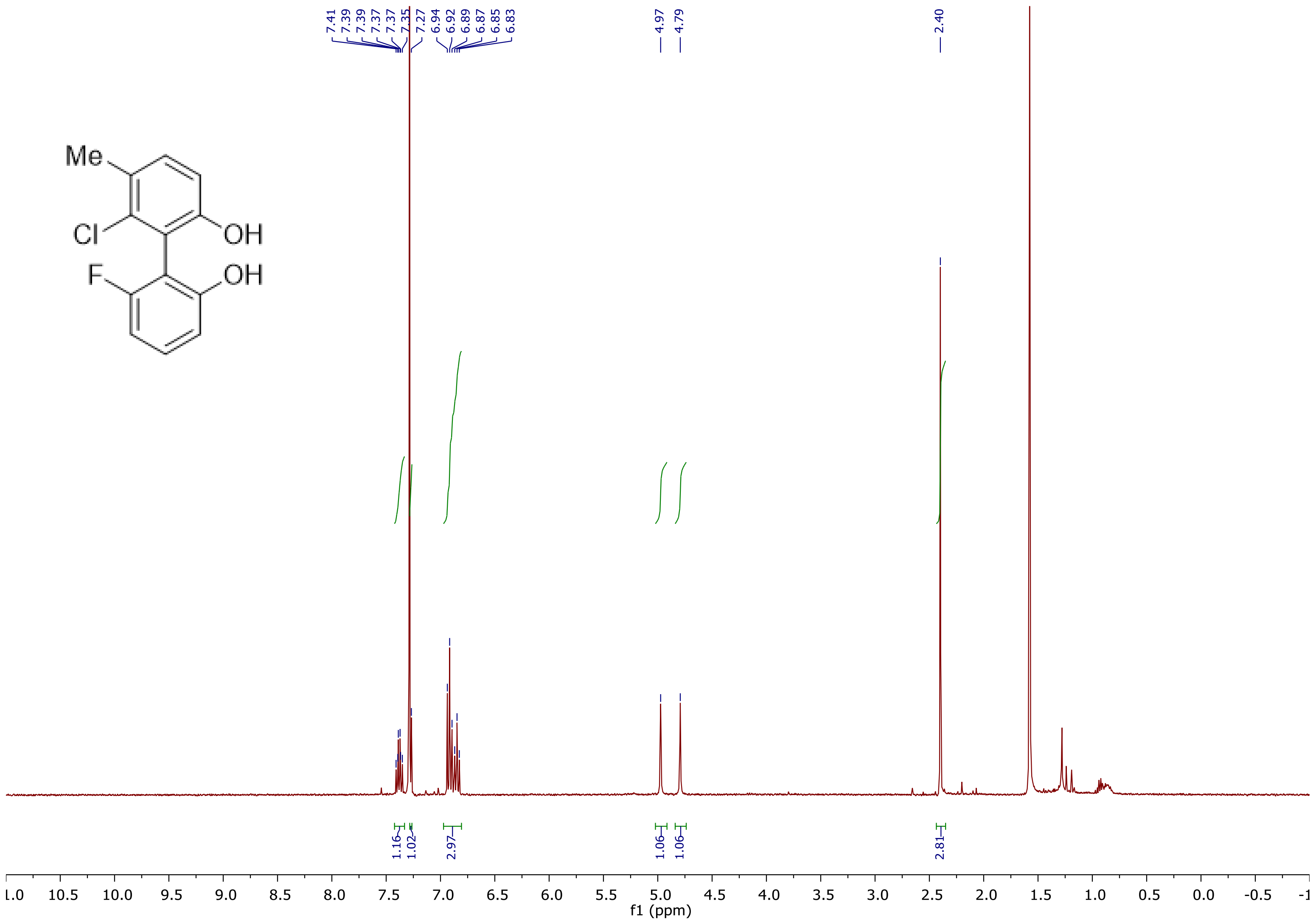


**$^{13}$C NMR** (CDCl$_3$)**:** 6-chloro-6'-fluoro-5-methyl-[1,1'-biphenyl]-2,2'-diol (**3f**)

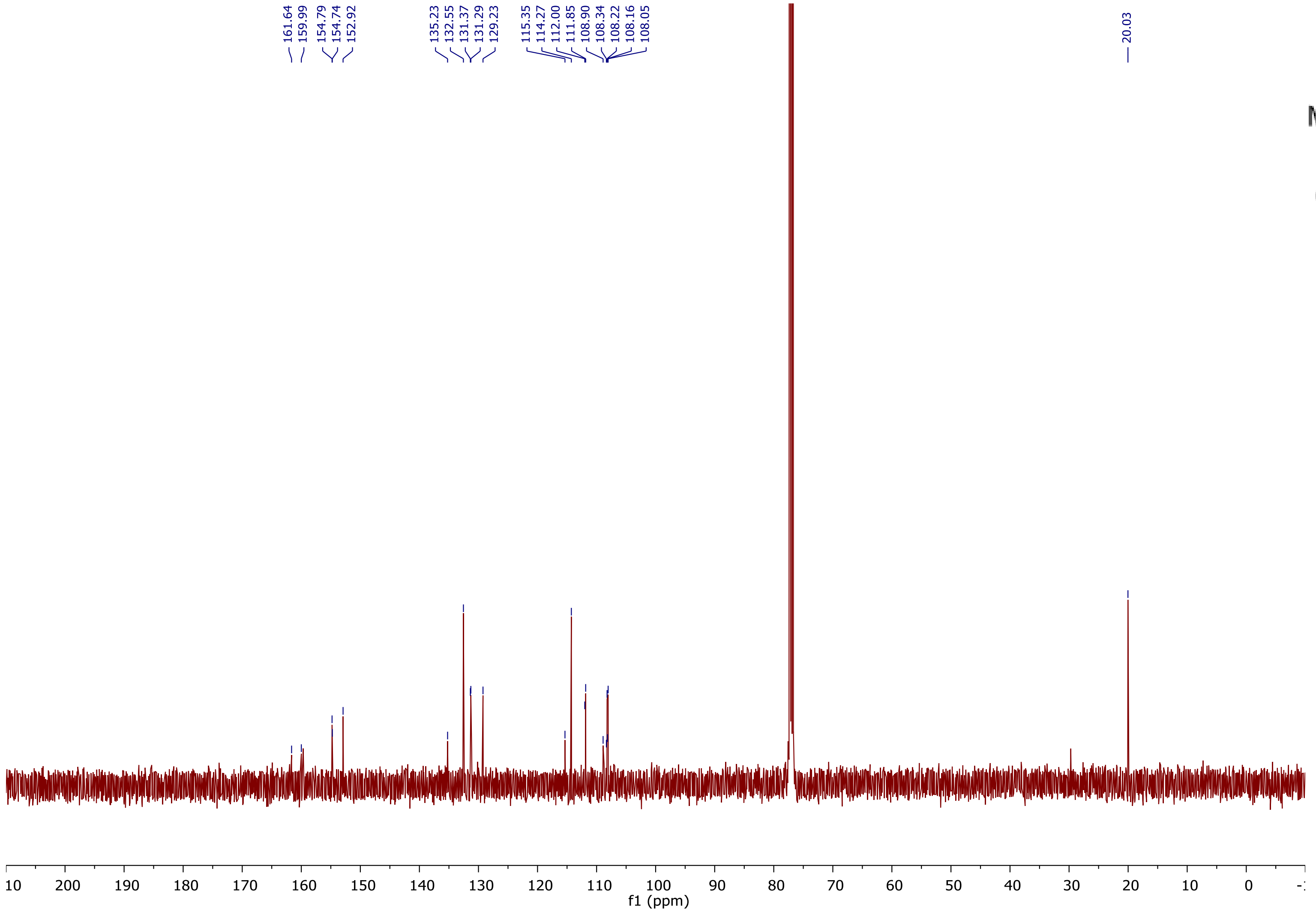
161.64
159.99
154.79
154.74
152.92
135.23
132.55
131.37
131.29
129.23
115.35
114.27
112.00
111.85
108.90
108.34
108.22
108.16
108.05
20.03
f1 (ppm)

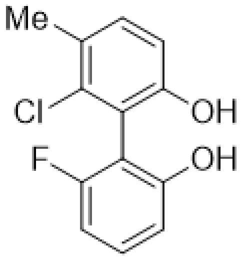
Me
Cl
OH
F
OH

**$^{19}$F NMR** ($CDCl_3$)**:** 6-chloro-6'-fluoro-5-methyl-[1,1'-biphenyl]-2,2'-diol (**3f**)

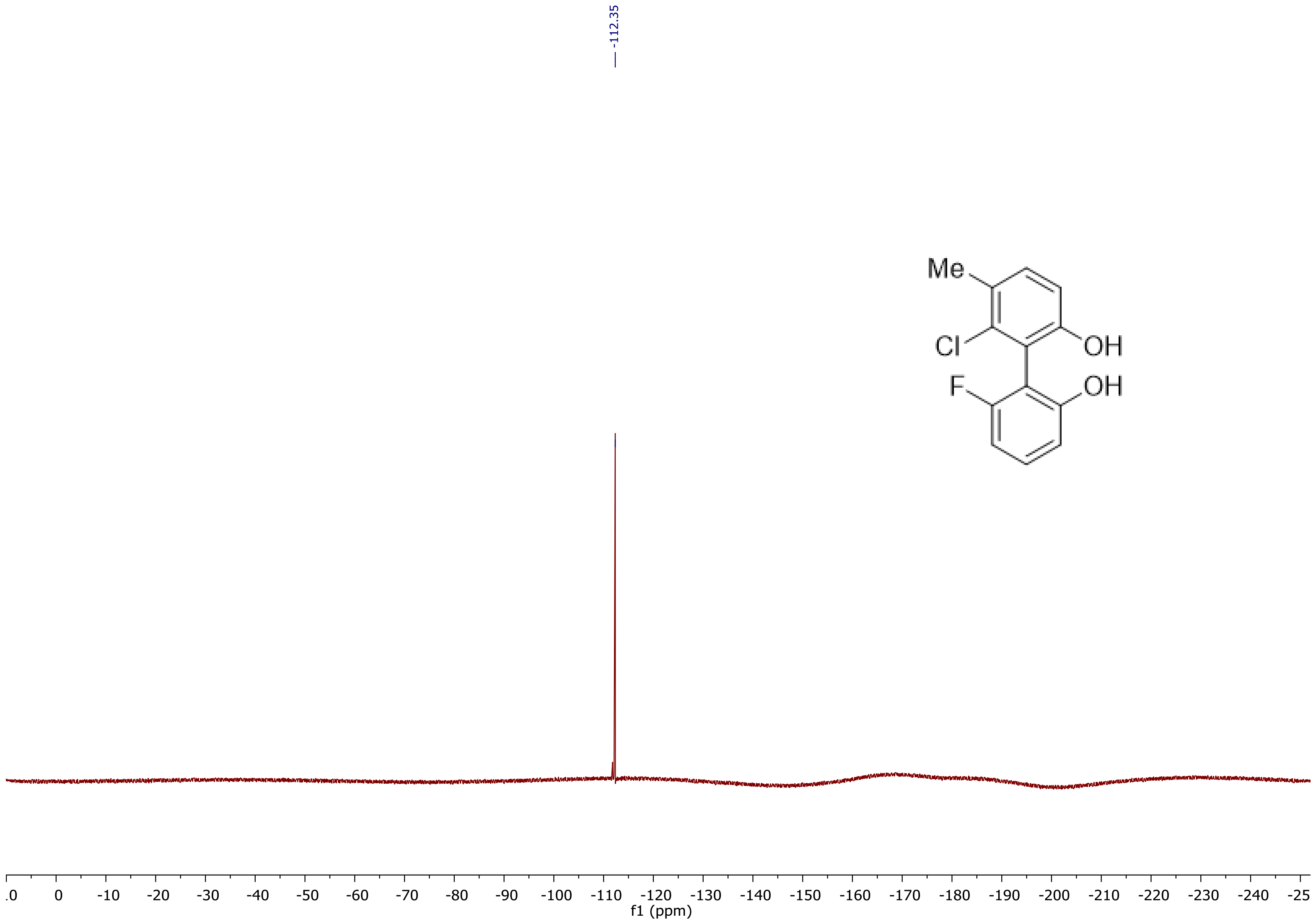

**$^1$H NMR** (CDCl$_3$)**:** 4,6-dichloro-6'-fluoro-[1,1'-biphenyl]-2,2'-diol (**3g**)

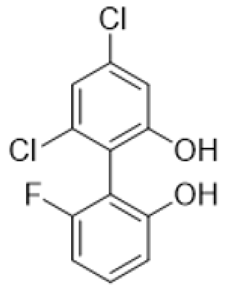


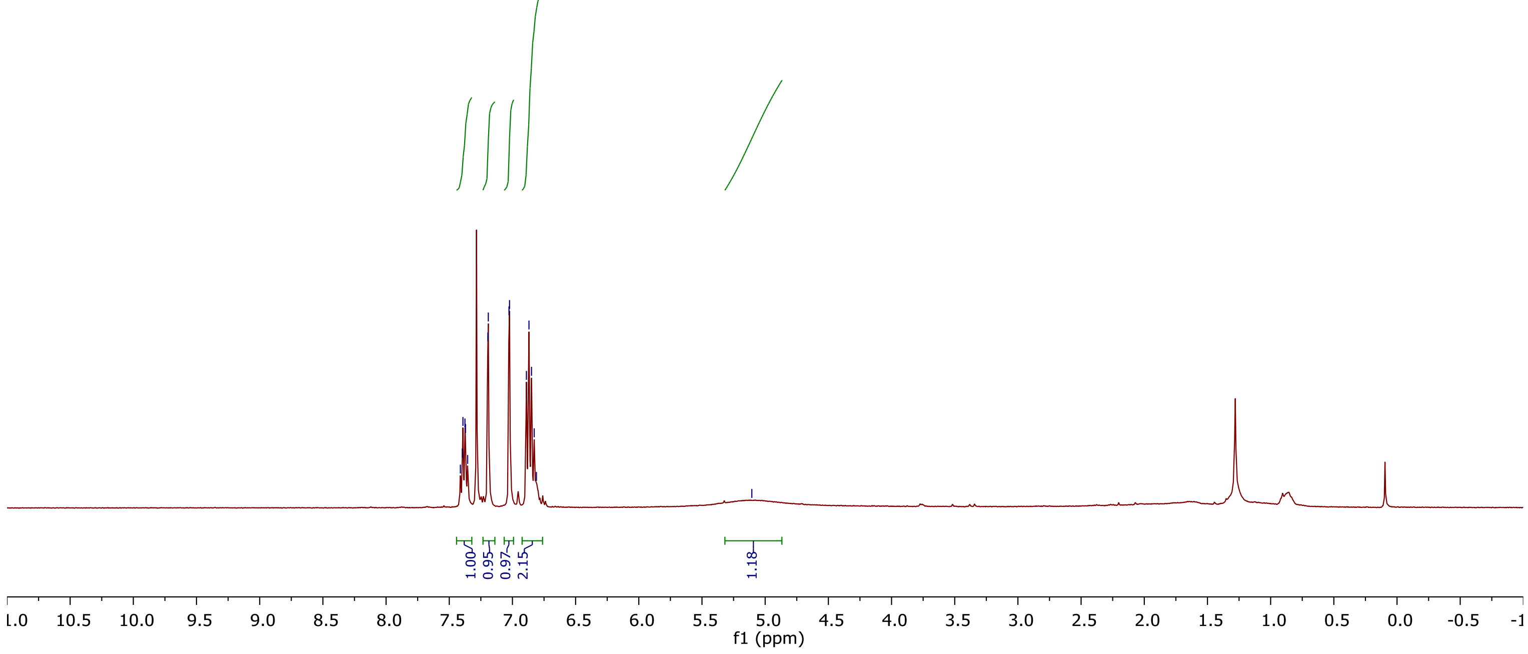

**$^{13}$C NMR** (CDCl$_3$)**:** 4,6-dichloro-6'-fluoro-[1,1'-biphenyl]-2,2'-diol (**3g**)

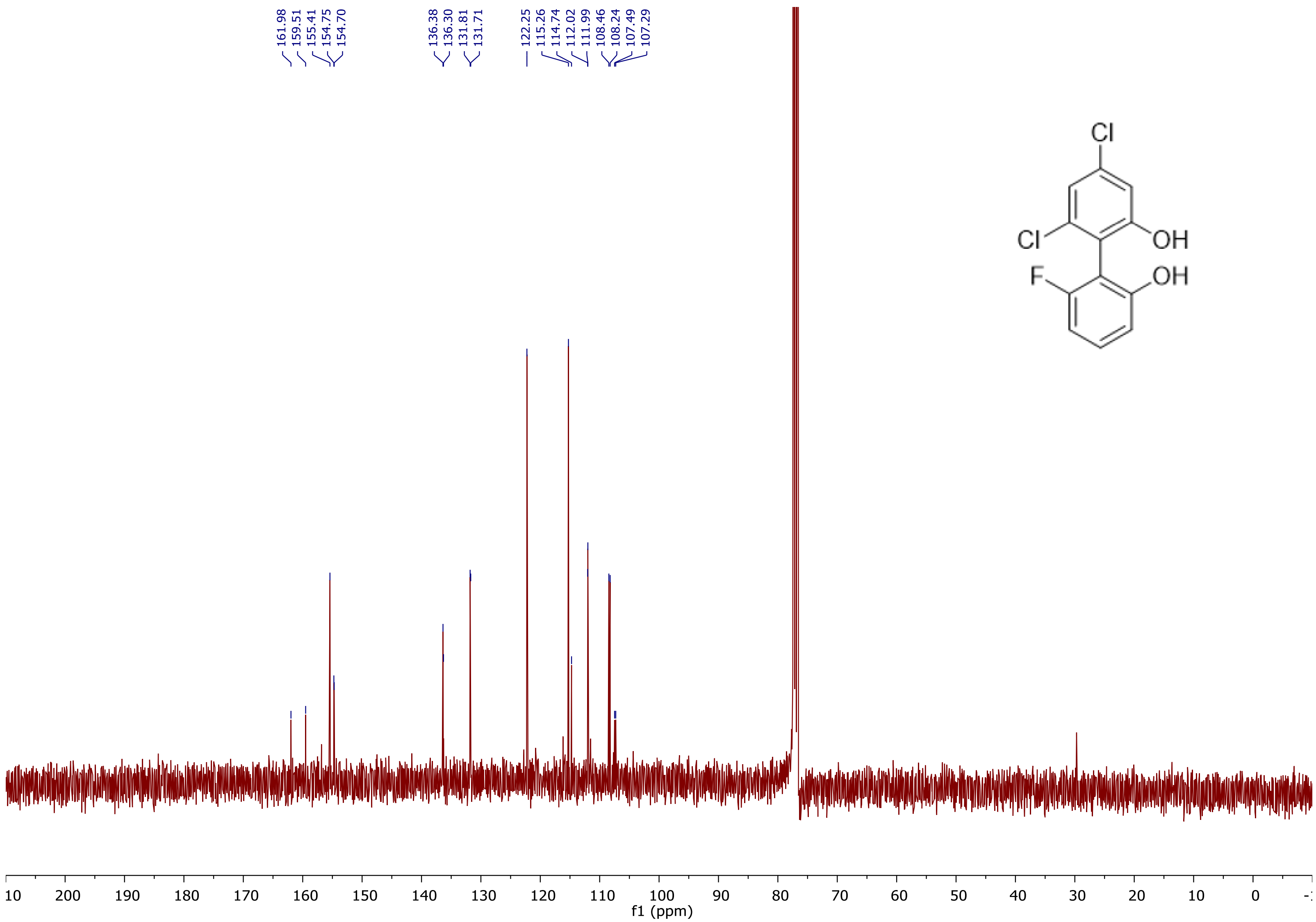

**$^{19}$F NMR** ($CDCl_3$)**:** 4,6-dichloro-6'-fluoro-[1,1'-biphenyl]-2,2'-diol (**3g**)

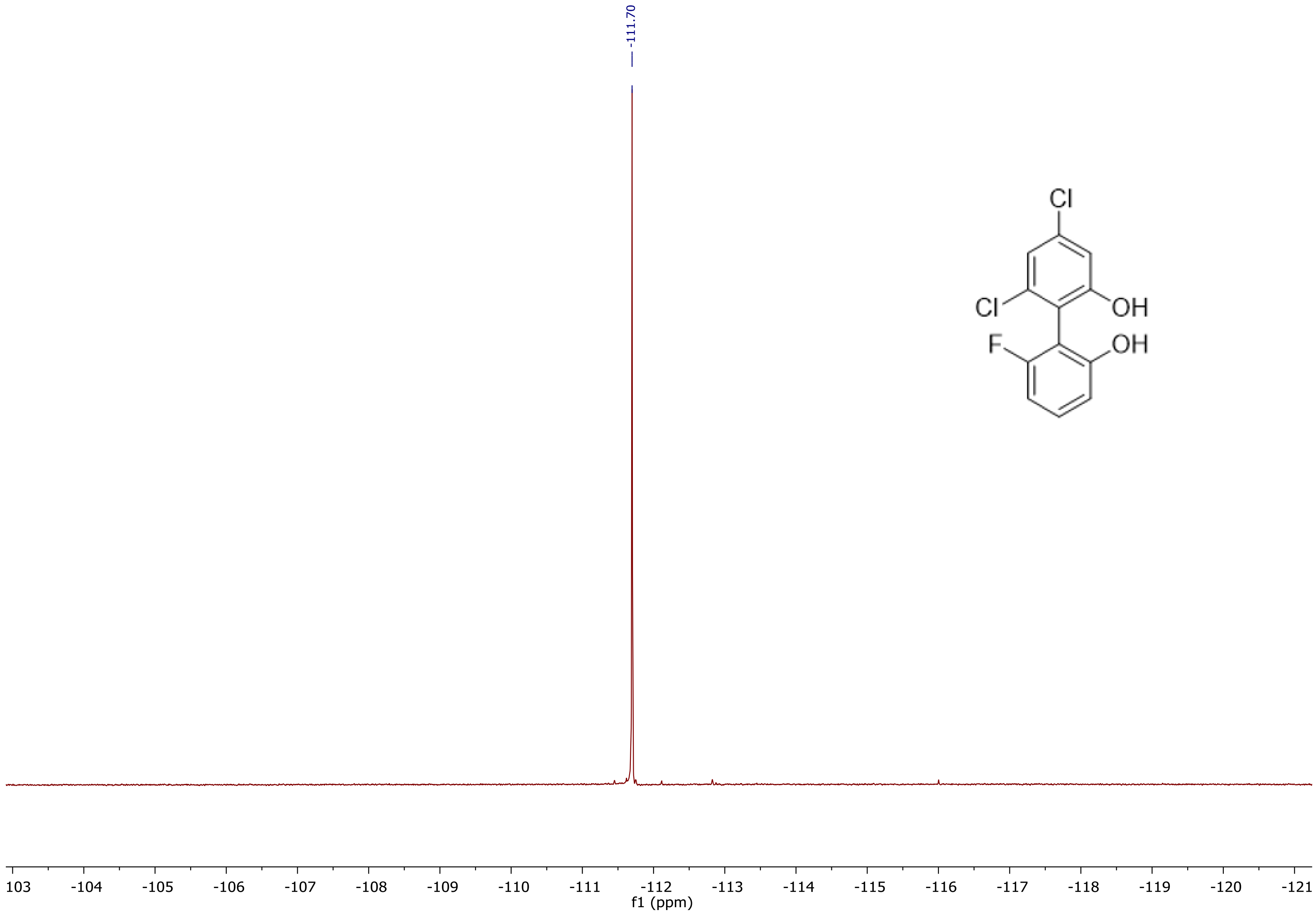

**¹H NMR** (CDCl$_3$)**:** 3,6-dichloro-6'-fluoro-[1,1'-biphenyl]-2,2'-diol (**3h**)

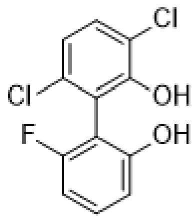


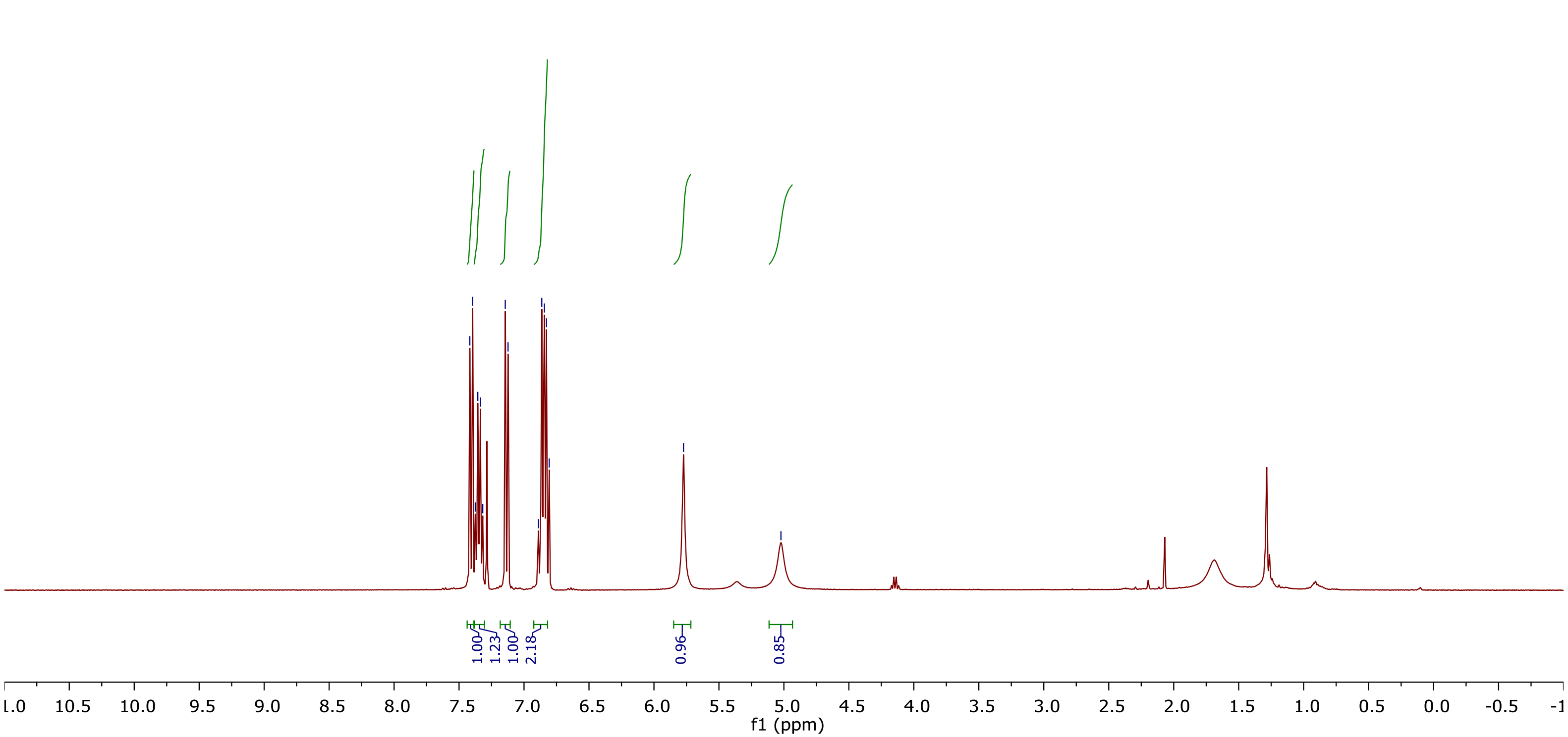

**$^{13}$C NMR** ($CDCl_3$)**:** 3,6-dichloro-6'-fluoro-[1,1'-biphenyl]-2,2'-diol (**3h**)

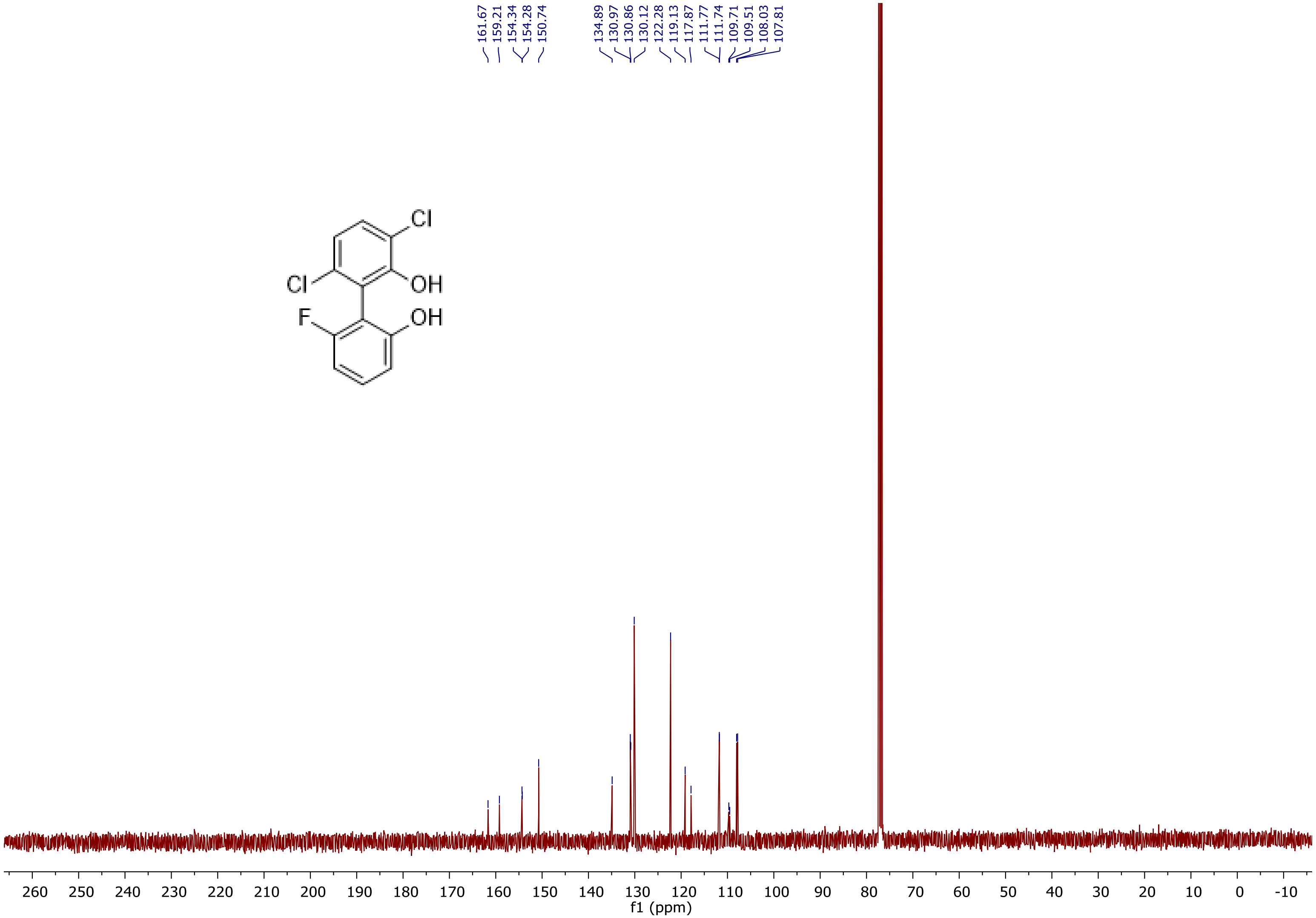

**$^{19}$F NMR** ($CDCl_3$)**:** 3,6-dichloro-6'-fluoro-[1,1'-biphenyl]-2,2'-diol (**3h**)

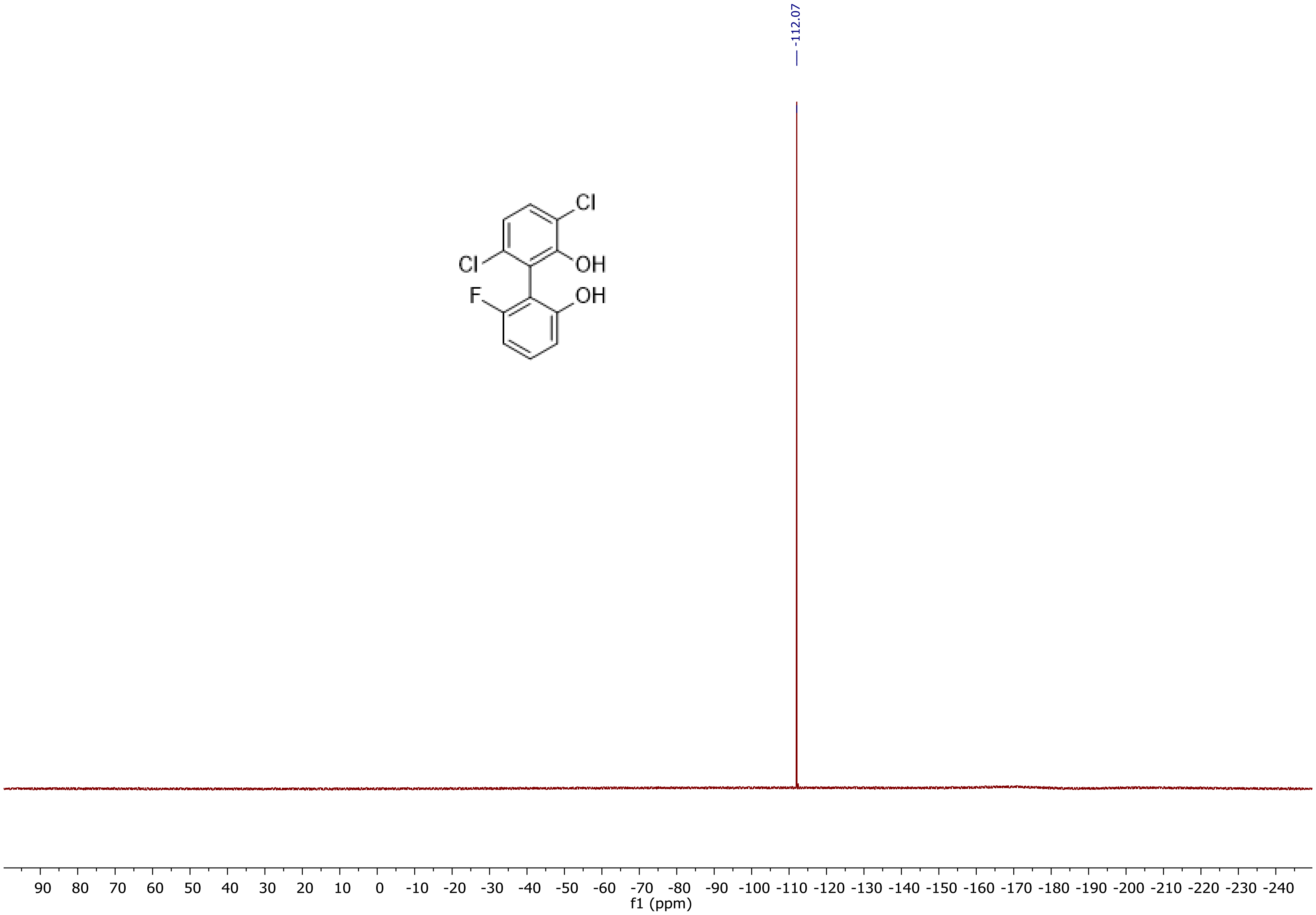

**$^{1}$H NMR** (CDCl$_3$)**:** tert-butyl (6-chloro-2'-fluoro-2,6'-dihydroxy-[1,1'-biphenyl]-3-yl)carbamate (**3i**)

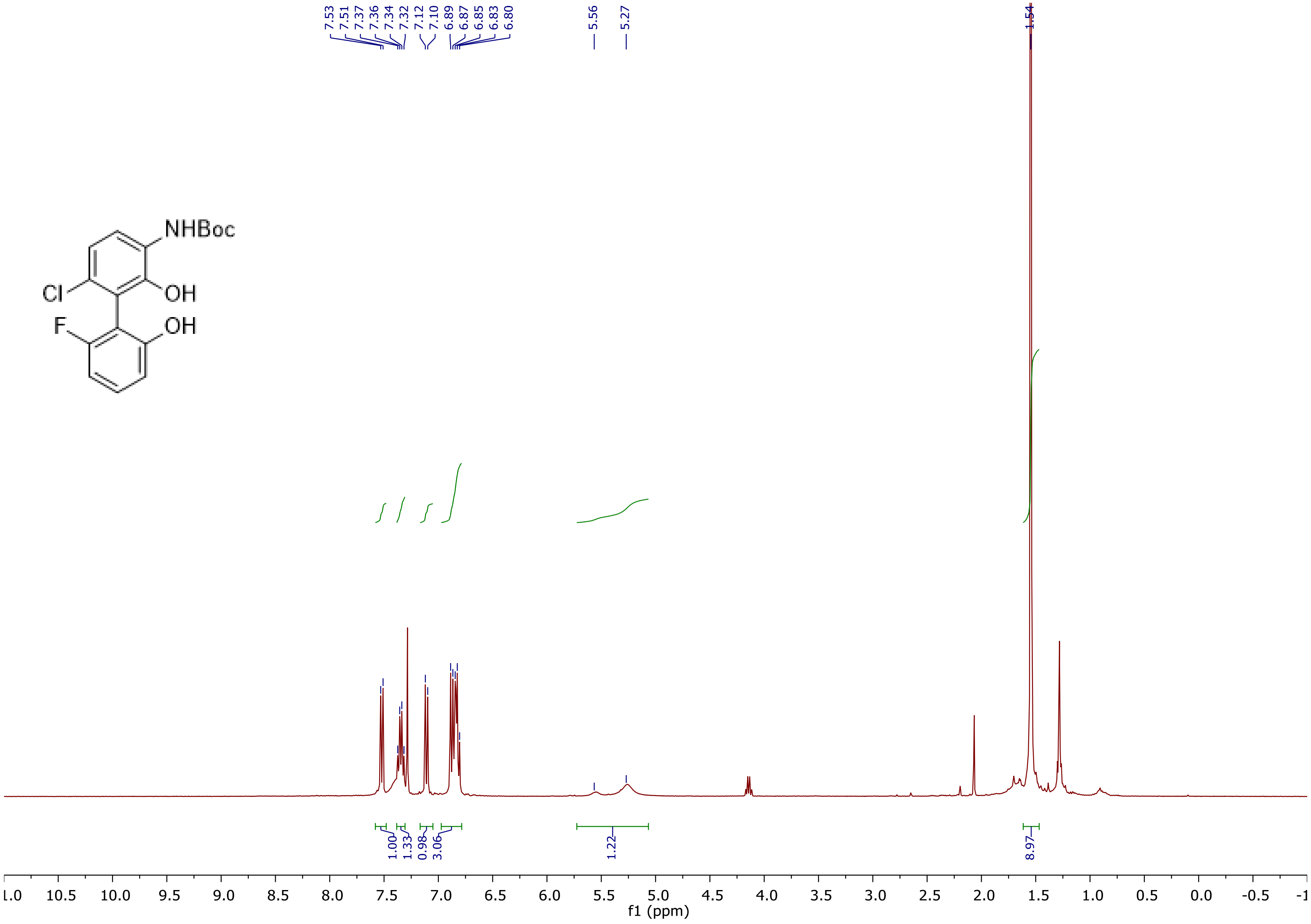

**$^{13}$C NMR** (CDCl$_3$)**:** tert-butyl (6-chloro-2'-fluoro-2,6'-dihydroxy-[1,1'-biphenyl]-3-yl)carbamate (**3i**)

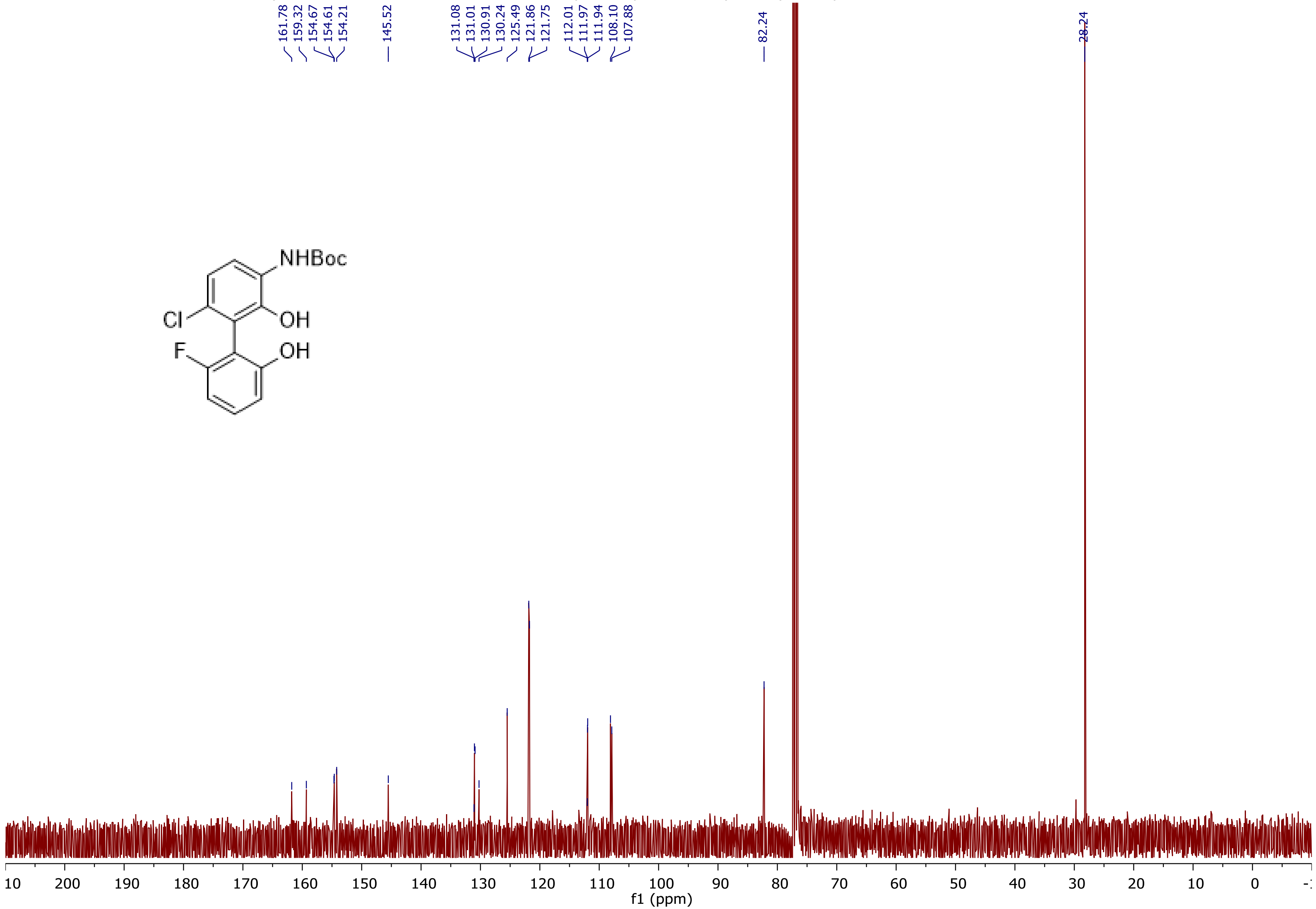

**$^{19}$F NMR** ($CDCl_3$)**:** tert-butyl (6-chloro-2'-fluoro-2,6'-dihydroxy-[1,1'-biphenyl]-3-yl)carbamate (**3i**)

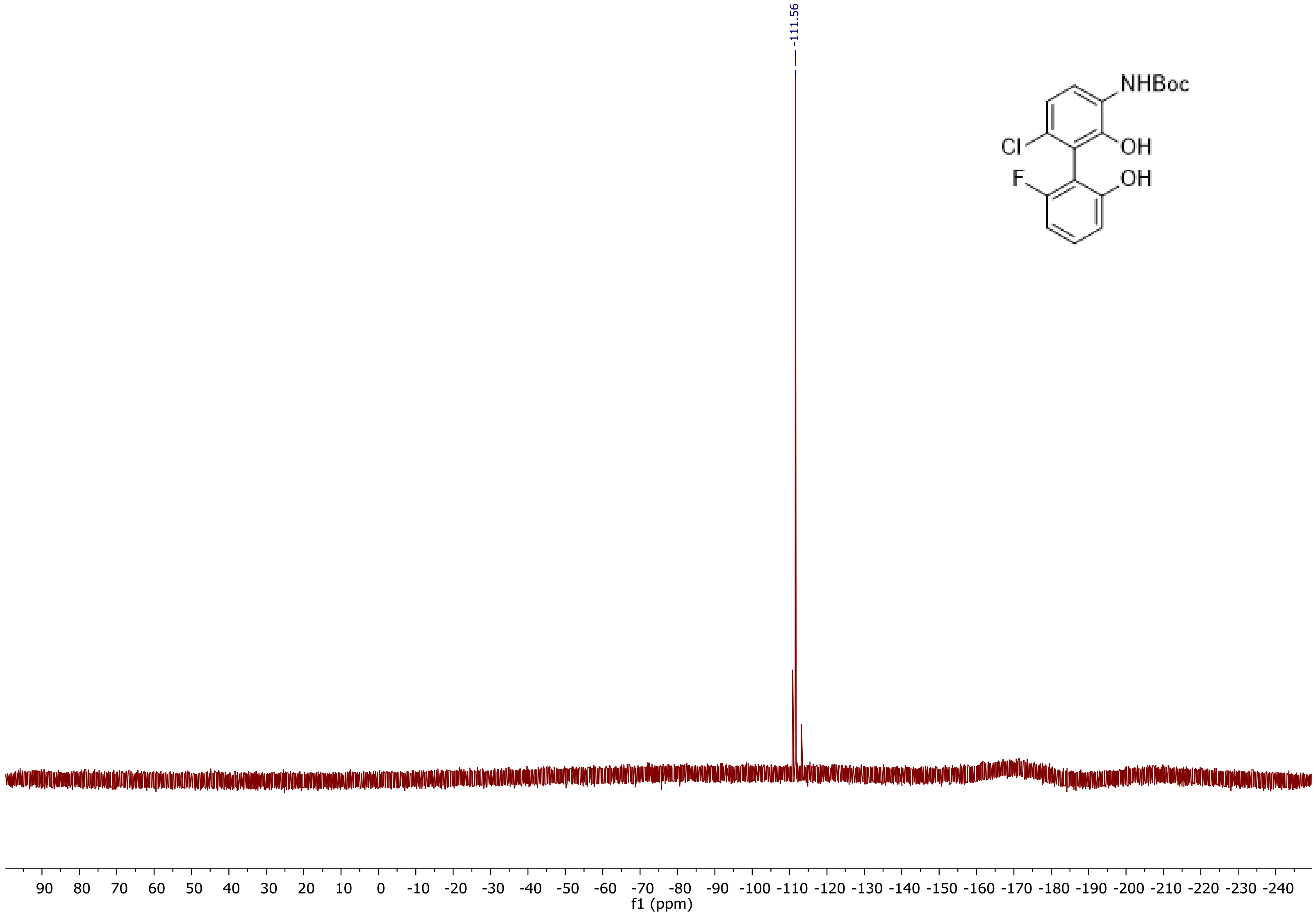

**¹H NMR** (CDCl$_3$)**:** 6-chloro-6'-fluoro-3-methoxy-[1,1'-biphenyl]-2,2'-diol (**3j**)

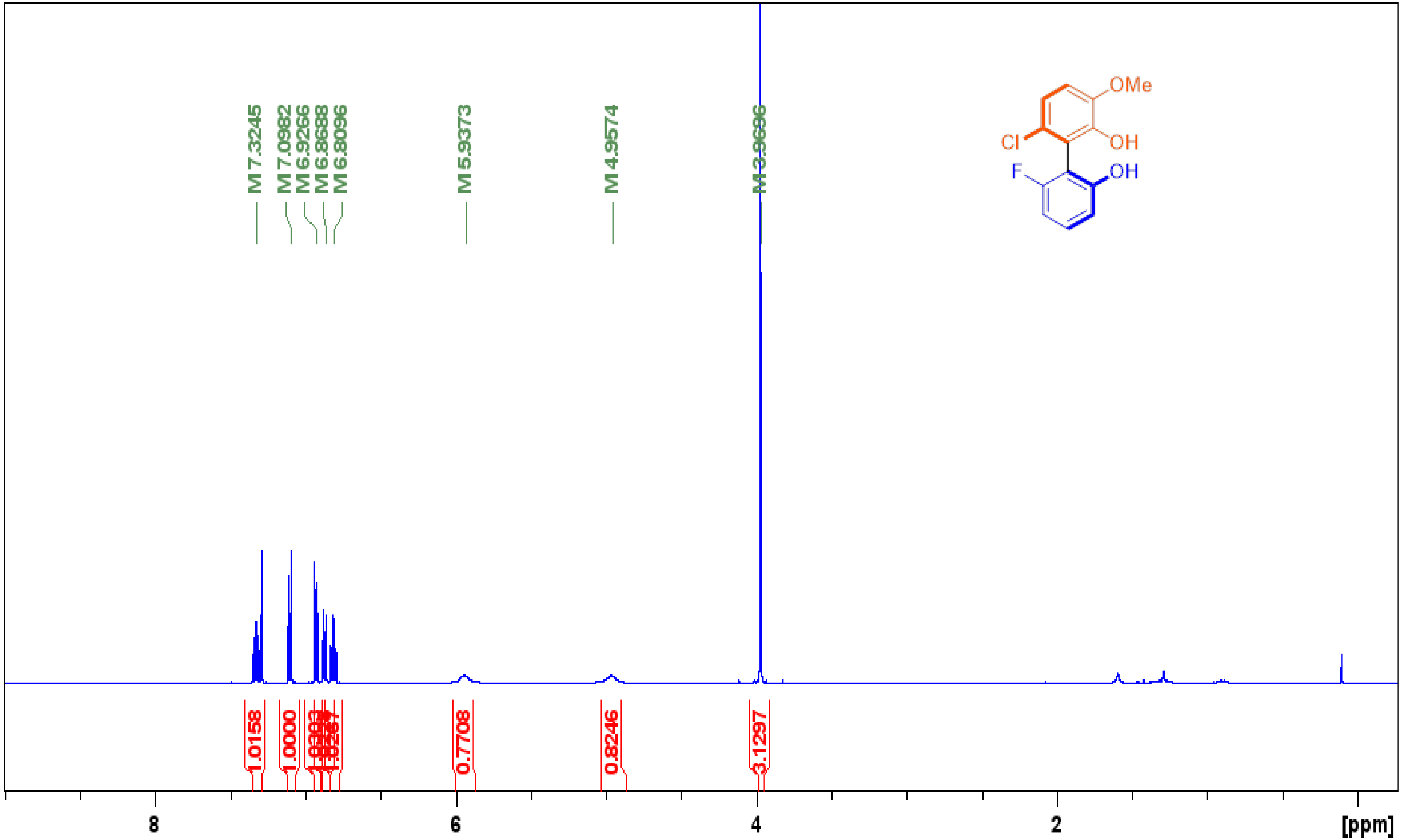

**$^{13}$C NMR** (CDCl$_3$)**:** 6-chloro-6'-fluoro-3-methoxy-[1,1'-biphenyl]-2,2'-diol (**3j**)

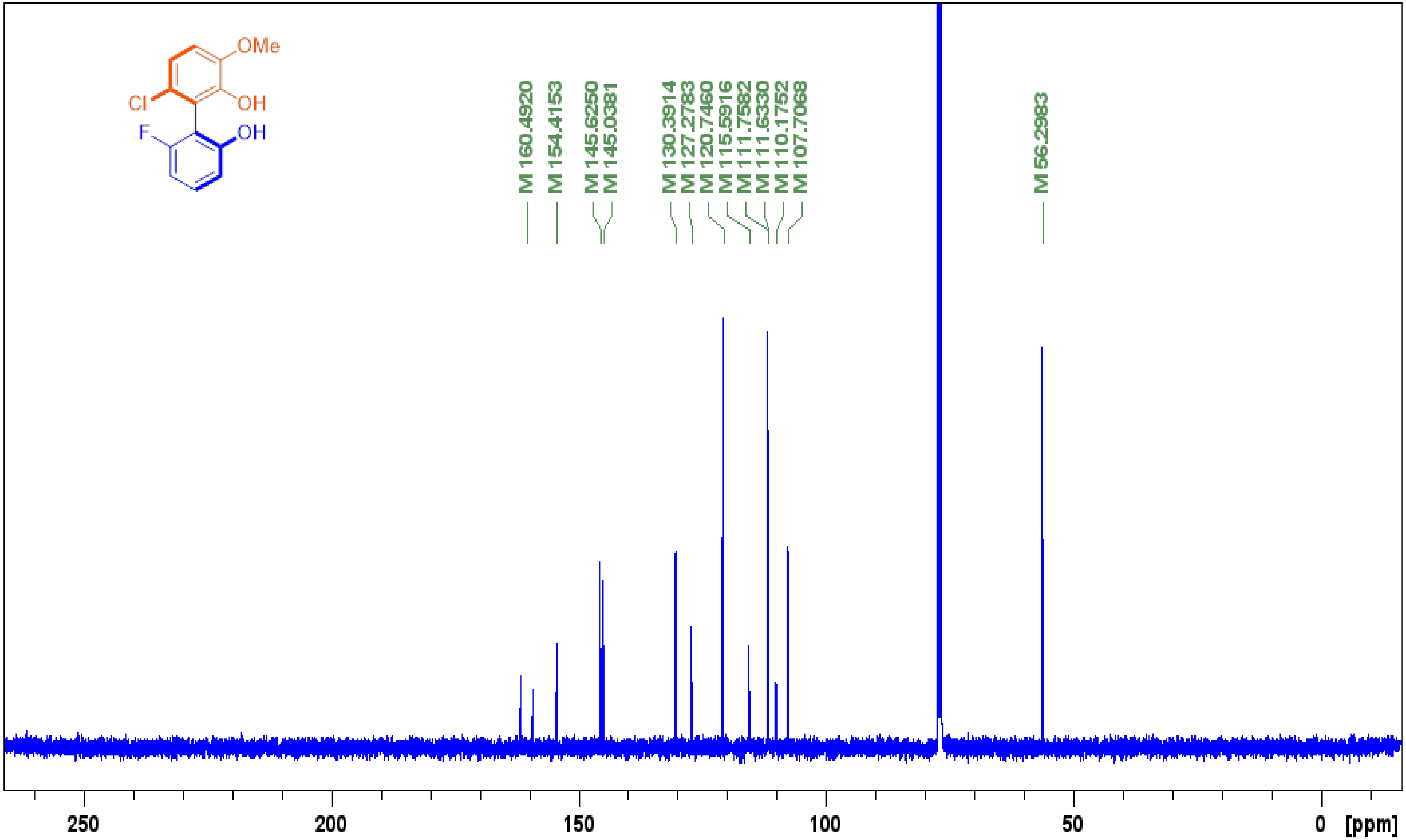

$^{19}$**F NMR** (CDCl$_3$) **):** 6-chloro-6'-fluoro-3-methoxy-[1,1'-biphenyl]-2,2'-diol (**3j**)

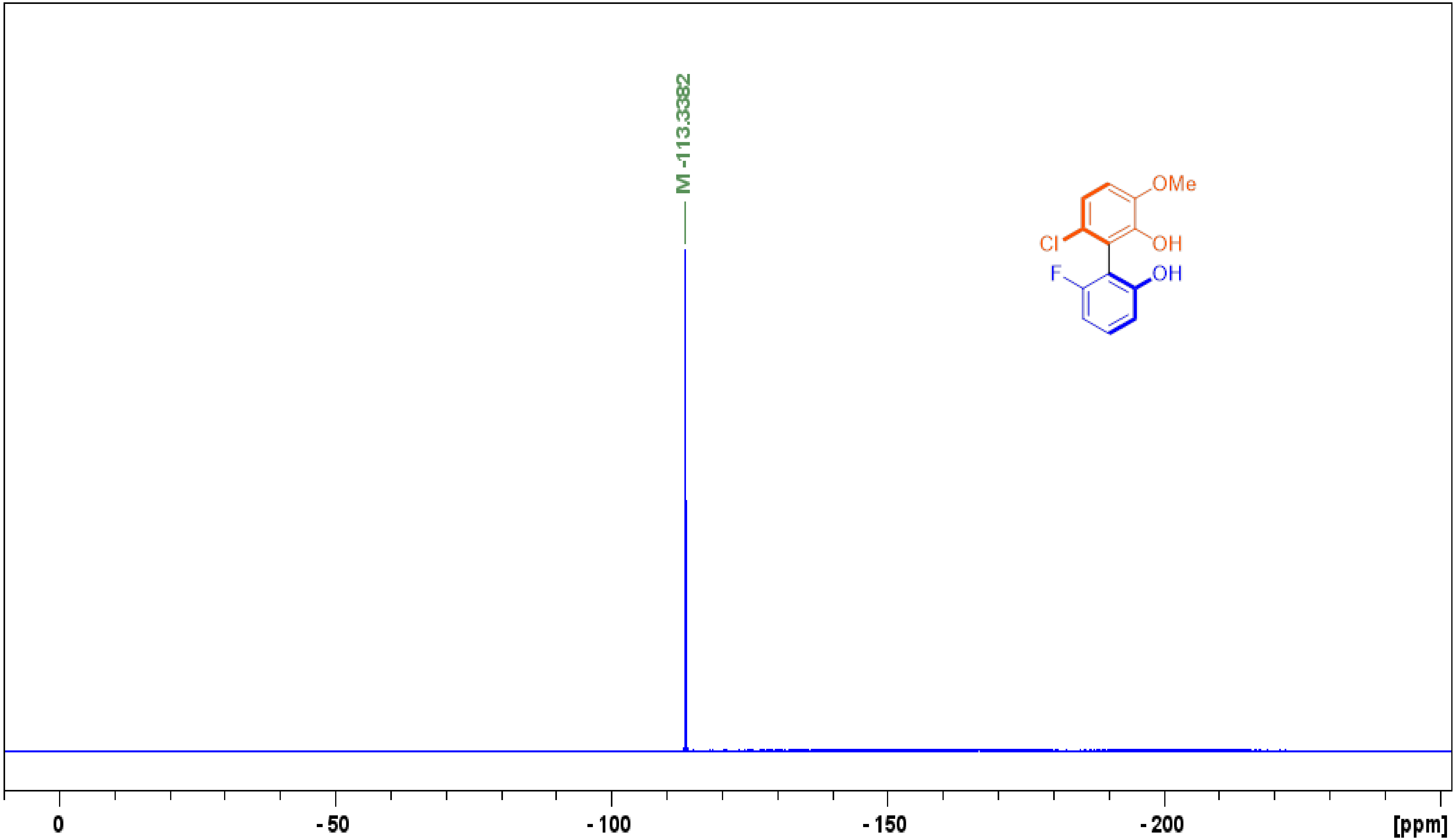

**$^{1}$H NMR** (CDCl$_3$): 6-fluoro-6'-(2-nitroethyl)-[1,1'-biphenyl]-2,2'-diol (**3k**)

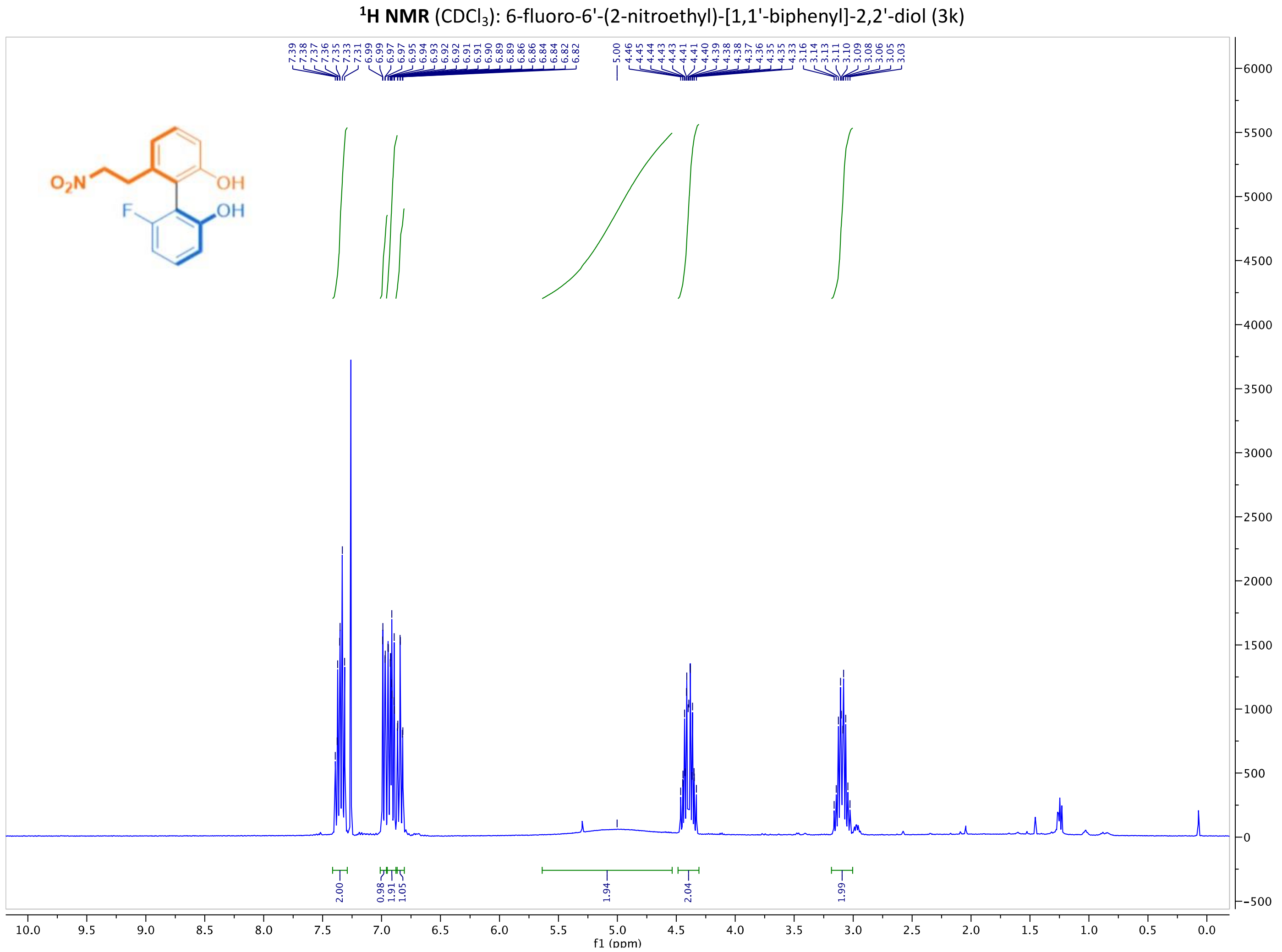

**$^{13}$C NMR** ($CDCl_3$): 6-fluoro-6'-(2-nitroethyl)-[1,1'-biphenyl]-2,2'-diol (**3k**)

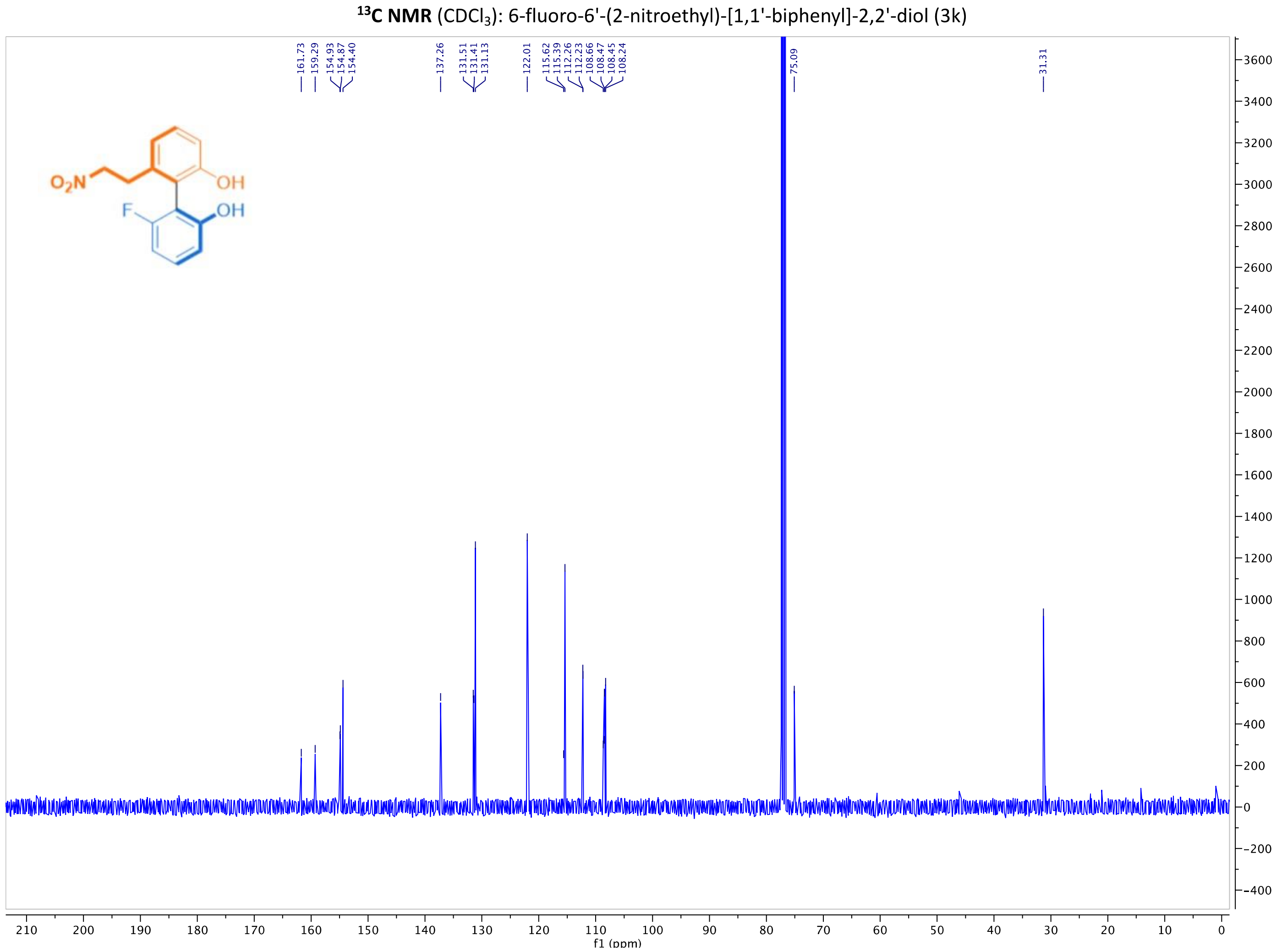

$^{19}$**F NMR** ($CDCl_3$) 6-fluoro-6'-(2-nitroethyl)-[1,1'-biphenyl]-2,2'-diol (**3k**)

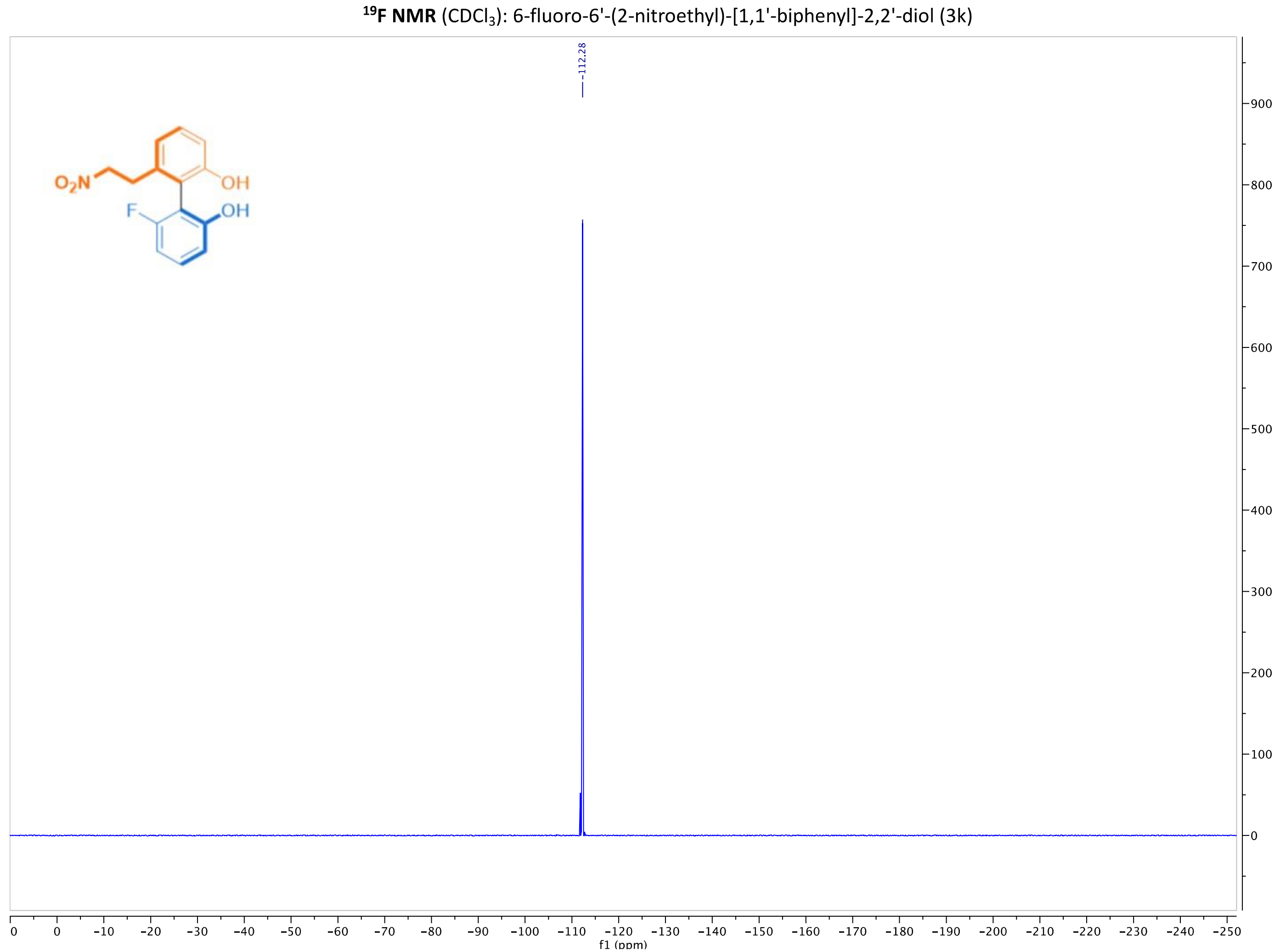

**$^1$H NMR** (Acetone-$d_6$): 6-(((*tert*-butyldimethylsilyl)oxy)methyl)-6'-fluoro-3-methoxy-[1,1'-biphenyl]-2,2'-diol (**3l**)

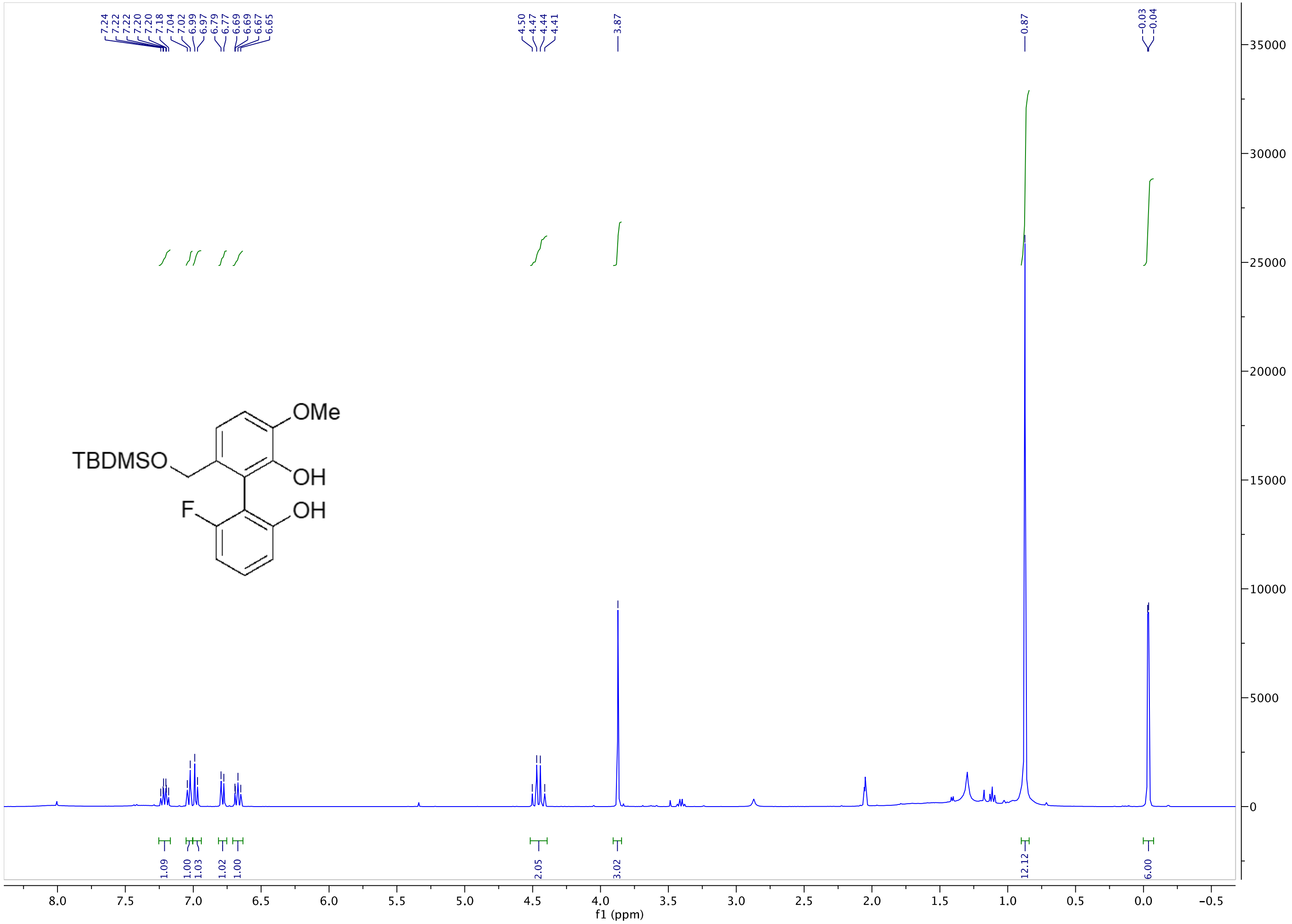

**$^{13}$C NMR** (Acetone-$d_6$): 6-(((*tert*-butyldimethylsilyl)oxy)methyl)-6'-fluoro-3-methoxy-[1,1'-biphenyl]-2,2'-diol (**3l**)

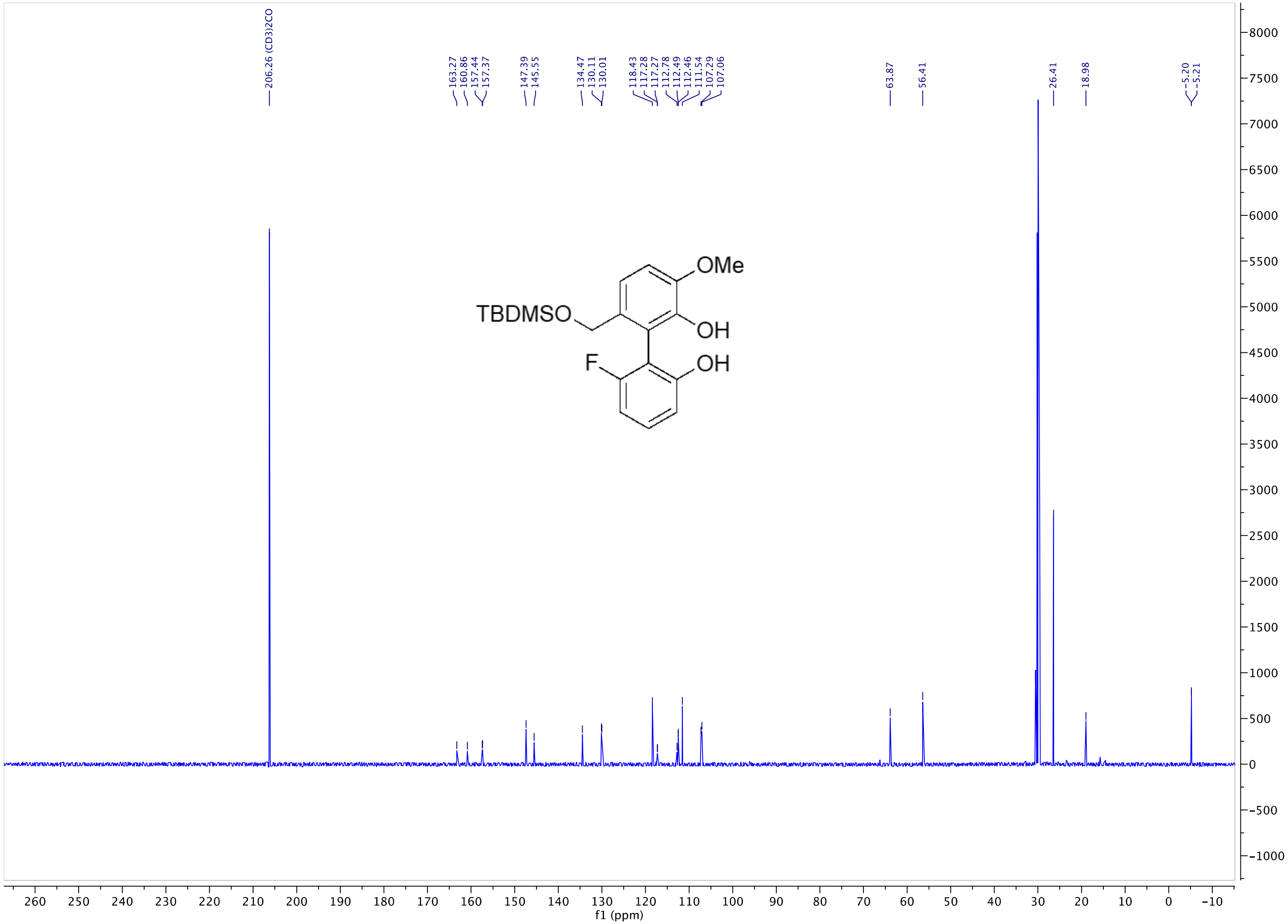

$^{19}$**F NMR** (Acetone-$d_6$) 6-(((*tert*-butyldimethylsilyl)oxy)methyl)-6'-fluoro-3-methoxy-[1,1'-biphenyl]-2,2'-diol (**3l**)

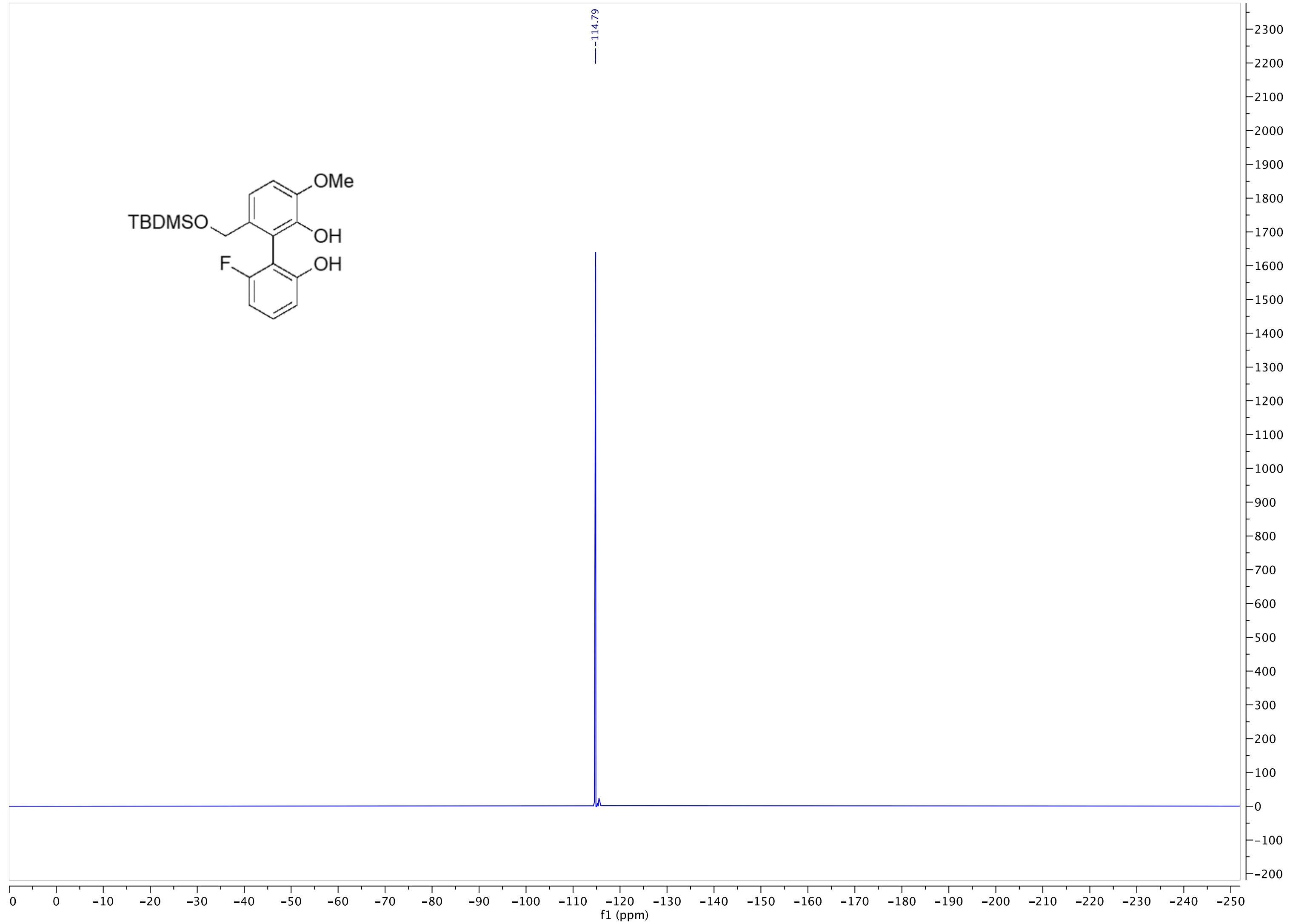

$^{1}$**H NMR** ($CDCl_3$): tert-butyl ((2'-fluoro-6,6'-dihydroxy-5-methoxy-[1,1'-biphenyl]-2-yl)methyl)carbamate (**3m**)

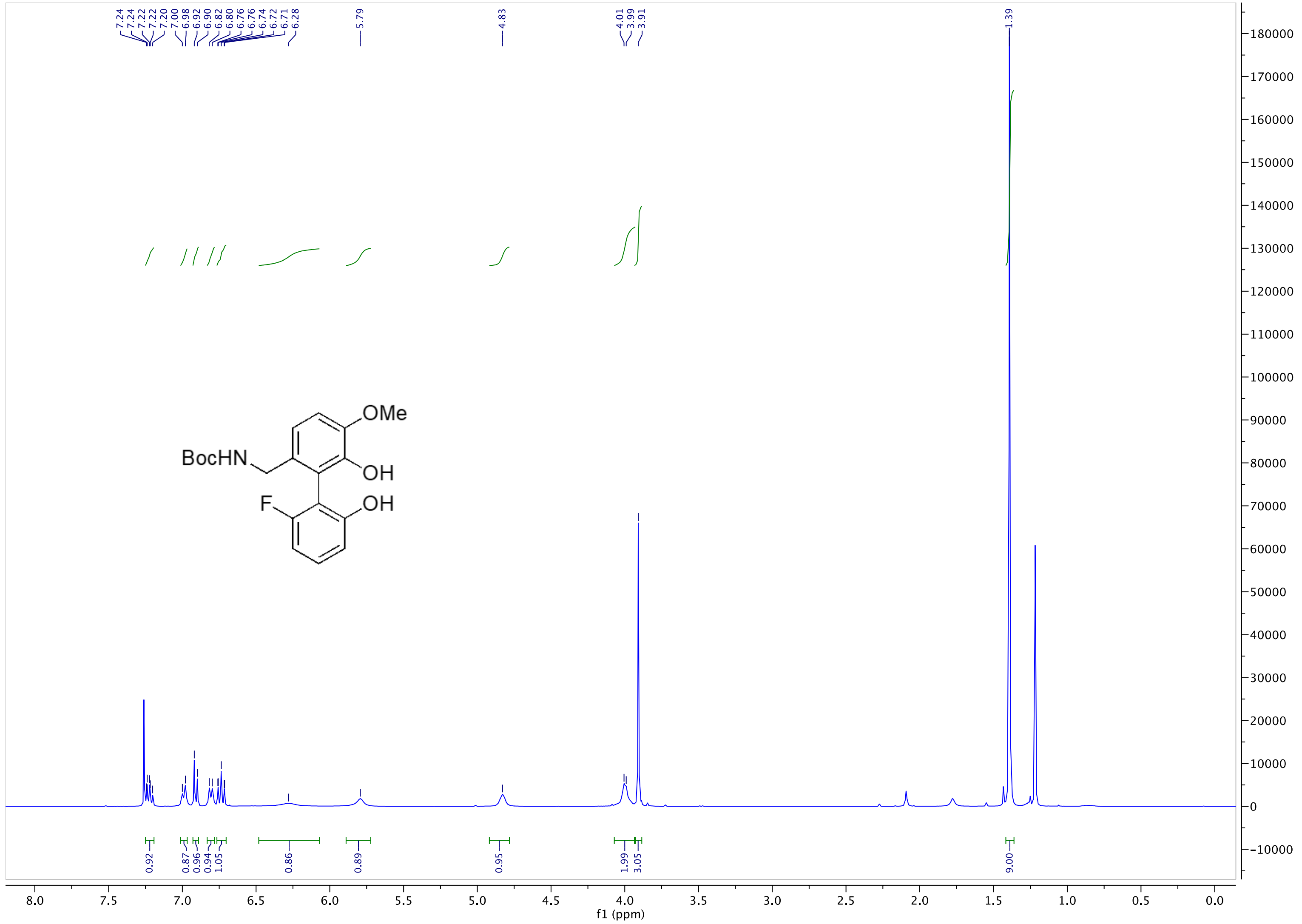

$^{13}$**C NMR** ($CDCl_3$): tert-butyl ((2'-fluoro-6,6'-dihydroxy-5-methoxy-[1,1'-biphenyl]-2-yl)methyl)carbamate (**3m**)

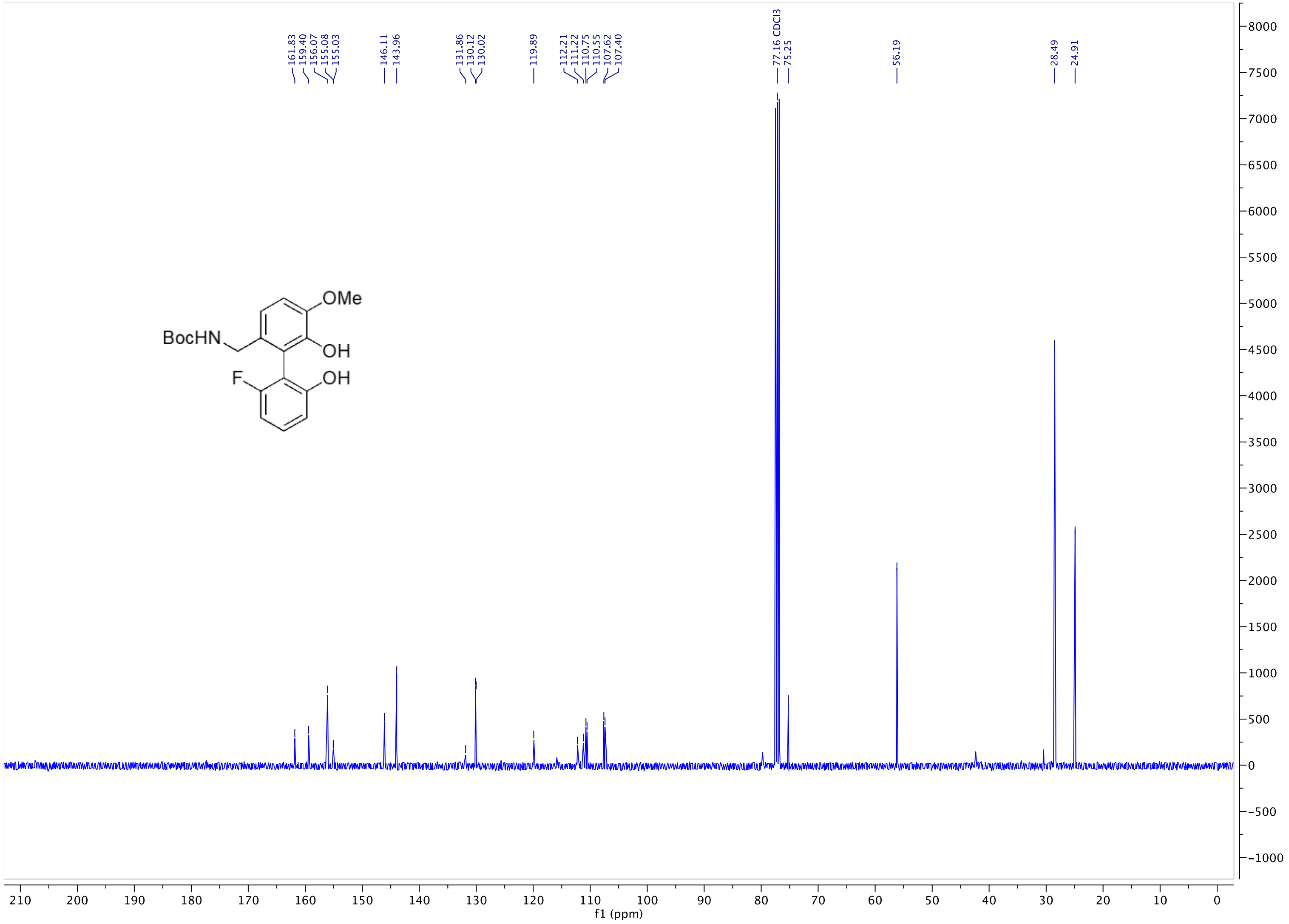

**$^{19}$F NMR** (CDCl$_3$): tert-butyl ((2'-fluoro-6,6'-dihydroxy-5-methoxy-[1,1'-biphenyl]-2-yl)methyl)carbamate (**3m**)

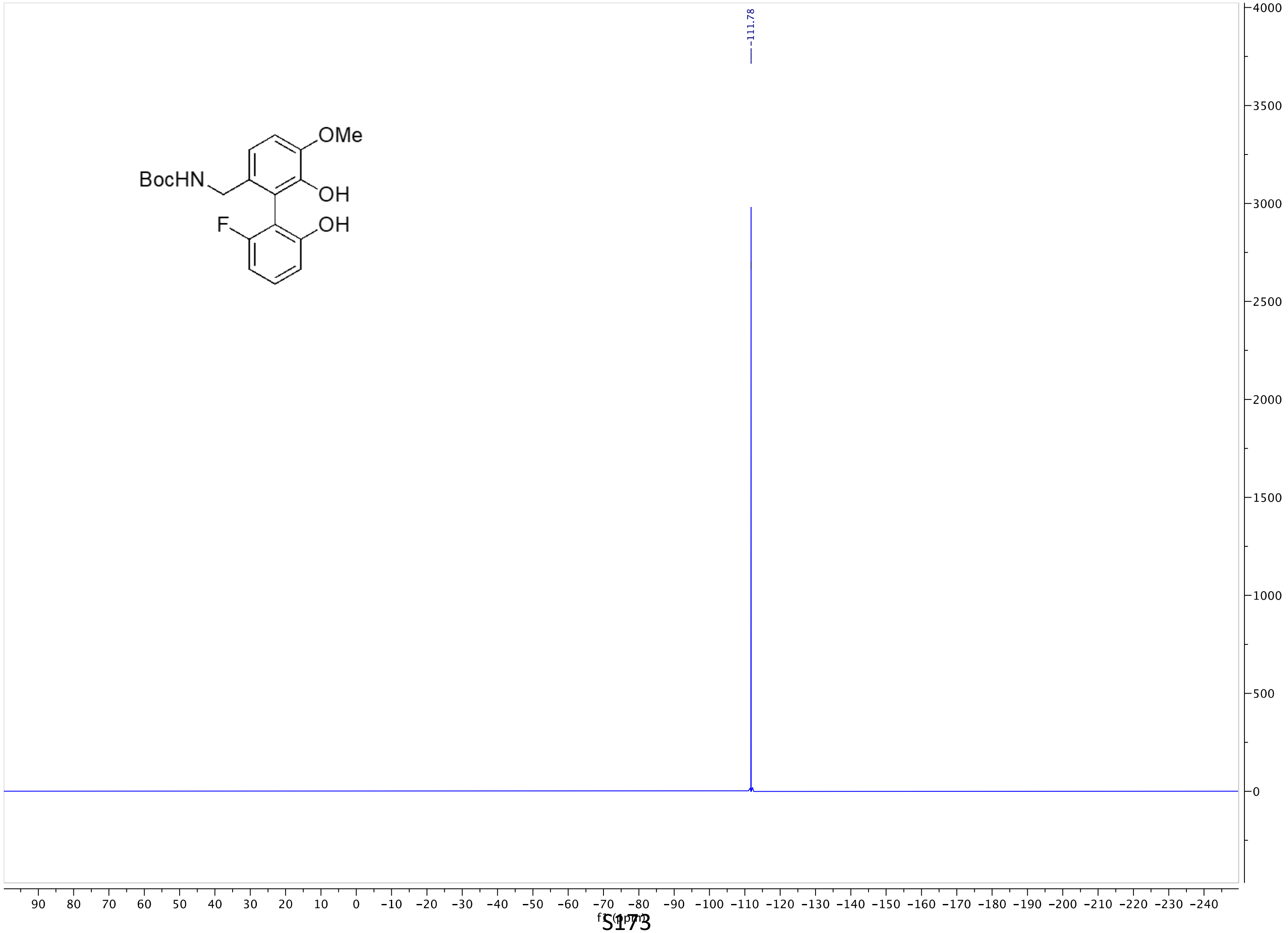

**¹H NMR** ($CDCl_3$)**:** 6-chloro-3-(2,4-dichlorophenoxy)-6'-fluoro-[1,1'-biphenyl]-2,2'-diol (**3n**)

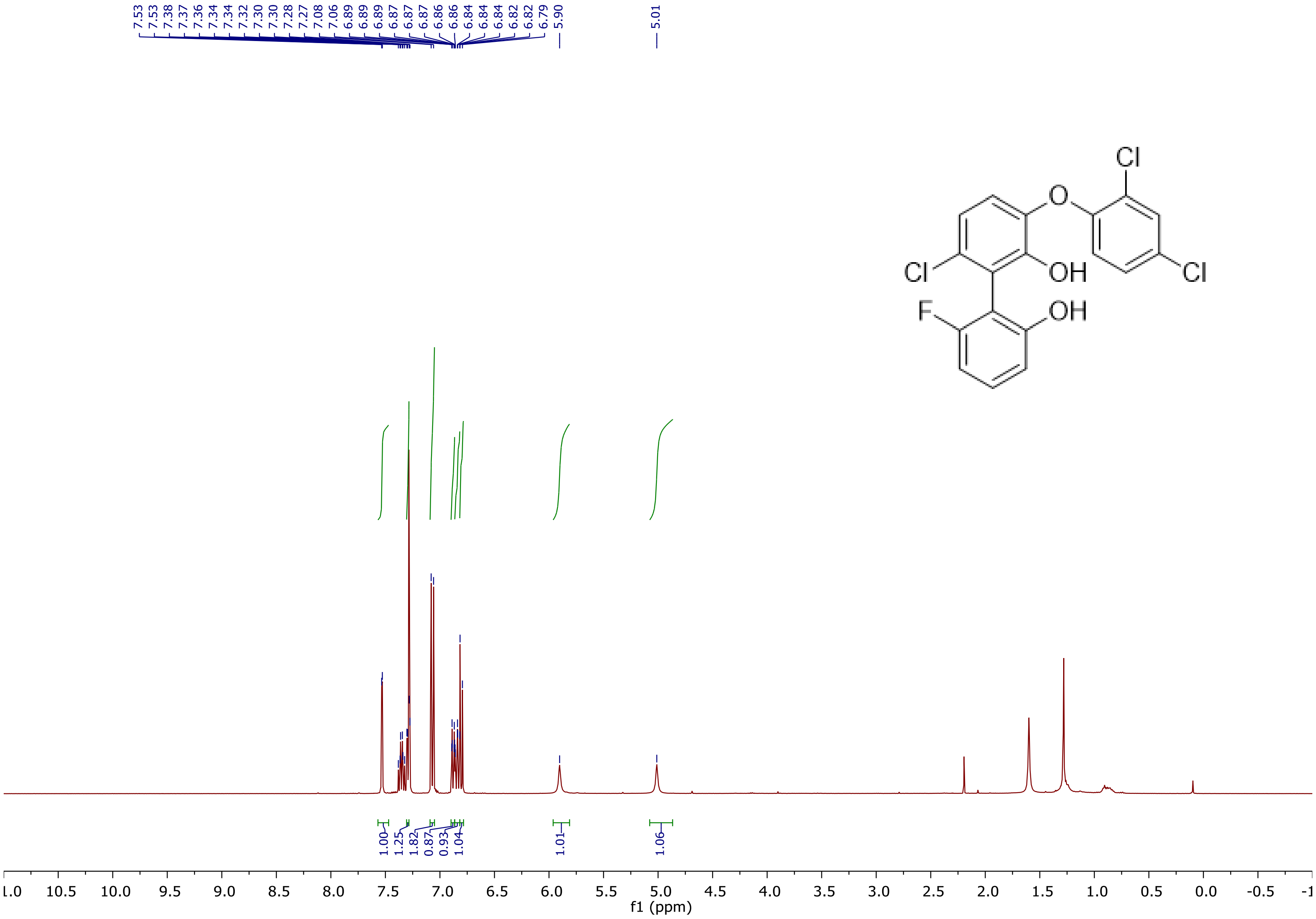

**$^{13}$C NMR** ($CDCl_3$)**:** 6-chloro-3-(2,4-dichlorophenoxy)-6'-fluoro-[1,1'-biphenyl]-2,2'-diol (**3n**)

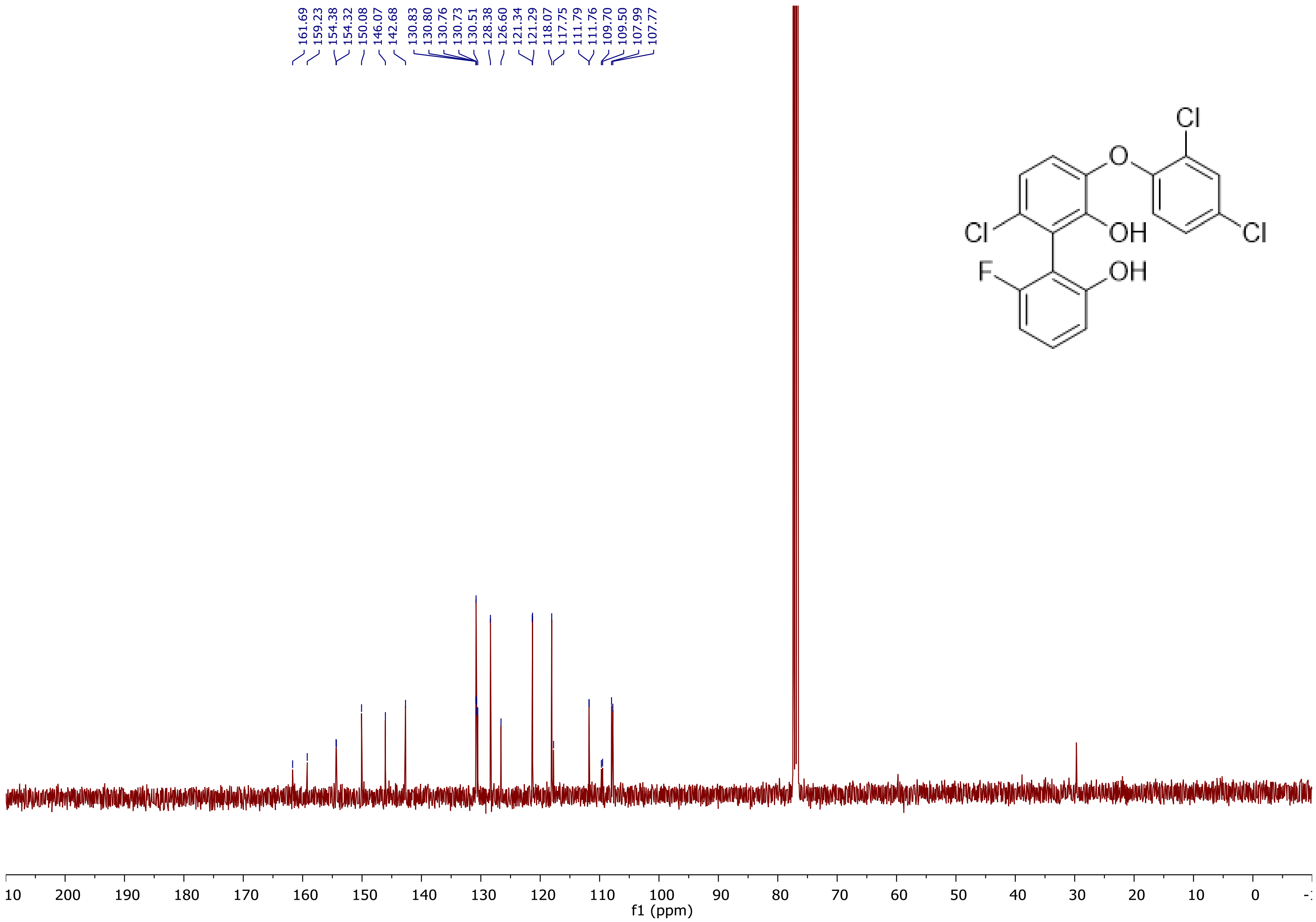

**$^{19}$F NMR** ($CDCl_3$)**:** 6-chloro-3-(2,4-dichlorophenoxy)-6'-fluoro-[1,1'-biphenyl]-2,2'-diol (**3n**)

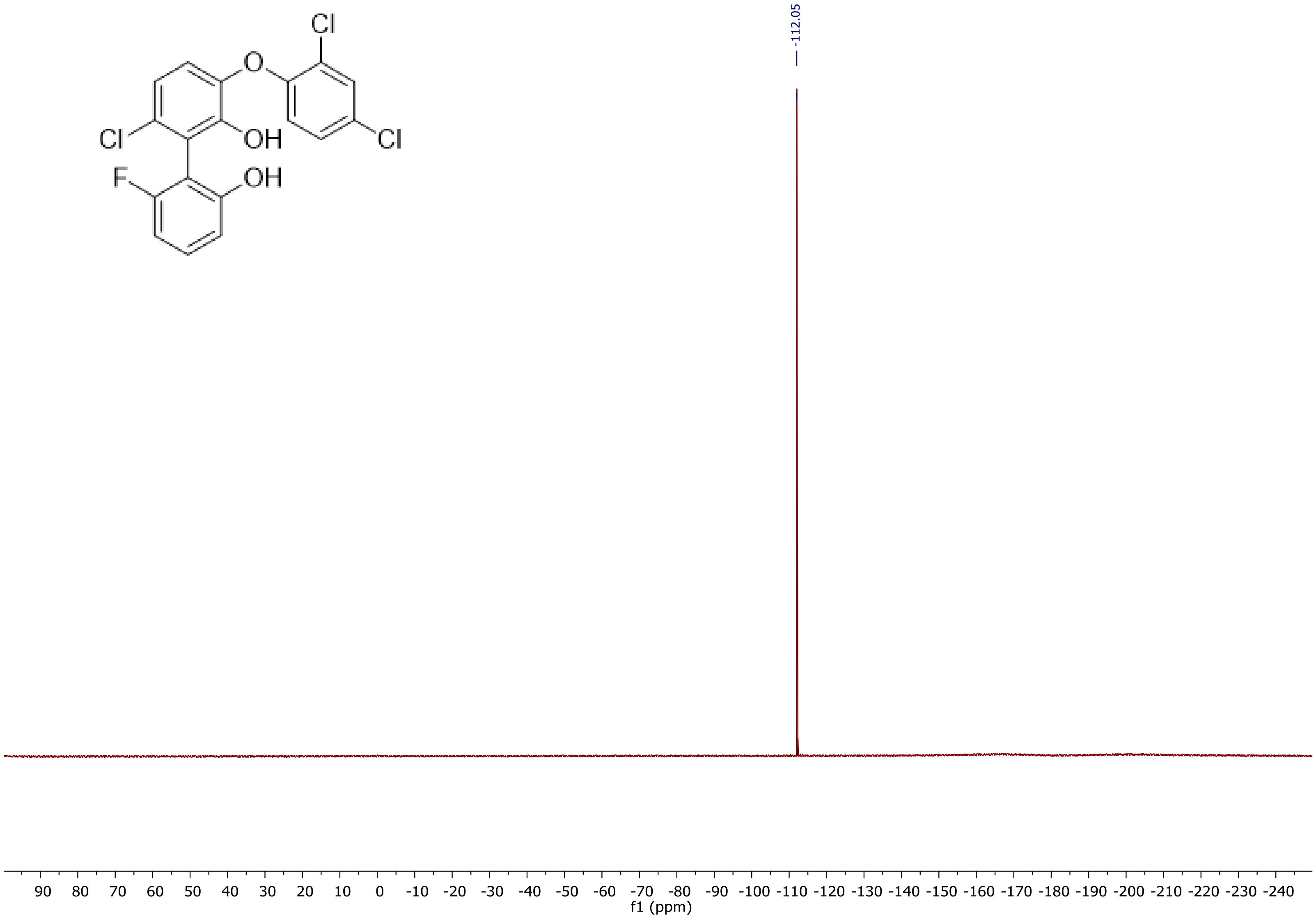

**$^1$H NMR** ($CDCl_3$)**:** (4R,8R,9S,13S,14S)-4-(2-fluoro-6-hydroxyphenyl)-3-hydroxy-13-methyl-6,7,8,9,11,12,13,14,15,16-decahydro-17H-cyclopenta[a]phenanthren-17-one (**3o**)

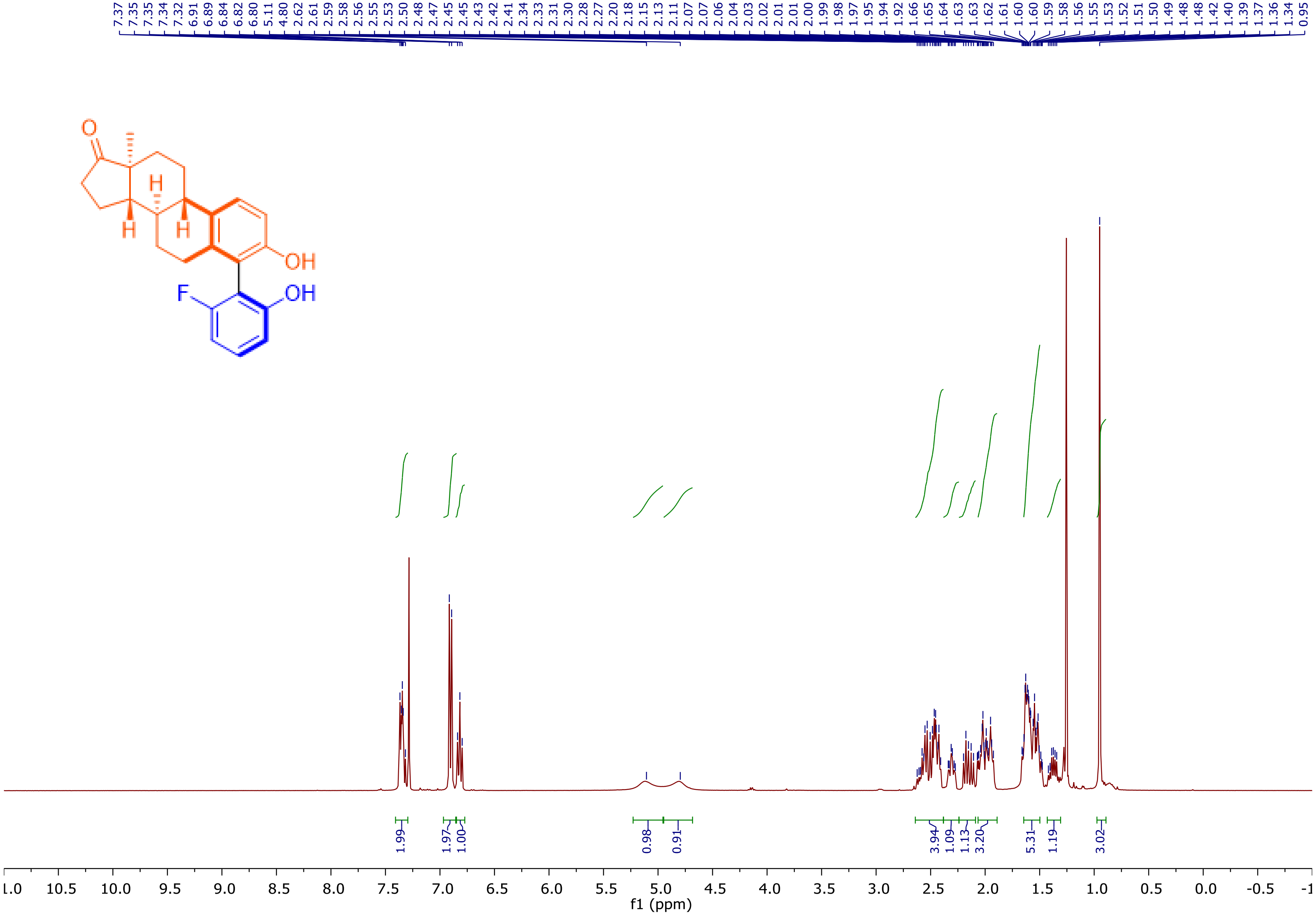

**13C NMR** ($CDCl_3$)**:** (4R,8R,9S,13S,14S)-4-(2-fluoro-6-hydroxyphenyl)-3-hydroxy-13-methyl-6,7,8,9,11,12,13,14,15,16-decahydro-17H-cyclopenta[a]phenanthren-17-one (**3o**)

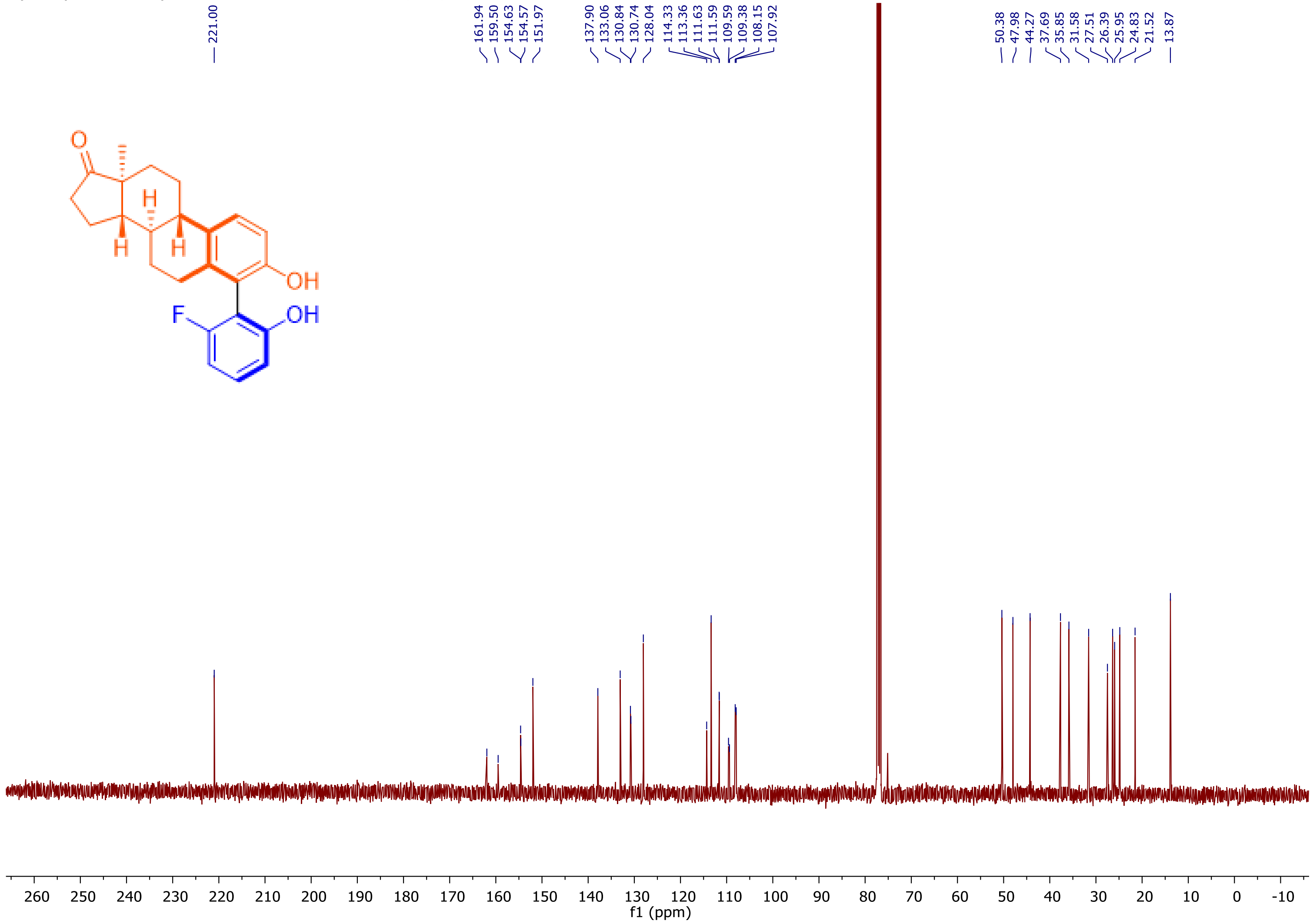

**$^{19}$F NMR** (CDCl$_3$)**:** (4R,8R,9S,13S,14S)-4-(2-fluoro-6-hydroxyphenyl)-3-hydroxy-13-methyl-6,7,8,9,11,12,13,14,15,16-decahydro-17H-cyclopenta[a]phenanthren-17-one (**3o**)

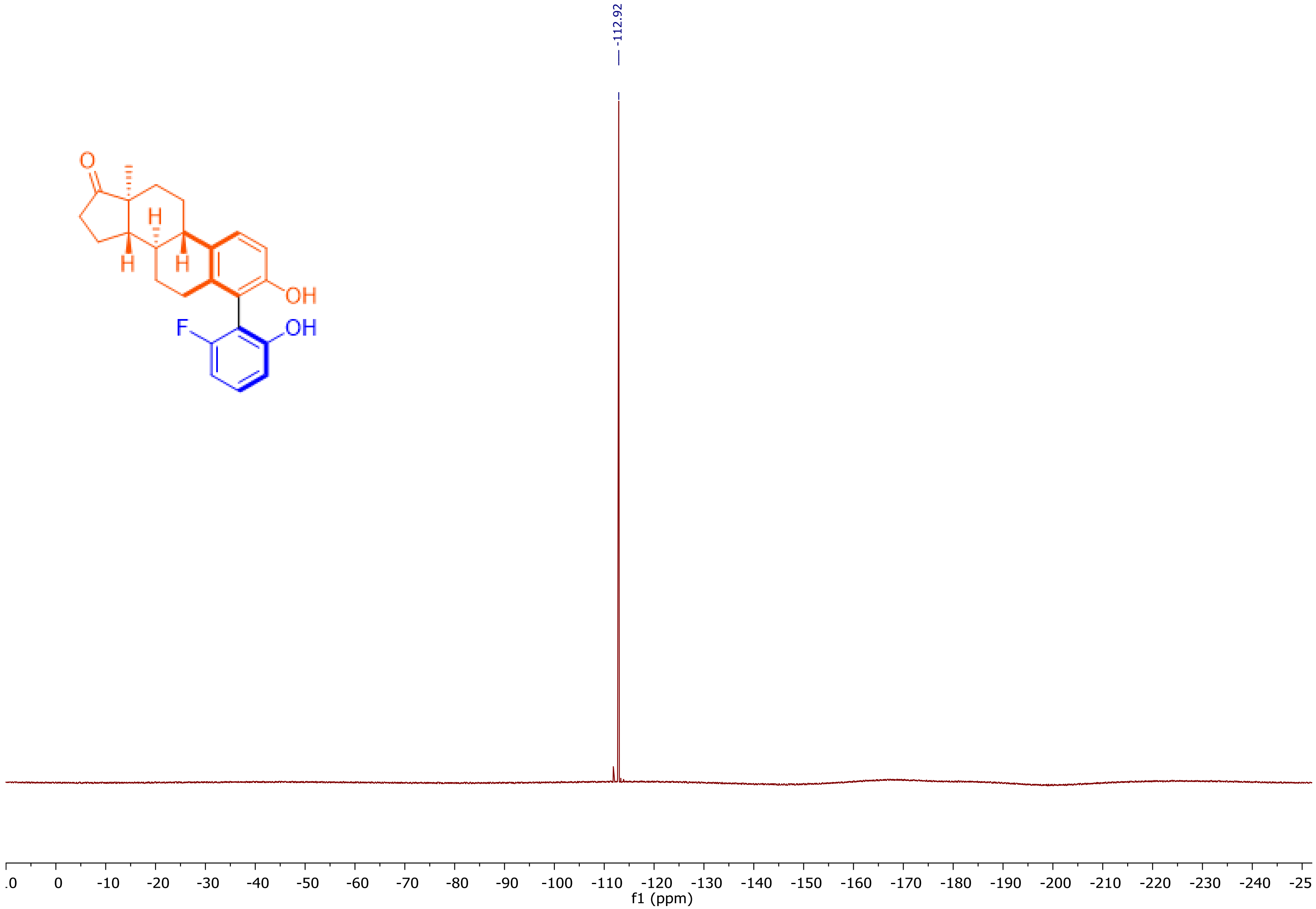

**$^{1}$H NMR** (CDCl$_3$)**:** 6-chloro-6'-methyl-[1,1'-biphenyl]-2,2'-diol (**3p**)

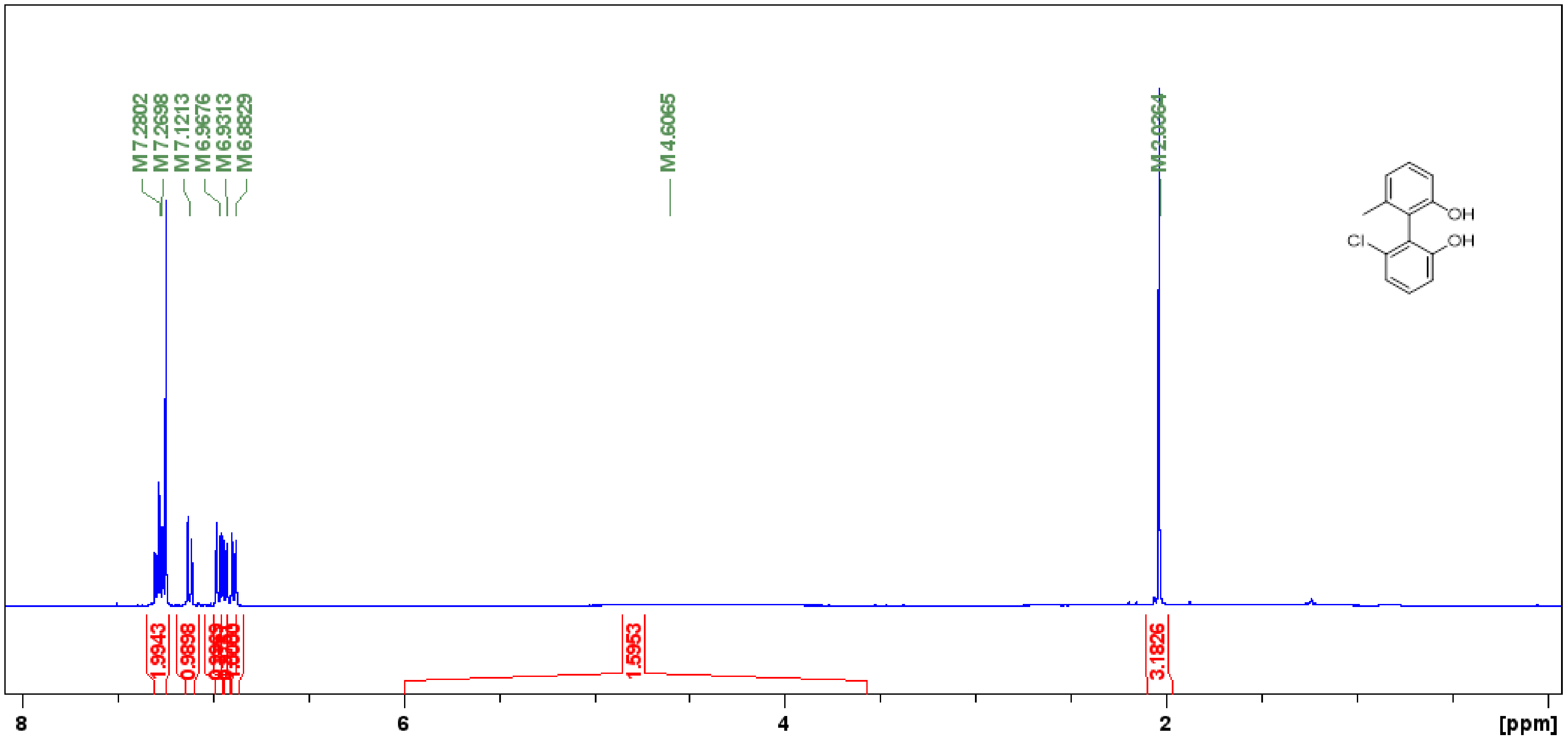

**$^{13}$C NMR** ($CDCl_3$)**:** 6-chloro-6'-methyl-[1,1'-biphenyl]-2,2'-diol (**3p**)

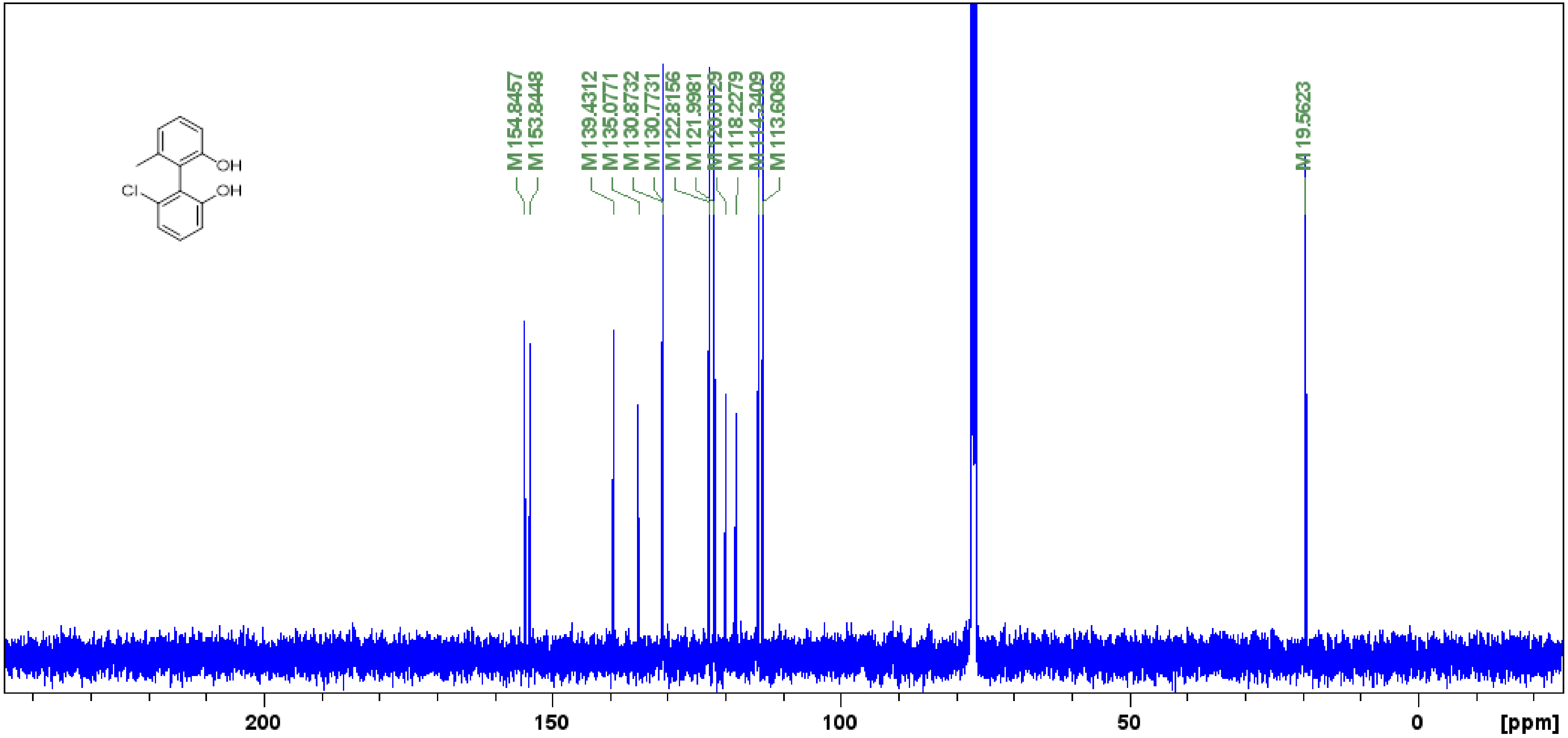

**$^1$H NMR** (CDCl$_3$)**:** 6-chloro-6'-methoxy-[1,1'-biphenyl]-2,2'-diol **(3q)**

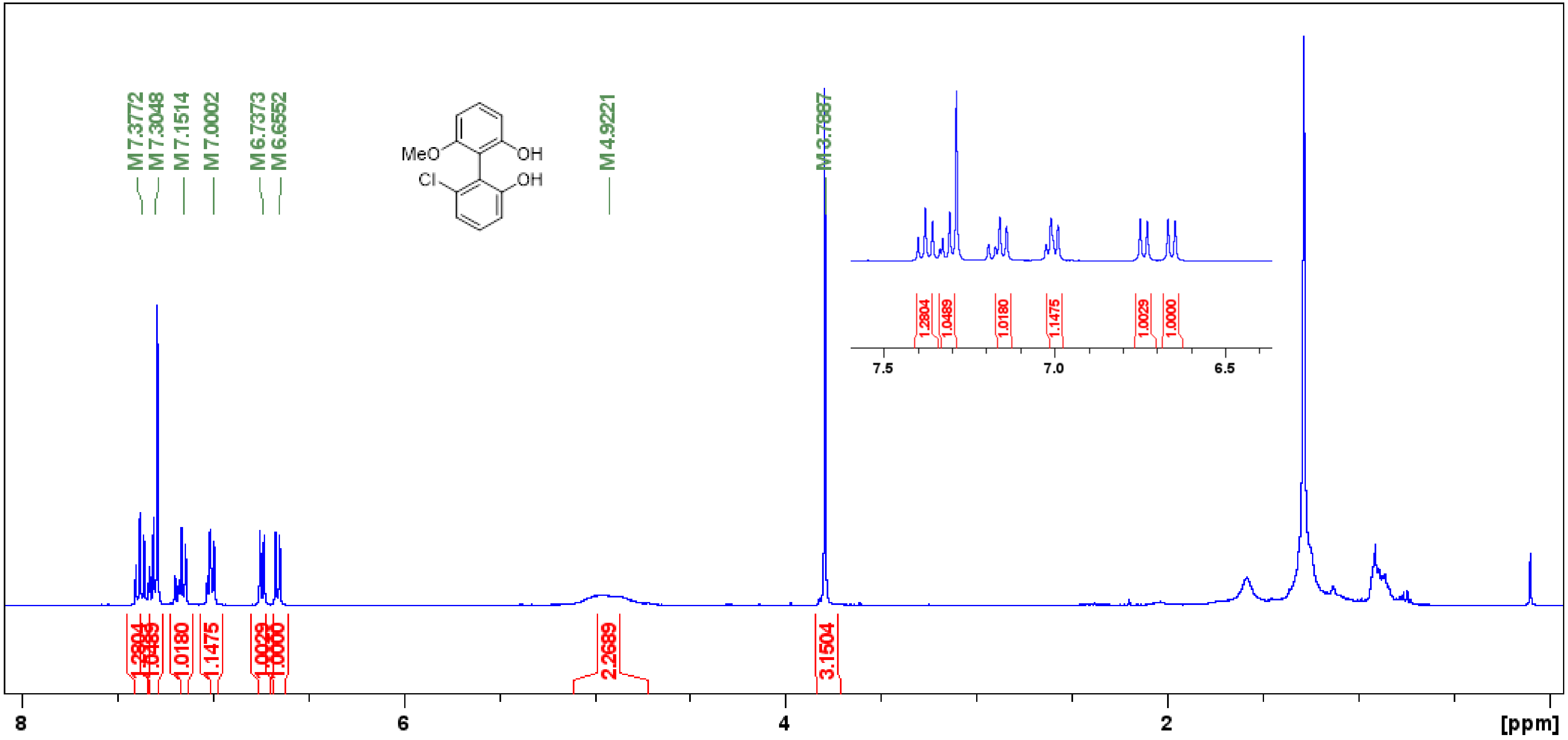

**$^{13}$C NMR** (CDCl$_3$)**:** 6-chloro-6'-methoxy-[1,1'-biphenyl]-2,2'-diol **(3q)**

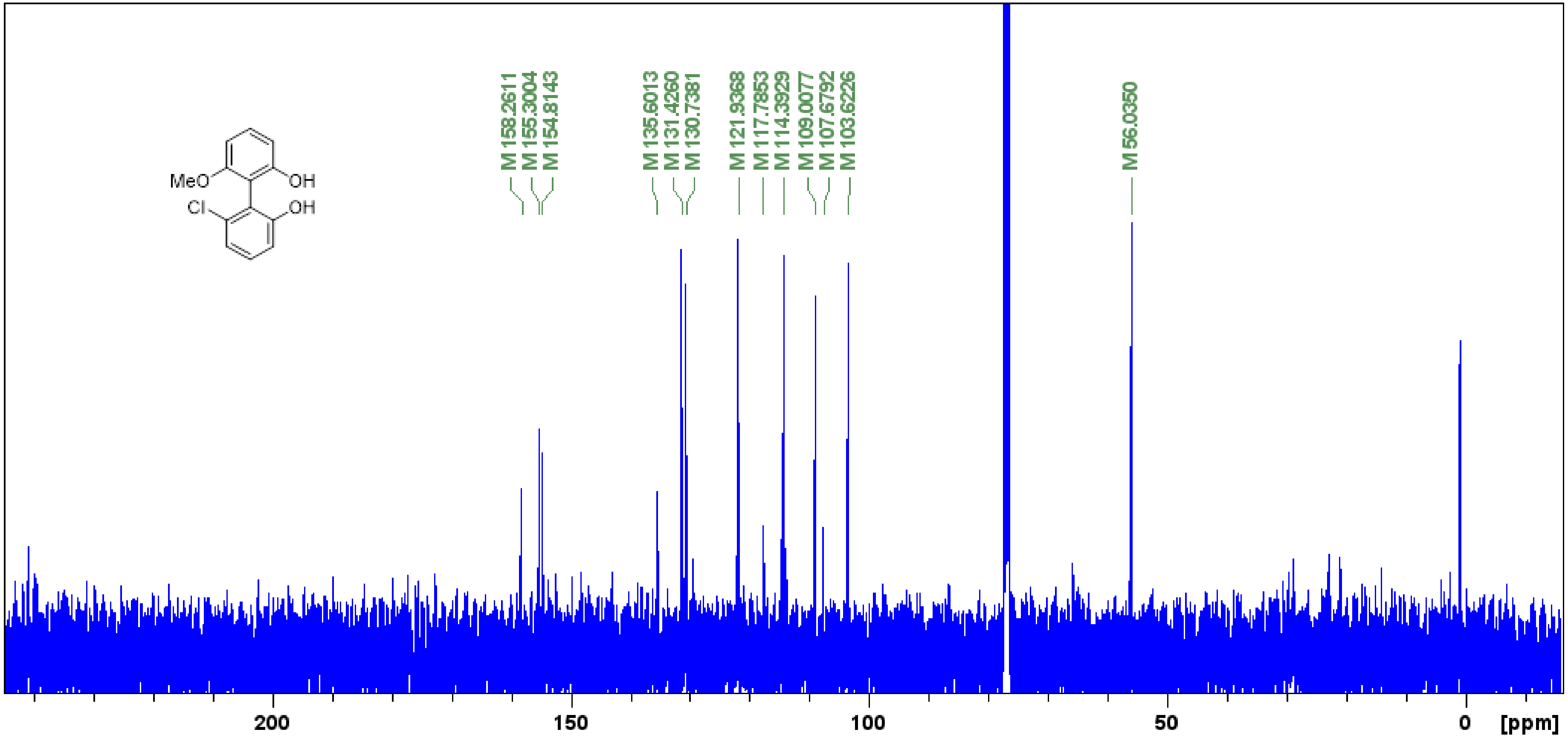

**$^1$H NMR** ($CDCl_3$)**:** 6,6'-dichloro-[1,1'-biphenyl]-2,2'-diol **(3r)**

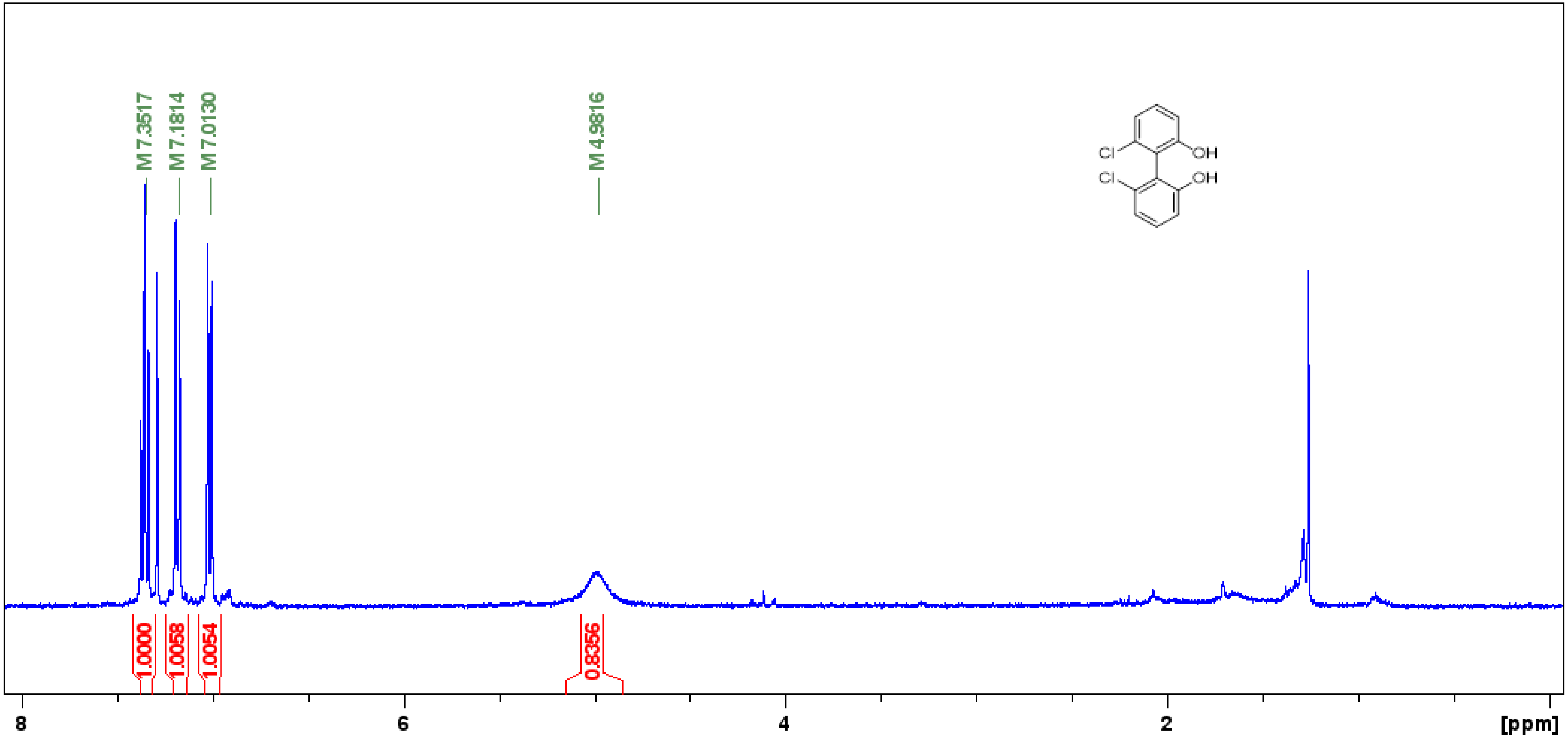

$^{13}$**C NMR** (CDCl$_3$)**:** 6,6'-dichloro-[1,1'-biphenyl]-2,2'-diol **(3r)**

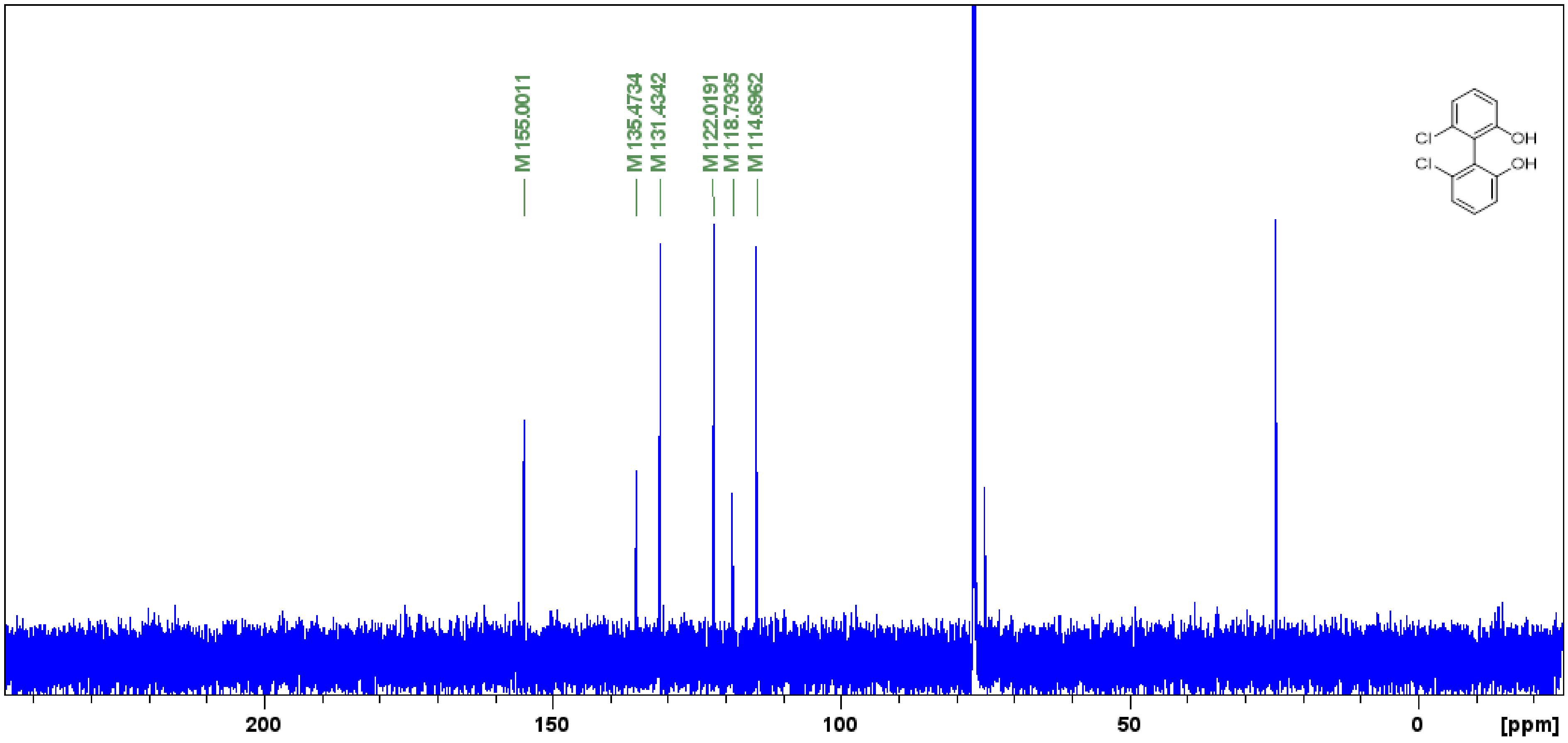

**$^{1}$H NMR** ($CDCl_3$)**:** 6,6'-dichloro-[1,1':3',1''-terphenyl]-2,2'-diol (**3s**)

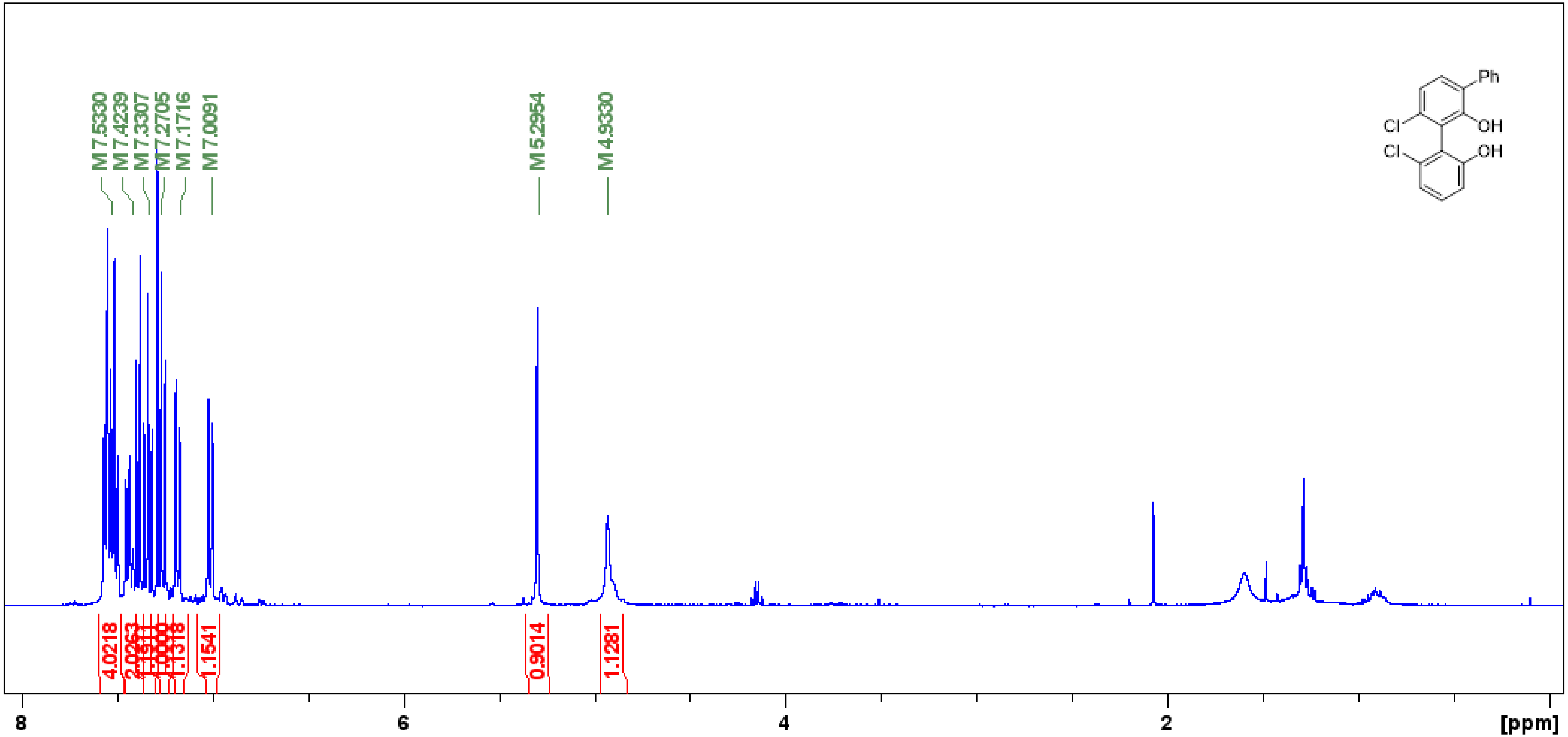

**$^{13}$C NMR** ($CDCl_3$)**:** 6,6'-dichloro-[1,1':3',1''-terphenyl]-2,2'-diol (**3s**)

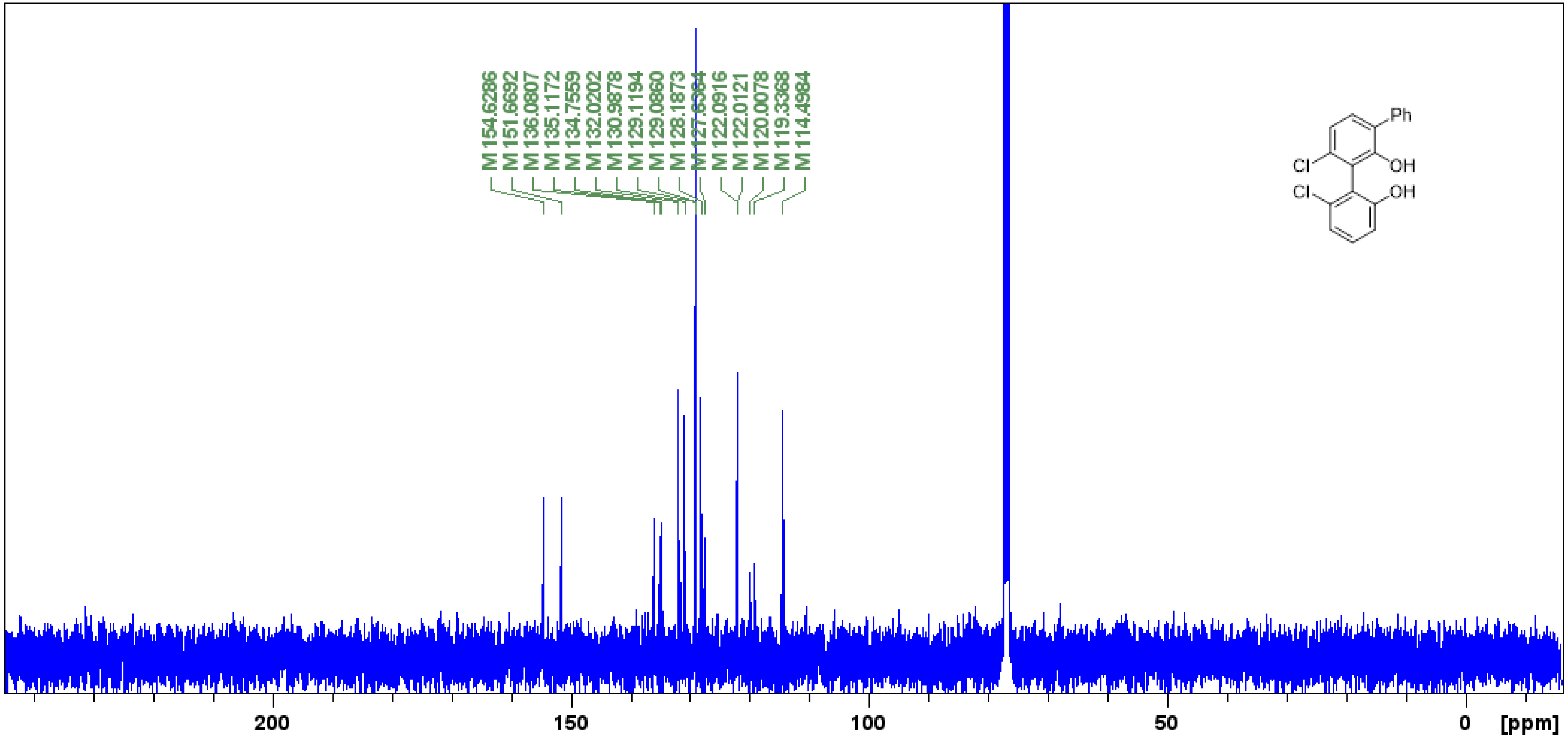

**$^{1}$H NMR** ($CDCl_3$)**:** 6,6'-dichloro-3-methyl-[1,1'-biphenyl]-2,2'-diol (**3t**)

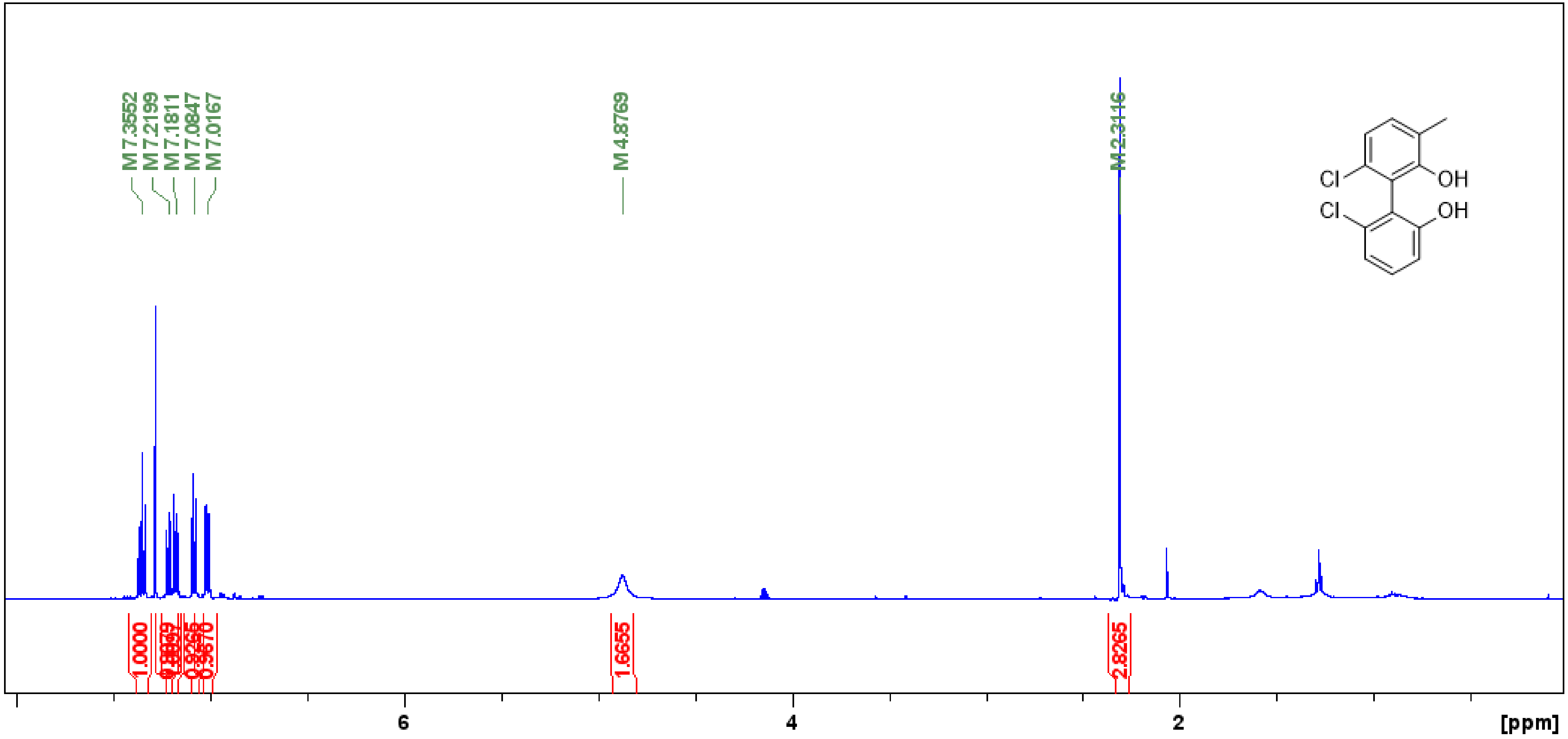

**$^{13}$C NMR** ($CDCl_3$)**:** 6,6'-dichloro-3-methyl-[1,1'-biphenyl]-2,2'-diol (**3t**)

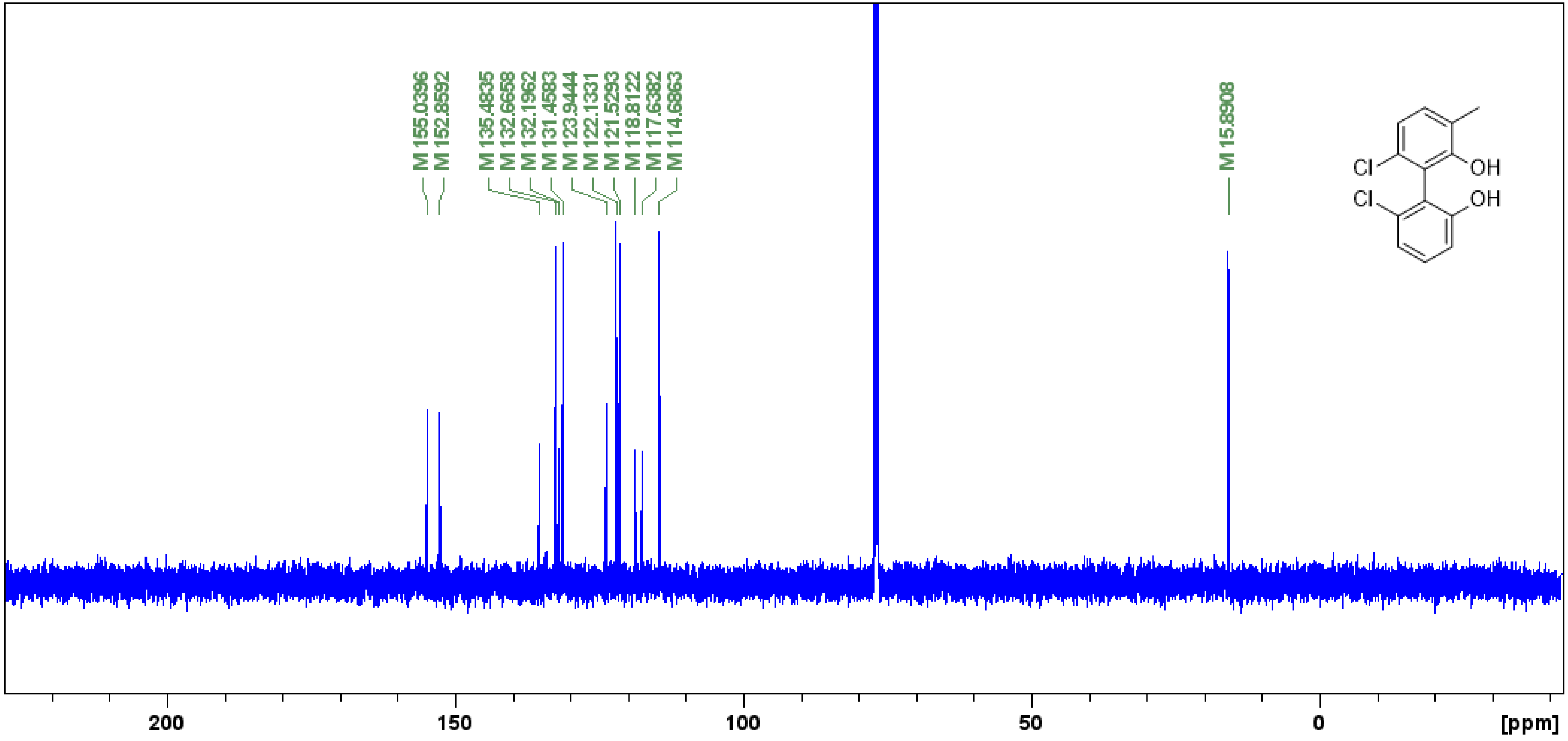

**¹H NMR** (CDCl₃)**:** 6-chloro-6'-(trifluoromethoxy)-[1,1'-biphenyl]-2,2'-diol (**3u**)

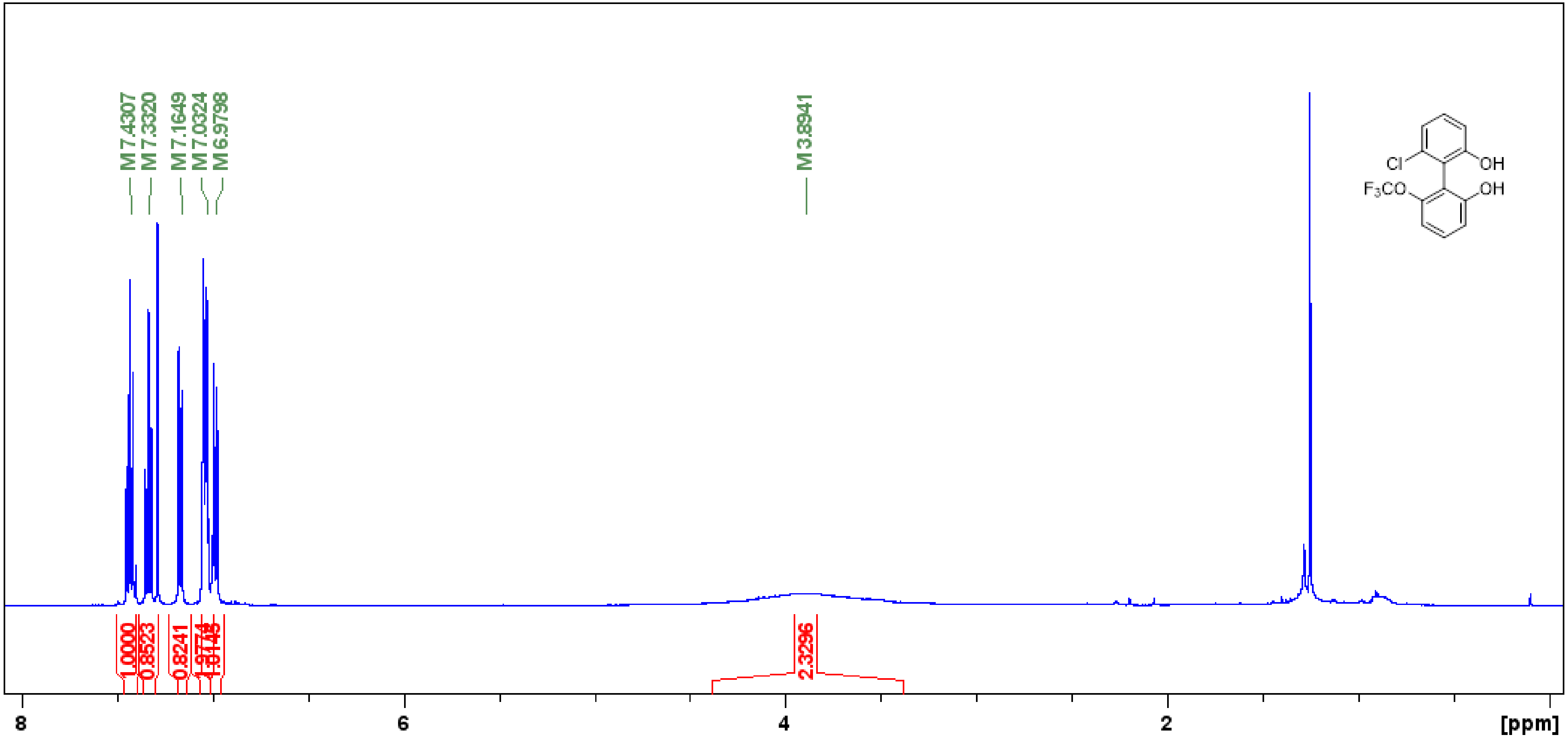

**$^{13}$C NMR** (CDCl$_3$)**:** 6-chloro-6'-(trifluoromethoxy)-[1,1'-biphenyl]-2,2'-diol (**3u**)

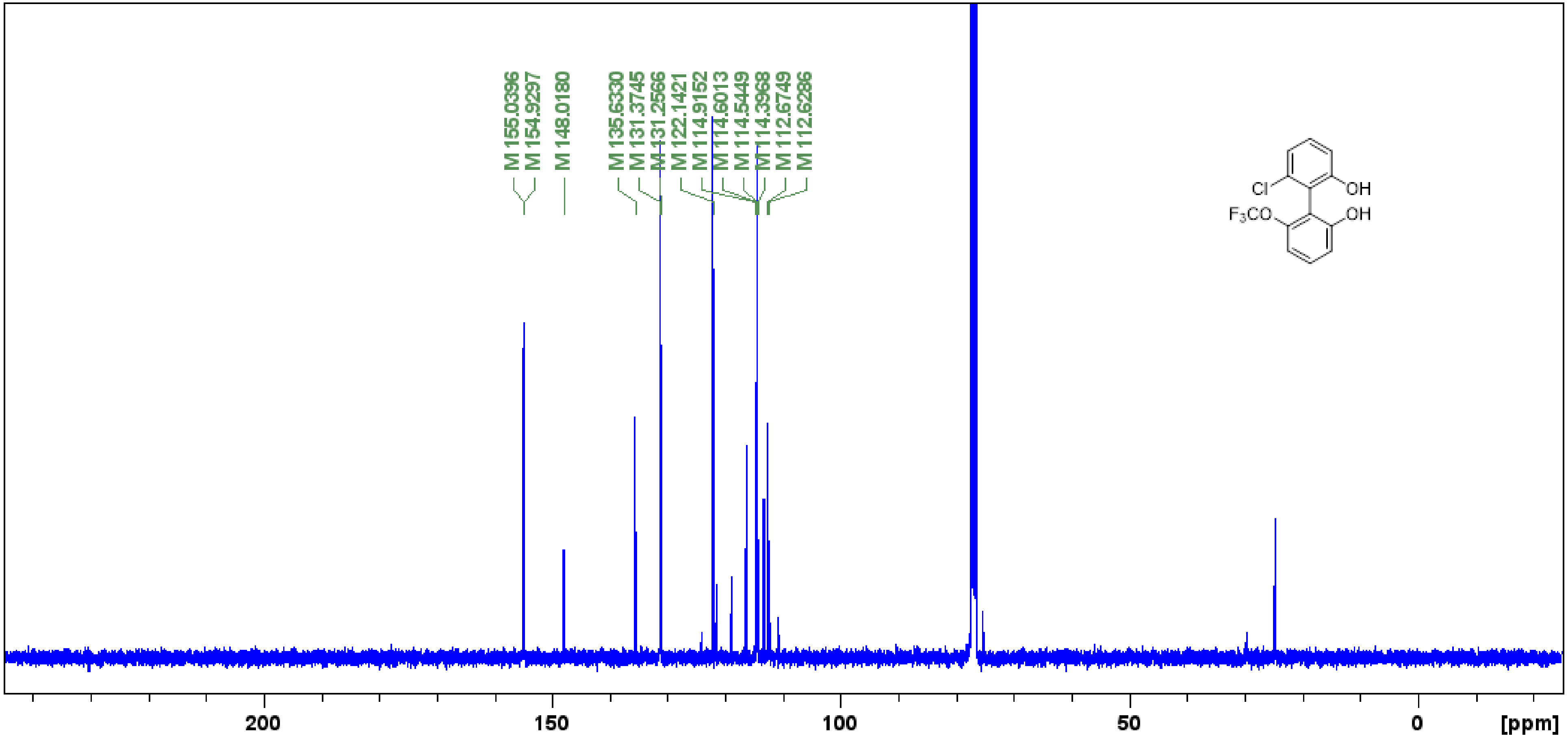

**$^{19}$F NMR** ($CDCl_3$)**:** 6-chloro-6'-(trifluoromethoxy)-[1,1'-biphenyl]-2,2'-diol (**3u**)

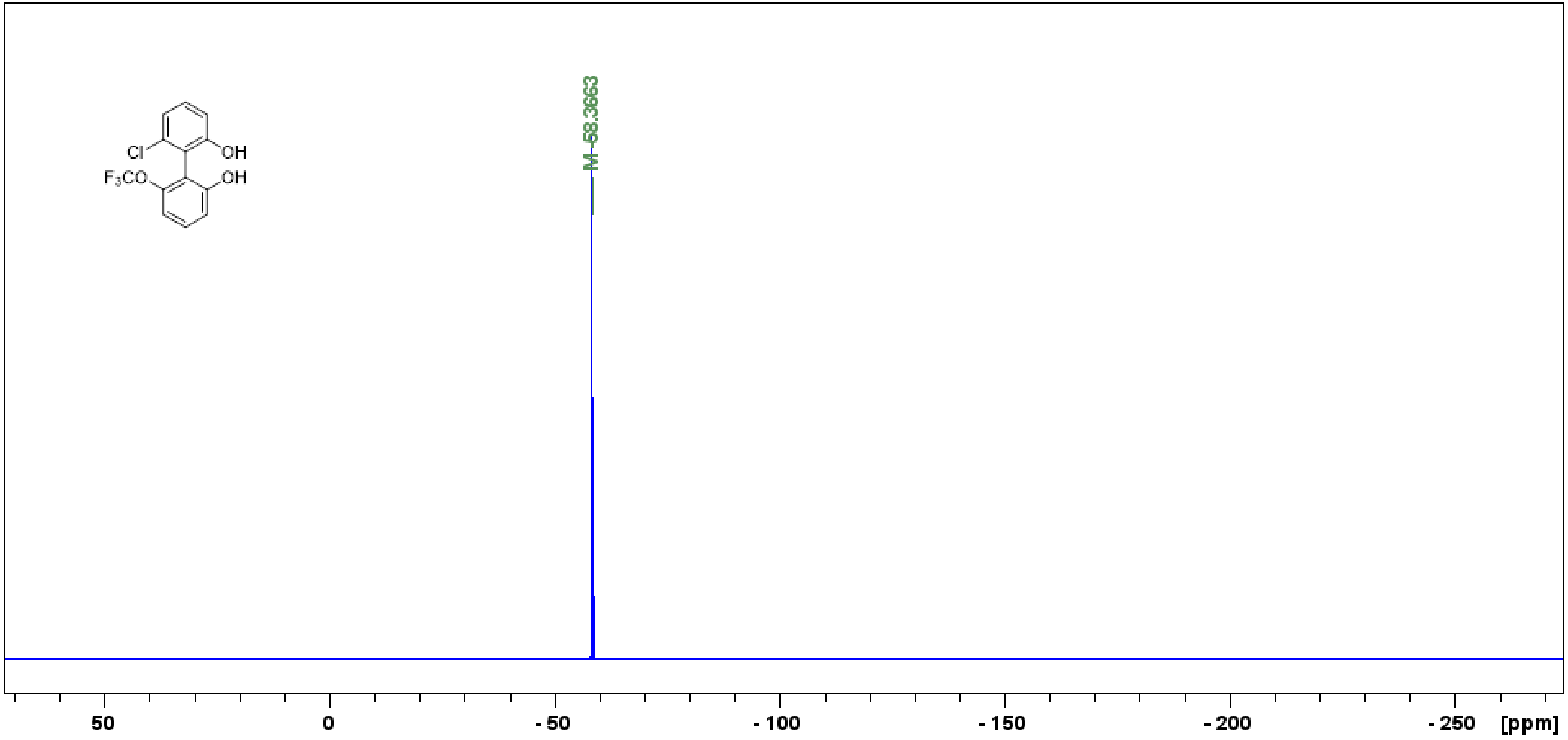

**$^{1}$H NMR** ($CDCl_3$)**:** 6-methyl-6'-(trifluoromethoxy)-[1,1'-biphenyl]-2,2'-diol (**3v**)

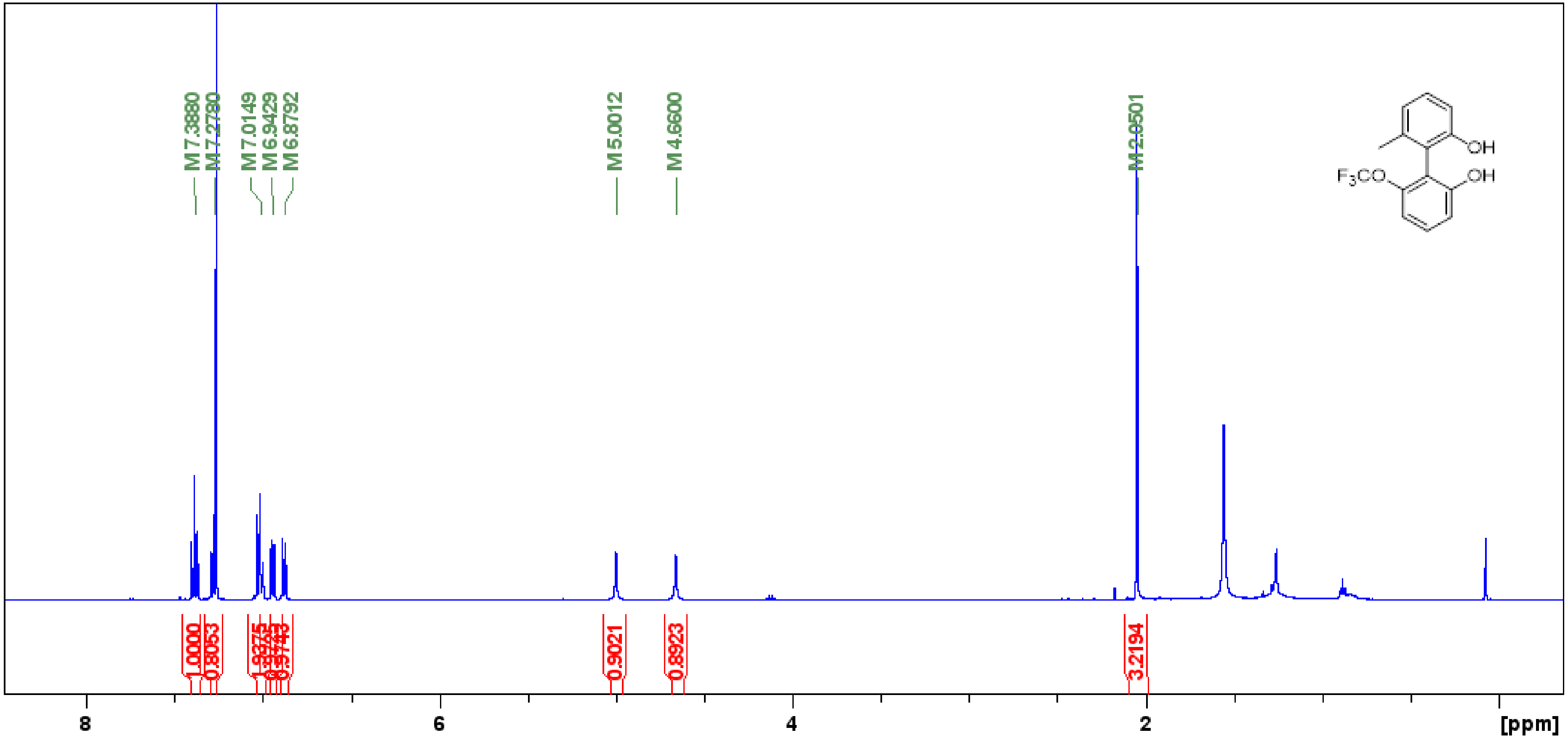

**$^{13}$C NMR** ($CDCl_3$)**:** 6-methyl-6'-(trifluoromethoxy)-[1,1'-biphenyl]-2,2'-diol (**3v**)

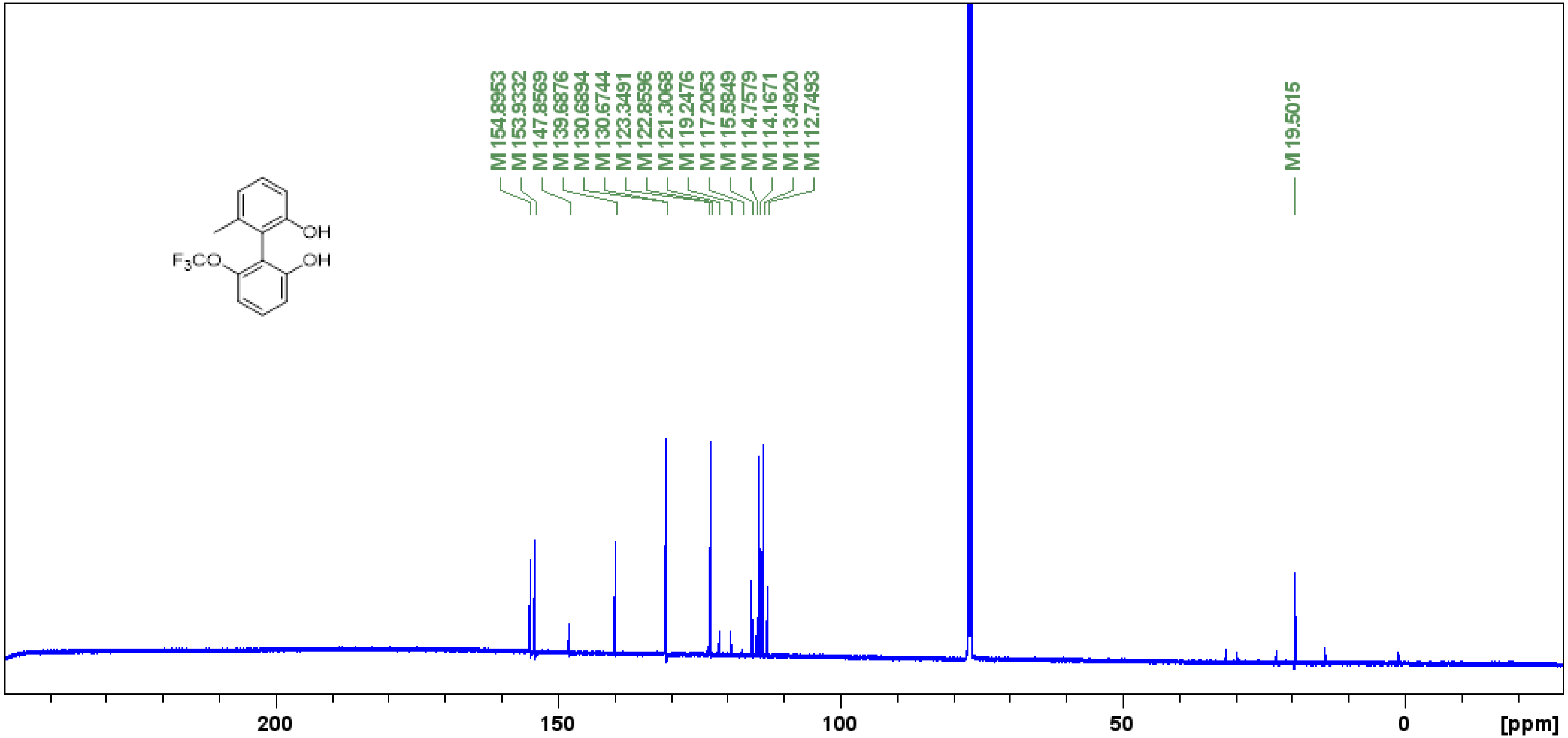

[19]**F NMR** (CDCl₃)**:** 6-methyl-6'-(trifluoromethoxy)-[1,1'-biphenyl]-2,2'-diol (**3v**)

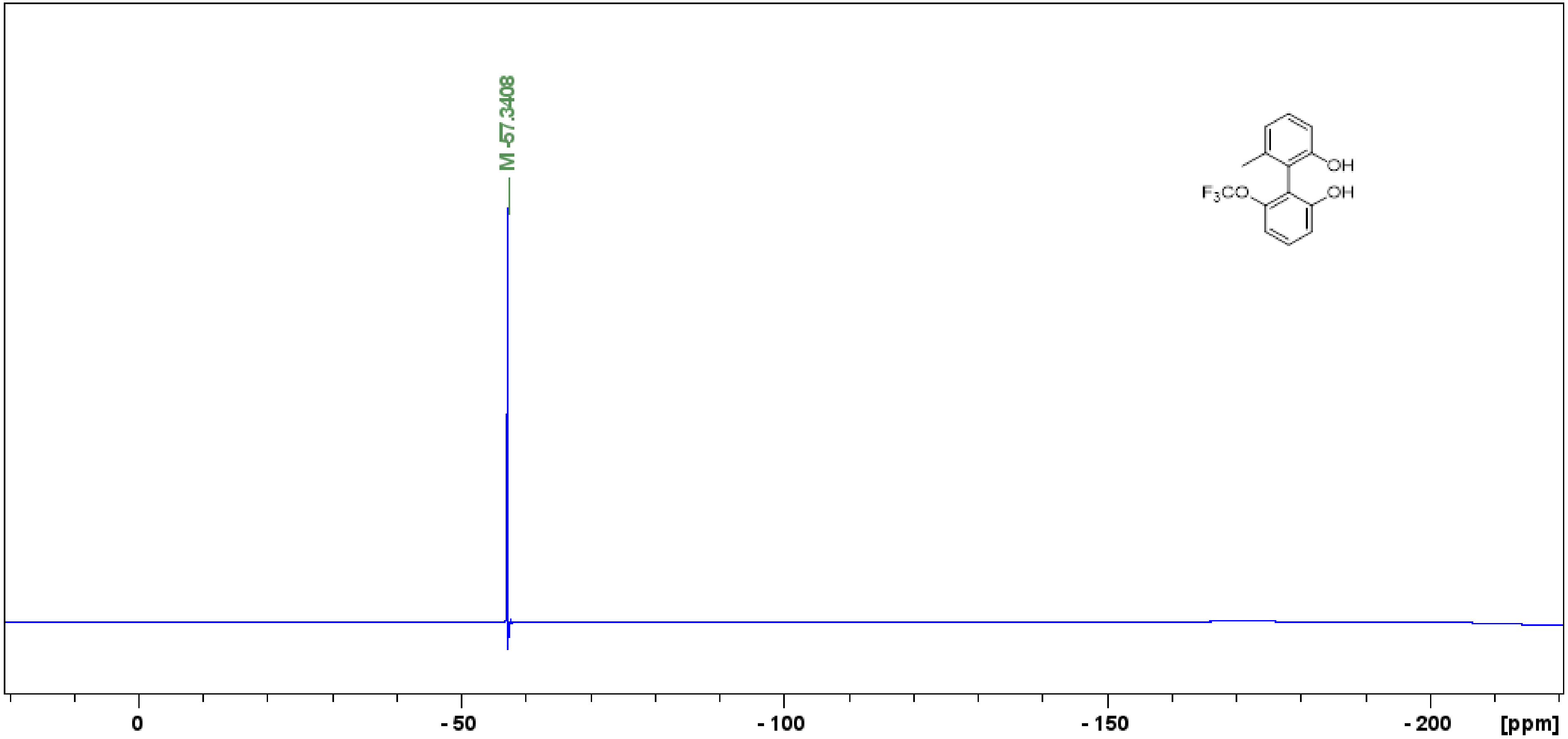

$^1$H NMR ($CDCl_3$) ethyl (*R*,*E*)-3-(2'-fluoro-6,6'-dihydroxy-[1,1'-biphenyl]-2-yl)acrylate

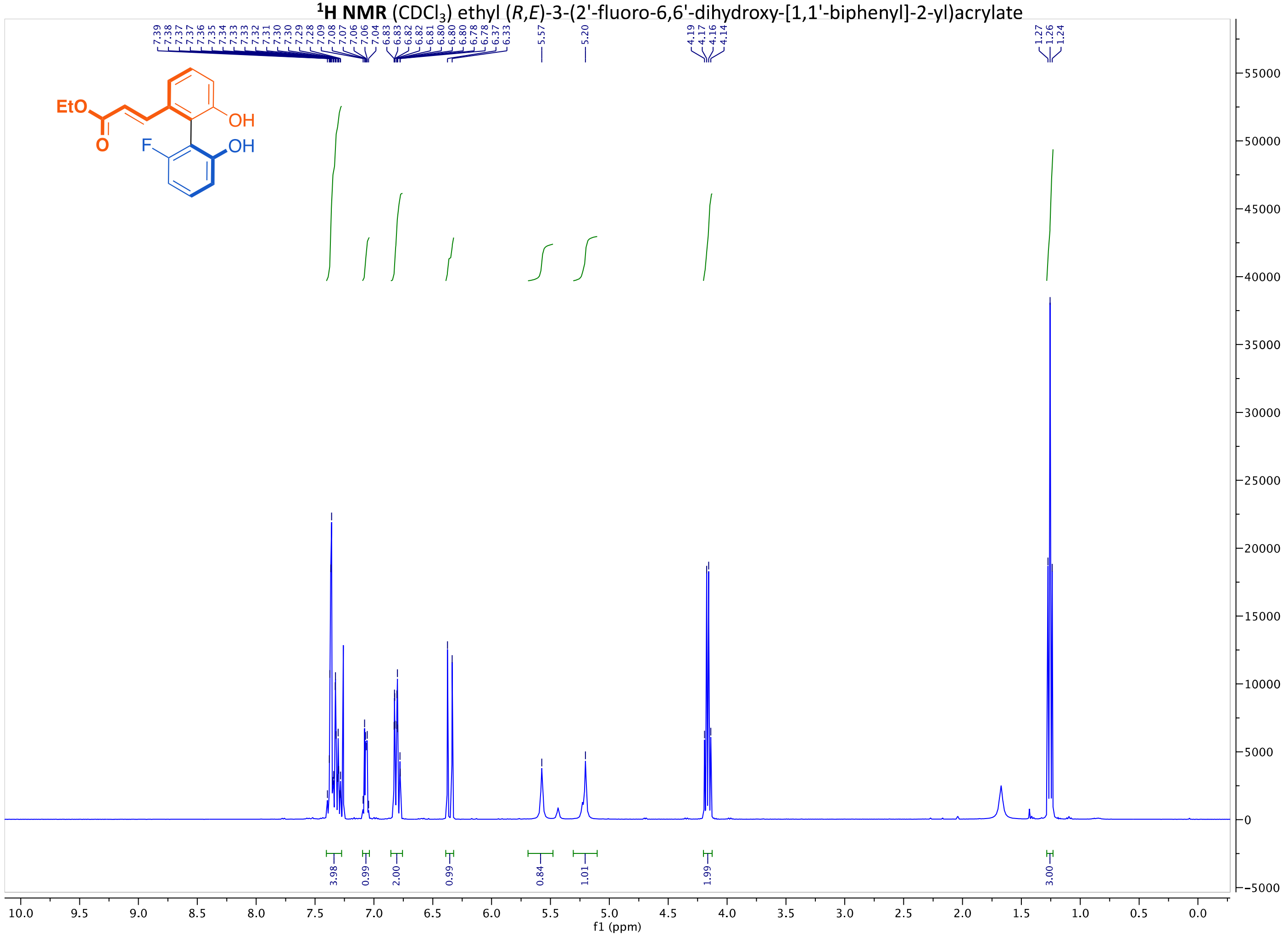

**$^{13}$C NMR** ($CDCl_3$) ethyl (*R*,*E*)-3-(2'-fluoro-6,6'-dihydroxy-[1,1'-biphenyl]-2-yl)acrylate

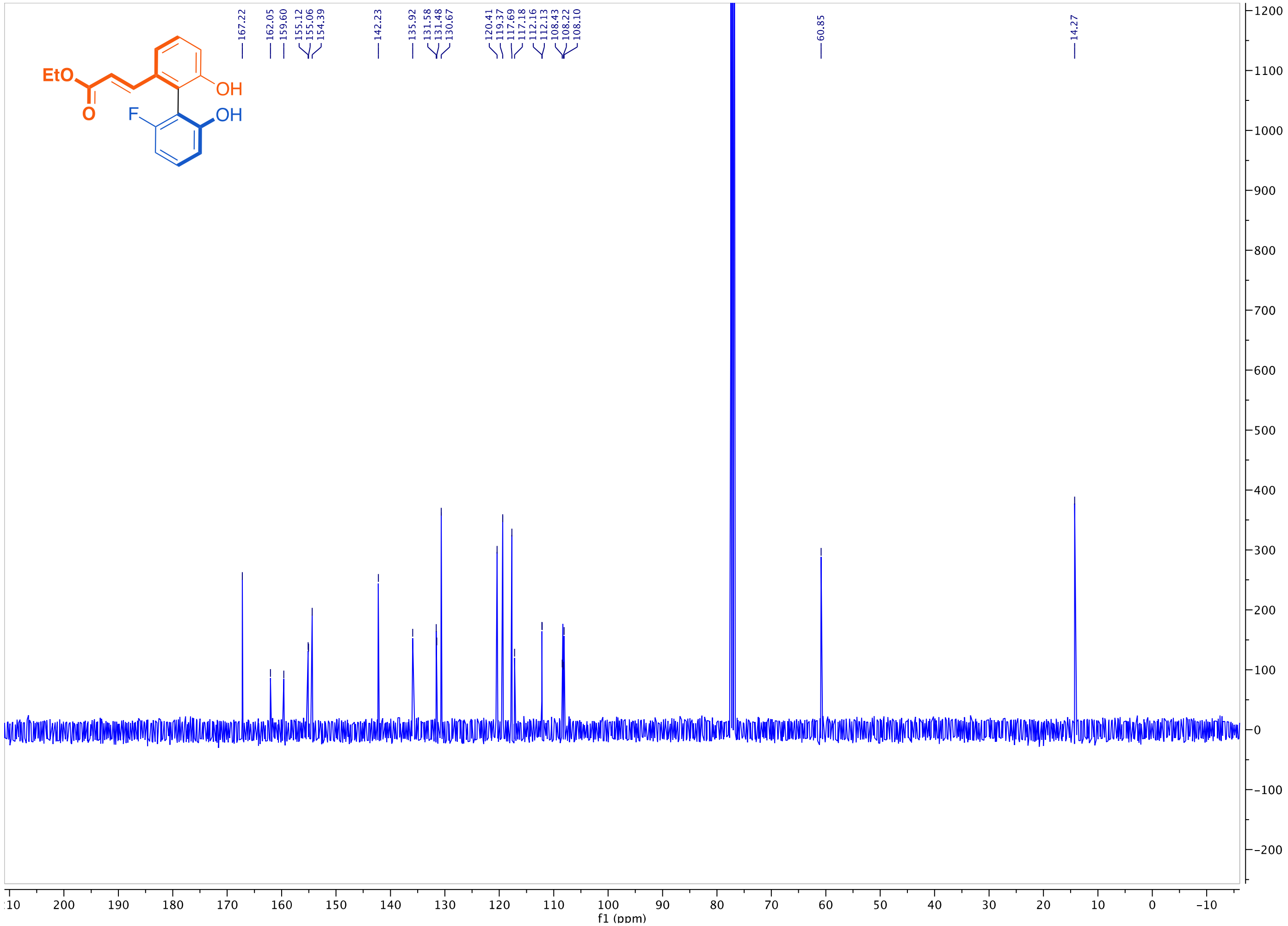

**$^{19}$F NMR** ($CDCl_3$) ethyl (*R*,*E*)-3-(2'-fluoro-6,6'-dihydroxy-[1,1'-biphenyl]-2-yl)acrylate

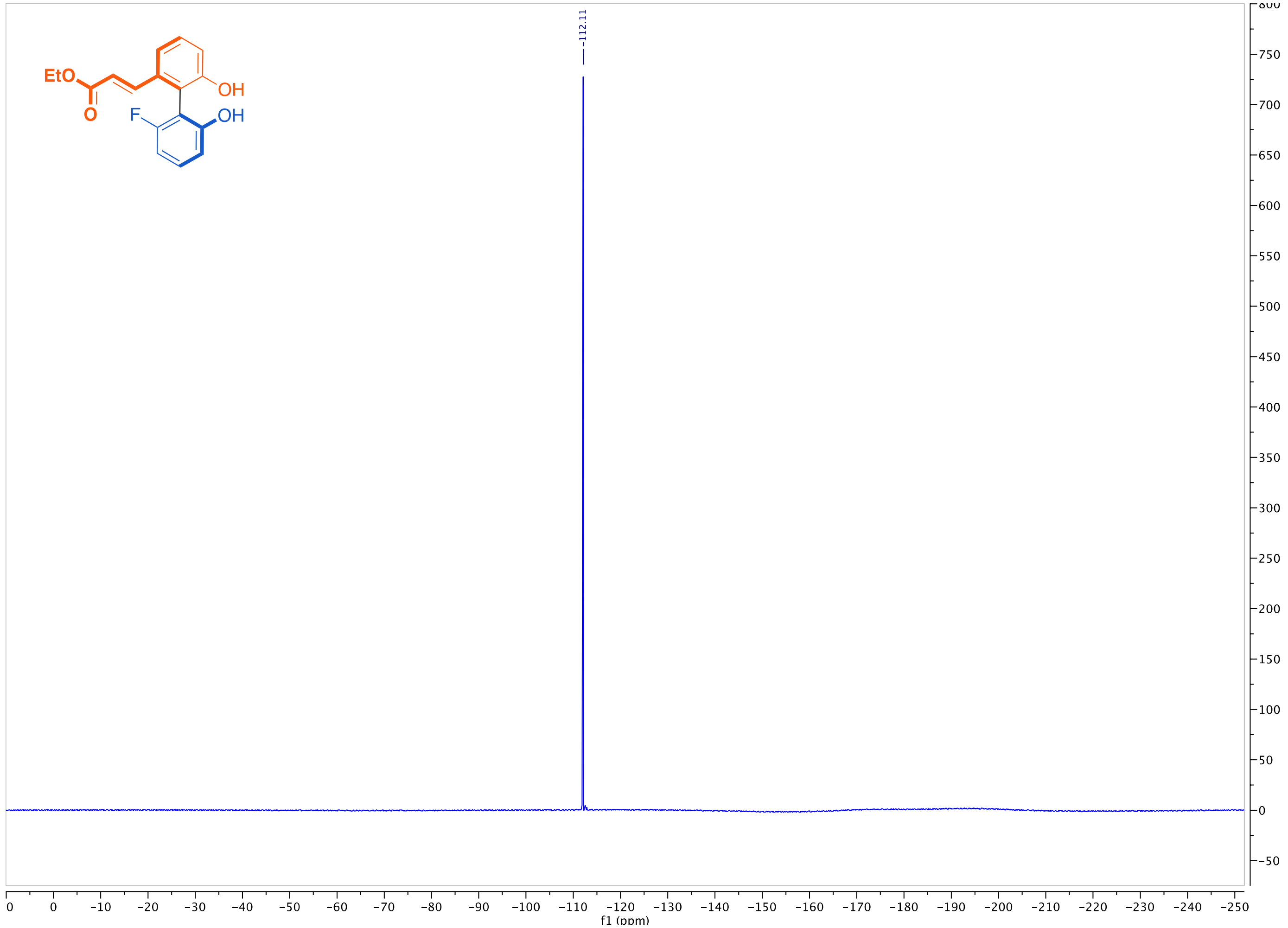

**$^{1}$H NMR** ($CD_3OD$): 6,6''-difluoro-4',6'-dichloro-[1,1':3',1''-terphenyl]-2,2',2''-triol (**5a**)

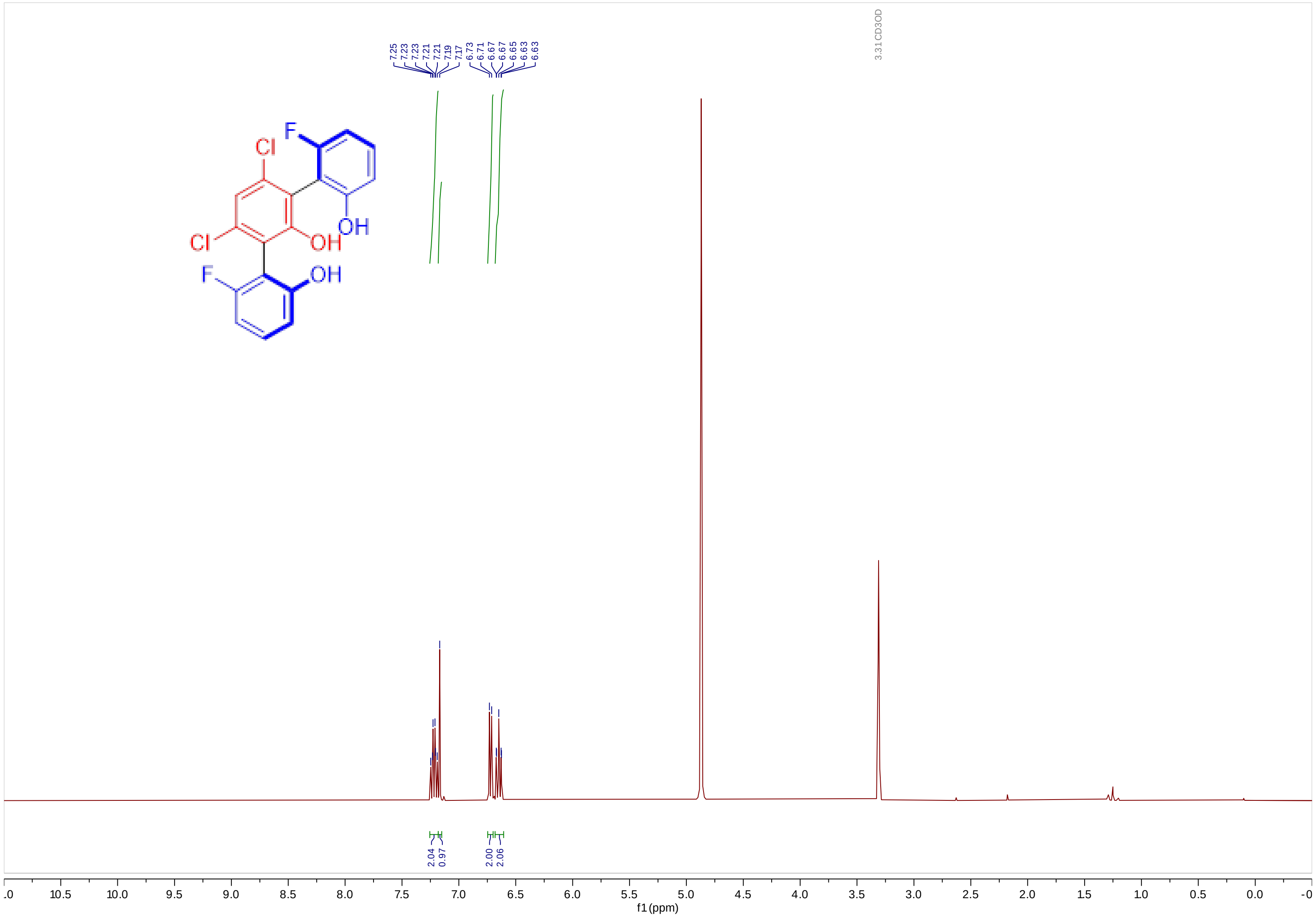

**$^{13}$C NMR** ($CD_3OD$): 6,6''-difluoro-4',6'-dichloro-[1,1':3',1''-terphenyl]-2,2',2''-triol (**5a**)

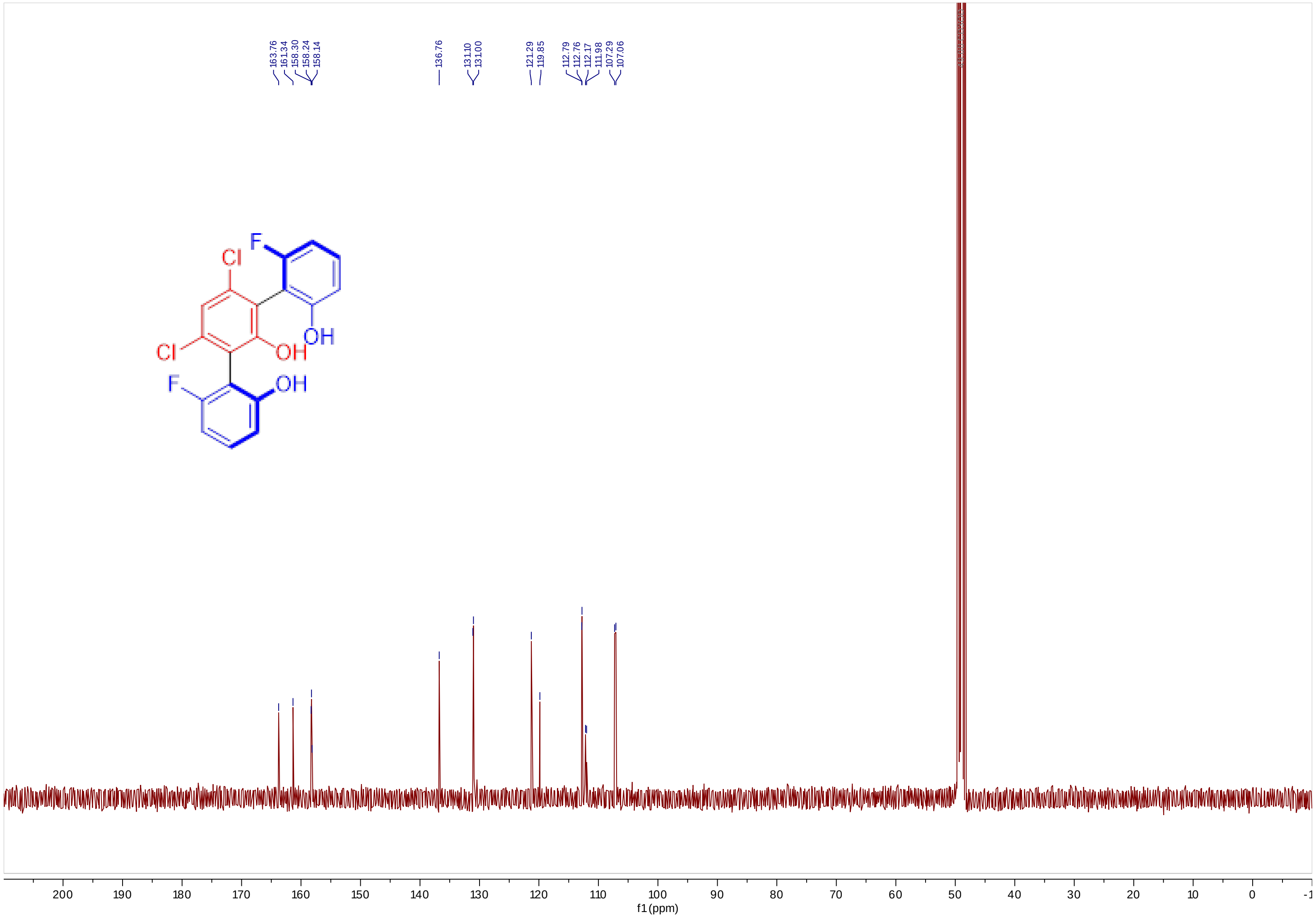

**¹⁹F NMR** ($CD_3OD$): 6,6''-difluoro-4',6'-dichloro-[1,1':3',1''-terphenyl]-2,2',2''-triol (**5a**)

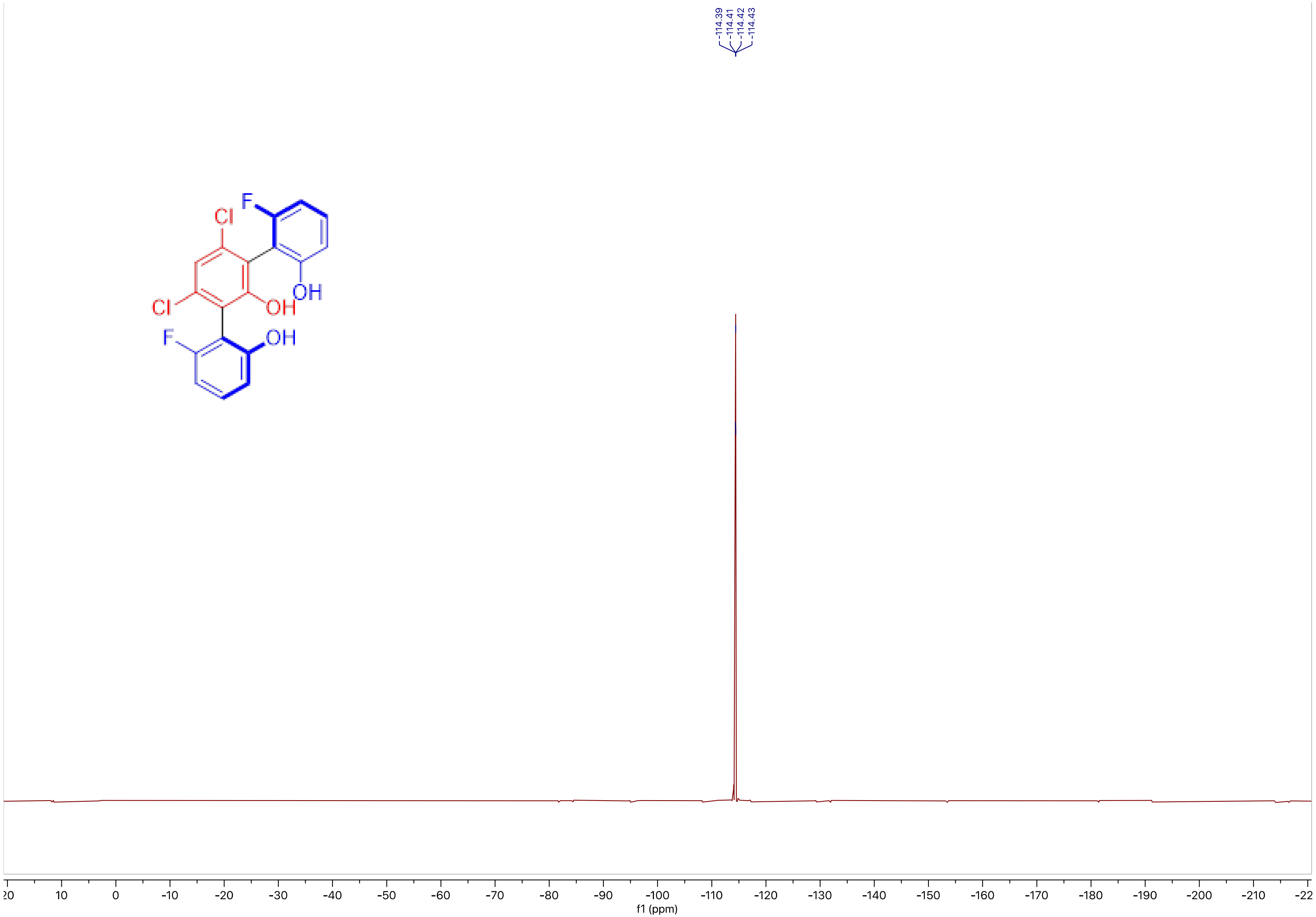

**$^{1}$H NMR** ($CD_3OD$): 6,6''-difluoro-4',6'-dichloro-[1,1':3',1''-terphenyl]-2,2',2''-triol (**5b**)

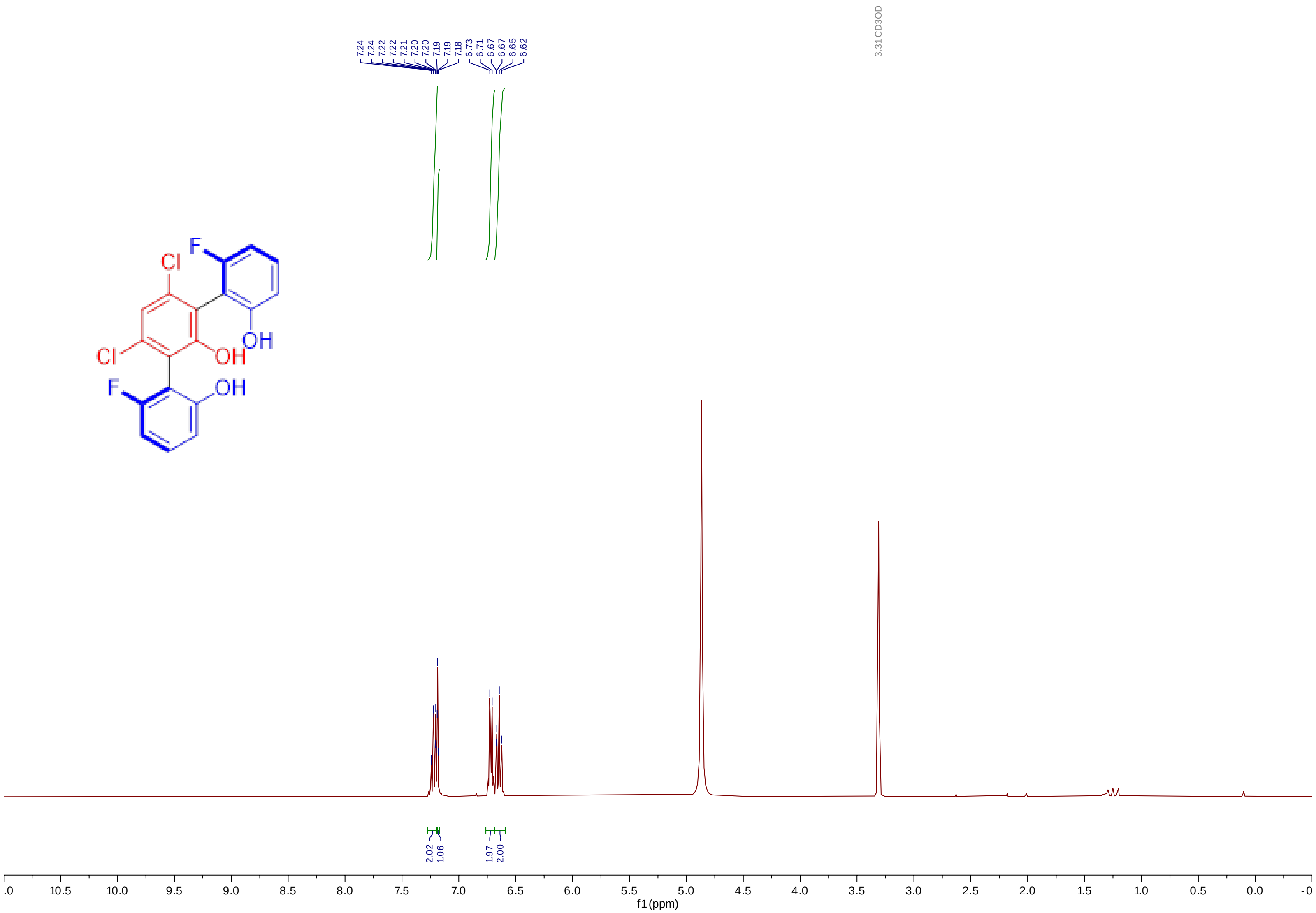

**$^{13}$C NMR** ($CD_3OD$): 6,6''-difluoro-4',6'-dichloro-[1,1':3',1''-terphenyl]-2,2',2''-triol (**5b**)

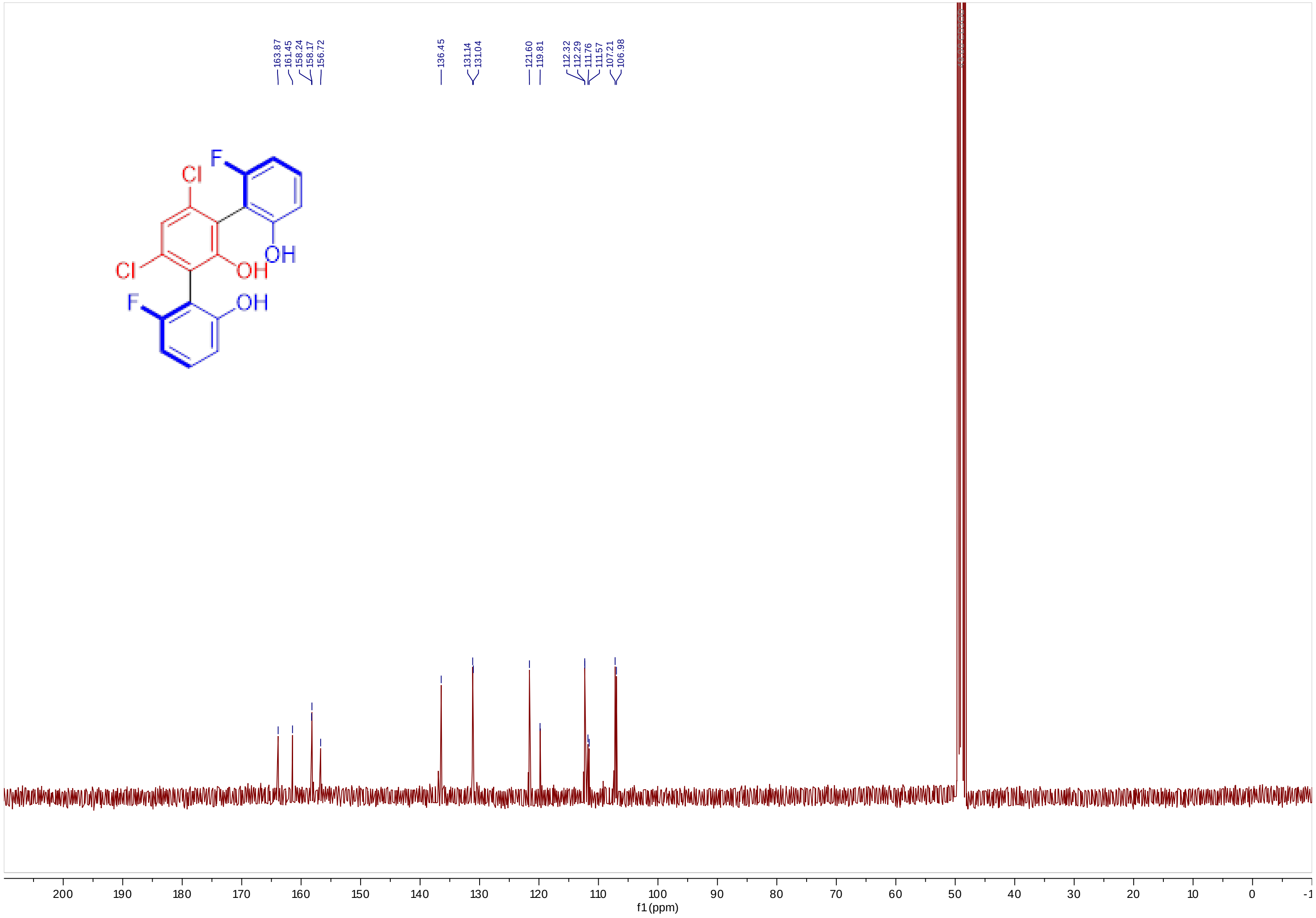

**$^{19}$F NMR** ($CD_3OD$): 6,6''-difluoro-4',6'-dichloro-[1,1':3',1''-terphenyl]-2,2',2''-triol (**5b**)

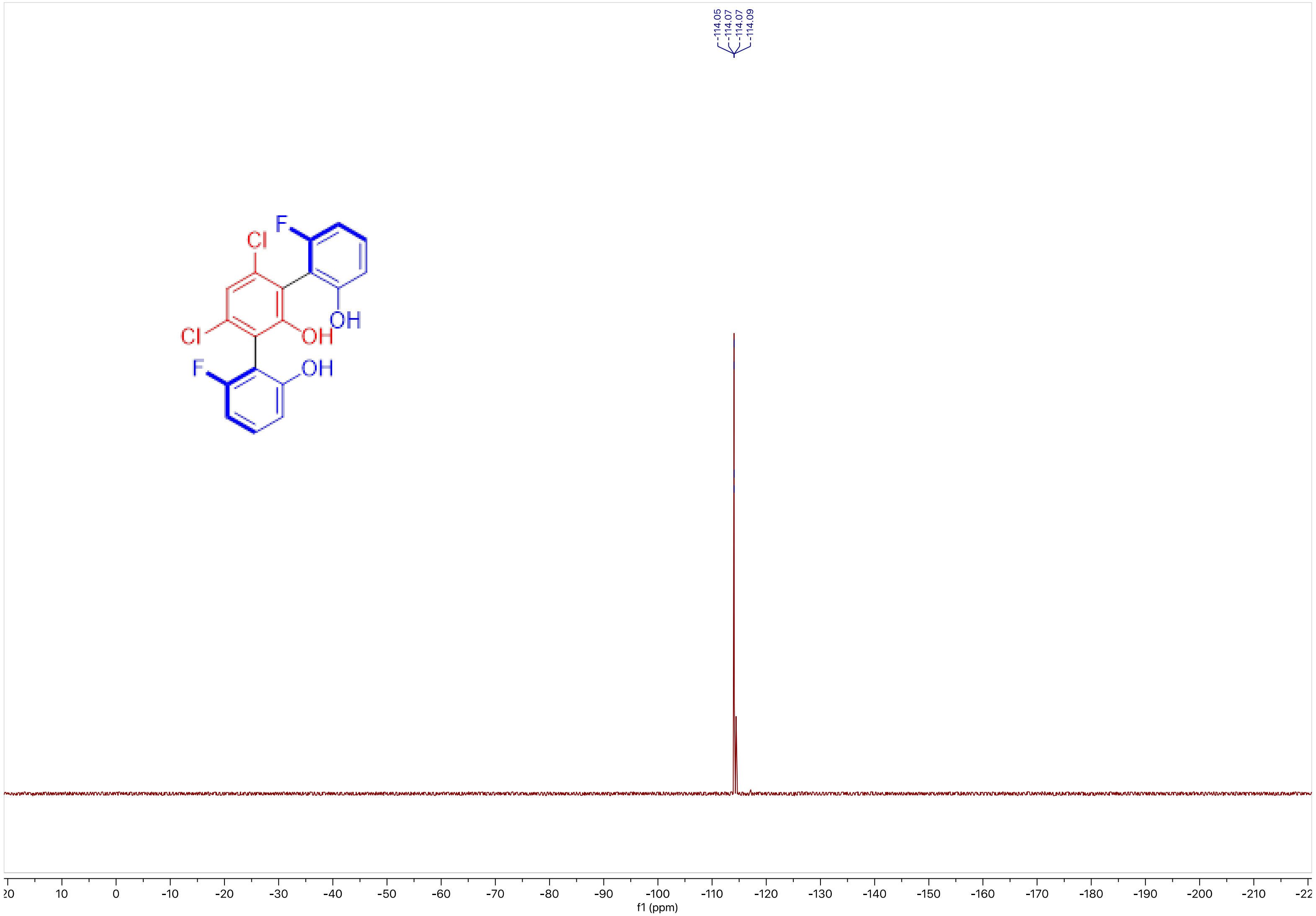

**$^{1}$H NMR** ($CDCl_3$): 6,6''-difluoro-4',6'-dimethyl-[1,1':3',1''-terphenyl]-2,2',2''-triol (**5c**)

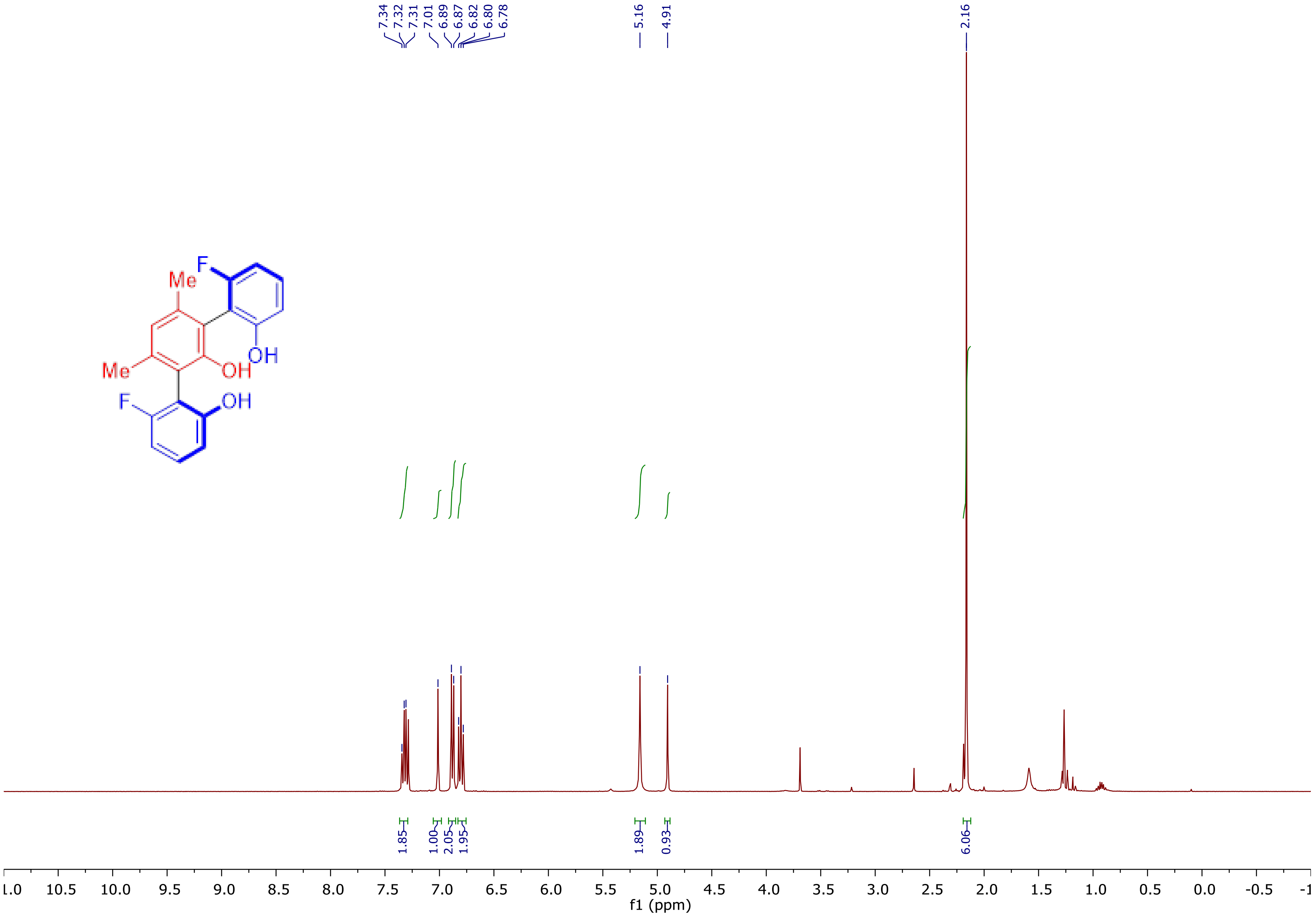

$^{13}$**C NMR** (CDCl$_3$): 6,6''-difluoro-4',6'-dimethyl-[1,1':3',1''-terphenyl]-2,2',2''-triol (**5c**)

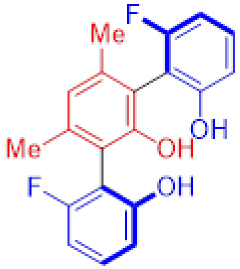


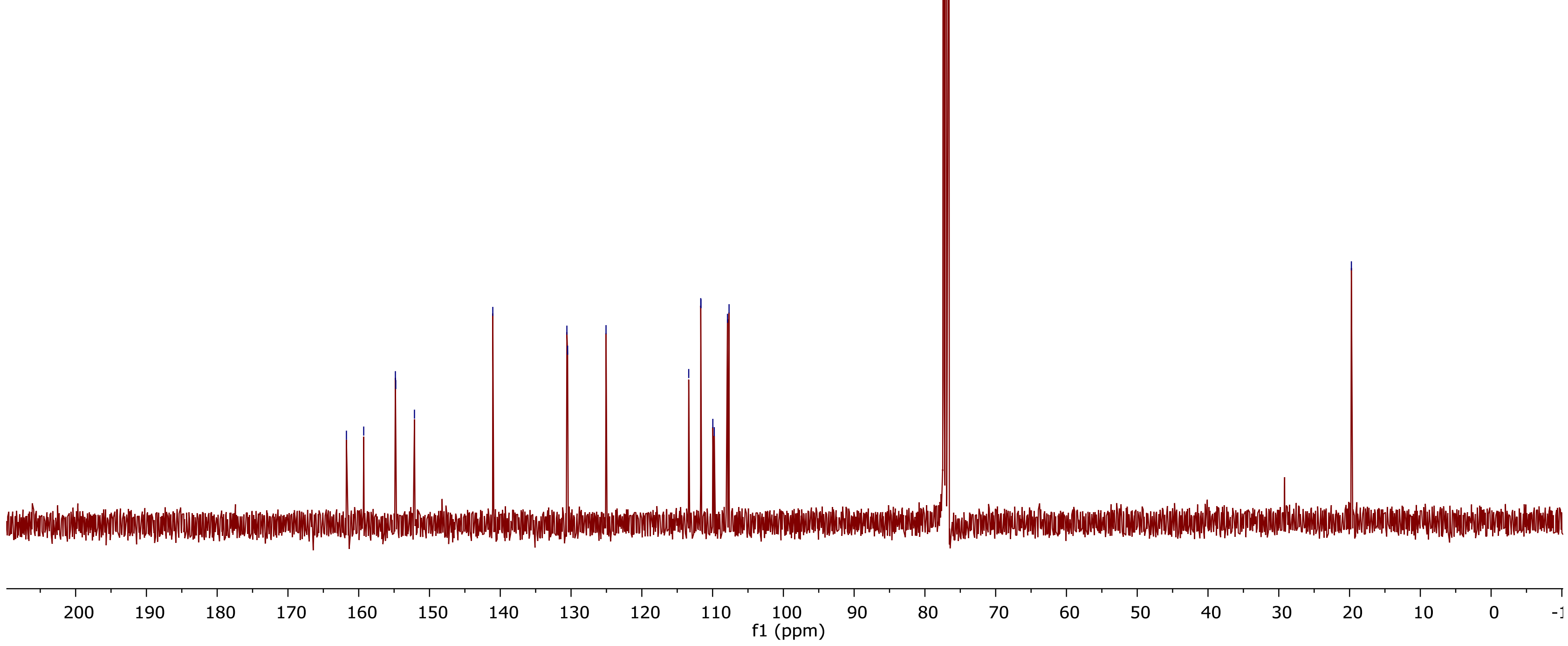

**$^{19}$F NMR** ($CDCl_3$): 6,6''-difluoro-4',6'-dimethyl-[1,1':3',1''-terphenyl]-2,2',2''-triol (**5c**)

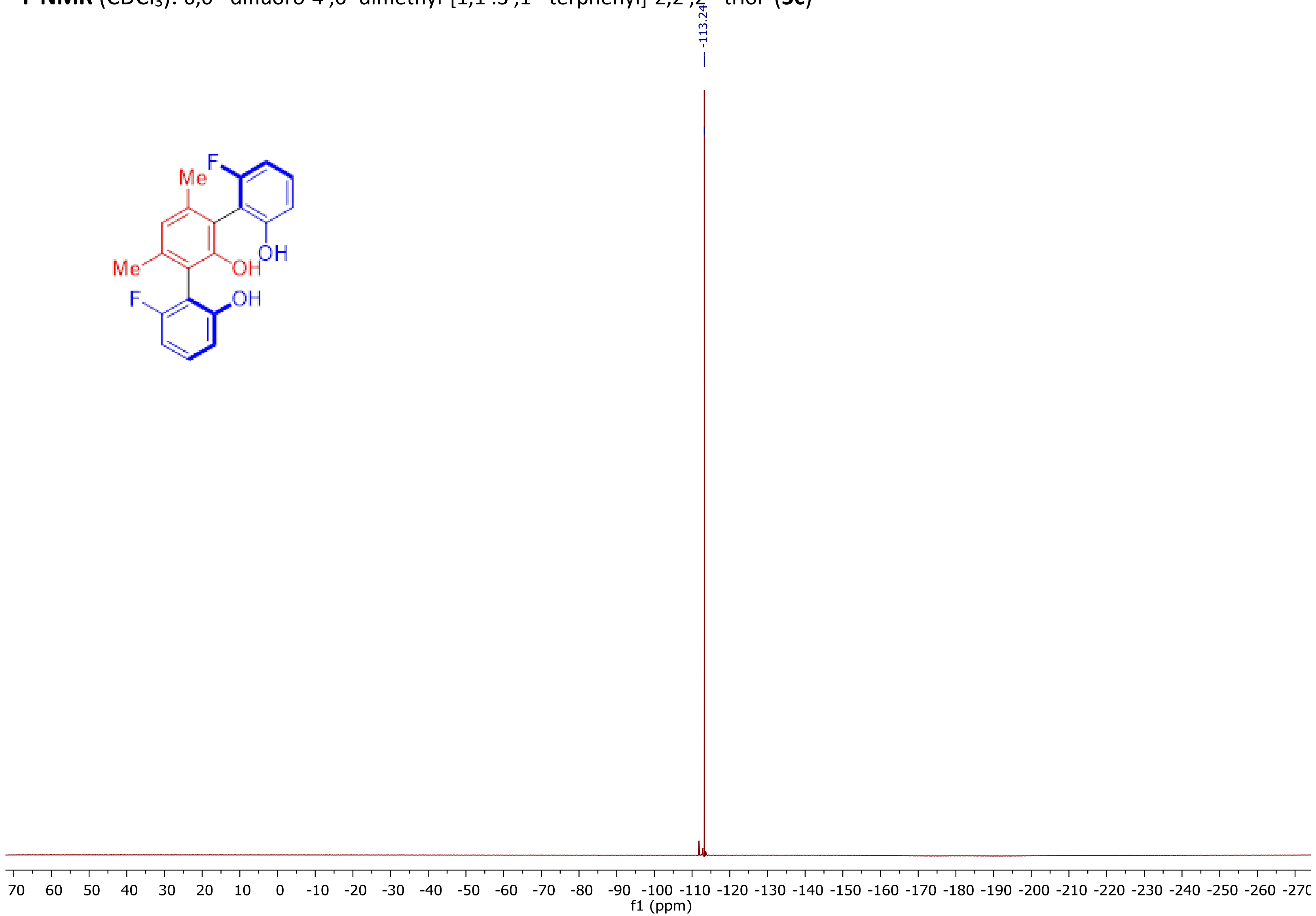

**$^{1}$H NMR** ($CDCl_3$): 6,6''-difluoro-4',6'-dimethyl-[1,1':3',1''-terphenyl]-2,2',2''-triol (**5d**)

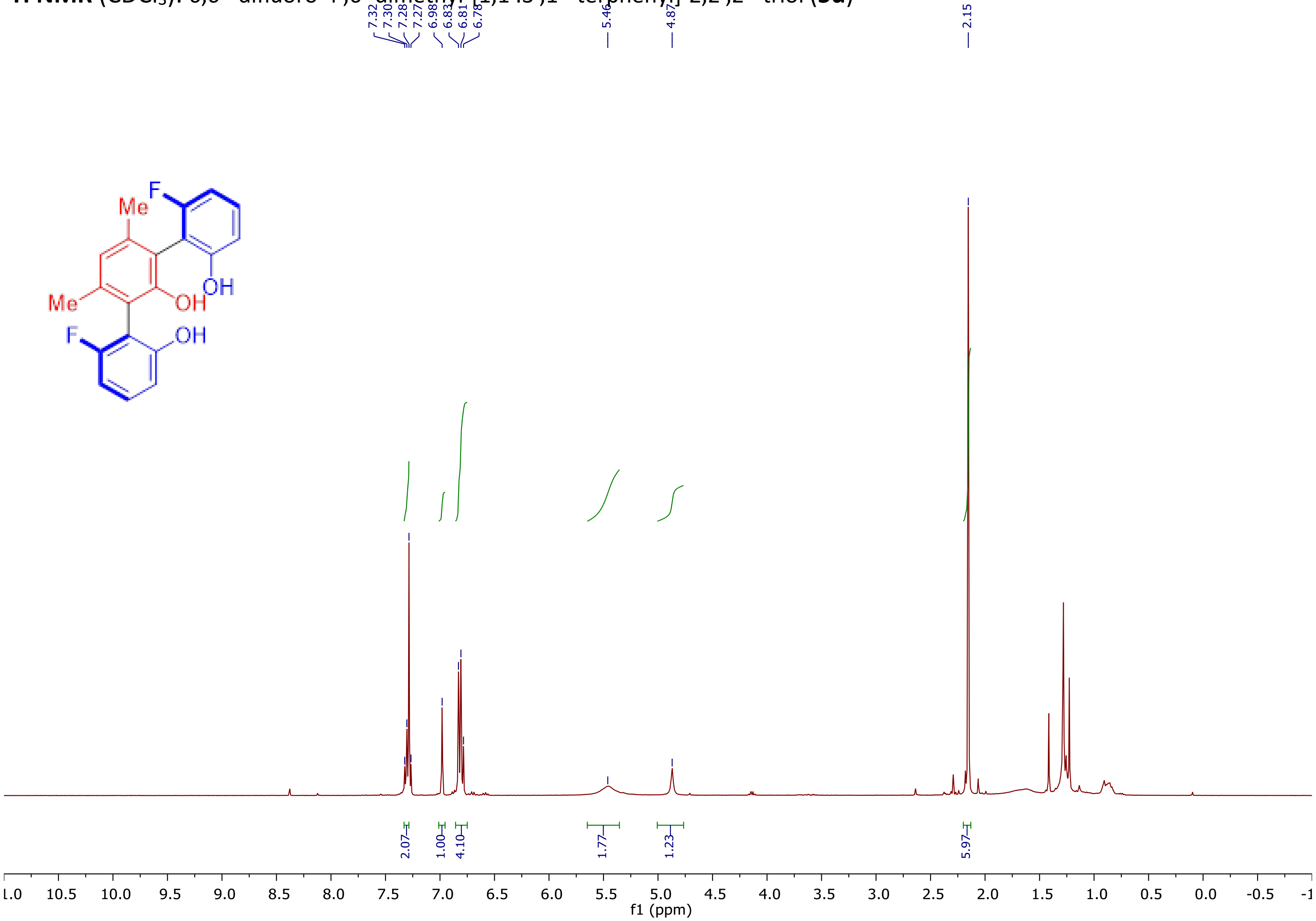

**$^{13}$C NMR** ($CDCl_3$): 6,6''-difluoro-4',6'-dimethyl-[1,1':3',1''-terphenyl]-2,2',2''-triol (**5d**)

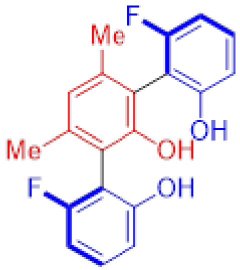

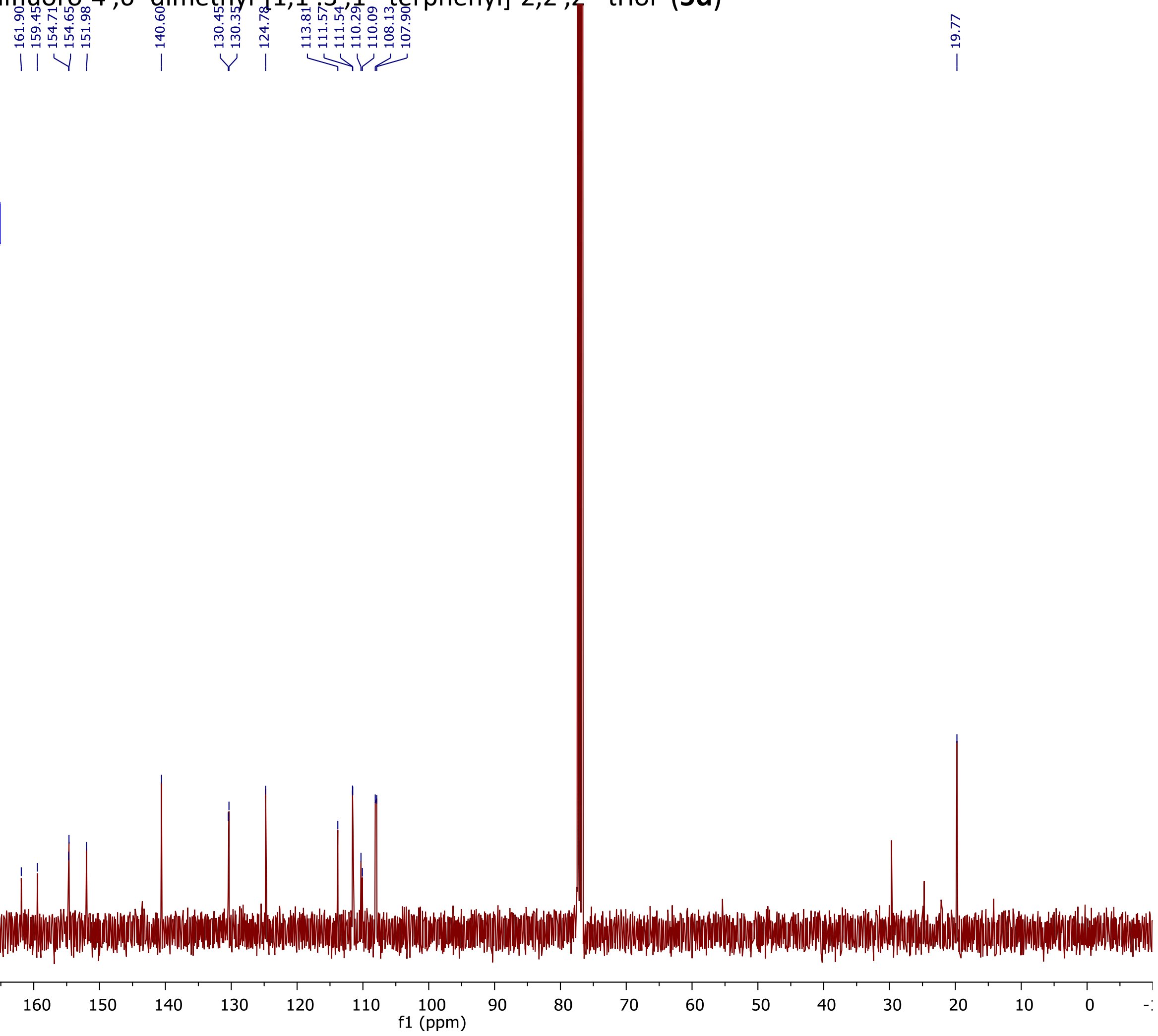

**19F NMR** (CDCl3): 6,6''-difluoro-4',6'-dimethyl-[1,1':3',1''-terphenyl]-2,2',2''-triol (**5d**)

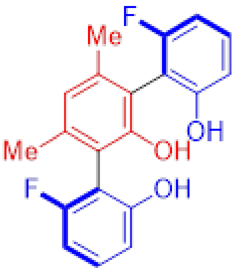


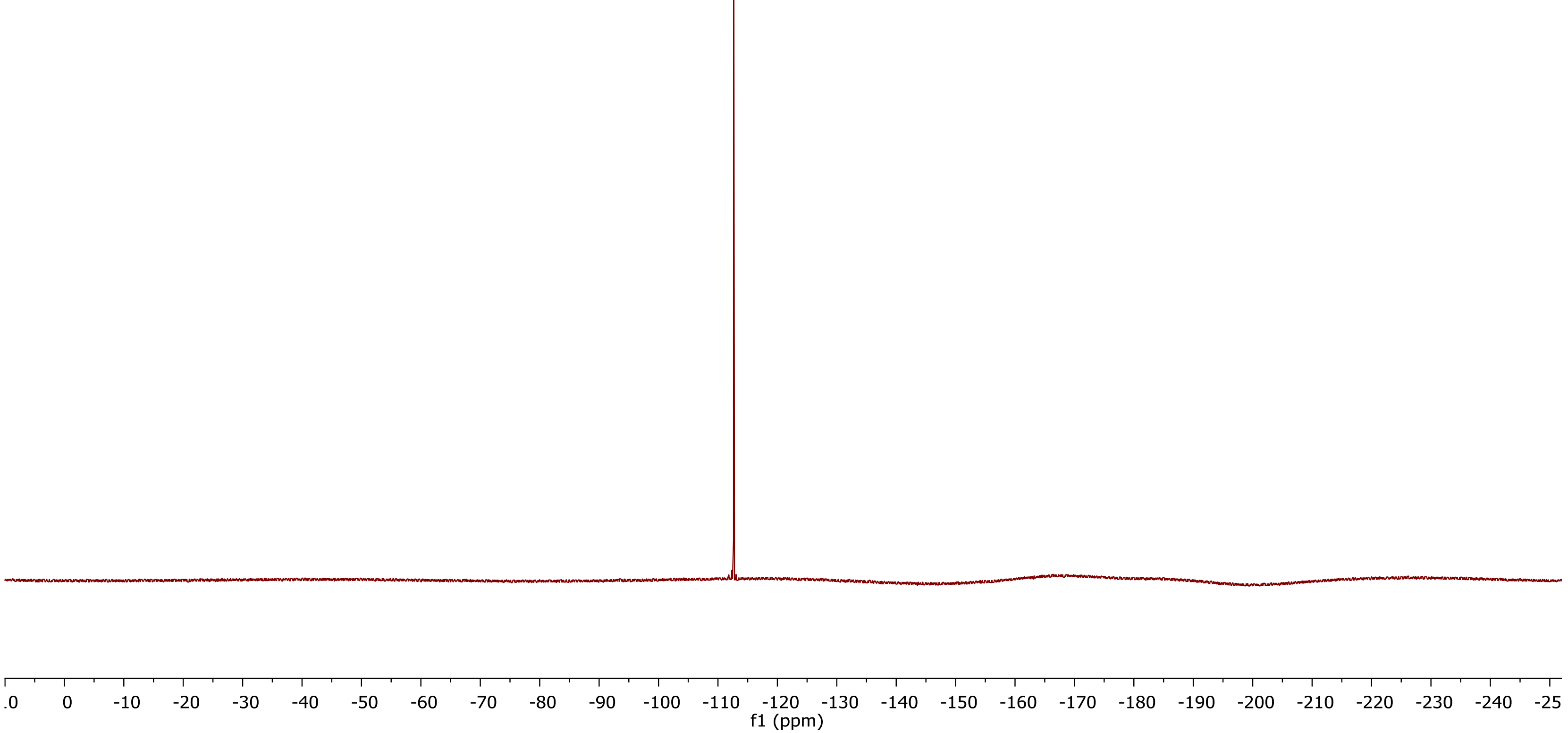

**$^{1}$H NMR** ($CDCl_3$): 6-fluoro-2'-methoxy-6'-methyl-[1,1'-biphenyl]-2-ol (**6a**)

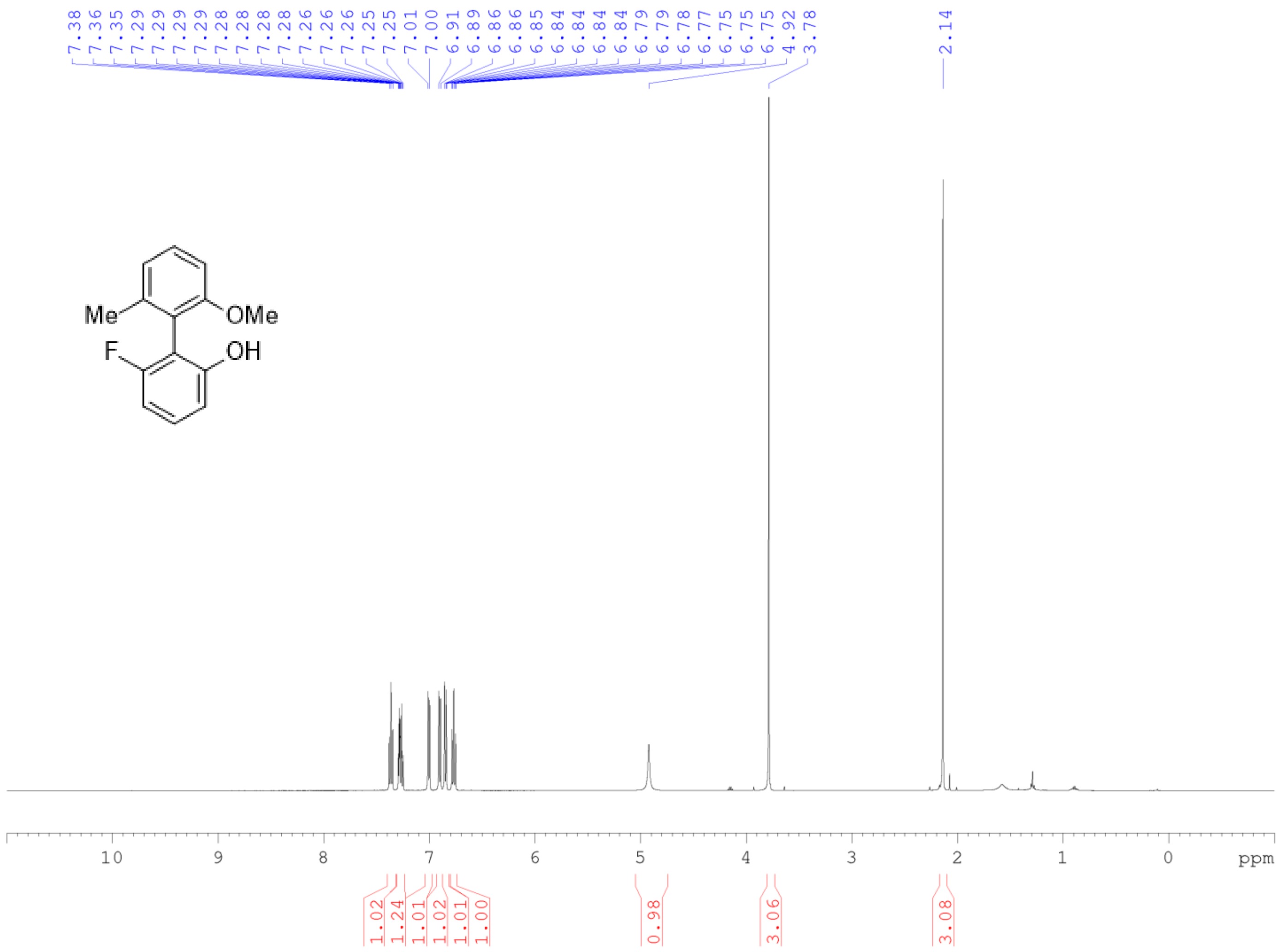

**$^{13}$C NMR** ($CDCl_3$): 6-fluoro-2'-methoxy-6'-methyl-[1,1'-biphenyl]-2-ol (**6a**)

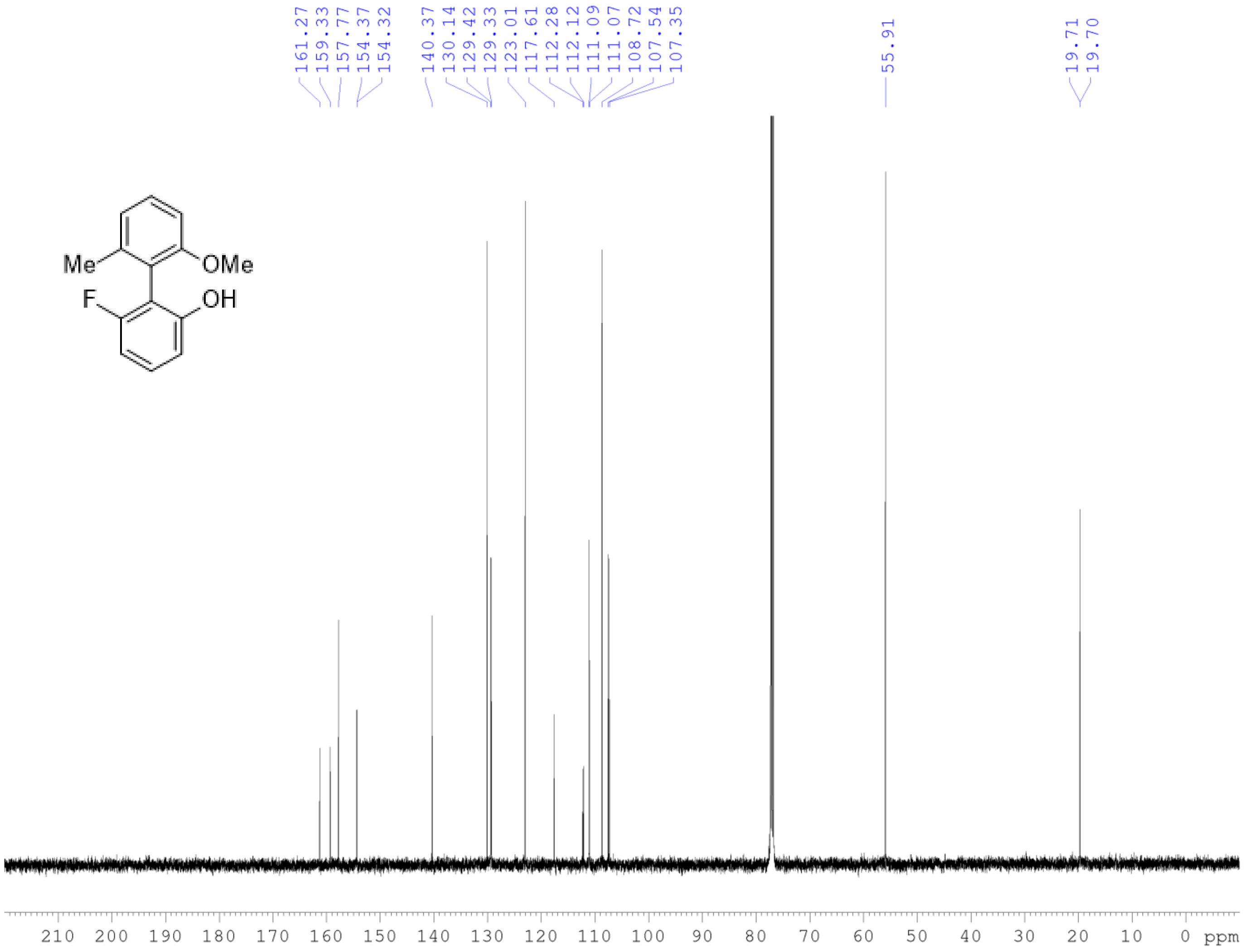

$^{19}$**F NMR** ($CDCl_3$): 6-fluoro-2'-methoxy-6'-methyl-[1,1'-biphenyl]-2-ol (**6a**)

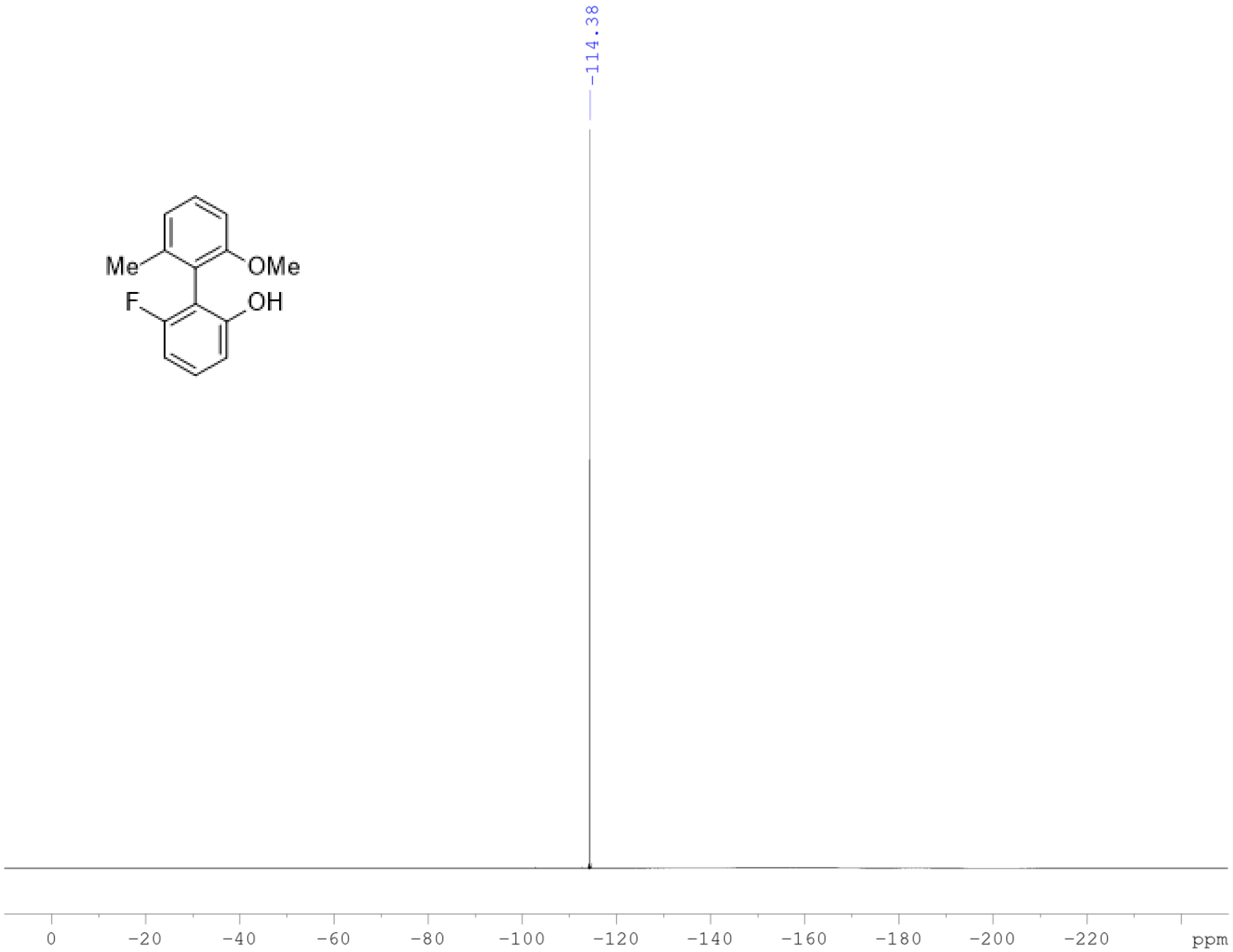

**$^{1}$H NMR** (CDCl$_3$): 2'-fluoro-6'-methoxy-6-methyl-[1,1'-biphenyl]-2-ol (**6b**)

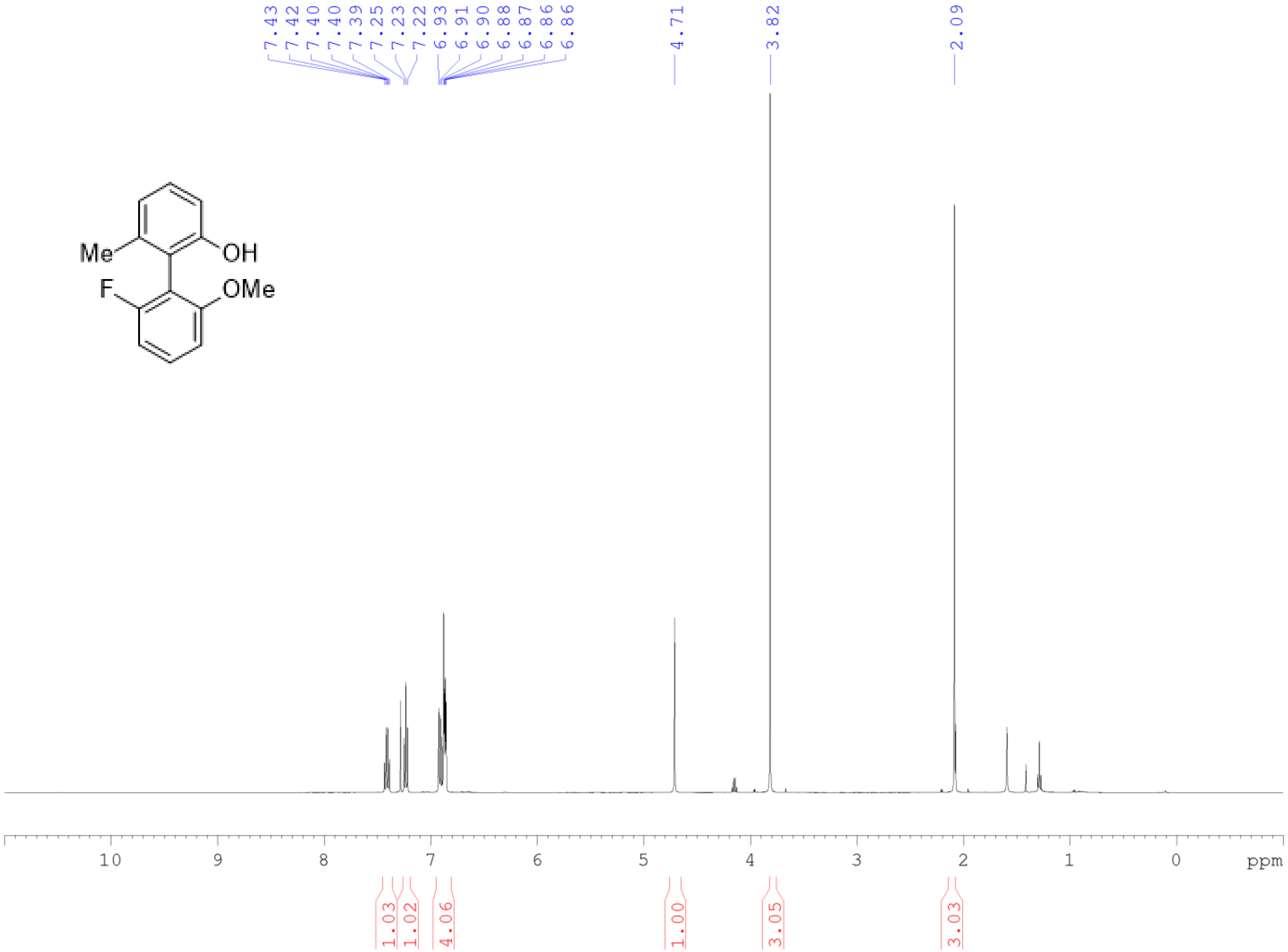

**$^{13}$C NMR** ($CDCl_3$): 2'-fluoro-6'-methoxy-6-methyl-[1,1'-biphenyl]-2-ol (**6b**)

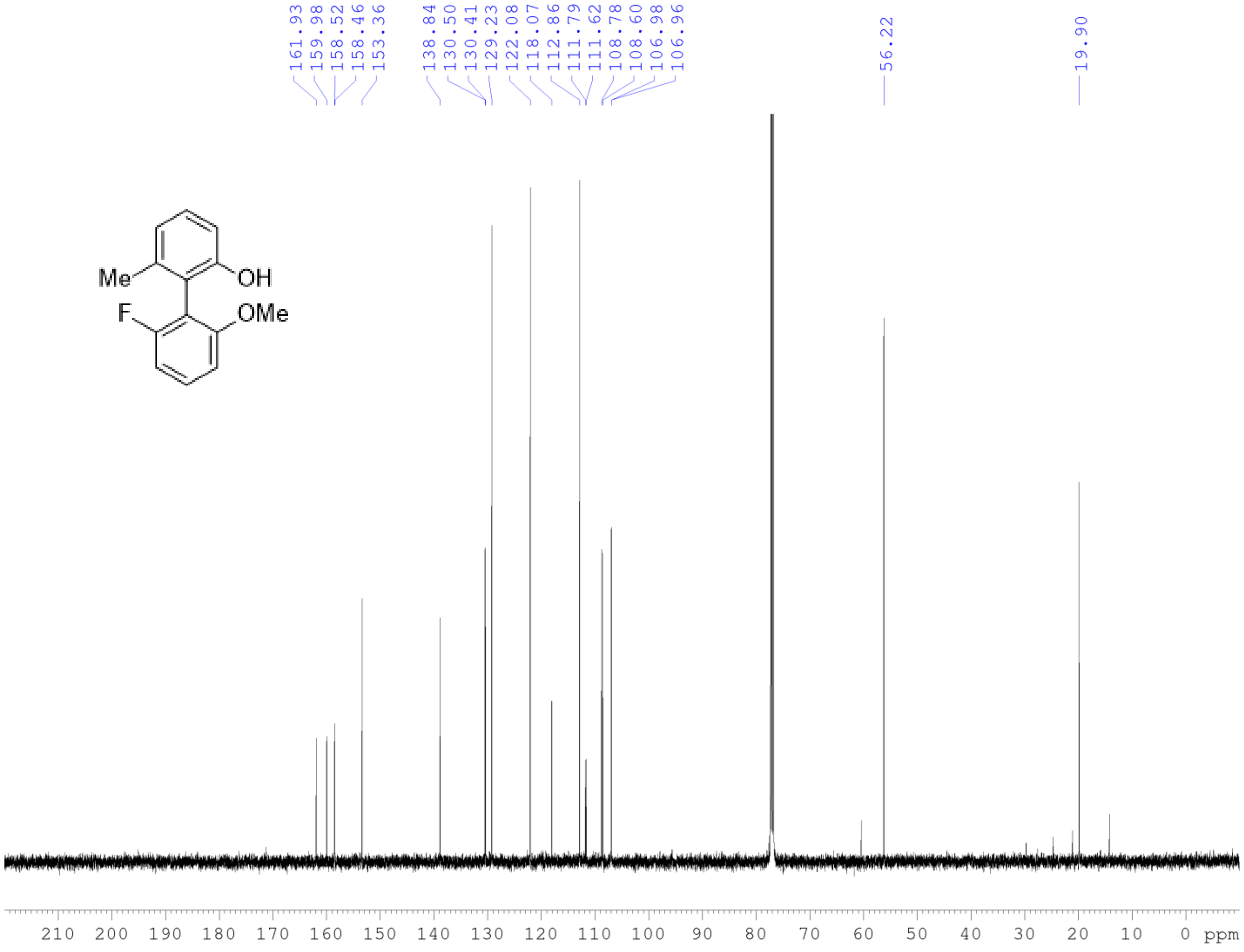

**¹⁹F NMR** ($CDCl_3$): 2'-fluoro-6'-methoxy-6-methyl-[1,1'-biphenyl]-2-ol (**6b**)

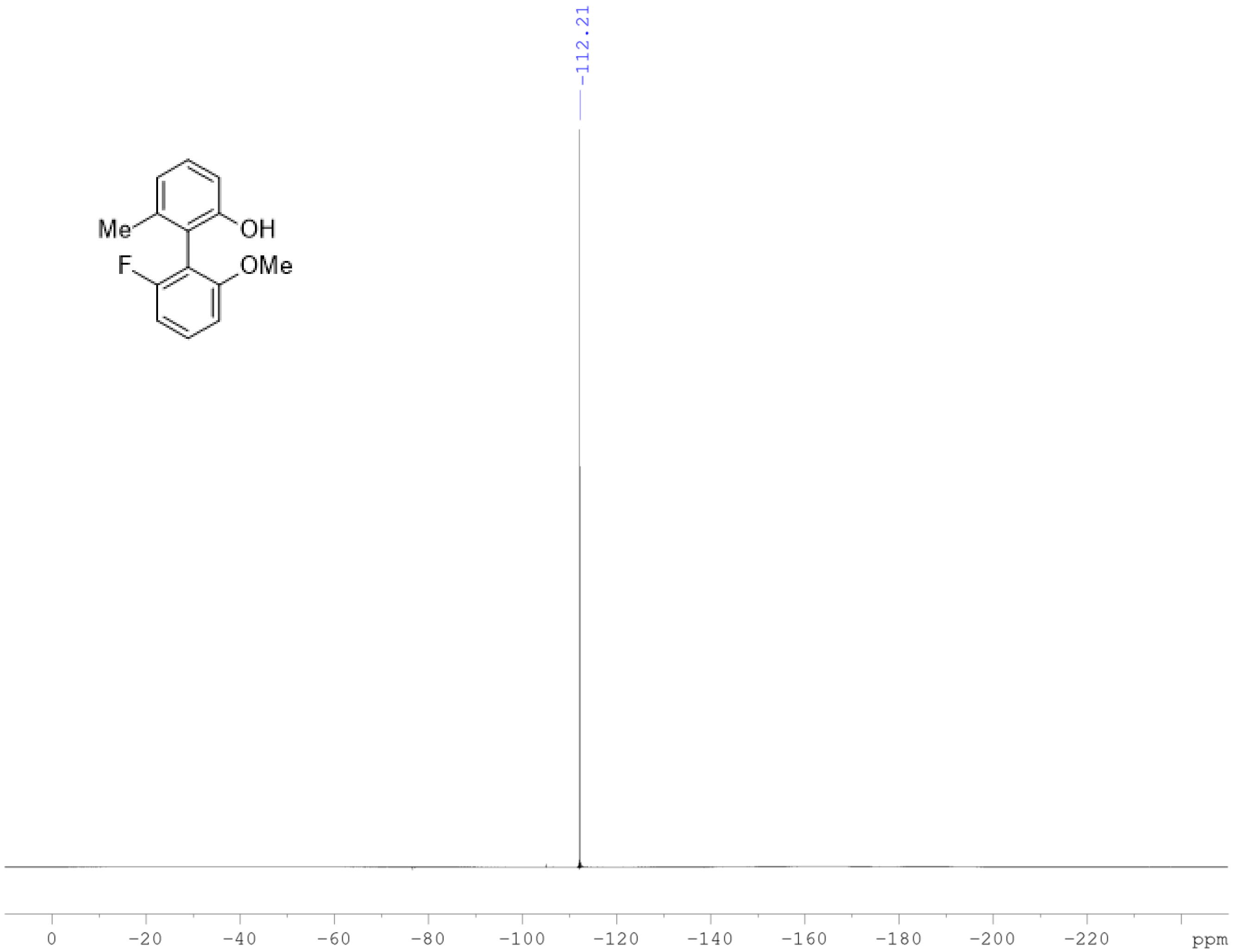

**$^1$H NMR** ($CDCl_3$): 2-fluoro-2',6-dimethoxy-6'-methyl-1,1'-biphenyl (**6c**)

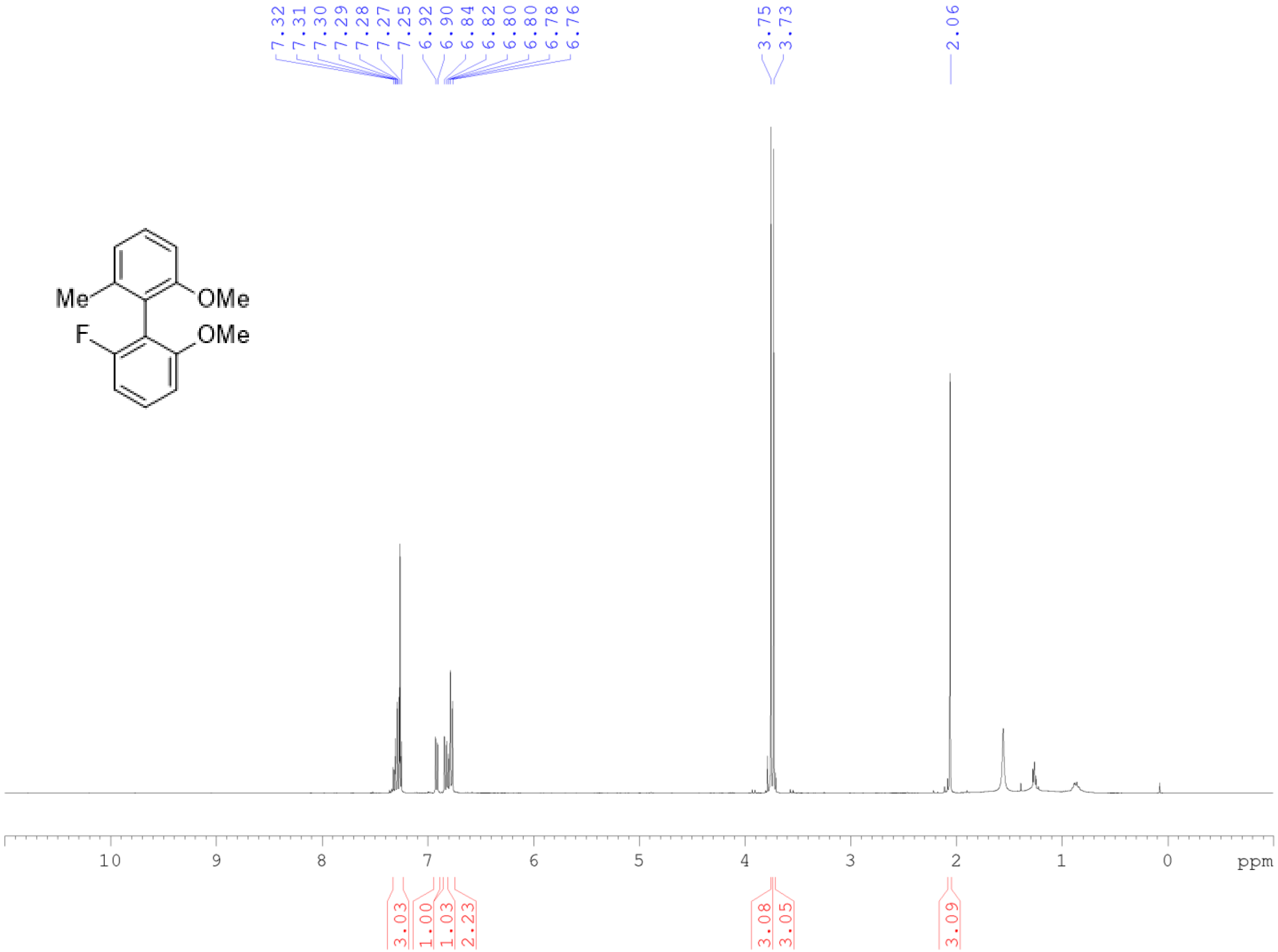

**$^{13}$C NMR** ($CDCl_3$): 2-fluoro-2',6-dimethoxy-6'-methyl-1,1'-biphenyl (**6c**)

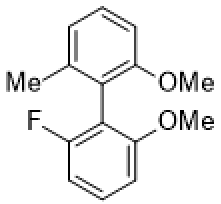


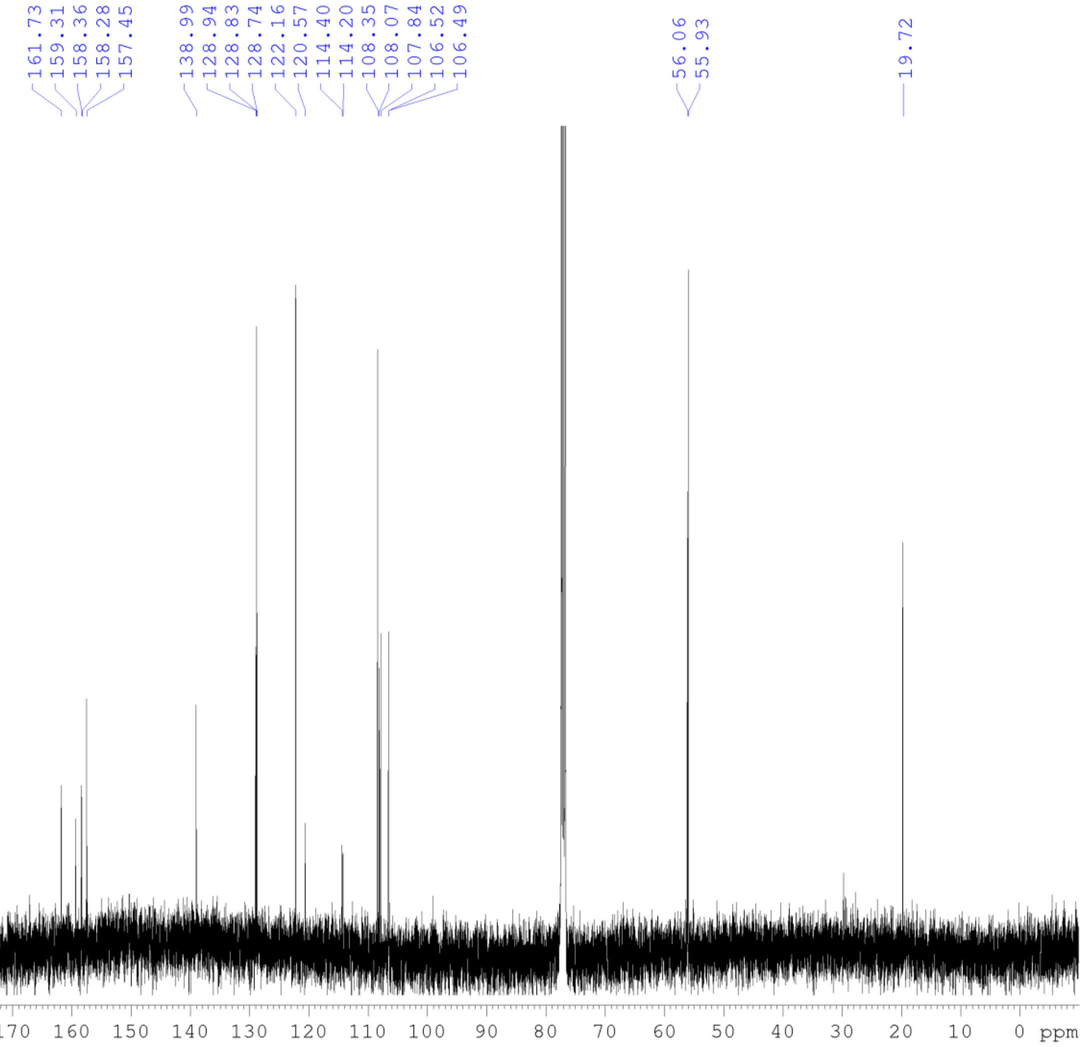

**$^{19}$F NMR** ($CDCl_3$): 2-fluoro-2',6-dimethoxy-6'-methyl-1,1'-biphenyl (**6c**)

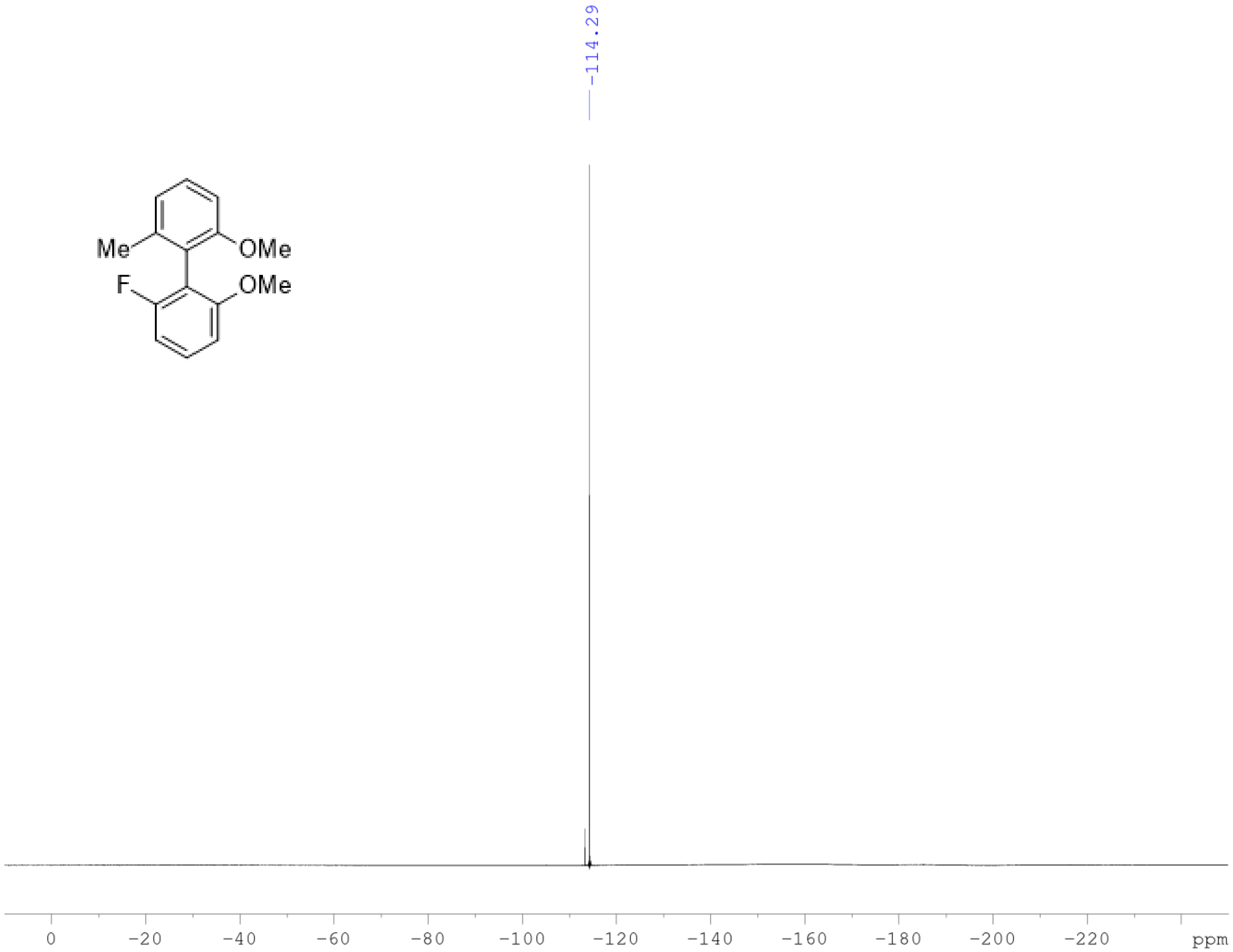

**$^{1}$H NMR** ($CDCl_3$)**:** N-(2'-fluoro-6'-hydroxy-6-methyl-[1,1'-biphenyl]-2-yl)acetamide (**7**)

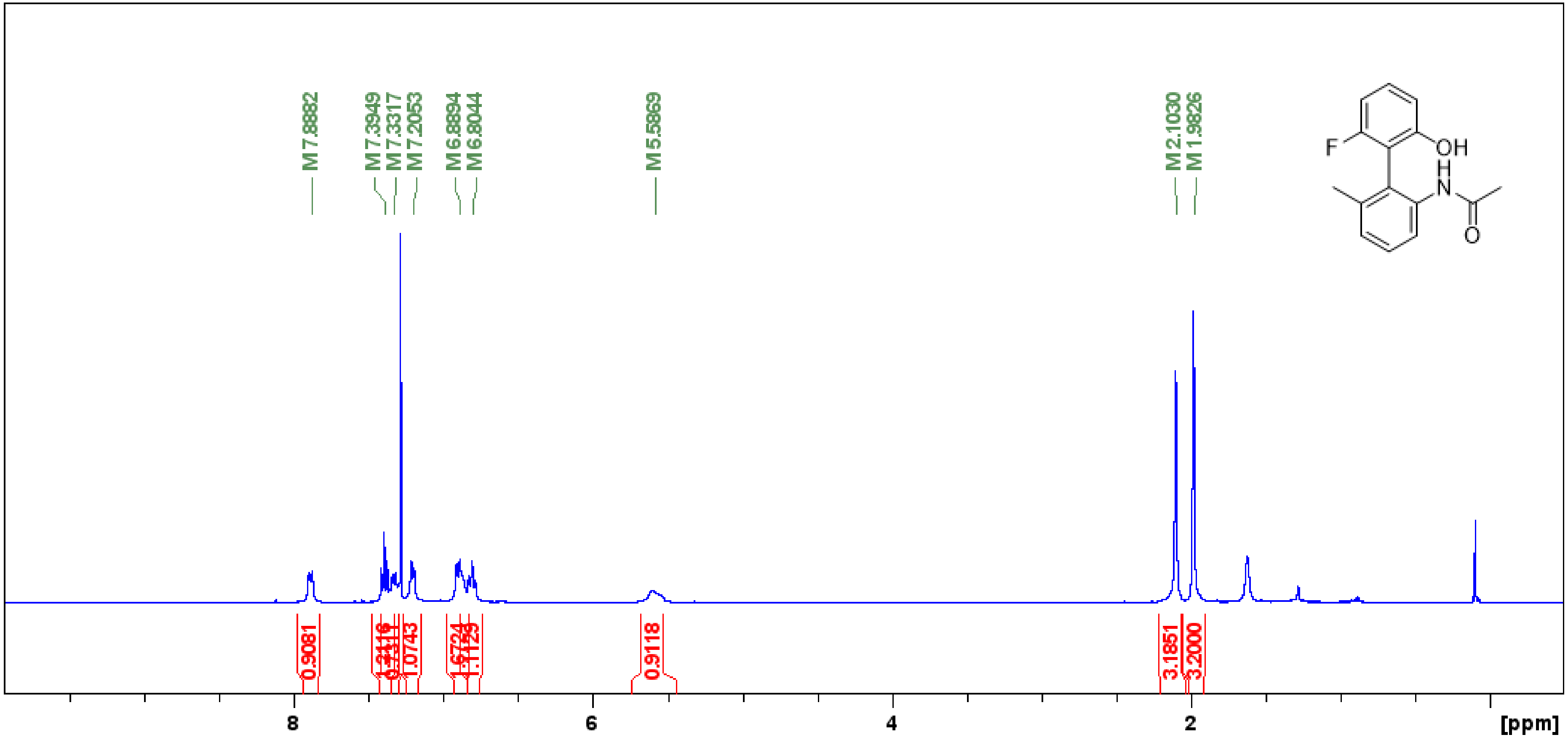

**$^{13}$C NMR** ($CDCl_3$)**:** N-(2'-fluoro-6'-hydroxy-6-methyl-[1,1'-biphenyl]-2-yl)acetamide (**7**)

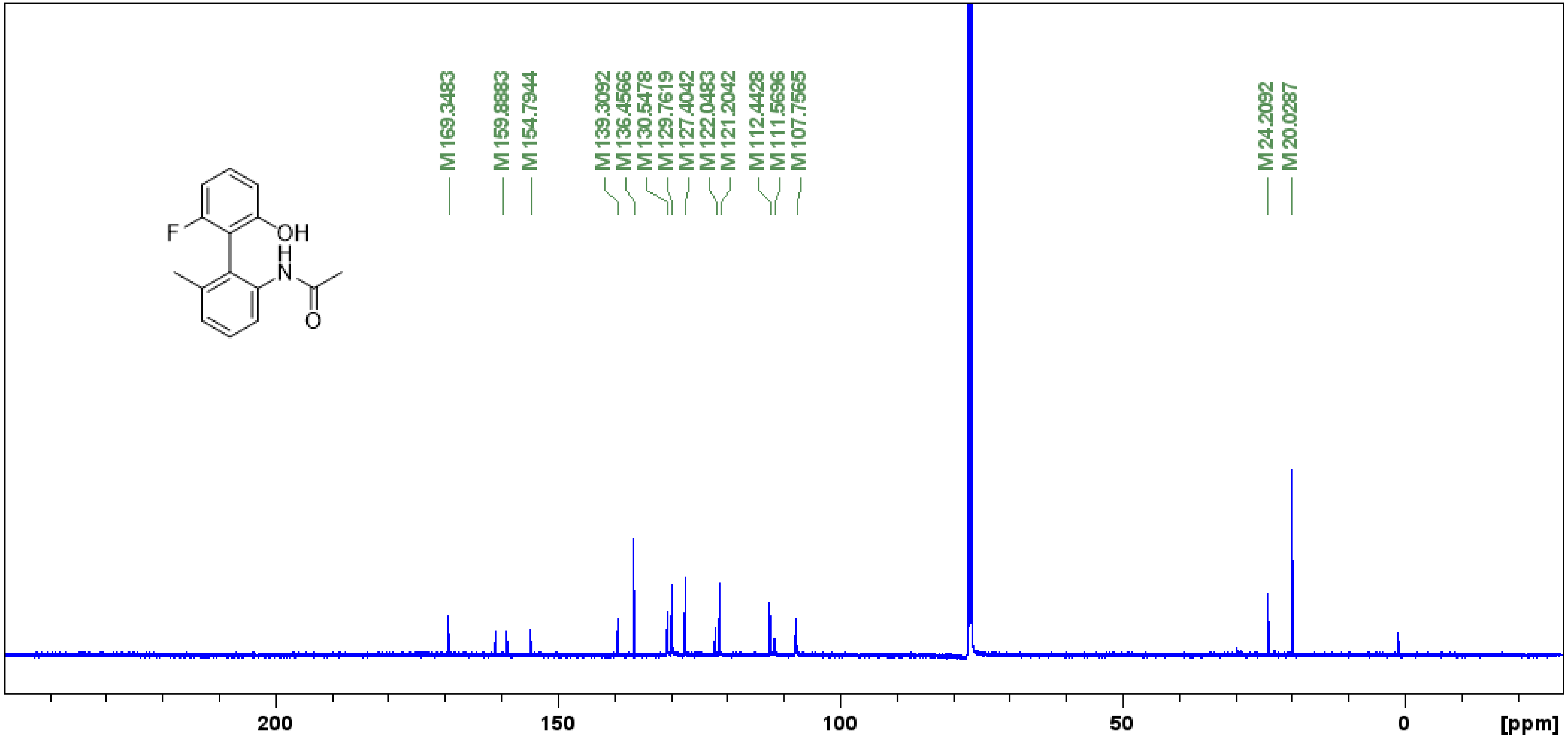

**$^{19}$F NMR** (CDCl$_3$)**:** N-(2'-fluoro-6'-hydroxy-6-methyl-[1,1'-biphenyl]-2-yl)acetamide (**7**)

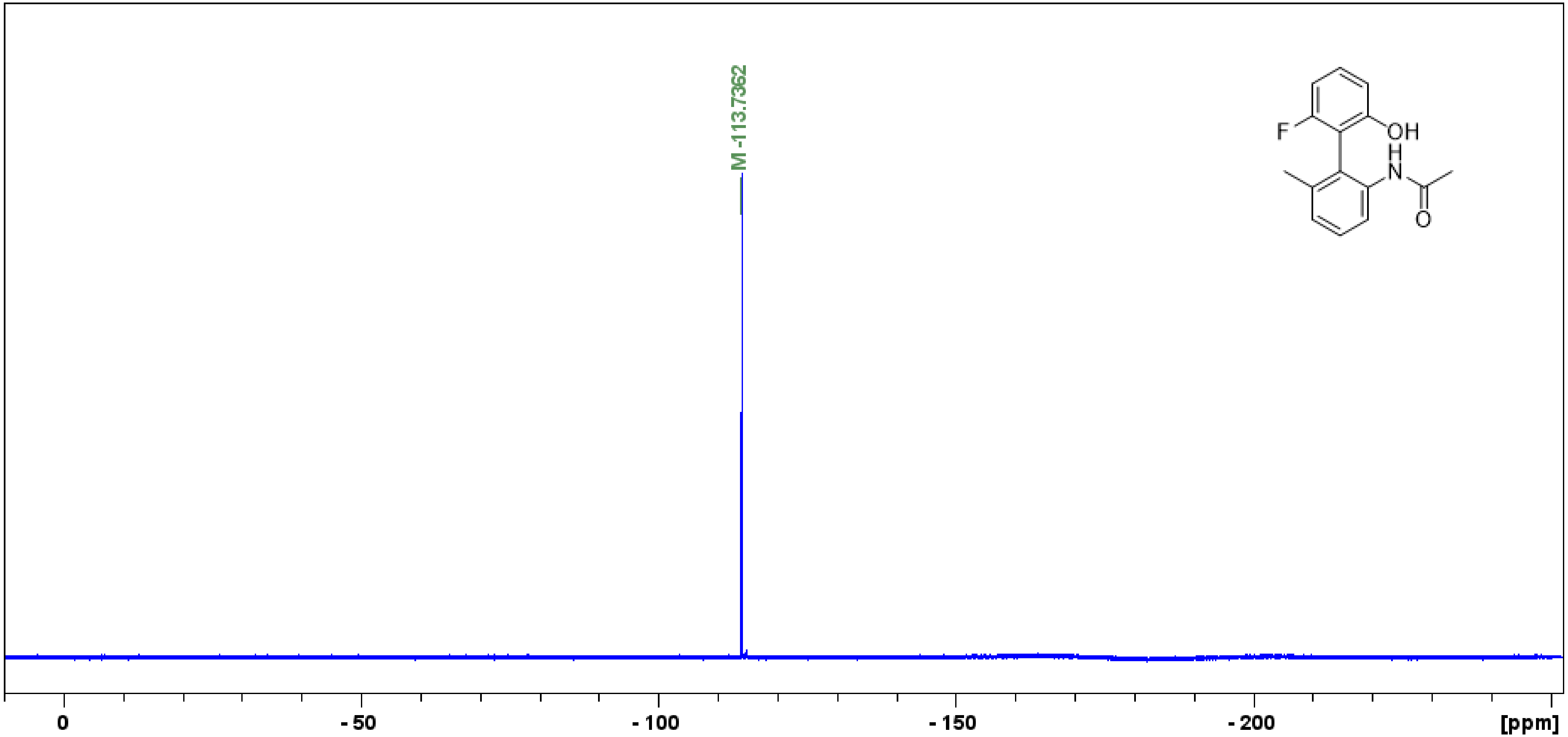

**$^{1}$H NMR** ($CD_3OD$): Bis(3-chloro-4-methylphenyl)methanaminium chloride

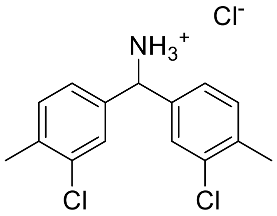


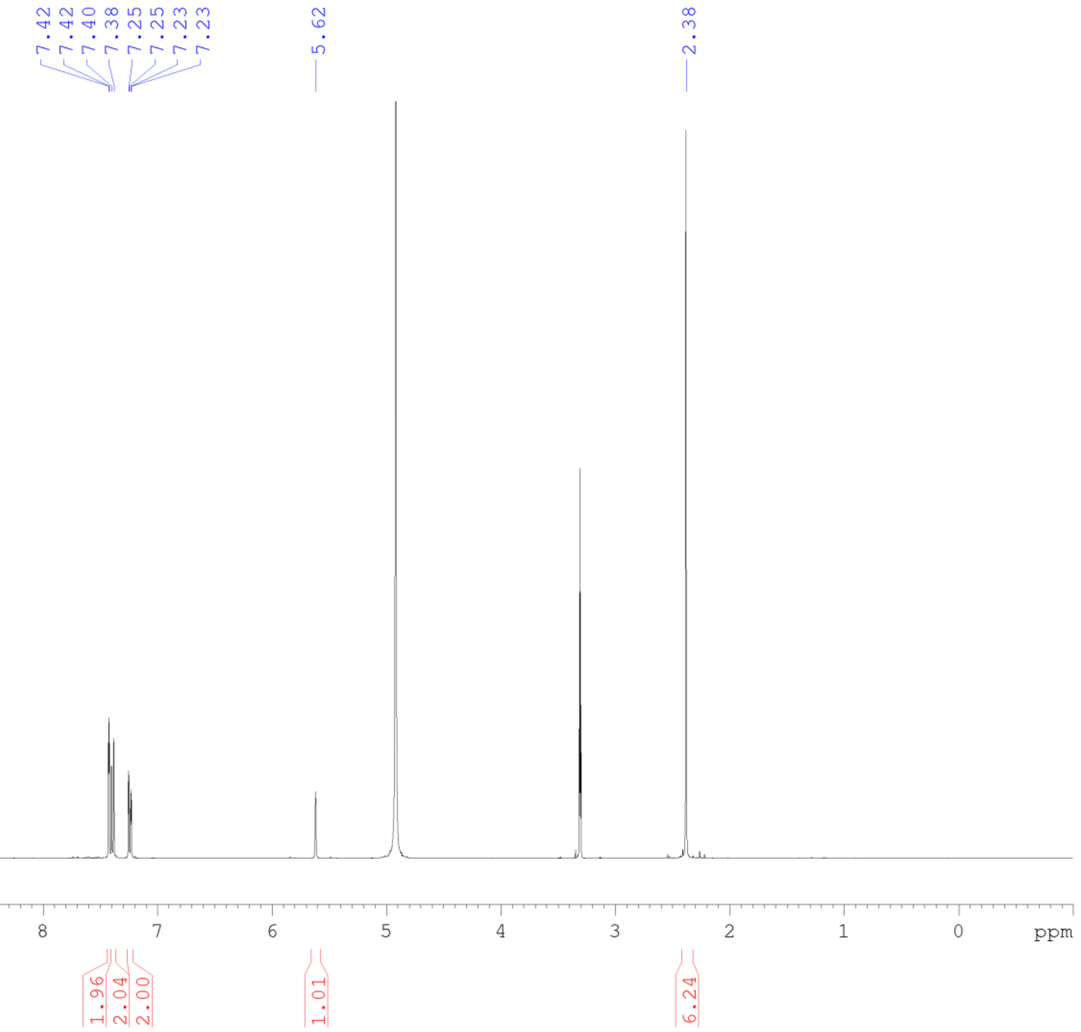

**$^{13}$C NMR** ($CD_3OD$): Bis(3-chloro-4-methylphenyl)methanaminium chloride

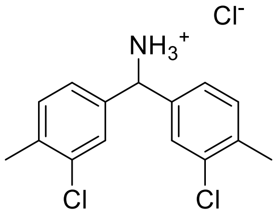


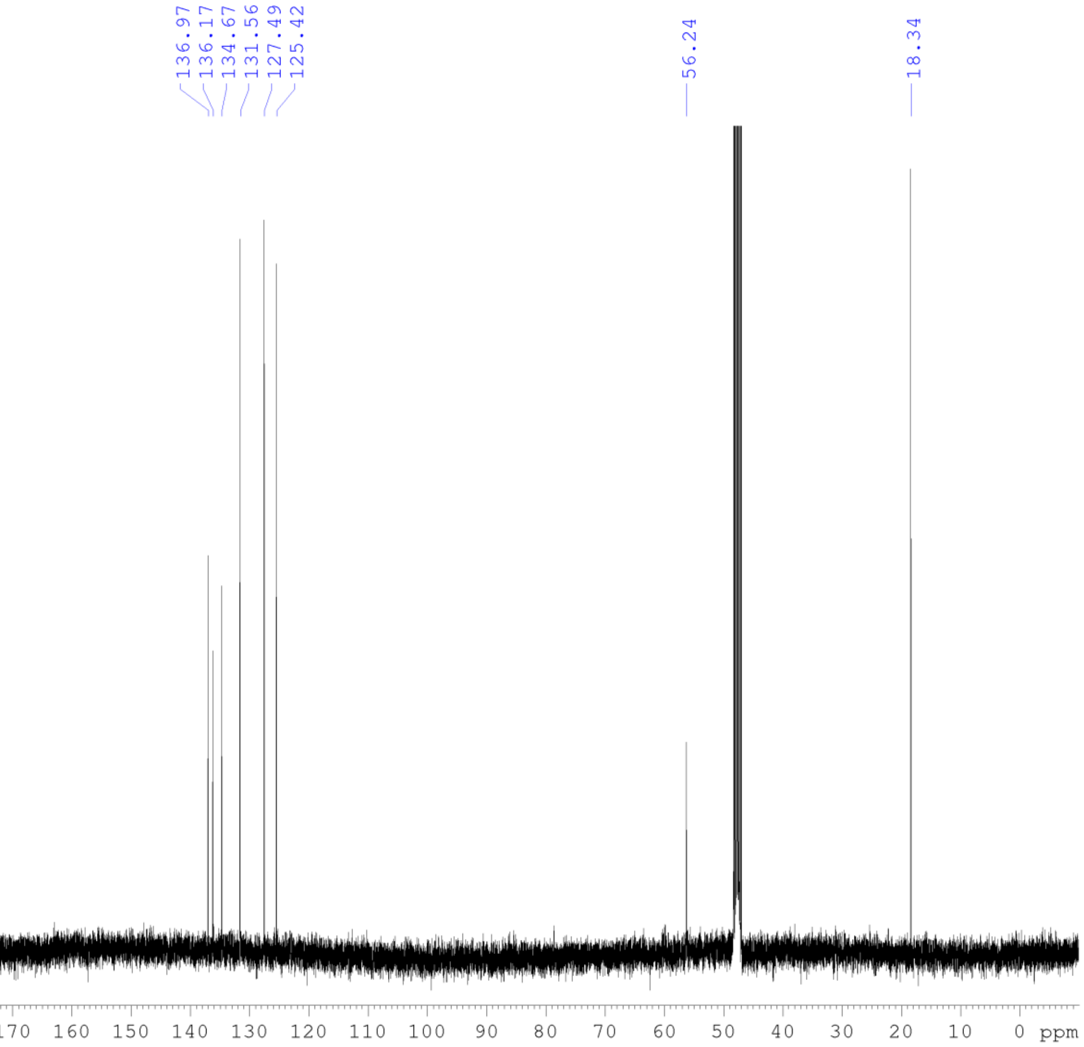

**¹H NMR** ($CDCl_3$): N-(bis(3-chloro-4-methylphenyl)methyl)-1,1,1-trifluoromethanesulfonamide (**8**)

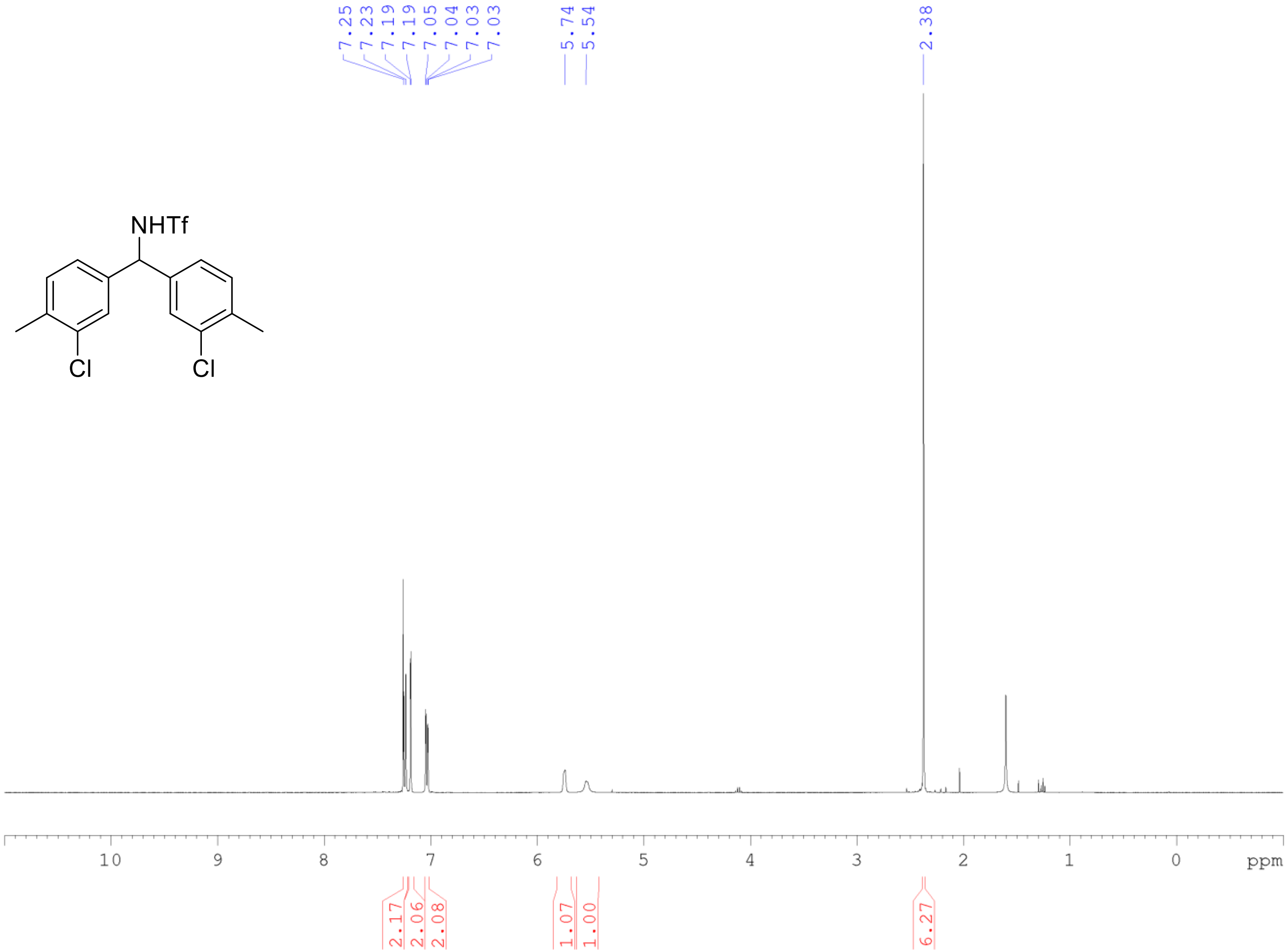

**$^{13}$C NMR** ($CDCl_3$): N-(bis(3-chloro-4-methylphenyl)methyl)-1,1,1-trifluoromethanesulfonamide (8)

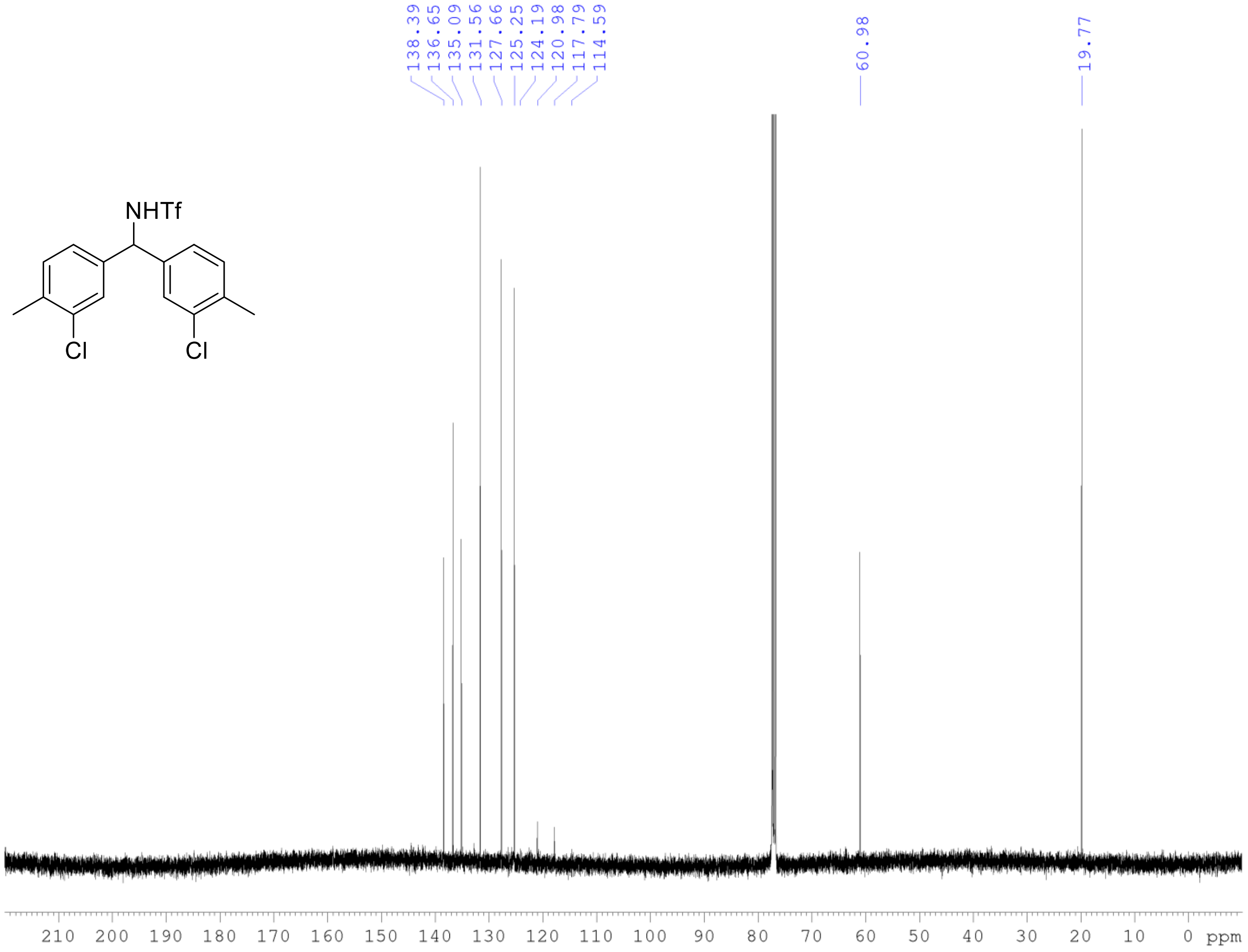

**¹⁹F NMR** ($CDCl_3$): N-(bis(3-chloro-4-methylphenyl)methyl)-1,1,1-trifluoromethanesulfonamide (**8**)

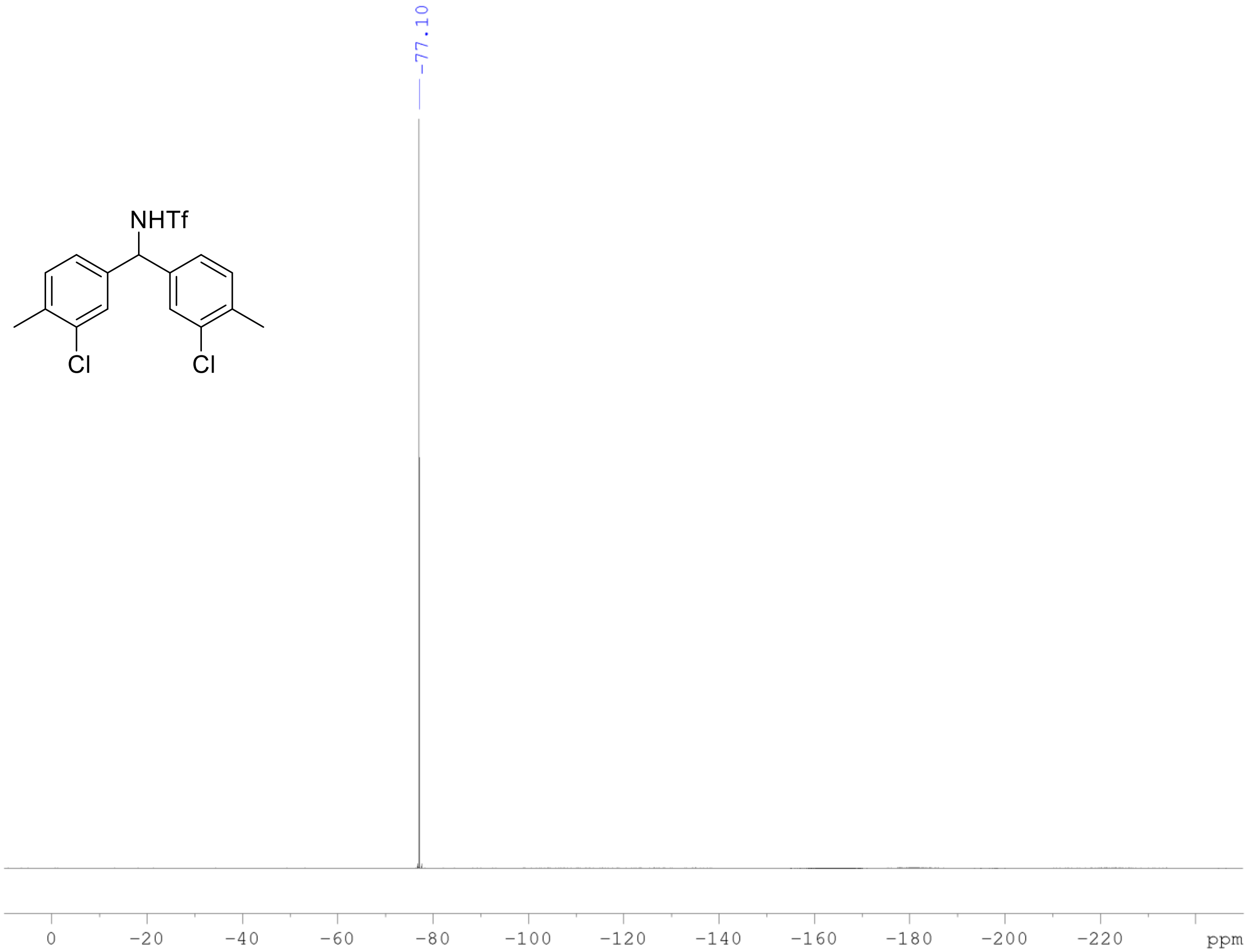

**¹H NMR** ($CDCl_3$): Ethyl 5'-((3-chloro-4-methylphenyl)((trifluoromethyl)sulfonamido)methyl)-2'-methyl-[1,1'-biphenyl]-4-carboxylate (**9**)

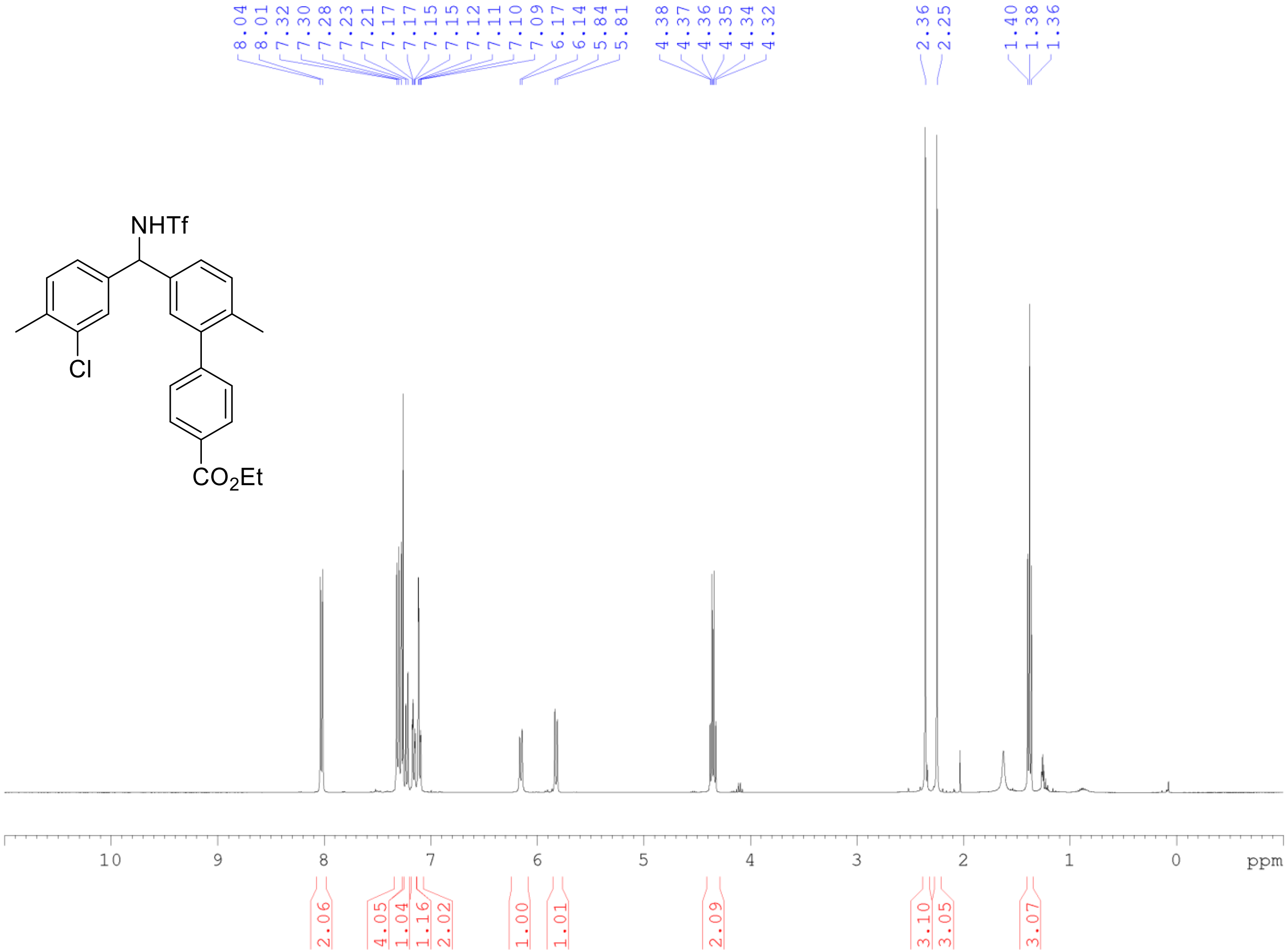

**$^{13}$C NMR** ($CDCl_3$): Ethyl 5'-((3-chloro-4-methylphenyl)((trifluoromethyl)sulfonamido)methyl)-2'-methyl-[1,1'-biphenyl]-4-carboxylate (**9**)

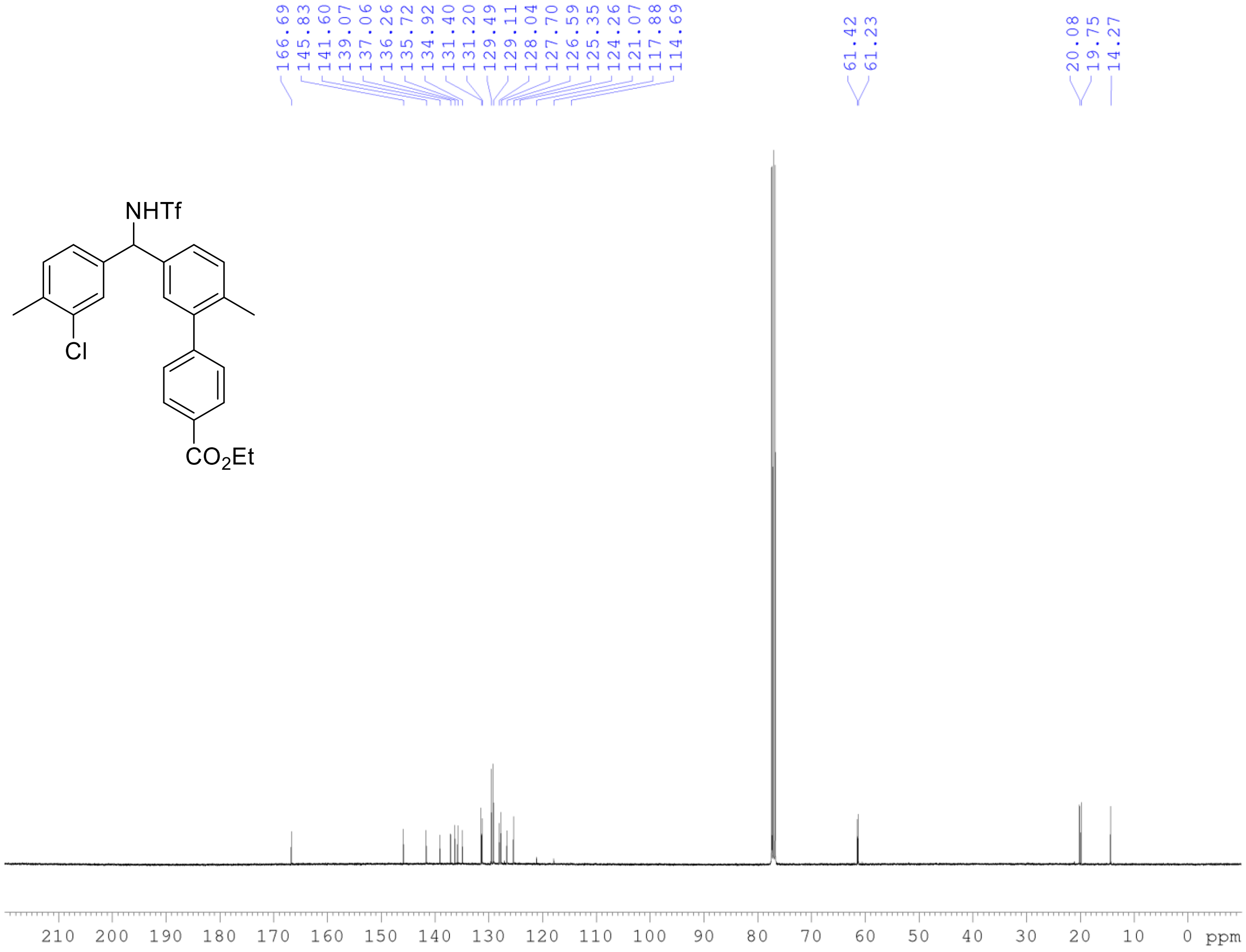

**19F NMR** ($CDCl_3$): Ethyl 5'-((3-chloro-4-methylphenyl)((trifluoromethyl)sulfonamido)methyl)-2'-methyl-[1,1'-biphenyl]-4-carboxylate (9)

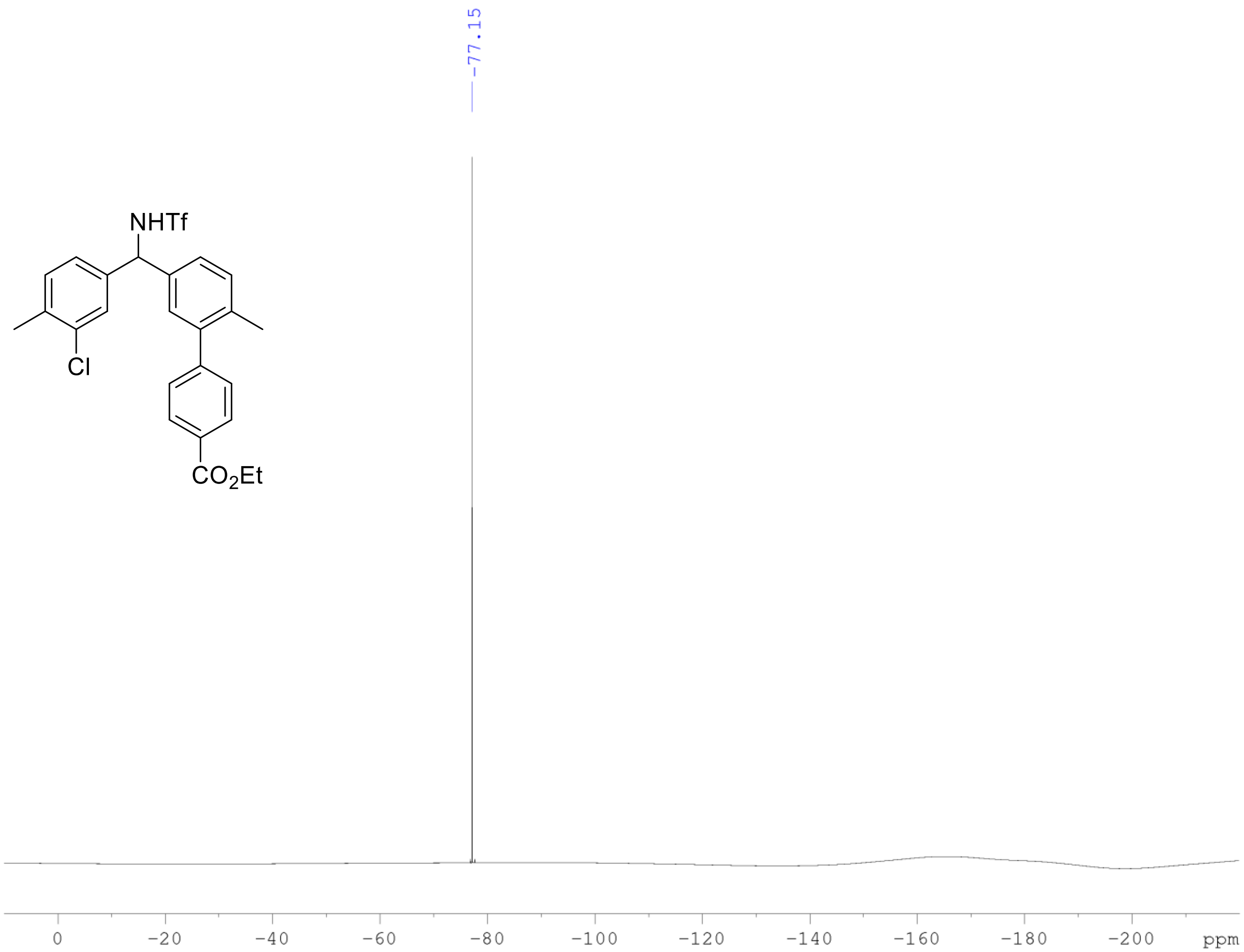

**$^1$H NMR** ($CDCl_3$) (*S*)-6-chloro-6'-fluoro-[1,1'-biphenyl]-2,2'-diol (**15**)

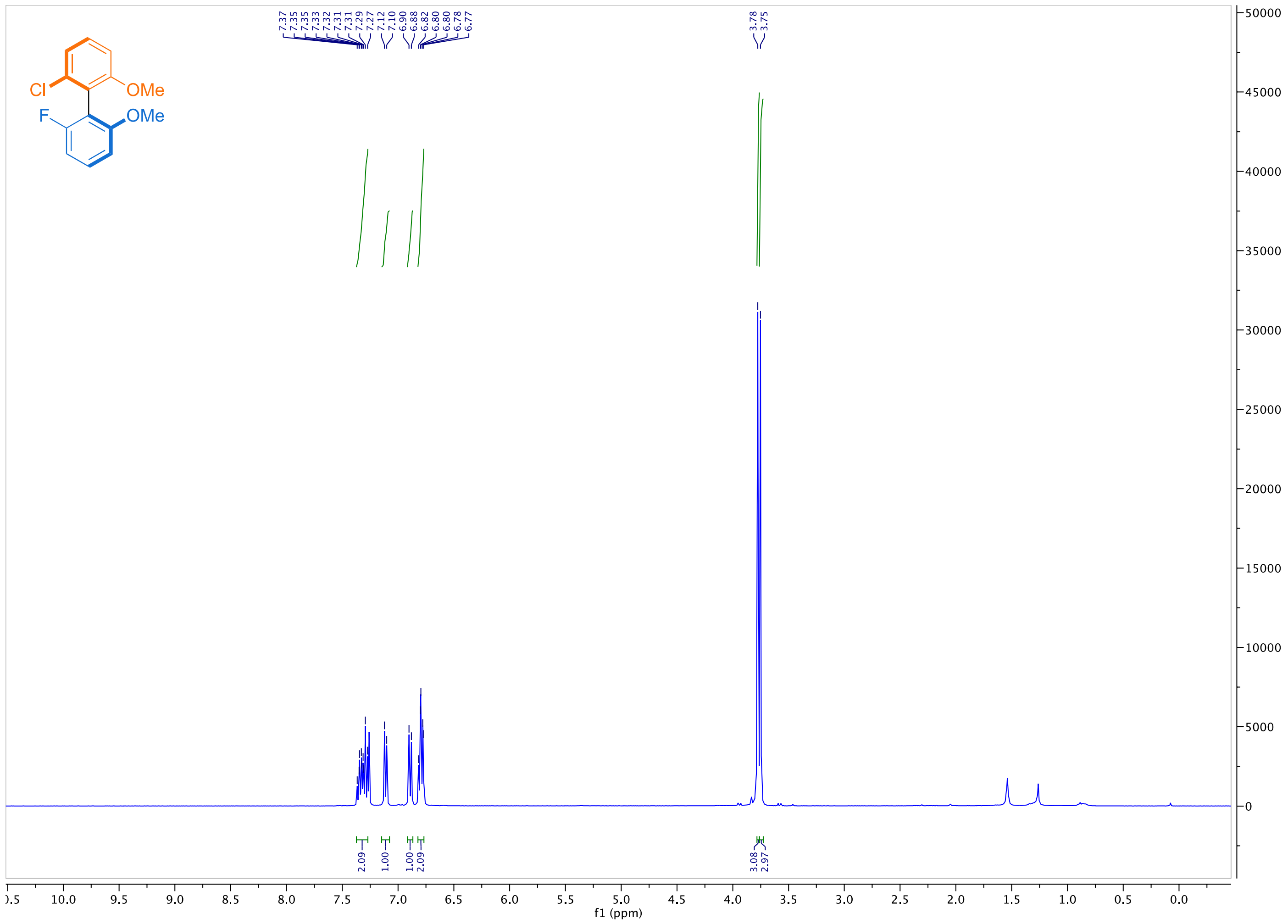

$^{13}$C NMR (CDCl3) (*S*)-6-chloro-6'-fluoro-[1,1'-biphenyl]-2,2'-diol(**15**)

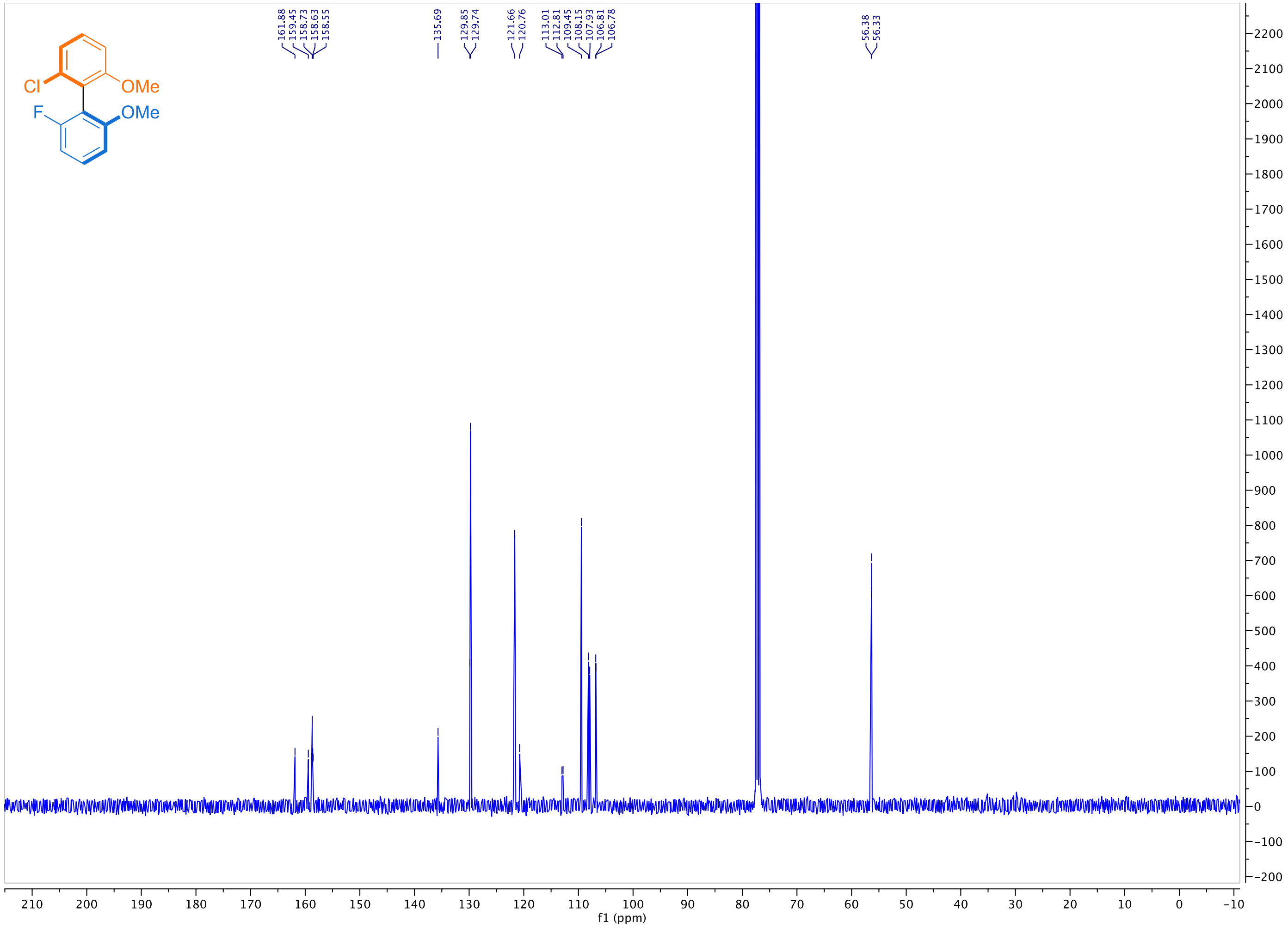

**$^{19}$F NMR** (CDCl$_3$) (*S*)-6-chloro-6'-fluoro-[1,1'-biphenyl]-2,2'-diol (**15**)

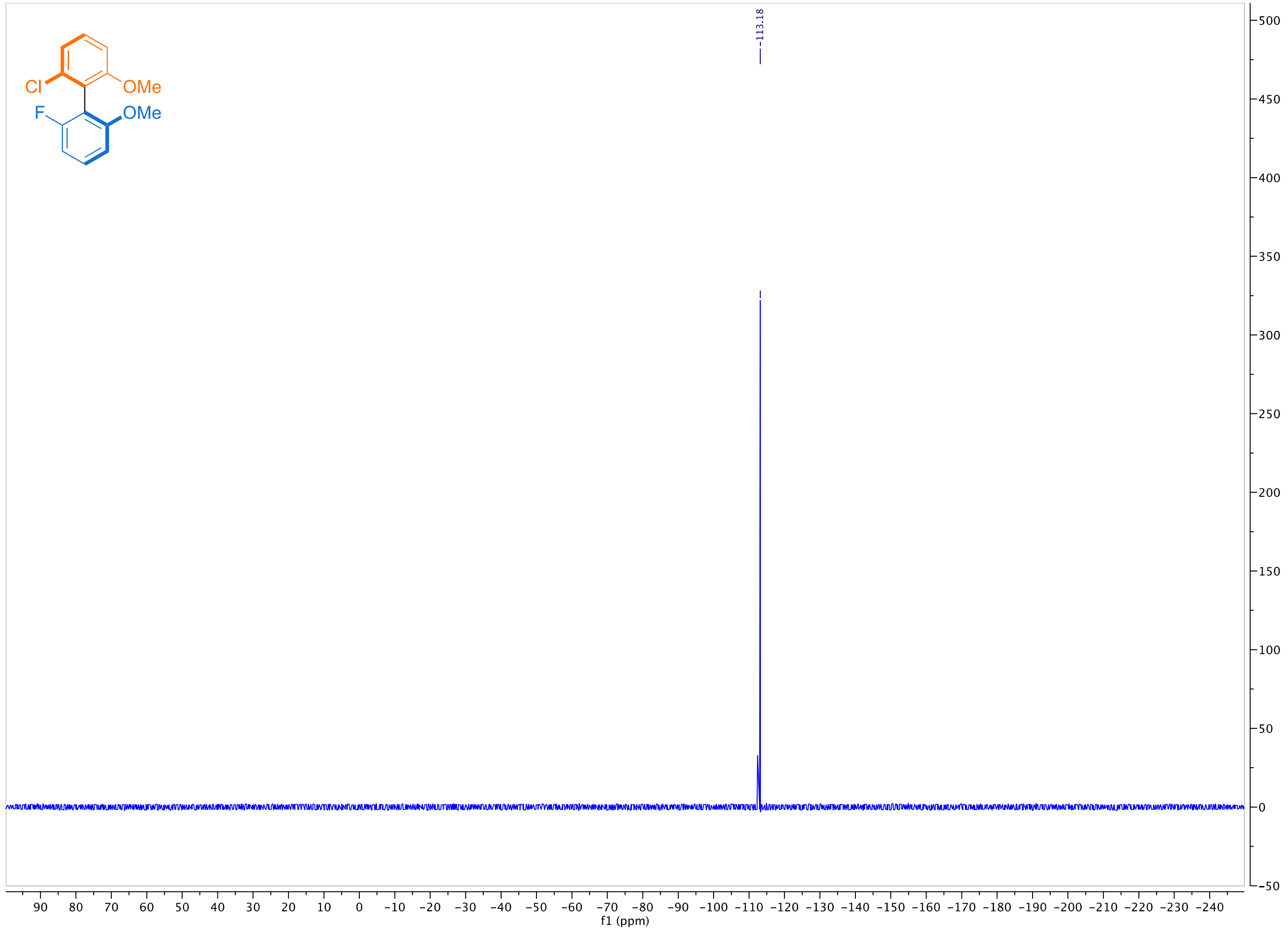

**$^1$H NMR** ($CDCl_3$) ethyl (*R*)-2''-fluoro-3',6''-dimethoxy-[1,1':2',1''-terphenyl]-4-carboxylate (**16**)

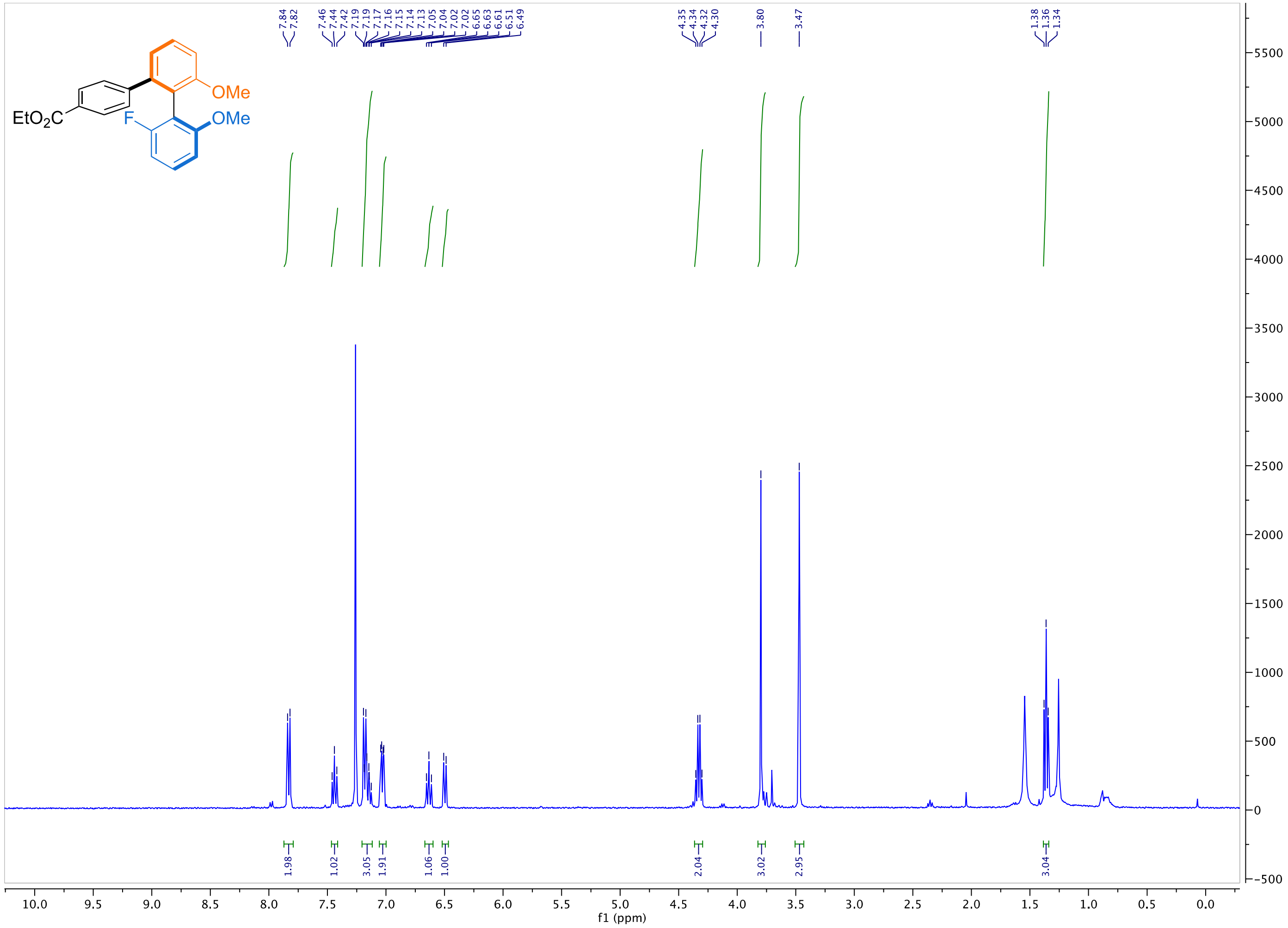

$^{13}$**C NMR** ($CDCl_3$) ethyl (*R*)-2''-fluoro-3',6''-dimethoxy-[1,1':2',1''-terphenyl]-4-carboxylate (**16**)

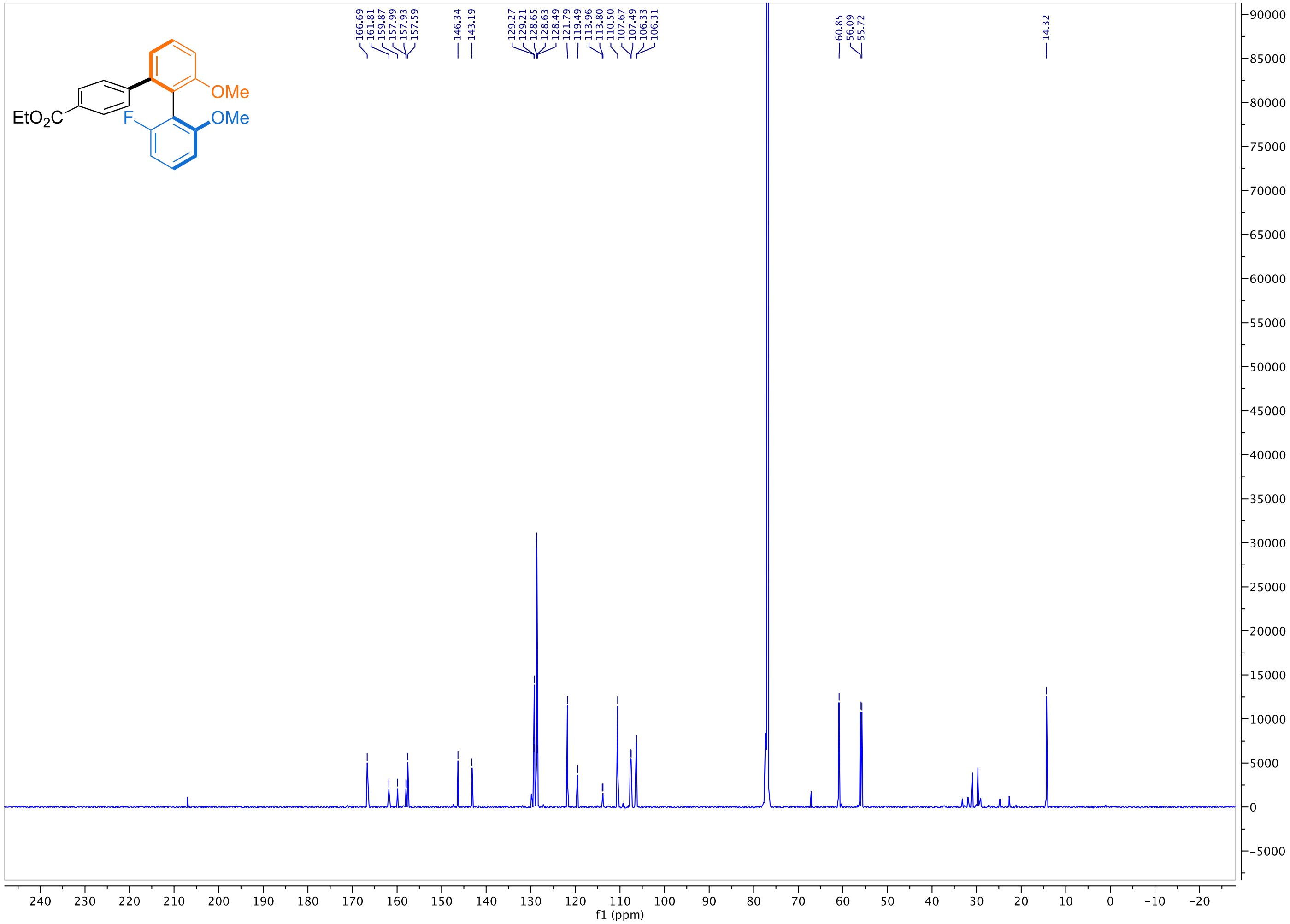

**$^{19}$F NMR** ($CDCl_3$) ethyl (*R*)-2''-fluoro-3',6''-dimethoxy-[1,1':2',1''-terphenyl]-4-carboxylate (**16**)

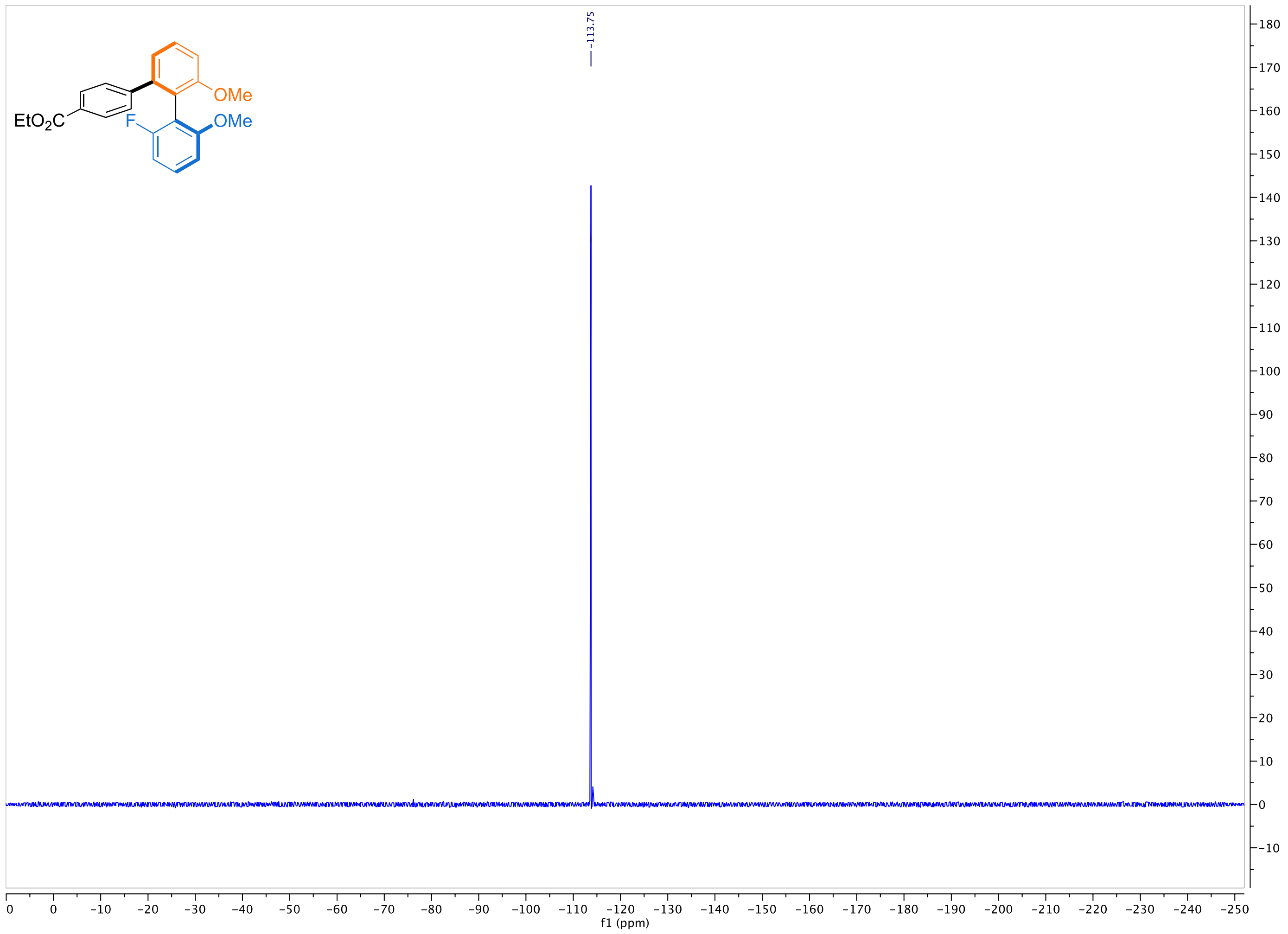

# NMR Spectra for Ligand Synthesis

**$^1$H NMR** ($CD_3OD$): Sodium 2'-(dicyclohexylphosphaneyl)-2,6-dimethoxy-[1,1'-biphenyl]-3-sulfonate (sSPhos)

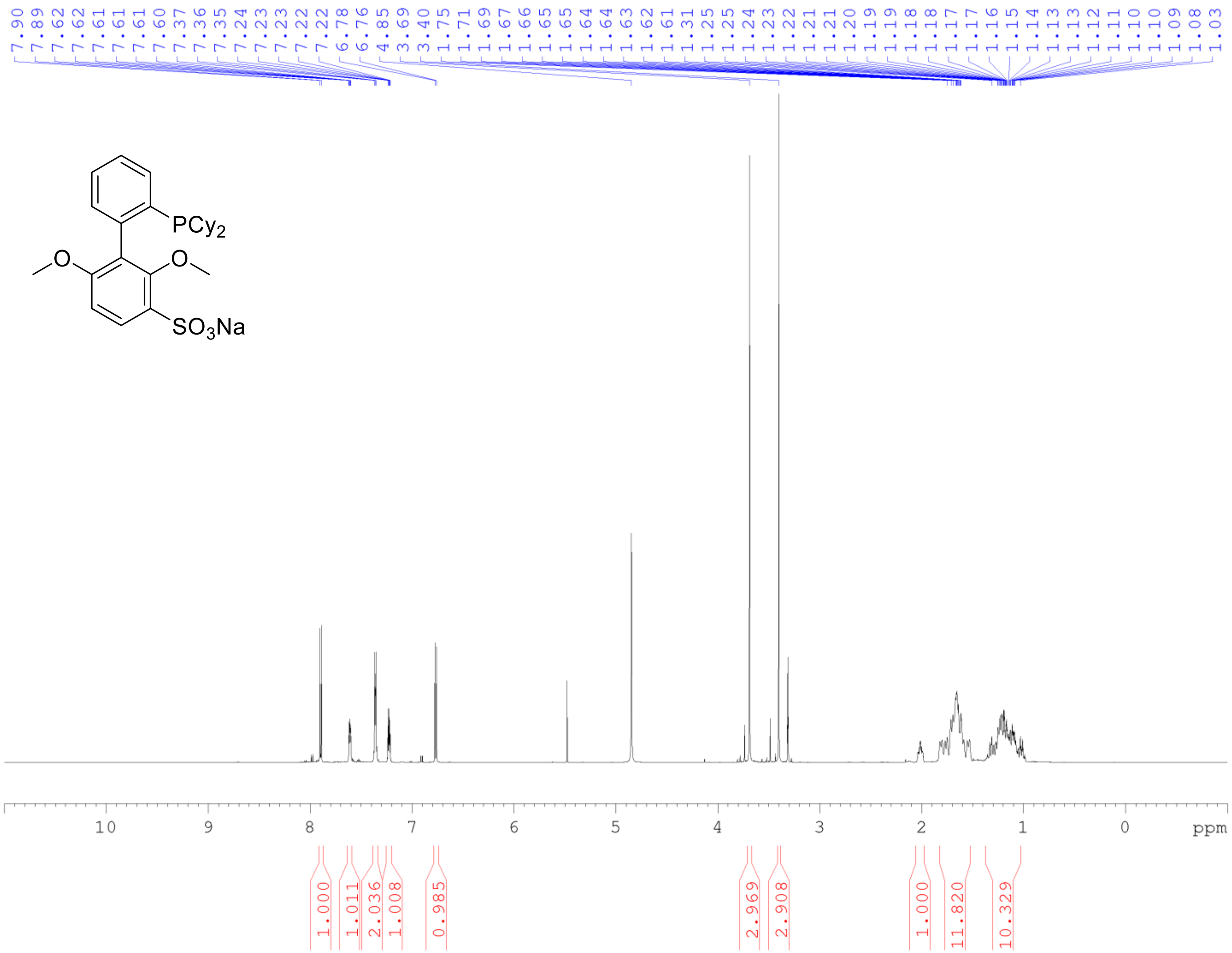

**$^{13}$C NMR** ($CD_3OD$): Sodium 2'-(dicyclohexylphosphaneyl)-2,6-dimethoxy-[1,1'-biphenyl]-3-sulfonate (sSPhos)

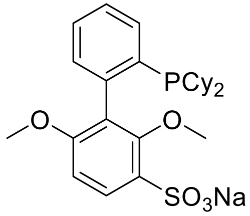


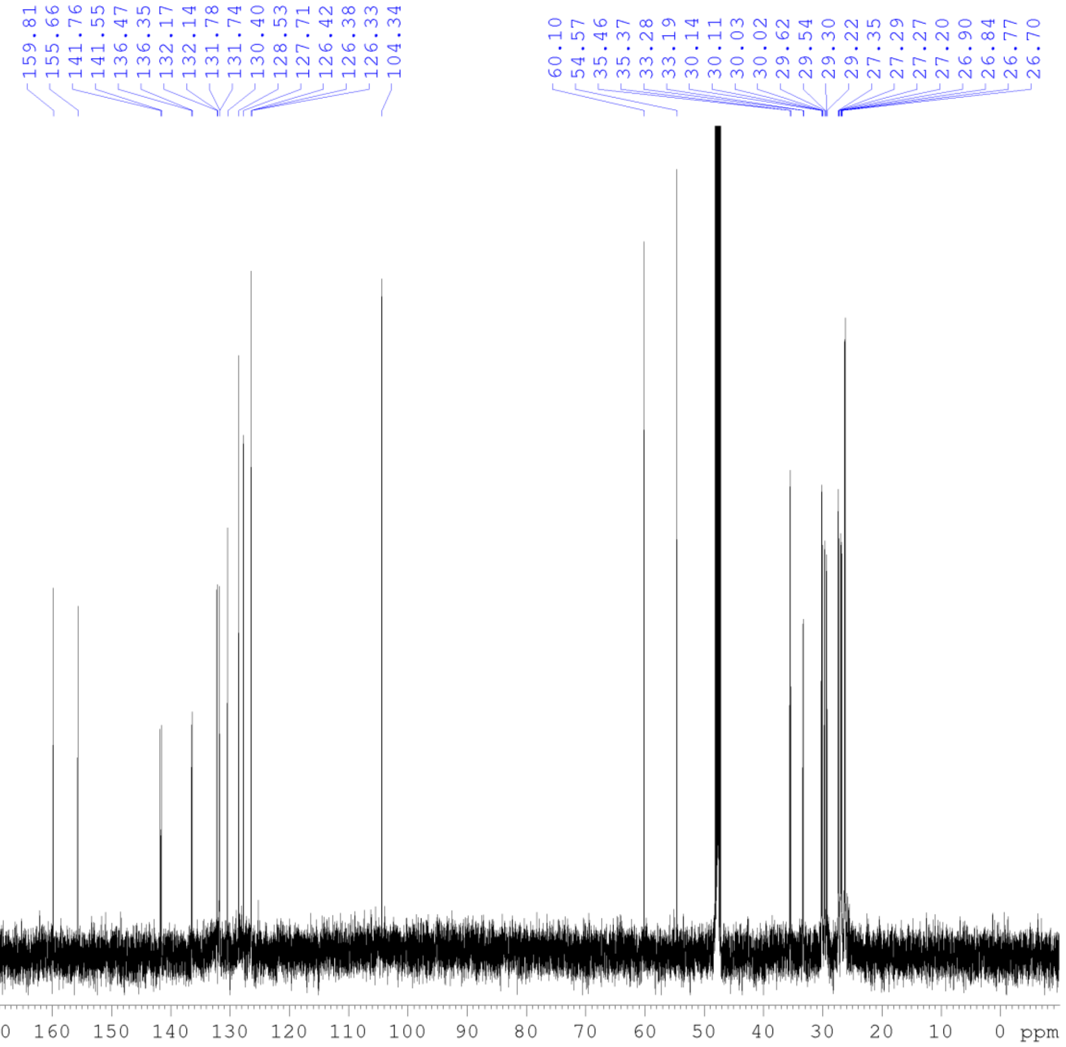

**$^{31}$P NMR** ($CD_3OD$): Sodium 2'-(dicyclohexylphosphaneyl)-2,6-dimethoxy-[1,1'-biphenyl]-3-sulfonate (sSPhos)

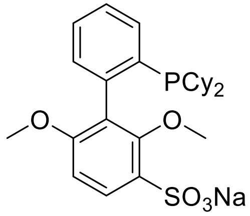


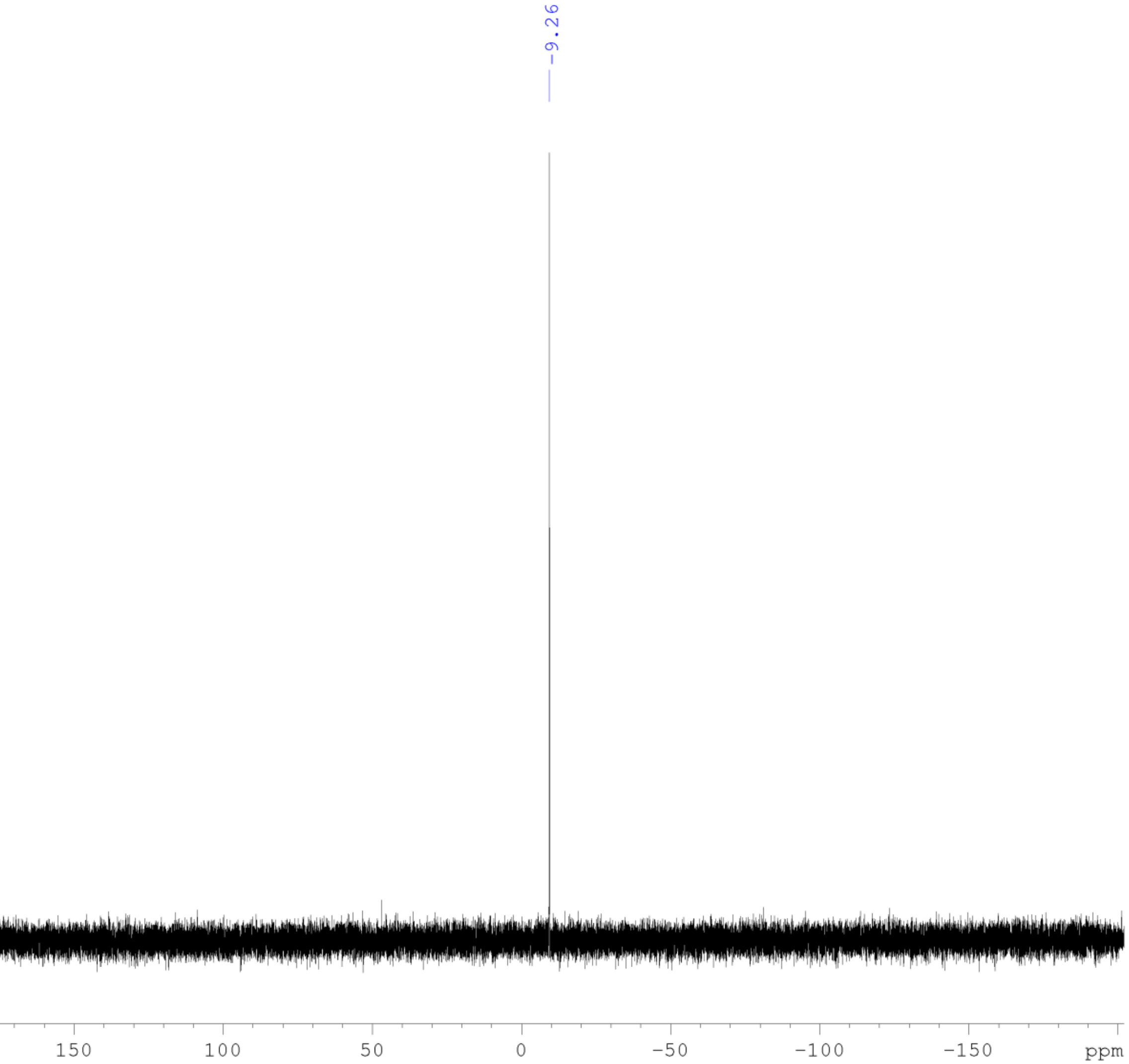

**$^1$H NMR** ($CDCl_3$): (*R*,*R*)-2'-hydroxy-[1,1'-binaphthalen]-2-yl 2'-(dicyclohexylphosphoryl)-2,6-dimethoxy-[1,1'-biphenyl]-3-sulfonate (*R*,*R*)-**10**

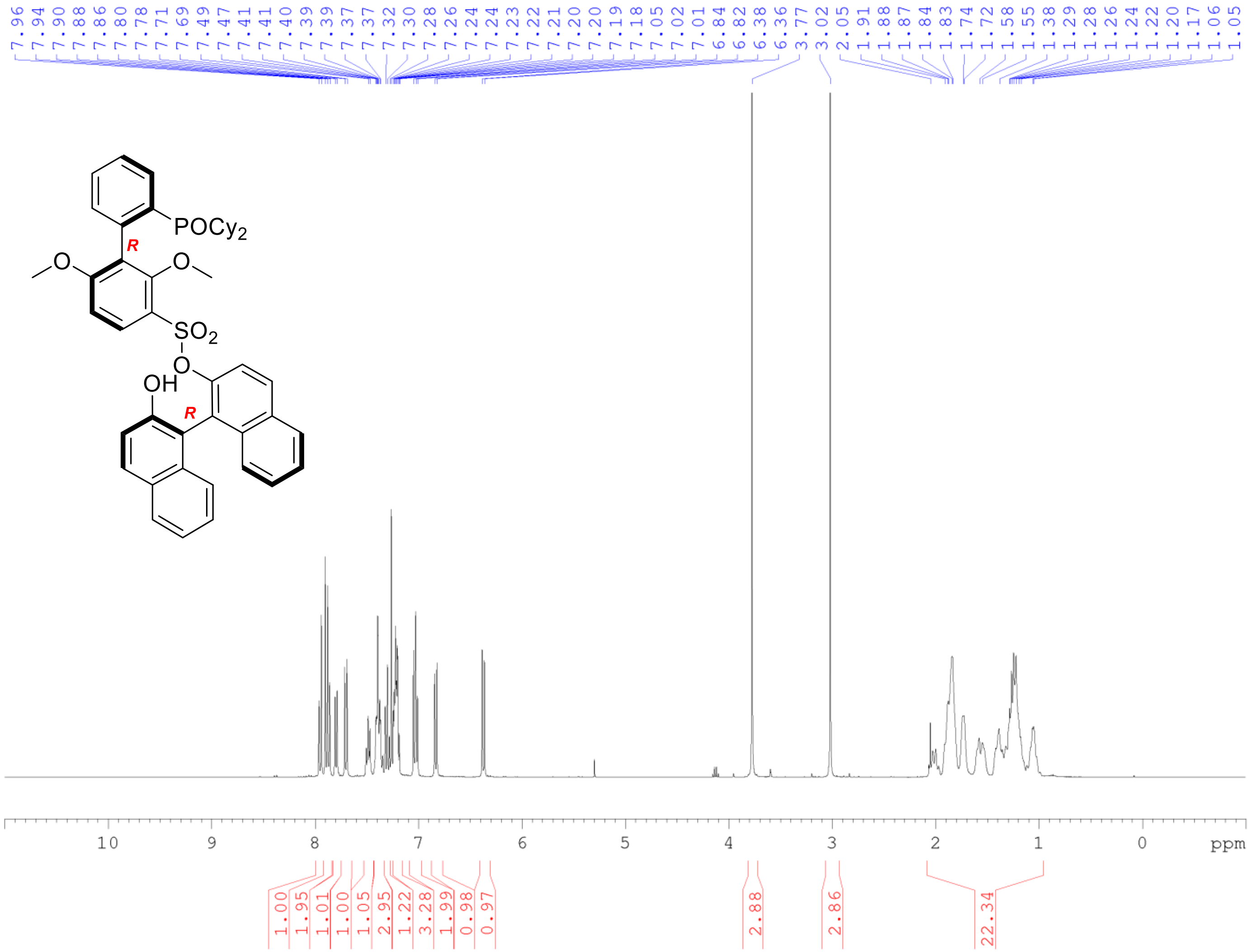

**$^{13}$C NMR** ($CDCl_3$): (*R*,*R*)-2'-hydroxy-[1,1'-binaphthalen]-2-yl 2'-(dicyclohexylphosphoryl)-2,6-dimethoxy-[1,1'-biphenyl]-3-sulfonate (*R,R*)-**10**

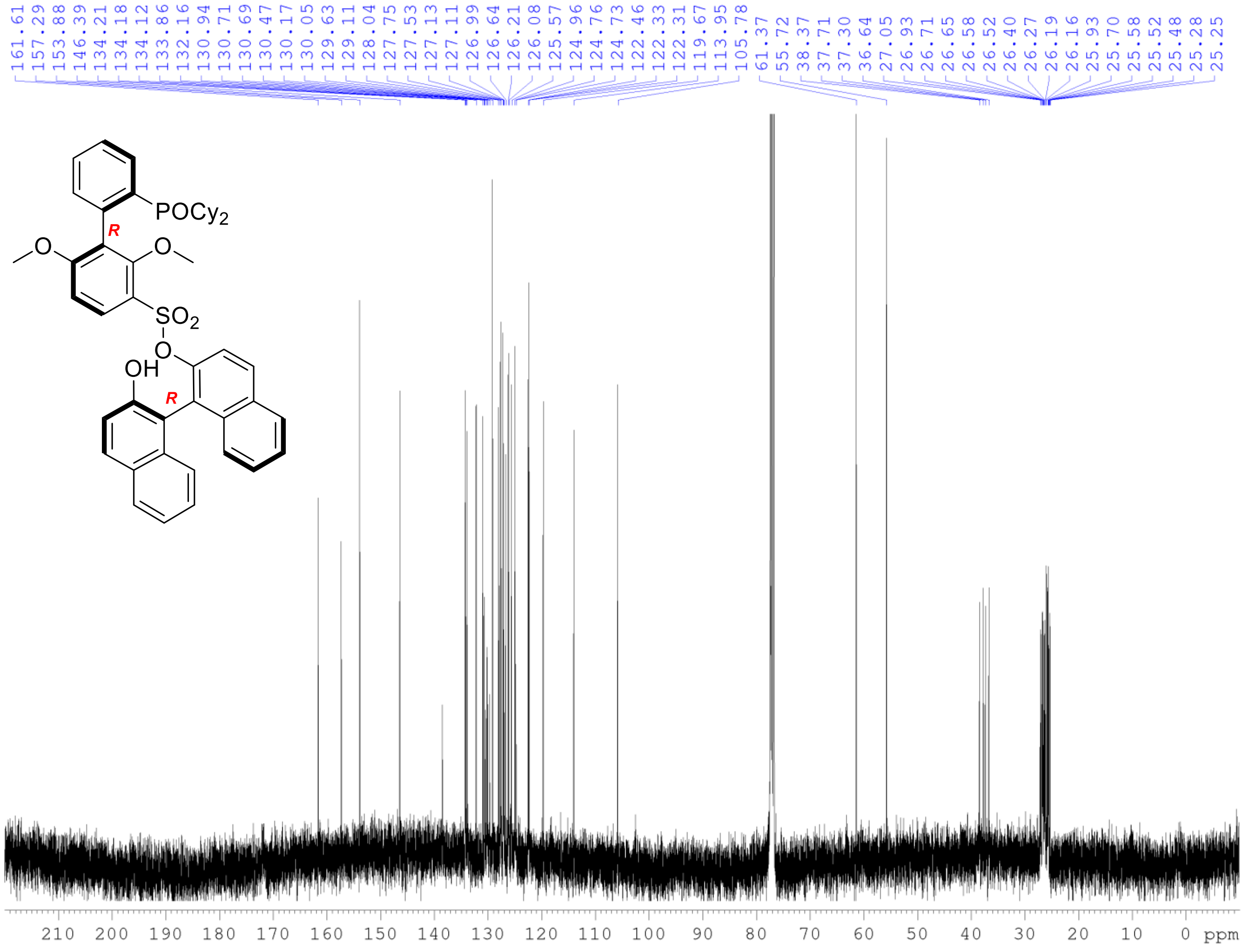

**$^{31}$P NMR** ($CDCl_3$): (*R*,*R*)-2'-hydroxy-[1,1'-binaphthalen]-2-yl 2'-(dicyclohexylphosphoryl)-2,6-dimethoxy-[1,1'-biphenyl]-3-sulfonate (*R,R*)-**10**

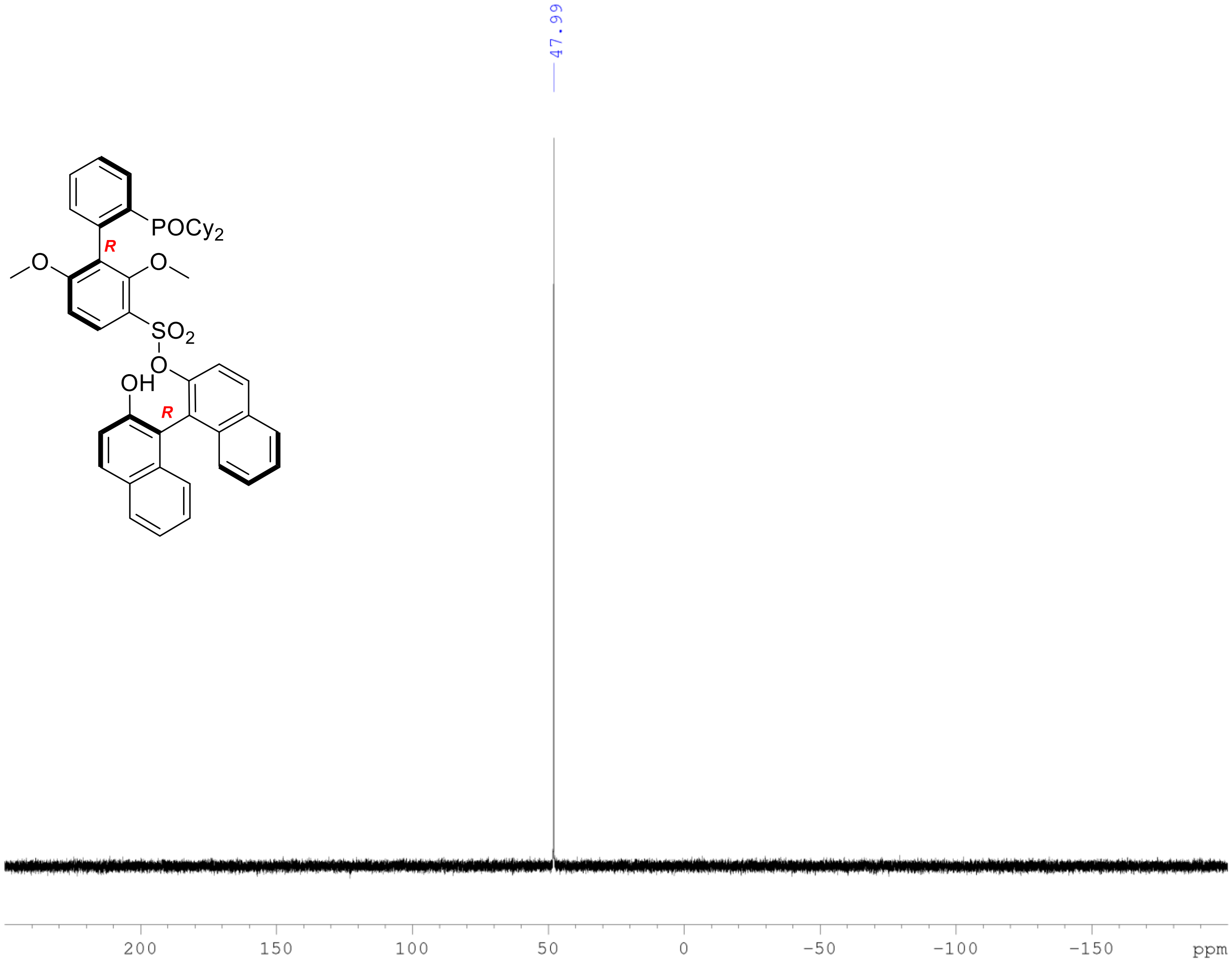

**$^1$H NMR** ($CDCl_3$): (R,S)-2'-hydroxy-[1,1'-binaphthalen]-2-yl 2'-(dicyclohexylphosphoryl)-2,6-dimethoxy-[1,1'-biphenyl]-3-sulfonate (*R*,*S*)-**10**

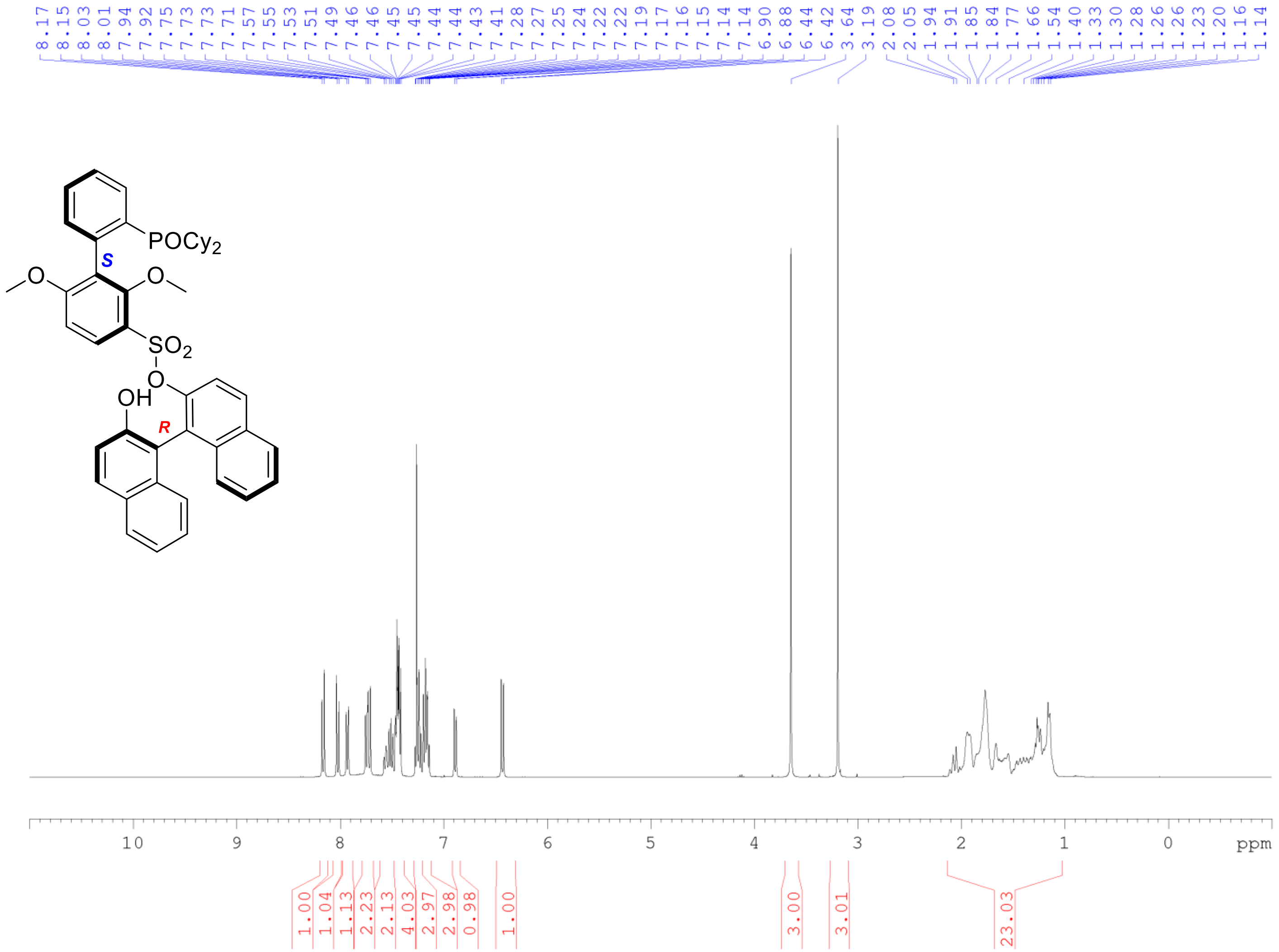

**$^{13}$C NMR** ($CDCl_3$): (*S*,*R*)-2'-hydroxy-[1,1'-binaphthalen]-2-yl 2'-(dicyclohexylphosphoryl)-2,6-dimethoxy-[1,1'-biphenyl]-3-sulfonate (*R,S*)-**10**

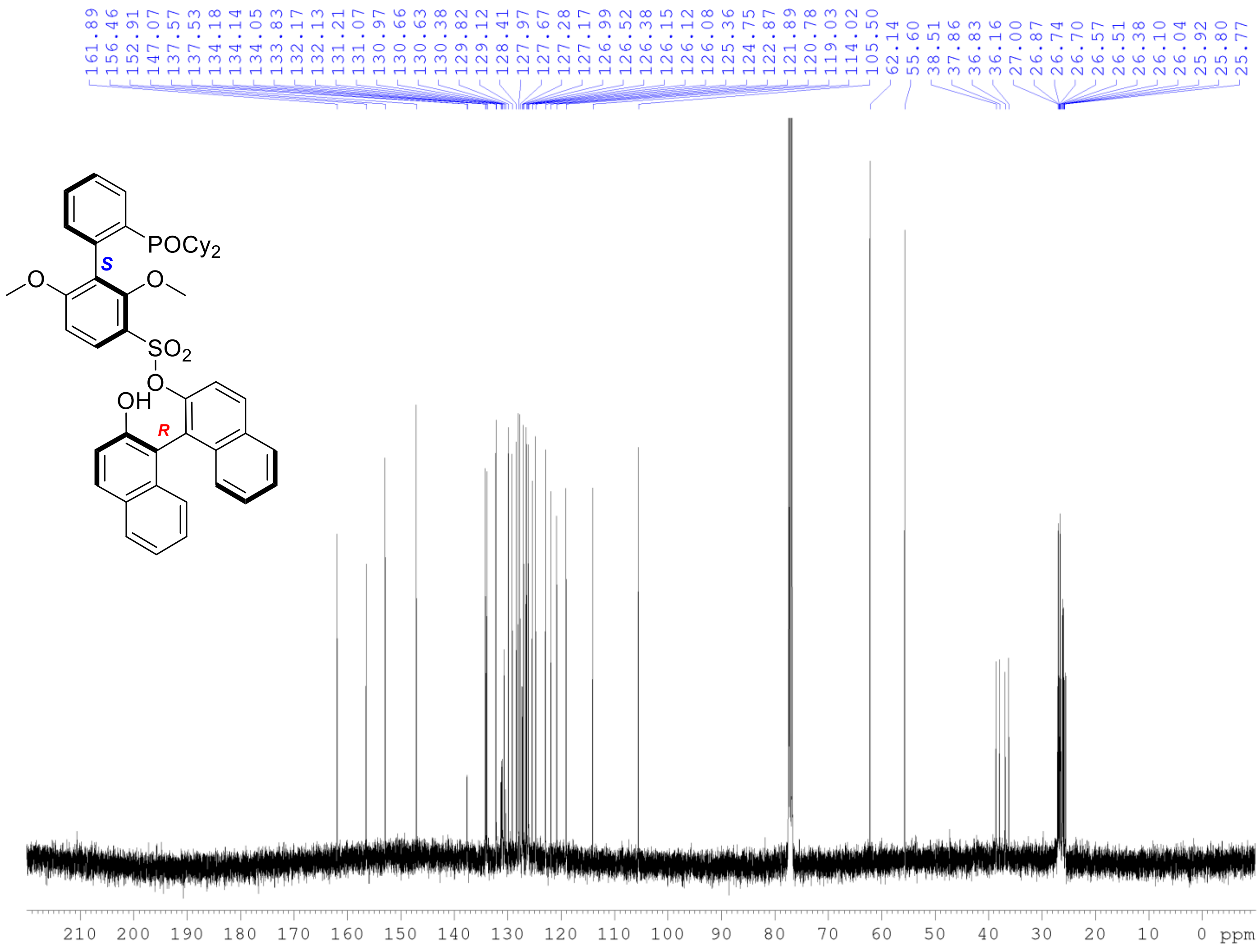

**$^{31}$P NMR** ($CDCl_3$): (*S*,*R*)-2'-hydroxy-[1,1'-binaphthalen]-2-yl 2'-(dicyclohexylphosphoryl)-2,6-dimethoxy-[1,1'-biphenyl]-3-sulfonate (*R,S*)-**10**

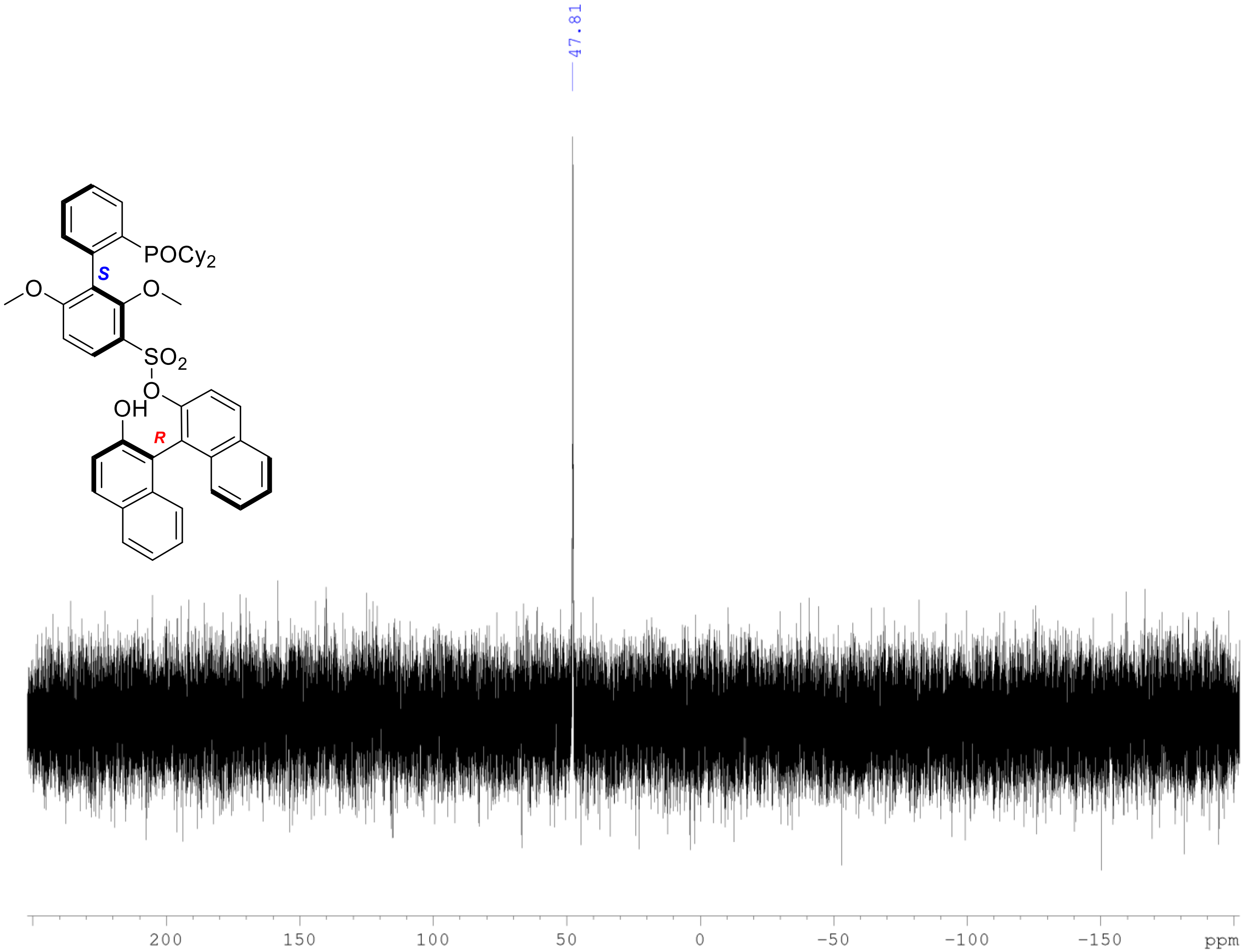

**$^1$H NMR** ($CD_3OD$): Sodium (S)-2'-(dicyclohexylphosphoryl)-2,6-dimethoxy-[1,1'-biphenyl]-3-sulfonate (*S*)-**11**

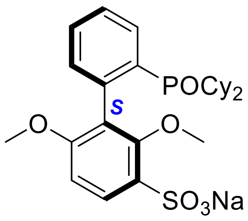


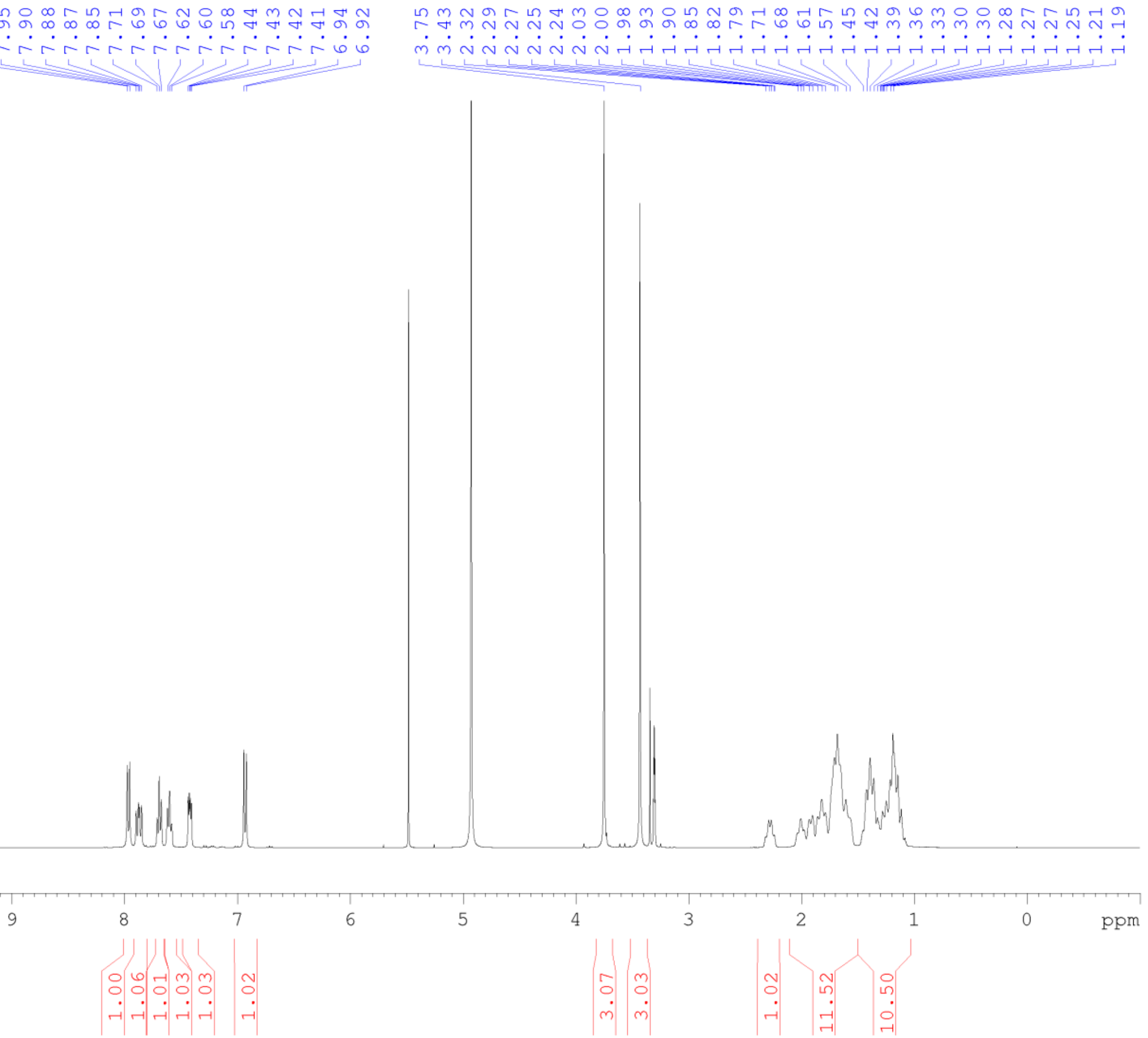

**$^{13}$C NMR** ($CD_3OD$): Sodium (S)-2'-(dicyclohexylphosphoryl)-2,6-dimethoxy-[1,1'-biphenyl]-3-sulfonate(*S*)-**11**

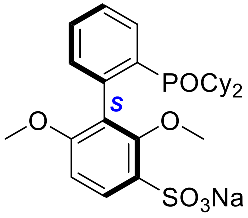


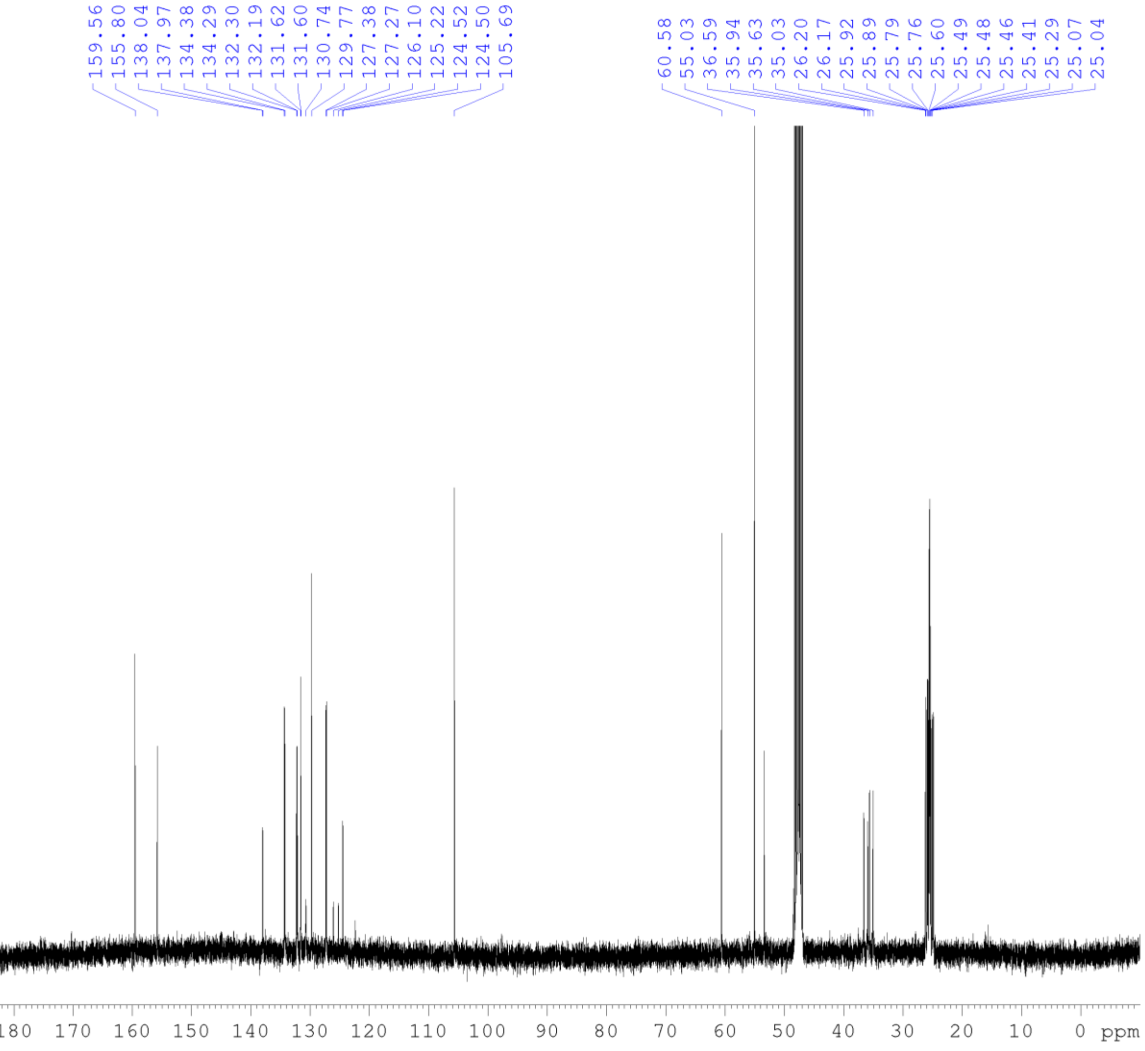

**$^{31}$P NMR** ($CD_3OD$): Sodium (S)-2'-(dicyclohexylphosphoryl)-2,6-dimethoxy-[1,1'-biphenyl]-3-sulfonate (*S*)-**11**

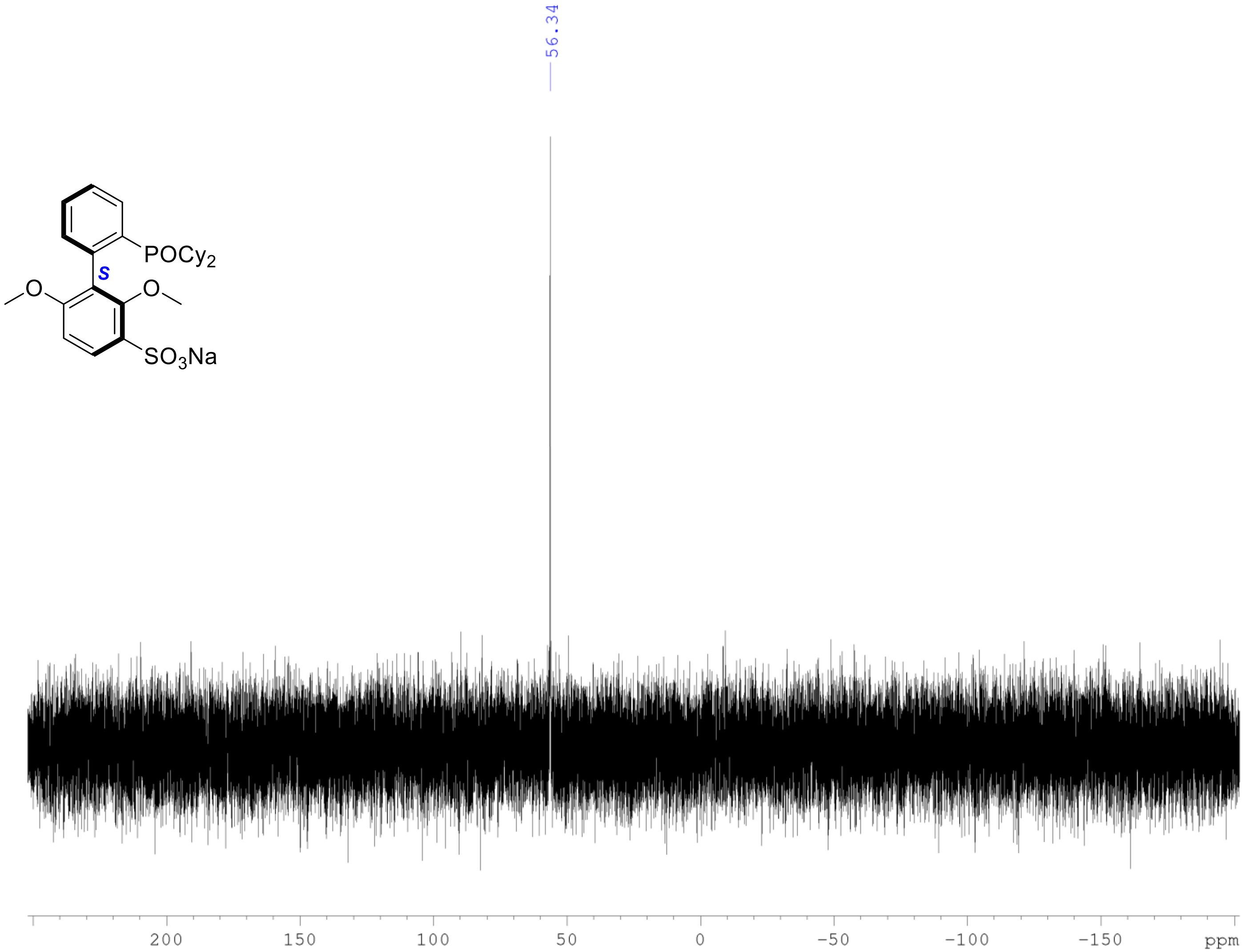

**$^1$H NMR** ($CDCl_3$): Isobutyl (S)-2'-(dicyclohexylphosphoryl)-2,6-dimethoxy-[1,1'-biphenyl]-3-sulfonate (*S*)-**12**

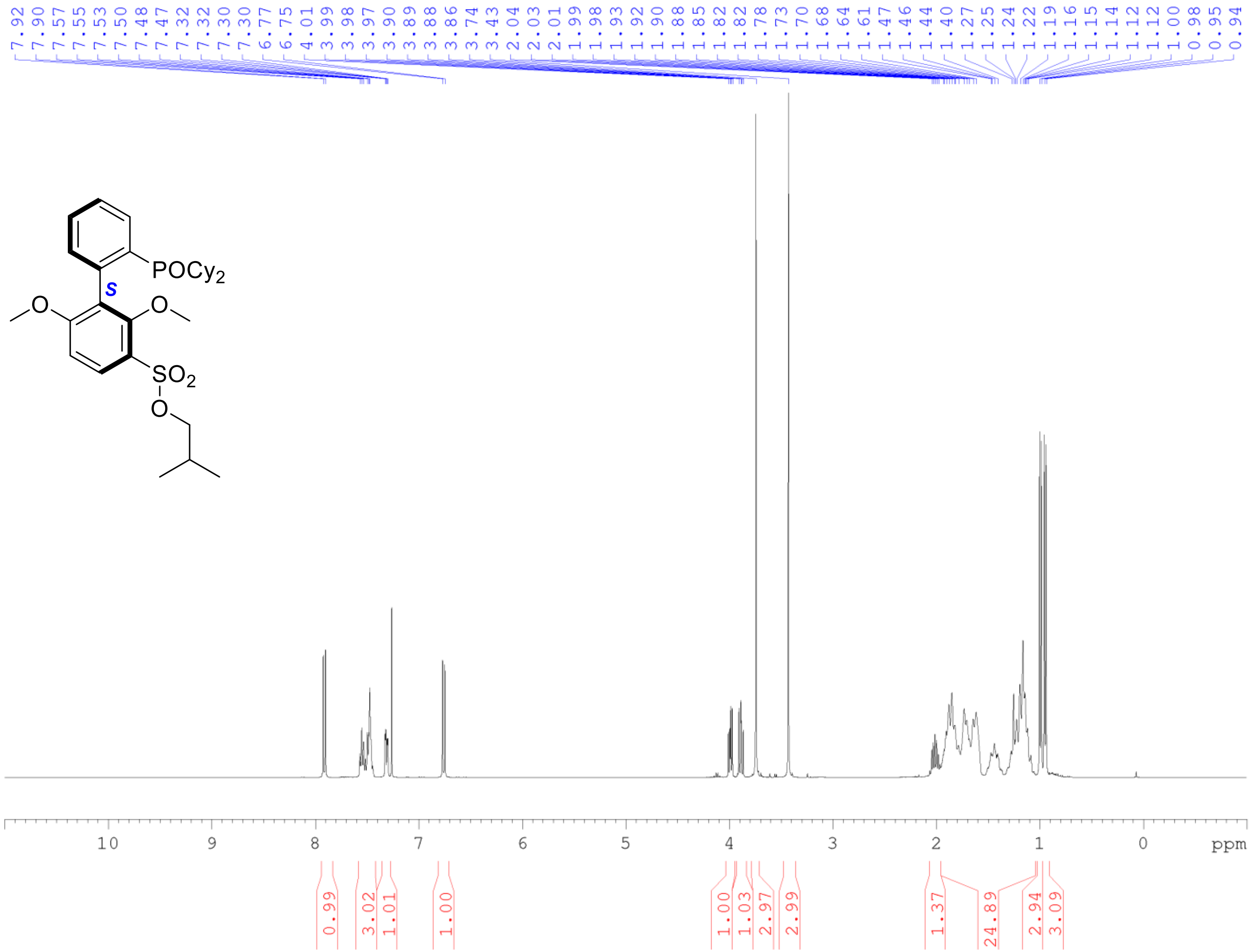

**13C NMR** (CDCl$_3$): Isobutyl (S)-2'-(dicyclohexylphosphoryl)-2,6-dimethoxy-[1,1'-biphenyl]-3-sulfonate (*S*)-**12**

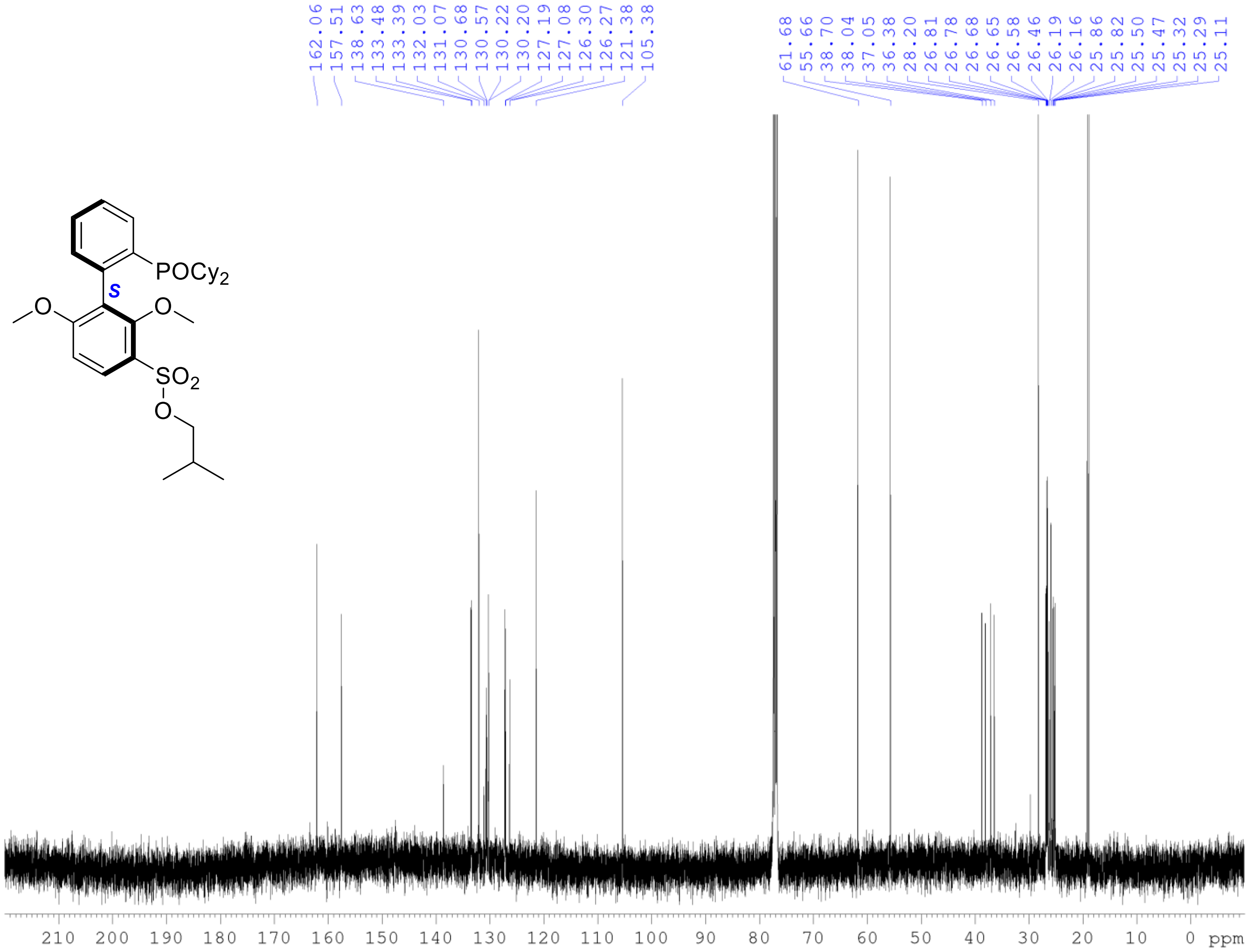

**$^{31}$P NMR** (CDCl$_3$): Isobutyl (S)-2'-(dicyclohexylphosphoryl)-2,6-dimethoxy-[1,1'-biphenyl]-3-sulfonate (*S*)-**12**

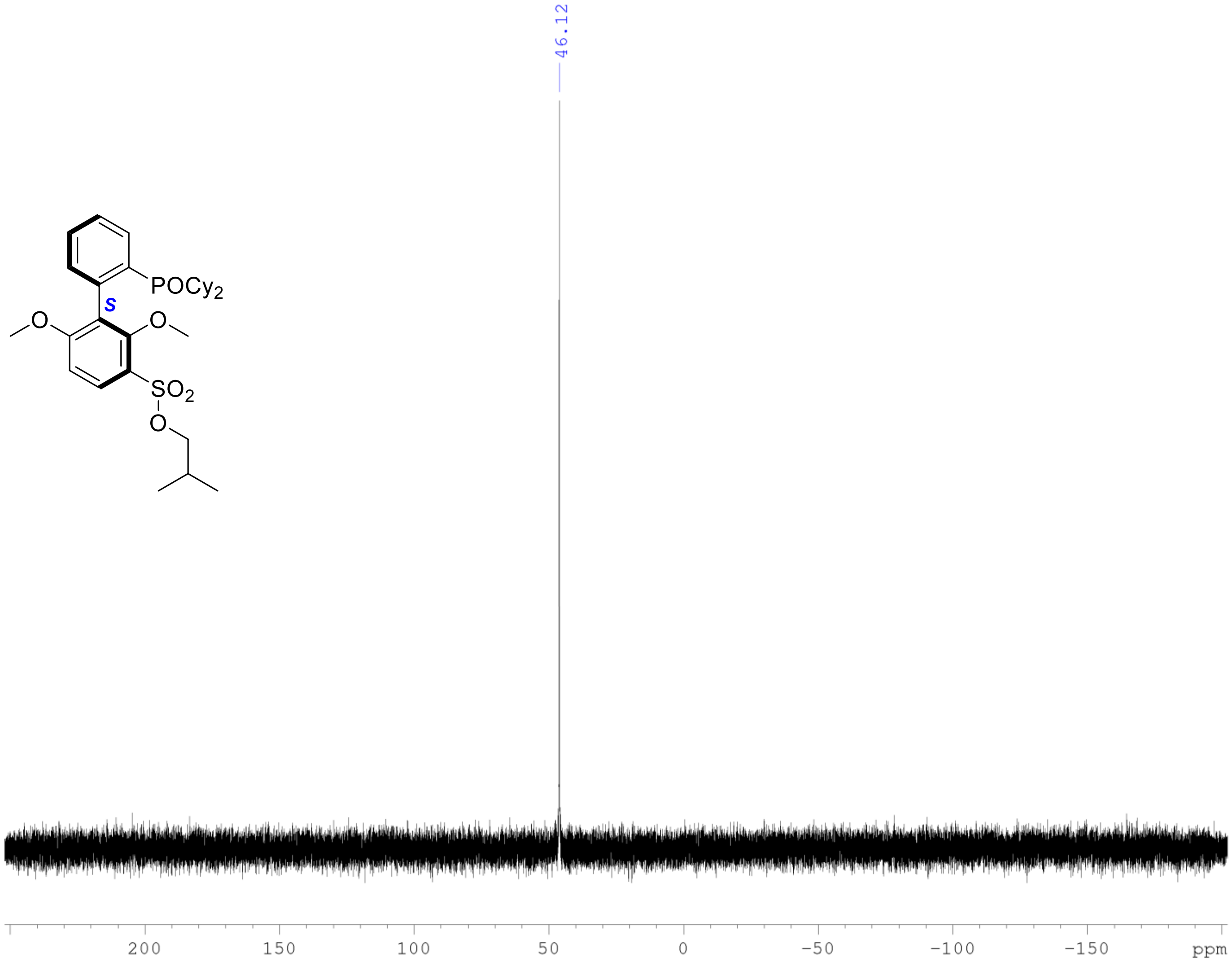

**$^1$H NMR** ($CDCl_3$): Isobutyl (S)-2'-(dicyclohexylphosphaneyl)-2,6-dimethoxy-[1,1'-biphenyl]-3-sulfonate (*S*)-**13**

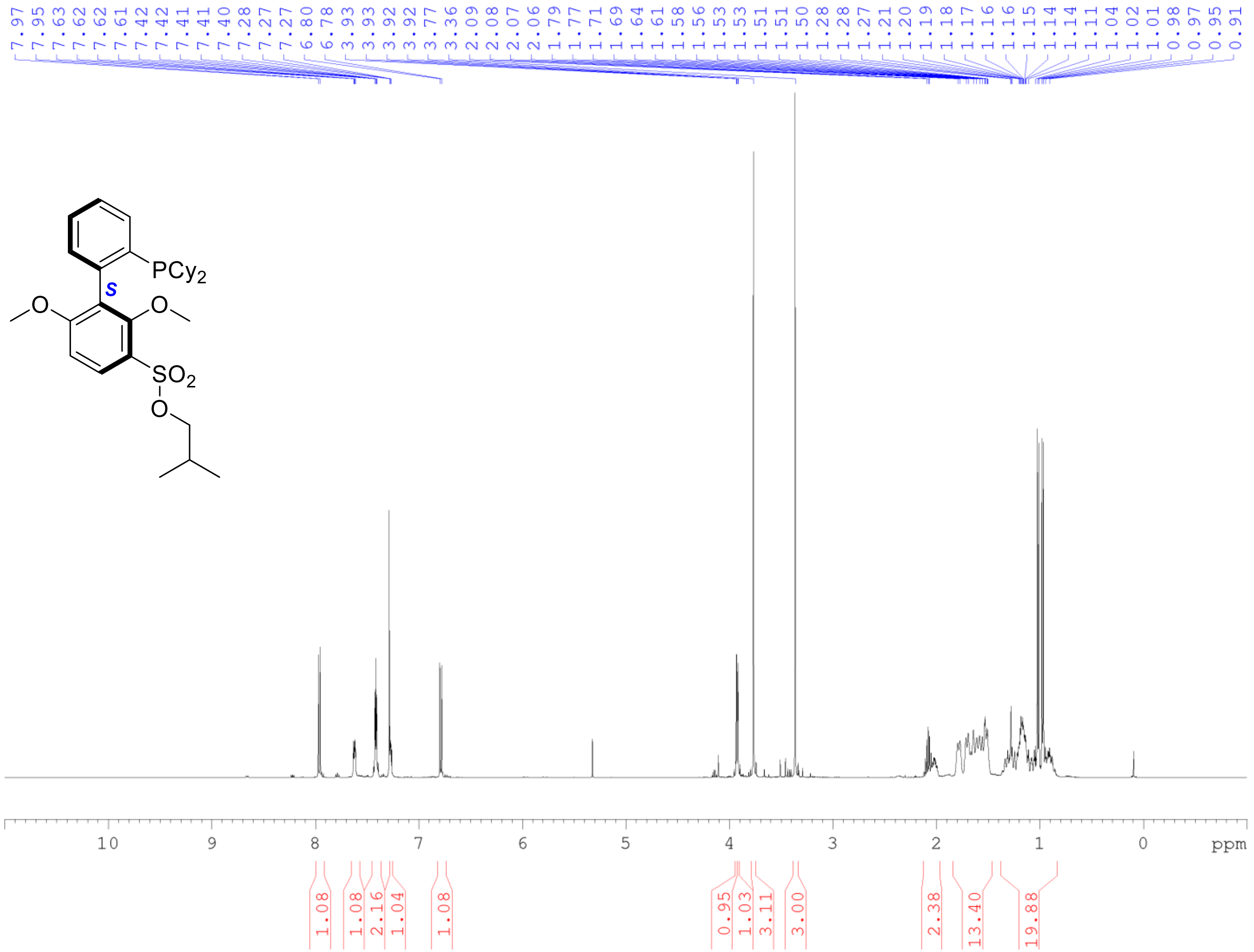

**$^{13}$C NMR** ($CDCl_3$): Isobutyl (S)-2'-(dicyclohexylphosphaneyl)-2,6-dimethoxy-[1,1'-biphenyl]-3-sulfonate (*S*)-**13**

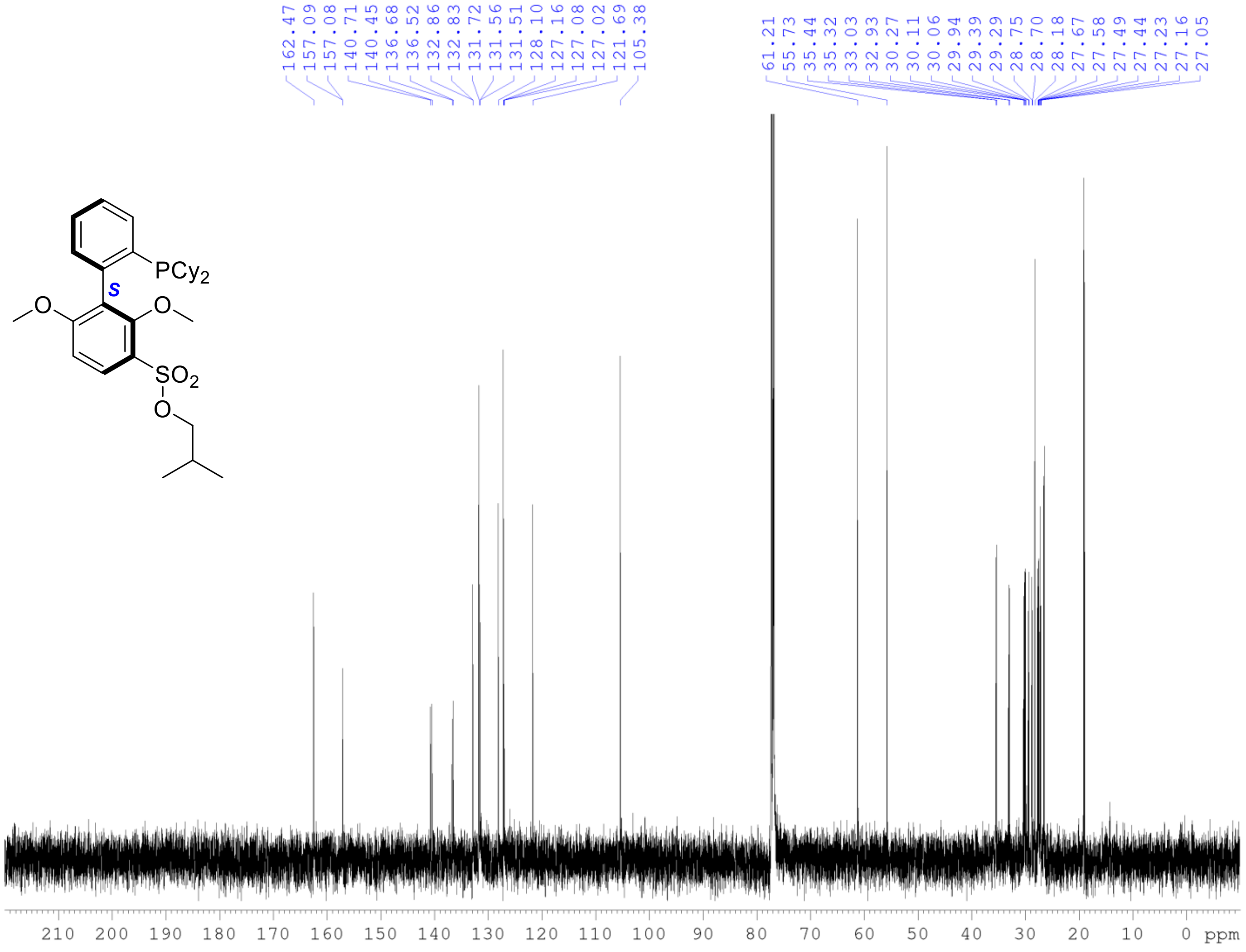

**$^{31}$P NMR** ($CDCl_3$): Isobutyl (S)-2'-(dicyclohexylphosphaneyl)-2,6-dimethoxy-[1,1'-biphenyl]-3-sulfonate (*S*)-**13**

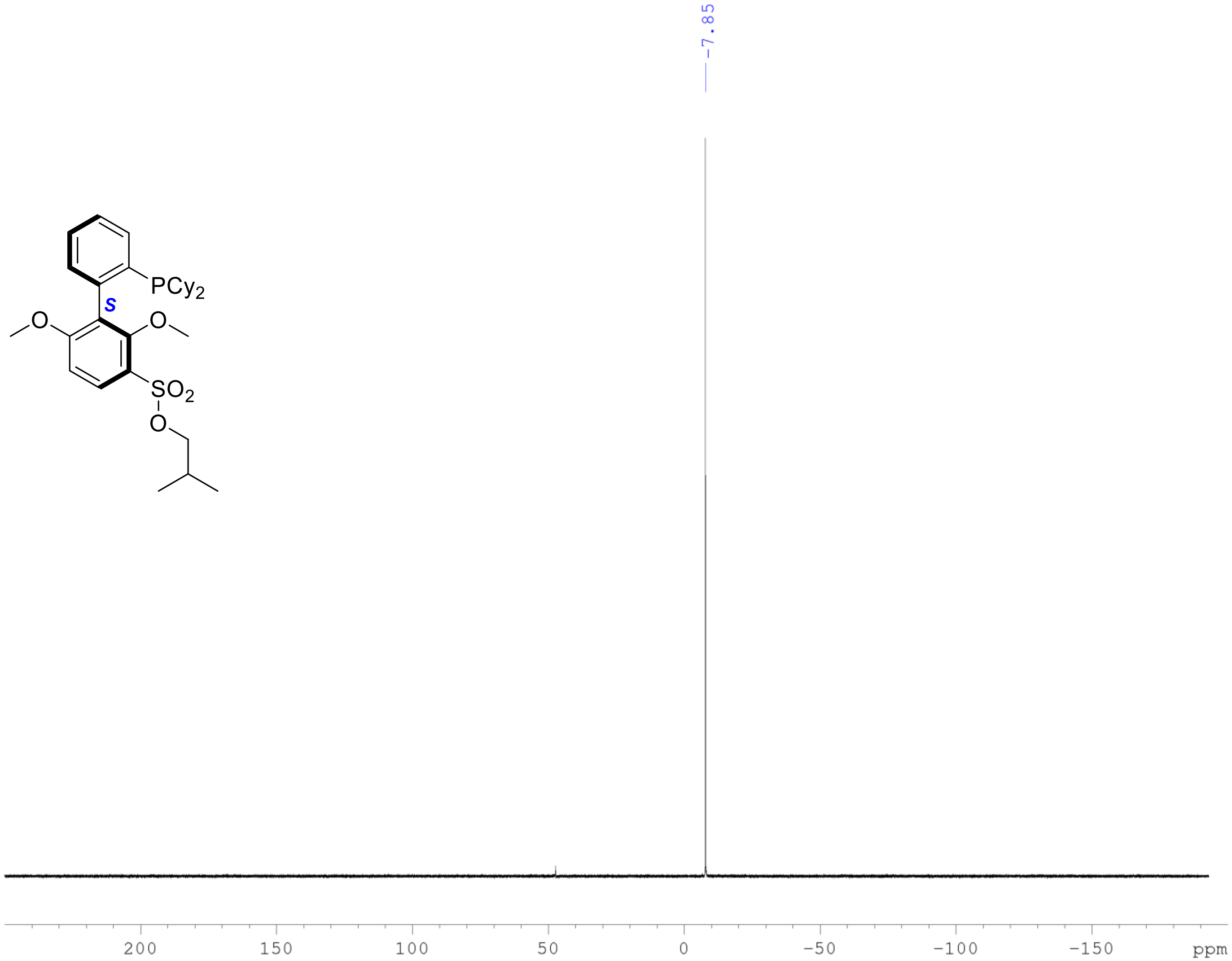

$^{1}$H NMR ($CDCl_3$): Commercially obtained quinidine (Acros) with approximately 10% dihydroquinidine

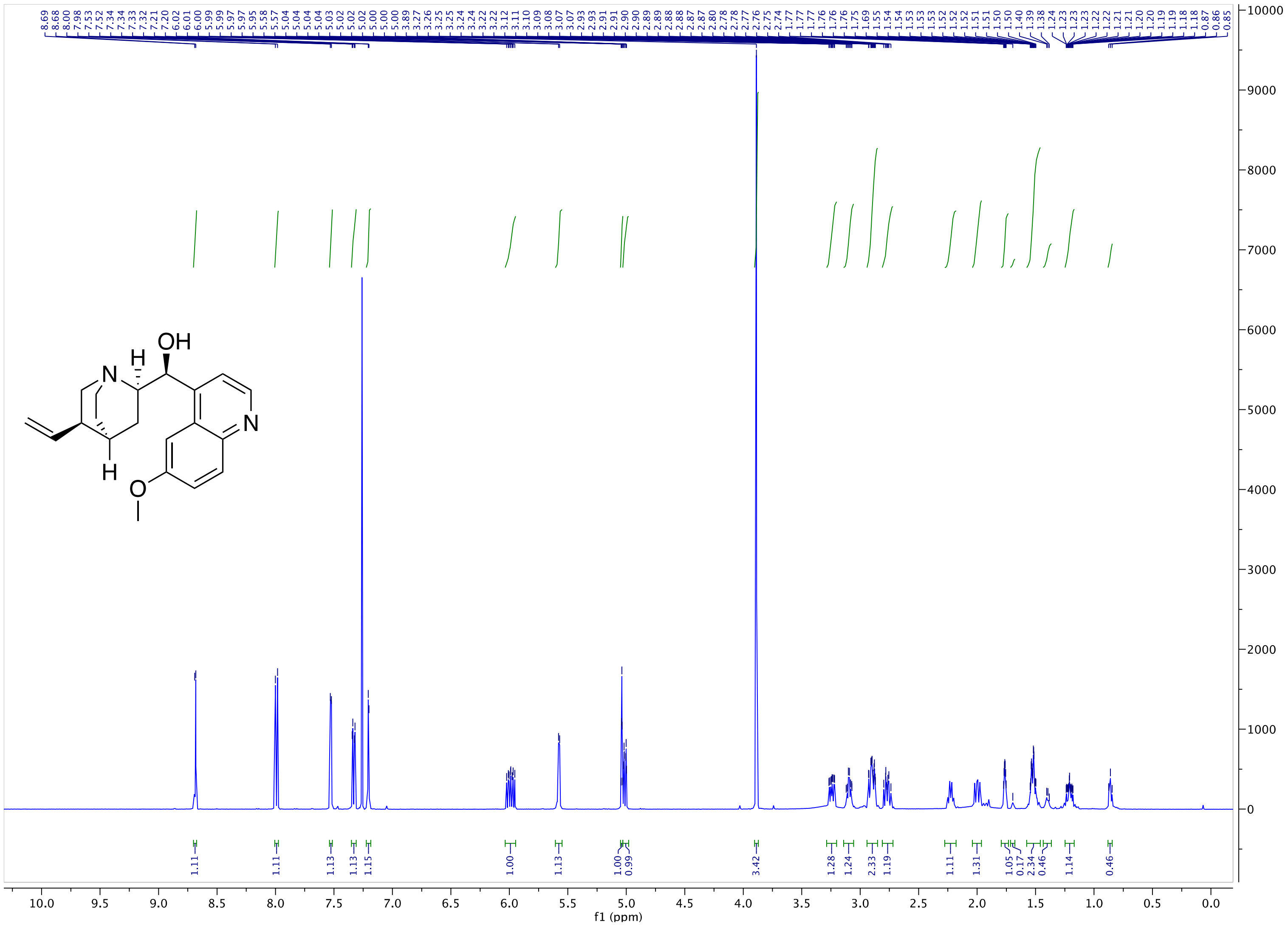

[1]**H NMR** (CDCl$_3$): Quinidinium trifluoroacetate (with approximately 18 mol% dihydroquinidinium trifluoroacetate)

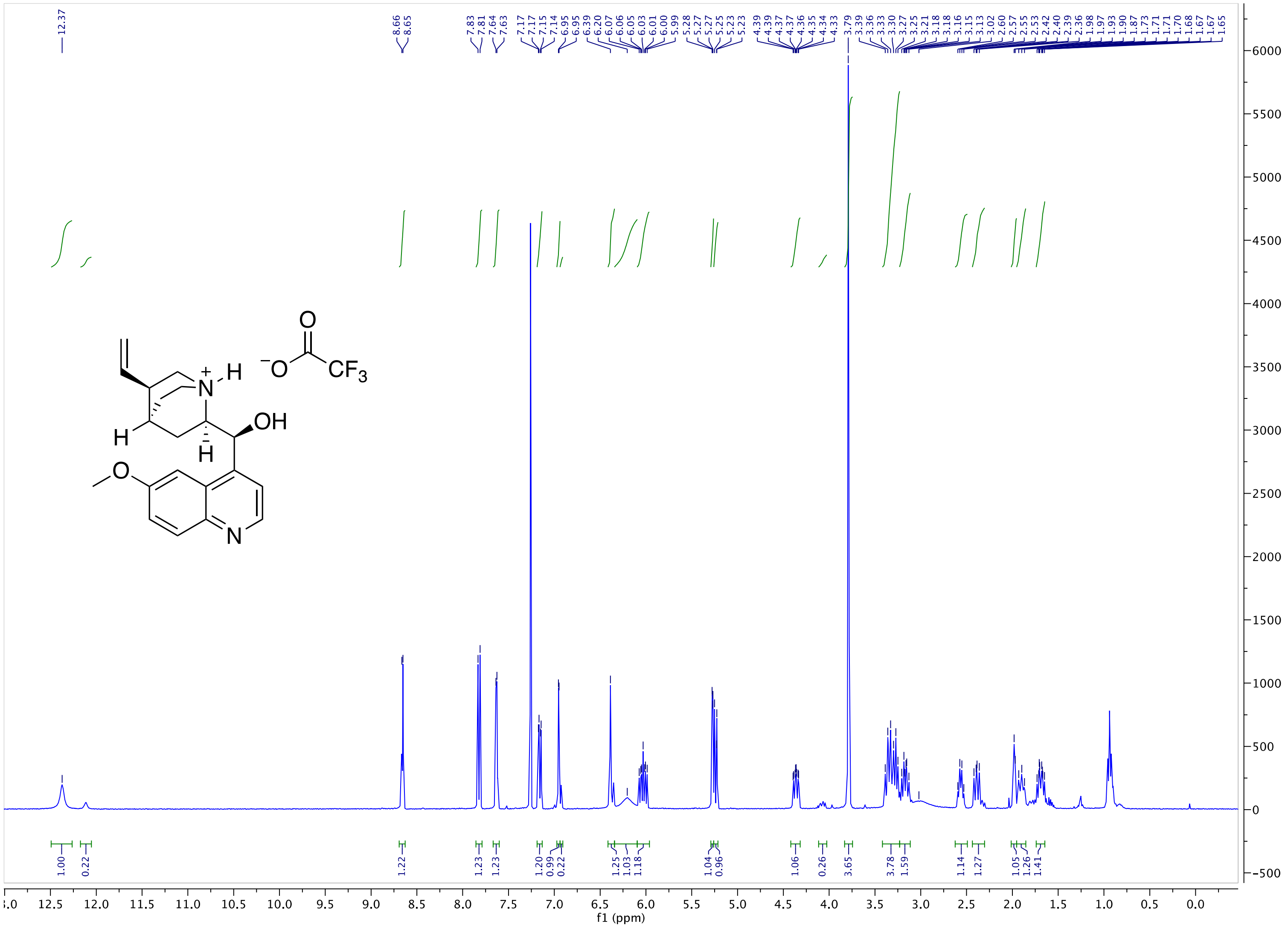

**$^{13}$C NMR** ($CDCl_3$): Quinidinium trifluoroacetate (with approximately 18 mol% dihydroquinidinium trifluoroacetate). Peak picking showing quinidinium trifluoroacetate peaks only.

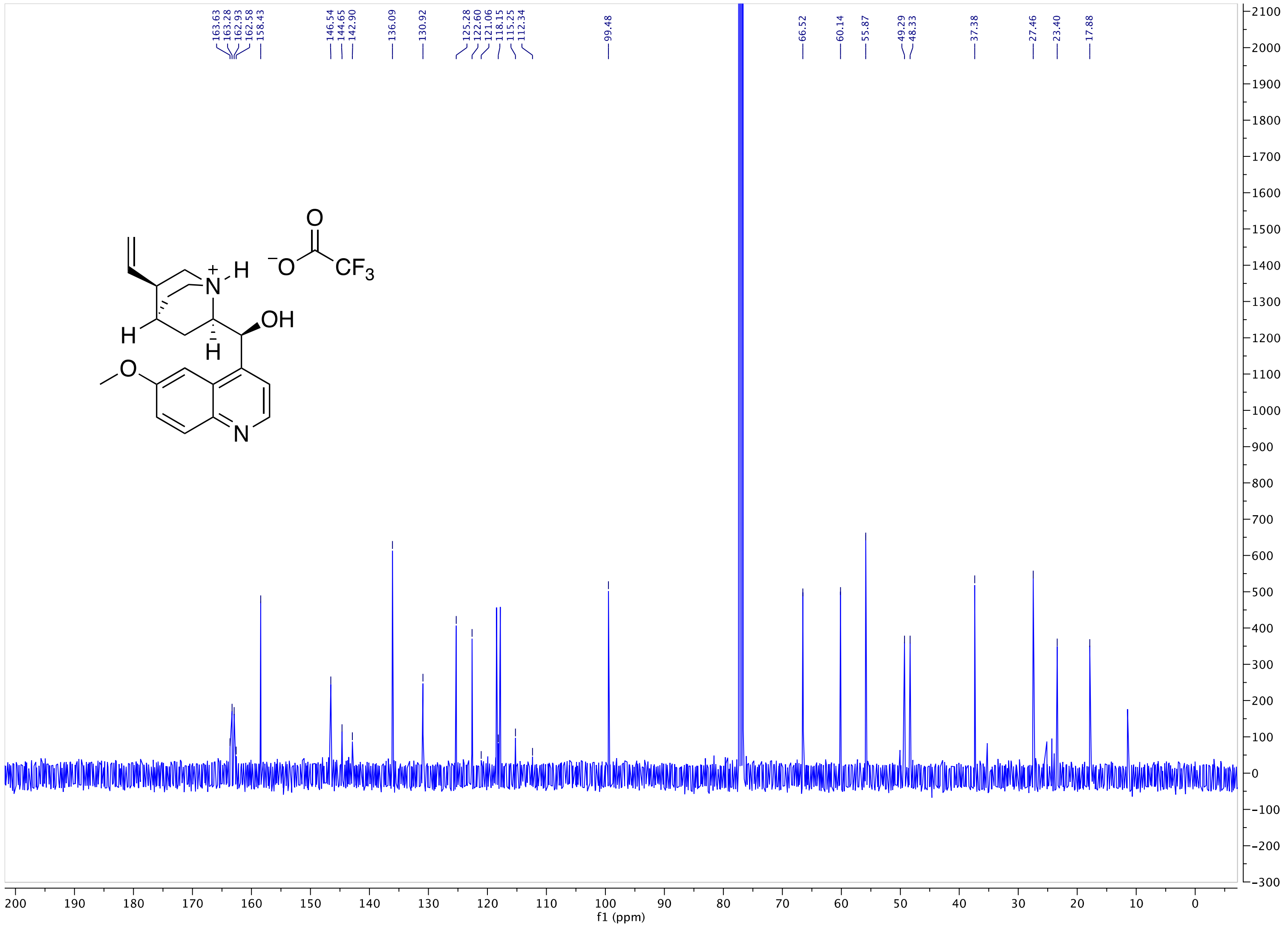

**$^{19}$F NMR** ($CDCl_3$): Quinidinium trifluoroacetate

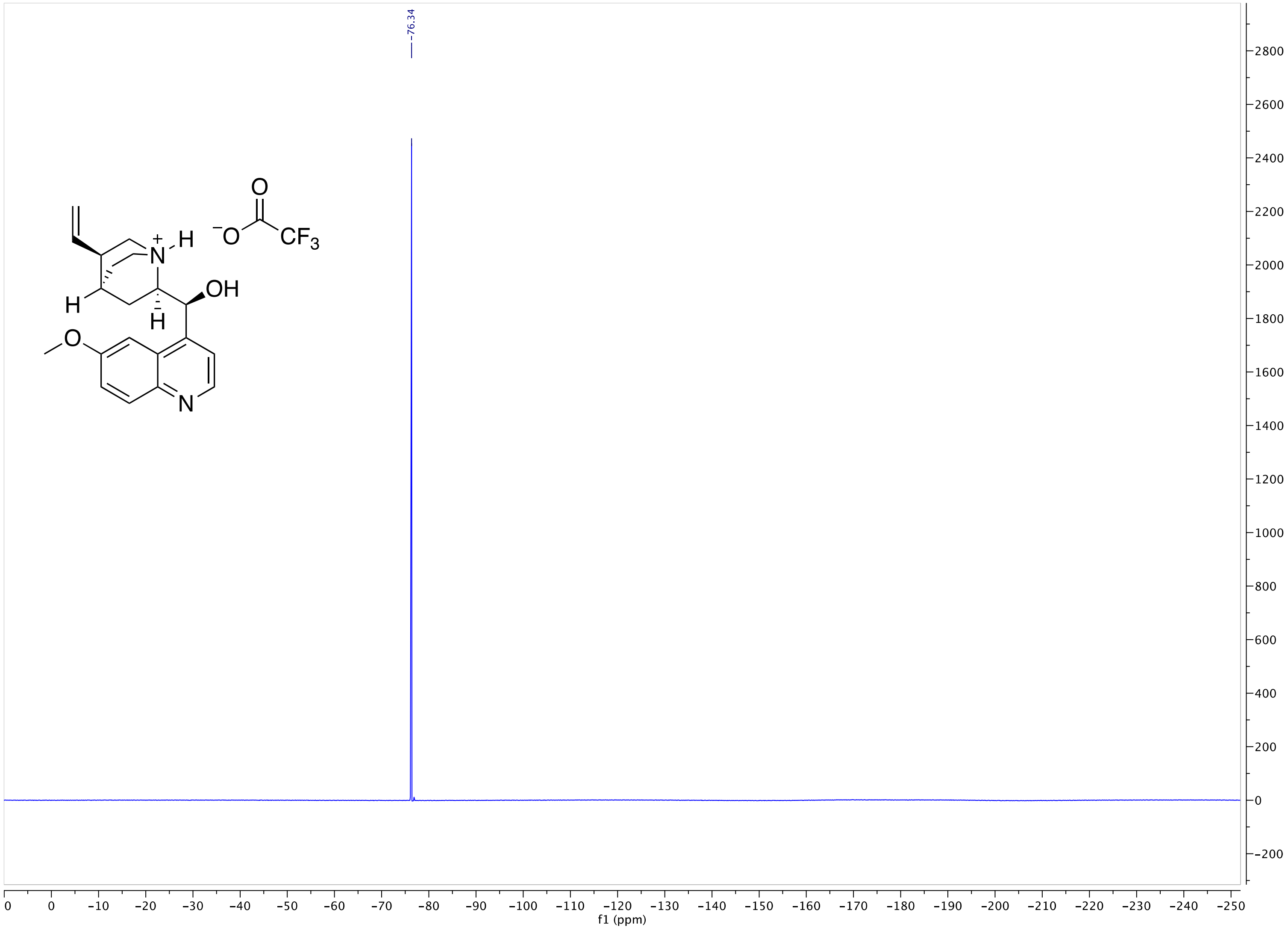

**$^{1}$H NMR** (500 MHz, $CDCl_3$) Quinidinium sSPhos salt before MeCN recrystallisation containing ~15% of dihydroquinidine impurity

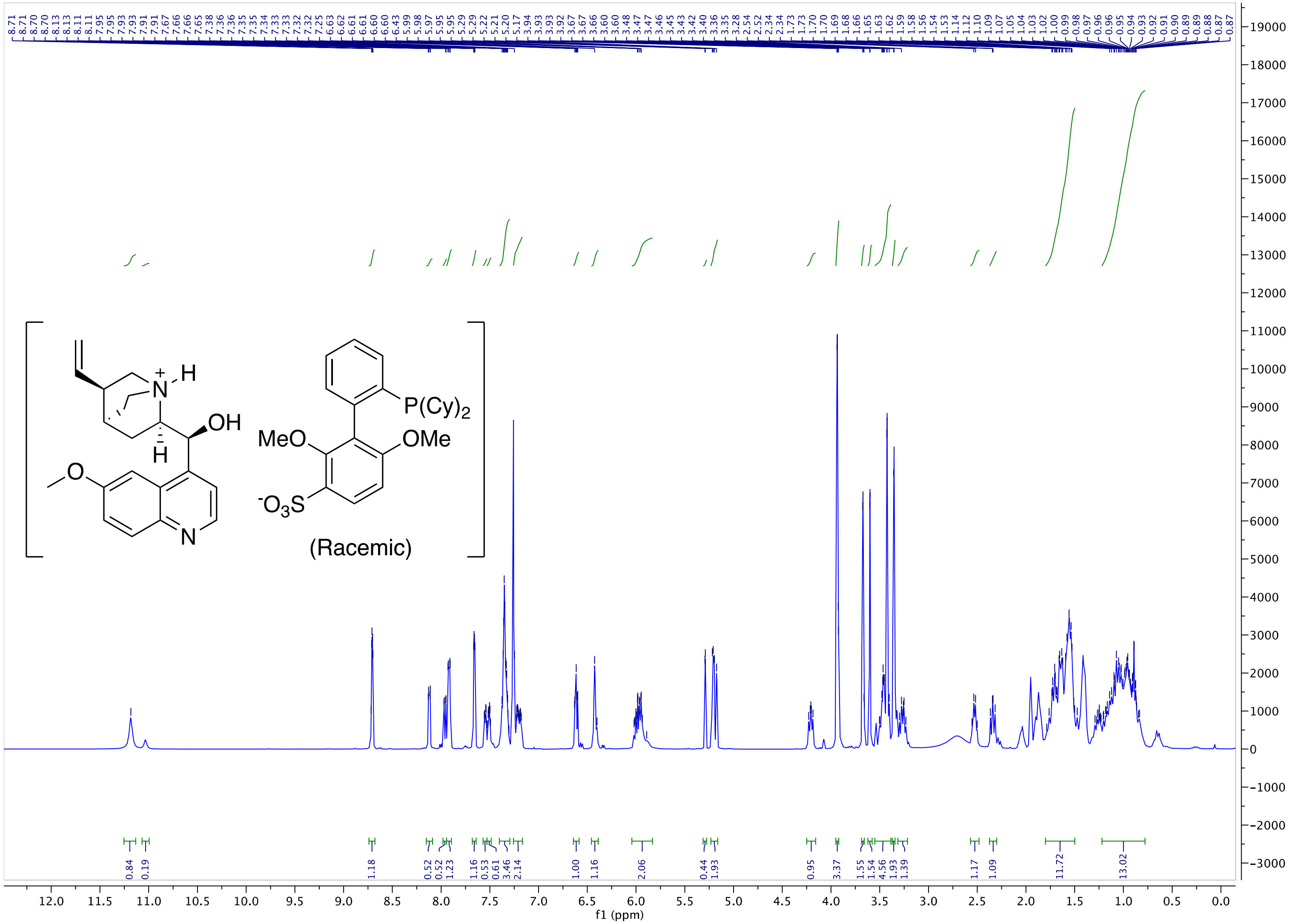

**$^{13}$C NMR** (126 MHz, $CDCl_3$) Quinidinium sSPhos salt before MeCN recrystallisation containing ~15% of dihydroquinidine impurity

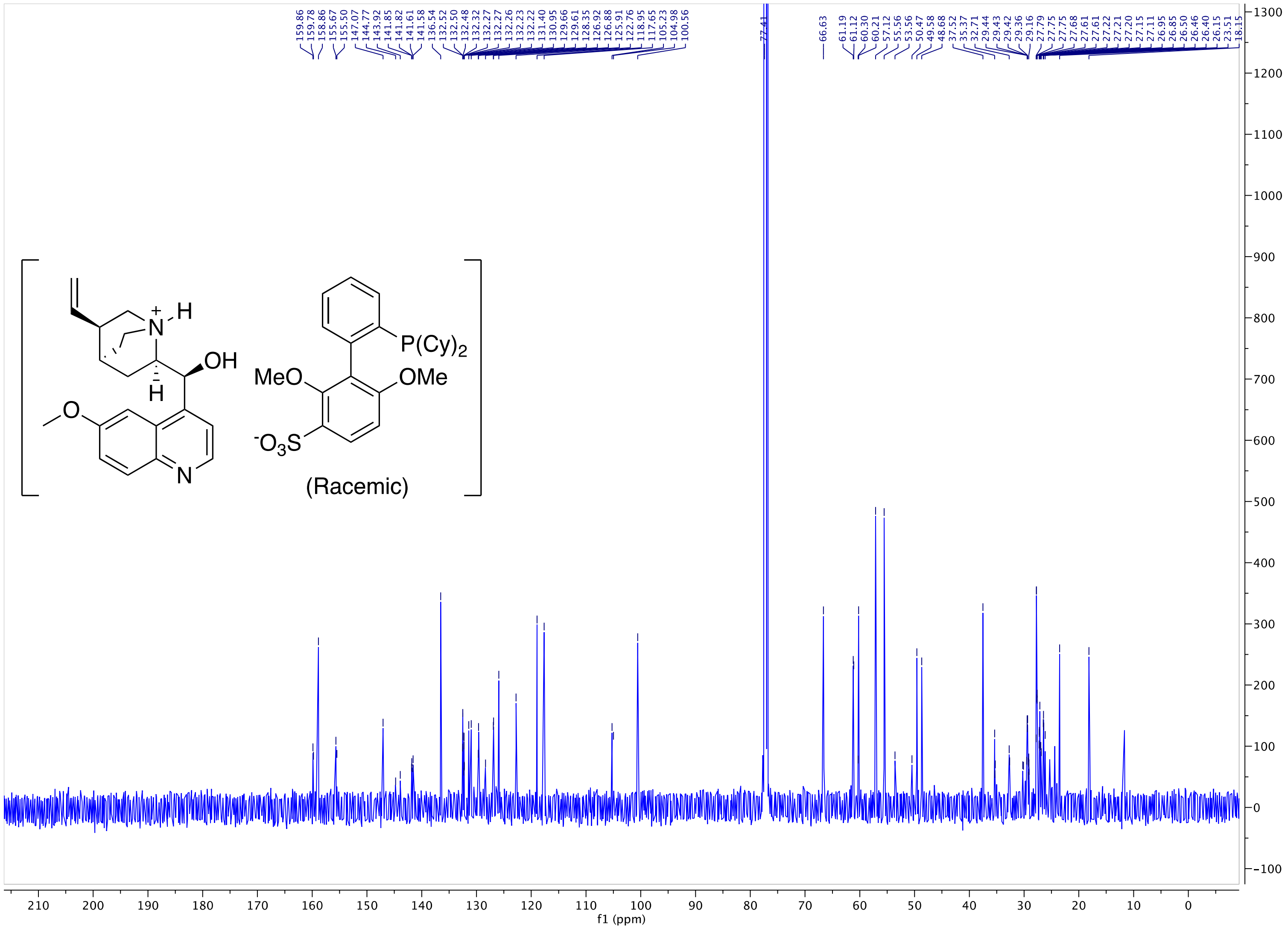

$^{31}$**P NMR** (162 MHz, $CDCl_3$) Quinidinium sSPhos salt before MeCN recrystallisation containing ~15% of dihydroquinidine impurity and 1-2% oxidised sSPhos

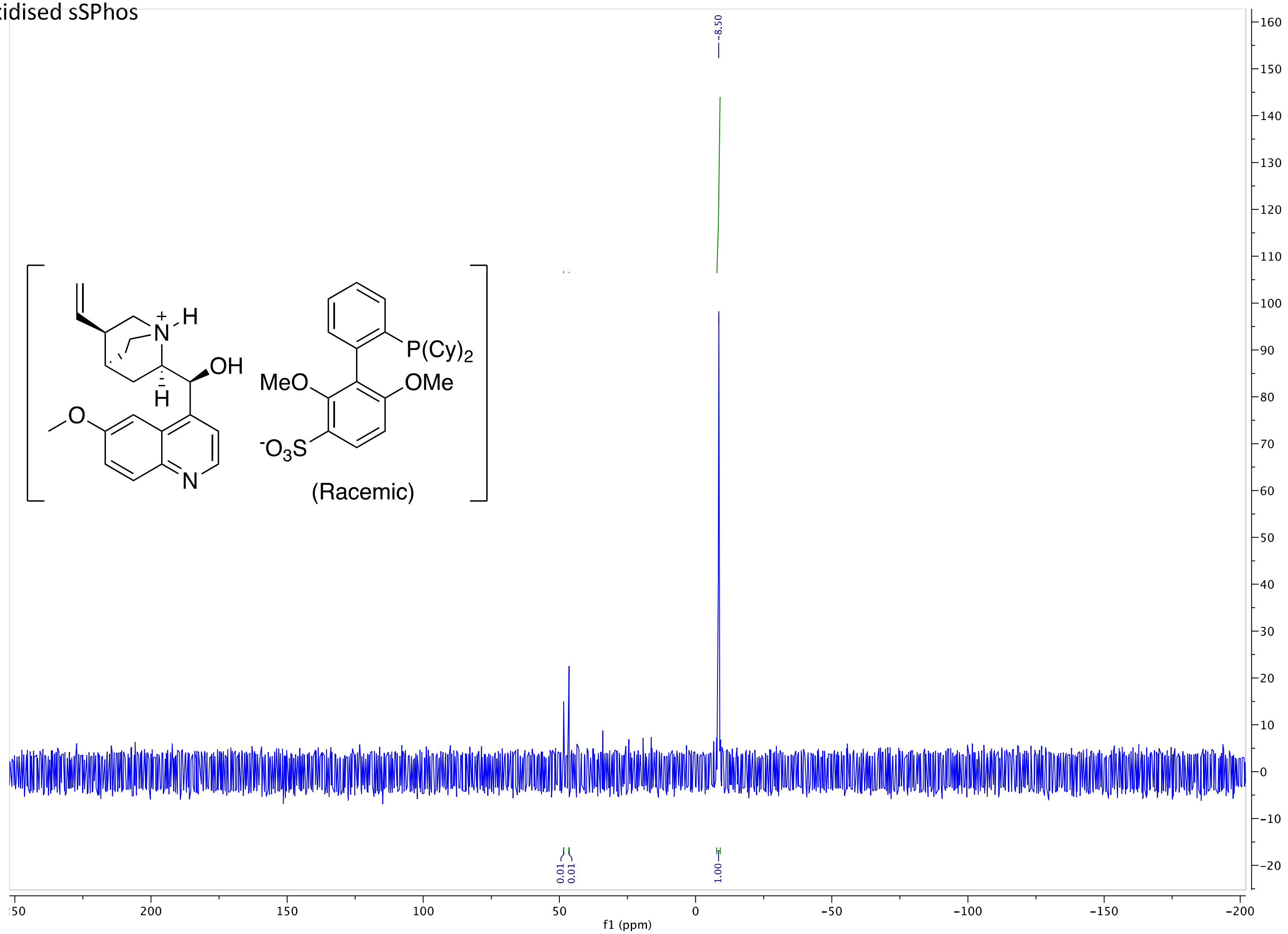

**$^{1}$H NMR (**400 MHz $CDCl_3$): Quinidinium sSPhos salt after single recrystallisation (containing ~12% of dihydroquinidine impurity)

Material obtained from Experiment number 1 in SI table.

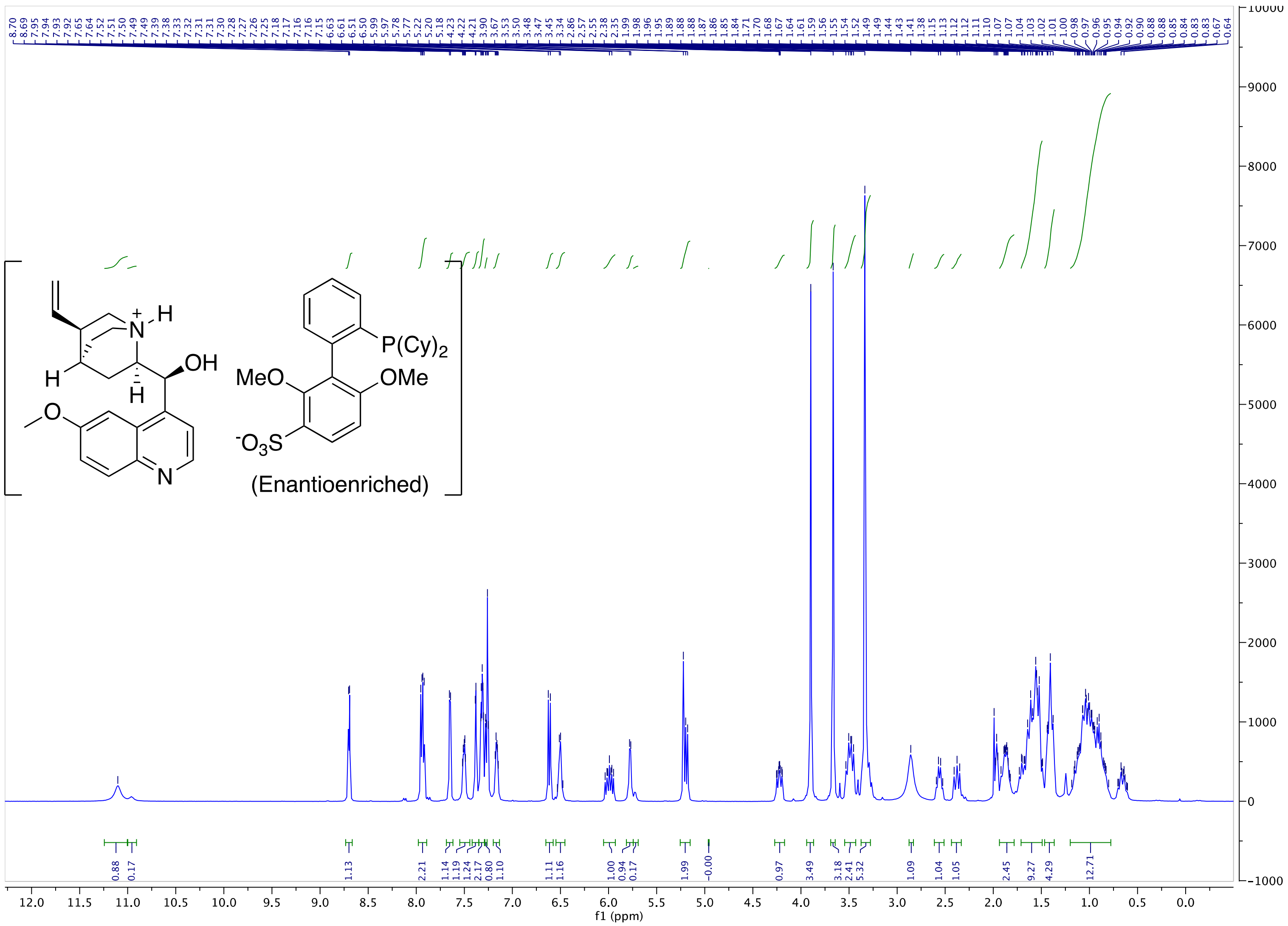

**$^{13}$C NMR** (101 MHz $CDCl_3$): Quinidinium sSPhos salt after single recrystallisation containing ~12% of dihydroquinidine impurity

Material obtained from Experiment number 1 in SI table.

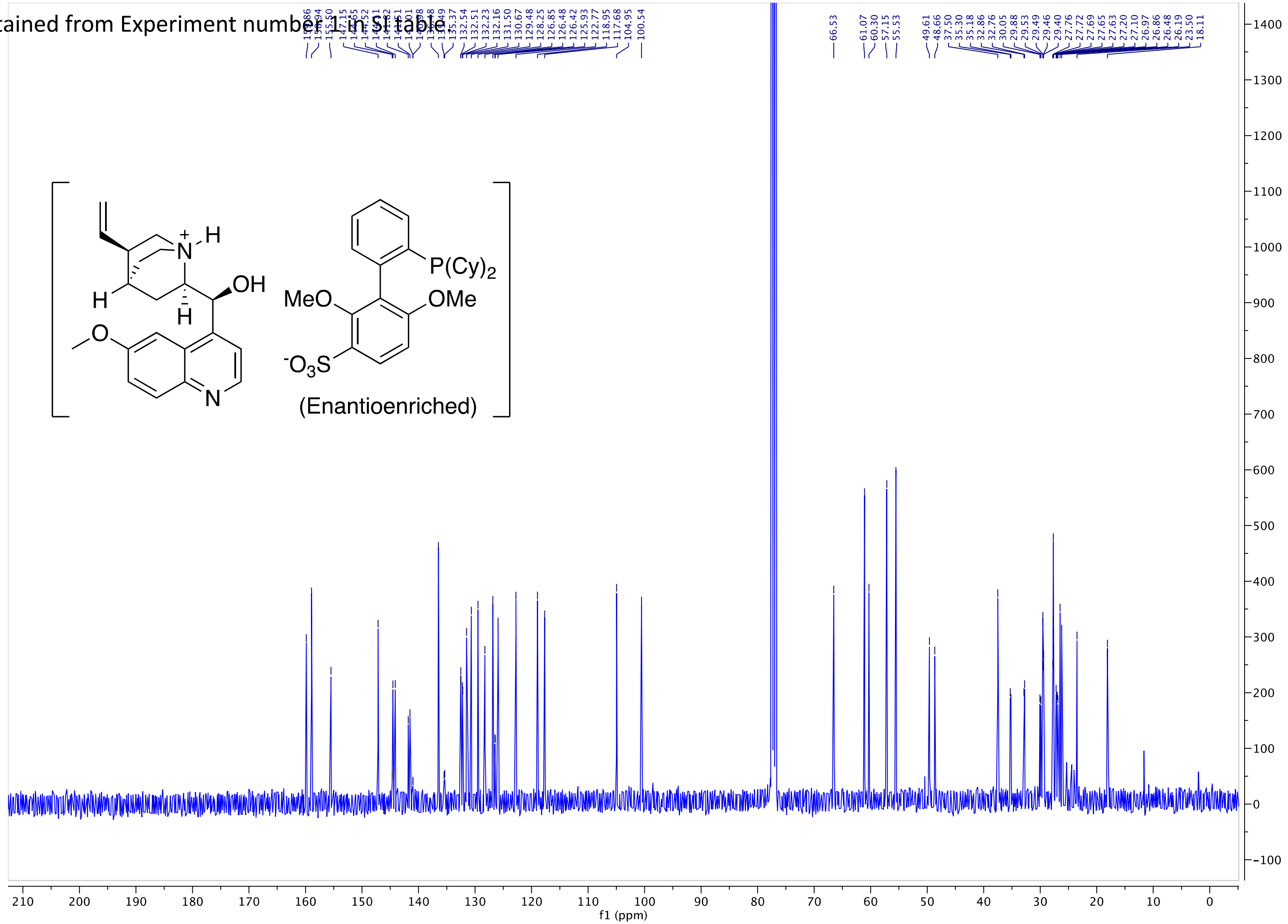

$^{31}$**P NMR** (162 MHz $CDCl_3$): Quinidinium sSPhos salt after single recrystallisation containing ~12% of dihydroquinidine impurity. Material obtained from Experiment number 1 in SI table.

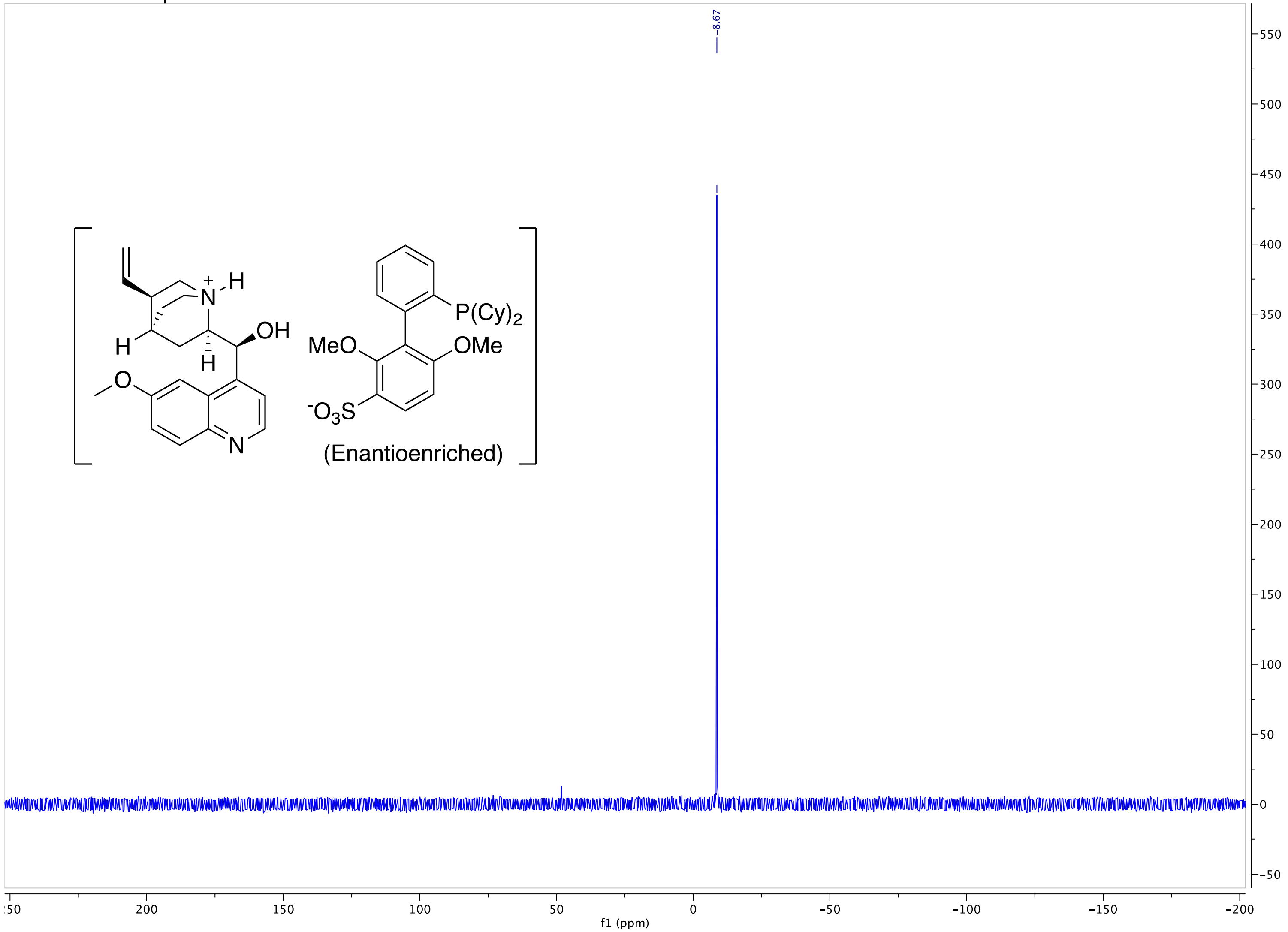

**$^{1}$H NMR** (MeOD): Final (*R*)-sSPhos obtained from double recrystallisation procedure

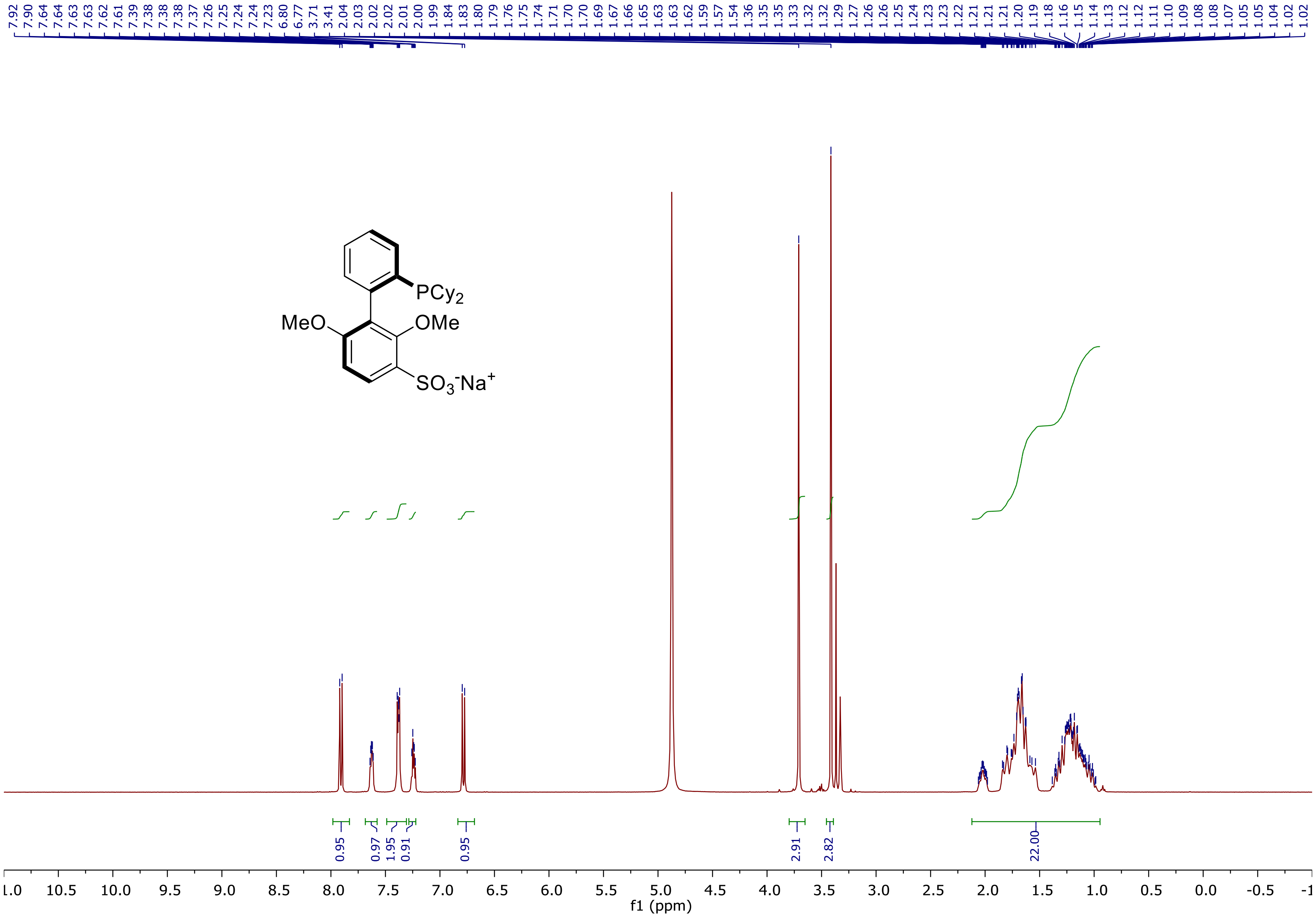

**$^{13}$C NMR** (MeOD): Final (*R*)-sSPhos obtained from double recrystallisation procedure

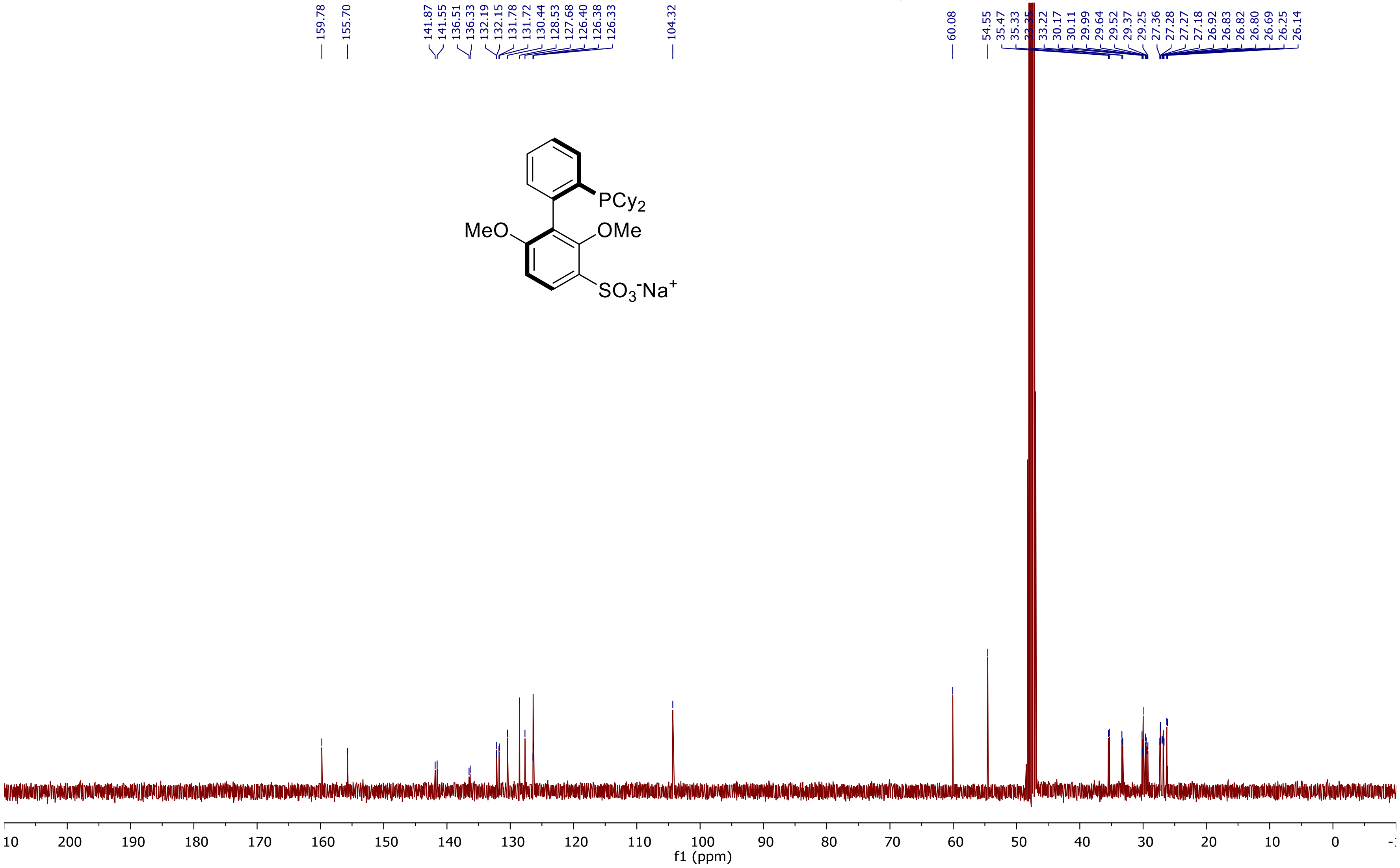

**$^{31}$P NMR** (MeOD): Final (*R*)-sSPhos obtained from double recrystallisation procedure

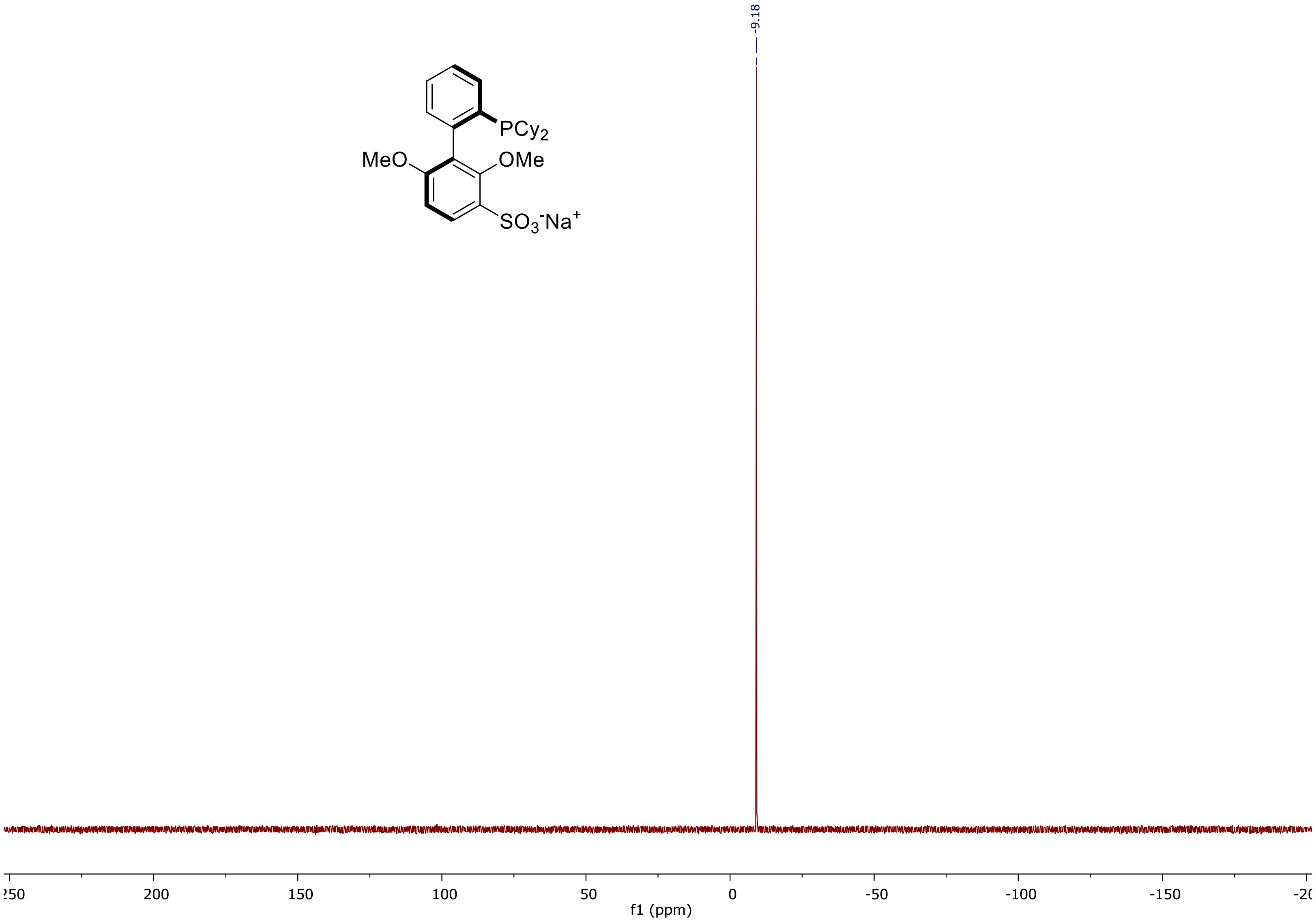

**$^1$H NMR** ($CDCl_3$): neopentyl (R)-2'-(dicyclohexylphosphoryl)-2,6-dimethoxy-[1,1'-biphenyl]-3-sulfonate

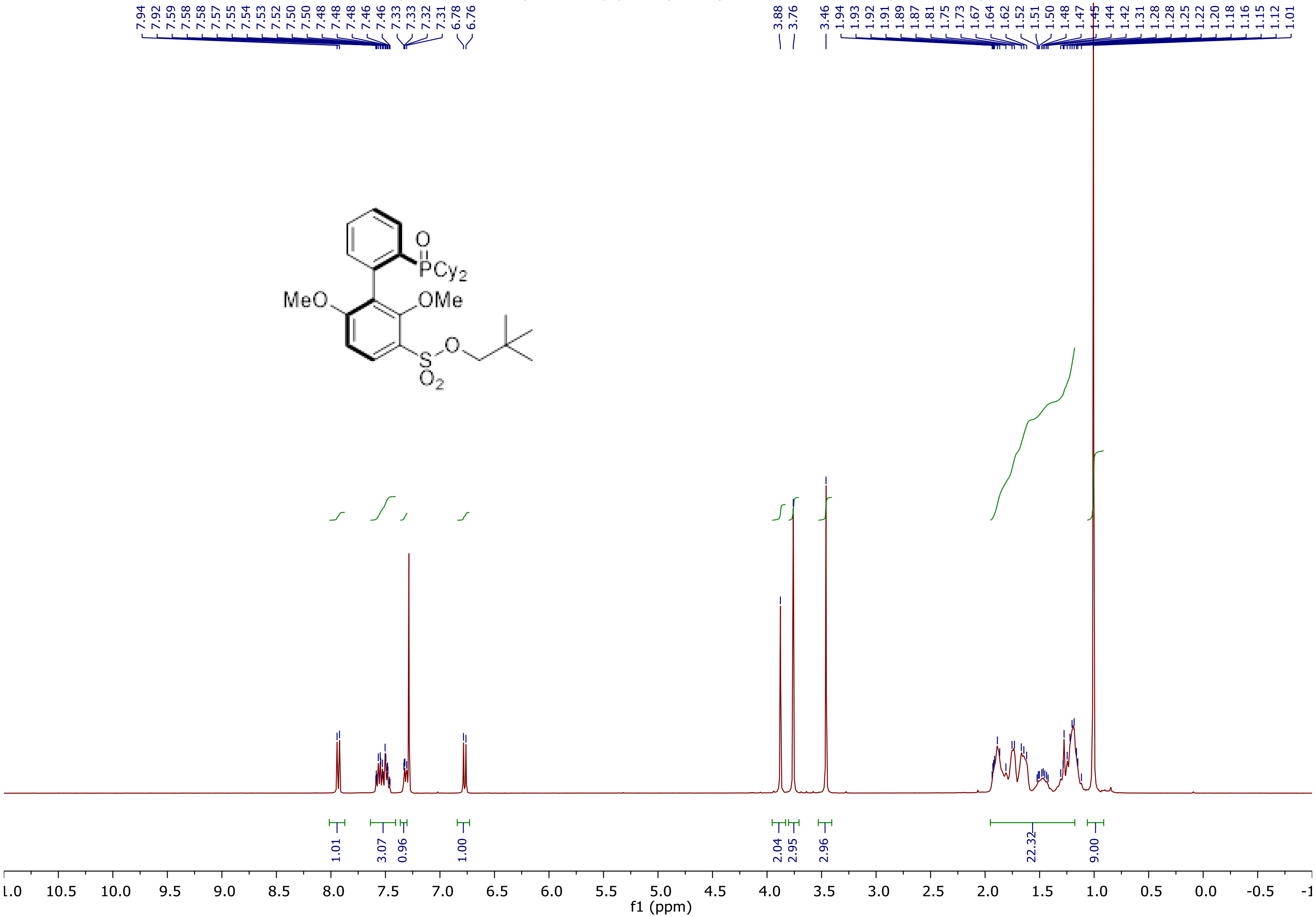

**$^{13}$C NMR** ($CDCl_3$): neopentyl (R)-2'-(dicyclohexylphosphoryl)-2,6-dimethoxy-[1,1'-biphenyl]-3-sulfonate

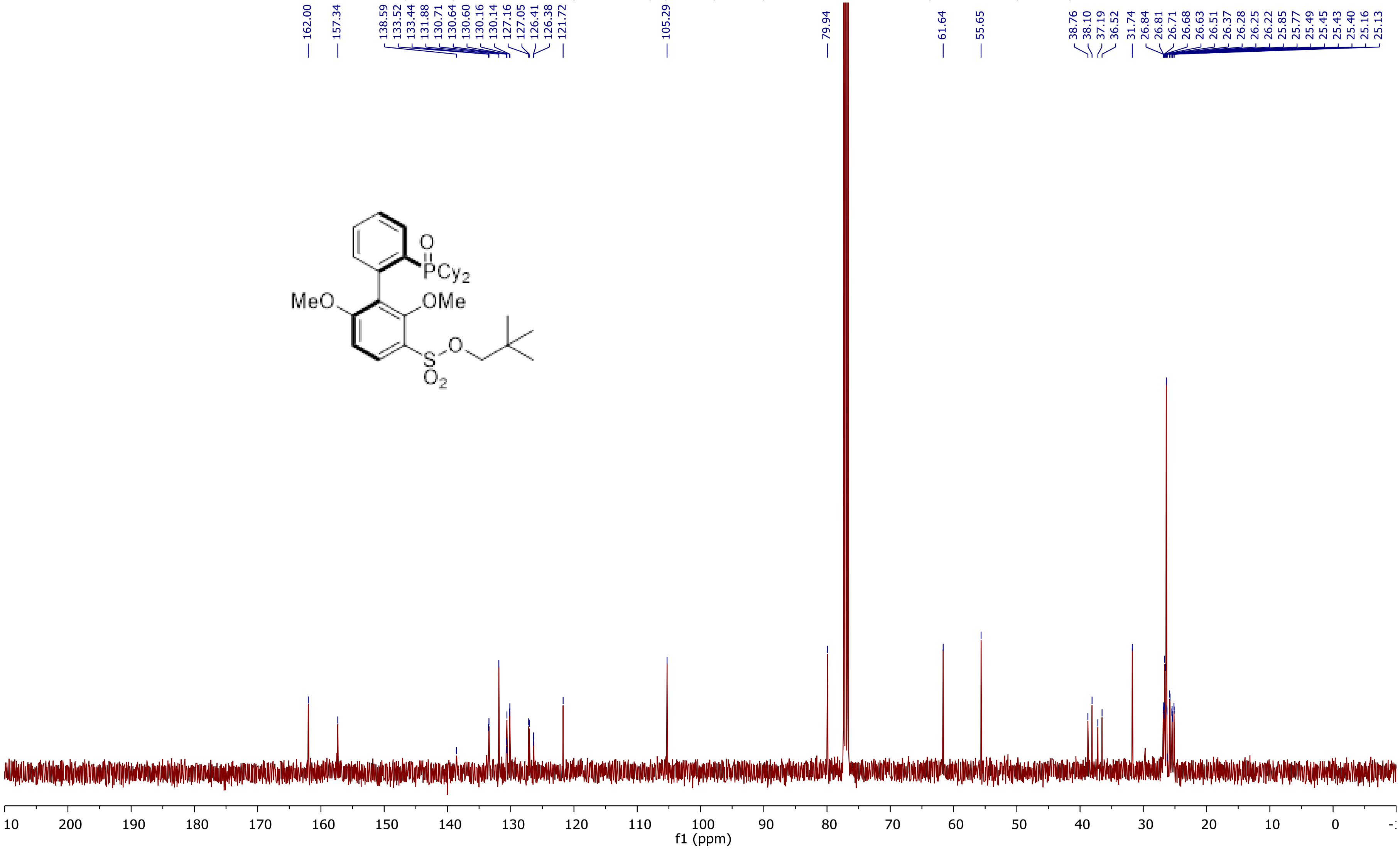

**$^{31}$P NMR** ($CDCl_3$): neopentyl (R)-2'-(dicyclohexylphosphoryl)-2,6-dimethoxy-[1,1'-biphenyl]-3-sulfonate

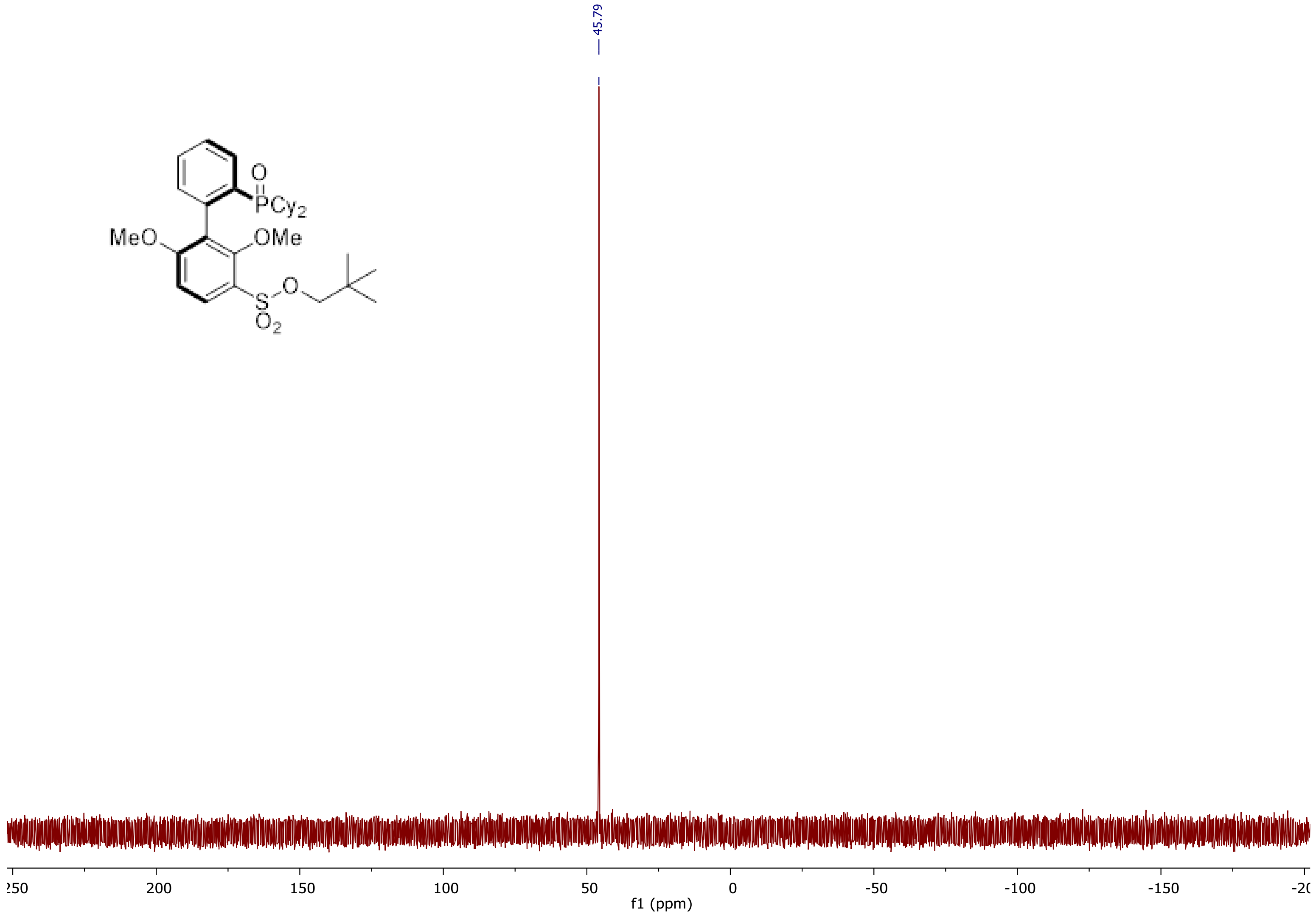

# Chiral SFC Traces

6-fluoro-6'-methyl-[1,1'-biphenyl]-2,2'-diol (**3a**)

**Chiral-SFC** (CHIRAL ART SJ ($CO_2$:MeOH 90:10, 2.5 mL min$^{-1}$, 40 °C) $t_R$ = 5.73 (minor), 6.32 (major) minutes.

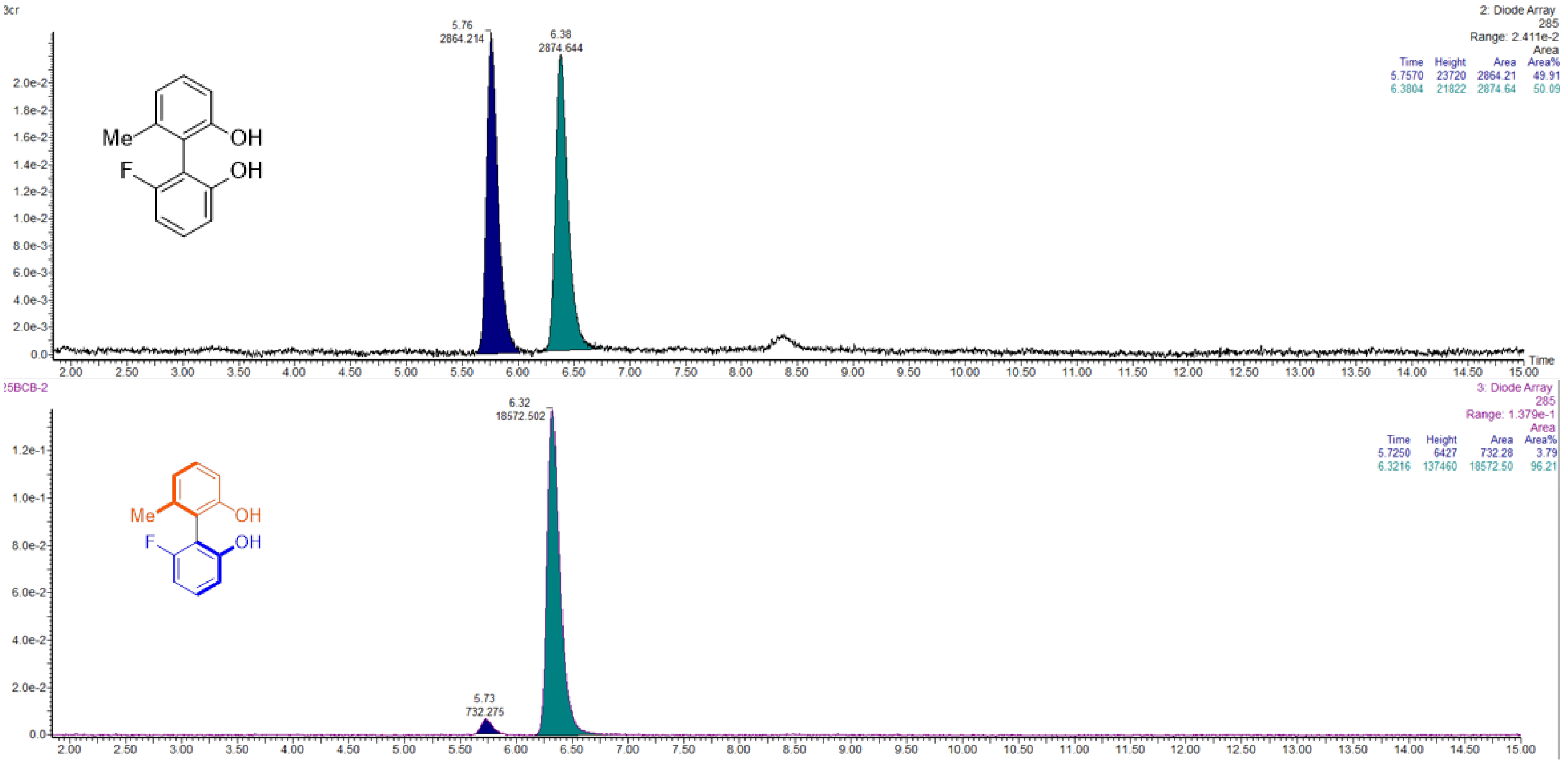

3-chloro-6'-fluoro-6-methyl-[1,1'-biphenyl]-2,2'-diol (**3b**)

**Chiral-SFC** (CHIRAL ART SJ ($CO_2$:MeOH 85:15, 2.5 mL min$^{-1}$, 40 °C) $t_R$ = 4.55 (minor), 5.24 (major) minutes.

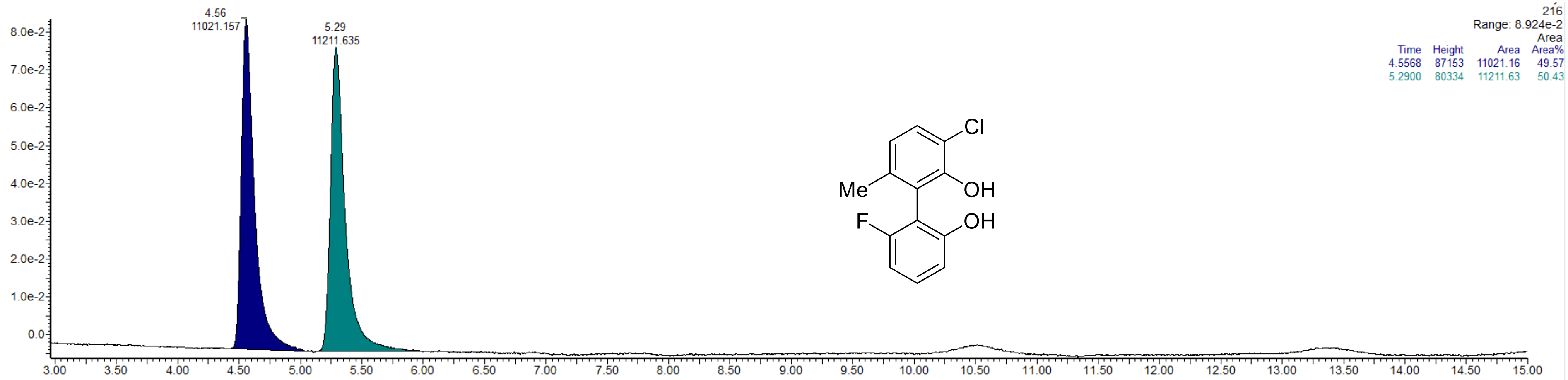


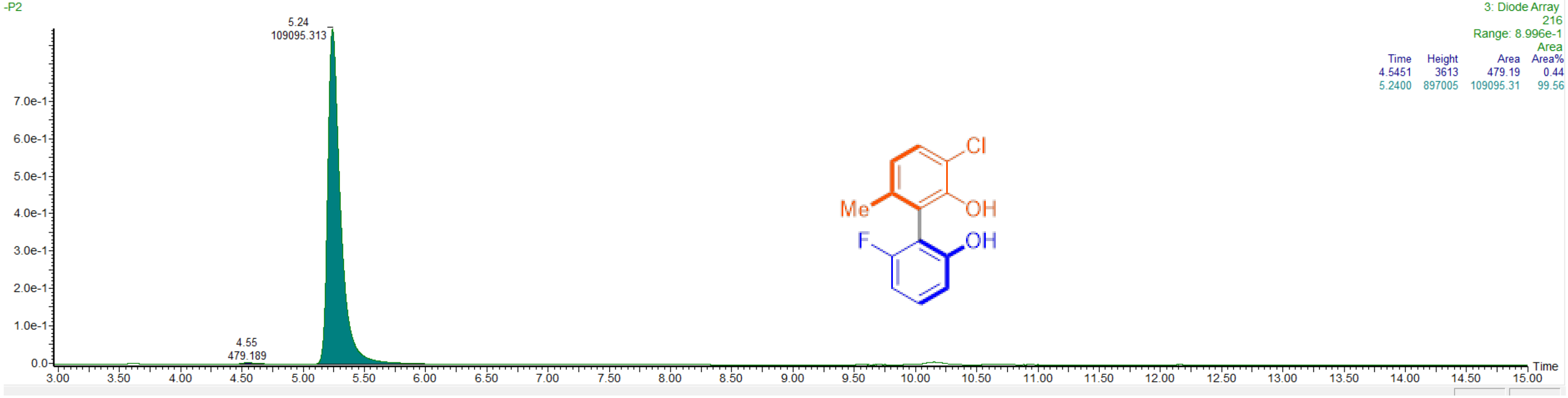

6'-fluoro-4,6-dimethyl-[1,1'-biphenyl]-2,2'-diol (**3c**)

**Chiral-SFC** (CHIRAL ART SJ ($CO_2$:MeOH 93:07, 2.5 mL $min^{-1}$, 40 °C) $t_R$ = 6.64 (minor), 7.25 (major) minutes.

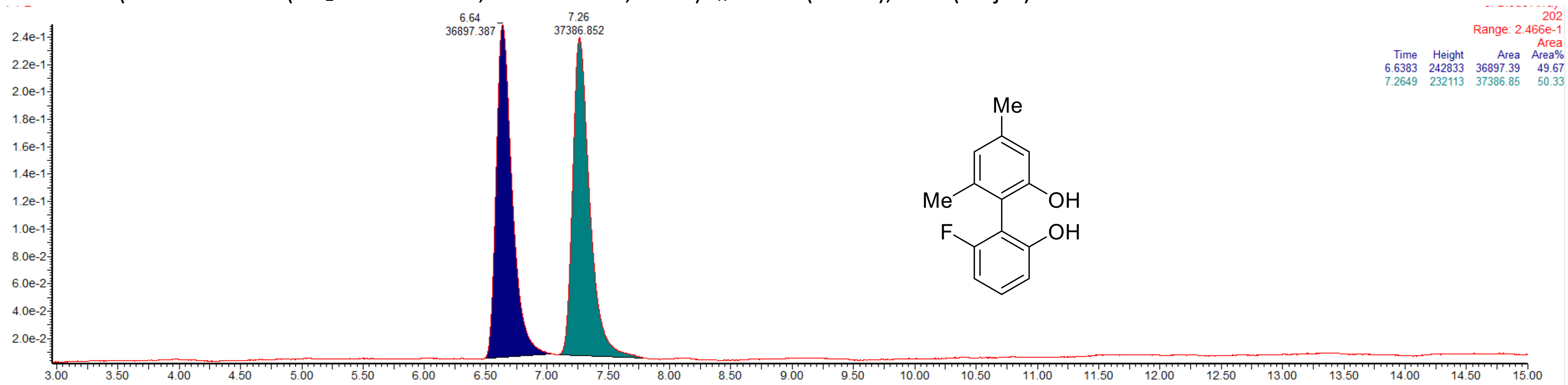


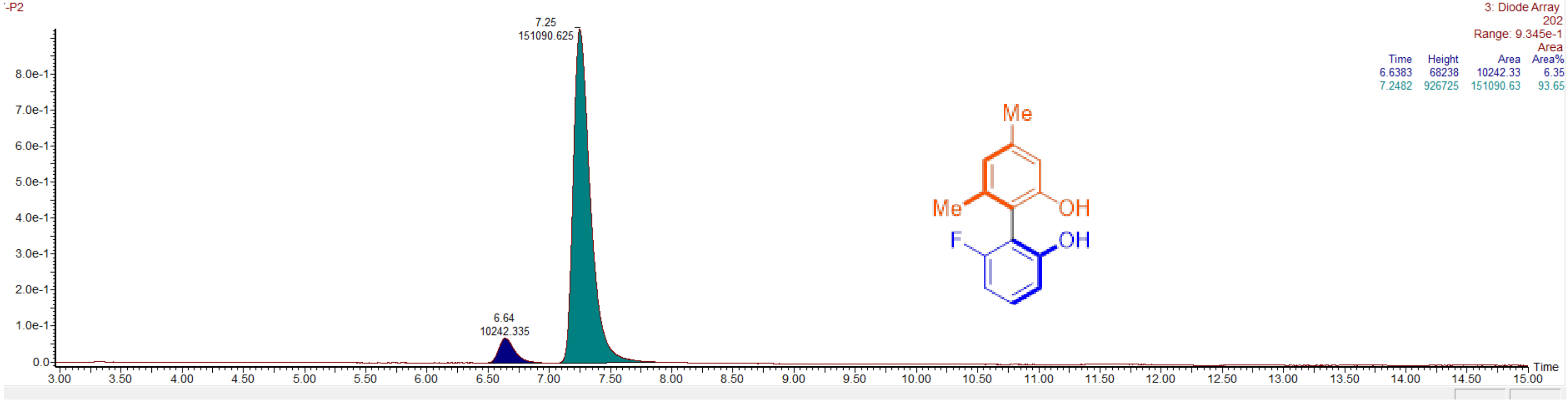

1-(2-fluoro-6-hydroxyphenyl)-5,6,7,8-tetrahydronaphthalen-2-ol (**3d**)

**Chiral-SFC** (CHIRAL ART SJ ($CO_2$:MeOH 93:07, 2.5 mL min$^{-1}$, 40 °C) $t_R$ = 10.87 (minor), 11.76 (major) minutes.

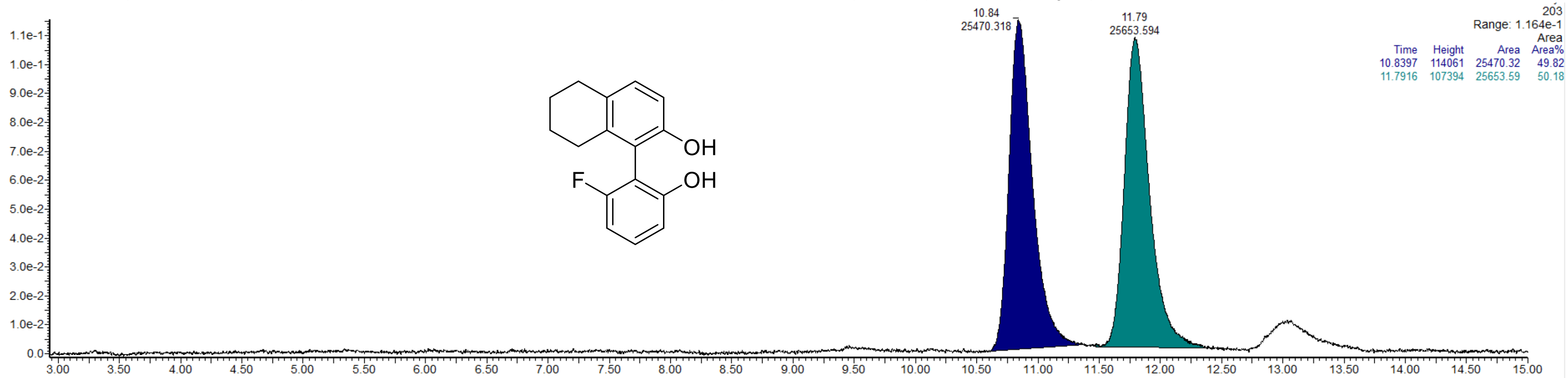


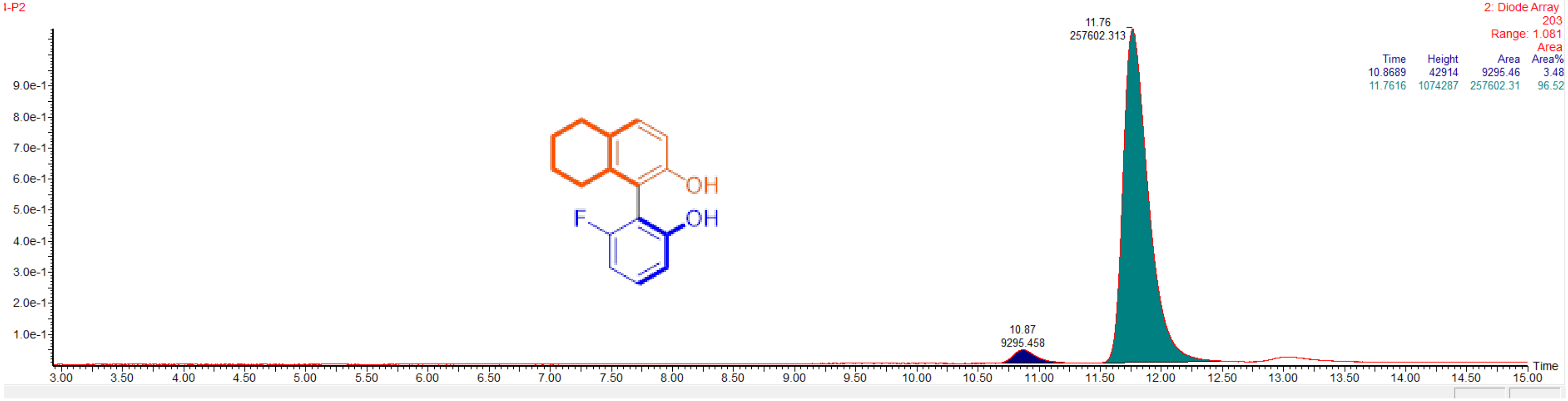

6-chloro-6'-fluoro-[1,1'-biphenyl]-2,2'-diol (**3e**)

**Chiral-SFC** (CHIRAL ART SJ ($CO_2$:MeOH 90:10, 2.5 mL min$^{-1}$, 40 °C) $t_R$ = 12.12 (minor), 13.12 (major) minutes.

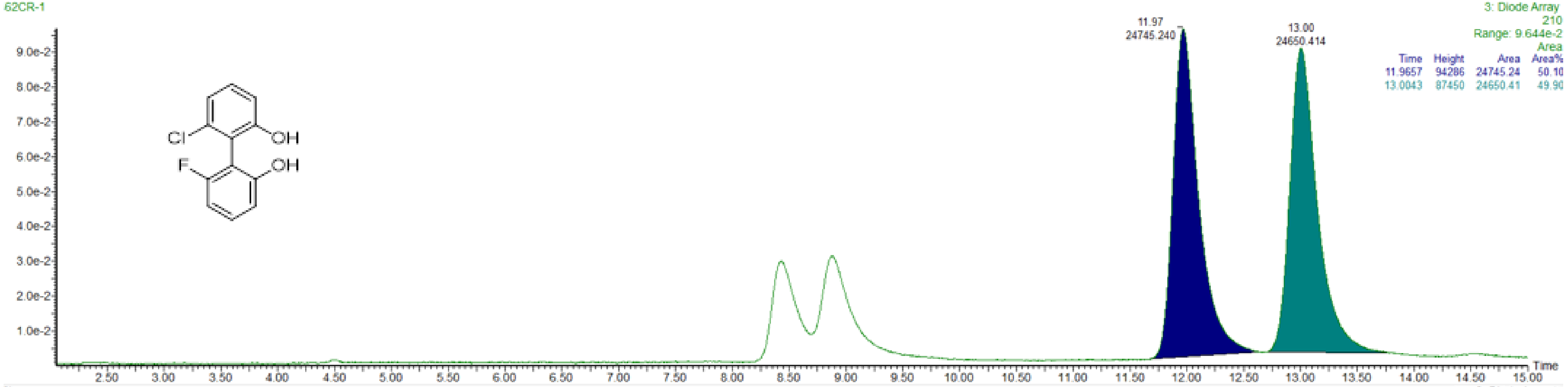


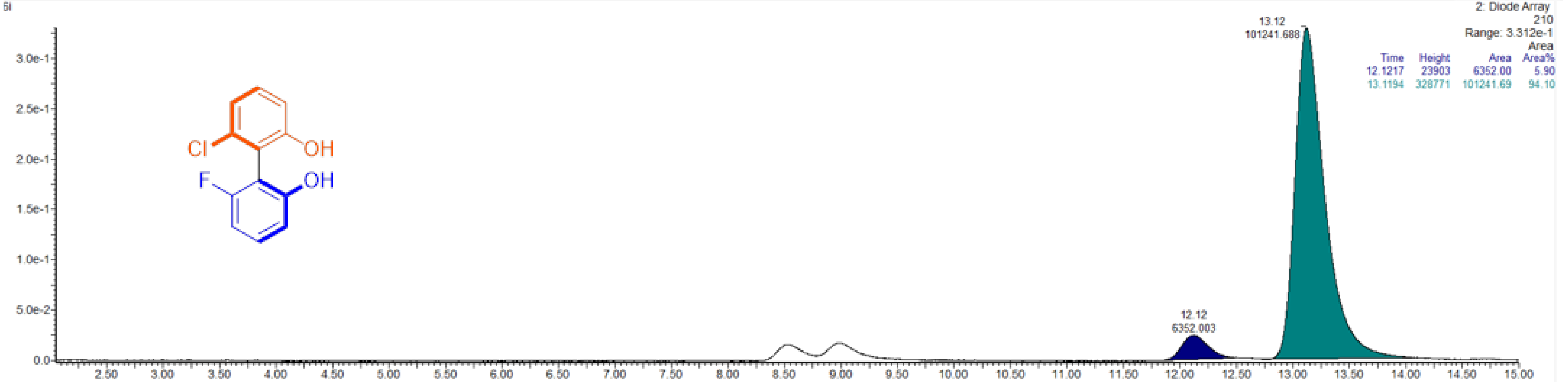

6-chloro-6'-fluoro-5-methyl-[1,1'-biphenyl]-2,2'-diol (**3f**)

**Chiral-SFC** (CHIRAL ART SJ ($CO_2$:MeOH 90:10, 2.5 mL min$^{-1}$, 40 °C) $t_R$ = 10.70 (minor), 11.71 (major) minutes.

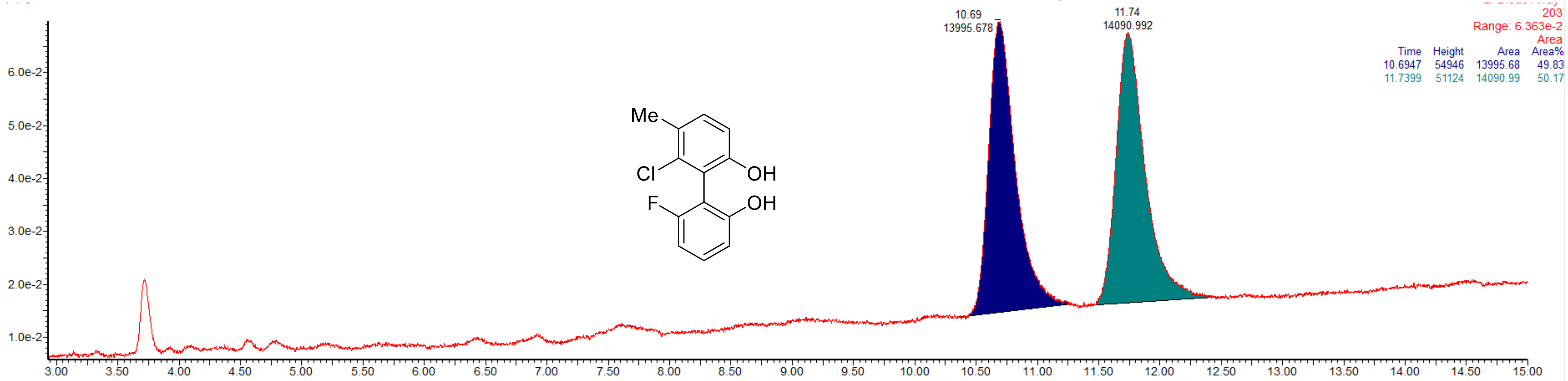


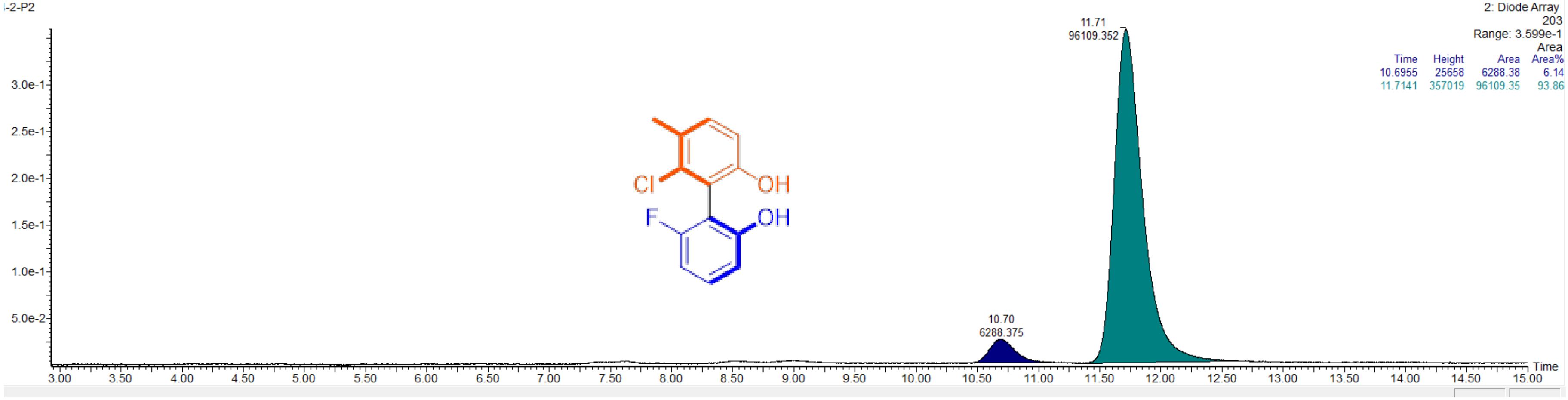

4,6-dichloro-6'-fluoro-[1,1'-biphenyl]-2,2'-diol (**3g**)

**Chiral-SFC** (CHIRAL PAK IG ($CO_2$:MeOH 90:10, 2.5 mL min$^{-1}$, 40 °C) $t_R$ = 7.24 (major), 9.25 (minor) minutes.

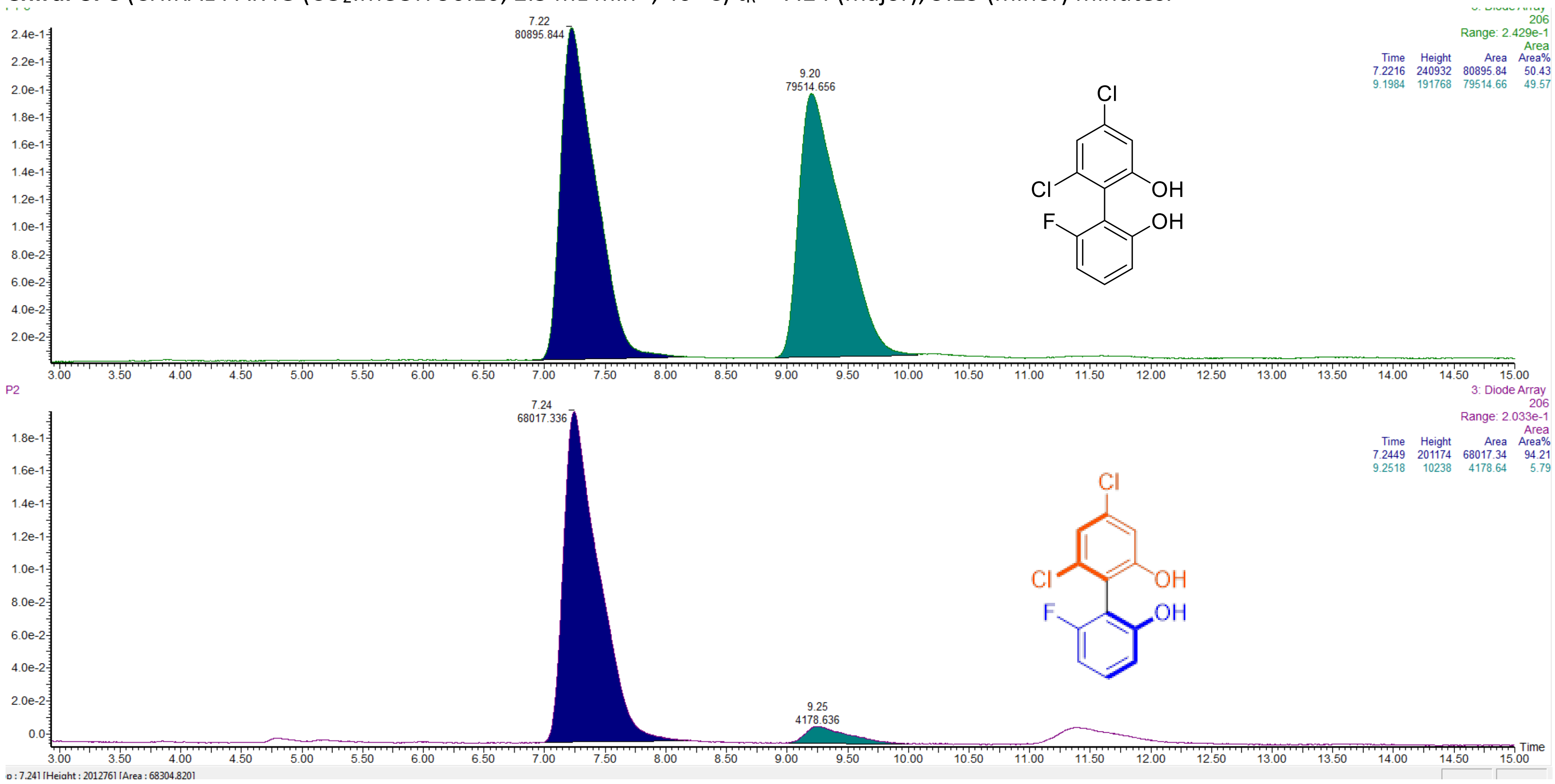

3,6-dichloro-6'-fluoro-[1,1'-biphenyl]-2,2'-diol (**3h**)

**Chiral-SFC** (CHIRAL ART SJ ($CO_2$:MeOH 85:15, 2.5 mL $min^{-1}$, 40 °C) $t_R$ = 7.55 (minor), 8.49 (major) minutes.

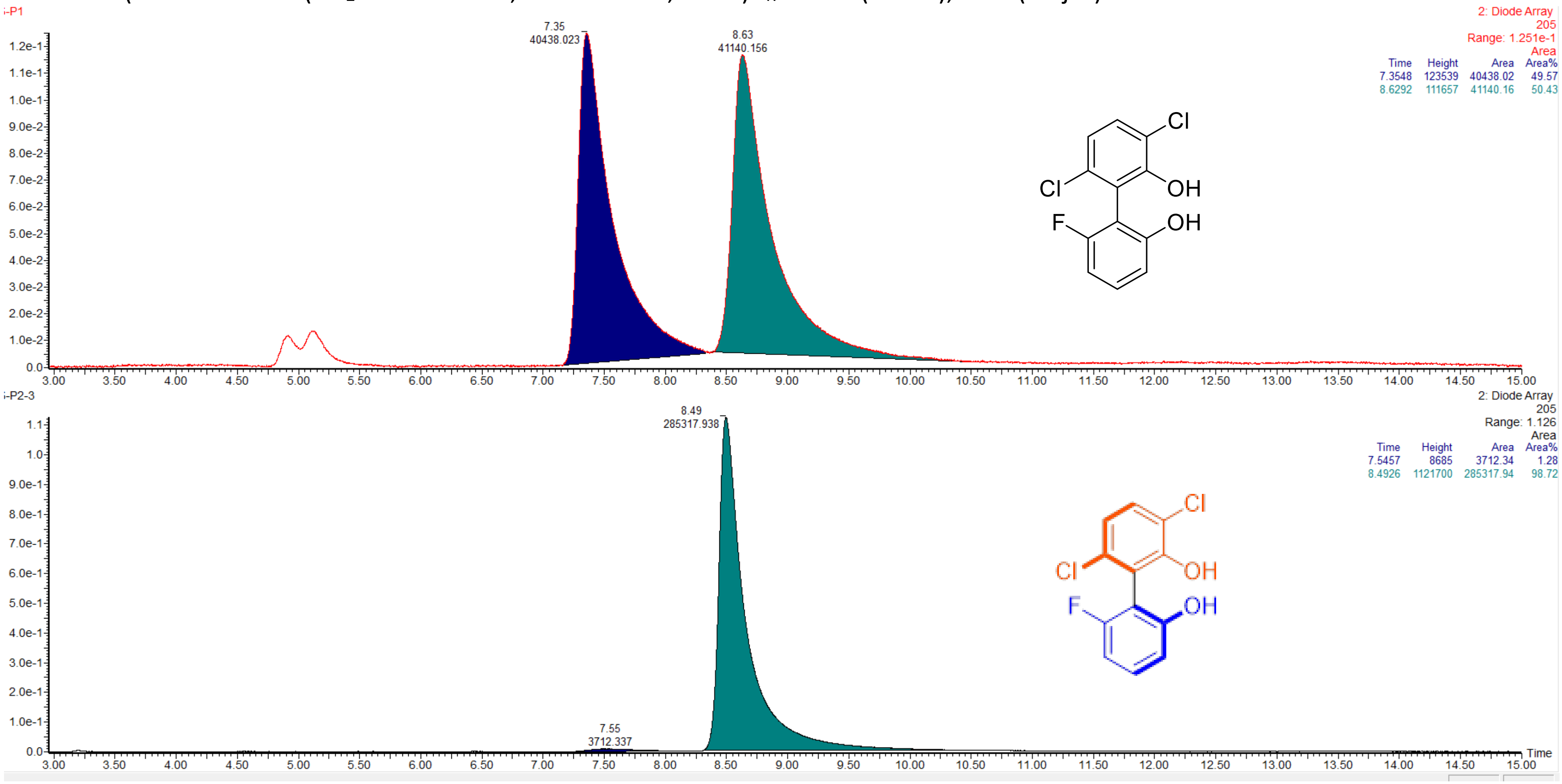

tert-butyl (6-chloro-2'-fluoro-2,6'-dihydroxy-[1,1'-biphenyl]-3-yl)carbamate (**3i**)

**Chiral-SFC** (CHIRAL ART IG ($CO_2$:MeOH 90:10, 2.5 mL min$^{-1}$, 40 °C) $t_R$ = 6.84 (minor), 13.49 (major) minutes.

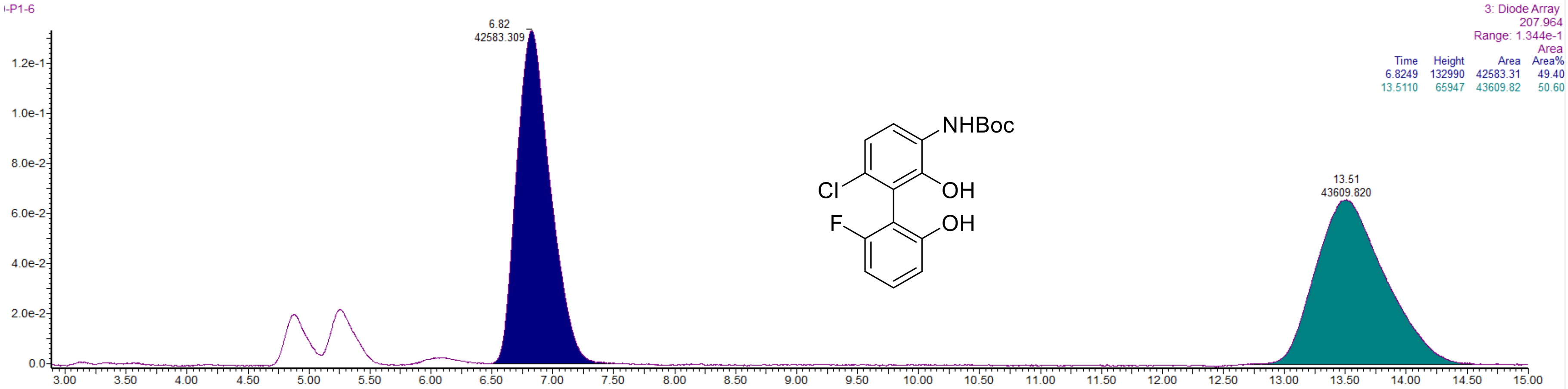


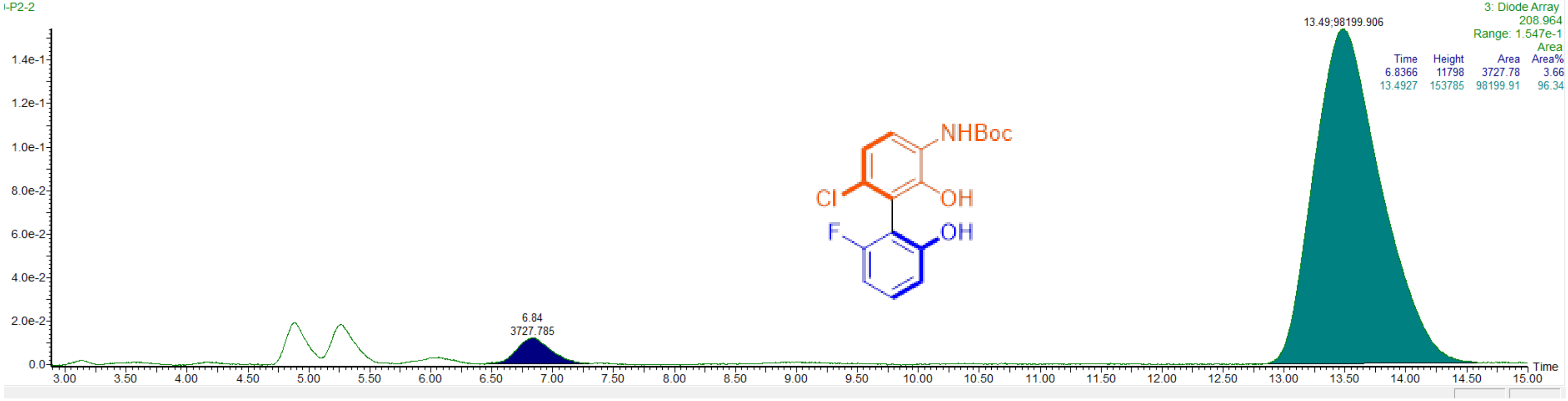

6-chloro-6'-fluoro-3-methoxy-[1,1'-biphenyl]-2,2'-diol (**3j**)

**Chiral-SFC** (CHIRAL ART SJ ($CO_2$:MeOH 85:15, 2.5 mL $min^{-1}$, 40 °C) $t_R$ = 9.91 (minor), 13.40 (major) minutes.

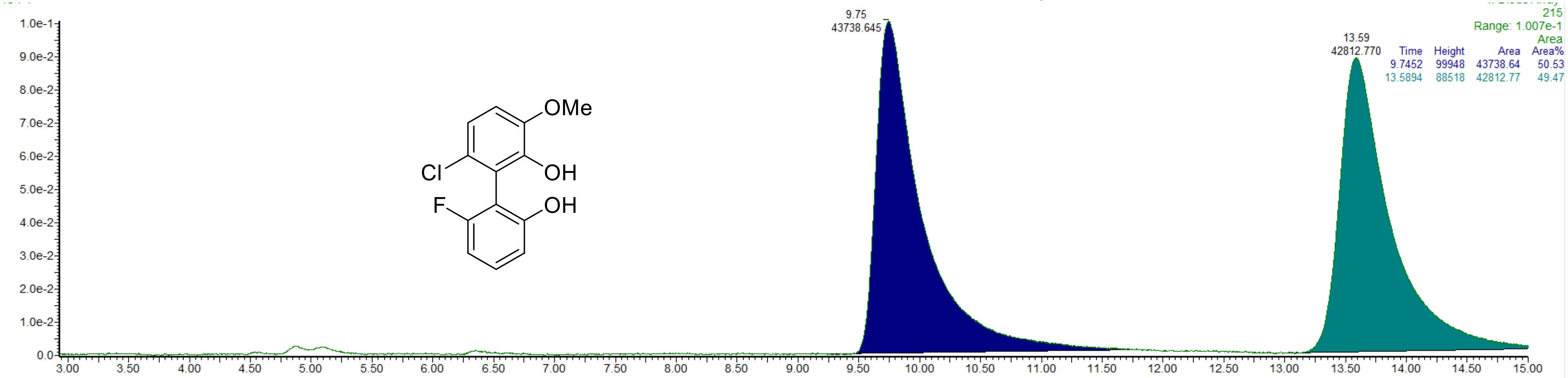


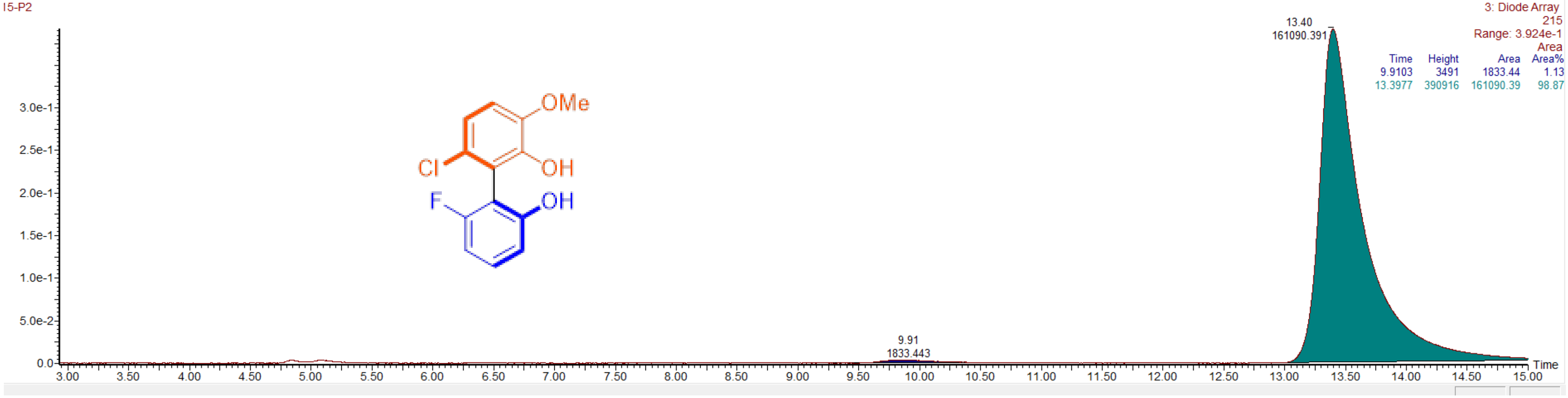

6-fluoro-6'-(2-nitroethyl)-[1,1'-biphenyl]-2,2'-diol (**3k**)

**Chiral-SFC** (CHIRAL PAK IE ($CO_2$:MeOH 95:5, 2.5 mL min$^{-1}$, 40 °C) $t_R$ = 10.85 (major), 11.91 (minor) minutes.

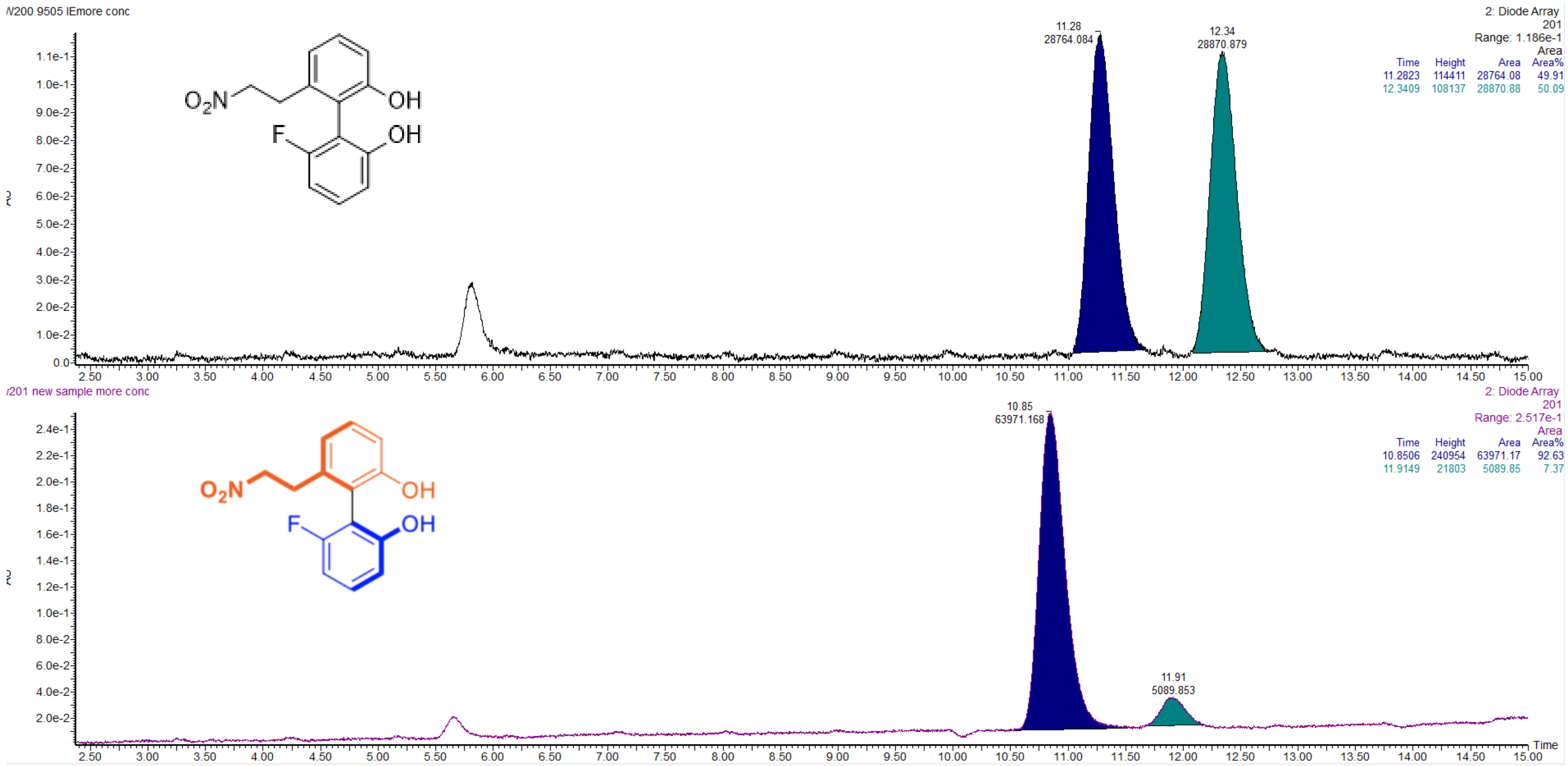

6-(((*tert*-butyldimethylsilyl)oxy)methyl)-6'-fluoro-3-methoxy-[1,1'-biphenyl]-2,2'-diol (**3l**)

**Chiral-SFC** (CHIRAL PAK IE ($CO_2$:MeOH, 97:3, 2.5 mL min$^{-1}$, 40 °C) $t_R$ = 7.02 (major) 9.11 (minor) minutes.

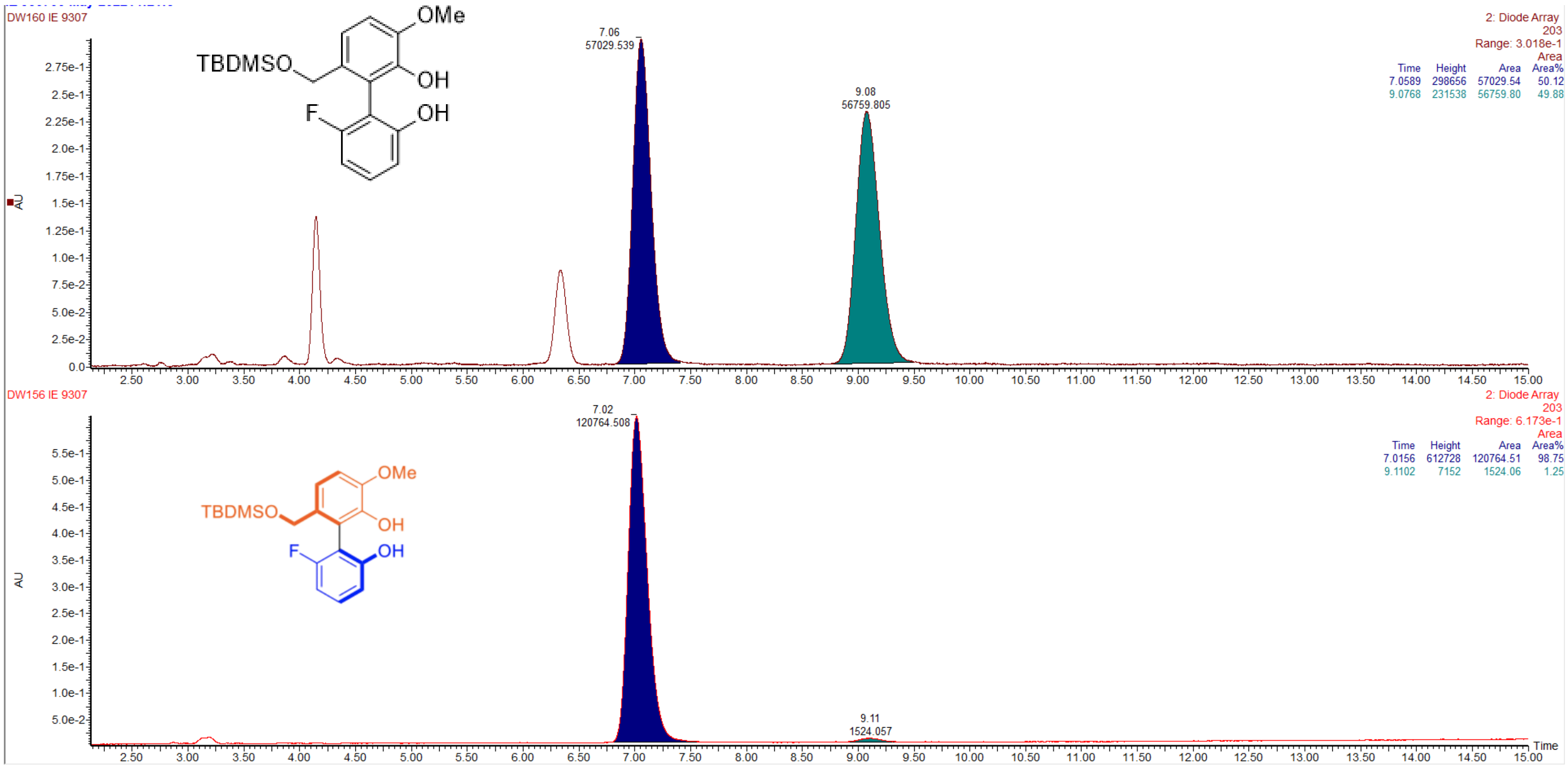

<u>*tert*-butyl ((2'-fluoro-6,6'-dihydroxy-5-methoxy-[1,1'-biphenyl]-2-yl)methyl)carbamate (**3m**)</u>

**Chiral-SFC** (CHIRAL ART SB ($CO_2$:MeOH, 90:10, 2.5 mL min$^{-1}$, 40 °C) $t_R$ = 7.88 (major), 12.46 (minor) minutes.

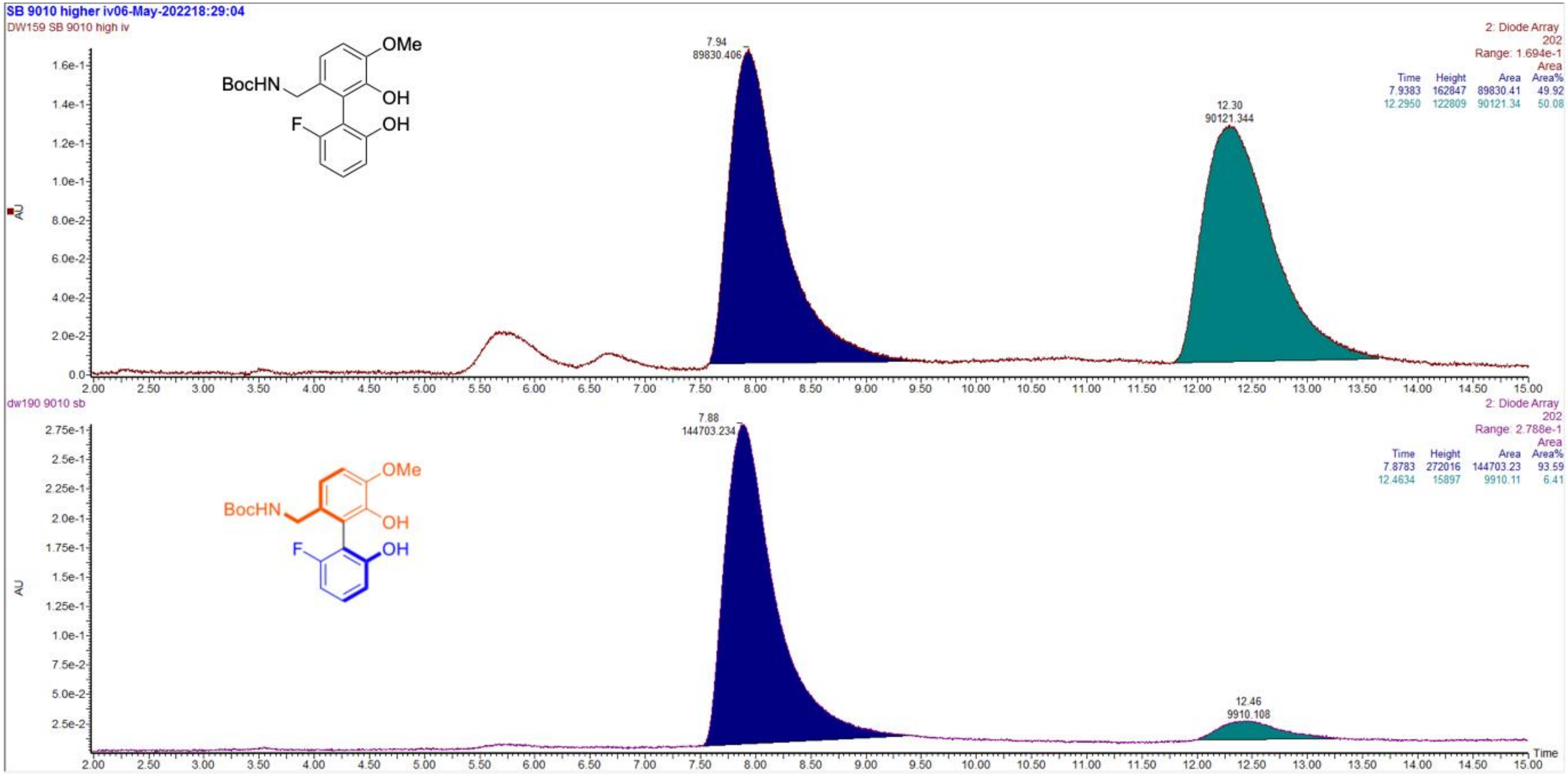

6-chloro-3-(2,4-dichlorophenoxy)-6'-fluoro-[1,1'-biphenyl]-2,2'-diol (**3n**)

**Chiral-SFC** (CHIRAL ART SJ ($CO_2$:MeOH, 80:20, 2.5 mL min$^{-1}$, 40 °C) $t_R$ = 7.80 (major), 8.89 (minor) minutes.

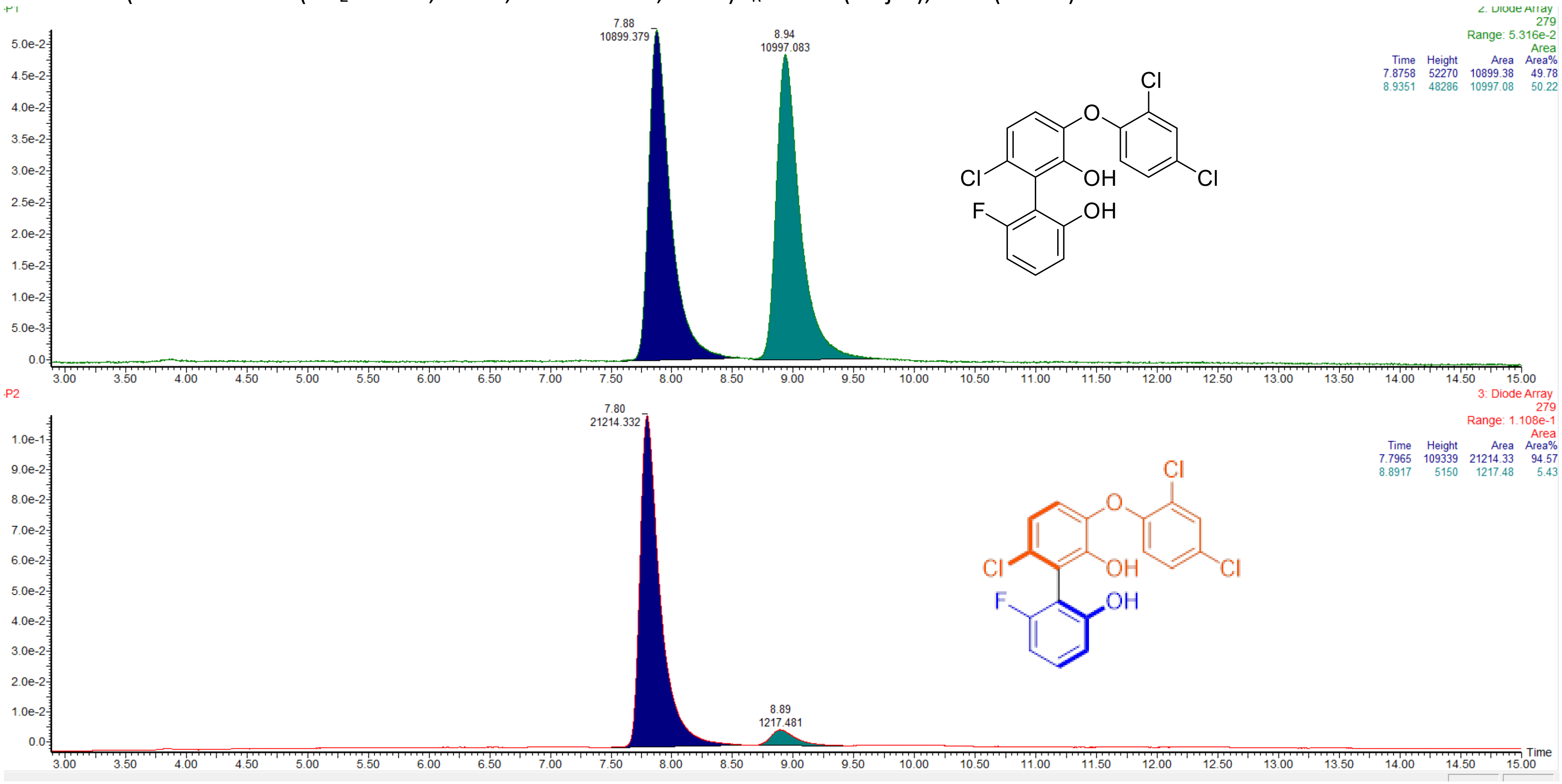

(8*R*,9*S*,13*S*,14*S*)-4-(2-fluoro-6-hydroxyphenyl)-3-hydroxy-13-methyl-6,7,8,9,11,12,13,14,15,16-decahydro-17H-cyclopenta[a]phenanthren-17-one (**3o**)

**Chiral-SFC** (CHIRAL ART SJ ($CO_2$:MeOH, 85:15, 2.5 mL min$^{-1}$, 40 °C) $t_R$ = 12.34 (major), 13.83 (minor) minutes.

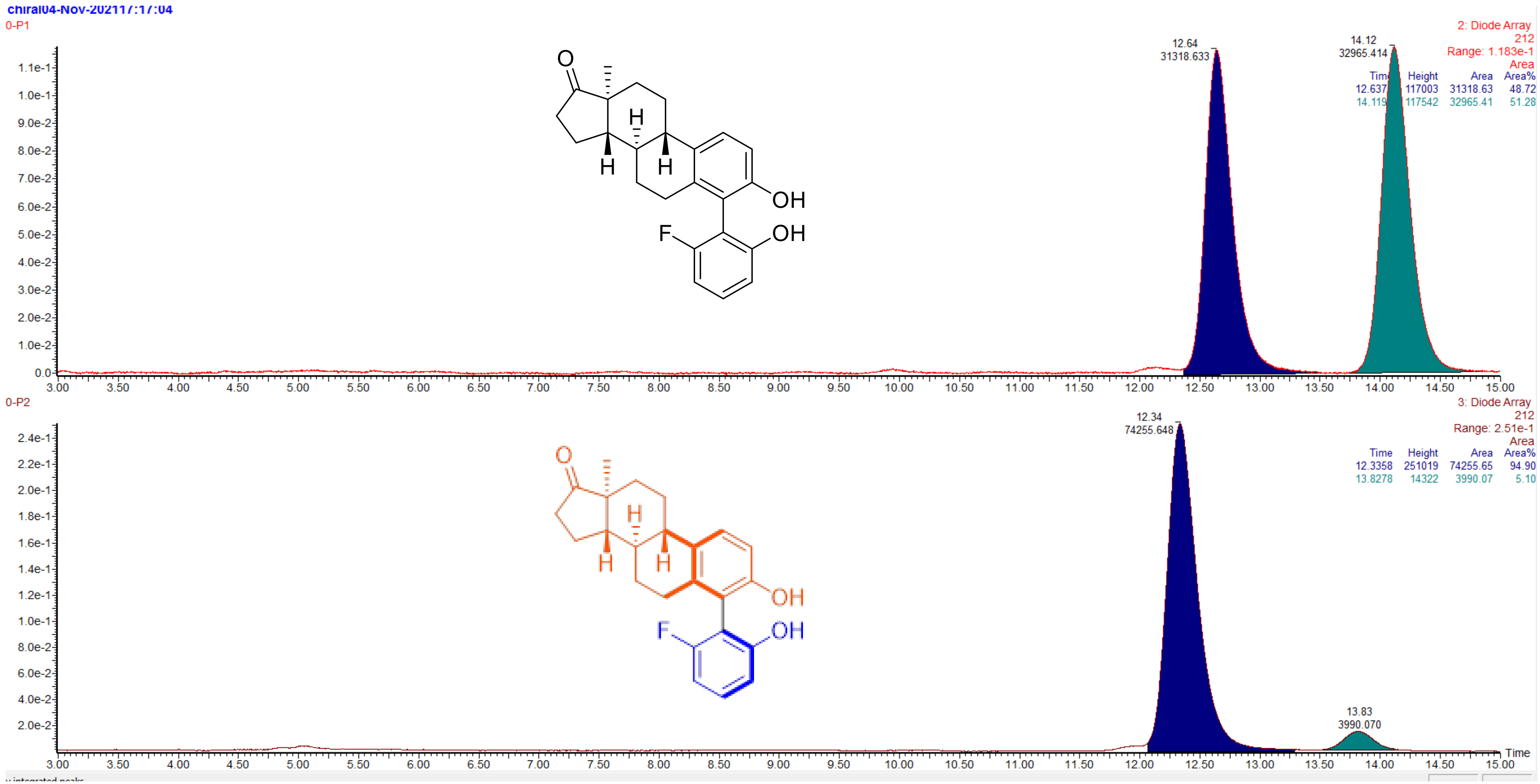

6-chloro-6'-methyl-[1,1'-biphenyl]-2,2'-diol (**3p**)

**Chiral-SFC** (CHIRAL PAK IG ($CO_2$:MeOH 90:10, 2.5 mL min$^{-1}$, 40 °C) $t_R$ = 7.18 (major), 7.92 (minor) minutes.

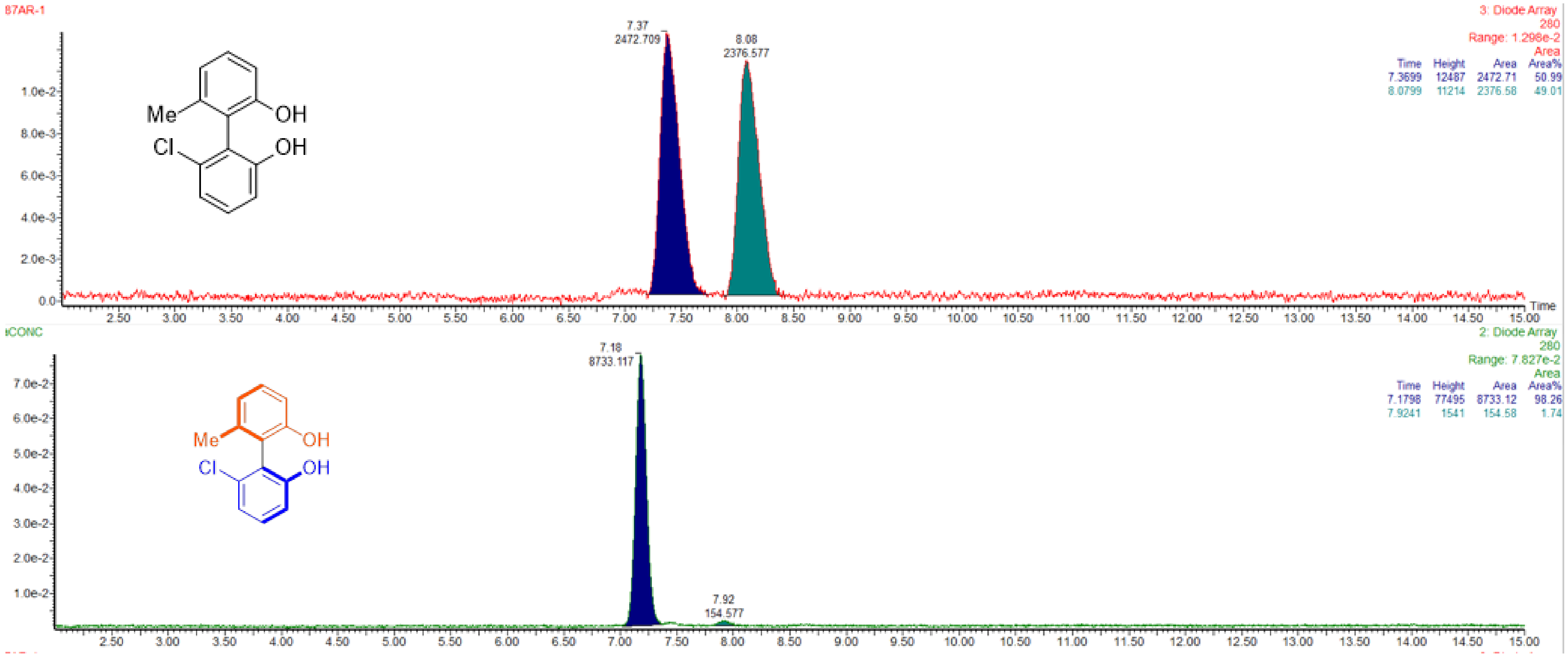

6-chloro-6'-methoxy-[1,1'-biphenyl]-2,2'-diol (**3q**)

**Chiral-SFC** (CHIRAL PAK IG ($CO_2$:MeOH 90:10, 2.5 mL min$^{-1}$, 40 °C) $t_R$ = 9.16 (major), 10.62 (minor) minutes.

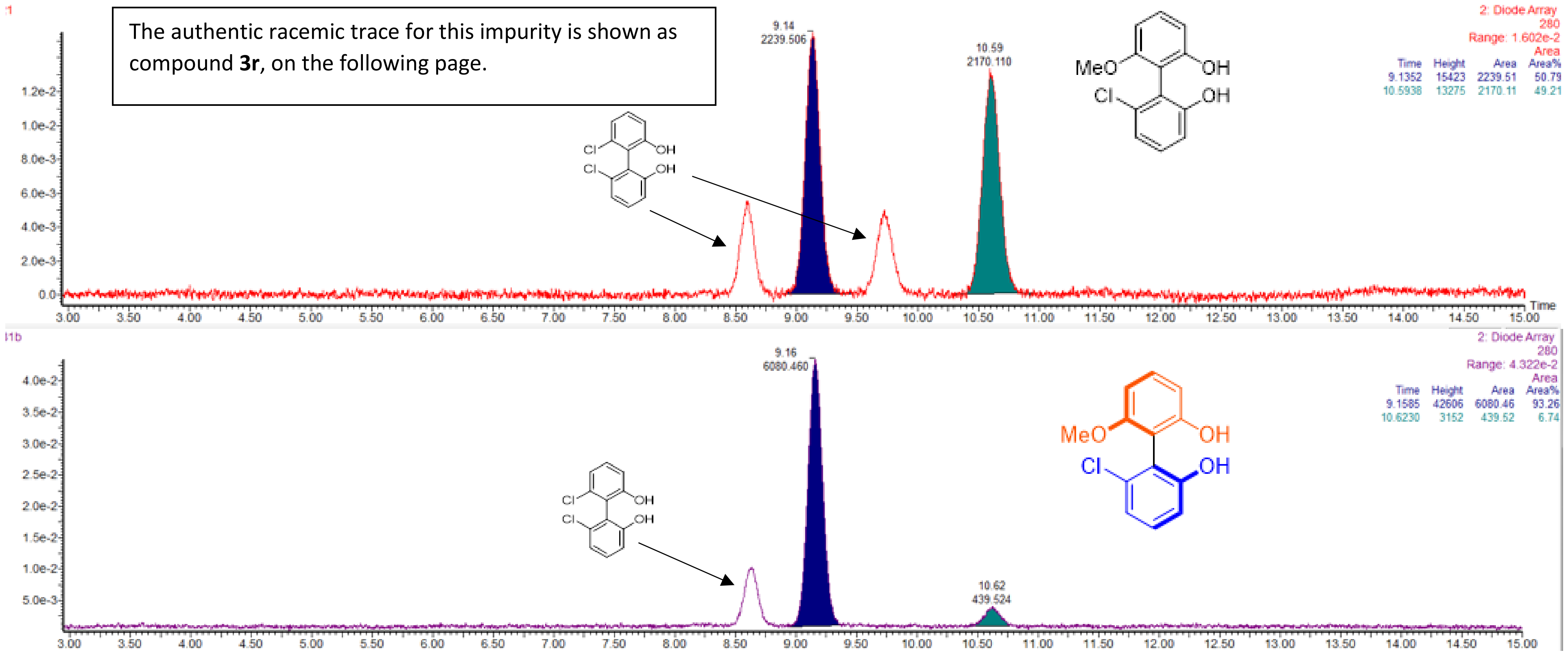


Product contains a 23 mol% impurity of 6,6'-dichloro-[1,1'-biphenyl]-2,2'-diol which was inseparable *via* column chromatography.

6,6'-dichloro-[1,1'-biphenyl]-2,2'-diol (**3r**)

**Chiral-SFC** (CHIRAL PAK IG ($CO_2$:MeOH 90:10, 2.5 mL min$^{-1}$, 40 °C) $t_R$ = 8.62 (major), 9.80 (minor) minutes.

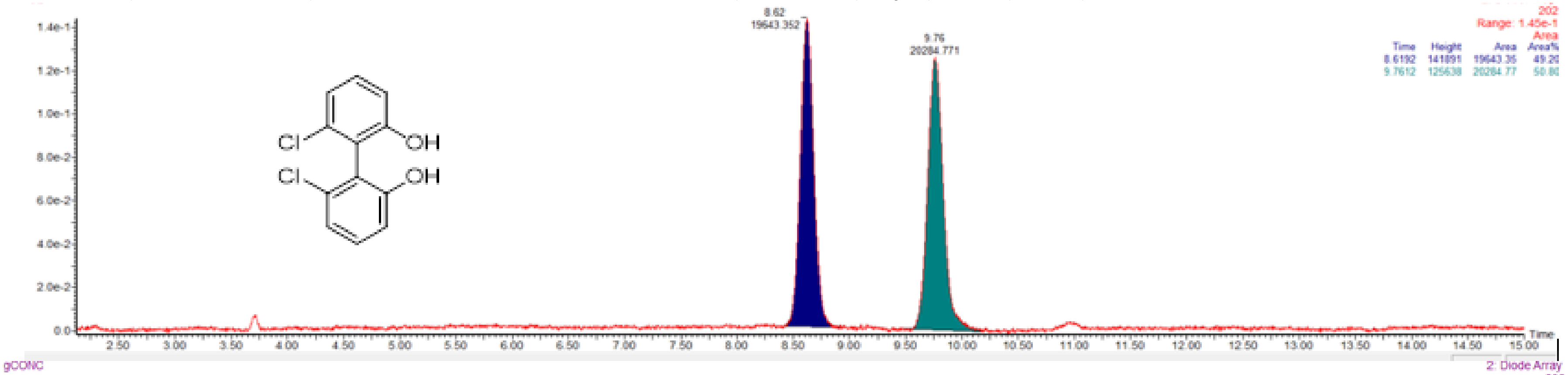


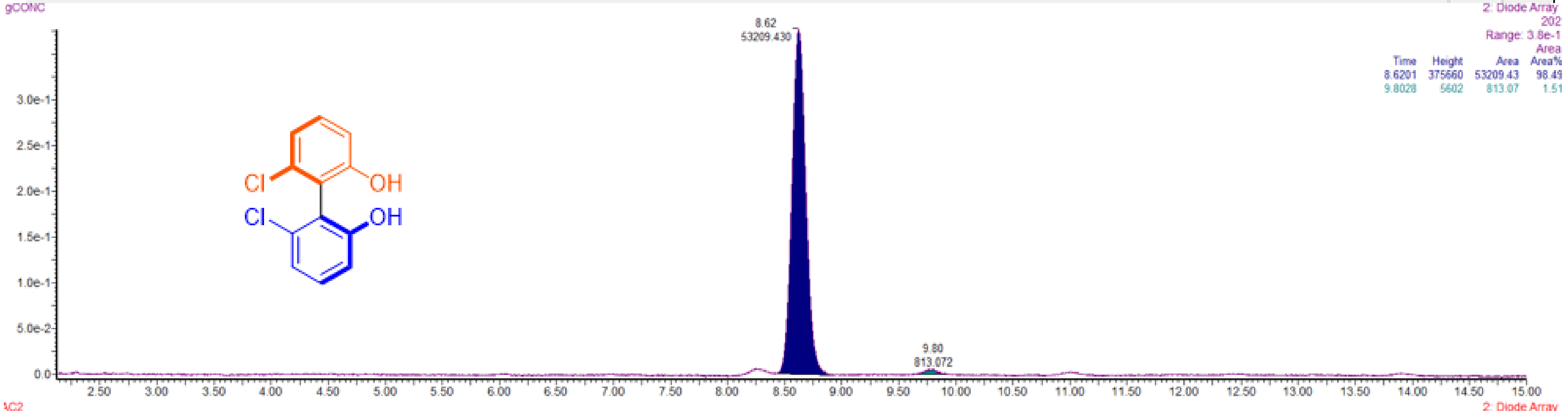

6,6'-dichloro-[1,1':3',1''-terphenyl]-2,2'-diol (**3s**)

**Chiral-SFC** (CHIRAL ART SJ ($CO_2$:MeOH 80:20, 2.5 mL min$^{-1}$, 40 °C) $t_R$ = 9.84 (major), 12.00 (minor) minutes.

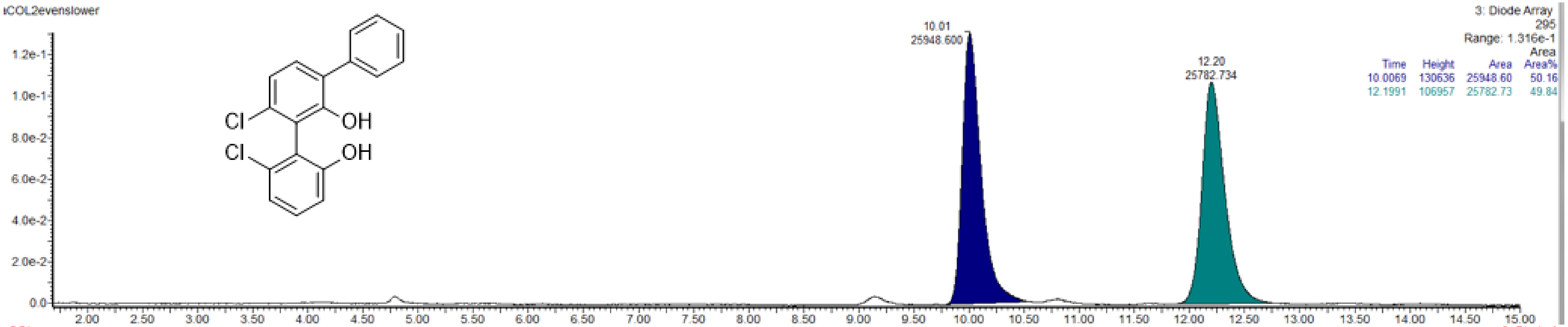


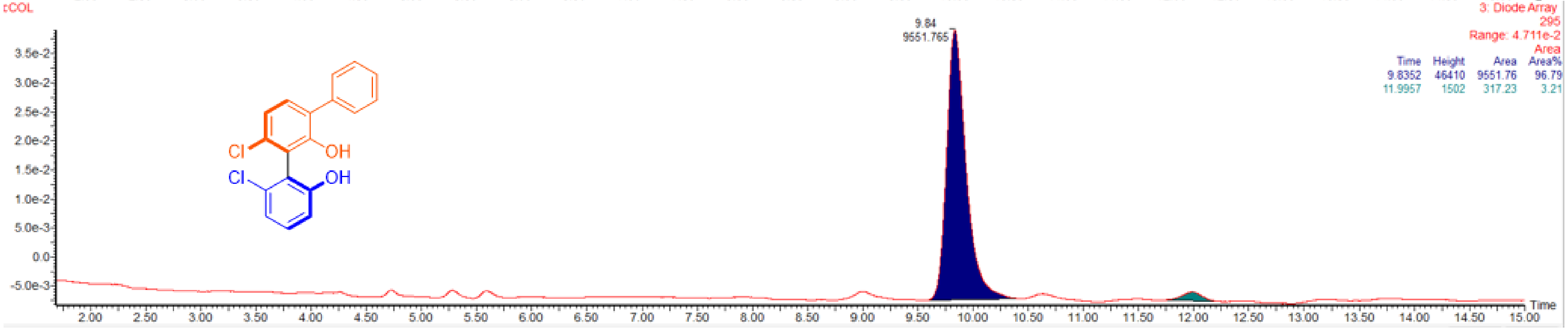

6,6'-dichloro-3-methyl-[1,1'-biphenyl]-2,2'-diol (**3t**)

**Chiral-SFC** (CHIRAL PAK IG ($CO_2$:MeOH 90:10, 2.5 mL min$^{-1}$, 40 °C) $t_R$ = 7.31 (major), 8.65 (minor) minutes.

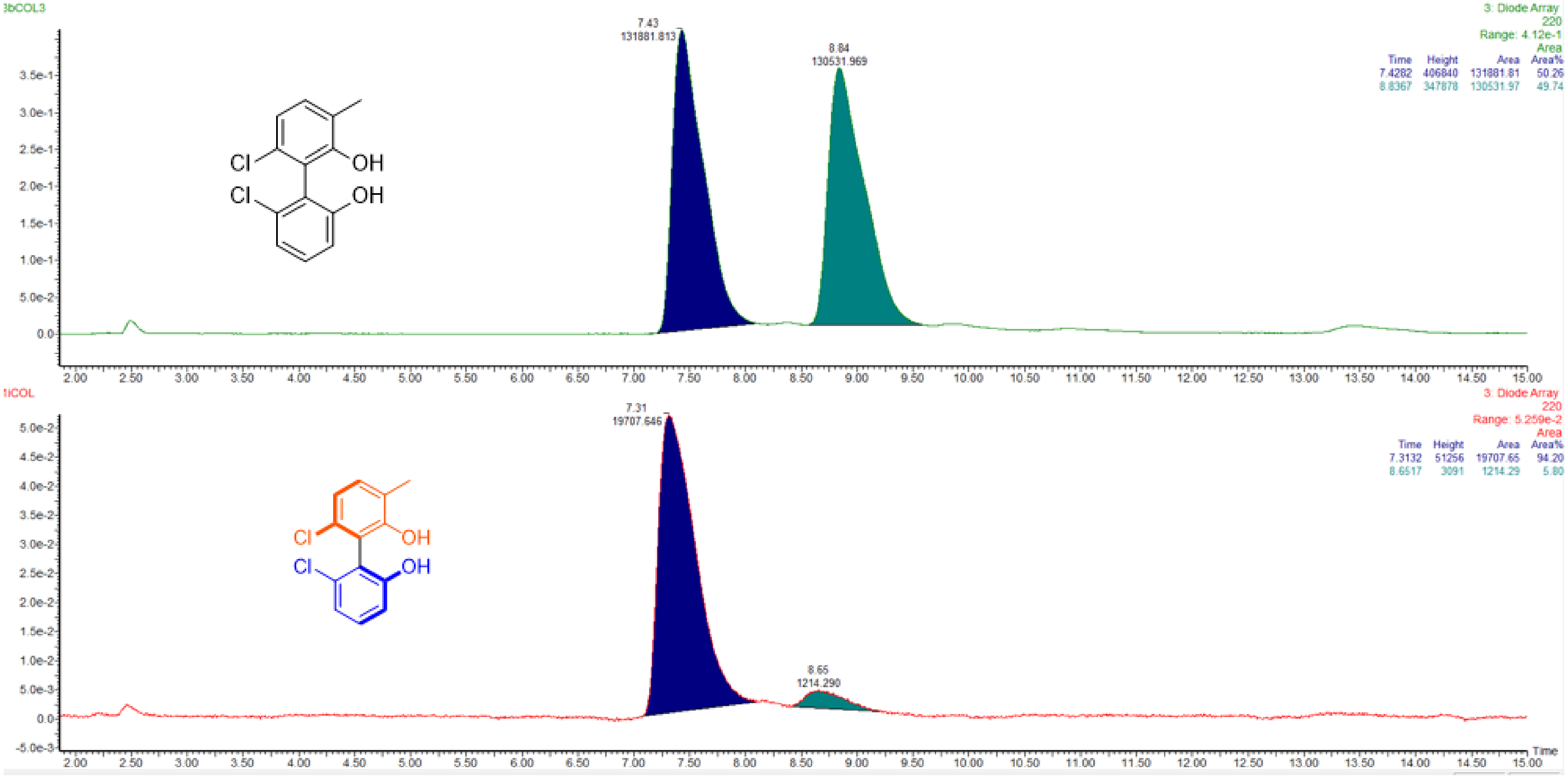

6-chloro-6'-(trifluoromethoxy)-[1,1'-biphenyl]-2,2'-diol (**3u**)

**Chiral-SFC** (CHIRAL PAK IG ($CO_2$:MeOH 95:5, 2.5 mL min$^{-1}$, 40 °C) $t_R$ = 6.44 (major), 8.89 (minor) minutes.

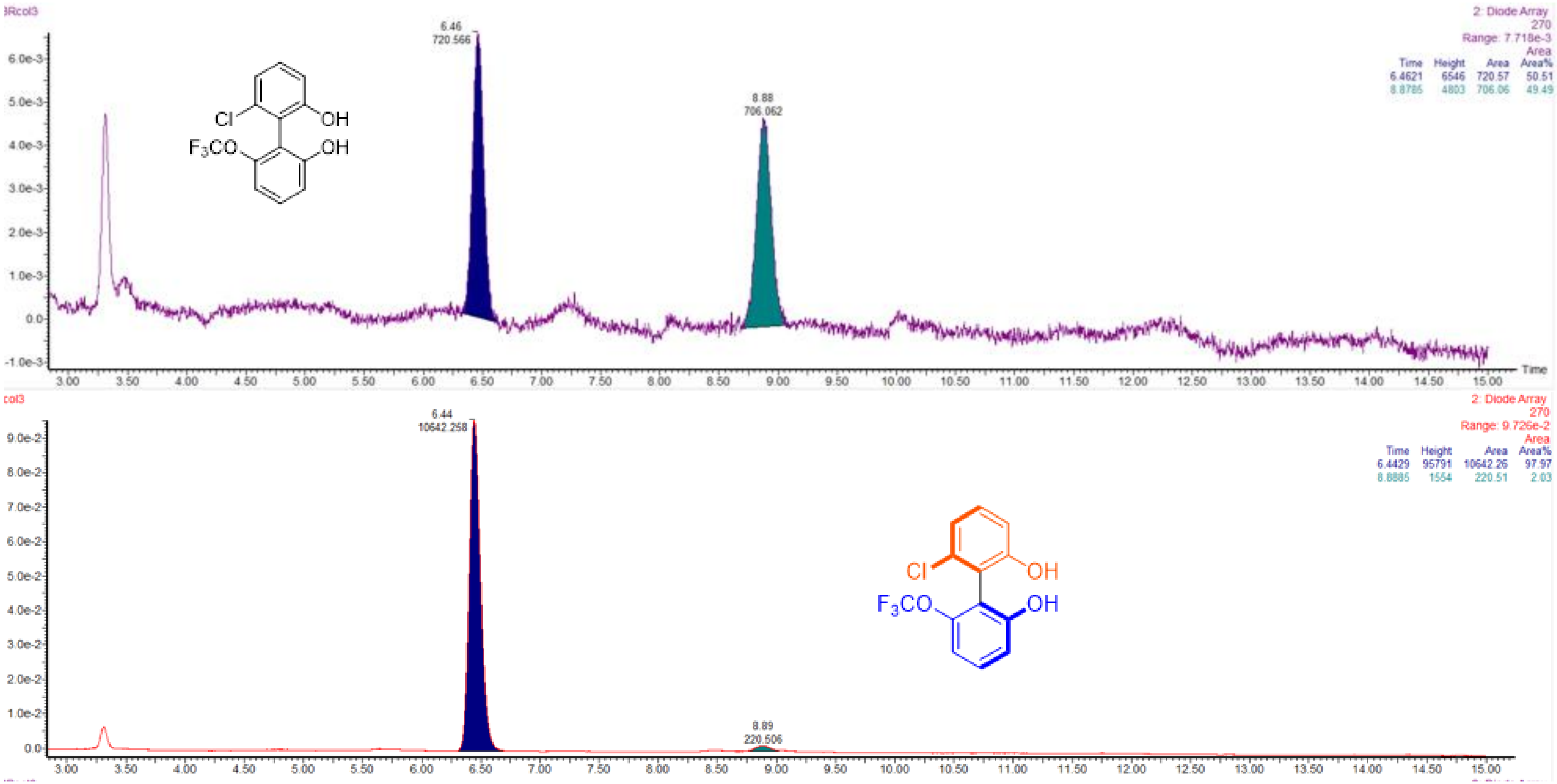

6-methyl-6'-(trifluoromethoxy)-[1,1'-biphenyl]-2,2'-diol (**3v**)

**Chiral-SFC** (CHIRAL ART SJ ($CO_2$:MeOH 96:4, 2.5 mL $min^{-1}$, 40 °C) $t_R$ = 6.15 (major), 6.76 (minor) minutes.

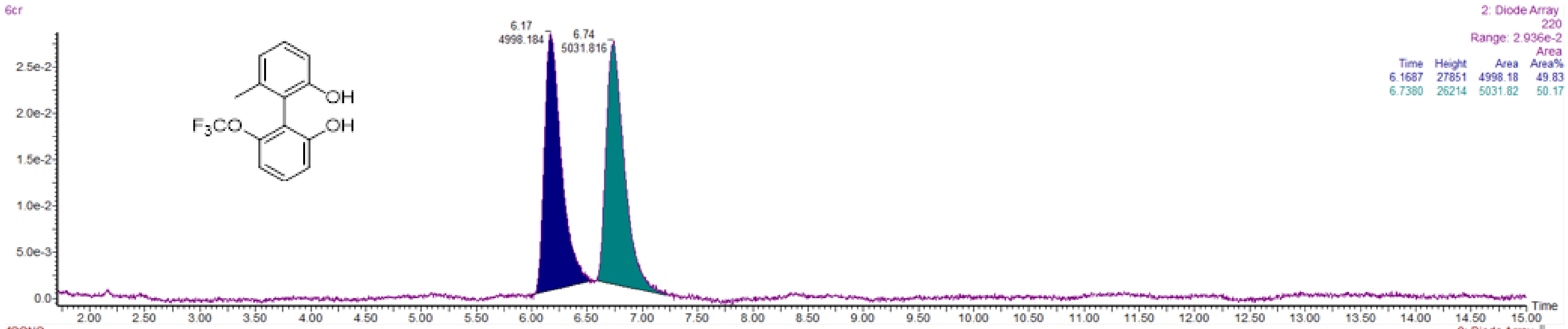


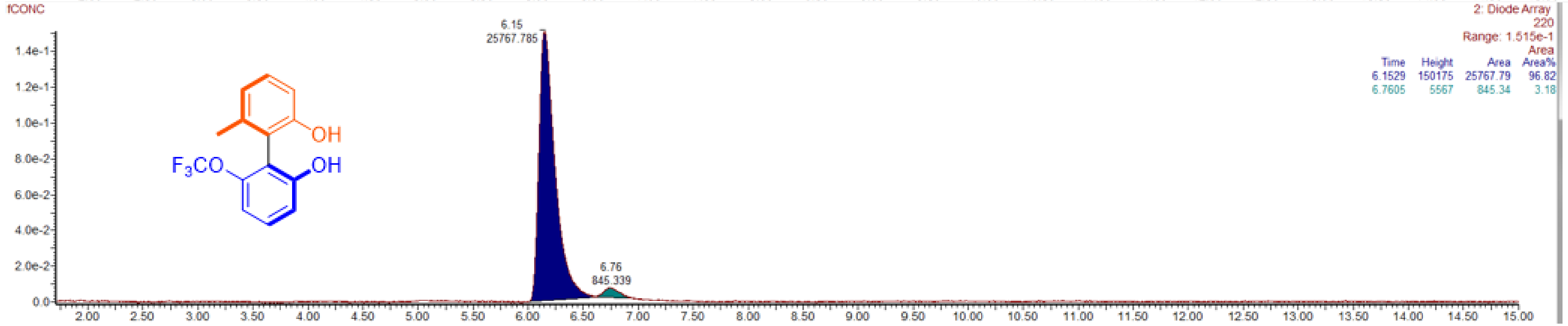

Ethyl (*R*,*E*)-3-(2'-fluoro-6,6'-dihydroxy-[1,1'-biphenyl]-2-yl)acrylate (**3w**)

**Chiral-SFC** (ChiralART SB($CO_2$:MeOH 85:15, 2.5 mLmin$^{-1}$, 40 °C) $t_R$ = 4.70 (major), 5.66 (minor) minutes.

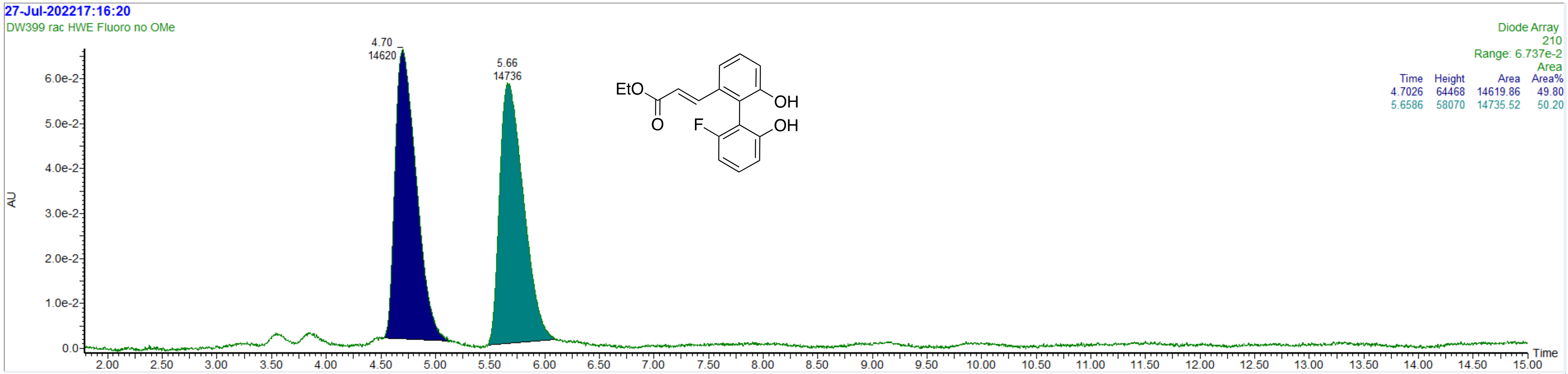


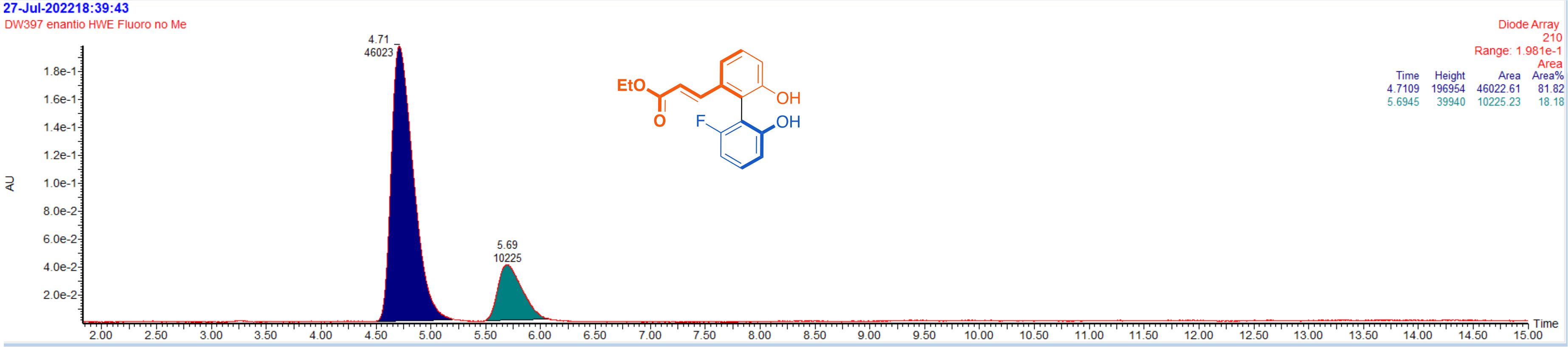

6-fluoro-2'-methoxy-6'-methyl-[1,1'-biphenyl]-2-ol (**6a**)

**Chiral-SFC** (CHIRAL PAK IG ($CO_2$:MeOH 98:2, 2.5 mL min$^{-1}$, 40 °C) $t_R$ = 5.90 (major), 6.62 (minor) minutes.

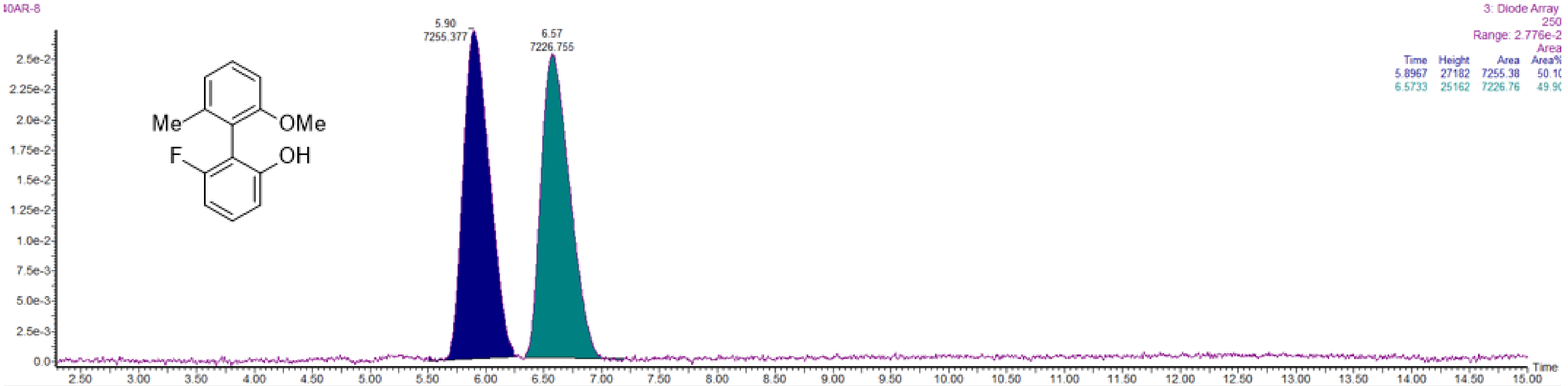


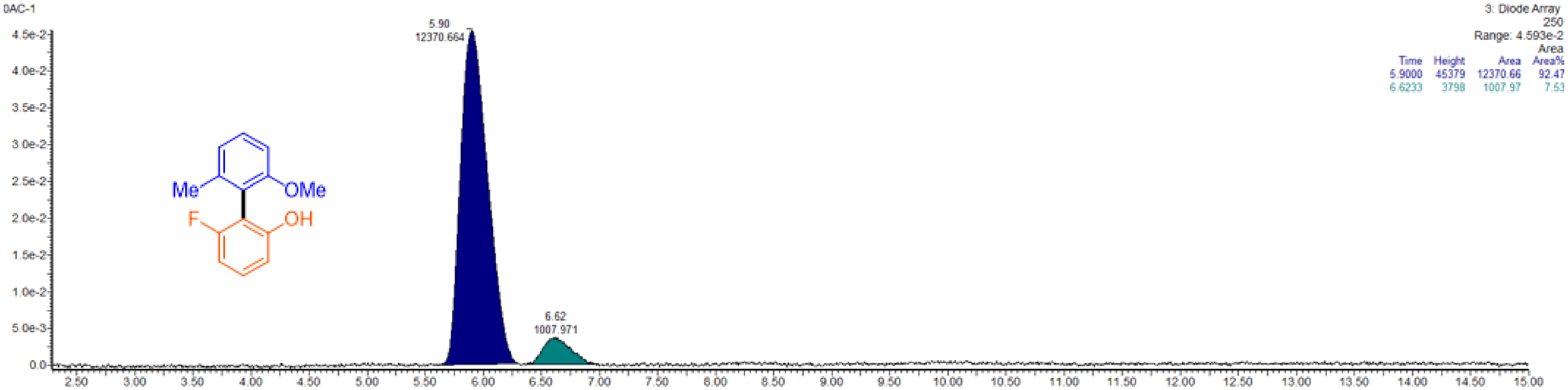

2'-fluoro-6'-methoxy-6-methyl-[1,1'-biphenyl]-2-ol (**6b**)

**Chiral-SFC** (CHIRAL ART SJ ($CO_2$:MeOH 94:6, 2.5 mL min$^{-1}$, 40 °C) $t_R$ = 5.43 (minor), 6.05 (major) minutes.

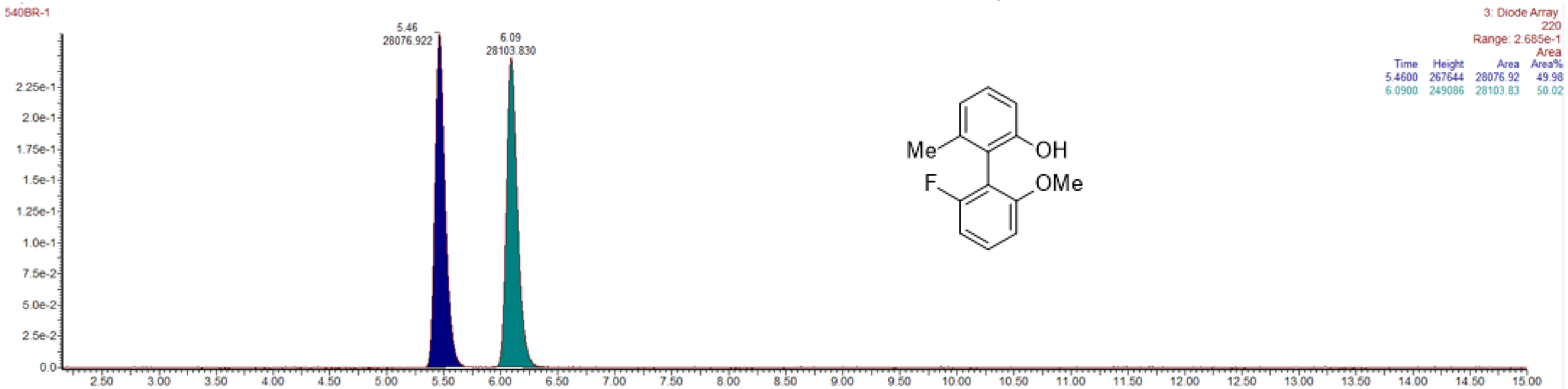


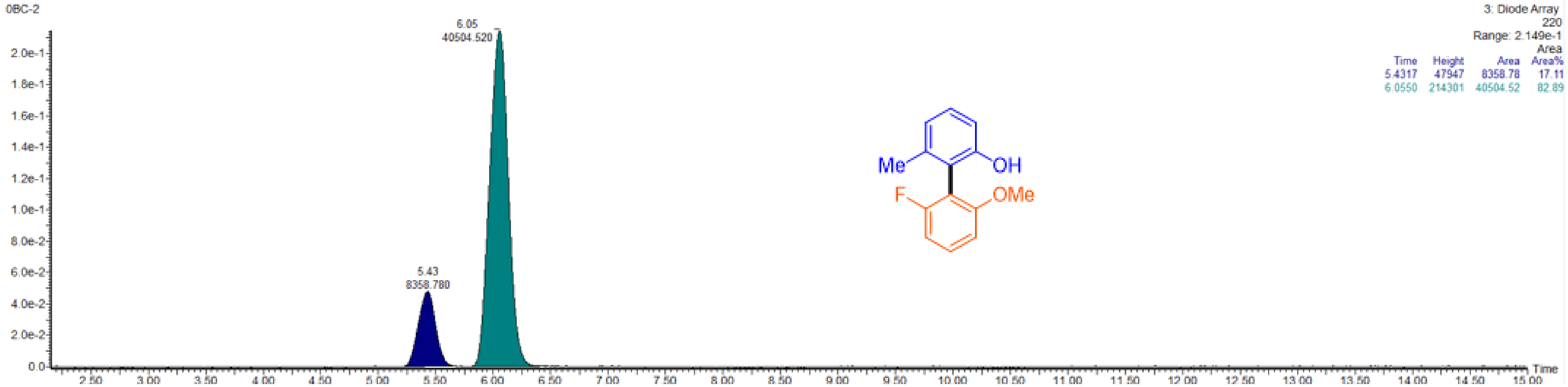

2-fluoro-2',6-dimethoxy-6'-methyl-1,1'-biphenyl (**6c**)

**Chiral-SFC** (CHIRAL ART SJ ($CO_2$:MeOH 99:1, 2.5 mL $min^{-1}$, 40 °C) $t_R$ = 3.76 (minor), 4.02 (major) minutes.

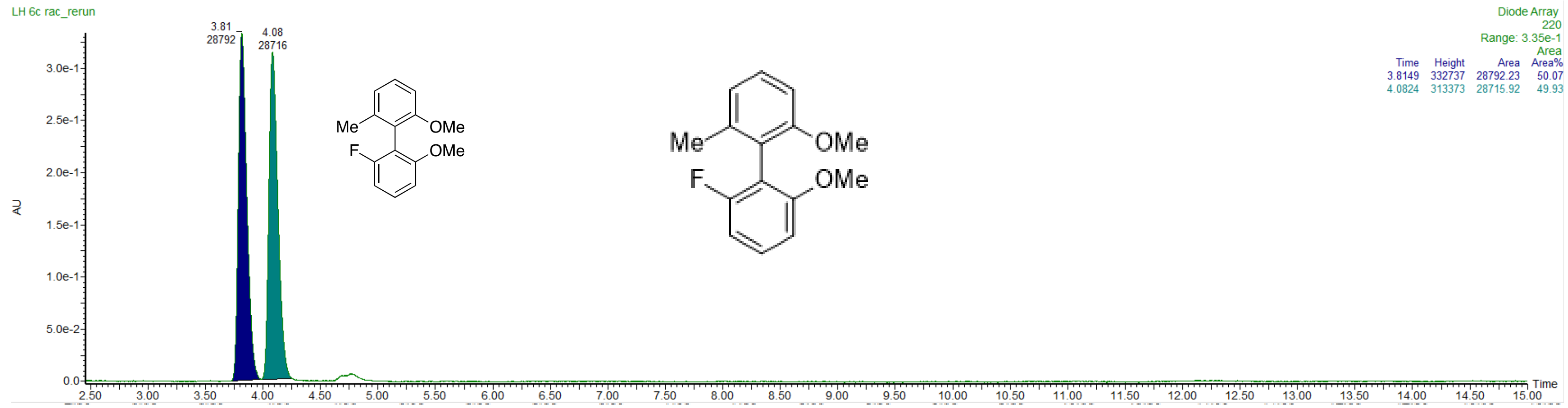


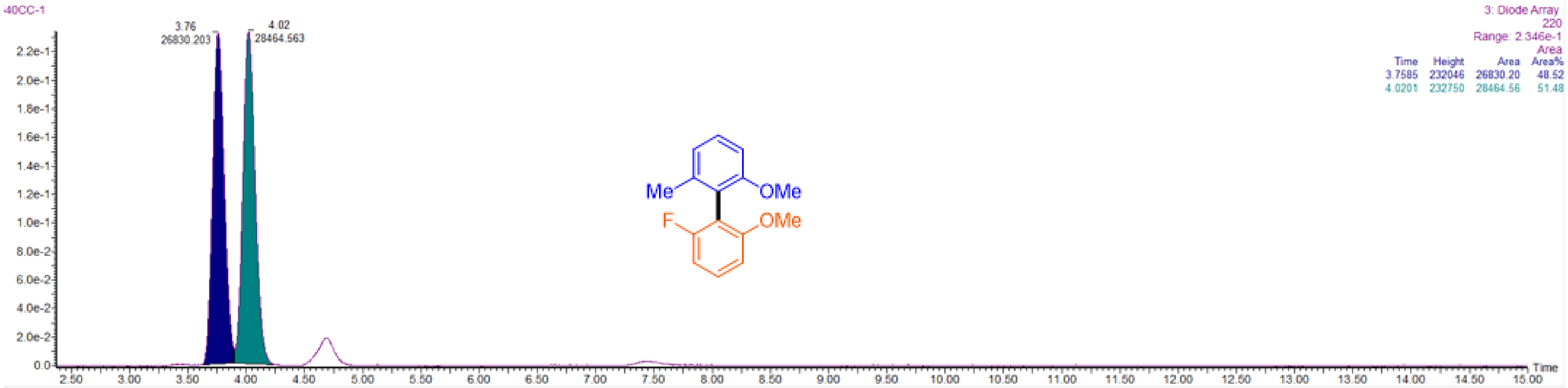

6,6''-difluoro-4',6'-dichloro-[1,1':3',1''-terphenyl]-2,2',2''-triol (**5b**)

**Chiral-SFC** (CHIRAL PAK IE ( $CO_2$:MeOH 90:10, 2.5 mL $min^{-1}$, 40 °C) $t_R$ = 6.13 (major), 7.58 (minor) minutes.

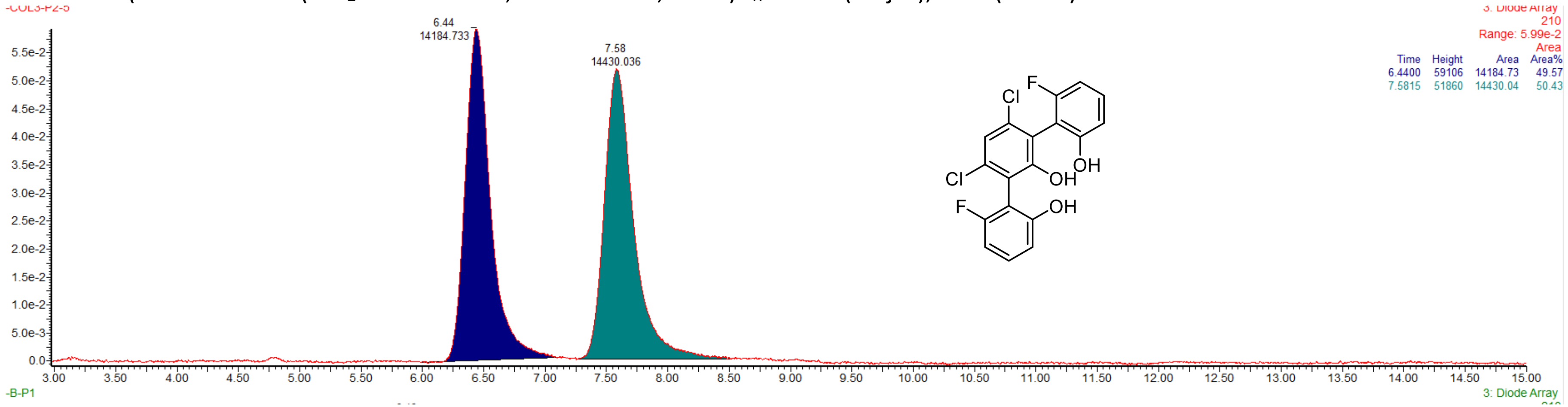


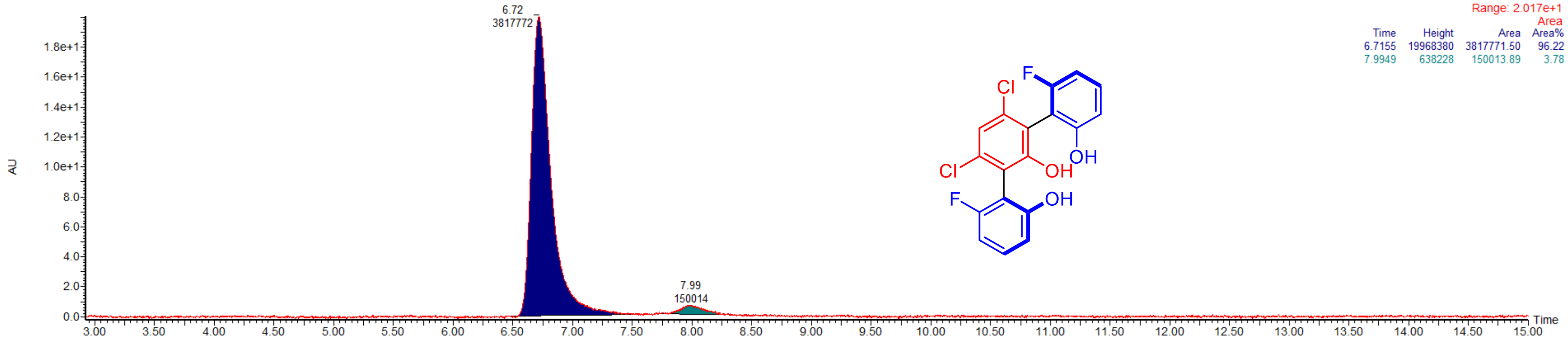

6,6''-difluoro-4',6'-dimethyl-[1,1':3',1''-terphenyl]-2,2',2''-triol (**5d**)

**Chiral-SFC** (CHIRAL ART SJ ($CO_2$:MeOH 90:10, 2.5 mL min$^{-1}$, 40 °C) $t_R$ = 6.77 (minor), 7.66 (major) minutes.

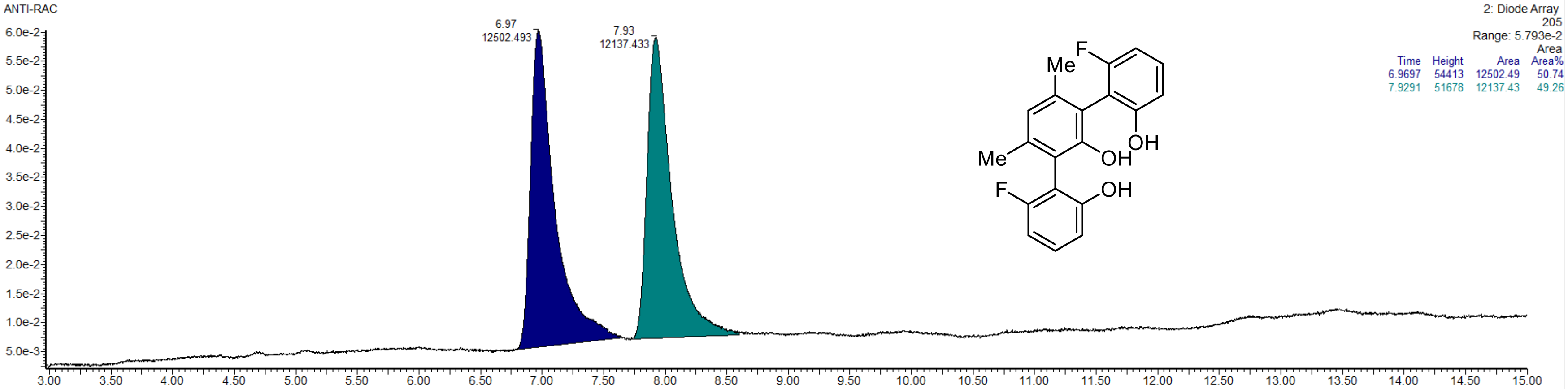


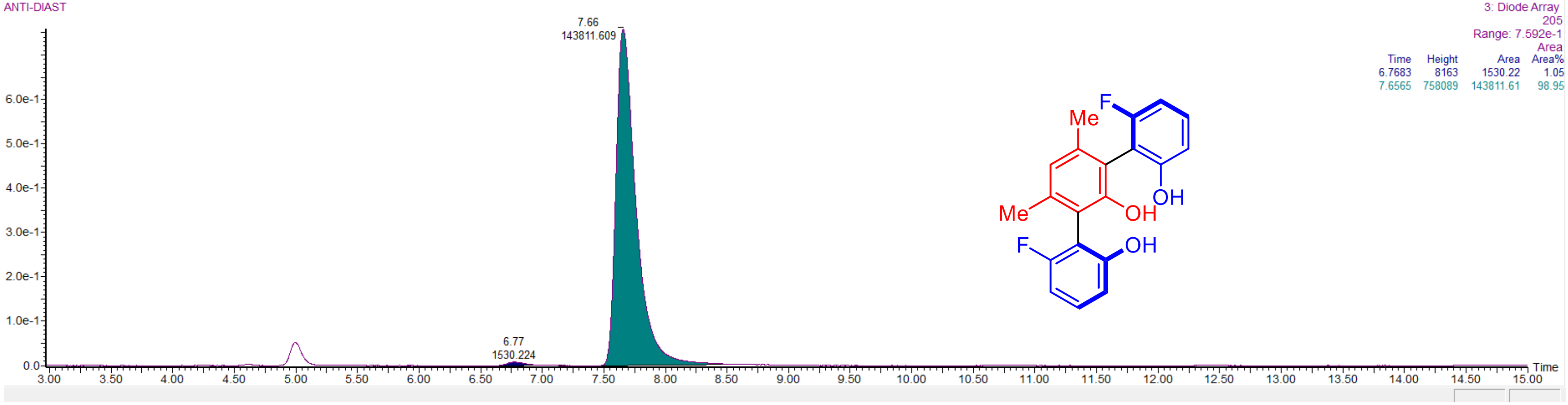

*N*-(2'-fluoro-6'-hydroxy-6-methyl-[1,1'-biphenyl]-2-yl)acetamide (**7**)

**Chiral-SFC** (ChiralPAK IE($CO_2$:MeOH 95:05, 2.5 mLmin$^{-1}$, 40 °C) $t_R$ = 10.85 (major), 11.98 (minor) minutes.

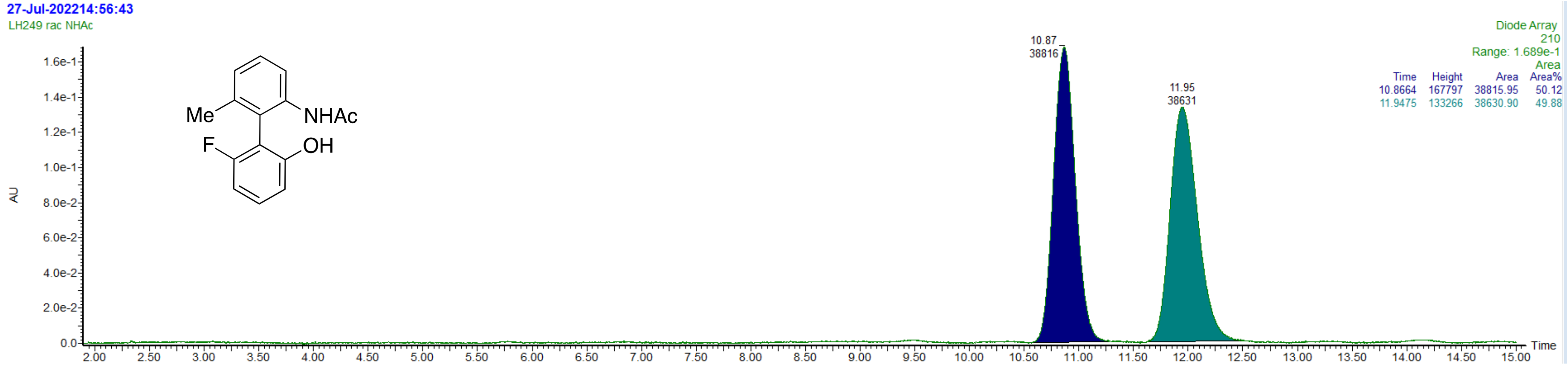


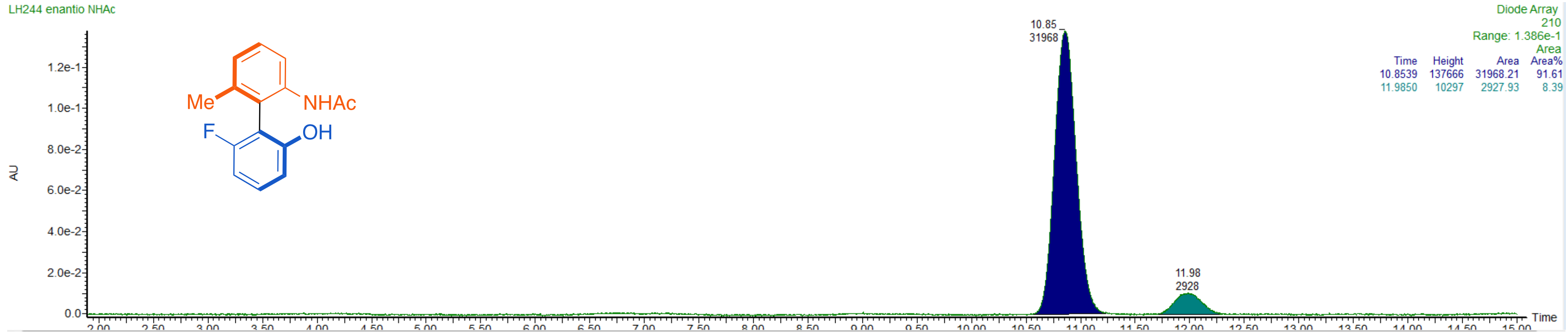

ethyl 5'-((3-chloro-4-methylphenyl)((trifluoromethyl)sulfonamido)methyl)-2'-methyl-[1,1'-biphenyl]-4-carboxylate (**9**)

**Chiral-SFC** (CHIRAL PAK IE($CO_2$:MeOH 92:8, 2.5 mL $min^{-1}$, 40 °C) $t_R$ = 4.84 (major), 5.49 (minor) minutes.

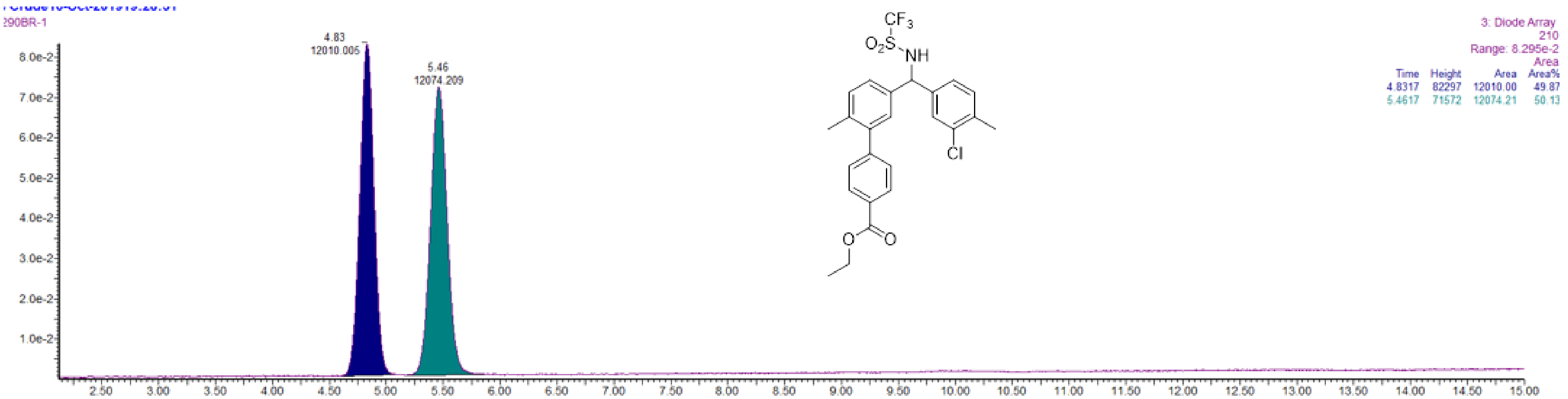


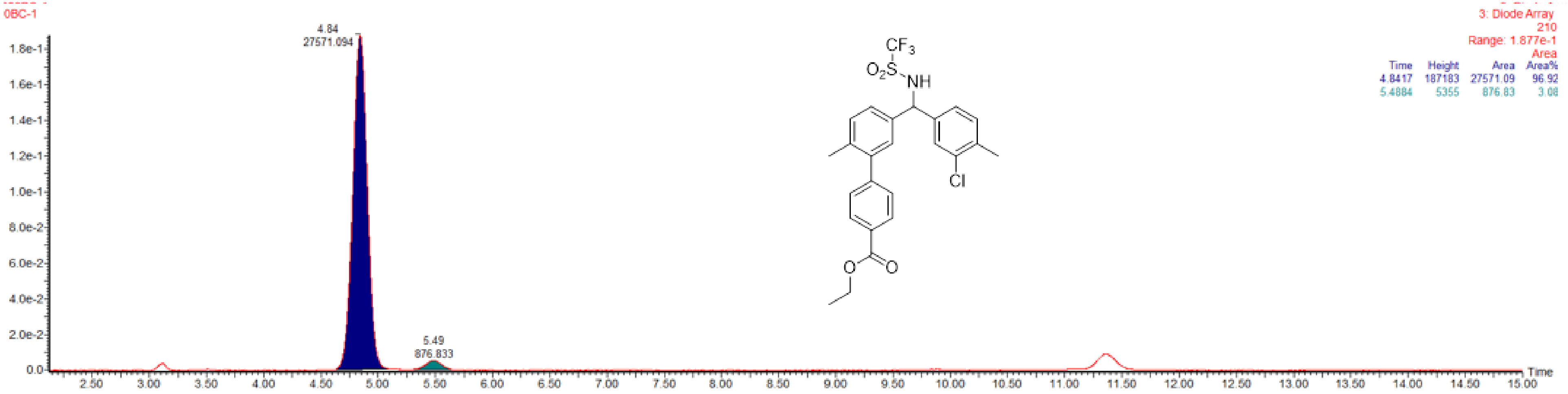

Ethyl (*R*)-2''-fluoro-3',6''-dimethoxy-[1,1':2',1''-terphenyl]-4-carboxylate (**16**)

**Chiral-SFC** (CHIRAL PAK IG (CO2:MeOH 96:4, 2.5 mL min-1, 40 °C) tR= 7.35 (minor), 8.65 (major) minutes.

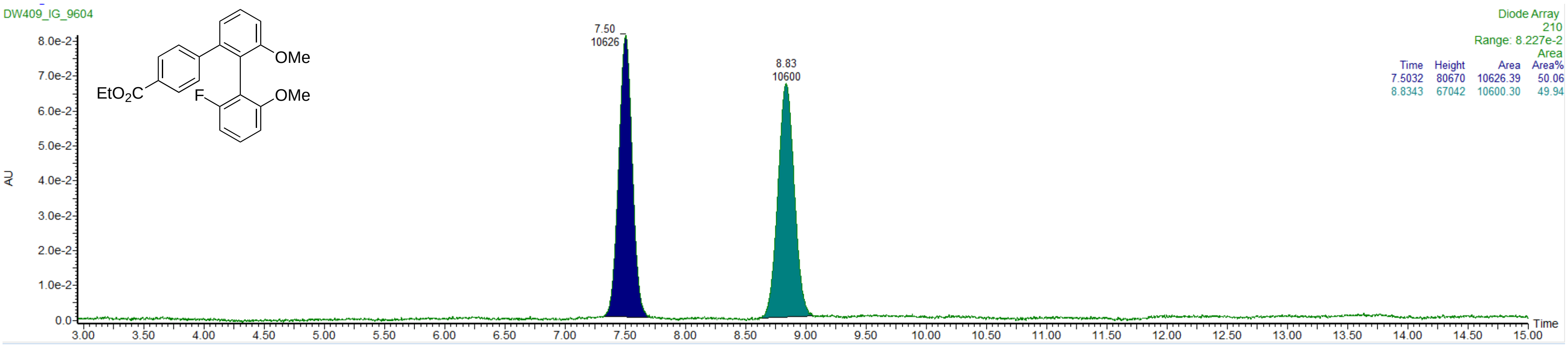


| Time | Height | Area | Area% |
|---|---|---|---|
| 7.5032 | 80670 | 10626.39 | 50.06 |
| 8.8343 | 67042 | 10600.30 | 49.94 |

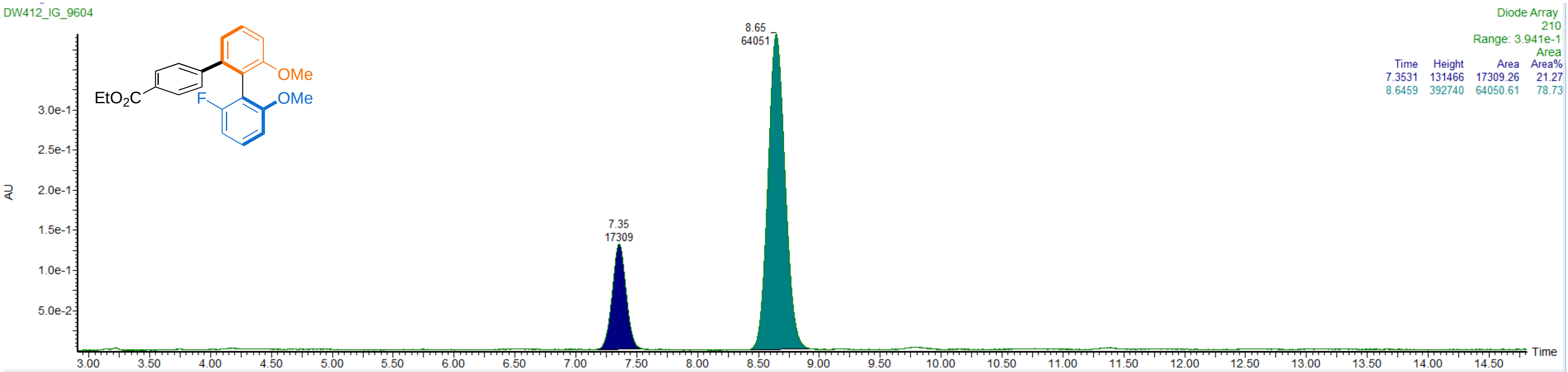


| Time | Height | Area | Area% |
|---|---|---|---|
| 7.3531 | 131466 | 17309.26 | 21.27 |
| 8.6459 | 392740 | 64050.61 | 78.73 |

neopentyl 2'-(dicyclohexylphosphoryl)-2,6-dimethoxy-[1,1'-biphenyl]-3-sulfonate

**Chiral-SFC** (CHIRAL ART SC($CO_2$:MeOH 82:18, 2.5 mL min$^{-1}$, 40 °C) $t_R$ = 7.95 (minor), 9.64 (major) minutes.
*Iterative recrystallisations of rac-sSPhos/quindinium salt*

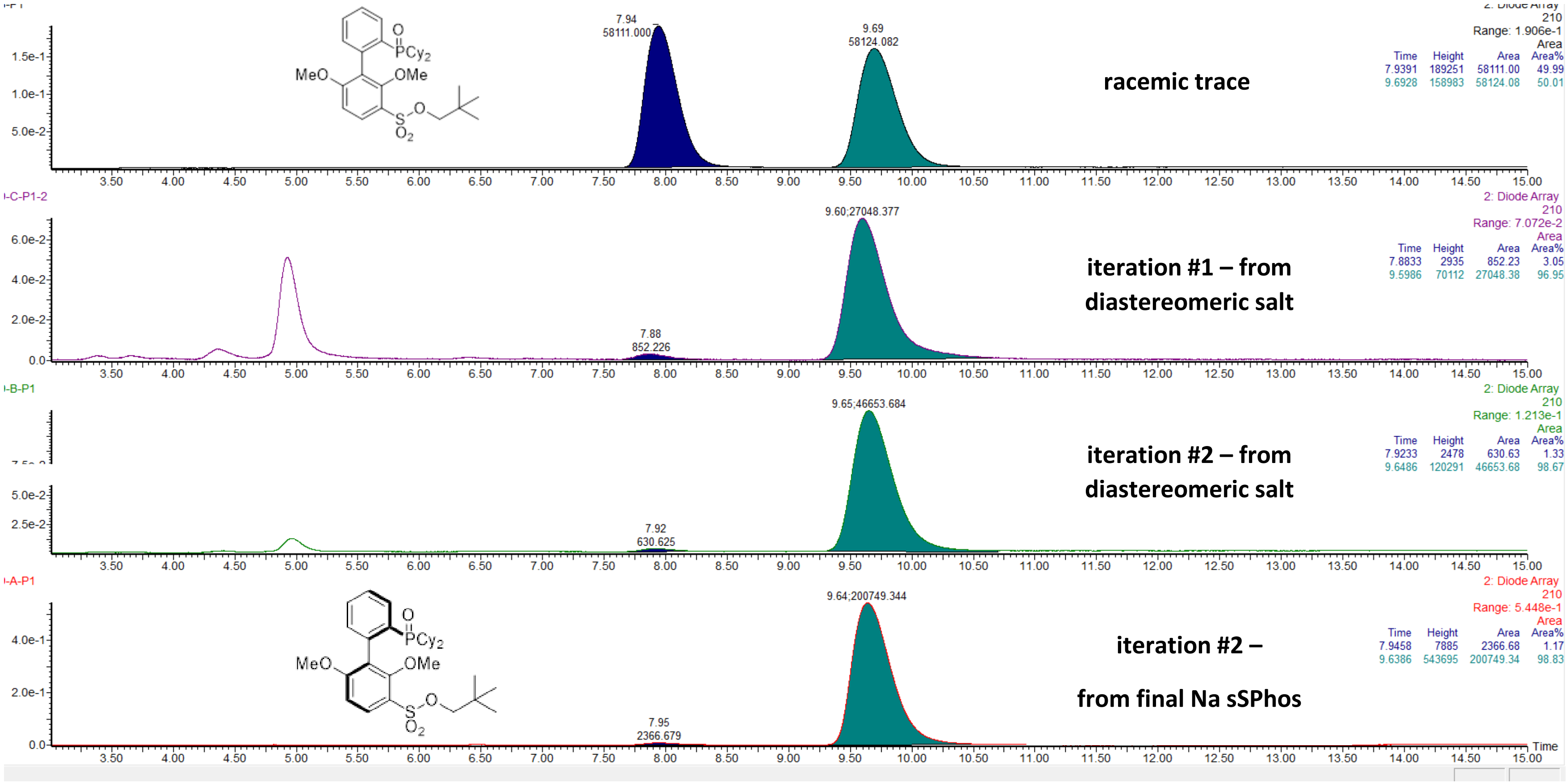

# References


1. K. Fujiwara, R. Motousu, D. Sato, Y. Kondo, U. Akiba, T. Suzuki, T. Tokiwano, Total synthesis of kehokorins A and B. *Tetrahedron Lett*. **60**, 1299–1301 (2019).

2. F. Shizuo, E. Hisao, O. Atsushi, O. Atsushi, N. Akiko, Halogenation Using N-Halogenocompounds. I. Effect of Amines on ortho-Bromination of Phenols with NBS. *B Chem Soc Jpn*. **66**, 1576–1579 (2006).

3. J. Shi, L. Li, C. Shan, Z. Chen, L. Dai, M. Tan, Y. Lan, Y. Li, Benzyne 1,2,4-Trisubstitution and Dearomative 1,2,4-Trifunctionalization. *J Am Chem Soc*. **143**, 10530–10536 (2021).

4. O. Shigenori, T. Tetsuya, K. Yoshihiro, G. Mika, Y. Shouzou, S. Masato, S. Masaki, Cannabinoid Receptor Modulator, Patent EP1637527A1 (2006).

5. Z. G. Chen, Y. Wang, J. F. Wei, P. F. Zhao, X. Y. Shi, K3PO4-catalyzed regiospecific aminobromination of βnitrostyrene derivatives with N-Bromoacetamide as aminobtominating agent. *J Org Chem*. **75**, 2085–2088 (2010).

6. Y. He, J. Tang, M. Luo, X. Zeng, Regioselective and Chemoselective Reduction of Naphthols Using Hydrosilane in Methanol: Synthesis of the 5,6,7,8-Tetrahydronaphthol Core. *Org Lett.* **20**, 4159–4163 (2018).

7. M. Berliner, K. Belecki, Synthesis of Alpha-Halo Ethers from Symmetric Acetals and in situ Methoxymethylation of an Alcohol. *Org Synth*. **84**, 102 (2007).

8. D. Augros, B. Yalcouye, A. Berthelot-Bréhier, M. Chessé, S. Choppin, A. Panossian, F. R. Leroux, Atropo-diastereoselective coupling of aryllithiums and arynes — variations around the chiral auxiliary. *Tetrahedron*. **72**, 5208–5220 (2016).

9. R. Sanz, M. P. Castroviejo, Y. Fernández, F. J. Fañanás, A new and efficient synthesis of 4-functionalized benzo[6]furans from 2,3-dihalophenols. *J Org Chem*. **70**, 6548–6551 (2005).

10. S. Chetty, T. Armstrong, S. Sharma Kharkwal, W. C. Drewe, C. I. de Matteis, D. Evangelopoulos, S. Bhakta, N. R. Thomas, New InhA Inhibitors Based on Expanded Triclosan and Di-Triclosan Analogues to Develop a New Treatment for Tuberculosis. *Pharmaceuticals 2021, Vol. 14, Page 361*. **14**, 361 (2021).

11. W. Li, G. Gao, Y. Gao, C. Yang, W. Xia, Direct oxidation of the C(sp2)–C(sp3) bond from benzyltrimethylsilanes to phenols. *Chem Comm*. **53**, 5291–5293 (2017).

12. Y. Liu, B. Kim, S. D. Taylor, Synthesis of 4-formyl estrone using a positional protecting group and its conversion to other C-4-substituted estrogens. *J. Org. Chem.* **72**, 8824–8830 (2007).

13. R. Yamano, J. Hara, K. Murayama, H. Sugiyama, K. Teraoka, H. Uekusa, S. Kawauchi, Y. Shibata, K. Tanaka, Rh-mediated enantioselective synthesis, crystal structures, and photophysical/chiroptical properties of phenanthrenol-based [9]helicene-like molecules. *Org Lett*. **19**, 42–45 (2017).

14. S. Duan, Y. Xu, X. Zhang, X. Fan, Synthesis of 2,2′-biphenols through direct C(sp2)–H hydroxylation of [1,1′-biphenyl]-2-ols. *Chem Comm*. **52**, 10529–10532 (2016).

15. J. Shi, L. Li, C. Shan, Z. Chen, L. Dai, M. Tan, Y. Lan, Y. Li, Benzyne 1,2,4-Trisubstitution and Dearomative 1,2,4-Trifunctionalization. *J Am Chem Soc*. **143**, 10530–10536 (2021).

16. A. A. Friedman, J. Panteleev, J. Tsoung, V. Huynh, M. Lautens, Rh/Pd catalysis with chiral and achiral ligands: Domino synthesis of aza-dihydrodibenzoxepines. *Angew Chem Int Edit*. **52**, 9755–9758 (2013).

17. E. Marzi, M. Schlosser, The site-selective functionalization of halogen-bearing phenols: An exercise in diversity-oriented organometallic synthesis. *Tetrahedron*. **61**, 3393–3401 (2005).

18. Y. Wang, L. Wang, L. Y. Chen, P. S. Bhadury, Z. Sun, Transition metal-free synthesis of pinacol arylboronate: Regioselective boronation of 1,3-disubstituted benzenes. *Aust J Chem*. **67**, 675–678 (2014).

19. K. W. Anderson, S. L. Buchwald, General catalysts for the Suzuki-Miyaura and Sonogashira coupling reactions of aryl chlorides and for the coupling of challenging substrate combinations in water. *Angew Chem Int Edit*. **44**, 6173–6177 (2005).

20. W. A. Golding, R. Pearce-Higgins, R. J. Phipps, Site-Selective Cross-Coupling of Remote Chlorides Enabled by Electrostatically Directed Palladium Catalysis. *J Am Chem Soc*. **140**, 13570–13574 (2018).

21. J. Rodriguez, H. H. Dhanjee, S. L. Buchwald, Amphiphilic Biaryl Monophosphine Ligands by Regioselective Sulfonation. *Org Lett*. **23**, 777–780 (2021).

22. Z. Che, J. Yang, D. Sun, Y. Tian, S. Liu, X. Lin, J. Jiang, G. Chen, Synthesis of Novel (9S)-Acyloxy Derivatives of Quinidine and Dihydroquinidine as Insecticidal Agents. *Chemistry & Biodiversity*. **17**, e1900696 (2020).
